# Desymmetrization of *meso*-bisphosphates using copper catalysis and alkylzirconocene nucleophiles

**DOI:** 10.1038/s41467-018-07871-x

**Published:** 2019-01-03

**Authors:** Reece Jacques, Robert D. C. Pullin, Stephen P. Fletcher

**Affiliations:** 1 Department of Chemistry, Chemistry Research Laboratory, University of Oxford, 12 Mansfield Road, Oxford, OX1 3TA UK; 2 Vertex Pharmaceuticals (Europe) Ltd., 86-88 Jubilee Avenue, Milton Park, Abingdon, Oxfordshire, OX14 4RW UK

## Abstract

The desymmetrization of *meso*-compounds is a useful synthetic method, as illustrated by numerous applications of this strategy in natural product synthesis. Cu-catalyzed allylic desymmetrizations enable the enantioselective formation of carbon-carbon bonds, but these transformations are limited in substrate scope and by the use of highly reactive premade organometallic reagents at cryogenic temperatures. Here we show that diverse *meso*-bisphosphates in combination with alkylzirconium nucleophiles undergo highly regio-, diastereo- and enantio-selective Cu-catalyzed desymmetrization reactions. In addition, *C*_2_-symmetric chiral bisphosphates undergo stereospecific reactions and a racemic substrate undergoes a Cu-catalyzed kinetic resolution. The reaction tolerates functional groups incompatible with many common organometallic reagents and provides access to a broad range of functionalized carbo- and hetero-cyclic structures. The products bear up to three contiguous stereogenic centers, including quaternary centers and spirocyclic ring systems. We anticipate that the method will be a useful complement to existing catalytic enantioselective reactions.

## Abstract (web summary)

Copper-catalyzed allylic desymmetrizations are, so far, limited to carbocyclic products with two tertiary stereocenters. Here, the authors report a highly enantioselective Cu-catalyzed desymmetrization of *meso* carbo- and hetero-cyclic bisphosphates to give products with up to three contiguous stereocenters.

## Introduction

The catalytic desymmetrization of *meso*-compounds is a powerful method of preparing chiral molecules. This strategy allows stereogenic features already present in symmetrical molecules to be unmasked, giving chiral molecules with multiple stereogenic centers in a single step^1–3^. These desymmetrizations have been used in the synthesis of enantiomerically enriched cyclic molecules, with metal-catalyzed asymmetric allylic addition (AAA) a proven strategy in C–C and C–X bond forming desymmetrizations^4–7^. Several natural product syntheses rely on a key desymmetrizing Pd-catalyzed AAA stage^8–12^. However, these processes are normally limited by the necessity to use stabilized nucleophiles (with pKa’s of less than 15), such as 1,3-dicarbonyl compounds, restricting the range of molecules that can be made using this strategy.

**Fig. 1:**
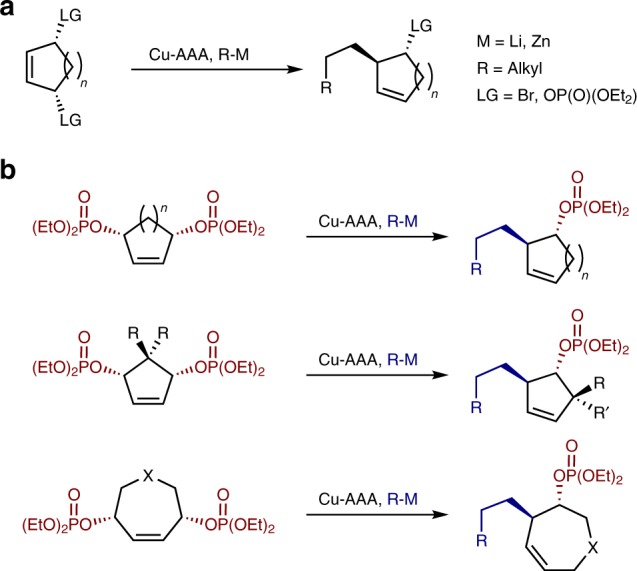
Copper-catalyzed desymmetrizations. **a** Previous Cu-catalyzed desymmetrizations of *meso*-cyclic substrates using unfunctionalized nucleophiles. **b** This work, using Cu-AAA and hydrozirconated alkenes to desymmetrize a wide array of *meso*-cyclic bisphosphates

Cu-catalyzed AAAs enable use of nonstabilized carbon nucleophiles^13–19^, but Cu-catalyzed C–C bond forming allylic desymmetrizations are rare and have only been reported using simple unfunctionalized nucleophiles (Fig. 1a). The Cu-catalyzed desymmetrization of *meso*-cyclic bisphosphates using dialkylzinc reagents by Gennari and co-workers^20,21^ proceeds with high enantioselectivity, but requires cryogenic temperatures and is limited to the addition of just Me, Et, and *i*Pr nucleophiles. Desymmetrizing AAA of cyclic bisphosphates using an allyl-nucleophile has been reported for two examples in 66 and 77% ee^22^. Recently, Feringa and co-workers reported highly enantioselective addition of alkyl lithium reagents to *meso*-dibromoalkenes, but this is again limited to additions of simple hydrocarbons^23^.

Thus far Cu-catalyzed C–C bond forming allylic desymmetrizations have only been applied to the simplest of *meso-*substrates to give carbocyclic products with two tertiary stereocenters. We envisaged that the scope of these transformations could be tremendously expanded to access diverse structures by exploiting the *meso*-symmetry of carbo- and heterocyclic diol derivatives and introduce additional stereocenters during desymmetrization. In order to achieve this, milder AAA conditions would be required, moving away from highly reactive organometallic reagents seen in previous methods. By using zirconocene nucleophiles we envisaged that much milder reaction conditions and greater compatibility with useful functional groups could be achieved^24–26^. In principle this would represent a simple, powerful and broadly useful strategy for the synthesis of highly complex molecules bearing functional groups and multiple stereogenic centers, including stereogenic features which are difficult to make, such as quaternary centers^27–33^.

Here, we demonstrate highly enantioselective Cu-catalyzed desymmetrization of diverse *meso*-cyclic bisphosphates using a wide range of in situ generated hydrozirconated alkene nucleophiles. We show the reaction can be applied to desymmetrize *meso*-cyclic bisphosphates bearing highly desirable quaternary centers and spirocycles to give products with up to three contiguous stereocenters. Pharmaceutically relevant heterocycles can also be accessed in an enantioselective fashion (Fig. 1b).

## Results

### Desymmetrization development

Initial experiments applied conditions from AAA’s with racemic allylic chlorides^34–36^ to the *meso*-cyclic bisdiethylphosphate **1a**; hydrozirconated 4-phenyl-1-butene in the presence of CuI and phosphoramidite ligand (*S*,*S*,*S*)-**A**^37^ (Table 1, entry 1). Good conversion to desymmetrized product **1b** was observed (71%), but as a racemate. The relative stereochemistry of product **1b** suggests the nucleophile adds via an S_N_2’ pathway, giving almost exclusively the *trans*-isomer (no S_N_2 product observed). The reaction proved sensitive to the copper source and in situ generated Lewis-acidic Cu(I) salts such as CuNTf_2_ gave up to 76% conversion and 69% ee using ligand **A** (entry 4). Attention then moved towards the effect of ligands.

**Table 1:** Optimization of desymmetrization reaction^a^


Entry	Cu salt	Ag salt^b^	Ligand	Yield (%)	ee (%)
1	Cu1	–	**A**	71^c^	0
2	CuClO_4_	–	**A**	52^c^	69
3	CuCl	AgOTf	**A**	64^c^	66
4	CuCl	AgNTf_2_	**A**	76^c^	69
5	CuCl	AgNTf_2_	**B**	61	65
6	CuCl	AgNTf_2_	**C**	61	75
7	CuCl	AgNTf_2_	**D**	67	83
8	CuCl	AgNTf_2_	**E**	76	84
9	CuCl	AgNTf_2_	**F**	70	86
10^d^	CuCl	AgNTf_2_	**F**	70	90
11^e^	CuCl	AgNTf_2_	**F**	27	92

^a^Reaction conditions: Alkene (2.5 eq.), Cp_2_ZrHCl (2.0 eq.), CH_2_Cl_2_, then, Cu salt (10 mol%), Ligand (12 mol%), Ag Salt (15 mol%), CH_2_Cl_2_, then **1a** (1.0 eq), 16 h. ee determined by HPLC. All products > 20:1 *trans*:*cis*

^b^Catalyst solution filtered to remove AgCl

^c^NMR yield

^d^0 °C

^e^–10 °C

Phosphoramidite ligands bearing a BINOL scaffold and indane moiety on nitrogen performed well in the reaction. Qualitatively, as the size of the ligand’s amido alkyl group increased so did the ee (entries 5–9). The best performing ligand was **F**^38,39^, giving 70% isolated yield, >20:1 *trans*:*cis* ratio and 86% ee (entry 9). A solvent screen revealed that this variable had little effect on the reaction, hence CH_2_Cl_2_ was retained. The dialkylphosphate group was also shown to have minimal impact on results (see Supplementary Figure 1). Lowering the temperature of the reaction increased the ee; up to 90% at 0 °C (entry 10). Lowering the temperature further (entry 11) resulted in diminished conversion, hence 0 °C was used to balance yield, ee and ease of reaction setup.

**Fig. 2:**
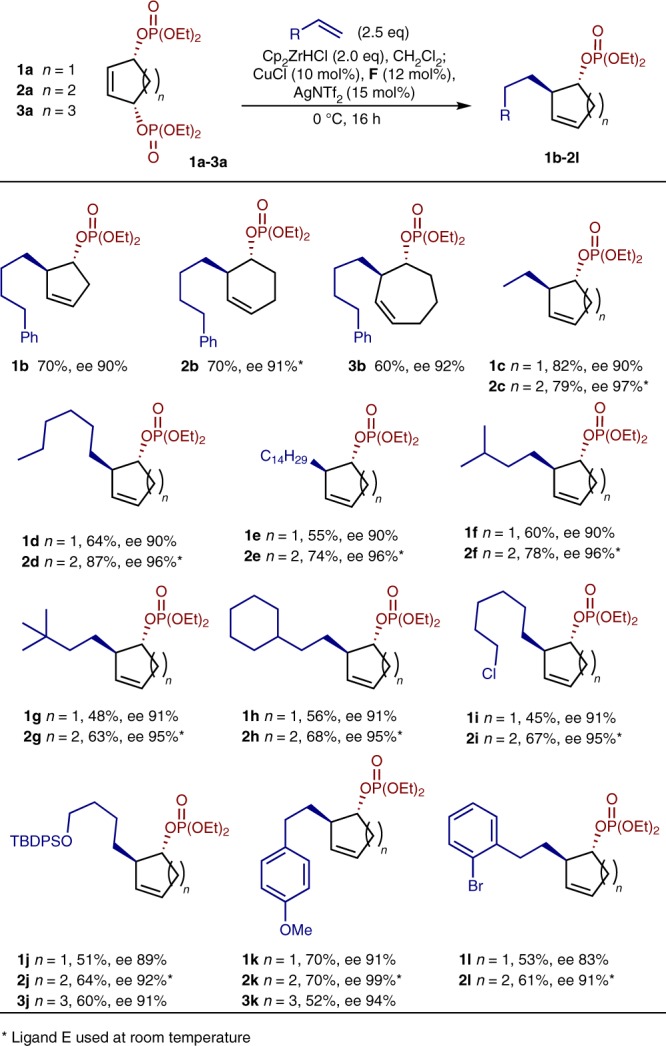
Initial reaction scope. Examination of alkene *pro*-nucleophiles and unsubstituted bisphosphates of different ring sizes

In order to show the reaction was general across different ring sizes, 6- and 7-membered *meso*-bisphosphates (**2a** and **3a**, respectively) were desymmetrized with 4-phenyl-1-butene. Both systems gave >90% ee (Fig. 2). A lower yield was observed for 6-membered **2b** (55% yield), hence the reaction was done at room temperature (70% yield). A range of alkene *pro*-nucleophiles were investigated in the desymmetrizations of **1a**–**3a**. Simple linear and branched alkenes performed well, consistently providing good yields and high enantioselectivity, with larger substituents such as *t*Bu reducing the yield. (**c**–**h**). Functionalized nucleophiles were investigated; a silyl protected alcohol and alkyl chloride was well tolerated (**i**, **j**). Styrenes also performed well in the reaction (**k**, **l**), with aryl bromides **1l** and **2l** allowing further manipulation if desired. A reaction using **1a** and ethylene to give 633 mg of **1c** on 4 mmol scale (1.48 g **1a**, 67%, 90% ee) illustrates the reaction is scalable.

**Fig. 3:**
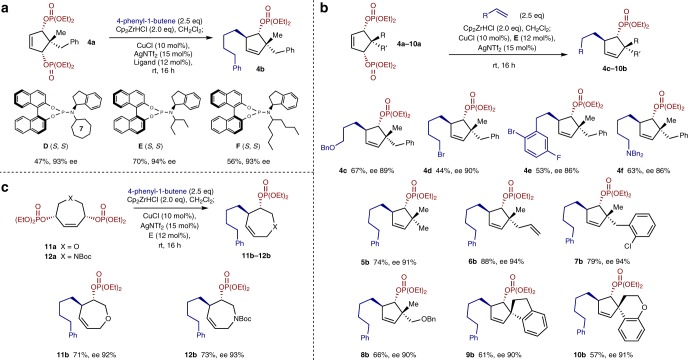
Further exploration of desymmetrization reaction. **a** Ligand optimization for electrophiles bearing a *pro*-quaternary center. **b** Substrate scope for electrophiles bearing a *pro*-quaternary center. **c** Desymmetrization of heterocyclic bisphosphates

We next explored the effect of further substitution on the bisphosphate electrophile, as this may provide products with additional stereogenic centers. Substrate **4a** with a methyl group *anti* to the phosphates and a benzyl group *syn* was prepared as a single diastereomer and subjected to the desymmetrization conditions with ligand **F** (Fig. 3a). The S_N_2’ addition product was obtained as the *trans* diastereomer (**4b**) in 56% yield and 93% ee. Here, the use of ligand **E** maintained the ee and improved the yield to 70%. Additionally, room temperature reactions gave high enantioselectivity in forming the quaternary center containing product. **4a** was then desymmetrized with nucleophiles bearing functional groups very likely incompatible with asymmetric addition procedures of alkyl zinc and lithium reagents (Fig. 3b). A benzyl protected alcohol (**4c**) and an alkyl bromide (**4d**) were well tolerated. In addition, an (arbitrarily) disubstituted styrene bearing aryl fluoride and bromide moieties (**4e**) was tolerated, as was the use of a basic nitrogen atom (**4f**), a group normally problematic in Cu-catalysis (63% yield, 86% ee).

A range of substituted bisphosphates bearing *pro*-quaternary centers (**5a**–**10a**), were prepared and examined (Fig. 3b). Useful functional groups such as allyl, and an aryl chloride were all well tolerated on the bisphosphate. These products are obtained as a single diastereoisomers in good yields with >90% ee (**5b**–**7b**). A substrate featuring a benzyl protected alcohol is similarly effective (61%, 90% ee, **8b**).

Spirocyclic systems, which can be challenging to prepare with either diastereo- or enantio-control^40–43^, performed well in the reaction effectively giving a single isomer product when both indane (**9b**) and chromane (**10b**) based ring systems were examined.

As well as carbocyclic structures, we envisaged that medicinally relevant 7-membered heterocyclic^44,45^ *meso*-bisphosphates could be accessed, allowing synthesis of substituted oxepenes and azepenes. Direct catalytic asymmetric manipulation of these ring systems to form new C–C bonds appears to be unexplored. In this case, it was not obvious that these reactions would be successful as tolerance to heteroatoms is often problematic in asymmetric Cu-catalyzed reactions^46^. However, we found that oxepene **11a** and Boc protected azepene **12a** performed very well under our desymmetrization conditions, and hydrometallation-addition of 4-phenyl-1-butene gave **11b** and **12b** in over 70% yield with >92% ee (Fig. 3c).

**Fig. 4:**
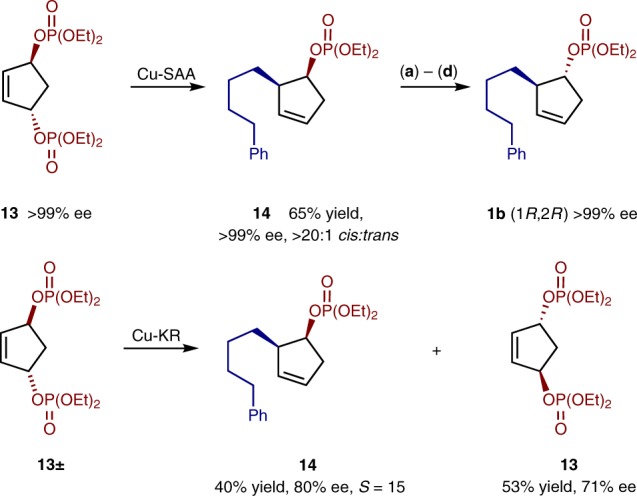
Cu-SAA/KR using *trans*-cyclic phosphates. Reaction conditions: Cu-SAA and Cu-KR see Table 1, entry 9; **a** LiAlH_4_ (2.2 eq), Et_2_O, rt; **b** HCO_2_H (2.05 eq), DIAD (2.0 eq), PPh_3_ (2.0 eq), Toluene 0 °C; **c** NEt_3_ (0.05 eq), MeOH, rt; **d** (EtO)_2_P(O)Cl (2.0 eq), NEt (2.0 eq), DMAP (0.33 eq), CH_2_Cl_2_, rt. Selectivity factor *S* calculated using *S* = ln[1 − *c*(1 + ee)]/ln[1 − *c*(1 − ee)]

Further stereocontrolled additions to bisphosphates were explored by examining *C*_2_-symmetric *trans*-cyclopentene epimers; enantiopure (*S*,*S*)-**13** and *rac-trans*-**13**. Cu-catalyzed kinetic resolutions of racemic starting material are also rare, and to the best of our knowledge there have been no reports of Cu-catalyzed asymmetric addition to cyclic *C*_*2*_-symmetric *trans*-diol derivatives. Both *trans*-bisphosphates were synthesized from the *trans*-diol, which can be prepared as a single enantiomer by enzymatic desymmetrization^47^. To our delight both substrates underwent stereospecific allylic alkylation (SAA). (Fig. 4). It was hypothesized that these SAA products would have *cis*-stereochemistry, rather than the *trans* exclusively obtained in the above *meso*-desymmetrization if the Cu-catalyst was mediating *anti*-S_N_2’ type processes.

Enantiopure (*S*,*S*)*-***13** stereospecifically reacts with hydrozirconated 4-phenyl-1-butene under desymmetrization conditions to give **14** in 65% yield, as a single isomer. Both the (*S*,*S*) and (*R*,*R*) forms of ligand **F** give the same product **14** in >20:1 *cis*:*trans* ratio with >99% ee. Inversion of the phosphate stereocenter of **14** in a multistep process gave (1*R*, 2*R*) **1b**, confirming the absolute configuration of **1b** produced in the enantioselective *meso*-desymmetrization. All other absolute configurations were assigned by analogy to **1b**.

Use of *rac-***13** led to a rare example of a Cu-catalyzed asymmetric kinetic resolution of racemic starting materials. Under desymmetrization conditions, if conversion is stopped at 43%, then **14** is obtained with 40% yield and 80% ee, and enantioenriched **13** is recovered in 53% yield with 71% ee.

**Fig. 5:**
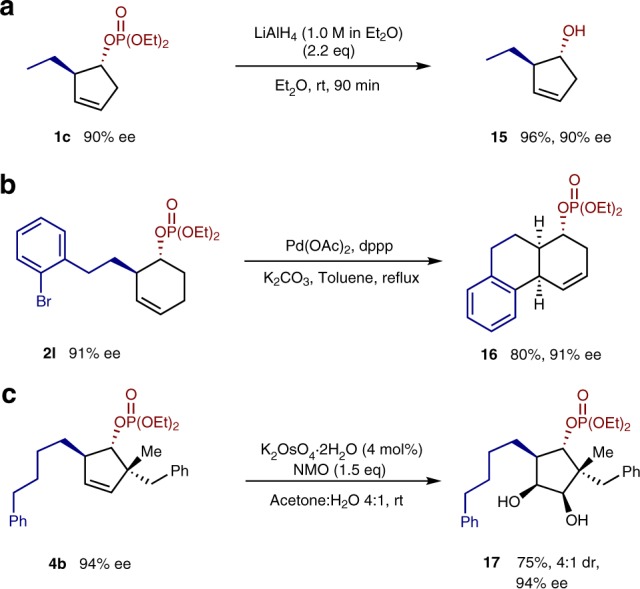
Further functionalization of products. **a** Reduction of the phosphate to give an alcohol. **b** Heck reaction to form tricyclic scaffold. **c** Alkene dihydroxylation to give a fully substituted cyclopentane

The downstream reactivity of the reaction products were briefly investigated to demonstrate that they could be further elaborated. (Fig. 5). Reduction of phosphate **1c** to give alcohol **15** proceeded in excellent yield. Aryl bromide **2l** was also able to undergo a Heck reaction to give *cis*-fused tricycle **16** in 80% yield. In addition, dihydroxylation of **4b** furnished **17**, a diol with five contiguous stereocenters, in 75% yield with 4:1 diastereoselectivity.

**Fig. 6:**
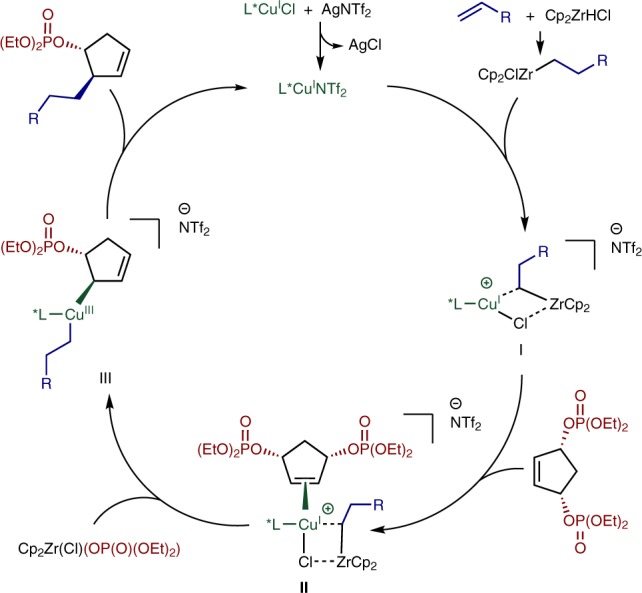
Postulated reaction mechanism. Catalytic cycle illustrating *anti*-S_N_2’ addition of alkylcopper species generated from alkylzirconium intermediates

Mechanistically, we tentatively postulate that a Cu-phosphoramidite complex forms a hybrid species **I** with the hydrozirconated alkene, which can co-ordinate to the bisphosphate and form an alkene donor complex (**II**). We suggest species **I** as in situ NMR spectroscopic studies of reactant and catalyst mixtures revealed no explicit transmetallation from Zr to Cu. **II** may react to form Cu-(III) σ-allyl species **III**, where the copper displaces the phosphate in a stereodetermining *anti*-S_N_2’ like process^16,48^. Reductive elimination of intermediate **III** would give the product and regenerate the Cu-phosphoramidite catalyst (Fig. 6).

In conclusion, we have developed a broadly applicable highly regio-, diastereo-, and enantio-selective desymmetrization of *meso*-cyclic bisphosphates using alkylzirconocene nucleophiles. A range of substituted bisphosphates can be used in the reaction, including heterocycles, and the protocol was also applied to *C*_2_-symmetric *trans*-cyclicbisphosphates. The products bear up to three stereocenters, including quaternary centers and spirocyclic ring systems.

## Methods

### General methods

For synthetic details and analytical data for all reaction products see Supplementary Methods.

### General procedure for enantioselective desymmetrization

Note: All manipulations are carried out under an Ar atmosphere and in the absence of light where possible. A flame-dried 5 mL round bottomed flask was charged with CuCl (4 mg, 0.04 mmol, 0.1 eq) and phosphoramidite ligand **E** (24.7 mg, 0.048 mmol, 0.12 eq). Dry CH_2_Cl_2_ (1.3 mL) was added to the mixture which was left to stir at room temperature for 1 h. AgNTf_2_ (23 mg, 0.06 mmol, 0.15 eq) was then added to the flask and left to stir at room temperature for 15 min. A pale brown suspension was formed. Meanwhile a separate 5 mL flame-dried round bottomed flask was charged with Cp_2_ZrHCl (206 mg, 0.8 mmol, 2.0 eq) and suspended in dry CH_2_Cl_2_ (0.8 mL). Alkene (1.0 mmol, 2.5 eq) was added to the mixture which was left to stir at room temperature until the mixture became a homogenous yellow solution (approx. 30 min, varies with alkene). The flask containing Cu-catalyst was transferred to the alkylzirconium reagent using a syringe equipped with a Camlab PTFE syringe filter (13 mm size, pore diameter 0.22 µm). The combined flask contents formed a black mixture which was left to stir at room temperature in the absence of light for 10 min. *Meso*-cyclic bisphosphate (0.4 mmol) in CH_2_Cl_2_ (0.2 mL) was added to the mixture which was left to stir at room temperature in the absence of light for 16 h. The mixture was quenched with 1 M aq. NH_4_Cl (1 mL) and left to stir for 10 min. The organic layer was separated, filtered through a plug of Celite and evaporated in vacuo to give an off-white mixture of solid and oil. The crude product was purified on silica to give the product.

## Supplementary Information

Supplementary Information

## Data Availability

All data supporting the findings of this study are available within the Article and its accompanying Supplementary Information file, which are both free of charge to access. For NMR spectra and HPLC/GC/SFC traces see Supplementary Figures 2–67.
