# Supplementary material for: Desymmetrization of meso-bisphosphates using copper catalysis and alkylzirconocene nucleophiles
Source: Nat Commun. 2019 Jan 3;10:21. doi: 10.1038/s41467-018-07871-x (PMC6318275; doi:10.1038/s41467-018-07871-x)
Supplement: Supplementary file 1 — Supplementary Information [file 41467_2018_7871_MOESM1_ESM.pdf]

Desymmetrization of *Meso*-Bisphosphates Using Copper Catalysis and Alkylzirconocene Nucleophiles

Reece Jacques, Robert D. C. Pullin and Stephen P. Fletcher*

Department of Chemistry, Chemistry Research Laboratory, University of Oxford, Mansfield Road, Oxford, OX1 3TA, UK.

Supplementary Information

Contents

Supplementary Table 1 - Dialkylphosphate Optimisation Table S1

General Information S2

Chemicals S3

Supplementary Methods S4

General procedure 1a: Racemic allylic alkylation products S4

General procedure 1b: Asymmetric allylic alkylation products S5

General procedure 1c: Asymmetric allylic alkylation products with quaternary centers S6

General procedure 2: Phosphate reduction and esterification for HPLC analysis S7

General procedure 3: Oxidation of diones S8

General procedure 4: Luche reduction of dienones S8

General procedure 5: Phosphorylation of diols via anion approach S9

Experimental Procedures and Characterisation of Compounds S10

Starting Materials S10

Cis-4-cyclopentene-1,3-diol S10

Cis-5-cyclopentene-1,4-diol S11

Cis-6-cyclopentene-1,5-diol S12

Meso-4-cyclopentene-1,3-bisdimethylphosphate S13

Meso-4-Cyclopentene-1,3-bisdiethylphosphate (**1a**) S13

Meso-4-Cyclopentene-1,3-bisdiisopropylphosphate	S14
Meso-4-Cyclopentene-1,3-bisdiphenylphosphate	S15
Meso-5-Cyclohexene-1,4-bisdiethylphosphate (**2a**)	S16
Meso-6-Cycloheptene-1,5-bisdiethylphosphate (**3a**)	S17
(1*S*,3*S*)-Cyclopent-4-ene-1,3-diyl tetraethyl bis(phosphate) (**13**)	S18
2,2-Dimethylcyclopentane-1,3-dione	S21
2-Allyl-2-methylcyclopentane-1,3-dione	S22
2-Benzyl-2-methylcyclopentane-1,3-dione	S19
2-(2-Chlorobenzyl)-2-methylcyclopentane-1,3-dione	S23
2-((Benzyloxy)methyl)-2-methylcyclopentane-1,3-dione	S23
2',3'-Dihydrospiro[cyclopentane-1,1'-indene]-2,5-dione	S24
Spiro[chromane-4,1'-cyclopentane]-2',5'-dione	S25
2,2-Dimethylcyclopent-4-ene-1,3-dione	S25
2-Allyl-2-methylcyclopent-4-ene-1,3-dione	S26
2-Benzyl-2-methylcyclopent-4-ene-1,3-dione	S26
2-(2-Chlorobenzyl)-2-methylcyclopent-4-ene-1,3-dione	S27
2-((Benzyloxy)methyl)-2-methylcyclopent-4-ene-1,3-dione	S27
2',3'-Dihydrospiro[cyclopent-4-ene-1,1'-indene]-2,5-dione	S28
Spiro[chromane-4,1'-cyclopent-4-ene]-2',5'-dione	S28
(1*R*,2*S*,3*S*)-2-Benzyl-2-methylcyclopent-4-ene-1,3-diol	S29
Cis-2,2-dimethylcyclopent-4-ene-1,3-diol	S30

(1*R*,2*S*,3*S*)-2-Allyl-2-methylcyclopent-4-ene-1,3-diol S30

(1*R*,2*S*,3*S*)-2-(2-Chlorobenzyl)-2-methylcyclopent-4-ene-1,3-diol S31

(1*R*,2*S*,3*S*)-2-((Benzyloxy)methyl)-2-methylcyclopent-4-ene-1,3-diol S32

(1*S*,2*R*,5*S*)-2',3'-Dihydrospiro[cyclopentane-1,1'-inden]-3-ene-2,5-diol S33

(2'*R*,4*S*,5'*S*)-Spiro[chromane-4,1'-cyclopentan]-3'-ene-2',5'-diol S34

(1*R*,2*S*,3*S*)-2-Benzyl-2-methylcyclopent-4-ene-1,3-bis(diethyl phosphate) (**4a**) S35

Cis-2,2-dimethylcyclopent-4-ene-1,3-bis(diethyl phosphate) (**5a**) S36

(1*R*,2*S*,3*S*)-2-Allyl-2-methylcyclopent-4-ene-1,3-bis(diethyl phosphate) (**6a**) S37

(1*R*,2*S*,3*S*)-2-(2-Chlorobenzyl)-2-methylcyclopent-4-ene-1,3-bis(diethyl phosphate) (**7a**) S38

(1*R*,2*S*,3*S*)-2-((Benzyloxy)methyl)-2-methylcyclopent-4-ene-1,3-bis(diethyl phosphate) (**8a**) S39

(1*S*,2*R*,5*S*)-2',3'-Dihydrospiro[cyclopentane-1,1'-inden]-3-ene-2,5-bis(diethyl phosphate) (**9a**) S40

(2'*R*,4*S*,5'*S*)-Spiro[chromane-4,1'-cyclopentan]-3'-ene-2',5'-bis(diethyl phosphate) (**10a**) S41

1,1'-Oxybis(but-3-en-2-ol) S42

Cis-2,3,6,7-tetrahydrooxepine-3,6-diol S43

Tetraethyl ((3*R*,6*S*)-2,3,6,7-tetrahydrooxepine-3,6-diyl) bis(phosphate) (**11a**) S43

tert-Butyl (3*R*,4*S*)-3,4-dihydroxypyrrolidine-1-carboxylate S44

tert-Butyl bis(2-hydroxybut-3-en-1-yl)carbamate S45

tert-Butyl (3*R*,6*S*)-3,6-dihydroxy-2,3,6,7-tetrahydro-1*H*-azepine-1-carboxylate S46

tert-Butyl (3*R*,6*S*)-3,6-bis((diethoxyphosphoryl)oxy)-2,3,6,7-tetrahydro-1*H*-azepine-1-carboxylate (**12a**) S46

Desymmetrization Products S48

Dimethyl ((1*R*,2*R*)-2-(4-phenylbutyl)cyclopent-3-en-1-yl) phosphate S48

Diethyl ((1*R*,2*R*)-2-(4-phenylbutyl)cyclopent-3-en-1-yl) phosphate (**1b**) S49

Diisopropyl ((1*R*,2*R*)-2-(4-phenylbutyl)cyclopent-3-en-1-yl) phosphate S50

Diphenyl ((1*R*,2*R*)-2-(4-phenylbutyl)cyclopent-3-en-1-yl) phosphate S51

Diethyl ((1*R*,2*R*)-2-(4-phenylbutyl)cyclohex-3-en-1-yl) phosphate (**2b**) S53

Diethyl ((1*R*,2*R*)-2-(4-phenylbutyl)cyclohept-3-en-1-yl) phosphate (**3b**) S54

Diethyl ((1*R*,2*R*)-2-ethylcyclopent-3-en-1-yl) phosphate (**1c**) S55

Diethyl ((1*R*,2*R*)-2-ethylcyclohex-3-en-1-yl) phosphate (**2c**) S56

Diethyl ((1*R*,2*R*)-2-hexylcyclopent-3-en-1-yl) phosphate (**1d**) S57

Diethyl ((1*R*,2*R*)-2-hexylcyclohex-3-en-1-yl) phosphate (**2d**) S59

Diethyl ((1*R*,2*R*)-2-tetradecylcyclopent-3-en-1-yl) phosphate (**1e**) S60

Diethyl ((1*R*,2*R*)-2-tetradecylcyclohex-3-en-1-yl) phosphate (**2e**) S61

Diethyl ((1*R*,2*R*)-2-isopentylcyclopent-3-en-1-yl) phosphate (**1f**) S62

Diethyl ((1*R*,2*R*)-2-isopentylcyclohex-3-en-1-yl) phosphate (**2f**) S63

Diethyl (1*R*,2*R*)-2-(3,3-dimethylbutyl)cyclopent-3-en-1-yl) phosphate (**1g**) S64

Diethyl (1*R*,2*R*)-2-(3,3-dimethylbutyl)cyclohex-3-en-1-yl) phosphate (**2g**) S65

Diethyl (1*R*,2*R*)-2-(2-cyclohexylethyl)cyclopent-3-en-1-yl phosphate (**1h**) S67

Diethyl (1*R*,2*R*)-2-(2-cyclohexylethyl)cyclohex-3-en-1-yl phosphate (**2h**) S68

(1*R*,2*R*)-2-(6-chlorohexyl)cyclopent-3-en-1-yl diethyl phosphate (**1i**) S69

(1*R*,2*R*)-2-(6-chlorohexyl)cyclohex-3-en-1-yl diethyl phosphate (**2i**) S70

(1*R*,2*R*)-2-(4-((*tert*-butyldiphenylsilyl)oxy)butyl)cyclopent-3-en-1-yl diethyl phosphate (**1j**) S71

(1*R*,2*R*)-2-(4-((*tert*-butyldiphenylsilyl)oxy)butyl)cyclohex-3-en-1-yl diethyl phosphate (**2j**) S73

(1*R*,2*R*)-2-(4-((*tert*-butyldiphenylsilyl)oxy)butyl)cyclohept-3-en-1-yl diethyl phosphate (**3j**) S74

Diethyl ((1*R*,2*R*)-2-(4-methoxyphenethyl)cyclopent-3-en-1-yl) phosphate (**1k**) S76

Diethyl ((1*R*,2*R*)-2-(4-methoxyphenethyl)cyclohex-3-en-1-yl) phosphate (**2k**) S77

Diethyl ((1*R*,2*R*)-2-(4-methoxyphenethyl)cyclohept-3-en-1-yl) phosphate (**3k**) S78

(1*R*,2*R*)-2-(2-bromophenethyl)cyclopent-3-en-1-yl diethyl phosphate (**1l**) S79

(1*R*,2*R*)-2-(2-bromophenethyl)cyclohex-3-en-1-yl diethyl phosphate (**2l**) S81

(1*S*,2*R*,5*R*)-2-benzyl-2-methyl-5-(4-phenylbutyl)cyclopent-3-en-1-yl diethyl phosphate (**4b**) S83

(1*S*,2*R*,5*R*)-2-Benzyl-5-(3-(benzyloxy)propyl)-2-methylcyclopent-3-en-1-yl diethyl phosphate (**4c**) S84

(1*S*,2*R*,5*R*)-2-Benzyl-5-(4-bromobutyl)-2-methylcyclopent-3-en-1-yl diethyl phosphate (**4d**) S85

1*S*,2*R*,5*R*)-2-Benzyl-5-(2-bromo-5-fluorophenethyl)-2-methylcyclopent-3-en-1-yl diethyl phosphate (**4e**) S87

(1*S*,2*R*,5*R*)-2-Benzyl-5-(4-(dibenzylamino)butyl)-2-methylcyclopent-3-en-1-yl diethyl phosphate (**4f**) S88

(1*S*,5*R*)-2,2-Dimethyl-5-(4-phenylbutyl)cyclopent-3-en-1-yl diethyl phosphate (**5b**) S90

(1*S*,2*R*,5*R*)-2-Allyl-2-methyl-5-(4-phenylbutyl)cyclopent-3-en-1-yl diethyl phosphate (**6b**) S91

(1*S*,2*R*,5*R*)-2-(2-chlorobenzyl)-2-methyl-5-(4-phenylbutyl)cyclopent-3-en-1-yl diethyl phosphate (**7b**) S93

(1*S*,2*S*,5*R*)-2-((benzyloxy)methyl)-2-methyl-5-(4-phenylbutyl)cyclopent-3-en-1-yl diethyl phosphate (**8b**) S94

Diethyl ((1*S*,4*R*,5*S*)-4-(4-phenylbutyl)-2',3'-dihydrospiro[cyclopentane-1,1'-inden]-2-en-5-yl) phosphate (**9b**) S96

Diethyl ((4S,4'R,5'S)-4'-(4-phenylbutyl)spiro[chromane-4,1'-cyclopentan]-2'-en-5'-yl) phosphate (**10b**) S97

Diethyl ((3*S*,4*R*)-4-(4-phenylbutyl)-2,3,4,7-tetrahydrooxepin-3-yl) phosphate (**11b**) S99

tert-Butyl (3*S*,4*R*)-3-((diethoxyphosphoryl)oxy)-4-(4-phenylbutyl)-2,3,4,7-tetrahydro-1*H*-azepine-1-carboxylate (**12b**) S100

Diethyl ((1*S*,2*R*)-2-(4-phenylbutyl)cyclopent-3-en-1-yl) phosphate (**14**) S102

Derivatization Products S104

(1*R*,2*R*)-2-ethylcyclopent-3-en-1-ol (**15**) S104

Diethyl ((1R,4aR,10aR)-1,4,4a,9,10,10a-hexahydrophenanthren-1-yl) phosphate (**16**) S105

(1*S*,2*R*,3*R*,4*S*,5*R*)-2-benzyl-3,4-dihydroxy-2-methyl-5-(4-phenylbutyl) cyclopentyl diethyl phosphate (**17**) S106

Supplementary Figures 2 – 67 S108

Supplementary References S227

Supplementary Table 1 - Dialkylphosphate Optimisation Table

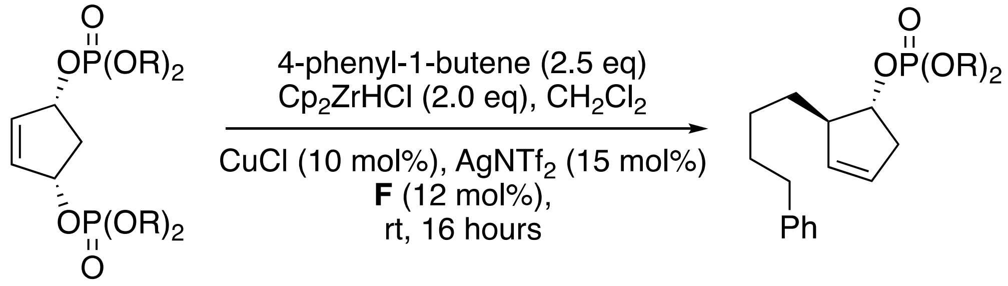

R	Yield (%)	ee (%)
Me	55	86
*i*Pr	45	84
Ph	20	70
Et	70	86

General Information

Procedures using oxygen and/or moisture-sensitive materials were performed with anhydrous solvents under an atmosphere of anhydrous argon in flame-dried flasks, using standard Schlenk techniques. Analytical thin-layer chromatography was performed on precoated glass-backed plates (Silica Gel 60 F254; Merck), and visualized using a combination of UV light (254 nm) and anisaldehyde solution or aqueous basic potassium permanganate stain. Flash column chromatography was carried out using Apollo Scientific silica gel 60 (0.040 – 0.063 nm), Merck 60 Å silica gel, VWR (40-63 μm) silica gel and Sigma Aldrich silica gel. Pressure was applied at the column head via hand bellows or a flow of nitrogen with the solvent system used in parentheses.

Reactions at 0 ˚C were performed using an ice-water bath, covered with cotton wool and aluminium foil if overnight stirring is needed. Other temperatures were obtained using a Julabo FT902 immersion cooler or the heating plate of the stirrer.

Unless stated otherwise, solution NMR spectra were recorded at room temperature; ^{1} H, ^{31}P and ^{13}C NMR experiments were carried out using Bruker AVIII HD 400 (400/101/162 MHz) or AVIII HD 500 (500/126/202 MHz) spectrometers. Chemical shifts are reported in ppm from the residual solvent peak. Chemical shifts (δ) are given in ppm and coupling constants (J) are quoted in hertz (Hz). Resonances are described as s (singlet), d (doublet), t (triplet), q (quartet) and m (multiplet). Assignments were made with the assistance of COSY, HSQC, HMBC or NOESY NMR spectra.

Chiral HPLC separations were achieved using an Agilent 1230 Infinity series normal phase HPLC unit and HP Chemstation software. Chiral SFC separations were achieved using a Waters Acquity UPC^{2}™ unit. Chiralpak® columns (250 × 4.6 mm), fitted with matching Chiralpak® Guard Cartridges (10 × 4 mm), were used as specified in the text. Solvents used were of HPLC grade (Fisher Scientific, Sigma Alrich or Rathburn); all eluent systems were isocratic.

Chiral SFC (supercritical fluid chromatography) separations were conducted on a Waters Acquity UPC2 system using Waters Empower software. Chiralpak® columns (150 × 3 mm, particle size 3 μm) were used as specified in the text. Solvents used were of HPLC grade (Fisher Scientific, Sigma Aldrich or Rathburn). Compounds **14e**, **14f** and **22** used specific instruments and conditions detailed later in the text.

Chiral GC measurements were conducted on an Agilent 7820A GC (He as a vector gas) with the stated column in the characterisation. Temperature programs are described as follows: initial temperature (°C) – initial time (min) – temperature gradient (°C/min) – final temperature (°C) – holding time (min). Flow rate is given in mL/min. Retention times (R_T) are given in min.

Low-resolution mass spectra were recorded using a Walters LCT premier XE. High-resolution mass spectra (EI and ESI) were recorded using a Bruker MicroTOF spectrometer by the internal service at the University of Oxford. Infrared measurements (neat, thin film) were carried out using a Bruker Tensor 27 FT-IR with internal calibration in the range 600-4000 cm^{-1}. Optical rotations were recorded on a Perkin-Elmer 241 polarimeter at 25°C in a 10 cm cell in the stated solvent; $[\alpha]_D$ values are given in 10^{-1} $deg.cm^2$ g^{-1} (concentration c given as g/100 mL).

Chemicals

Dry THF and CH_2Cl_2 were collected fresh from an mBraun SPS-5 solvent purification system having been passed through anhydrous alumina columns. All other dry solvents used were dried over 3 Å molecular sieves and stored under argon. All other solvents were used as purchased from Sigma Aldrich, Honeywell or Fisher Scientific. Unless stated otherwise, commercially available reagents were purchased from Sigma-Aldrich, Fisher Scientific, Apollo Scientific, Acros Organics, Strem Chemicals, Alfa Aesar or TCI UK and were used without purification. Petroleum ether refers to light petroleum boiling in the range 40-60 °C. Deuterated solvents were purchased from Sigma-Aldrich ($CDCl_3$). PPL refers to Lipase from porcine pancreas, Type II, 100-500 units/mg of protein, purchased from Sigma-Aldrich (Cat no. L3126). Schwartz reagent was prepared according to the literature procedure[1] from Cp_2ZrCl_2 purchased from Acros or Strem Chemicals.

Supplementary Methods

General procedure 1a: Racemic allylic alkylation products

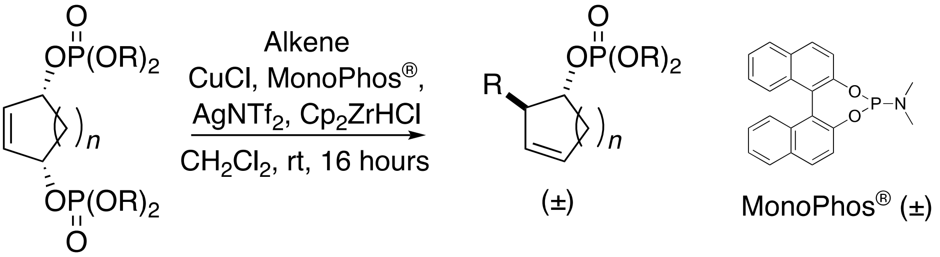

Note: All manipulations are carried out under an Ar atmosphere and in the absence of light where possible. Additional details on the procedure are described here: *Nature Protoc.*, 2014, **9**, 104-111. Procedures can be carried out without flame drying, instead using oven dried glassware and N_2 purging without detriment to yield or ee.

Flask **A**: A flame-dried 5 mL round bottomed flask was charged with CuCl (4 mg, 0.04 mmol, 0.1 eq) and racemic (3,5-dioxo-4-phosphacyclohepta[2,1-a:3,4-a']dinaphthalen-4-yl)dimethylamine (MonoPhos®) (17.2 mg, 0.048 mmol, 0.12 eq). Dry CH_2Cl_2 (1.5 mL) was added to the mixture which was left to stir at room temperature for 1 hour. $AgNTf_2$ (23 mg, 0.06 mmol, 0.15 eq) was then added to the flask and left to stir at room temperature for 15 mins. A pale brown suspension was formed.

Flask **B**: Meanwhile a separate flame-dried round bottomed flask was charged with Cp_2ZrHCl (206 mg, 0.8 mmol, 2.0 eq) and suspended in dry CH_2Cl_2 (0.8 mL). Alkene (1.0 mmol, 2.5 eq) was added to the mixture which was left to stir at room temperature in the absence of light, until the mixture became a homogenous yellow solution (approx. 30 mins, varies with alkene). Note: The alkylzirconium species can be left for up to 2-3 hours without issue.

The contents of flask **A** were transferred into flask **B** using a syringe equipped with a Camlab PTFE syringe filter (13 mm size, pore diameter 0.22 µm) to remove excess AgCl. The combined flask contents formed a black mixture which was left to stir at room temperature in the absence of light for 10 mins. *Meso*-cyclic bisphosphate (**2a-**

c) (0.4 mmol) was added to the mixture which was left to stir at room temperature in the absence of light for 16 hours.

The mixture was quenched with 1M aq. NH_4Cl (1 mL) and left to stir for 10 mins. The organic layer was separated, filtered through a plug of Celite and evaporated *in vacuo* to give an off-white mixture of solid and oil. The crude product was purified on silica (eluent specified below) to give a yellow oil.

General procedure 1b: 5 and 7-membered asymmetric allylic alkylation products

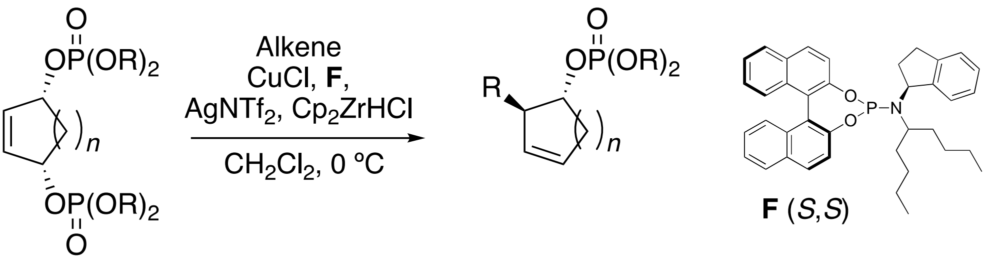

Note: All manipulations are carried out under an Ar atmosphere and in the absence of light where possible. Additional details on the procedure are described here: *Nature Protoc.*, 2014, **9**, 104-111. Procedures can be carried out without flame drying, instead using oven dried glassware and N_2 purging without detriment to yield or ee.

Flask **A**: A flame-dried 5 mL round bottomed flask was charged with CuCl (4 mg, 0.04 mmol, 0.1 eq) and phosphoramidite ligand **F** (28 mg, 0.048 mmol, 0.12 eq). Dry CH_2Cl_2 (1.5 mL) was added to the mixture which was left to stir at room temperature for 1 hour. $AgNTf_2$ (23 mg, 0.06 mmol, 0.15 eq) was then added to the flask and left to stir at room temperature for 15 mins. A pale brown suspension was formed.

Flask **B**: Meanwhile a separate flame-dried round bottomed flask was charged with Cp_2ZrHCl (206 mg, 0.8 mmol, 2.0 eq) and suspended in dry CH_2Cl_2 (0.8 mL). Alkene (1.0 mmol, 2.5 eq) was added to the mixture which was left to stir at room temperature in the absence of light, until the mixture became a homogenous yellow solution (approx. 30 mins, varies with alkene). Note: The alkylzirconium species can be left for up to 2-3 hours without issue.

The contents of flask **A** were transferred into flask **B** using a syringe equipped with a Camlab PTFE syringe filter (13 mm size, pore diameter 0.22 µm) to remove excess AgCl. The combined flask contents formed a black mixture which was left to stir at 0 °C in the absence of light for 10 mins. *Meso*-cyclic bisphosphate (**2a-c**) (0.4 mmol) was added to the mixture which was left to stir at 0 °C in the absence of light for 16 hours.

The mixture was quenched with 1M aq. NH_4Cl (1 mL) and left to stir for 10 mins. The organic layer was separated, filtered through a plug of Celite and evaporated *in vacuo* to give an off-white mixture of solid and oil. The crude product was purified on silica (eluent specified below) to give a yellow oil.

General procedure 1c: Asymmetric allylic alkylation products with quaternary centers, 6-membered rings and heterocycles

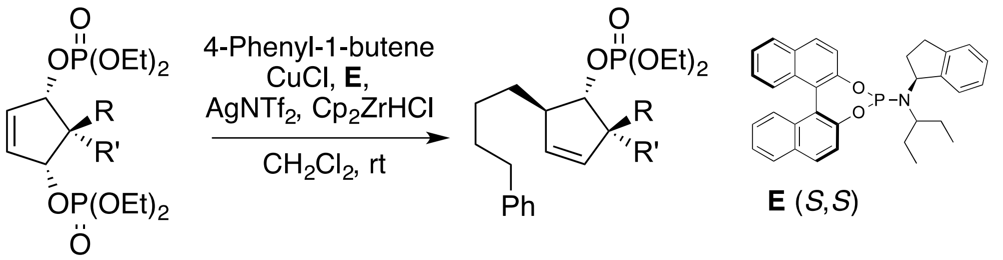

Note: All manipulations are carried out under an Ar atmosphere and in the absence of light where possible. Additional details on the procedure are described here: *Nature Protoc.*, 2014, **9**, 104-111.

Flask **A**: A flame-dried 5 mL round bottomed flask was charged with CuCl (4 mg, 0.04 mmol, 0.1 eq) and phosphoramidite ligand **E** (24.7 mg, 0.048 mmol, 0.12 eq). Dry CH_2Cl_2 (1.3 mL) was added to the mixture which was left to stir at room temperature for 1 hour. $AgNTf_2$ (23 mg, 0.06 mmol, 0.15 eq) was then added to the flask and left to stir at room temperature for 15 mins. A pale brown suspension was formed.

Flask **B**: Meanwhile a separate flame-dried round bottomed flask was charged with Cp_2ZrHCl (206 mg, 0.8 mmol, 2.0 eq) and suspended in dry CH_2Cl_2 (0.8 mL). Alkene

(1.0 mmol, 2.5 eq) was added to the mixture which was left to stir at room temperature in the absence of light, until the mixture became a homogenous yellow solution (approx. 30 mins, varies with alkene). Note: The alkylzirconium species can be left for up to 2-3 hours without issue.

The contents of flask **A** were transferred into flask **B** using a syringe equipped with a Camlab PTFE syringe filter (13 mm size, pore diameter 0.22 µm) to remove excess AgCl. The combined flask contents formed a black mixture which was left to stir at room temperature in the absence of light for 10 mins. *Meso*-cyclic bisphosphate (**4a-12a**) (0.4 mmol) in CH_2Cl_2 (0.2 mL) was added to the mixture which was left to stir at room temperature in the absence of light for 16 hours.

The mixture was quenched with 1M aq. NH_4Cl (1 mL) and left to stir for 10 mins. The organic layer was separated, filtered through a plug of Celite and evaporated *in vacuo* to give an off-white mixture of solid and oil. The crude product was purified on silica (eluent specified below) to give a yellow oil.

General procedure 2: Phosphate reduction and esterification for HPLC analysis

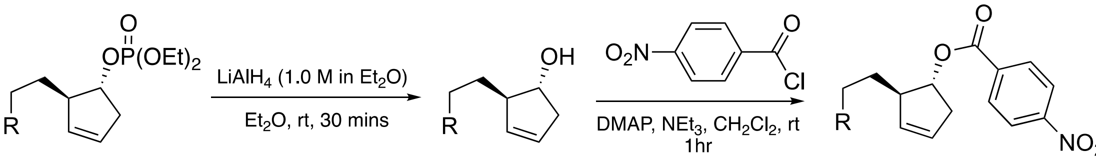

Phosphate (0.1 mmol, 1.0 eq) was dissolved in Et_2O (1.0 mL) and cooled to 0 ˚C before adding $LiAlH_4$ (1.0 M in Et_2O, 0.6 mL, 0.6 mmol, 6 eq) dropwise. The solution was warmed to room temperature and left to stir for 30 mins. The reaction mixture was quenched with H_2O dropwise until effervescence stopped. The residue was suspended in CH_2Cl_2 and filtered through celite, washing with CH_2Cl_2. The filtrate was evaporated *in vacuo* to give the crude alcohol.

The alcohol (0.1 mmol) was dissolved in CH_2Cl_2 (2.0 mL) before adding NEt_3 (28 µL, 0.2 mmol, 2.0 eq), DMAP (5 mg, 0.04 mmol, 0.4 eq) and *p*-nitrobenzoyl chloride (37 mg, 0.2 mmol, 2.0 eq). The mixture was left to stir for 1 hour, and was then

evaporated *in vacuo* to give a yellow residue. The residue was purified on silica (5% EtOAc in petroleum ether) to give a colourless oil.

General procedure 3: Oxidation of diones

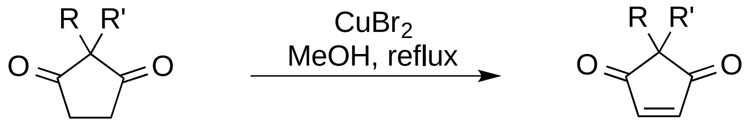

Under Ar dione (15 mmol, 1.0 eq) was dissolved in anhydrous MeOH (100 mL) before addition of $CuBr_2$ (7.37 g, 33.0 mmol, 2.2 eq). The mixture was heated to reflux and stirred for 1 hour. The resulting black mixture was cooled, quenched with dropwise addition of H_2O (25 ml) and 1M HCl (50 mL). The mixture was concentrated *in vacuo* to remove MeOH. The resulting mixture was extracted with EtOAc (3 x 75 mL) and the organic extracts were combined, washed with brine (2 x 50 mL), dried ($MgSO_4$) and concentrated *in vacuo*. The crude material was purified on silica (eluent specified below) to give the dienone product. **NOTE**: All examples presented give bright yellow products.

General procedure 4: Luche reduction of dienones

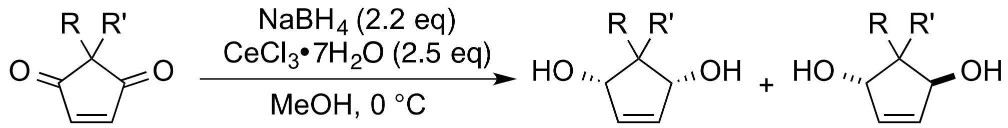

Dienone (5.00 mmol, 1.0 eq) was dissolved in MeOH (60 mL, not dry) and stirred until dissolution. $CeCl_3{\cdot}7H_2O$ (4.66 g, 12.50 mmol, 2.5 eq) was added and stirred until dissolution. The resulting solution was cooled to 0 ˚C before portionwise addition of $NaBH_4$ (416 mg, 11 mmol, 2.2 eq) over 10 minutes. The resulting suspension was left to stir at 0 ˚C for 2 hours before slow quenching with H_2O (5 ml) and 1M HCl (10 mL). The mixture was concentrated *in vacuo* to remove MeOH. The resulting mixture was extracted with EtOAc (3 x 30 mL) and the organic extracts were combined, washed with brine (2 x 30 mL), dried ($MgSO_4$) and concentrated *in vacuo*. The crude material was purified on silica (eluent specified below) to give the

diol product. **NOTE**: The examples presented gave separable 1:1 *cis*/*trans* mixtures unless stated otherwise. The *cis* isomer was isolated and characterised in all cases.

General procedure 5: Phosphorylation of diols via a dianion approach

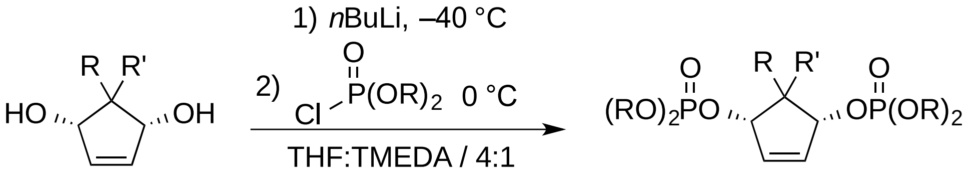

A solution of diol (10.0 mmol, 1.0 eq.) in THF (60 mL) and TMEDA (15 mL) was cooled to -40 ˚C using a MeCN/CO_2 bath before *n*BuLi (2.5 M in hexane, 8.80 mL, 22.0 mmol, 2.2 eq.) was added dropwise. The resulting solution was left to stir for 10 minutes at -40 ˚C before dialkyl chlorophosphate (25.0 mmol, 2.5 eq.) was added dropwise. The resulting mixture was left to stir at -40 ˚C for 2 hours and then warmed to 0 °C. Brine (10 mL) was added slowly to the mixture which was then poured over H_2O (80 mL) and extracted with CH_2Cl_2 (3 x 80 mL). The organic extracts were combined, washed with brine (100 mL), dried ($MgSO_4$) and evaporated *in vacuo*. The crude material was purified on silica (eluent specified below) to give the diphosphate product.

Experimental Procedures and Characterisation of Compounds

Starting Materials

Unsubstituted Diphosphates

Cis-4-cyclopentene-1,3-diol

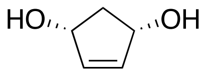

Chemical Formula: $C_5H_8O_2$
Molecular Weight: 100.1170

Cyclopentadiene (2.00 mL, 24.3 mmol, 1.0 eq.), thiourea (1.84g, 24.3 mmol, 1.0 eq.) and Rose Bengal (50 mg, 0.048 mmol, 0.002 eq.) were added to a round-bottomed flask containing methanol (400 mL). O_2 gas was bubbled through the resulting pink solution for 15 mins before being maintained under an atmosphere of O_2. The flask was partially submersed in an ice bath and irradiated with a 500 W halogen lamp with stirring for 8 hours (**Supplementary Figure 1**). The lamp was turned off and the mixture was left to stir in the dark at room temperature for 16 hours. The mixture was evaporated *in vacuo* to give a viscous pink residue. The residue was dissolved in water (250 mL) and extracted with Et_2O (3 x 200 mL). The aqueous layer was evaporated *in vacuo* to give a pink residue, which was dissolved in methanol (50 mL) and loaded onto Chem Tube-Hydromatrix for purification on silica (1% MeOH in EtOAc) to give *cis*-4-cyclopentene-1,3-diol as a white solid (1.72 g, 71% yield).

^{1}H NMR (400 MHz, DMSO-d_6) δ_H/ppm: 5.75 (s, 2H), 4.84 (dd, 2H, J = 7.8, 2.0 Hz), 2.96-2.85 (m, 2H), 1.28-1.21 (m, 2H); ^{13}C NMR (101 MHz, $CDCl_3$) δ_C/ppm: 137.0, 75.7, 44.2. Consistent with data in the literature.[2]

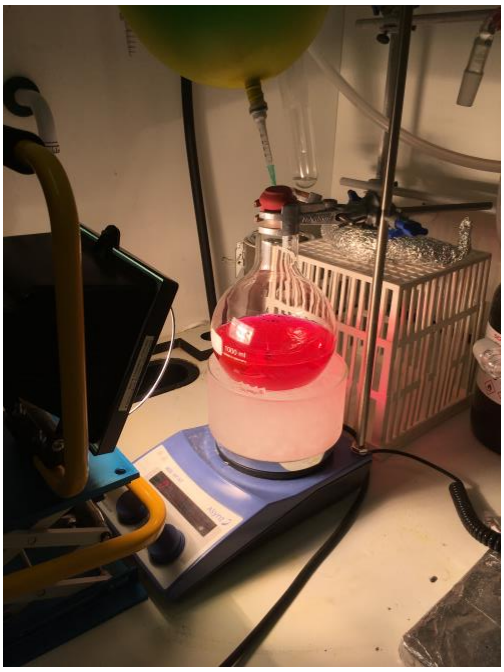

Supplementary Figure 1. Setup of photo-oxidation, illustrating partial submersion of flask in an ice bath.

***Cis*-5-cyclohexene-1,4-diol**

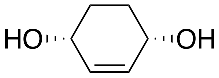

Chemical Formula: $C_6H_{10}O_2$
Molecular Weight: 114.1440

1,3-Cyclohexadiene (2.50 mL, 26.2 mmol, 1.0 eq.) was dissolved in CH_2Cl_2 (105 mL) before adding *meso*-tetraphenylporphyrin (56 mg, 0.09 mmol, 0.0035 eq.). O_2 gas was bubbled through the resulting purple solution for 15 mins before being maintained under an atmosphere of O_2. The flask was partially submersed in an ice bath and irradiated with a 500 W halogen lamp with stirring for 8 hours (**Supplementary Figure 1**). The reaction mixture was then evaporated *in vacuo* to give a purple residue which was then dissolved in MeOH (105 mL) and thiourea (2.01 g, 26.5 mmol, 1.01 eq.) was added. The mixture was left to stir in the dark at room temperature for 16 hours. The mixture was then evaporated *in vacuo* to give a viscous, dark brown residue which was dissolved in methanol (50 mL) and loaded onto Chem Tube-Hydromatrix for purification on silica (2% MeOH in Et_2O), to give *cis*-5-cyclohexene-1,4-diol as a white solid (2.28 g, 76% yield).

^{1}H NMR (400 MHz, DMSO-d_6) δ_H/ppm: 5.75 (s, 2H), 4.07 (br. s, 2H), 3.90 (br. s, 2H), 1.78-1.71 (m, 4H); ^{13}C NMR (101 MHz, $CDCl_3$) δ_C/ppm: 132.6, 64.1, 28.6. Consistent with data in the literature.[3]

***Cis*-6-cycloheptene-1,5-diol**

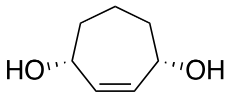

Chemical Formula: $C_7H_{12}O_2$
Molecular Weight: 128.1710

1,3-Cycloheptadiene (1.00 g, 210.6 mmol, 1.0 eq.) was dissolved in CH_2Cl_2 (80 mL) before adding *meso*-tetraphenylporphyrin (23 mg, 0.04 mmol, 0.0035 eq.). O_2 gas was bubbled through the resulting purple solution for 15 mins before being maintained under an atmosphere of O_2. The flask was partially submersed in an ice bath and irradiated with a 500 W halogen lamp with stirring for 8 hours (**Supplementary Figure 1**). The reaction mixture was then evaporated *in vacuo* to give a purple residue which was then dissolved in MeOH (80 mL) and thiourea (816 mg, 10.7 mmol, 1.01 eq.) was added. The mixture was left to stir in the dark at room temperature for 16 hours. The mixture was then evaporated *in vacuo* to give a viscous, dark brown residue which was dissolved in methanol (50 mL) and loaded onto Chem Tube-Hydromatrix for purification on silica (2-3% MeOH in CH_2Cl_2), to give *cis*-6-cycloheptene-1,5-diol as a white solid (576 mg, 42% yield).

^{1}H NMR (400 MHz, $CDCl_3$) δ_H/ppm: 5.76 (s, 2H), 4.30 (d, 2H, J = 8.0 Hz), 2.10-2.00 (m, 1H) 1.78-1.73 (m, 2H), 1.75 (br. s, 1H), 1.72-1.56 (m, 4H); ^{13}C NMR (101 MHz, $CDCl_3$) δ_C/ppm: 135.8, 71.4, 36.0, 23.1. Consistent with data in the literature.[4]

Meso-4-cyclopentene-1,3-bisdimethylphosphate

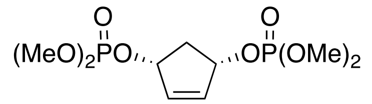

Chemical Formula: $C_9H_{18}O_8P_2$
Molecular Weight: 316.1825

Synthesised according to general procedure 5 using *cis*-4-cyclopentene-1,3-diol on 1.0 mmol scale. Purified by flash chromatography (4% MeOH in CH_2Cl_2) to give *meso*-4-cyclopentene-1,3-bisdimethylphosphate as a pale yellow oil (207 mg, 65% yield).

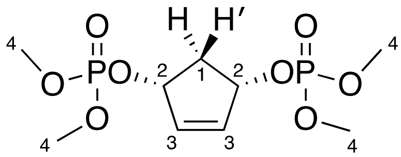

^{1}H NMR (400 MHz, $CDCl_3$) δ_H/ppm: 6.15 (s, 2H, 2CH-(3)), 5.27 – 5.18 (m, 2H, 2CH-(2)), 3.77 (d, *J* = 11.1 Hz, 12H, $4CH_3$-(4)), 2.98 – 2.85 (m, 1H, C***H***H′-(1)), 2.10 – 1.99 (m, 1H, CH***H′***-(1)); ^{13}C NMR (101 MHz, $CDCl_3$) δ_C/ppm: 135.1 (C(3)), 79.4 (C(2)), 54.3 (C(4)), 39.5 (C1)); ^{31}P NMR (162 MHz, $CDCl_3$) δ_P/ppm: 0.29; HRMS (ESI) *m/z* calcd for $C_9H_{18}O_8P_2Na$ $[M+Na]^+$: 339.0369, found: 339.0365; IR (ATR) ν (cm^{-1}) thin film, $CHCl_3$: 2980.7 (w), 1461.7 (w), 1377.8 (w), 1266.4 (m), 1185.8 (w), 1037.9 (s), 991.8 (s), 934.3 (w), 849.5 (m), 751.8 (w).

Meso-4-Cyclopentene-1,3-bisdiethylphosphate (1a)

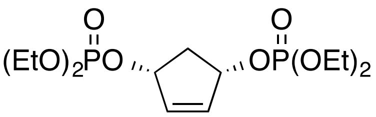

1a

Chemical Formula: $C_{13}H_{26}O_8P_2$
Molecular Weight: 372.2905

To a solution of *cis*-4-cyclopentene-1,3-diol (3.15 g, 31.5 mmol, 1.0 eq) in CH_2Cl_2 (142 mL) was added NEt_3 (13.2 mL, 94.4 mmol, 3.0 eq) and DMAP (1.92 g, 15.8 mmol, 0.5 eq). The resulting solution was cooled to 0 °C before dropwise addition of

diethylchlorophosphate (13.7 mL, 94.4 mmol, 3.0 eq). The resulting mixture was left to stir at room temperature for 16 hours. The mixture was then quenched with H_2O (100 mL) and extracted with CH_2Cl_2 (3 x 100 mL). The organic extracts were combined, washed with brine (100 mL), dried ($MgSO_4$) and evaporated *in vacuo* to give a yellow oil. The yellow oil was purified by flash chromatography (3% MeOH in EtOAc) to give *meso*-4-cyclopentene-1,3-bisdiethylphosphate (**1a**) as a pale yellow oil (11.23g, 96% yield).

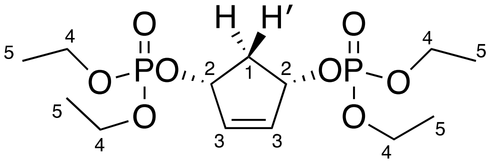

^{1}H NMR (400 MHz, $CDCl_3$) δ_H/ppm: 6.12 (d, J = 0.8 Hz, 2H, 2CH-(3)), 5.21 (d, J = 4.4 Hz, 2H, 2CH-(2)), 4.10 (m, J = 8.3, 7.1, 1.3 Hz, 8H, 4CH_2-(4)), 2.91 (dt, J = 14.6, 7.3 Hz, 1H, C***H***H´-(1)), 2.03 (dt, J = 14.6, 4.3 Hz, 1H, CH***H*´**-(1)), 1.33 (t, J = 7.1 Hz, 12H, 4CH_3-(5)); ^{13}C NMR (101 MHz, $CDCl_3$) δ_C/ppm: 135.2 (C(3)), 79.3 (C(2)), 64.0 (C(4)), 39.7 (C(1)), 16.3 (C(5)); ^{31}P NMR (162 MHz, $CDCl_3$) δ_P/ppm: -1.91; HRMS (ESI) *m/z* calcd for $C_{13}H_{26}O_8P_2Na$ $[M+Na]^+$: 395.0995, found: 395.0993; IR (ATR) ν (cm^{-1}) thin film, $CHCl_3$: 2982.8 (w), 1373.3 (w), 1261.4 (m), 1165.5 (w), 1024.0 (s), 980.7 (s), 802.9 (w), 749.9 (w). Consistent with data in the literature.[5]

***Meso*-4-Cyclopentene-1,3-bisdiisopropylphosphate**

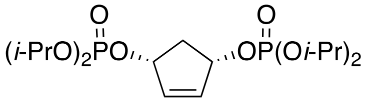

Chemical Formula: $C_{17}H_{34}O_8P_2$
Molecular Weight: 428.3985

Synthesised according to general procedure 5 using *cis*-4-cyclopentene-1,3-diol on 1.50 mmol scale. Purified by flash chromatography (3% MeOH in CH_2Cl_2) to give *meso*-4-cyclopentene-1,3-bisdiisopropylphosphate as a pale yellow oil (456 mg, 71% yield).

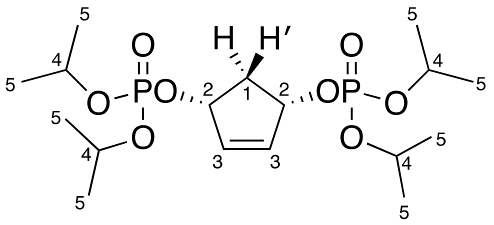

^{1}H NMR (400 MHz, $CDCl_3$) δ_H/ppm: 6.09 (s, 2H, 2CH-(3)), 5.16 (q, *J* = 6.4 Hz, 2H, 2CH-(2)), 4.61 (m, *J* = 12.3, 6.7, 6.2 Hz, 4H, 4CH-(4)), 2.88 (dt, *J* = 14.6, 7.3 Hz, 1H, C***H***H´-(1)), 2.00 (dt, *J* = 14.5, 4.6 Hz, 1H, CH***H´***-(1)), 1.31 (d, *J* = 6.2 Hz, 24H, 8CH_3-(5)); ^{13}C NMR (101 MHz, $CDCl_3$) δ_c/ppm: 135.4 (C(3)), 79.5 (C(2)), 73.0 (C(4)), 40.2 (C(1)), 24.2 (C(5)); ^{31}P NMR (162 MHz, $CDCl_3$) δ_P/ppm: -3.50; HRMS (ESI) *m/z* calcd for $C_{17}H_{34}O_8P_2Na$ $[M+Na]^+$: 451.1621, found: 451.1618; IR (ATR) ν (cm^{-1}) thin film, $CHCl_3$: 2981.0 (w), 1467.8 (w), 1376.3 (w), 1259.8 (m), 1179.3 (w), 1142.8 (w), 1108.6 (w), 979.5 (s), 928.5 (m), 779.3 (w), 729.3 (w).

***Meso*-4-Cyclopentene-1,3-bisdiphenylphosphate**

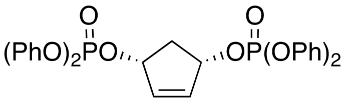

Chemical Formula: $C_{29}H_{26}O_8P_2$
Molecular Weight: 564.4665

Synthesised according to general procedure 5 using *cis*-4-cyclopentene-1,3-diol on 1.0 mmol scale. Purified by flash chromatography (EtOAc:Petroleum Ether, 1:1, using silica which was pre-washed with 2% NEt_3 in petroleum ether) to give *meso*-4-cyclopentene-1,3-bisdiphenylphosphate as a yellow oil (462 mg, 82% yield).

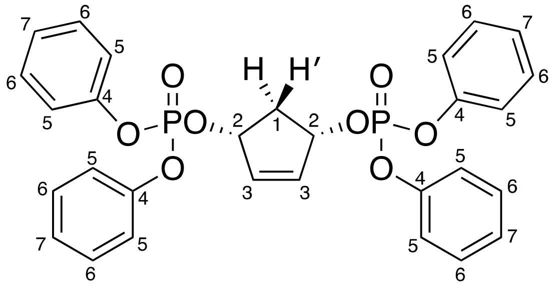

^{1}H NMR (400 MHz, $CDCl_3$) δ_H/ppm: 7.36 – 7.31 (m, 8H, ArH-(5)), 7.23 – 7.16 (m, 12H, ArH-(6,7)), 6.11 (s, 2H, 2CH-(3)), 5.46 – 5.42 (m, 2H, 2CH-(2)), 2.89 (dt, *J* =

14.7, 7.2 Hz, 1H, C***H***H′-(1)), 2.08 (dt, J = 14.9, 3.9 Hz, 1H, CH***H***′-(1)); ^{13}C NMR (101 MHz, $CDCl_3$) δ_C/ppm: 150.5 (C(4)), 135.3 (C(3)), 129.9 (C(6)), 125.6 (C(5)), 120.2 (C(7)), 80.9 (C(2)), 39.3 (C(1)); ^{31}P NMR (162 MHz, $CDCl_3$) δ_P/ppm: -12.74; HRMS (ESI) *m/z* calcd for $C_{29}H_{26}O_8P_2Na$ $[M+Na]^+$: 587.0995, found: 587.0988; IR (ATR) ν (cm^{-1}) thin film, $CHCl_3$: 2980.6 (w), 1589.6 (w), 1486.9 (m), 1573.2 (w), 1284.1 (m), 1218.5 (w), 1186.1 (s), 1161.8 (m), 1071.3 (w), 990.1 (m), 942.4 (s), 754.8 (s), 687.8 (m), 616.7 (w).

***Meso*-5-Cyclohexene-1,4-bisdiethylphosphate (2a)**

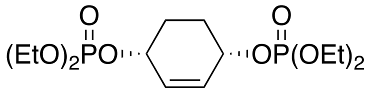

2a

Chemical Formula: $C_{14}H_{28}O_8P_2$
Molecular Weight: 386.3175

To a solution of *cis*-5-cyclohexene-1,4-diol (2.20 g, 19.3 mmol, 1.0 eq) in CH_2Cl_2 (87 mL) was added NEt_3 (8.06 mL, 57.8 mmol, 3.0 eq) and DMAP (1.18 g, 9.64 mmol, 0.5 eq). The resulting solution was cooled to 0 °C before dropwise addition of diethylchlorophosphate (8.36 mL, 57.8 mmol, 3.0 eq). The resulting mixture was left to stir at room temperature for 16 hours. The mixture was then quenched with H_2O (100 mL) and extracted with CH_2Cl_2 (3 x 100 mL). The organic extracts were combined, washed with brine (100 mL), dried ($MgSO_4$) and evaporated *in vacuo* to give a yellow oil. The yellow oil was purified by flash chromatography (3% MeOH in EtOAc) to give *meso*-5-cyclohexene-1,4-bisdiethylphosphate (**2a**) as a yellow oil (6.56 g, 88% yield).

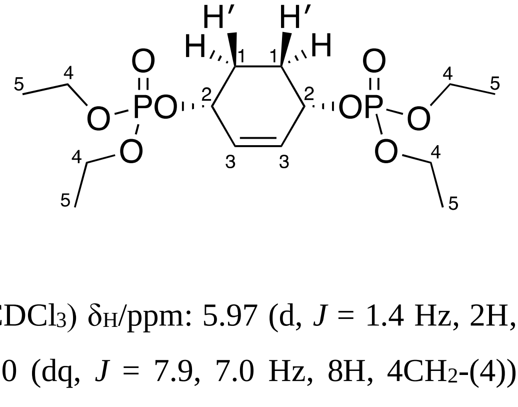

^{1}H NMR (400 MHz, $CDCl_3$) δ_H/ppm: 5.97 (d, J = 1.4 Hz, 2H, 2CH-(3)), 4.92 – 4.70 (m, 2H, 2CH-(2)), 4.10 (dq, J = 7.9, 7.0 Hz, 8H, $4CH_2$-(4)), 2.07 – 1.99 (m, 2H, 2C***H***H′-(1)), 1.98 – 1.89 (m, 2H, 2CH***H***′-(1)), 1.34 (td, J = 7.0, 1.0 Hz, 12H, $4CH_3$-

(5)); ^{13}C NMR (101 MHz, $CDCl_3$) δ_C/ppm: 130.7 (C(3)), 70.9 (C(2)), 63.8 (C(4)), 26.1 (C(1)), 16.2 (C(5)); ^{31}P NMR (162 MHz, $CDCl_3$) δ_P/ppm: -1.38; HRMS (ESI) *m/z* calcd for $C_{14}H_{28}O_8P_2$ $[M+H]^+$: 387.1338, found: 387.1379; IR (ATR) ν (cm^{-1}) thin film, $CHCl_3$: 2985.2 (w), 1644.4 (w), 1396.8 (w), 1261.4 (m), 1164.2 (w), 1027.0 (s), 989.3 (s), 817.6 (w). Consistent with data in the literature.[5]

***Meso*-6-Cycloheptene-1,5-bisdiethylphosphate (3a)**

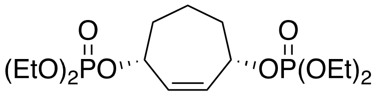

3a

Chemical Formula: $C_{15}H_{30}O_8P_2$
Molecular Weight: 400.3445

To a solution of *cis*-6-cycloheptene-1,5-diol (400 mg, 3.12 mmol, 1.0 eq) in CH_2Cl_2 (14 mL) was added NEt_3 (1.30 mL, 9.36 mmol, 3.0 eq) and DMAP (191 mg, 1.56 mmol, 0.5 eq). The resulting solution was cooled to 0 °C before dropwise addition of diethylchlorophosphate (1.35 mL, 9.36 mmol, 3.0 eq). The resulting mixture was left to stir at room temperature for 16 hours. The mixture was then quenched with H_2O (50 mL) and extracted with CH_2Cl_2 (3 x 50 mL). The organic extracts were combined, washed with brine (50 mL), dried ($MgSO_4$) and evaporated *in vacuo* to give a yellow oil. The yellow oil was purified by flash chromatography (2% MeOH in CH_2Cl_2) to give *meso*-6-cycloheptene-1,5-bisdiethylphosphate (**3a**) as a yellow oil (1.10 g, 88% yield).

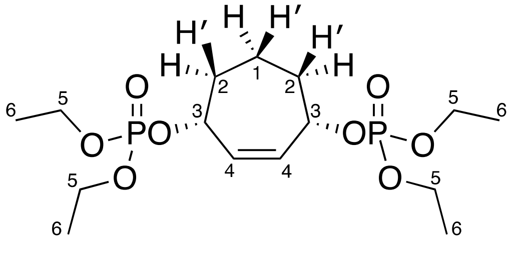

^{1}H NMR (400 MHz, $CDCl_3$) δ_H/ppm: 5.79 (s, 2H, 2CH-(4)), 5.06 – 4.71 (m, 2H, 2CH-(3)), 4.34 – 3.92 (m, 8H, 4CH_2-(5)), 2.12 – 1.94 (m, 3H, 3C***H***H´-(1, 2)), 1.74 – 1.59 (m, 3H, 3CH***H***´-(1, 2)), 1.31 (tq, *J* = 7.1, 1.1 Hz, 12H, 2CH_3-(6)); ^{13}C NMR (101 MHz, $CDCl_3$) δ_C/pm: 133.07 (C(4)), 77.57 (C(3)), 63.86 (C(5)), 34.01 (C(2)), 22.64

(C(1)), 16.24 (C(6)); ^{31}P NMR (162 MHz,$CDCl_3$) δ_P/ppm: -1.68; HRMS (ESI) *m/z* calcd for $C_{15}H_{30}O_8P_2$ $[M+H]^+$: 400.1316, found: 400.1441; IR (ATR) ν (cm^{-1}) thin film, $CHCl_3$: 2985.4 (w), 1646.2 (w), 1396.3 (w), 1263.5 (m), 1164.6 (w), 1027.5 (s), 988.9 (s), 804.1 (w) Consistent with data in the literature.[5]

Trans-Bisphosphate

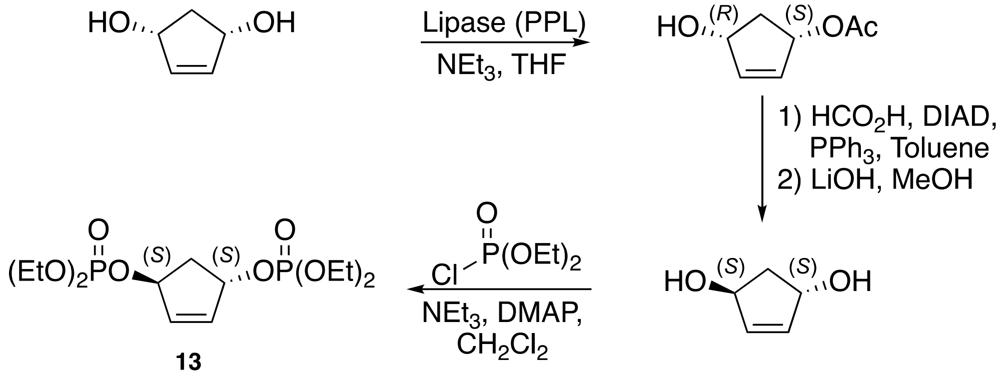

13

Scheme S1. Synthesis of **13**

(1*S*,3*S*)-Cyclopent-4-ene-1,3-diyl tetraethyl bis(phosphate) (13)

(1*S*,4*R*)-4-hydroxycyclopent-2-en-1-yl acetate

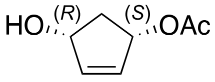

Chemical Formula: $C_7H_{10}O_3$
Molecular Weight: 142.1540

To a solution of *cis*-4-cyclopentene-1,3-diol (200 mg, 2.00 mmol, 1.0 eq) in THF (5.33 mL) was added NEt_3 (0.195 mL, 1.40 mmol, 0.7 eq) and vinyl acetate (1.29 mL, 14.0 mmol, 7.0 eq). PPL lipase (400 mg) was then added, and the resulting beige suspension was left to stir at room temperature for 22 hours. The reaction mixture was filtered through Celite and evaporated *in vacuo* to give a yellow oil. The oil was purified on silica (30-40-50% EtOAc in petroleum ether) to give a white solid. The white solid was recrystallised using a 3:1 mixture of pentane : Et_2O to give (1*S*,4*R*)-4-hydroxycyclopent-2-en-1-yl acetate as colourless needles (126 mg, 44%). **NOTE**: Diacetate by-product (135 mg, 37%) obtained from column and recycled.

GC analysis indicated an enantiomeric excess of >99% [Beta DEX 325 (Supelco) column; initial temperature 80 ˚C, initial hold time 5 min, progress rate 5 ˚C/min, final temperature 160 ˚C, final hold time 10 min; Flow rate 2 mL/min; minor enantiomer, t_R = 14.789 min; major enantiomer, t_R = 15.175 min].

^{1}H NMR (400 MHz, $CDCl_3$) δ_H/ppm: 6.10 (d, J = 5.5 Hz, 1H), 5.97 (d, J = 5.5 Hz, 1H), 5.61 – 5.19 (m, 1H), 4.71 (s, 1H), 2.79 (dt, J = 14.6, 7.3 Hz, 1H), 2.04 (s, 3H), 1.64 (dt, J = 14.6, 3.9 Hz, 1H); ^{13}C NMR (101 MHz, $CDCl_3$) δ_C/ppm: 170.9, 138.6, 132.7, 77.2, 74.9, 40.6, 21.3. Consistent with data found in the literature.[6]

(1*S*,3*S*)-Cyclopent-4-ene-1,3-diyl tetraethyl bis(phosphate) (13)

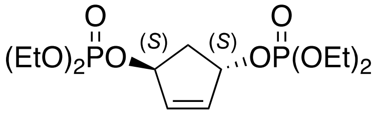

13

(1*S*,4*R*)-4-hydroxycyclopent-2-en-1-yl acetate (529 mg, 3.72 mmol, 1.0 eq) and PPh_3 (1.95 g, 7.44 mmol, 2.0 eq) were dissolved in toluene (10.5 mL) before addition of HCO_2H (288 µL, 7.63 mmol, 2.05 eq). The resulting solution was cooled to 0 °C before dropwise addition of DIAD (1.46 mL, 7.44 mmol, 2.00 eq). The resulting solution was left to stir at 0 °C for 4 hours 40 mins, before being poured over sat. aq. $NaHCO_3$ (50 mL). The aqueous layer was extracted with EtOAc (3 x 50 mL). The organic extracts were combined, washed with brine (30 mL), dried ($MgSO_4$) and evaporated *in vacuo* to give a yellow oil. The oil was filtered through a short plug of silica, washing with CH_2Cl_2. The filtrate was evaporated *in vacuo* to give the crude diester (518 mg) which was used immediately in the next step.

The diester was dissolved in MeOH (21 mL) before addition of H_2O (323 µL, 18.27 mmol, 6.0 eq) and LiOH (437 mg, 18.27 mmol, 6.0 eq). The resulting mixture was left to stir at room temperature for 13 hours. The mixture was evaporated *in vacuo* and the resulting residue was filtered through a short plug of silica, rinsing with 5% MeOH in CH_2Cl_2. The filtrate was evaporated *in vacuo* to give the crude alcohol (290 mg) that was used immediately in the next step.

To a solution of the alcohol in CH_2Cl_2 (13 mL) was added NEt_3 (1.21 mL, 8.69 mmol, 3.0 eq) and DMAP (172 mg, 1.45 mmol, 0.5 eq). The resulting solution was cooled to 0 °C before dropwise addition of diethylchlorophosphate (1.26 mL, 8.69 mmol, 3.0 eq). The resulting mixture was left to stir at room temperature for 16 hours. The mixture was then quenched with H_2O (30 mL) and extracted with CH_2Cl_2 (3 x 30 mL). The organic extracts were combined, washed with brine (20 mL), dried ($MgSO_4$) and evaporated *in vacuo* to give a yellow oil. The yellow oil was purified by flash chromatography (2-3% MeOH in EtOAc) to give (1*S*,3*S*)-cyclopent-4-ene-1,3-diyl tetraethyl bis(phosphate) (**13**) as colourless oil (948 mg, 68% yield over 3 steps). **NOTE**: Procedure used to produce racemic **±13** from racemic 4-hydroxycyclopent-2-en-1-yl acetate.

Enantiomeric excess of **13** determined by use in General Procedure 1a.

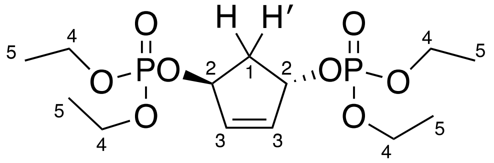

^{1}H NMR (400 MHz, $CDCl_3$) δ_H/ppm: 6.16 (d, *J* = 1.0 Hz, 2H, 2CH-(3)), 5.54 (m, *J* = 5.2 Hz, 2H, 2CH-(2)), 4.08 (m, *J* = 7.9, 7.1, 5.1 Hz, 8H, 4CH_2-(4)), 2.36 (t, *J* = 4.9 Hz, 2H, CH_2-(1)), 1.32 (tdd, *J* = 7.1, 3.0, 1.0 Hz, 12H, 4CH_3-(5)); ^{13}C NMR (101 MHz, $CDCl_3$) δ_c/ppm: 136.1 (C(3)), 81.2 (C(2)), 63.9 (C(4)), 40.0 (C(1)), 16.3 (C(5)); ^{31}P NMR (162 MHz, $CDCl_3$) δ_P/ppm: -1.53; HRMS (ESI) *m/z* calcd for $C_{13}H_{26}O_8P_2Na$ $[M+Na]^+$: 395.09951, found: 395.09958; IR (ATR) ν (cm^{-1}) thin film, $CHCl_3$: 2984.8 (w), 1445.1 (w), 1371.8 (w), 1262.7 (m), 1165.9 (w), 1028.0 (s), 978.1 (s), 804.2 (w), 754.9 (w). $[\alpha]^{25}_{589}$ = -123.2 (c=1.0 in $CHCl_3$, >99% ee)

2-Substituted Bisphosphates

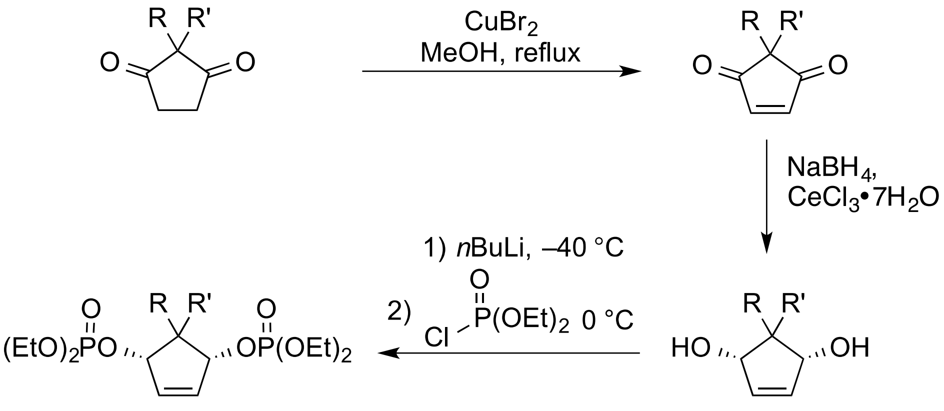

Scheme S2. General synthesis of 2-substituted diphosphates

2,2-Dimethylcyclopentane-1,3-dione

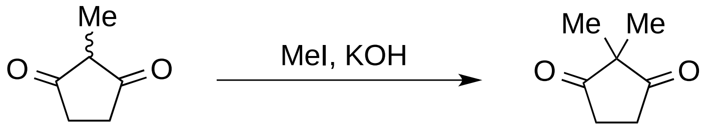

2-Methyl-1,3-cyclopentanedione (2.50 g, 22.23 mmol, 1.0 eq) was dissolved in 1,4-dioxane (18.75 mL) and water (6.5 mL) before addition of KOH (1.31 g, 23.41 mmol, 1.05 eq) and methyl iodide (1.53 mL, 24.53 mmol, 1.1 eq). The resulting mixture was heated to reflux at left to stir for 12 hours. The reaction mixture was poured over water (50 mL) and extracted with EtOAc (3 x 50 mL). The organic extracts were combined, washed with brine (50 mL), dried ($MgSO_4$) and evaporated *in vacuo* to give a yellow oil. The yellow oil was purified by flash chromatography (10-20-30% EtOAc in petroleum ether) to give 2,2-dimethylcyclopentane-1,3-dione (1.765 g, 63%) as a white solid.

^{1}H NMR (400 MHz, $CDCl_3$) δ_H/ppm: 2.76 (s, 4H), 1.10 (s, 6H); ^{13}C NMR (101 MHz, $CDCl_3$) δ_C/ppm: 216.4, 52.7, 34.6, 20.3. Consistent with data in the literature.[7]

2-Allyl-2-methylcyclopentane-1,3-dione

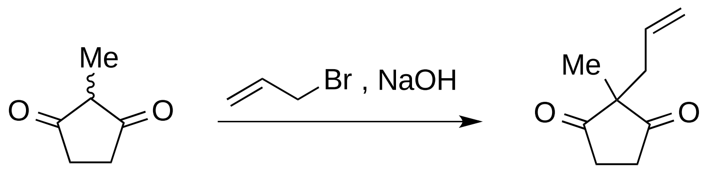

2-Methyl-1,3-cyclopentanedione (2.50 g, 22.30 mmol, 1 eq) was dissolved in 1M aq. NaOH (25.0 mL) before addition of allyl bromide (3.86 mL, 44.60 mmol, 2.0 eq). The resulting mixture was vigorously stirred (1000 rpm) at room temperature for 40 hours. The reaction mixture was extracted with EtOAc (3 x 20 mL). The organic extracts were combined, washed with brine (20 mL), dried ($MgSO_4$) and evaporated *in vacuo* to give a yellow oil. The yellow oil was purified by flash chromatography (5-10% EtOAc in petroleum ether) to give 2-benzyl-2-methylcyclopentane-1,3-dione (2.555 g, 75%) as a yellow oil.

^{1}H NMR (400 MHz, $CDCl_3$) δ_H/ppm: 5.54 (ddt, *J* = 16.1, 10.9, 7.5 Hz, 1H), 5.05 – 5.03 (m, 1H), 5.02 – 4.98 (m, 1H), 2.77 – 2.59 (m, 4H), 2.30 (dt, *J* = 7.5, 1.1 Hz, 2H), 1.07 (s, 3H); ^{13}C NMR (101 MHz, $CDCl_3$) δ_C/ppm: 216.3, 131.6, 119.9, 56.8, 40.1, 35.5, 18.9. Consistent with data in the literature.[8]

2-Benzyl-2-methylcyclopentane-1,3-dione

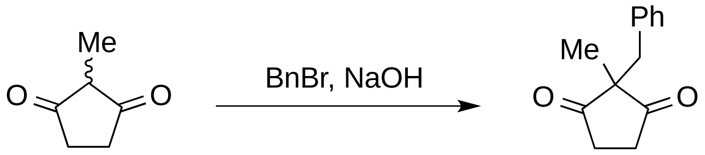

2-Methyl-1,3-cyclopentanedione (1.50 g, 13.4 mmol, 1 eq) was dissolved in 1M aq. NaOH (15.0 mL) before addition of benzyl bromide (3.20 mL, 26.8 mmol, 2.0 eq). The resulting mixture was vigorously stirred (1000 rpm) at room temperature for 44 hours. The reaction mixture was extracted with EtOAc (3 x 20 mL). The organic extracts were combined, washed with brine (20 mL), dried ($MgSO_4$) and evaporated *in vacuo* to give a yellow oil. The yellow oil was purified by flash chromatography (5-10-15% EtOAc in petroleum ether) to give 2-benzyl-2-methylcyclopentane-1,3-dione (2.025 g, 75%) as an off-white solid.

^{1}H NMR (400 MHz, $CDCl_3$) δ_H/ppm: 7.25 – 7.18 (m, 3H), 7.06 – 7.01 (m, 2H), 2.95 (s, 2H), 2.54 (dd, J = 19.2, 7.3 Hz, 2H), 2.05 (dd, J = 18.4, 6.2 Hz, 2H), 1.20 (s, 3H); ^{13}C NMR (101 MHz, $CDCl_3$) δ_c/ppm: 217.6, 135.9, 129.7, 128.7, 127.4, 58.4, 43.2, 35.6, 20.2. Consistent with data in the literature.[8]

2-(2-Chlorobenzyl)-2-methylcyclopentane-1,3-dione

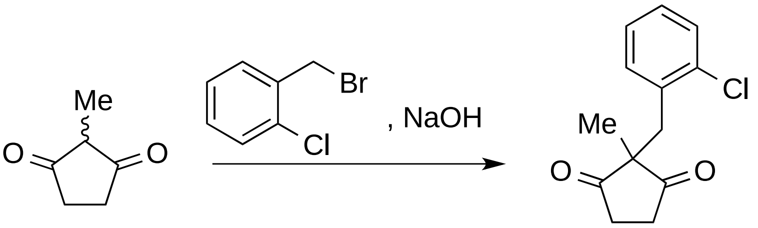

2-Methyl-1,3-cyclopentanedione (2.50 g, 22.30 mmol, 1 eq) was dissolved in 1M aq. NaOH (25.0 mL) before addition of 2-chlorobenzyl bromide (5.80 mL, 44.60 mmol, 2.0 eq). The resulting mixture was vigorously stirred (1000 rpm) at room temperature for 96 hours. The reaction mixture was extracted with EtOAc (3 x 20 mL). The organic extracts were combined, washed with brine (20 mL), dried ($MgSO_4$) and evaporated *in vacuo* to give a yellow oil. The yellow oil was purified by flash chromatography (5-10-20% EtOAc in petroleum ether) to give 2-benzyl-2-methylcyclopentane-1,3-dione (1.453 g, 28%) as a yellow oil.

^{1}H NMR (400 MHz, $CDCl_3$) δ_H/ppm: 7.35 – 7.30 (m, 1H), 7.19 – 7.14 (m, 2H), 7.12 – 7.08 (m, 1H), 3.10 (s, 2H), 2.67 – 2.48 (m, 4H), 1.18 (s, 3H); ^{13}C NMR (101 MHz, $CDCl_3$) δ_c/ppm: 215.8, 134.7, 133.4, 132.0, 130.0, 128.9, 126.9, 57.2, 39.1, 35.7, 18.6. Consistent with data in the literature.[8]

2-((Benzyloxy)methyl)-2-methylcyclopentane-1,3-dione

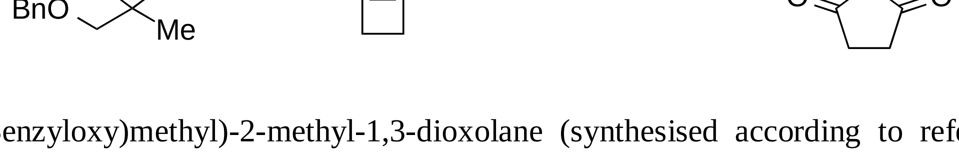

2-((Benzyloxy)methyl)-2-methyl-1,3-dioxolane (synthesised according to reference [10]) (1.00 g, 4.81 mmol, 1.0 eq) and 1,2-bis(trimethylsiloxy)cyclobutene (1.85 mL, 7.21 mmol, 1.50 eq) were dissolved in CH_2Cl_2 (8.0 mL) and cooled to 0 °C before

dropwise addition of boron trifluoride diethyl etherate (0.89 mL, 7.21 mmol, 1.50 eq). The resulting solution was left to stir at room temperature for 16 hours. The reaction mixture was poured over water (50 mL) and extracted with CH_2Cl_2 (3 x 50 mL). The organic extracts were combined, washed with brine (50 mL), dried ($MgSO_4$) and evaporated *in vacuo* to give a yellow oil. The yellow oil was purified by flash chromatography (10-15% EtOAc in petroleum ether) to give 2-((benzyloxy)methyl)-2-methylcyclopentane-1,3-dione (188 mg, 17%) as a colourless oil.

^{1}H NMR (400 MHz, $CDCl_3$) δ_H/ppm: 7.36 – 7.25 (m, 3H), 7.21 – 7.17 (m, 2H), 4.39 (s, 2H), 3.61 (s, 2H), 2.86 – 2.67 (m, 4H), 1.00 (s, 3H); ^{13}C NMR (101 MHz, $CDCl_3$) δ_c/ppm: 216.8, 137.4, 128.5, 127.9, 127.5, 75.4, 73.7, 56.4, 36.4, 15.2. Consistent with data in the literature.[9]

2',3'-Dihydrospiro[cyclopentane-1,1'-indene]-2,5-dione

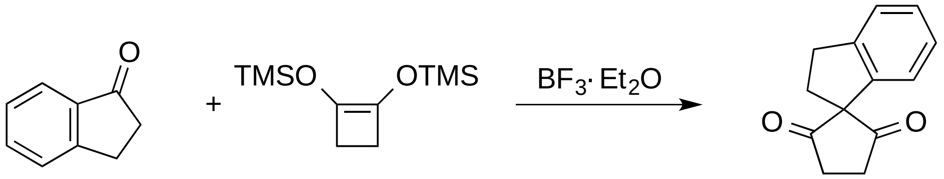

1-Indanone (514 mg, 3.89 mmol, 1.0 eq) and 1,2-bis(trimethylsiloxy)cyclobutene (1.50 mL, 5.84 mmol, 1.50 eq) were dissolved in CH_2Cl_2 (7.0 mL) and cooled to 0 °C before dropwise addition of boron trifluoride diethyl etherate (0.72 mL, 5.84 mmol, 1.50 eq). The resulting solution was left to stir at room temperature for 24 hours. The reaction mixture was poured over water (50 mL) and extracted with CH_2Cl_2 (3 x 50 mL). The organic extracts were combined, washed with brine (50 mL), dried ($MgSO_4$) and evaporated *in vacuo* to give a yellow oil. The yellow oil was purified by flash chromatography (10-20% EtOAc in petroleum ether) to give 2',3'-dihydrospiro[cyclopentane-1,1'-indene]-2,5-dione (454 mg, 58%) as a pale yellow oil.

^{1}H NMR (400 MHz, $CDCl_3$) δ_H/ppm: 7.31 – 7.27 (m, 1H), 7.23 (td, J = 7.5, 1.2 Hz, 1H), 7.15 (t, J = 7.5 Hz, 1H), 6.90 (d, J = 7.6 Hz, 1H), 3.18 (t, J = 7.5 Hz, 2H), 3.11 – 2.83 (m, 4H), 2.40 (t, J = 7.5 Hz, 2H); ^{13}C NMR (101 MHz, $CDCl_3$) δ_c/ppm: 213.3, 145.0, 140.8, 128.5, 127.1, 125.6, 122.6, 70.0, 35.7, 33.0, 31.8. Consistent with data in the literature.[10]

Spiro[chromane-4,1'-cyclopentane]-2',5'-dione

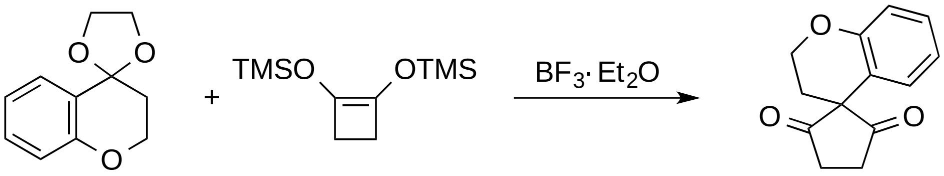

Spiro[chromane-4,2'-[1,3]dioxolane] (961 mg, 5.00 mmol, 1.0 eq) and 1,2-bis(trimethylsiloxy)cyclobutene (1.93 mL, 7.50 mmol, 1.50 eq) were dissolved in CH_2Cl_2 (8.0 mL) and cooled to 0 °C before dropwise addition of boron trifluoride diethyl etherate (0.92 mL, 7.50 mmol, 1.50 eq). The resulting solution was left to stir at room temperature for 18 hours. The reaction mixture was poured over water (50 mL) and extracted with CH_2Cl_2 (3 x 50 mL). The organic extracts were combined, washed with brine (50 mL), dried ($MgSO_4$) and evaporated *in vacuo* to give a yellow oil. The yellow oil was purified by flash chromatography (10-20% EtOAc in petroleum ether) to give spiro[chromane-4,1'-cyclopentane]-2',5'-dione (471 mg, 43%) as a colourless, crystalline solid.

^1H NMR (400 MHz, $CDCl_3$) δ_H/ppm: 7.21 – 7.15 (m, 1H), 6.91 (dd, *J* = 8.3, 1.1 Hz, 1H), 6.85 (td, *J* = 7.7, 1.3 Hz, 1H), 6.57 (dd, *J* = 7.7, 1.6 Hz, 1H), 4.35 – 4.31 (m, 2H), 3.11 – 2.90 (m, 4H), 2.10 – 2.06 (m, 2H); ^{13}C NMR (101 MHz, $CDCl_3$) δ_c/ppm: 213.7, 155.5, 129.5, 128.1, 121.2, 118.2, 117.8, 61.1, 57.2, 35.6, 29.3. Consistent with data in the literature.[10]

2,2-Dimethylcyclopent-4-ene-1,3-dione

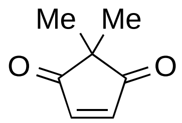

Synthesised according to general procedure 3 using 2,2-Dimethylcyclopentane-1,3-dione on a 13.62 mmol scale. Purified by flash chromatography (10-15-20% EtOAc in petroleum ether) to give 2,2-Dimethylcyclopent-4-ene-1,3-dione as a pale yellow oil (568 mg, 34% yield). **NOTE**: Product volatile.

^1H NMR (400 MHz, $CDCl_3$) δ_H/ppm: 7.17 (s, 2H), 1.12 (s, 6H); ^{13}C NMR (101 MHz, $CDCl_3$) δ_c/ppm: 207.8, 147.2, 46.5, 19.7. Consistent with data in the literature.[11]

2-Allyl-2-methylcyclopent-4-ene-1,3-dione

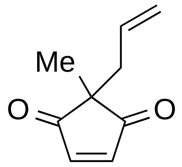

Synthesised according to general procedure 3 using 2-allyl-2-methylcyclopentane-1,3-dione on a 16.30 mmol scale. Purified by flash chromatography (2-5-10% EtOAc in petroleum ether) to give 2-allyl-2-methylcyclopent-4-ene-1,3-dione as a bright yellow oil (761 mg, 31% yield). **NOTE**: Product volatile.

^{1}H NMR (400 MHz, $CDCl_3$) δ_H/ppm: 7.20 (s, 2H), 5.44 (ddt, J = 16.9, 10.1, 7.5 Hz, 1H), 5.01 – 4.92 (m, 2H), 4.97 – 4.95 (m, 1H), 2.34 (d, J = 7.5 Hz, 2H), 1.11 (s, 3H); ^{13}C NMR (101 MHz, $CDCl_3$) δ_C/ppm: 207.2, 148.4, 131.5, 119.7, 50.6, 38.8, 18.6. Consistent with data in the literature.[8]

2-Benzyl-2-methylcyclopent-4-ene-1,3-dione

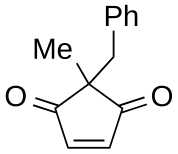

Synthesised according to general procedure 3 using 2-benzyl-2-methylcyclopentane-1,3-dione on a 16.81 mmol scale. Purified by flash chromatography (5-10-15% EtOAc in petroleum ether) to give 2-benzyl-2-methylcyclopent-4-ene-1,3-dione as a bright yellow solid (2.99 g, 89% yield).

^{1}H NMR (400 MHz, $CDCl_3$) δ_H/ppm: 7.18 – 7.12 (m, 3H), 6.98 (s, 2H), 6.94 – 6.91 (m, 2H), 2.99 (s, 2H), 1.25 (s, 3H); ^{13}C NMR (101 MHz, $CDCl_3$) δ_C/ppm: 207.4, 148.9, 135.7, 129.8, 127.2, 52.7, 41.1, 19.5. Consistent with data in the literature.[8]

2-(2-Chlorobenzyl)-2-methylcyclopent-4-ene-1,3-dione

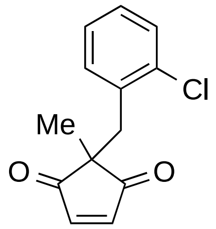

Synthesised according to general procedure 3 using 2-(2-chlorobenzyl)-2-methylcyclopentane-1,3-dione on a 5.93 mmol scale. Purified by flash chromatography (5-10-15% EtOAc in petroleum ether) to give 2-(2-chlorobenzyl)-2-methylcyclopent-4-ene-1,3-dione as a bright yellow solid (1.14 g, 82% yield).

^{1}H NMR (400 MHz, $CDCl_3$) δ_H/ppm: 7.29 – 7.25 (m, 1H), 7.14 – 7.08 (m, 2H), 7.10 (s, 2H), 7.06 – 7.02 (m, 1H), 3.14 (s, 2H), 1.25 (s, 3H); ^{13}C NMR (101 MHz, $CDCl_3$) δ_C/ppm: 206.4, 148.4, 134.5, 133.4, 132.0, 130.1, 128.7, 126.7, 51.5, 37.6, 18.6. Consistent with data in the literature.[8]

2-((Benzyloxy)methyl)-2-methylcyclopent-4-ene-1,3-dione

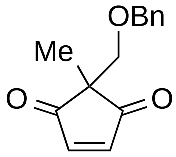

Synthesised according to general procedure 3 using 2-((Benzyloxy)methyl)-2-methylcyclopentane-1,3-dione on a 0.81 mmol scale. Purified by flash chromatography (5-10% EtOAc in petroleum ether) to give 2-((Benzyloxy)methyl)-2-methylcyclopent-4-ene-1,3-dione as a yellow oil (118 mg, 63% yield).

^{1}H NMR (400 MHz, $CDCl_3$) δ_H/ppm: 7.35 – 7.25 (m, 3H), 7.34 (s, 2H), 7.18 – 7.15 (m, 2H), 4.39 (s, 2H), 3.66 (s, 2H), 1.06 (s, 3H); ^{13}C NMR (101 MHz, $CDCl_3$) δ_C/ppm: 206.3, 149.3, 137.5, 128.5, 127.8, 127.4, 73.5, 72.1, 51.4, 14.9. Consistent with data in the literature.[9]

2',3'-Dihydrospiro[cyclopent-4-ene-1,1'-indene]-2,5-dione

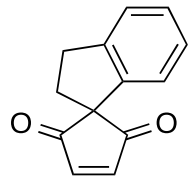

Synthesised according to general procedure 3 using 2',3'-Dihydrospiro[cyclopentane-1,1'-indene]-2,5-dione on a 2.25 mmol scale. Purified by flash chromatography (5-10-15% EtOAc in petroleum ether) to give 2',3'-Dihydrospiro[cyclopent-4-ene-1,1'-indene]-2,5-dione as a bright yellow solid (343 mg, 77% yield).

^{1}H NMR (400 MHz, $CDCl_3$) δ_H/ppm: 7.49 (s, 2H), 7.31 (dp, *J* = 7.5, 1.0 Hz, 1H), 7.24 (td, *J* = 7.5, 1.1 Hz, 1H), 7.12 (tq, *J* = 7.5, 1.0 Hz, 1H), 6.78 (dd, *J* = 7.5, 0.4 Hz, 1H), 3.23 (t, *J* = 7.4 Hz, 2H), 2.41 (t, *J* = 7.4 Hz, 2H); ^{13}C NMR (101 MHz, $CDCl_3$) δ_C/ppm: 204.3, 150.3, 145.5, 140.2, 128.6, 126.9, 125.3, 122.3, 63.3, 60.4, 32.0, 31.8, 14.2. Consistent with data in the literature.[12]

Spiro[chromane-4,1'-cyclopent-4-ene]-2',5'-dione

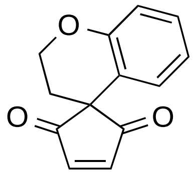

Synthesised according to general procedure 3 using Spiro[chromane-4,1'-cyclopentane]-2',5'-dione on a 2.13 mmol scale. Purified by flash chromatography (10-20-30% EtOAc in petroleum ether) to give Spiro[chromane-4,1'-cyclopent-4-ene]-2',5'-dione as a bright yellow solid (413 mg, 91% yield).

^{1}H NMR (400 MHz, $CDCl_3$) δ_H/ppm: 7.48 (s, 2H), 7.23 – 7.10 (m, 1H), 6.92 (dd, *J* = 8.3, 1.3 Hz, 1H), 6.81 (td, *J* = 7.5, 1.3 Hz, 1H), 6.60 (dd, *J* = 7.8, 1.6 Hz, 1H), 4.43 (t, *J* = 5.3 Hz, 2H), 2.10 (t, *J* = 5.3 Hz, 2H); ^{13}C NMR (101 MHz, $CDCl_3$) δ_C/ppm: 204.5, 155.9, 149.9, 129.6, 127.0, 121.1, 118.2, 117.3, 61.9, 50.3, 28.0. Consistent with data in the literature.[11]

(1*R*,2*S*,3*S*)-2-Benzyl-2-methylcyclopent-4-ene-1,3-diol

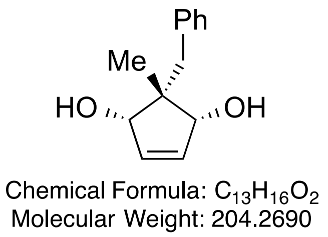

Synthesised according to general procedure 4 using 2-Benzyl-2-methylcyclopent-4-ene-1,3-dione on a 5.00 mmol scale. Purified by flash chromatography (40-50% EtOAc in petroleum ether) to give (1*R*,2*S*,3*S*)-2-Benzyl-2-methylcyclopent-4-ene-1,3-diol as a colourless, crystalline solid (489 mg, 48% yield). Stereochemistry confirmed by 2D NOESY.

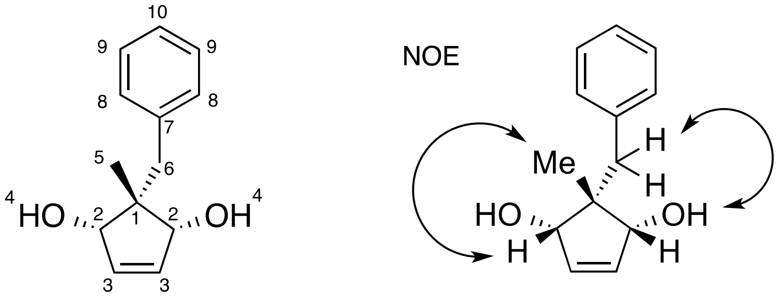

^{1}H NMR (500 MHz, $CDCl_3$) δ_H/ppm: 7.44 – 7.40 (m, 2H, 2ArH-(9)), 7.29 (t, *J* = 7.5 Hz, 2H, 2ArH(8)), 7.21 (tt, *J* = 7.3, 1.3 Hz, 1H, ArH-(10)), 6.19 (dd, *J* = 1.5, 0.9 Hz, 2H, 2CH-(3)), 4.02 (d, *J* = 7.2 Hz, 2H, 2CH-(2)), 2.97 (s, 2H, CH_2-(6)), 2.27 (d, *J* = 7.5 Hz, 2H, 2OH-(4)), 0.72 (s, 3H, CH_3-(5)); ^{13}C NMR (101 MHz, $CDCl_3$) δ_c/ppm: 139.5 (C(7)), 137.2 (C(3)), 130.7 (C(8)), 128.2 (C(9)), 126.0 (C(10)), 82.1 (C(2)), 48.4 (C(1)), 36.7 (C(6)), 24.1 (C(5)); HRMS (ESI) *m/z* calcd for $C_{13}H_{16}O_2Na$ $[M+Na]^+$: 227.10425, found: 227.10421; IR (ATR) ν (cm^{-1}) thin film, $CHCl_3$: 3275.6 (br. m), 2961.1 (w), 2911.1 (w), 1461.0 (w), 1343.4 (w), 1293.5 (w), 1147.5 (w), 1098.8 (w), 1029.2 (m), 981.4 (m), 931.7 (m), 744.2 (m), 700.15 (m), 623.5 (m). Melting point: 99-102 °C.

***Cis*-2,2-dimethylcyclopent-4-ene-1,3-diol**

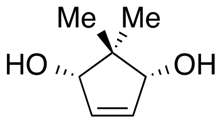

Chemical Formula: $C_7H_{12}O_2$
Molecular Weight: 128.1710

Synthesised according to general procedure 4 using 2,2-dimethylcyclopent-4-ene-1,3-dione on a 3.72 mmol scale. Purified by flash chromatography (1-2% MeOH in EtOAc) to give *cis*-2,2-dimethylcyclopent-4-ene-1,3-diol (single diastereomer) as a colourless, crystalline solid (337 mg, 71% yield).

^{1}H NMR (400 MHz, $CDCl_3$) δ_H/ppm: 6.00 (d, J = 1.1 Hz, 2H), 4.05 (d, J = 6.8 Hz, 2H), 2.14 (d, J = 7.2 Hz, 2H), 1.02 (s, 3H), 1.01 (s, 3H); ^{13}C NMR (101 MHz, $CDCl_3$) δ_C/ppm: 136.1, 82.9, 46.0, 26.9, 16.7. Consistent with data in the literature.[13]

(1*R*,2*S*,3*S*)-2-Allyl-2-methylcyclopent-4-ene-1,3-diol

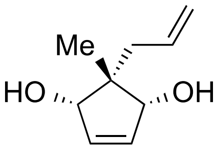

Chemical Formula: $C_9H_{14}O_2$
Molecular Weight: 154.2090

Synthesised according to general procedure 4 using 2-allyl-2-methylcyclopent-4-ene-1,3-dione on a 5.07 mmol scale. Purified by flash chromatography (40-50% EtOAc in petroleum ether) to give (1*R*,2*S*,3*S*)-2-allyl-2-methylcyclopent-4-ene-1,3-diol as a colourless, crystalline solid (334 mg, 43% yield). Stereochemistry confirmed by 2D NOESY.

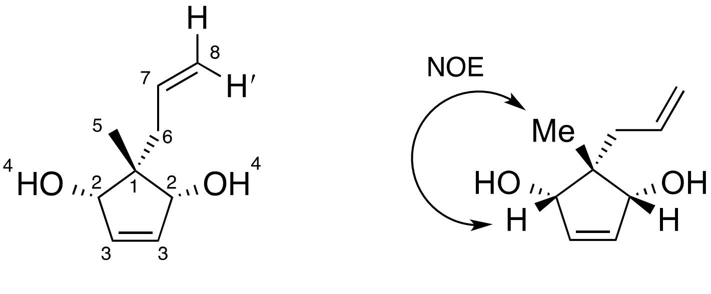

^{1}H NMR (400 MHz, $CDCl_3$) δ_H/ppm: 6.07 (d, *J* = 1.6 Hz, 2H, 2CH-(3)), 6.00 (ddt, *J* = 17.3, 10.1, 7.3 Hz, 1H, CH-(7)), 5.15 (ddt, *J* = 17.3, 2.7, 1.4 Hz, 1H, CH***H*´**-(8)), 5.10 (ddt, *J* = 10.1, 2.2, 1.1 Hz, 1H, C***H***H´-(8)), 4.00 (d, *J* = 0.7 Hz, 2H, 2CH-(2)), 2.74 (s, 2H, 2OH-(4)), 2.33 (d, *J* = 7.3 Hz, 2H, CH_2-(6)), 0.88 (s, 3H, CH_3-(5)); ^{13}C NMR (101 MHz, $CDCl_3$) δ_c/ppm: 136.5 (C(3)), 136.1 (C(7)), 117.4 (C(8)), 82.4 (C(2)), 47.0 (C(1)), 35.9 (C(6)), 24.5 (C(5)); HRMS (ESI) *m/z* calcd for $C_9H_{14}O_2Na$ $[M+Na]^+$: 177.08860, found: 177.08862; IR (ATR) ν (cm^{-1}) thin film, $CHCl_3$: 3309.2 (br. m), 2970.6 (w), 2910.1 (w), 1638.0 (w), 1453.8 (w), 1399.1 (w), 1298.3 (w), 1245.3 (w), 1116.1 (w), 1072.7 (m), 1031.7 (m), 997.9 (m), 987.5 (m), 953.7 (m), 902.5 (m), 828.1 (m), 778.0 (m). Melting point: 70-72 °C.

(1*R*,2*S*,3*S*)-2-(2-Chlorobenzyl)-2-methylcyclopent-4-ene-1,3-diol

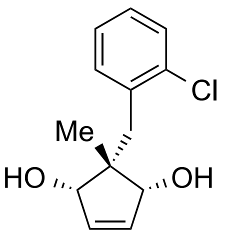

Chemical Formula: $C_{13}H_{15}ClO_2$
Molecular Weight: 238.7110

Synthesised according to general procedure 4 using 2-(2-chlorobenzyl)-2-methylcyclopent-4-ene-1,3-dione on a 2.14 mmol scale. Purified by flash chromatography (40-50% EtOAc in petroleum ether) to give (1*R*,2*S*,3*S*)-2-(2-chlorobenzyl)-2-methylcyclopent-4-ene-1,3-diol as a colourless, crystalline solid (188 mg, 37% yield). Stereochemistry confirmed by 2D NOESY.

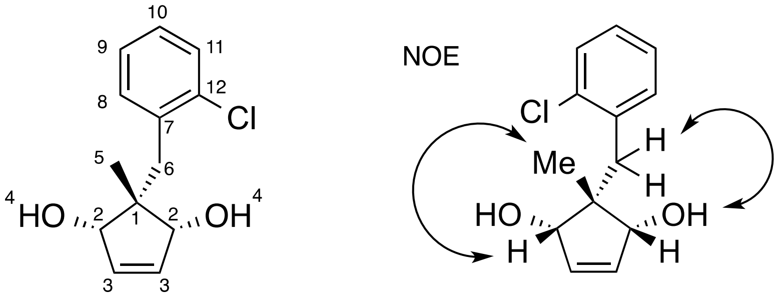

^{1}H NMR (500 MHz, $CDCl_3$) δ_H/ppm: 7.76 (dd, *J* = 7.7, 1.8 Hz, 1H, ArH-(11)), 7.35 (dd, *J* = 7.9, 1.5 Hz, 1H, ArH-(8)), 7.19 (td, *J* = 7.6, 1.5 Hz, 1H, ArH-(10)), 7.13 (td, *J*

= 7.6, 1.8 Hz, 1H, ArH-(9)), 6.20 (dd, *J* = 1.5, 0.9 Hz, 2H, 2CH-(3)), 4.09 – 4.05 (m, 2H, 2CH-(2)), 3.20 (s, 2H, CH_2-(6)), 2.55 – 2.51 (m, 2H, 2OH-(4)), 0.71 (s, 3H, CH_3-(5)); ^{13}C NMR (101 MHz, $CDCl_3$) δ_C/ppm: 137.7 (C(7)), 137.3 (C(3)), 135.2 (C(12)), 132.3 (C(8)), 129.6 (C(11)), 127.4 (C(10)), 126.9 (C(9)), 82.0 (C(2)), 49.1 (C(1)), 31.7 (C(6)), 22.3 (C(5)); HRMS (ESI) *m/z* calcd for $C_{13}H_{15}O_2{}^{35}ClNa$ $[M+Na]^+$: 261.06528, found: 261.06531; IR (ATR) ν (cm^{-1}) thin film, $CHCl_3$: 3315.1 (br. w), 2980.1 (s), 2888.1 (m), 1461.2 (w), 1382.1 (m), 1251.8 (m), 1151.6 (m), 1087.0 (w), 1040.8 (m), 954.6 (m), 820.7 (w), 750.6 (m), 679.0 (w). Melting point: 126-127 °C.

(1*R*,2*S*,3*S*)-2-((Benzyloxy)methyl)-2-methylcyclopent-4-ene-1,3-diol

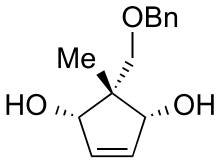

Chemical Formula: $C_{14}H_{18}O_3$
Molecular Weight: 234.2950

Synthesised according to general procedure 4 using 2-((benzyloxy)methyl)-2-methylcyclopent-4-ene-1,3-dione on a 1.27 mmol scale. Purified by flash chromatography (40-50% EtOAc in petroleum ether) to give (1*R*,2*S*,3*S*)-2-((benzyloxy)methyl)-2-methylcyclopent-4-ene-1,3-diol as a colourless oil (137 mg, 46% yield). Stereochemistry confirmed by 2D NOESY.

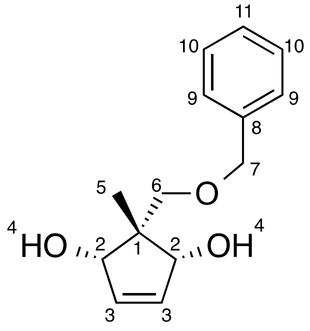

NOE

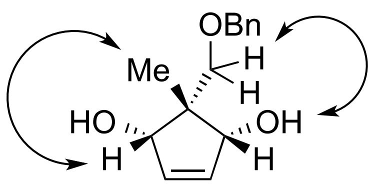

^{1}H NMR (500 MHz, $CDCl_3$) δ_H/ppm: 7.38 – 7.28 (m, 5H, 5ArH-(9-11)), 5.95 (d, *J* = 1.0 Hz, 2H, 2CH-(3)), 4.51 (s, 2H, CH_2-(7)), 4.20 (d, *J* = 8.1 Hz, 2H, 2CH-(2)), 3.72 (s, 2H, CH_2-(6)), 2.79 (d, *J* = 8.8 Hz, 2H, 2OH-(4)), 1.10 (s, 3H, CH_3-(5)); ^{13}C NMR (101 MHz, $CDCl_3$) δ_C/ppm: 137.4 (C(8)), 135.7 (C(3)), 128.7 (C(10)), 128.1 (C(11)), 127.8 (C(9)), 83.7 (C(2)), 73.6 (C(6)), 73.0 (7)), 49.1 (C(1)), 23.3 (C(5)); HRMS

(ESI) *m/z* calcd for $C_{14}H_{18}O_3Na$ $[M+Na]^+$: 257.11482, found: 257.11469; IR (ATR) ν (cm^{-1}) thin film, $CHCl_3$: 3384.8 (br. m), 2980.5 (s), 2885.4 (m), 1454.3 (w), 1382.8 (m), 1251.4 (w), 1144.4 (m), 1073.6 (m), 1030.6 (m), 955.8 (m), 736.7 (w), 698.1 (w).

(1*S*,2*R*,5*S*)-2',3'-Dihydrospiro[cyclopentane-1,1'-inden]-3-ene-2,5-diol

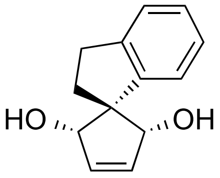

(1*s*,2*R*,5*S*)
Chemical Formula: $C_{13}H_{14}O_2$
Molecular Weight: 202.2530

Synthesised according to general procedure 4 using 2',3'-Dihydrospiro[cyclopentane-1,1'-inden]-3-ene-2,5-dione on a 1.72 mmol scale. Purified by flash chromatography (3% MeOH in CH_2Cl_2) to give (1*S*,2*R*,5*S*)-2',3'-Dihydrospiro[cyclopentane-1,1'-inden]-3-ene-2,5-diol as a colourless, crystalline solid (158 mg, 46% yield, **NOTE**: Mixture of both *cis*-diastereomers and *trans*-diastereomer obtained. Unable to remove *trans* by chromatography; used crude in next step). Stereochemistry confirmed by 2D NOESY.

Analytical data for (1*S*,2*R*,5*S*)-2',3'-Dihydrospiro[cyclopentane-1,1'-inden]-3-ene-2,5-diol

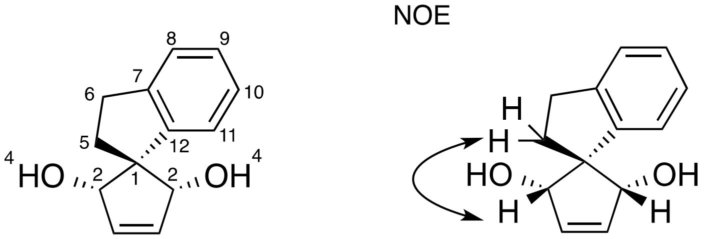

^1H NMR (500 MHz, $CDCl_3$) δ_H/ppm: 7.43 – 7.37 (m, 1H, ArH-(8)), 7.34 – 7.31 (m, 1H, ArH-(11)), 7.31 – 7.25 (m, 1H, ArH-(10)), 7.24 – 7.17 (m, 1H, ArH-(9)), 6.17 (d, *J* = 0.7 Hz, 2H, 2CH-(3)), 4.53 (d, *J* = 8.7 Hz, 2H, 2CH-(2)), 2.99 (t, *J* = 7.4 Hz, 2H, CH_2-(6)), 2.25 (t, *J* = 7.4 Hz, 2H, CH_2-(5)), 1.69 (d, *J* = 9.0 Hz, 2H, 2OH-(4)); ^{13}C

NMR (101 MHz, $CDCl_3$) δ_C/ppm: 147.3 (C(12)), 140.0 (C(7)), 136.8 (C(3)), 128.3 (C(8)), 126.4 (C(10)), 126.2 (C(9)), 125.7 (C(11)), 82.1 (C(2)), 66.3 (C(1)), 36.6 (C(5)), 31.1 (C(6)); HRMS (ESI) *m/z* calcd for $C_{13}H_{14}O_2Na$ $[M+Na]^+$: 225.08860, found: 225.08861; IR (ATR) ν (cm^{-1}) thin film, $CHCl_3$: 3355.8 (br. m), 1479.3 (w), 1293.3 (m), 1031.2 (m), 987.5 (m), 738.4 (m), 632.0 (m). Melting point: 126-130 °C.

(2'*R*,4*S*,5'*S*)-Spiro[chromane-4,1'-cyclopentan]-3'-ene-2',5'-diol

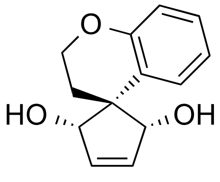

(2'*R*,4*S*,5'*S*)
Chemical Formula: $C_{13}H_{14}O_3$
Molecular Weight: 218.2520

Synthesised according to general procedure 4 using spiro[chromane-4,1'-cyclopent-4-ene]-2',5'-dione on a 1.87 mmol scale. Purified by flash chromatography (2-3% MeOH in CH_2Cl_2) to give (2'*R*,4*S*,5'*S*)-spiro[chromane-4,1'-cyclopentan]-3'-ene-2',5'-diol as a colourless, crystalline solid (121 mg, 30% yield, **NOTE**: Mixture of both *cis*-diastereomers and *trans*-diastereomer obtained. Unable to remove unknown impurity) Stereochemistry confirmed by 2D NOESY.

Analytical data for (2'*R*,4*S*,5'*S*)-Spiro[chromane-4,1'-cyclopentan]-3'-ene-2',5'-diol

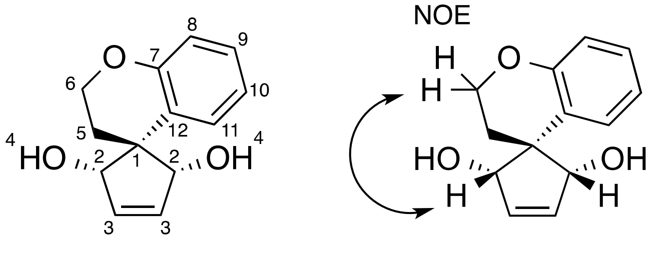

^{1}H NMR (500 MHz, $CDCl_3$) δ_H/ppm: 7.30 (dd, *J* = 7.8, 1.6 Hz, 1H, ArH-(11)), 7.19 (ddd, *J* = 8.3, 7.2, 1.7 Hz, 1H, ArH-(9)), 6.92 (dd, *J* = 8.3, 1.2 Hz, 1H, ArH-(8)), 6.86 (ddd, *J* = 7.8, 7.2, 1.4 Hz, 1H, ArH-(10)), 6.04 (s, 2H, 2CH-(3)), 4.53 (d, *J* = 10.0 Hz, 2H, 2CH-(2)), 4.34 (t, *J* = 5.4 Hz, 2H, CH_2-(6)), 2.26 (t, *J* = 5.4 Hz, 2H, CH_2-(5)), 1.72 (d, *J* = 10.0 Hz, 2H, 2OH-(4)); ^{13}C NMR (101 MHz, $CDCl_3$) δ_C/ppm: 157.3

(C(7)), 135.9 (C(3)), 129.4 (C(9)), 128.4 (C(11)), 120.2 (C(10)), 118.8 (C(8)), 117.9 (C(12)), 83.7 (C(2)), 63.7 (C(6)), 54.6 (C(1)), 34.5 (C(5)); HRMS (ESI) *m/z* calcd for $C_{13}H_{14}O_3Na$ [M+Na]$^+$: 241.08352, found: 241.08354; IR (ATR) ν (cm^{-1}) thin film, $CHCl_3$: 3326.1 (br. m), 1488.0 (w), 1303.5 (w), 1225.4 (m), 1063.9 (m), 1042.7 (m), 1014.3 (w), 973.7 (w), 756.9 (m), 643.9 (w). Melting point: 125-137 °C.

(1*R*,2*S*,3*S*)-2-Benzyl-2-methylcyclopent-4-ene-1,3-bis(diethyl phosphate) (4a)

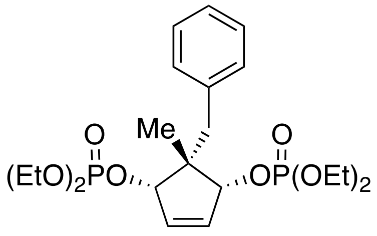

4a

Chemical Formula: $C_{21}H_{34}O_8P_2$
Molecular Weight: 476.4425

Synthesised according to general procedure 5 using (1*R*,2*S*,3*S*)-2-benzyl-2-methylcyclopent-4-ene-1,3-diol on a 12.24 mmol scale. Purified by flash chromatography (1-2-3% MeOH in EtOAc) to give (1*R*,2*S*,3*S*)-2-benzyl-2-methylcyclopent-4-ene-1,3-bis(diethyl phosphate) (**4a**) as a colourless oil (4.614 g, 79%).

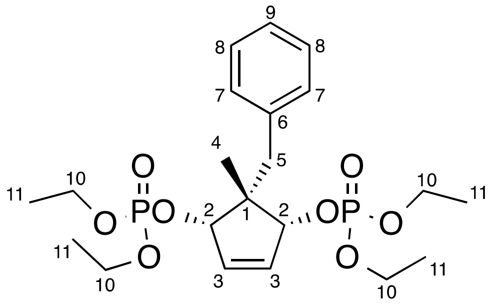

^{1}H NMR (400 MHz, $CDCl_3$) δ_H/ppm: 7.35 – 7.30 (m, 2H, ArH-(7)), 7.27 – 7.22 (m, 2H, ArH-(8)), 7.17 (tt, *J* = 7.2, 1.4 Hz, 1H, ArH-(9)), 6.33 (s, 2H, 2CH-(3)), 4.66 – 4.63 (m, 2H, 2CH-(2)), 4.16 – 4.03 (m, 8H, 4CH$_2$-(10)), 2.98 (s, 2H, CH$_2$-(5)), 1.31 (qd, *J* = 7.1, 1.0 Hz, 12H, 4CH$_3$-(11)), 0.81 (s, 3H, CH$_3$-(4)); ^{13}C NMR (101 MHz, $CDCl_3$) δ_C/ppm: 138.3 (C(6)), 136.0 (C(3)), 130.7 (C(7)), 128.2 (C(8)), 126.2 (C(9)), 86.2 (C(2)), 63.9 (C(10)), 48.6 (C(1)), 36.2 (C(5)), 24.0 (C(4)), 16.3 (C(11)); ^{31}P

NMR (162 MHz, $CDCl_3$) δ_P/ppm: -2.00; HRMS (ESI) *m/z* calcd for $C_{17}H_{35}O_8P_2$ $[M+H]^+$: 477.17291, found: 477.17285; IR (ATR) ν (cm^{-1}) thin film, $CHCl_3$: 2985.8 (w), 1368.0 (w), 1260.7 (m), 1162.4 (w), 1021.5 (s), 987.6 (s), 911.1 (m), 821.3 (w), 756.2 (w).

***Cis*-2,2-dimethylcyclopent-4-ene-1,3-bis(diethyl phosphate) (5a)**

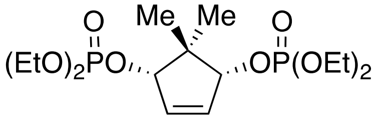

5a

Chemical Formula: $C_{15}H_{30}O_8P_2$
Molecular Weight: 400.3445

Synthesised according to general procedure 5 using *cis*-2,2-dimethylcyclopent-4-ene-1,3-diol on a 1.66 mmol scale. Purified by flash chromatography (2-3-4% MeOH in CH_2Cl_2) to give *cis*-2,2-dimethylcyclopent-4-ene-1,3-bis(diethyl phosphate) (**5a**) as a colourless oil (458 mg, 69%).

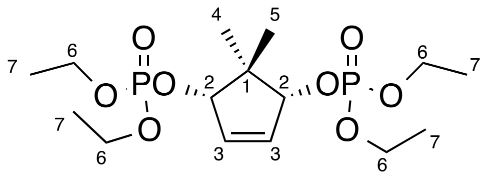

^{1}H NMR (400 MHz, $CDCl_3$) δ_H/ppm: 6.01 (s, 2H, 2CH-(3)), 4.71 (d, *J* = 6.4 Hz, 2H, 2CH-(2)), 4.06 (ddq, *J* = 10.6, 8.0, 7.1 Hz, 8H, $4CH_2$-(6)), 1.29 (tdd, *J* = 7.1, 4.4, 1.0 Hz, 12H, $4CH_3$-(7)), 1.17 (s, 3H, CH_3-(4)), 1.00 (s, 3H, CH_3-(5)); ^{13}C NMR (101 MHz, $CDCl_3$) δ_c/ppm: 133.9 (C(3)), 86.6 (C(2)), 63.8 (C(6)), 47.5 (C1)), 25.9 (C(4)), 17.3 (C5)), 16.2 (C(7)); ^{31}P NMR (162 MHz, $CDCl_3$) δ_P/ppm: -1.65; HRMS (ESI) *m/z* calcd for $C_{15}H_{31}O_8P_2$ $[M+H]^+$: 401.14887, found: 401.14848; IR (ATR) ν (cm^{-1}) thin film, $CHCl_3$: 2984.7 (w), 1368.2 (w), 1262.4 (m), 1165.7 (w), 1023.2 (s), 997.1 (s), 951.4 (s), 912.1 (m), 818.4 (w), 755.3 (w).

(1*R*,2*S*,3*S*)-2-Allyl-2-methylcyclopent-4-ene-1,3-bis(diethyl phosphate) (6a)

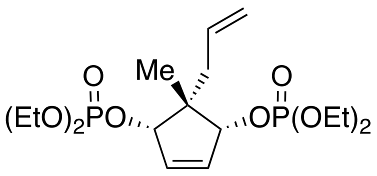

6a

Chemical Formula: $C_{17}H_{32}O_8P_2$
Molecular Weight: 426.3825

Synthesised according to general procedure 5 using (1*R*,2*S*,3*S*)-2-allyl-2-methylcyclopent-4-ene-1,3-diol on a 1.82 mmol scale. Purified by flash chromatography (2-3% MeOH in EtOAc) to give (1*R*,2*S*,3*S*)-2-allyl-2-methylcyclopent-4-ene-1,3-bis(diethyl phosphate) (**6a**) as a colourless oil (632 mg, 81%).

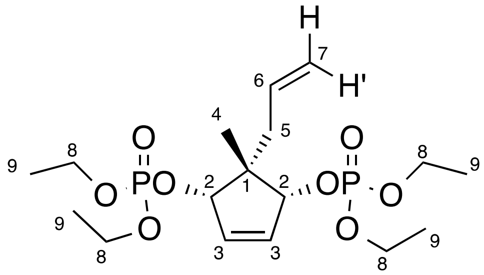

^{1}H NMR (400 MHz, $CDCl_3$) δ_H/ppm: 6.22 (d, *J* = 0.8 Hz, 2H, 2CH-(3)), 5.89 (ddt, *J* = 17.3, 10.2, 7.2 Hz, 1H, CH-(6)), 5.13 – 5.08 (m, 1H, CH***H′***-(7)), 5.08 – 5.05 (m, 1H, C***H***H′-(7)), 4.68 (d, *J* = 5.6 Hz, 2H, 2CH-(2)), 4.15 – 4.01 (m, 8H, $4CH_2$-(8)), 2.37 (d, *J* = 7.2 Hz, 2H, CH_2-(5)), 1.31 (qd, *J* = 7.0, 1.0 Hz, 12H, $4CH_3$-(9)), 1.00 (s, 3H, CH_3-(4)); ^{13}C NMR (101 MHz, $CDCl_3$) δ_c/ppm: 135.3 (C(3)), 134.8 (C(6)), 117.9 (C(7)), 86.2 (C(2)), 63.9 (C(8)), 47.6 (C(1)), 35.7 (C(5)), 24.3 (C(4)), 16.2 (C(9)); ^{31}P NMR (162 MHz, $CDCl_3$) δ_P/ppm: -1.85; HRMS (ESI) *m/z* calcd for $C_{17}H_{32}O_8NaP_2$ $[M+Na]^+$: 449.146.46, found: 449.14596; IR (ATR) ν (cm^{-1}) thin film, $CHCl_3$: 2986.5 (w), 1382.2 (w), 1251.9 (m), 1156.7 (w), 1023.2 (s), 996.4 (s), 949.6 (s), 913.2 (m), 816.9 (w), 754.4 (w).

(1*R*,2*S*,3*S*)-2-(2-Chlorobenzyl)-2-methylcyclopent-4-ene-1,3-bis(diethyl phosphate) (7a)

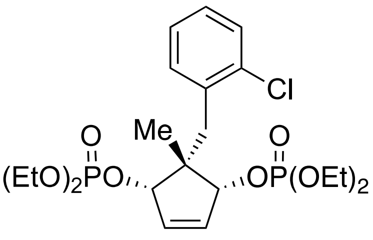

7a

Chemical Formula: $C_{21}H_{33}ClO_8P_2$
Molecular Weight: 510.8845

Synthesised according to general procedure 5 using (1*R*,2*S*,3*S*)-2-(2-chlorobenzyl)-2-methylcyclopent-4-ene-1,3-diol on a 0.79 mmol scale. Purified by flash chromatography (2-3% MeOH in EtOAc) to give (1*R*,2*S*,3*S*)-2-(2-chlorobenzyl)-2-methylcyclopent-4-ene-1,3-bis(diethyl phosphate) (**7a**) as a colourless oil (324 mg, 80%).

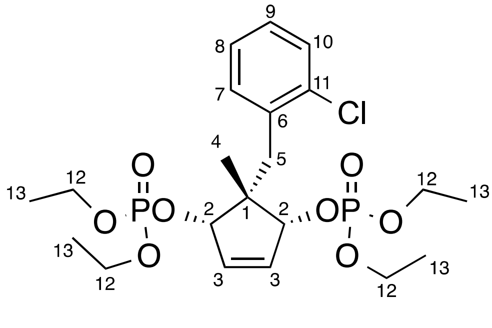

^{1}H NMR (400 MHz, $CDCl_3$) δ_H/ppm: 7.58 (dd, *J* = 7.6, 1.8 Hz, 1H, ArH-(10)), 7.34 (dd, *J* = 7.8, 1.5 Hz, 1H, ArH-(7)), 7.18 (td, *J* = 7.6, 1.5 Hz, 1H, ArH-(9)), 7.12 (td, *J* = 7.8, 1.8 Hz, 1H, ArH-(8)), 6.36 (s, 2H, 2CH-(3)), 4.72 – 4.69 (m, 2H, 2CH-(2)), 4.18 – 4.04 (m, 8H, $4CH_2$-(12)), 3.23 (s, 2H, CH_2-(5)), 1.33 (qd, *J* = 7.1, 1.0 Hz, 12H, $4CH_3$-(13)), 0.82 (s, 3H, CH_3-(4)); ^{13}C NMR (101 MHz, $CDCl_3$) δ_c/ppm: 136.6 (C(11)), 136.0 (C(3)), 135.4 (C(6)), 131.4 (C(7)), 129.7 (C(10)), 127.6 (C(9)), 127.0 (C(8)), 86.1 (C(2)), 63.9 (C(12)), 48.9 (C(1)), 31.2 (C(5)), 22.3 (C(4)), 16.3 (C(13)); ^{31}P NMR (162 MHz, $CDCl_3$) δ_P/ppm: -2.06; HRMS (ESI) *m/z* calcd for $C_{21}H_{34}O_8{}^{35}ClP_2$ $[M+H]^+$: 511.14120, found: 511.14102; IR (ATR) ν (cm^{-1}) thin film, $CHCl_3$: 2986.9 (w), 1369.9 (w), 1262.2 (m), 1163.4 (w), 1023.0 (s), 995.4 (s), 952.1 (s), 909.9 (m), 816.7 (w), 756.5 (w).

(1*R*,2*S*,3*S*)-2-((Benzyloxy)methyl)-2-methylcyclopent-4-ene-1,3-bis(diethyl phosphate) (8a)

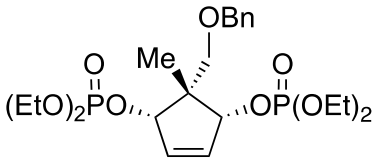

8a

Chemical Formula: $C_{22}H_{36}O_9P_2$
Molecular Weight: 506.4685

Synthesised according to general procedure 5 using (1*R*,2*S*,3*S*)-2-((benzyloxy)methyl)-2-methylcyclopent-4-ene-1,3-diol on a 0.59 mmol scale. Purified by flash chromatography (2-3-4% MeOH in EtOAc) to give (1*R*,2*S*,3*S*)-2-((benzyloxy)methyl)-2-methylcyclopent-4-ene-1,3-bis(diethyl phosphate) (**8a**) as a colourless oil (242 mg, 81%).

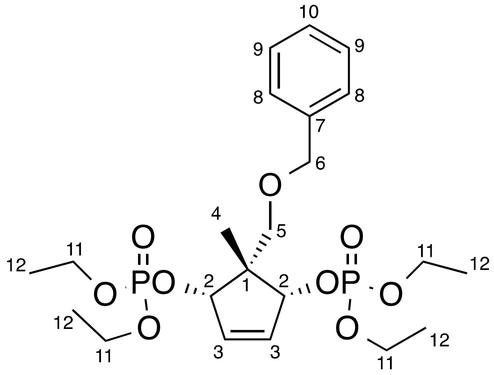

^{1}H NMR (400 MHz, $CDCl_3$) δ_H/ppm: 7.33 – 7.25 (m, 4H, 4ArH-(8, 9)), 7.25 – 7.19 (m, 1H, ArH-(10)), 6.24 – 6.20 (m, 2H, 2CH-(3)), 4.76 – 4.73 (m, 2H, 2CH-(2)), 4.51 (s, 2H, CH_2-(6)), 4.06 – 3.95 (m, 8H, 4CH_2-(11)), 3.62 (s, 2H, CH_2-(5)), 1.23 (qd, *J* = 7.1, 1.0 Hz, 12H, 4CH_3-(12)), 1.15 (s, 3H, CH_3-(4)); ^{13}C NMR (101 MHz, $CDCl_3$) δ_c/ppm: 138.7 (C(7)), 135.3 (C(3)), 128.2 (C(9)), 127.4 (C(10)), 127.3 (C(8)), 85.8 (C(2)), 73.4 (C(5)), 69.7 (C(6)), 63.7 (C(11)), 48.4 (C(1)), 22.6 (C(4)), 16.1 (C(12)); ^{31}P NMR (162 MHz, $CDCl_3$) δ_P/ppm: -1.83; HRMS (ESI) *m/z* calcd for $C_{27}H_{37}O_9P_2$ $[M+H]^+$: 507.19073, found: 507.19021; IR (ATR) ν (cm^{-1}) thin film, $CHCl_3$: 2980.8 (w), 1392.9 (w), 1262.5 (m), 1164.6 (w), 1098.7 (w), 1072.2 (w), 1021.0 (s), 965.3 (s), 919.7 (w), 879.9 (w), 742.3 (w), 700.0 (w).

(1*S*,2*R*,5*S*)-2',3'-Dihydrospiro[cyclopentane-1,1'-inden]-3-ene-2,5-bis(diethyl phosphate) (9a)

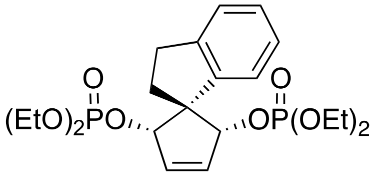

9a

Chemical Formula: $C_{21}H_{32}O_8P_2$
Molecular Weight: 474.42652

Synthesised according to general procedure 5 using (1*S*,2*R*,5*S*)-2',3'-dihydrospiro[cyclopentane-1,1'-inden]-3-ene-2,5-diol on a 0.72 mmol scale. Purified by flash chromatography (1-2-3% MeOH in EtOAc) to give (1*S*,2*R*,5*S*)-2',3'-dihydrospiro[cyclopentane-1,1'-inden]-3-ene-2,5-bis(diethyl phosphate) (**9a**) as a colourless oil (221 mg, 65%).

^{1}H NMR (400 MHz, $CDCl_3$) δ_H/ppm: 7.32 (d, *J* = 7.6 Hz, 1H, ArH-(7)), 7.18 – 7.14 (m, 1H, ArH-(10)), 7.12 (td, *J* = 7.1, 1.3 Hz, 1H, ArH-(9)), 7.05 (td, *J* = 7.6, 7.1, 0.8 Hz, 1H, ArH-(8)), 6.26 – 6.25 (m, 2H, 2CH-(3)), 5.11 – 5.08 (m, 2H, 2CH-(2)), 3.90 – 3.79 (m, 4H, 2CH$_2$-(12)), 3.63 – 3.54 (m, 4H, 2CH$_2$-(12)), 2.97 (t, *J* = 7.3 Hz, 2H,

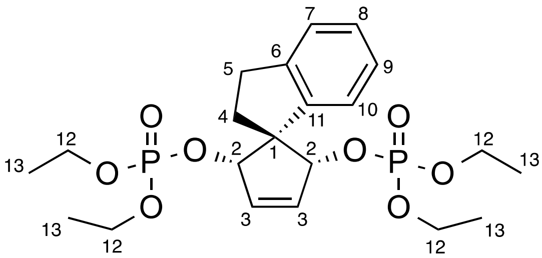

CH$_2$-(5)), 2.31 (t, *J* = 7.3 Hz, 2H, CH$_2$-(4)), 1.20 – 1.15 (m, 6H, 2CH$_3$-(13)), 1.06 – 1.01 (m, 6H, 2CH$_3$-(13)); ^{13}C NMR (101 MHz, $CDCl_3$) δ_C/ppm: 145.4 (C(11)), 139.9 (C(6)), 135.2 (C(3)), 128.4 (C(7)), 127.4 (C(9)), 125.2 (C(8)), 124.1 (C(10)), 85.6 (C(2)), 63.9 (C(1)), 63.6 (C(12)), 37.5 (C(4)), 30.7 (C(5)), 16.0 (C(13)); ^{31}P NMR (162 MHz, $CDCl_3$) δ_P/ppm: -2.29; HRMS (ESI) *m/z* calcd for $C_{21}H_{33}O_8P_2$ $[M+H]^+$: 475.16452, found: 475.16455; IR (ATR) ν (cm^{-1}) thin film, $CHCl_3$: 2981.6 (w), 1262.6 (m), 1165.4 (w), 1009.2 (s), 953.5 (s), 902.6 (w), 883.4 (w), 818.6 (w), 803.3 (w), 760.3 (w).

(2'*R*,4*S*,5'*S*)-Spiro[chromane-4,1'-cyclopentan]-3'-ene-2',5'-bis(diethyl phosphate) (10a)

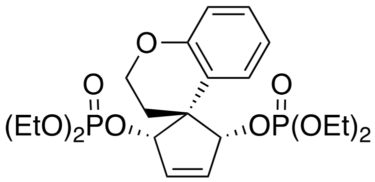

10a

Chemical Formula: $C_{21}H_{32}O_9P_2$
Molecular Weight: 490.4255

Synthesised according to general procedure 5 using (2'*R*,4*S*,5'*S*)-spiro[chromane-4,1'-cyclopentan]-3'-ene-2',5'-diol on a 0.57 mmol scale. Purified by flash chromatography (3-4-5% MeOH in EtOAc) to give (2'*R*,4*S*,5'*S*)-spiro[chromane-4,1'-cyclopentan]-3'-ene-2',5'-bis(diethyl phosphate) (**10a**) as a colourless oil (193 mg, 69%).

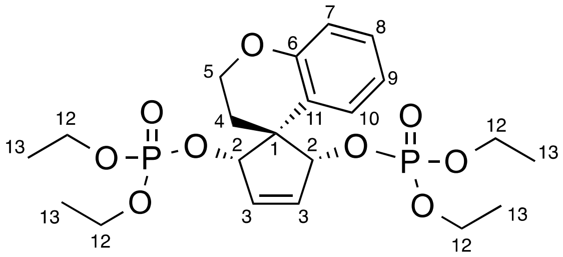

^{1}H NMR (400 MHz, CDCl$_3$) δ_H/ppm: 7.17 (dd, *J* = 7.9, 1.5 Hz, 1H, ArH-(10)), 7.03 (td, *J* = 7.1, 1.7 Hz, 1H, ArH-(8)), 6.76 – 6.69 (m, 2H, 2ArH-(7, 9)), 6.19 (s, 2H, 2CH-(3)), 5.10 (dd, *J* = 7.2, 1.0 Hz, 2H, 2CH-(2)), 4.30 (t, *J* = 5.4 Hz, 2H, CH$_2$-(5)), 3.91 – 3.81 (m, 4H, 2CH$_2$-(12)), 3.63 – 3.53 (m, 4H, 2CH$_2$-(12)), 2.32 (t, *J* = 5.4 Hz, 2H, CH$_2$-(4)), 1.19 (td, *J* = 7.1, 1.0 Hz, 6H, 2CH$_3$-(13)), 1.03 (td, *J* = 7.1, 1.1 Hz, 6H, 2CH$_3$-(13)); ^{13}C NMR (101 MHz, CDCl$_3$) δ_c/ppm: 155.7 (C(6)), 134.3 (C(3)), 132.1 (C(10)), 128.2 (C(8)), 118.7 (C(9)), 118.0 (C(11)), 116.9 (C(7)), 86.7 (C(2)), 63.8 (C(12)), 63.1 (C(5)), 52.3 (C(1)), 34.8 (C(4)), 16.0 (C(13)); ^{31}P NMR (162 MHz, CDCl$_3$) δ_P/ppm: -2.29; HRMS (ESI) *m/z* calcd for $C_{21}H_{33}O_9P_2$ [M+H]$^+$: 491.15943, found: 491.15935; IR (ATR) ν (cm^{-1}) thin film, CHCl$_3$: 2981.5 (w), 1490.2 (w), 1450.9 (w), 1308.6 (m), 1261.2 (w), 1009.0 (s), 957.5 (s), 889.0 (w), 818.3 (w), 803.8 (w), 754.5 (m).

Heterocyclic Bisphosphates

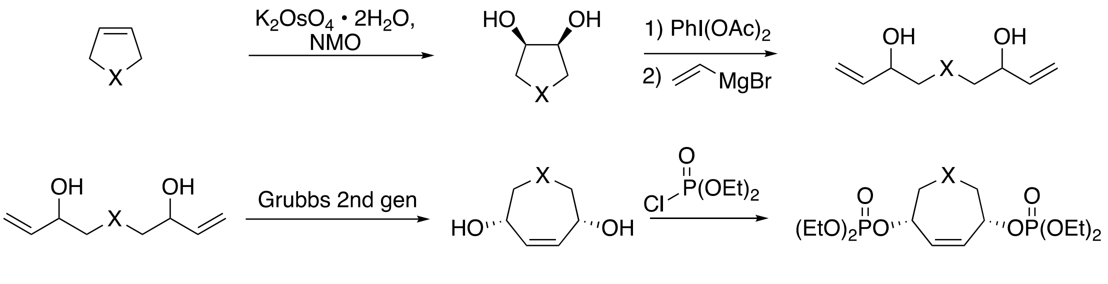

Scheme SII. General synthesis of heterocyclic bisphosphates

1,1'-Oxybis(but-3-en-2-ol)

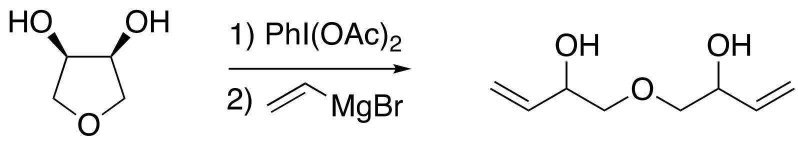

1,4-Anhydroerythritol (1.37 mL, 16.7 mmol, 1 eq) was dissolved in CH_2Cl_2 (35 mL) and cooled to 0 ˚C before addition of $PhI(OAc)_2$ (8.05 g, 25.0 mmol, 1.5 eq). the resulting mixture was allowed to warm to room temperature and stir for 2 hours. The mixture was cooled to –78 ˚C and vinylmagnesium bromide (1.0 M in THF, 100 mL, 100 mmol, 6 eq) was added slowly via canula. The resulting mixture was warmed to 0 ˚C and left to stir for 1 hour and then poured over sat. aq. NH_4Cl (150 mL). The mixture was extracted with EtOAc (3 x 100 mL). The organic extracts were combined, washed with brine (150 mL), dried ($MgSO_4$) and evaporated *in vacuo* to give a yellow oil. The yellow oil was purified by flash chromatography (40-50% EtOAc in petroleum ether) to give 1,1'-oxybis(but-3-en-2-ol) (1.644 g, 62%) as a colourless oil.

^{1}H NMR (500 MHz, $CDCl_3$) δ_H/ppm: 5.81 (dddd, *J* = 17.2, 10.6, 5.7, 3.4 Hz, 2H), 5.34 (d, *J* = 17.2 Hz, 2H), 5.17 (dd, *J* = 10.6, 1.4 Hz, 2H), 4.36 – 4.31 (m, 2H), 3.57 (dd, *J* = 10.0, 3.1 Hz, 1H), 3.53 (dd, *J* = 10.1, 3.1 Hz, 1H), 3.45 (s, 2H), 3.42 (dd, *J* = 10.1, 7.9 Hz, 1H), 3.37 (dd, *J* = 10.0, 8.2 Hz, 1H); ^{13}C NMR (126 MHz, $CDCl_3$) δ_C/ppm: 136.6, 116.6, 75.4, 71.6. Consistent with data in the literature.[15]

Cis-2,3,6,7-tetrahydrooxepine-3,6-diol

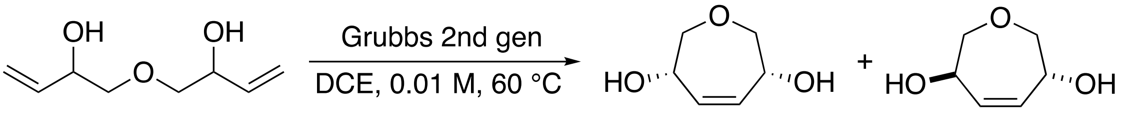

To a solution of 1,1'-oxybis(but-3-en-2-ol) (1.36 g, 8.60 mmol, 1.0 eq) in 1,2-dichloroethane (860 ml) was added Grubbs Catalyst 2nd Generation (146 mg, 0.172 mmol, 2 mol%). The resulting solution was left to stir at 60 ˚C for 24 hours. Another portion of Grubbs Catalyst 2nd Generation (146 mg, 0.172 mmol, 2 mol%) was added and the reaction was left to stir at 60 ˚C for a further 24 hours. The reaction mixture was concentrated *in vacuo* to give a brown oil. The brown oil was purified by flash chromatography (75% EtOAc in petroleum ether) to give *cis*-2,3,6,7-tetrahydrooxepine-3,6-diol (less polar, eluted first, 204 mg, 18%) and *trans*-2,3,6,7-tetrahydrooxepine-3,6-diol (more polar, eluted second, 203 mg, 18%) as brown oils. *Cis*-diol characterised.

^{1}H NMR (400 MHz, $CDCl_3$) δ_H/ppm: 5.91 (dd, *J* = 3.1, 1.5 Hz, 2H), 4.14 (s, 2H), 3.88 (dd, *J* = 12.4, 5.1 Hz, 4H), 3.63 (dd, *J* = 12.4, 2.3 Hz, 2H); ^{13}C NMR (126 MHz, $CDCl_3$) δ_C/ppm: 133.5, 76.2, 69.9. Consistent with data in the literature.[16]

Tetraethyl ((3*R*,6*S*)-2,3,6,7-tetrahydrooxepine-3,6-diyl) bis(phosphate) (11a)

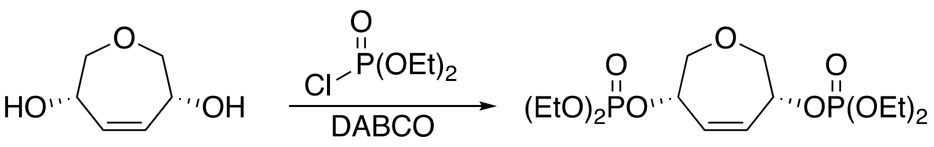

11a

Cis-2,3,6,7-tetrahydrooxepine-3,6-diol (204 mg, 1.57 mmol, 1.0 eq) was dissolved in THF (10 mL) before addition of DABCO (527 mg, 4.70 mmol, 3.0 eq). The resulting solution was cooled to 0 ˚C before dropwise addition of diethyl chlorophosphate (0.68 mL, 4.70 mmol, 3.0 eq). The resulting mixture was allowed to warm to room temperature and stir for 17 hours before being poured over sat. aq. $NaHCO_3$ (50 mL). The mixture was extracted with EtOAc (3 x 50 mL). The organic extracts were combined, washed with brine (100 mL), dried ($MgSO_4$) and evaporated *in vacuo* to

give a yellow oil. The yellow oil was purified by flash chromatography (0-1-2% MeOH in CH_2Cl_2) to give tetraethyl ((3*R*,6*S*)-2,3,6,7-tetrahydrooxepine-3,6-diyl) bis(phosphate) (**11a**) (289 mg, 46%) as a colourless oil.

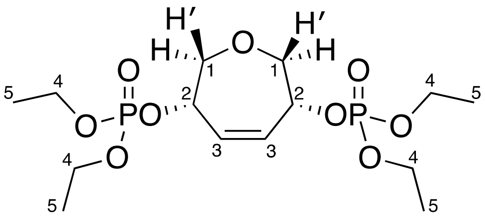

^{1}H NMR (400 MHz, $CDCl_3$) δ_H/ppm: 5.86 (s, 2H, 2CH-(3)), 5.03 – 4.95 (m, 2H, 2CH-(2)), 4.16 – 4.03 (m, 8H, 4CH_2-(4)), 4.03 (dd, *J* = 11.7, 4.2 Hz, 2H, 2CH***H*′**-(1)), 3.51 (dd, *J* = 11.7, 9.2 Hz, 2H, 2C***H***H′-(1)), 1.32 (tt, *J* = 7.1, 1.3 Hz, 6H); ^{13}C NMR (126 MHz, $CDCl_3$) δ_c/ppm: 138.9 (C(3)), 75.2 (C(2)), 74.0 (C(1)), 64.2 (C(4)), 16.2 (C(5)); ^{31}P NMR (162 MHz, $CDCl_3$) δ_P/ppm: –1.48; HRMS (ESI) *m/z* calcd for $C_{14}H_{28}O_9NaP$ $[M+Na]^+$: 425.11008, found: 425.10994; IR (ATR) ν (cm^{-1}) thin film, $CHCl_3$: 2889.3 (w), 1461.4 (w), 1389.2 (w), 1263.0 (m), 1029.9 (s), 988.5 (s), 808.4 (w), 701.6 (w).

***tert*-Butyl (3*R*,4*S*)-3,4-dihydroxypyrrolidine-1-carboxylate**

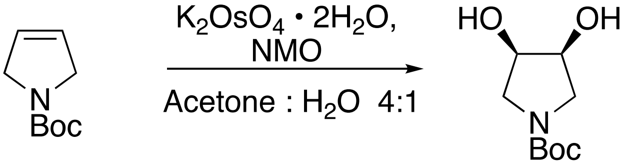

To a solution of N-Boc-2,5-dihydro-1*H*-pyrrole (2.50 g, 14.77 mmol, 1 eq) in acetone (24 mL) and water (6 mL) was added NMO (2.60 g, 22.19 mmol, 1.5 eq) followed by potassium osmate(VI) dihydrate (163 mg, 0.44 mmol, 0.03 eq). The resulting orange/brown suspension was left to stir at room temperature overnight. The resulting mixture was quenched by addition of sat. aq. $Na_2S_2O_3$ (100 mL). The mixture was extracted with EtOAc (3 x 100 mL). The organic extracts were combined, washed with brine (150 mL), dried ($MgSO_4$) and evaporated *in vacuo* to give a yellow oil. The yellow oil was purified by flash chromatography (0-10% MeOH in CH_2Cl_2) to give *tert*-butyl (3*R*,4*S*)-3,4-dihydroxypyrrolidine-1-carboxylate (2.98g, 99%) as a clear amorphous solid.

^{1}H NMR (500 MHz, $CDCl_3$) δ_H/ppm: 4.20 – 4.15 (m, 2H), 3.77 (s, 2H), 3.50 (dd, J = 11.0, 5.2 Hz, 2H), 3.29 (dd, J = 11.0, 4.0 Hz, 2H), 1.41 (s, 9H); ^{13}C NMR (126 MHz, $CDCl_3$) δ_C/ppm: 155.0, 80.1, 71.0, 50.5, 28.6. Consistent with data in the literature.[15]

***tert*-Butyl bis(2-hydroxybut-3-en-1-yl)carbamate**

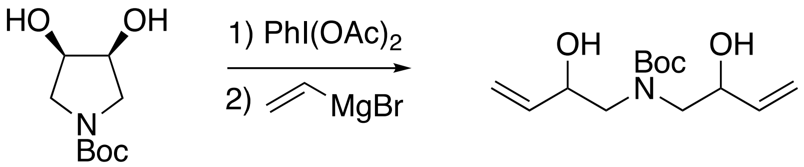

tert-Butyl (3*R*,4*S*)-3,4-dihydroxypyrrolidine-1-carboxylate (3.57 g, 16.7 mmol, 1 eq) was dissolved in CH_2Cl_2 (35 mL) and cooled to 0 ˚C before addition of $PhI(OAc)_2$ (8.05 g, 25.0 mmol, 1.5 eq). the resulting mixture was allowed to warm to room temperature and stir for 2 hours. The mixture was cooled to –78 ˚C and vinylmagnesium bromide (1.0 M in THF, 100 mL, 100 mmol, 6 eq) was added slowly via canula. The resulting mixture was warmed to 0 ˚C and left to stir for 1 hour and then poured over sat. aq. NH_4Cl (150 mL). The mixture was extracted with EtOAc (3 x 100 mL). The organic extracts were combined, washed with brine (150 mL), dried ($MgSO_4$) and evaporated *in vacuo* to give a yellow oil. The yellow oil was purified by flash chromatography (40-50% EtOAc in petroleum ether) to give *tert*-butyl bis(2-hydroxybut-3-en-1-yl)carbamate (3.59 g, 84%) as a colourless oil. NMR shows a mixture of rotamers.

^{1}H NMR (500 MHz, $CDCl_3$) δ_H/ppm: 5.88 – 5.72 (m, 2H), 5.38 – 5.24 (m, 2H), 5.14 (d, J = 10.6 Hz, 2H), 4.58 – 4.28 (m, 2H), 4.00 (s, 2H), 3.74 – 2.77 (m, 4H), 1.45 (s, 9H); ^{13}C NMR (126 MHz, $CDCl_3$) δ_C/ppm: 157.1, 156.4, 138.3, 138.1, 115.9, 80.6, 72.8, 72.2, 72.1, 57.4, 57.1, 55.7, 55.4, 28.5, 28.5. Consistent with data in the literature.[16]

tert-Butyl (3_R_,6_S_)-3,6-dihydroxy-2,3,6,7-tetrahydro-1_H_-azepine-1-carboxylate

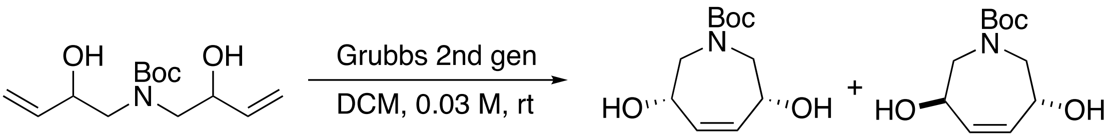

To a solution of *tert*-butyl bis(2-hydroxybut-3-en-1-yl)carbamate (3.09 g, 12.01 mmol, 1.0 eq) in CH_2Cl_2 (400 ml) was added Grubbs Catalyst 2nd Generation (307 mg, 0.36 mmol, 3 mol%). The resulting solution was left to stir at room temperature for 5 days. The reaction mixture was concentrated *in vacuo* to give a brown oil. The brown oil was purified by flash chromatography (75% EtOAc in petroleum ether) to give *tert*-butyl *cis*-3,6-dihydroxy-2,3,6,7-tetrahydro-1*H*-azepine-1-carboxylate (less polar, eluted first, 472 mg, 17%) and *tert*-butyl *trans*-3,6-dihydroxy-2,3,6,7-tetrahydro-1*H*-azepine-1-carboxylate (more polar, eluted second, 469 mg, 17%) as brown oils. Both obtained as a mixture of rotamers. *Cis*-diol characterised.

^{1}H NMR (400 MHz, $CDCl_3$) δ_H/ppm: 5.75 (s, 2H), 4.40 – 4.24 (m, 2H), 3.87 (br. s, 1H), 3.82 – 3.71 (m, 2H), 3.30 (br. s, 1H), 3.22 – 3.14 (m, 2H), 1.44 (s, 9H); ^{13}C NMR (126 MHz, $CDCl_3$) δ_C/ppm: 155.8, 133.9, 80.7, 69.0, 53.3, 28.4. Consistent with data in the literature.[16]

tert-Butyl (3_R_,6_S_)-3,6-bis((diethoxyphosphoryl)oxy)-2,3,6,7-tetrahydro-1_H_-azepine-1-carboxylate (12a)

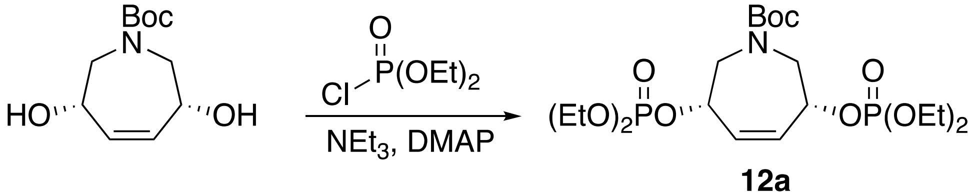

12a

To a solution of *tert*-butyl *cis*-3,6-dihydroxy-2,3,6,7-tetrahydro-1*H*-azepine-1-carboxylate (470 mg, 2.05 mmol, 1.0 eq) in CH_2Cl_2 (8.2 mL) was added NEt_3 (0.86 mL, 6.15 mmol, 3.0 eq) and DMAP (124 mg, 1.01 mmol, 0.5 eq). The resulting solution was cooled to 0 °C before dropwise addition of diethylchlorophosphate (0.89 mL, 6.15 mmol, 3.0 eq). The resulting mixture was left to stir at room temperature for 16 hours. The mixture was then quenched with H_2O (20 mL) and extracted with

CH_2Cl_2 (3 x 20 mL). The organic extracts were combined, washed with brine (50 mL), dried ($MgSO_4$) and evaporated *in vacuo* to give a yellow oil. The yellow oil was purified by flash chromatography (50-100% EtOAc in heptane) to give *tert*-Butyl (3*R*,6*S*)-3,6-bis((diethoxyphosphoryl)oxy)-2,3,6,7-tetrahydro-1*H*-azepine-1-carboxylate (**12a**) as a pale yellow oil (494 mg, 48% yield). Obtained as a mixture of rotamers.

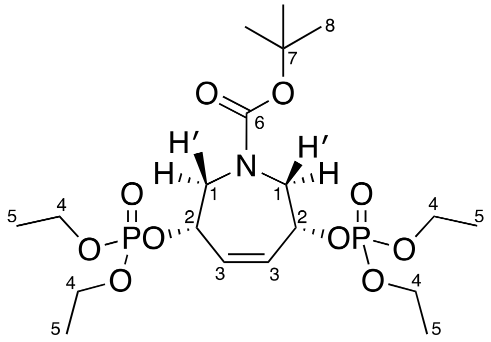

^{1}H NMR (400 MHz, DMSO-d_6, 338K) δ_H/ppm: 5.83 (s, 2H, 2CH-(3)), 4.92 – 4.86 (m, 2H, 2CH-(2)), 4.11 – 4.03 (m, 8H, 4CH_2-(4)), 4.02 (dd, *J* = 13.6, 4.3 Hz, 2H, 2CH***H*´**-(1)), 3.22 (dd, *J* = 13.6, 9.5 Hz, 2H, 2C***H***H´-(1)), 1.46 (s, 9H, 3CH_3-(8)), 1.29 (t, *J* = 6.9 Hz, 12H, 4CH_3-(5)); ^{13}C NMR (126 MHz, $CDCl_3$) δ_C/ppm: 154.41 (C(6)), 131.95 (C(3)), 131.69 (C(3)), 81.00 (C(2)), 74.18 (C(1)), 73.79 (C(1)), 64.13 (C(4)), 52.12 (C(7)), 51.33 (C(7)), 28.39 (C(8)), 16.25 (C(5)); ^{31}P NMR (162 MHz, $CDCl_3$) δ_P/ppm: –1.50; HRMS (ESI) *m/z* calcd for $C_{19}H_{37}NO_{10}P$ $[M+H]^+$: 501.18927, found: 501.19836; IR (ATR) ν (cm^{-1}) thin film, $CHCl_3$: 2888.4 (w), 1693.7 (s), 1465.7 (w), 1367.8 (w), 1254.0 (m), 1027.8 (s), 989.6 (m), 806.3 (w), 701.2 (w)

Desymmetrization Products

Dimethyl ((1*R*,2*R*)-2-(4-phenylbutyl)cyclopent-3-en-1-yl) phosphate

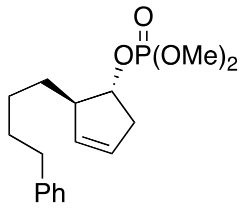

Chemical Formula: $C_{17}H_{25}O_4P$
Molecular Weight: 324.3568

Synthesised according to general procedure 1b, using *meso*-4-cyclopentene-1,3-bisdimethylphosphate and 4-phenyl-1-butene as the alkene partner (hydrozirconation time 20 mins). Purified by flash chromatography (20-50% EtOAc in petroleum ether) to give dimethyl ((1*R*,2*R*)-2-(4-phenylbutyl)cyclopent-3-en-1-yl) phosphate as a yellow oil (71 mg, 55%).

HPLC analysis indicated an enantiomeric excess of 86% [Chiralpak® IA; flow: 1 mL/min; hexane/iPrOH: 94:6; λ = 210 nm; major enantiomer, t_R = 8.075 min; minor enantiomer, t_R = 10.003 min].

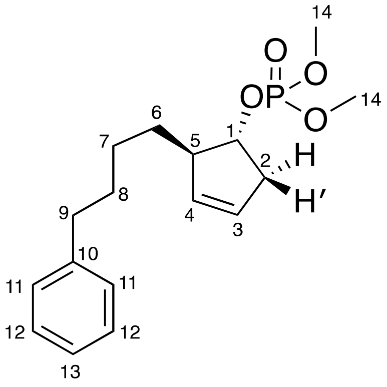

^{1}H NMR (400 MHz, $CDCl_3$) δ_H/ppm: 7.33 – 7.24 (m, 2H, ArH-(12)), 7.21 – 7.14 (m, 3H, ArH-(11, 13)), 5.77 – 5.58 (m, 2H, 2CH-(3, 4)), 4.68 (tt, *J* = 6.3, 2.9 Hz, 1H, CH-(1)), 3.74 (dd, *J* = 11.1, 0.9 Hz, 6H, $2CH_3$-(14)), 2.87 – 2.71 (m, 2H, C***H***H′-(2), CH-(5)), 2.66 – 2.57 (m, *J* = 7.7 Hz, 2H, CH_2-(9)), 2.54 – 2.44 (m, *J* = 17.8 Hz, 1H, C H***H′***-(2)), 1.73 – 1.58 (m, 2H, CH_2-(6)), 1.53 – 1.27 (m, 4H, $2CH_2$-(7, 8).; ^{13}C NMR (101 MHz, $CDCl_3$) δ_C/ppm: 142.7 (C(10)), 132.8 (C(4)), 128.5 (C(12)), 128.4 (C(11)), 127.3 (C(3)), 125.8 (C(13)), 83.5 (C(1)), 54.3 (C(14)), 53.06 (C(5)), 39.9 (C(2)), 36.0

(C(9)), 32.7 (C(6)), 31.7 (C(8)), 27.2 (C(7)); ^{31}P NMR (162 MHz, $CDCl_3$) δ_P/ppm: 0.76; HRMS (ESI) *m/z* calcd for $C_{17}H_{25}O_4P$ $[M+H]^+$: 325.149, found: 325.1569; IR (ATR) ν (cm^{-1}) thin film, $CHCl_3$: 2929.8 (w), 2584.7 (w), 1453.9 (w), 1218.4 (m), 1186.0 (w), 1035.0 (s), 922.3 (w), 848.1 (m), 750.0 (w), 700.8 (w); $[\alpha]^{25}_{589}$ = -72.6 (c=1.0 in $CHCl_3$, 86% ee).

Diethyl ((1*R*,2*R*)-2-(4-phenylbutyl)cyclopent-3-en-1-yl) phosphate (1b)

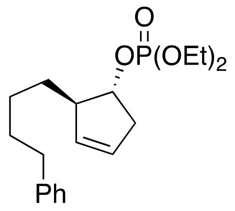

1b

Chemical Formula: $C_{19}H_{29}O_4P$
Molecular Weight: 352.4108

Synthesised according to general procedure 1b, using *meso*-4-cyclopentene-1,3-bisdiethylphosphate (**1a**) and 4-phenyl-1-butene as the alkene partner (hydrozirconation time 20 mins). Purified by flash chromatography (20-40% EtOAc in petroleum ether) to give **1b** as a yellow oil (93 mg, 66%).

HPLC analysis indicated an enantiomeric excess of 90% [Chiralpak® IA; flow: 1.0 mL/min; hexane/iPrOH: 95:5; λ = 210 nm; major enantiomer, t_R = 8.687 min; minor enantiomer, t_R = 10.302 min].

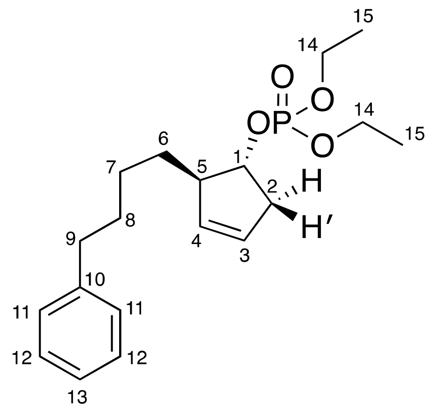

^{1}H NMR (400 MHz, $CDCl_3$) δ_H/ppm: 7.31 – 7.22 (m, 2H, ArH-(12)), 7.22 – 7.13 (m, 3H, ArH-(11, 13)), 5.66 (dq, *J* = 6.0, 1.8 Hz, 2H, 2CH-(3, 4)), 4.67 (tt, *J* = 6.4, 3.0 Hz, 1H, CH-(1)), 4.09 (p, *J* = 7.2 Hz, 4H, $2CH_2$-(14)), 2.86 – 2.69 (m, 2H, C***H***H′-(2),

CH-(5)), 2.67 – 2.57 (m, *J* = 7.7 Hz, 2H, CH_2-(9)), 2.53 – 2.46 (m, 1H, CH***H*´**-(2)), 1.72 – 1.56 (m, 2H, CH_2-(6)), 1.56 – 1.38 (m, 4H, 2CH_2-(7, 8)), 1.33 (t, *J* = 7.3 Hz, 6H, 2CH_3-(15)); ^{13}C NMR (101 MHz, $CDCl_3$) δ_C/ppm: 142.7 (C(10)), 132.7 (C(4)), 128.4 (C(12)), 128.3 (C(11)), 127.3 (C(3)), 125.7 (C(13)), 83.1 (C(1)), 63.6 (C(14)), 53.0 (C(5)), 39.9 (C(2)), 35.9 (C(9)), 32.6 (C(6)), 31.7 (C(8)), 27.2 (C(7)), 16.3 (C(15)); ^{31}P NMR (162 MHz, $CDCl_3$) δ_P/ppm: -1.45; HRMS (ESI) *m/z* calcd for $C_{19}H_{29}O_4P$ $[M+H]^+$: 353.1803, found: 353.1883; IR (ATR) ν (cm^{-1}) thin film, $CHCl_3$: 2930.2 (w), 2586.7 (w), 1453.8 (w), 1369.2 (w), 1262.6 (m), 1165.9 (w), 1028.6 (s), 976.8 (m), 820.8 (w), 748.9 (w), 700.4 (w); $[\alpha]^{25}_{589}$ = -65.6 (c=1.0 in $CHCl_3$, 90% ee).

Diisopropyl ((1*R*,2*R*)-2-(4-phenylbutyl)cyclopent-3-en-1-yl) phosphate

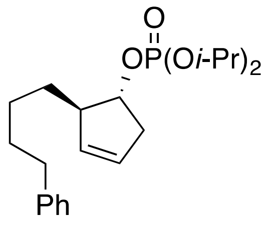

2c

Chemical Formula: $C_{21}H_{33}O_4P$
Molecular Weight: 380.4648

Synthesised according to general procedure 1b, using *meso*-4-cyclopentene-1,3-bisdiisopropylphosphate and 4-phenyl-1-butene as the alkene partner (hydrozirconation time 20 mins). Purified by flash chromatography (30-40% EtOAc in petroleum ether) to give diisopropyl ((1*R*,2*R*)-2-(4-phenylbutyl)cyclopent-3-en-1-yl) phosphate as a yellow oil (69 mg, 45%).

HPLC analysis indicated an enantiomeric excess of 84% [Chiralpak® IA; flow: 1 mL/min; hexane/iPrOH: 96:4; λ = 210 nm; major enantiomer, t_R = 7.985 min; minor enantiomer, t_R = 10.348 min].

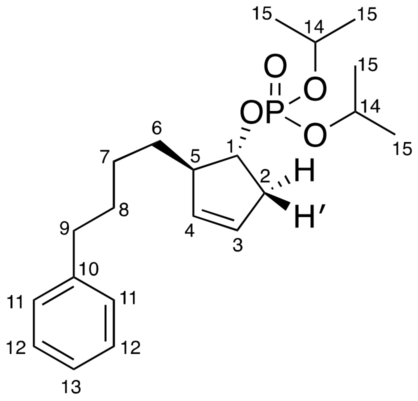

^{1}H NMR (400 MHz, $CDCl_3$) δ_H/ppm: 7.30 – 7.24 (m, 2H, ArH-(12)), 7.20 – 7.13 (m, 3H, 2ArH-(11, 13)), 5.65 (dq, J = 6.0, 1.9 Hz, 2H, 2CH-(3, 4)), 4.69 – 4.57 (m, 3H, 3CH-(1, 14)), 2.81 – 2.70 (m, 2H, C***H***H´-(2), CH-(5)), 2.64 – 2.57 (m, 2H, CH_2-(9)), 2.50 (dd, J = 17.5, 1.8 Hz, 1H, CH***H´***-(2)), 1.64 – 1.58 (m, 2H, CH_2-(6)), 1.53 – 1.38 (m, 4H, 2CH_2-(7, 8)), 1.32 (d, J = 6.3 Hz, 12H, 4CH_3-(15)); ^{13}C NMR (101 MHz, $CDCl_3$) δ_c/ppm: 142.7 (C(10)), 132.7 (C(4)), 128.5 (C(12)), 128.4 (C(11)), 127.4 (C(3)), 125.8 (C(13)), 82.9 (C(1)), 72.3 (C(14)), 53.0 (C(5)), 39.9 (C(2)), 36.0 (C(9)), 32.8 (C(6)), 31.8 (C(8)), 27.3 (C(7)), 23.8 (C(15)); ^{31}P NMR (162 MHz, $CDCl_3$) δ_P/ppm: -3.05; HRMS (ESI) *m/z* calcd for $C_{21}H_{33}O_4NaP$ $[M+Na]^+$: 403.2009, found: 403.2002; IR (ATR) ν (cm^{-1}) thin film, $CHCl_3$: 2980.6 (m), 2931.3 (w), 1454.3 (w), 1385.4 (w), 1260.3 (m), 1143.1 (w), 999.2 (s), 700.1 (w).; $[\alpha]^{25}_{589}$ = -63.1 (c=1.0 in $CHCl_3$, 84% ee).

Diphenyl ((1*R*,2*R*)-2-(4-phenylbutyl)cyclopent-3-en-1-yl) phosphate

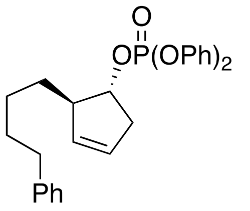

Chemical Formula: $C_{27}H_{29}O_4P$
Molecular Weight: 448.4988

Synthesised according to general procedure 1b, using *meso*-4-cyclopentene-1,3-bisdiphenylphosphate and 4-phenyl-1-butene as the alkene partner (hydrozirconation time 20 mins). Purified by flash chromatography (10% EtOAc in petroleum ether with 1% NEt_3) on silica which was pre-washed with 2% NEt_3 in petroleum ether solution

to give diphenyl ((1*R*,2*R*)-2-(4-phenylbutyl)cyclopent-3-en-1-yl) phosphate as a yellow oil (121 mg, 68%).

HPLC analysis indicated an enantiomeric excess of 70% [Chiralpak® IA; flow: 1 mL/min; hexane/iPrOH: 90:10; λ = 210 nm; major enantiomer, t_R = 13.473 min; minor enantiomer, t_R = 17.902 min].

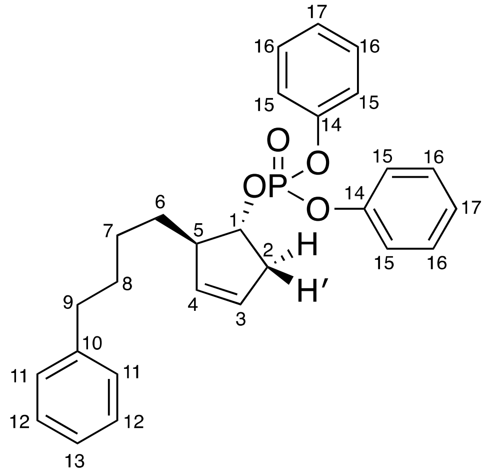

^{1}H NMR (400 MHz, $CDCl_3$) δ_H/ppm: 7.35 – 7.12 (m, 15H, ArH-(11, 12, 13, 15, 16, 17)), 5.65 (dq, *J* = 6.0, 1.8 Hz, 2H, 2CH-(3, 4)), 4.94 – 4.88 (m, *J* = 6.4, 3.8, 2.9 Hz, 1H, CH-(1)), 2.82 – 2.71 (m, 2H, C***H***H´-(2), CH-(5)), 2.58 – 2.53 (m, 2H, CH_2-(9)), 2.53 – 2.46 (m, 1H, CH***H´***-(2)), 1.62 – 1.52 (m, 2H, CH_2-(6)), 1.43 – 1.25 (m, 4H, 2CH_2-(7, 8)); ^{13}C NMR (101 MHz, $CDCl_3$) δ_C/ppm: 150.6 (C(14)), 142.6 (C(10), 132.6 (C(4)), 129.8 (C(12)), 128.4 (C(11)), 128.3 (C(13)), 127.1 (C(3)), 125.7 (C(16)), 125.3 (C(17)), 120.2 (C(15)), 85.1 (C(1)), 52.9 (C(5)), 39.7 (C(2)), 35.8 (C(9)), 32.4 (C(6)), 31.6 (C(8)), 27.1 (C(7)); ^{31}P NMR (162 MHz, $CDCl_3$) δ_P/ppm: -12.3; HRMS (ESI) *m/z* calcd for $C_{27}H_{29}O_4NaP$ $[M+Na]^+$: 471.1696, found: 471.1688; IR (ATR) ν (cm^{-1}) thin film, $CHCl_3$: 2929.9 (w), 2856.3 (w), 1591.2 (w), 1489.3 (m), 1455.3 (w), 1286.5 (m), 1221.1 (w), 1190.9 (s), 1163.1 (w), 1022.4 (m), 948.6 (s), 753.9 (m), 689.2 (m); $[\alpha]^{25}_{589}$ = -36.1 (c=1.0 in $CHCl_3$, 70% ee).

Diethyl ((1*R*,2*R*)-2-(4-phenylbutyl)cyclohex-3-en-1-yl) phosphate (2b)

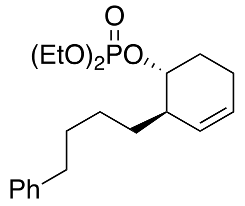

2b

Chemical Formula: $C_{20}H_{31}O_4P$
Molecular Weight: 366.4378

Synthesised according to general procedure 1b, using *meso*-5-cyclohexene-1,4-bisdiethylphosphate (**2a**) and 4-phenyl-1-butene as the alkene partner (hydrozirconation time 20 mins). Purified by flash chromatography (40-50% EtOAc in petroleum ether) to give **2b** as a yellow oil (102 mg, 79%).

HPLC analysis indicated an enantiomeric excess of 91% [Chiralpak® IB; flow: 1 mL/min; hexane/iPrOH: 98:2; λ = 210 nm; major enantiomer, t_R = 9.164 min; minor enantiomer, t_R = 9.966 min].

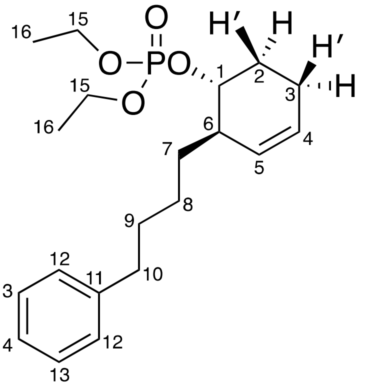

^{1}H NMR (400 MHz, $CDCl_3$) δ_H/ppm: 7.31 – 7.23 (m, 2H, ArH-(13), 7.20 – 7.13 (m, 3H, ArH-(12, 14)), 5.69 – 5.61 (m, 1H, CH-(4)), 5.54 – 5.47 (m, 1H, CH-(5)), 4.33 (dtd, *J* = 9.4, 6.7, 2.9 Hz, 1H, CH-(1)), 4.09 (p, *J* = 7.1 Hz, 4H, $2CH_2$-(15)), 2.61 (t, *J* = 7.7 Hz, 2H, CH_2-(10)), 2.30 – 2.20 (m, 1H, CH-(6)), 2.20 – 2.13 (m, 1H, C***H***H′-(3)), 2.13 – 2.07 (m, 1H, CH***H′***-(3)), 2.07 – 1.99 (m, 1H, C***H***H′-(2)), 1.87 – 1.76 (m, 1H, CH***H′***-(2)), 1.68 – 1.34 (m, 6H, $3CH_2$-(7, 8, 9)), 1.32 (tdd, *J* = 7.1, 2.8, 1.0 Hz, 6H, $2CH_3$-(16)); ^{13}C NMR (101 MHz, $CDCl_3$) δ_C/ppm: 142.7 (C(11)), 128.5 (C(13)), 128.4 (C(12)), 128.1 (C(5)), 126.4 (C(4)), 125.7 (C(14)), 78.4 (C(1)), 63.6 (C(15)),

41.8 (C(6)), 36.0 (C(10)), 32.5 (C(7)), 31.9 (C(9)), 27.8 (C(2)), 26.1 (C(8)), 23.3 (C(3)), 16.2 (C(16)); ^{31}P NMR (162 MHz, $CDCl_3$) δ_P/ppm: -1.47; HRMS (ESI) *m/z* calcd for $C_{20}H_{31}O_4NaP$ $[M+Na]^+$: 389.18522, found: 389.18602; IR (ATR) ν (cm^{-1}) thin film, $CHCl_3$: 2981.0 (w), 2931.1 (w), 2858.5 (w), 1262.0 (m), 1030.7 (s), 1010.4 (s), 967.4 (m), 817.8 (w), 784.2 (w), 700.0 (w); $[\alpha]^{25}_{589}$ = -70.2 (c=1.0 in $CHCl_3$, 95% ee).

Diethyl ((1*R*,2*R*)-2-(4-phenylbutyl)cyclohept-3-en-1-yl) phosphate (3b)

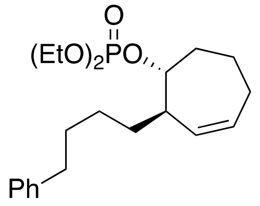

3b

Chemical Formula: $C_{21}H_{33}O_4P$
Molecular Weight: 380.4648

Synthesised according to general procedure 1b, using *meso*-6-cycloheptene-1,5-bisdiethylphosphate (**3a**) and 4-phenyl-1-butene as the alkene partner (hydrozirconation time 20 mins). Purified by flash chromatography (40-50% EtOAc in petroleum ether) to give **3b** as a yellow oil (92 mg, 60%).

HPLC analysis indicated an enantiomeric excess of 92% [Chiralpak® IC; flow: 1 mL/min; hexane/iPrOH: 96:4; λ = 210 nm; major enantiomer, t_R = 26.044 min; minor enantiomer, t_R = 29.793 min].

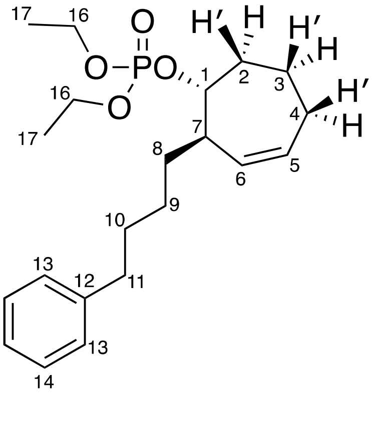

^{1}H NMR (400 MHz, $CDCl_3$) δ_H/ppm: 7.30 – 7.24 (m, 2H, ArH-(14)), 7.21 – 7.14 (m, 3H, ArH-(13, 15)), 5.84 (dtd, *J* = 11.2, 6.3, 1.6 Hz, 1H, CH-(5)), 5.41 (dd, *J* = 11.2, 4.6 Hz, 1H, CH-(6)), 4.26 (qd, *J* = 8.1, 3.1 Hz, 1H, CH-(1)), 4.08 (pd, *J* = 7.4, 3.1 Hz, 4H, 2CH_2-(16)), 2.60 (td, *J* = 8.1, 2.3 Hz, 2H, CH_2-(11)), 2.57 – 2.49 (m, 1H, CH-(7)), 2.25 – 2.18 (m, 1H, C***H***H´-(2)), 2.09 (q, *J* = 6.0 Hz, 2H, C***HH´***-(4)), 1.97 – 1.88 (m, 1H, CH***H´***-(2)), 1.78 – 1.69 (m, 1H, C***H***H´-(3)), 1.68 – 1.57 (m, 3H, 2CH_2-(8, 9)), 1.54 – 1.38 (m, 3H, CH***H´***(3), 3CH_2-(9, 10)), 1.32 (tt, *J* = 7.1, 1.2 Hz, 6H, 2CH_3-(17)) 1.32 - 1.24 (m, 1H, CH_2-(10)); ^{13}C NMR (101 MHz, $CDCl_3$) δ_C/ppm: 142.8 (C(12)), 133.0 (C(5)), 131.4 (C(6)), 128.5 (C(13)), 128.4 (C(14)), 125.7 (C(15)), 79.5 (C(1)), 63.6 (C(16)), 44.5 (C(7)), 36.4 (C(2)), 36.0 (C(11)), 31.84 (C(9)), 31.76 (C(8)), 28.1 (C(4)), 26.7 (C(10)), 23.0 (C(3)), 16.3 (C(17)); ^{31}P NMR (162 MHz, $CDCl_3$) δ_P/ppm: -1.74; HRMS (ESI) *m/z* calcd for $C_{21}H_{33}O_4NaP$ $[M+Na]^+$: 403.20087, found: 403.20154; IR (ATR) ν (cm^{-1}) thin film, $CHCl_3$: 2930.1 (w), 2858.0 (w), 1453.3 (w), 1392.8 (w), 1261.0 (m), 1032.3 (s), 997.3 (s), 802.3 (w), 748.7 (w), 699.9 (w); $[\alpha]^{25}_{589}$ = -41.3 (c=1.0 in $CHCl_3$, 92% ee).

Diethyl ((1*R*,2*R*)-2-ethylcyclopent-3-en-1-yl) phosphate (1c)

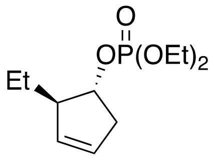

1c

Chemical Formula: $C_{11}H_{21}O_4P$
Molecular Weight: 248.2588

Synthesised according to general procedure 1b, using *meso*-4-cyclopentene-1,3-bisdiethylphosphate (**1a**) and ethylene as the alkene partner (hydrozirconation and reaction carried out under an ethylene atmosphere). Purified by flash chromatography (30-50% EtOAc in petroleum ether) to give **1c** as a yellow oil (81 mg, 82%).

The product was derivatised according to general procedure 2 for HPLC analysis.

HPLC analysis indicated an enantiomeric excess of 90% [Chiralpak® ID; flow: 0.7 mL/min; hexane/iPrOH: 99:1; λ = 210 nm; minor enantiomer, t_R = 14.157 min; major enantiomer, t_R = 14.785 min].

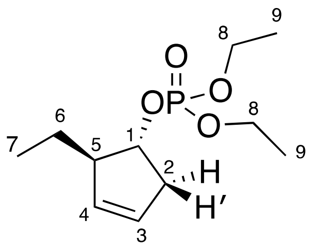

^{1}H NMR (500 MHz, $CDCl_3$) δ_H/ppm: 5.68 – 5.65 (m, 1H, CH-(4)), 5.64 – 5.61 (m, 1H, CH-(3)), 4.66 (tt, *J* = 6.6, 3.1 Hz, 1H, CH-(1)), 4.08 (dtd, *J* = 14.6, 7.1, 3.2 Hz, 4H, $2CH_2$-(8)), 2.77 – 2.72 (m, 1H, C***H***H´-(2)), 2.73 – 2.67 (m, 1H, CH-(5)), 2.51 – 2.44 (m, 1H, CH***H´***-(2)), 1.52 – 1.39 (m, 1H, CHH´-(6)), 1.39 – 1.33 (m, 1H, CHH´-(6)), 1.31 (tdd, *J* = 7.1, 2.3, 1.0 Hz, 6H, CH_3-(9)), 0.93 (t, *J* = 7.5 Hz, 3H, $2CH_3$-(7)); ^{13}C NMR (126 MHz, $CDCl_3$) δ_C/ppm: 132.5 (C(4)), 127.3 (C(3)), 82.9 (C(1), 63.7 (C(8)), 54.6 (C(5)), 40.0 (C(2)), 25.6 (C(6)), 16.3 (C(9)), 11.8 (C(7)); ^{31}P NMR (202 MHz, $CDCl_3$) δ_P/ppm: -1.46; HRMS (ESI) *m/z* calcd for $C_{21}H_{33}O_4NaP$ $[M+Na]^+$: 271.10697, found: 271.10697; IR (ATR) ν (cm^{-1}) thin film, $CHCl_3$: 2980.1 (w), 1460.5 (w), 1392.7 (w), 1263.4 (m), 1166.2 (w), 1026.8 (s), 974.6 (s), 887.0 (w), 820.82 (w), 715.4 (w); $[\alpha]^{25}_{589}$ = -71.1 (c=1.0 in $CHCl_3$, 90% ee). Optical rotation value consistent with those found in the literature.[5]

Diethyl ((1*R*,2*R*)-2-ethylcyclohex-3-en-1-yl) phosphate (2c)

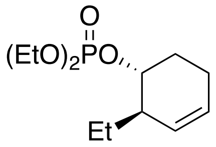

2c

Chemical Formula: $C_{12}H_{23}O_4P$
Molecular Weight: 262.2858

Synthesised according to general procedure 1b, using *meso*-5-cyclohexene-1,4-bisdiethylphosphate (**2a**) and ethylene as the alkene partner (hydrozirconation and reaction carried out under an ethylene atmosphere). Purified by flash chromatography (30-50% EtOAc in petroleum ether) to give **2c** as a yellow oil (83 mg, 79%).

The product was derivatised according to general procedure 2 for HPLC analysis.

HPLC analysis indicated an enantiomeric excess of 97% [Chiralpak® ID; flow: 0.7 mL/min; hexane/iPrOH: 99:1; λ = 210 nm; major enantiomer, t_R = 13.323 min ; minor enantiomer, t_R = 14.305 min].

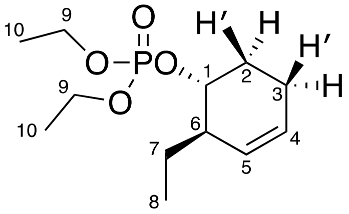

^{1}H NMR (500 MHz, $CDCl_3$) δ_H/ppm: 5.69 – 5.63 (m, 1H, CH-(4)), 5.54 – 5.48 (m, 1H, CH-(5)), 4.36 – 4.30 (m, 1H, CH-(1)), 4.14 – 4.05 (m, 4H, $2CH_2$-(9)), 2.23 – 2.17 (m, 1H, CH-(6)), 2.17 – 2.13 (m, 1H, C***H***H′-(3)), 2.12 – 2.07 (m, 1H, CH***H′***-(3)), 2.07 – 2.01 (m, 1H, C***H***H′-(2)), 1.84 – 1.76 (m, 1H, CH***H′***-(2)), 1.65 – 1.55 (m, 1H, CH_2-(7)), 1.42 – 1.35 (m, 1H, CH_2-(7)), 1.33 (td, *J* = 7.1, 1.0 Hz, 6H, $2CH_3$-(10)), 0.93 (t, *J* = 7.5 Hz, 3H, CH_3-(8)); ^{13}C NMR (126 MHz, $CDCl_3$) δ_c/ppm: 128.0 (C(5)), 126.5 (C(4)), 78.0 (C(1)), 63.7 (C(9)), 43.3 (C(6)), 28.0 (C(2)), 25.2 (C(7)), 23.5 (C(3)), 16.3 (C(10)), 10.7 (C(8)); ^{31}P NMR (202 MHz, $CDCl_3$) δ_P/ppm: -1.49; HRMS (ESI) *m/z* calcd for $C_{21}H_{33}O_4NaP$ $[M+Na]^+$: 285.12262, found: 285.12257; IR (ATR) ν (cm^{-1}) thin film, $CHCl_3$: 3024.8 (w), 2934.2 (w), 1444.3 (w), 1262.7 (m), 1166.6 (w), 1100.9 (w), 1026.5 (s), 1008.2 (s), 987.3 (m), 819.0 (w), 726.3 (w), 682.0 (w); $[\alpha]^{25}_{589}$ = -51.8 (c=1.0 in $CHCl_3$, 97% ee). Optical rotation value consistent with those found in the literature.[5]

Diethyl ((1*R*,2*R*)-2-hexylcyclopent-3-en-1-yl) phosphate (1d)

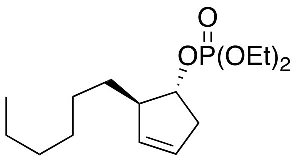

1d

Chemical Formula: $C_{15}H_{29}O_4P$
Molecular Weight: 304.3668

Synthesised according to general procedure 1b, using *meso*-4-cyclopentene-1,3-bisdiethylphosphate (**1a**) and 1-hexene as the alkene partner (hydrozirconation time

20 mins). Purified by flash chromatography (20-40% EtOAc in petroleum ether) to give **1d** as a yellow oil (78 mg, 64%).

GC analysis indicated an enantiomeric excess of 90% [Astec Chiraldex ß-DA (Supelco) column; initial temperature 140 ˚C, initial hold time 5 min, progress rate 0.5 ˚C/min, final temperature 160 ˚C, final hold time 15 min; Flow rate 2 mL/min; minor enantiomer, t_R = 46.800 min; major enantiomer, t_R = 47.341 min].

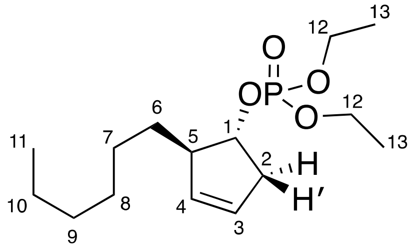

^{1}H NMR (400 MHz, $CDCl_3$) δ_H/ppm: 5.71 – 5.66 (m, 1H, CH-(4)), 5.66 – 5.62 (m, 1H, CH-(3)), 4.67 (tt, *J* = 6.4, 3.0 Hz, 1H, CH-(1)), 4.10 (pd, *J* = 7.2, 2.7 Hz, 4H, $2CH_2$-(12)), 2.80 – 2.71 (m, 2H, C***H***H´-(2), CH-(5)), 2.54 – 2.45 (m, 1H, CH***H*´**-(2)), 1.33 (tdd, *J* = 7.1, 2.2, 1.0 Hz, 6H, $2CH_3$-(13)), 1.47 – 1.18 (m, 10H, $5CH_2$-(6, 7, 8, 9, 10)), 0.90 – 0.84 (m, 3H, CH_3-(11)); ^{13}C NMR (101 MHz, $CDCl_3$) δ_c/ppm: 132.9 (C(4)), 127.2 (C(3)), 83.2 (C(1)), 63.7 (C(12)), 53.2 (C(5)), 40.0 (C(2)), 32.9 (C(6)), 31.9 (C(9)), 29.6 (C(8)), 27.5 (C(7)), 22.8 (C(10)), 16.3 (C(13)), 14.2 (C(11)); ^{31}P NMR (162 MHz, $CDCl_3$) δ_P/ppm: -1.46; HRMS (ESI) *m/z* calcd for $C_{15}H_{29}O_4NaP$ $[M+Na]^+$: 327.16957, found: 327.16953; IR (ATR) ν (cm^{-1}) thin film, $CHCl_3$: 2926.9 (w), 2856.2 (w), 1459.2 (w), 1392.7 (w), 1263.7 (m), 1165.8 (w), 1030.2 (s), 973.6 (m), 820.6 (w), 712.4 (w); $[\alpha]^{25}_{589}$ = -63.5 (c=1.0 in $CHCl_3$, 90% ee).

Diethyl ((1*R*,2*R*)-2-hexylcyclohex-3-en-1-yl) phosphate (2d)

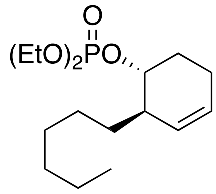

2d

Chemical Formula: $C_{16}H_{31}O_4P$
Molecular Weight: 318.3938

Synthesised according to general procedure 1b, using *meso*-5-cyclohexene-1,4-bisdiethylphosphate (**2a**) and 1-hexene as the alkene partner (hydrozirconation time 20 mins). Purified by flash chromatography (20-40% EtOAc in petroleum ether) to give **2d** as a yellow oil 111 mg, 87%).

GC analysis indicated an enantiomeric excess of 97% [Astec Chiraldex ß-DA (Supelco) column; initial temperature 160 ˚C, initial hold time 5 min, progress rate 0.1 ˚C/min, final temperature 165 ˚C, final hold time 10 min; Flow rate 2 mL/min; minor enantiomer, t_R = 44.980 min; major enantiomer, t_R = 45.671 min].

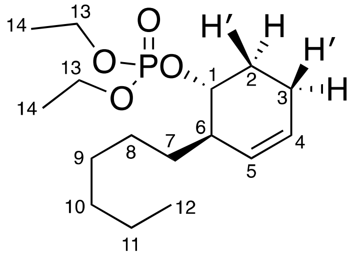

^{1}H NMR (400 MHz, $CDCl_3$) δ_H/ppm: 5.68 – 5.60 (m, 1H, CH-(4)), 5.56 – 5.48 (m, 1H, CH-(5)), 4.33 (dtd, *J* = 9.3, 6.7, 2.9 Hz, 1H, CH-(1)), 4.16 – 4.03 (m, 4H, $2CH_2$-(13)), 2.29 – 2.20 (m, 1H, CH-(6)), 2.20 – 2.12 (m, 1H, C***H***H´-(3)), 2.11 – 2.06 (m, 1H, CH***H´***-(3)), 2.06 – 1.97 (m, 1H, C***H***H´-(2)), 1.86 – 1.75 (m, 1H, CH***H´***-(2)), 1.59 – 1.16 (m, 10H, $5CH_2$-(7, 8, 9, 10, 11)), 1.33 (td, *J* = 7.1, 1.0 Hz, 6H, $2CH_3$-(14)), 0.91 – 0.84 (m, 3H, CH_3-(12)); ^{13}C NMR (101 MHz, $CDCl_3$) δ_C/ppm: 128.3 (C(5)), 126.3 (C(4)), 78.4 (C(1)), 63.6 (C(13)), 41.8 (C(6)), 32.7 (C(7)), 31.9 (C(10)), 29.7 (C(9)), 27.7 (C(2)), 26.3 (C(8)), 23.3 (C(3)), 22.8 (C(11)), 16.3 (C(14)), 14.2 (C(12)); ^{31}P NMR (162 MHz, $CDCl_3$) δ_P/ppm: -1.49; HRMS (ESI) *m/z* calcd for $C_{16}H_{31}O_4NaP$ $[M+Na]^+$: 341.18522, found: 341.18537; IR (ATR) ν (cm^{-1}) thin film, $CHCl_3$: 2928.1

(m), 2857.7 (w), 1458.2 (w), 1393.3 (w), 1263.1 (m), 1166.1 (w), 1011.0 (s), 974.6 (m), 817.9 (w), 726.3 (w); $[\alpha]^{25}_{589}$ = -71.5 (c=1.0 in $CHCl_3$, 97% ee).

Diethyl ((1*R*,2*R*)-2-tetradecylcyclopent-3-en-1-yl) phosphate (1e)

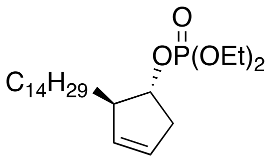

1e

Chemical Formula: $C_{23}H_{45}O_4P$
Molecular Weight: 416.5828

Synthesised according to general procedure 1b, using *meso*-4-cyclopentene-1,3-bisdiethylphosphate (**1a**) and 1-tetradecene as the alkene partner (hydrozirconation time 20 mins). Purified by flash chromatography (15-35% EtOAc in petroleum ether) to give **1e** as a yellow oil (92 mg, 55%).

The product was derivatised according to general procedure 2 for HPLC analysis.

HPLC analysis indicated an enantiomeric excess of 90% [Chiralpak® IC; flow: 0.7 mL/min; hexane/iPrOH: 99:1; λ = 210 nm; major enantiomer, t_R = 11.584 min ; minor enantiomer, t_R = 12.143 min].

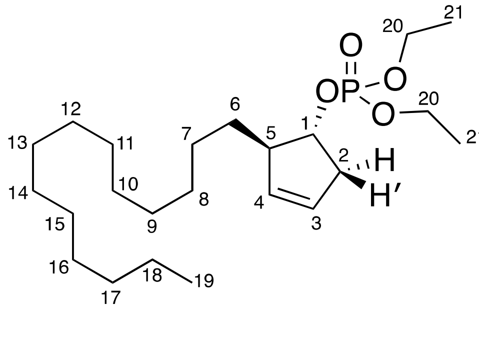

^1H NMR (400 MHz, $CDCl_3$) δ_H/ppm: 5.71 – 5.66 (m, 1H, CH-(4)), 5.65 – 5.61 (m, 1H, CH-(3)), 4.66 (tt, *J* = 6.4, 3.0 Hz, 1H, (CH-(1)), 4.09 (pd, *J* = 7.2, 2.7 Hz, 4H, $2CH_2$-(20)), 2.81 – 2.71 (m, 2H, C***H***H′-(2), CH-(5)), 2.53 – 2.44 (m, 1H, CH***H′***-(2)), 1.37 – 1.30 (m, 6H, $2CH_3$-(21)), 1.46 – 1.21 (m, 26H, $13CH_2$-(6-18)), 0.92 – 0.83 (m, 3H, CH_3-(19)); ^{13}C NMR (101 MHz, $CDCl_3$) δ_c/ppm: 132.9 (C(4)), 127.2 (C(3)), 83.3

(C(1)), 63.7 (C(20)), 53.2 (C(5)), 40.0 (C(2)), 32.9 (C(6)), 32.1 (C(7-18), 29.9 (C(7-18)), 29.8 (C(7-18)), 29.8 (C(7-18)), 29.8 (C(7-18)), 29.7 (C(7-18), 29.5 (C(7-18)), 27.6 (C(7-18)), 22.8 (C(7-18)), 16.3 (C(21)), 14.3 (C(19)); ^{31}P NMR (162 MHz, $CDCl_3$) δ_P/ppm: -1.46; HRMS (ESI) *m/z* calcd for $C_{23}H_{45}O_4NaP$ $[M+Na]^+$: 439.29477, found: 439.29420; IR (ATR) ν (cm^{-1}) thin film, $CHCl_3$: 2923.1(m), 2853.2 (w), 1465.9 (w), 1369.6 (w), 1264.1 (m), 1166.2 (w), 1030.3 (s), 974.4 (m), 820.6 (w), 712.6 (w); $[\alpha]^{25}_{589}$ = -56.0 (c=1.0 in $CHCl_3$, 90% ee).

Diethyl ((1*R*,2*R*)-2-tetradecylcyclohex-3-en-1-yl) phosphate (2e)

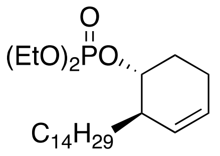

2e

Chemical Formula: $C_{24}H_{47}O_4P$
Molecular Weight: 430.6098

Synthesised according to general procedure 1b, using *meso*-5-cyclohexene-1,4-bisdiethylphosphate (**2a**) and 1-tetradecene as the alkene partner (hydrozirconation time 20 mins). Purified by flash chromatography (15-35% EtOAc in petroleum ether) to give **2e** as a yellow oil (128 mg, 74%).

The product was derivatised according to general procedure 2 for HPLC analysis.

HPLC analysis indicated an enantiomeric excess of 95% [Chiralpak® IC; flow: 0.7 mL/min; hexane/iPrOH: 99:1; λ = 210 nm; major enantiomer, t_R = 11.101 min ; minor enantiomer, t_R = 11.933 min].

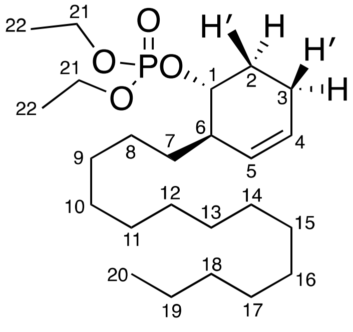

^1H NMR (400 MHz, $CDCl_3$) δ_H/ppm: 5.68 – 5.62 (m, 1H, CH-(4)), 5.55 – 5.49 (m, 1H, CH-(5)), 4.33 (dtd, *J* = 9.3, 6.7, 2.9 Hz, 1H, CH-(1)), 4.10 (pd, *J* = 7.2, 3.2 Hz, 4H, $2CH_2$-(21)), 2.29 – 2.21 (m, 1H, CH-(6)), 2.21 – 2.12 (m, 1H, C***H***H´-(3)), 2.12 – 2.06 (m, 1H, CH***H´***-(3)), 2.03 – 1.98 (m, 1H, C***H***H´-(2)), 1.86 – 1.75 (m, 1H, CH***H´***-(2)), 1.46 – 1.21 (m, 26H, $13CH_2$-(7-19)) 1.33 (td, *J* = 7.1, 1.0 Hz, 6H, $2CH_3$-(22)), 0.92 – 0.84 (m, 3H, CH_3-(20)); ^{13}C NMR (101 MHz, $CDCl_3$) δ_c/ppm: 128.3 (C(5)), 126.3 (C(4)), 78.4 (C(1)), 63.6 (C(21)), 41.8 (C(6)), 32.8 (C(7)), 32.1 (C(8-19)), 30.0 (C(8-19)), 29.8 (C(8-19), 29.8 (C(8-19)), 29.8 (C(8-19)), 29.7 (C(8-19)), 29.5 (C(8-19)), 27.7 (C(2)), 26.4 (C(8-19)), 23.3 (C(3)), 22.8 (C(8-19)), 16.3 (C(22)), 14.3 (C(20)); ^{31}P NMR (162 MHz, $CDCl_3$) δ_P/ppm: -1.49; HRMS (ESI) *m/z* calcd for $C_{24}H_{47}O_4NaP$ $[M+Na]^+$: 453.31042, found: 453.31042; IR (ATR) ν (cm^{-1}) thin film, $CHCl_3$: 2923.5 (m), 2853.4 (m), 1466.1 (w), 1264.3 (w), 1166.7 (w), 1034.2 (s), 1012.2 (s), 976.8 (m), 817.6 (w), 724.2 (w); $[\alpha]^{25}_{589}$ = -44.5 (c=1.0 in $CHCl_3$, 95% ee).

Diethyl ((1*R*,2*R*)-2-isopentylcyclopent-3-en-1-yl) phosphate (1f)

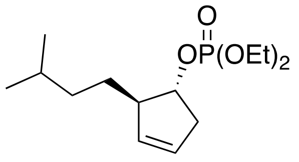

1f

Chemical Formula: $C_{14}H_{27}O_4P$
Molecular Weight: 290.3398

Synthesised according to general procedure 1b, using *meso*-4-cyclopentene-1,3-bisdiethylphosphate (**1a**) and 3-methyl-1-butene (reagent bottle under an Ar atmosphere and cooled to -78 ˚C before adding to flask **B**) as the alkene partner (hydrozirconation time 30 mins). Purified by flash chromatography (15-40% EtOAc in petroleum ether) to give **1f** as a yellow oil (70 mg, 60%).

GC analysis indicated an enantiomeric excess of 90% [Astec Chiraldex ß-DA (Supelco) column; initial temperature 120 ˚C, initial hold time 5 min, progress rate 2 ˚C/min, final temperature 170 ˚C, final hold time 10 min; Flow rate 3 mL/min; minor enantiomer, t_R = 27.398 min; major enantiomer, t_R = 27.708 min].

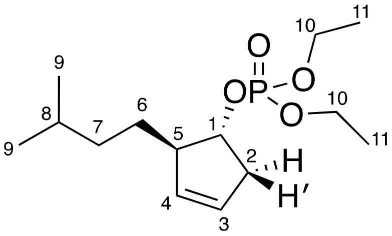

^{1}H NMR (400 MHz, $CDCl_3$) δ_H/ppm: 5.70 – 5.66 (m, 1H, CH-(4)), 5.66 – 5.61 (m, 1H, CH-(3)), 4.66 (tt, J = 6.5, 3.0 Hz, 1H, CH-(1)), 4.16 – 4.03 (m, 4H, $2CH_2$-(10)), 2.80 – 2.70 (m, 2H, C***H***H´-(2), CH-(5)), 2.53 – 2.44 (m, 1H, CH***H´***-(2)), 1.57 – 1.47 (m, 1H, CH-(8)), 1.47 – 1.16 (m, 4H, $2CH_2$-(6, 7)) 1.33 (tdd, J = 7.1, 2.2, 1.0 Hz, 6H, $2CH_3$-(11)), 0.86 (dd, J = 6.7, 1.2 Hz, 6H, $2CH_3$-(9)); ^{13}C NMR (101 MHz, $CDCl_3$) δ_c/ppm: 132.9 (C(4)), 127.2 (C(3)), 83.3 (C(1)), 63.7 (C(10)), 53.3 (C(5)), 40.0 (C(2)), 36.7 (C(7)), 30.6 (C(8)), 28.3 (C(6)), 22.7 (C(9)), 16.2 (C(11)); ^{31}P NMR (162 MHz, $CDCl_3$) δ_P/ppm: -1.47; HRMS (ESI) *m/z* calcd for $C_{14}H_{27}O_4NaP$ $[M+Na]^+$: 313.15392, found: 313.15373; IR (ATR) ν (cm^{-1}) thin film, $CHCl_3$: 2928.1 (w), 2869.4 (w), 1468.3 (w), 1367.5 (w), 1263.8 (m), 1167.4 (w), 1029.3 (s), 977.0 (m), 820.9 (w), 712.0 (w); $[\alpha]^{25}_{589}$ = -75.9 (c=1.0 in $CHCl_3$, 90% ee).

Diethyl ((1*R*,2*R*)-2-isopentylcyclohex-3-en-1-yl) phosphate (2f)

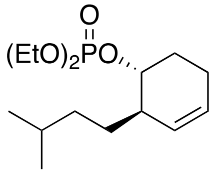

2f

Chemical Formula: $C_{15}H_{29}O_4P$
Molecular Weight: 304.3668

Synthesised according to general procedure 1b, using *meso*-5-cyclohexene-1,4-bisdiethylphosphate (**2a**) and 3-methyl-1-butene (reagent bottle under an Ar atmosphere and cooled to -78 ˚C before adding to flask **B**) as the alkene partner (hydrozirconation time 30 mins). Purified by flash chromatography (15-40% EtOAc in petroleum ether) to give **2f** as a yellow oil (95 mg, 78%).

GC analysis indicated an enantiomeric excess of 96% [Astec Chiraldex ß-DA (Supelco) column; initial temperature 120 ˚C, initial hold time 5 min, progress rate 2

°C/min, final temperature 170 °C, final hold time 10 min; Flow rate 3 mL/min; minor enantiomer, t_R = 33.344 min; major enantiomer, t_R = 33.699 min].

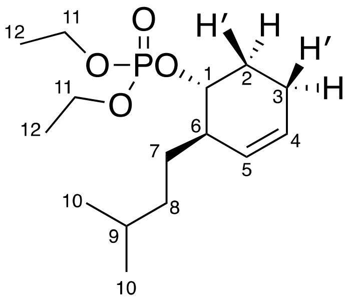

^{1}H NMR (400 MHz, $CDCl_3$) δ_H/ppm: 5.68 – 5.61 (m, 1H, CH-(4)), 5.54 – 5.48 (m, 1H, CH-(5)), 4.33 (dtd, *J* = 9.3, 6.7, 2.9 Hz, 1H, CH-(1)), 4.10 (pd, *J* = 7.2, 3.9 Hz, 4H, $2CH_2$-(11)), 2.27 – 2.18 (m, 1H, CH-(6)), 2.18 – 2.12 (m, 1H, C***H***H′-(3)), 2.12 – 2.05 (m, 1H, CH***H′***-(3)), 2.05 – 1.99 (m, 1H, C***H***H′-(2)), 1.86 – 1.74 (m, 1H, CH***H′***-(2)), 1.61 – 1.45 (m, 2H, CHH′ (7), CH-(9)), 1.36 – 1.12 (m, 3H, CHH′-(7) CH_2-(8)), 1.33 (td, *J* = 7.1, 1.0 Hz, 6H, $2CH_3$-(10)), 0.87 (dd, *J* = 6.6, 1.8 Hz, 6H, $2CH_3$-(12)); ^{13}C NMR (101 MHz, $CDCl_3$) δ_c/ppm: 128.3 (C(5)), 126.3 (C(4)), 78.4 (C(1)), 63.6 (C(13)), 42.0 (C(6)), 35.5 (C(8)), 30.5 (C(9)), 28.4 (C(7)), 27.8 (C(2)), 23.3 (C(3)), 22.7 (C(10)), 16.3 (C(12)); ^{31}P NMR (162 MHz, $CDCl_3$) δ_P/ppm: -1.49; HRMS (ESI) *m/z* calcd for $C_{15}H_{29}O_4NaP$ $[M+Na]^+$: 327.16957, found: 327.16949; IR (ATR) ν (cm^{-1}) thin film, $CHCl_3$: 2955.5 (w), 1458.1 (w), 1367.8 (w), 1263.6 (m), 1166.4 (w), 1011.0 (s), 973.9 (m), 817.5 (w), 726.0 (w); $[\alpha]^{25}_{589}$ = -76.8 (c=1.0 in $CHCl_3$, 96% ee).

Diethyl (1*R*,2*R*)-2-(3,3-dimethylbutyl)cyclopent-3-en-1-yl) phosphate (1g)

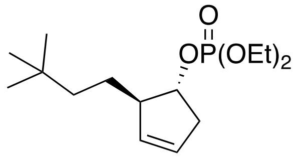

1g

Chemical Formula: $C_{15}H_{29}O_4P$
Molecular Weight: 304.3668

Synthesised according to general procedure 1b, using *meso*-4-cyclopentene-1,3-bisdiethylphosphate (**1a**) and 3,3-dimethyl-1-butene as the alkene partner

(hydrozirconation time 1hr 15 mins). Purified by flash chromatography (20-40% EtOAc in petroleum ether) to give **1g** as a yellow oil (59 mg, 48%).

GC analysis indicated an enantiomeric excess of 91% [Astec Chiraldex ß-DA (Supelco) column; initial temperature 120 ˚C, initial hold time 5 min, progress rate 4 ˚C/min, final temperature 170 ˚C, final hold time 5 min; Flow rate 3 mL/min; minor enantiomer, t_R = 22.524 min; major enantiomer, t_R = 22.903 min].

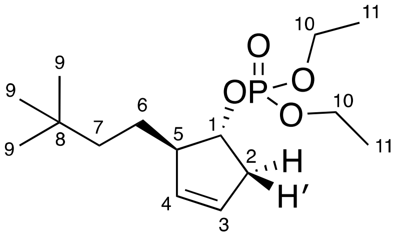

^{1}H NMR (400 MHz, $CDCl_3$) δ_H/ppm: 5.70 – 5.66 (m, 1H, CH-(4)), 5.65 – 5.61 (m, 1H, CH-(3)), 4.66 (tt, J = 6.5, 3.0 Hz, 1H, CH-(1)), 4.09 (pd, J = 7.1, 3.0 Hz, 4H, 4CH_2-(10)), 2.80 – 2.67 (m, 2H, C***H***H′-(2), CH-(5)), 2.53 – 2.45 (m, 1H, CH***H′***-(2)), 1.33 (tdd, J = 7.1, 2.3, 0.9 Hz, 6H, 2CH_3-(11)), 1.44 – 1.17 (m, 4H, 2CH_2-(6, 7)), 0.85 (s, 9H, 3CH_3-(9)); ^{13}C NMR (101 MHz, $CDCl_3$) δ_c/ppm: 132.9 (C(4)), 127.2 (C(3)), 83.2 (C(1)), 63.7 (C(10)), 53.7 (C(5)), 41.7 (C(8)), 40.0 (C(2)), 30.3 (C(7)), 29.4 (C(9)), 27.8 (C(6)), 16.3 (C(11)); ^{31}P NMR (162 MHz, $CDCl_3$) δ_P/ppm: -1.49; HRMS (ESI) *m/z* calcd for $C_{15}H_{29}O_4NaP$ $[M+Na]^+$: 327.16957, found: 327.16945; IR (ATR) ν (cm^{-1}) thin film, $CHCl_3$: 2952.4 (w), 2866.8 (w), 1476.1 (w), 1364.6 (w), 1263.0 (m), 1166.9 (w), 1033.9 (s), 1011.9 (s), 979.8 (m), 817.6 (w), 725.7 (w); $[\alpha]^{25}_{589}$ = -35.2 (c=1.0 in $CHCl_3$, 91% ee).

Diethyl (1*R*,2*R*)-2-(3,3-dimethylbutyl)cyclohex-3-en-1-yl) phosphate (2g)

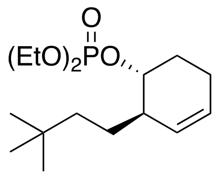

2g

Chemical Formula: $C_{16}H_{31}O_4P$
Molecular Weight: 318.3938

Synthesised according to general procedure 1b, using *meso*-5-cyclohexene-1,4-bisdiethylphosphate (**2a**) and 3,3-dimethyl-1-butene as the alkene partner

(hydrozirconation time 1hr 15 mins). Purified by flash chromatography (20-40% EtOAc in petroleum ether) to give **2g** as a yellow oil (80 mg, 63%).

GC analysis indicated an enantiomeric excess of 96% [Astec Chiraldex ß-DA (Supelco) column; initial temperature 120 ˚C, initial hold time 5 min, progress rate 4 ˚C/min, final temperature 170 ˚C, final hold time 15 min; Flow rate 3 mL/min; minor enantiomer, t_R = 28.911 min; major enantiomer, t_R = 29.547 min].

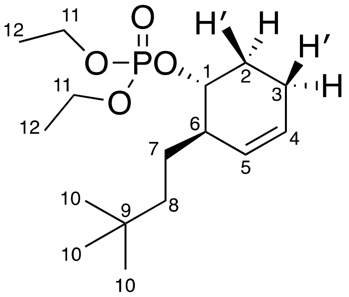

^1H NMR (400 MHz, $CDCl_3$) δ_H/ppm: 5.68 – 5.62 (m, 1H, CH-(4)), 5.53 – 5.47 (m, 1H, CH-(5)), 4.34 (dtd, *J* = 9.3, 6.7, 2.9 Hz, 1H, CH-(1)), 4.15 – 4.05 (m, 4H, $2CH_2$-(11)), 2.24 – 2.17 (m, 1H, CH-(6)), 2.17 – 2.12 (m, 1H, C***H***H´-(3)), 2.11 – 2.06 (m, 1H, CH***H´***-(3)), 2.06 – 2.00 (m, 1H, C***H***H´-(2)), 1.87 – 1.72 (m, 1H, CH***H´***-(2)), 1.60 – 1.47 (m, 1H, CHH´-(7)), 1.37 – 1.28 (m, 6H, $2CH_3$-(12)), 1.37 – 1.08 (m, 3H, CHH´-(7), CH_2-(8)), 0.86 (s, 9H, $3CH_3$-(10)); ^{13}C NMR (101 MHz, $CDCl_3$) δ_c/ppm: 128.3 (C(5)), 126.3 (C(4)), 78.4 (C(1)), 63.6 (C(11)), 42.3 (C(6)), 40.4 (C(9)), 30.4 (C(8)), 29.5 (C(10)), 27.9 (C(2)), 27.4 (C(7)), 23.4 (C(3)), 16.3 (C(12)); ^{31}P NMR (162 MHz, $CDCl_3$) δ_P/ppm: -1.49; HRMS (ESI) *m/z* calcd for $C_{16}H_{31}O_4NaP$ $[M+Na]^+$: 341.18522, found: 341.18521; IR (ATR) ν (cm^{-1}) thin film, $CHCl_3$: 2952.8 (w), 2867.0 (w), 1475.9 (w), 1393.6 (w), 1364.6 (w), 1262.9 (m), 1166.5 (w), 1033.0 (s), 1011.9 (s), 979.0 (m), 817.6 (w), 725.6 (w); $[\alpha]^{25}_{589}$ = -58.5 (c=1.0 in $CHCl_3$, 96% ee).

Diethyl (1*R*,2*R*)-2-(2-cyclohexylethyl)cyclopent-3-en-1-yl phosphate (1h)

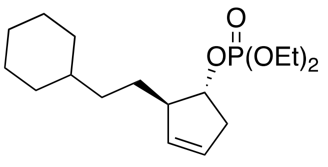

1h

Chemical Formula: $C_{17}H_{31}O_4P$
Molecular Weight: 330.4048

Synthesised according to general procedure 1b, using *meso*-4-cyclopentene-1,3-bisdiethylphosphate (**1a**) and vinylcyclohexane as the alkene partner (hydrozirconation time 30 mins). Purified by flash chromatography (15-40% EtOAc in petroleum ether) to give **1h** as a yellow oil (74 mg, 56%).

GC analysis indicated an enantiomeric excess of 91% [Astec Chiraldex ß-DA (Supelco) column; initial temperature 180 ˚C, initial hold time 5 min, progress rate 0.5 ˚C/min, final temperature 205 ˚C, final hold time 10 min; Flow rate 2 mL/min; minor enantiomer, t_R = 35.635 min; major enantiomer, t_R = 36.243 min].

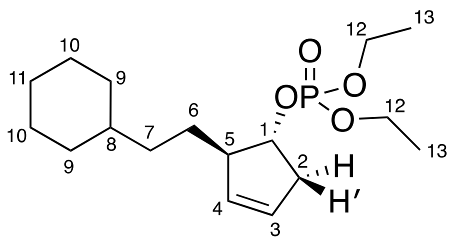

^{1}H NMR (400 MHz, $CDCl_3$) δ_H/ppm: 5.70 – 5.65 (m, 1H, CH-(4)), 5.65 – 5.61 (m, 1H, CH-(3)), 4.66 (tt, *J* = 6.5, 2.9 Hz, 1H, CH-(1)), 4.16 – 4.03 (m, 4H, 2CH_2-(12)), 2.79 – 2.69 (m, 2H, C***H***H′-(2), CH-(5)), 2.53 – 2.44 (m, 1H, CH***H′***-(2)), 1.73 – 1.58 (m, 5H, CH-(8), 2CH_2-(9)), 1.48 – 1.04 (m, 8H, 4CH_2-(6, 7, 10)), 1.33 (tdd, *J* = 7.1, 2.5, 1.0 Hz, 6H, 2CH_3-(13)), 0.86 (m, 2H, CH_2-(11)); ^{13}C NMR (101 MHz, $CDCl_3$) δ_c/ppm: 132.9 (C(4)), 127.2 (C(3)), 83.3 (C(1)), 63.7 (C(12)), 53.4 (C(5)), 39.9 (C(2)), 38.0 (C(9)), 35.2 (C(7)), 33.5 (C(8)), 30.1 (C(6)), 26.8 (C(11)), 26.5 (C(10)), 16.3 (C(13)); ^{31}P NMR (162 MHz, $CDCl_3$) δ_P/ppm: -1.47; HRMS (ESI) *m/z* calcd for $C_{17}H_{31}O_4NaP$ $[M+Na]^+$: 353.18522, found: 353.18493; IR (ATR) ν (cm^{-1}) thin film, $CHCl_3$: 2981.1 (w), 2922.0 (m), 2851.3 (w), 1448.1 (w), 1369.5 (m), 1263.4 (m),

1165.6 (w), 1029.3 (s), 974.7 (m), 820.6 (w), 712.0 (w); $[\alpha]^{25}_{589}$ = -73.4 (c=1.0 in $CHCl_3$, 91% ee).

Diethyl (1*R*,2*R*)-2-(2-cyclohexylethyl)cyclohex-3-en-1-yl phosphate (2h)

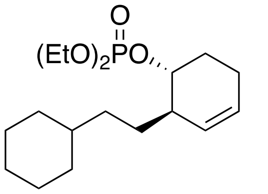

2h

Chemical Formula: $C_{18}H_{33}O_4P$
Molecular Weight: 344.4318

Synthesised according to general procedure 1b, using *meso*-5-cyclopentene-1,4-bisdiethylphosphate (**2a**) and vinylcyclohexane as the alkene partner (hydrozirconation time 30 mins). Purified by flash chromatography (15-40% EtOAc in petroleum ether) to give **2h** as a yellow oil (94 mg, 64%).

GC analysis indicated an enantiomeric excess of 95% [Astec Chiraldex ß-DA (Supelco) column; initial temperature 180 ˚C, initial hold time 5 min, progress rate 0.5 ˚C/min, final temperature 205 ˚C, final hold time 10 min; Flow rate 2 mL/min; minor enantiomer, t_R = 46.528 min; major enantiomer, t_R = 47.237 min].

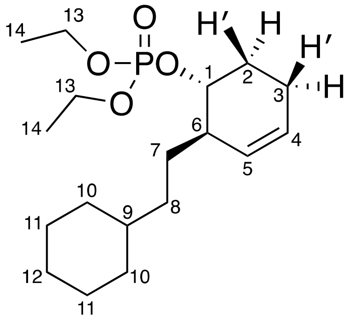

^{1}H NMR (400 MHz, $CDCl_3$) δ_H/ppm: 5.69 – 5.61 (m, 1H, CH-(4)), 5.54 – 5.47 (m, 1H, CH-(5)), 4.33 (dtd, *J* = 9.3, 6.7, 2.9 Hz, 1H, CH-(1)), 4.17 – 4.03 (m, 4H, $2CH_2$-(11)), 2.25 – 2.18 (m, 1H, CH-(6)), 2.18 – 2.12 (m, 1H, C***H***H′-(3)), 2.12 – 2.05 (m, 1H, CH***H′***-(3)), 2.05 – 1.98 (m, 1H, C***H***H′-(2)), 1.86 – 1.75 (m, 1H, CH***H′***-(2)), 1.74 – 1.50 (m, 7H, CH-(9), CHH′-(7), $2CH_2$-(10)), 1.33 (dt, *J* = 7.1, 1.0 Hz, 6H, $2CH_3$-

(14)), 1.31 – 1.09 (m, 7H, CHH´-(7), 3CH2-(8, 11)), 0.94 – 0.79 (m, 2H, CH2-(12)); ^{13}C NMR (101 MHz, CDCl3) δ_C/ppm: 128.3 (C(5)), 126.3 (C(4)), 78.4 (C(1)), 63.7 (C(13)), 42.0 (C(6)), 38.1 (C(10)), 34.0 (C(8)), 33.5 (C(9)), 30.0 (C(6)), 27.8 (C(2)), 26.8 (C(12)), 26.5 (C(11)), 23.3 (C(3)), 16.3 (C(14)); ^{31}P NMR (162 MHz, CDCl3) δ_P/ppm: -1.49; HRMS (ESI) *m/z* calcd for $C_{18}H_{33}O_4NaP$ $[M+Na]^+$: 367.20087, found: 367.20042; IR (ATR) ν (cm^{-1}) thin film, CHCl3: 2980.56 (w), 2922.3 (m), 2850.9 (w), 1447.8 (w), 1393.3 (w), 1263.2 (m), 1165.7 (w), 1034.3 (s), 1011.9 (s), 817.8 (w), 725.6 (w); $[\alpha]^{25}_{589}$ = -66.2 (c=1.0 in CHCl3, 96% ee).

(1*R*,2*R*)-2-(6-chlorohexyl)cyclopent-3-en-1-yl diethyl phosphate (1i)

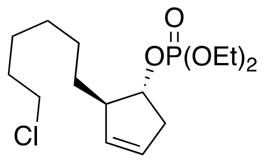

1i

Chemical Formula: $C_{15}H_{28}ClO_4P$
Molecular Weight: 338.8088

Synthesised according to general procedure 1b, using *meso*-4-cyclopentene-1,3-bisdiethylphosphate (**1a**) and 6-chloro-1-hexene as the alkene partner (hydrozirconation time 20 mins). Purified by flash chromatography (20-40% EtOAc in petroleum ether) to give **1i** as a yellow oil (61 mg, 45%).

The product was derivatised according to general procedure 2 for HPLC analysis.

HPLC analysis indicated an enantiomeric excess of 91% [Chiralpak® IC; flow: 0.8 mL/min; hexane/iPrOH: 98:2; λ = 210 nm; minor enantiomer, t_R = 17.579 min ; major enantiomer, t_R = 18.652 min].

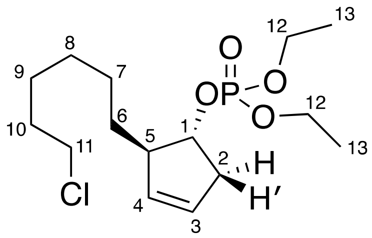

^{1}H NMR (400 MHz, $CDCl_3$) δ_H/ppm: 5.70 – 5.66 (m, 1H, CH-(4)), 5.66 – 5.62 (m, 1H, CH-(3)), 4.67 (tt, *J* = 6.5, 3.0 Hz, 1H, CH-(1)), 4.16 – 4.03 (m, 4H, $2CH_2$-(12)), 3.52 (t, *J* = 6.7 Hz, 2H, CH_2-(11)), 2.81 – 2.71 (m, 2H, C***H***H'-(2) CH-(5)), 2.54 – 2.45 (m, 1H, CH***H'***-(2)), 1.81 – 1.71 (m, 2H, CH_2-(10)), 1.50 – 1.25 (m, 8H, $4CH_2$-(6, 7, 8, 9)), 1.33 (tdd, *J* = 7.1, 1.8, 1.0 Hz, 6H, $2CH_3$-(13)); ^{13}C NMR (101 MHz, $CDCl_3$) δ_c/ppm: 132.8 (C(4)), 127.3 (C(3)), 83.2 (C(1)), 63.7 (C(12)), 53.1 (C(5)), 45.2 (C(11)), 39.9 (C(2)), 32.72 (C(10)) 32.70 (C(6)), 29.1 (C(7-9)), 27.4 (C(7-9)), 26.9 (C(7-9)), 16.3 (C(13)); ^{31}P NMR (162 MHz, $CDCl_3$) δ_P/ppm: -1.46; HRMS (ESI) *m/z* calcd for $C_{15}H_{28}O_4{}^{35}ClNaP$ $[M+Na]^+$: 361.13059, found: 361.13063; IR (ATR) ν (cm^{-1}) thin film, $CHCl_3$: 2930.2 (w), 2856.1 (w), 1443.8 (w), 1263.8 (m), 1166.9 (w), 1029.2 (s), 821.2 (w), 714.1 (w); $[\alpha]^{25}_{589}$ = -63.1 (c=1.0 in $CHCl_3$, 91% ee).

(1*R*,2*R*)-2-(6-chlorohexyl)cyclohex-3-en-1-yl diethyl phosphate (2i)

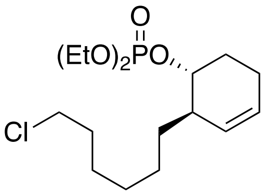

2i

Chemical Formula: $C_{16}H_{30}ClO_4P$
Molecular Weight: 352.8358

Synthesised according to general procedure 1b, using *meso*-5-cyclohexene-1,4-bisdiethylphosphate (**2a**) and 6-chloro-1-hexene as the alkene partner (hydrozirconation time 20 mins). Purified by flash chromatography (20-40% EtOAc in petroleum ether) to give **2i** as a yellow oil (95 mg, 67%).

The product was derivatised according to general procedure 2 for HPLC analysis.

HPLC analysis indicated an enantiomeric excess of 95% [Chiralpak® IC; flow: 0.8 mL/min; hexane/iPrOH: 98:2; λ = 210 nm; major enantiomer, t_R = 25.573 min ; minor enantiomer, t_R = 27.726 min].

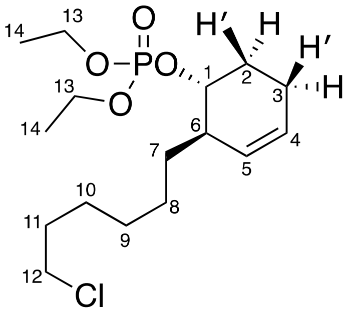

^{1}H NMR (400 MHz, $CDCl_3$) δ_H/ppm: 5.69 – 5.62 (m, 1H, CH-(4)), 5.55 – 5.47 (m, 1H, CH-(5)), 4.32 (dtd, *J* = 9.4, 6.7, 2.9 Hz, 1H, CH-(1)), 4.10 (pd, *J* = 7.1, 3.2 Hz, 4H, $2CH_2$-(13)), 3.52 (t, J = 6.7 Hz, 2H, CH_2-(12)), 2.29 – 2.21 (m, 1H, CH-(6)), 2.21 – 2.12 (m, 1H, C***H***H´-(3)), 2.12 – 2.05 (m, 1H, CH***H´***-(3)), 2.05 – 1.98 (m, 1H, C***H***H´-(2)), 1.86 – 1.75 (m, 1H, C***H***H´-(2)), 1.79 – 1.71 (m, 2H, CH_2-(11)), 1.59 – 1.25 (m, 8H, $4CH_2$-(7, 8, 9, 10)) 1.33 (td, *J* = 7.1, 1.1 Hz, 6H, $2CH_3$-(14)); ^{13}C NMR (101 MHz, $CDCl_3$) δ_C/ppm: 128.1 (C(5)), 126.4 (C(4)), 78.3 (C(1)), 63.7 (C(13)), 45.2 (C(12)), 41.7 (C(6)), 32.7 (C(11)), 32.6 (C(7)), 29.2 (C(8-10)), 27.8 (C(2)), 26.9 (C(8-10)), 26.2 (C(8-10)), 23.4 (C(3)), 16.3 (C(14)); ^{31}P NMR (162 MHz, $CDCl_3$) δ_P/ppm: -1.46; HRMS (ESI) *m/z* calcd for $C_{16}H_{30}O_4{}^{35}ClNaP$ $[M+Na]^+$: 375.14636, found: 35.14624; IR (ATR) ν (cm^{-1}) thin film, $CHCl_3$: 2930.6 (w), 2856.2 (w), 1444.0 (w), 1274.0 (w), 1167.1 (w), 1030.0 (s), 821.1 (w), 713.8 (w); $[\alpha]^{25}_{589}$ = -61.3 (c=1.0 in $CHCl_3$, 95% ee).

(1*R*,2*R*)-2-(4-((*tert*-butyldiphenylsilyl)oxy)butyl)cyclopent-3-en-1-yl diethyl phosphate (1j)

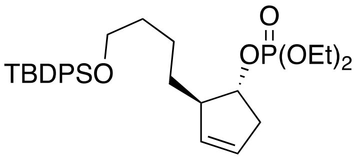

1j

Chemical Formula: $C_{29}H_{43}O_5PSi$
Molecular Weight: 530.7168

Synthesised according to general procedure 1b, using *meso*-4-cyclopentene-1,3-bisdiethylphosphate (**1a**) and (but-3-en-1-yloxy)(*tert*-butyl)diphenylsilane as the alkene partner (hydrozirconation time 20 mins). Purified by flash chromatography (15-40% EtOAc in petroleum ether) to give **1j** as a yellow oil (109 mg, 51%).

HPLC analysis indicated an enantiomeric excess of 89% [Chiralpak® ID; flow: 1.0 mL/min; hexane/iPrOH: 97:3; λ = 210 nm; major enantiomer, t_R = 21.054 min; minor enantiomer, t_R = 24.276 min].

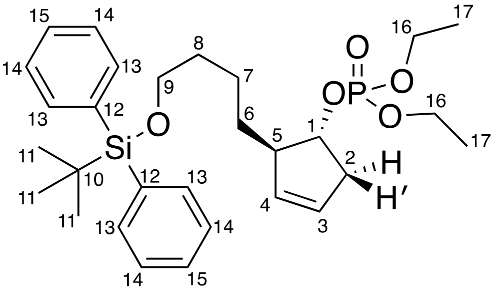

^{1}H NMR (400 MHz, $CDCl_3$) δ_H/ppm: 7.70 – 7.63 (m, 4H, ArH-(14)), 7.46 – 7.34 (m, 6H, ArH-(13, 15)), 5.71 – 5.61 (m, 2H, 2CH-(3, 4)), 4.65 (tt, *J* = 6.4, 2.9 Hz, 1H, CH-(1)), 4.09 (p, *J* = 7.1 Hz, 4H, $2CH_2$-(16)), 3.65 (t, *J* = 6.5 Hz, 2H, CH_2-(9)), 2.80 – 2.70 (m, 2H, C***H***H´-(2), CH-(5)), 2.54 – 2.46 (m, 1H, CH***H*´**-(2)), 1.64 – 1.51 (m, 2H, CH_2-(6)), 1.48 – 1.26 (m, 4H, $2CH_2$-(7, 8)), 1.32 (tdd, *J* = 7.1, 2.8, 1.0 Hz, 6H, $2CH_3$-(17)), 1.04 (s, 9H, $3CH_3$-(11)); ^{13}C NMR (101 MHz, $CDCl_3$) δ_C/ppm: 135.7 (C(12)), 134.2 (C(15)), 132.7 (C(4)), 128.7 (C(13)), 127.7 (C(14)), 127.3 (C(3)), 83.2 (C(1)), 63.9 (C(9)), 63.7 (C(16)), 53.1 (C(5)), 39.9 (C(2)), 32.8 (C(8)), 32.7 (C(6)), 27.0 (C(11)), 23.8 (C(7)), 19.4 (C(10)), 16.3 (C(17)); ^{31}P NMR (162 MHz, $CDCl_3$) δ_P/ppm: -1.47; HRMS (ESI) *m/z* calcd for $C_{29}H_{43}O_5NaPSi$ $[M+Na]^+$: 553.25096, found: 553.25022; IR (ATR) ν (cm^{-1}) thin film, $CHCl_3$: 3069.8 (w), 2931.9 (w), 2857.9 (w), 2361.5 (w), 1473.0 (w), 1428.4 (w), 1391.0 (w), 1361.8 (w), 1263.5 (w), 1166.1 (w), 1110.4 (m), 1031.7 (s), 977.2 (m), 822.8 (w), 741.3 (w), 703.6 (m), 613.9 (w); $[\alpha]^{25}_{589}$ = -46.2 (c=1.0 in $CHCl_3$, 89% ee).

(1*R*,2*R*)-2-(4-((*tert*-butyldiphenylsilyl)oxy)butyl)cyclohex-3-en-1-yl diethyl phosphate (2j)

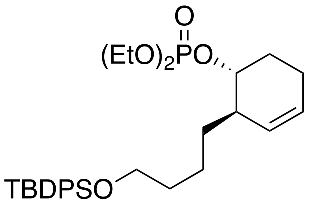

2j

Chemical Formula: $C_{30}H_{45}O_5PSi$
Molecular Weight: 544.7438

Synthesised according to general procedure 1b, using *meso*-5-cyclohexene-1,4-bisdiethylphosphate (**2a**) and (but-3-en-1-yloxy)(*tert*-butyl)diphenylsilane as the alkene partner (hydrozirconation time 20 mins). Purified by flash chromatography (15-40% EtOAc in petroleum ether) to give **2j** as a yellow oil (139 mg, 64%).

HPLC analysis indicated an enantiomeric excess of 92% [Chiralpak® IC; flow: 0.8 mL/min; hexane/iPrOH: 96:4; λ = 210 nm; major enantiomer, t_R = 18.135 min; minor enantiomer, t_R = 19.126 min].

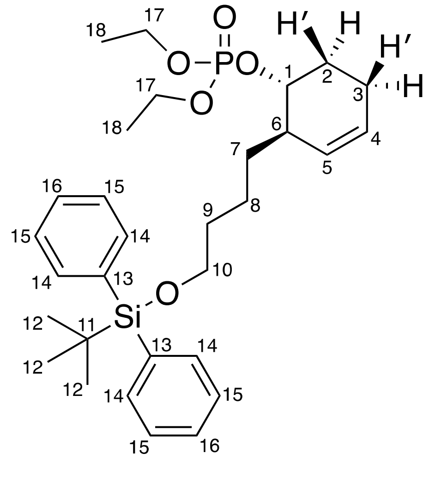

^{1}H NMR (400 MHz, $CDCl_3$) δ_H/ppm: 7.69 – 7.64 (m, 4H, ArH-(15)), 7.45 – 7.34 (m, 6H, ArH-(14, 16)), 5.69 – 5.63 (m, 1H, CH-(4)), 5.55 – 5.49 (m, 1H, CH-(5)), 4.32 (dtd, *J* = 9.1, 6.6, 2.8 Hz, 1H, CH-(1)), 4.09 (pd, *J* = 7.2, 2.2 Hz, 4H, 2CH_2-(17)), 3.40 (t, *J* = 6.8 Hz, 2H, CH_2-(10)), 2.28 – 2.20 (m, 1H, CH-(6)), 2.20 – 2.13 (m, 1H, C***H***H´-(3)), 2.13 – 2.05 (m, 1H, CH***H***´-(3)), 2.05 – 1.98 (m, 1H, C***H***H´-(2)), 1.87 – 1.76 (m, 1H, CH***H***´-(2)), 1.65 – 1.24 (m, 6H, 3CH_2-(7, 8, 9)), 1.31 (qd, *J* = 7.1, 1.0

Hz, 6H, 2CH3-(18)), 1.04 (s, 9H, 3CH3-(12)); ^{13}C NMR (101 MHz, CDCl3) δ_C/ppm: 135.7 (C(13)), 134.2 (C(16)), 129.6 (C(14)), 128.0 (C(5)), 127.7 (C(15)), 126.4 (C(4)), 78.4 (C(1)), 63.9 (C(10)), 63.7 (C(17)), 41.8 (C(6)), 32.9 (C(9)), 32.5 (C(7)), 27.7 (C(2)), 27.0 (C(12)), 23.3 (C(3)), 22.7 (C(8)), 19.4 (C(11)), 16.3 (C(18)); ^{31}P NMR (162 MHz, CDCl3) δ_P/ppm: -1.49; HRMS (ESI) *m/z* calcd for $C_{30}H_{45}O_5NaPSi$ $[M+Na]^+$: 567.26661, found: 567.26490; IR (ATR) ν (cm^{-1}) thin film, CHCl3: 3069.8 (w), 2931.9 (w), 2857.9 (w), 2361.5 (w), 1473.0 (w), 1428.4 (w), 1391.0 (w), 1361.8 (w), 1263.5 (w), 1166.1 (w), 1110.4 (m), 1031.7 (s), 977.2 (m), 822.8 (w), 741.3 (w), 703.6 (m), 613.9 (w); $[\alpha]^{25}_{589}$ = -43.3 (c=1.0 in CHCl3, 95% ee).

(1*R*,2*R*)-2-(4-((*tert*-butyldiphenylsilyl)oxy)butyl)cyclohept-3-en-1-yl diethyl phosphate (3j)

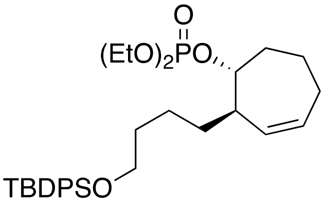

3j

Chemical Formula: $C_{31}H_{47}O_5PSi$
Molecular Weight: 558.7708

Synthesised according to general procedure 1b, using *meso*-6-cycloheptene-1,5-bisdiethylphosphate (**3a**) and (but-3-en-1-yloxy)(*tert*-butyl)diphenylsilane as the alkene partner (hydrozirconation time 20 mins). Purified by flash chromatography (20-40% EtOAc in petroleum ether) to give **3j** as a yellow oil (134 mg, 60%).

HPLC analysis indicated an enantiomeric excess of 91% [Chiralpak® IC; flow: 1.0 mL/min; hexane/iPrOH: 96:4; λ = 210 nm; major enantiomer, t_R = 15.451 min; minor enantiomer, t_R = 16.874 min].

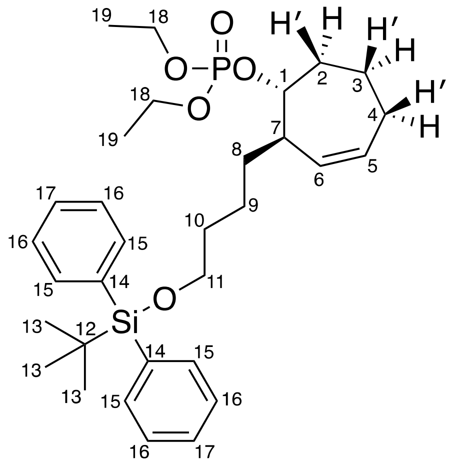

^{1}H NMR (400 MHz, $CDCl_3$) δ_H/ppm: 7.69 – 7.63 (m, 4H, ArH-(16)), 7.45 – 7.33 (m, 3H, ArH-(15, 17)), 5.84 (dtd, J = 11.2, 6.2, 1.5 Hz, 1H, CH-(5)), 5.41 (dd, J = 11.2, 5.3 Hz, 1H, CH-(6)), 4.25 (qd, J = 7.9, 3.1 Hz, 1H, CH-(1)), 4.08 (p, J = 7.1 Hz, 4H, $2CH_2$-(18)), 3.65 (t, J = 6.4 Hz, 2H, CH_2-(11)), 2.58 – 2.48 (m, 1H, CH-(7)), 2.24 – 2.16 (m, 1H, C***H***H´-(2)), 2.09 (q, J = 5.9 Hz, 2H, C***HH´***-(4)), 1.99 – 1.88 (m, 1H, CH***H´***-(2)), 1.79 – 1.69 (m, 1H, C***H***H´-(3)), 1.64 – 1.21 (m, 6H, $3CH_2$-(8, 9, 10)), 1.31 (qd, J = 7.1, 1.0 Hz, 6H, $2CH_3$-(19)), 1.04 (s, 9H, $3CH_3$-(13)); ^{13}C NMR (101 MHz, $CDCl_3$) δ_C/ppm: 135.7 (C(14)), 134.2 (C(17)), 133.0 (C(5)), 131.4 (C(6)), 129.6 (C(15)), 127.7 (C(16)), 79.5 (C(1)), 64.0 (C(11)), 63.6 (C(18)), 44.6 (C(7)), 36.3 (C(2)), 32.9 (C(10)), 31.6 (C(8)), 28.1 (C(4)), 27.00 (C(13)), 23.2 (C(9)), 23.0 (C(3)), 19.4 (C(12)), 16.3 (C(19)); ^{31}P NMR (162 MHz, $CDCl_3$) δ_P/ppm: -1.76; HRMS (ESI) *m/z* calcd for $C_{31}H_{47}O_5NaPSi$ $[M+Na]^+$: 581.28226, found: 581.28170; IR (ATR) ν (cm^{-1}) thin film, $CHCl_3$: 2931.3 (w), 2858.4 (w), 1472.9 (w), 1428.3 (w), 1390.6 (w), 1261.5 (m), 1166.0 (w), 1110.1 (m), 1033.5 (s), 998.1 (s), 822.4 (w), 741.6 (w), 703.1 (m), 613.6 (w); $[\alpha]^{25}_{589}$ = -20.7 (c=1.0 in $CHCl_3$, 91% ee).

Diethyl ((1*R*,2*R*)-2-(4-methoxyphenethyl)cyclopent-3-en-1-yl) phosphate (1k)

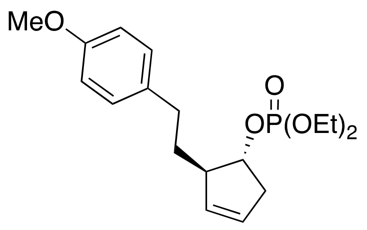

1k

Chemical Formula: $C_{18}H_{27}O_5P$
Molecular Weight: 354.3828

Synthesised according to general procedure 1b, using *meso*-4-cyclopentene-1,3-bisdiethylphosphate (**1a**) and 4-methoxystyrene as the alkene partner (hydrozirconation time 20 mins). Purified by flash chromatography (25-50% EtOAc in petroleum ether) to give **1k** as a yellow oil (99 mg, 70%). **NOTE**: Compound unstable in air and should be stored under an Ar atmosphere in a freezer.

HPLC analysis indicated an enantiomeric excess of 91% [Chiralpak® ID; flow: 1.0 mL/min; hexane/iPrOH: 90:10; λ = 210 nm; major enantiomer, t_R = 22.170 min; minor enantiomer, t_R = 24.908 min].

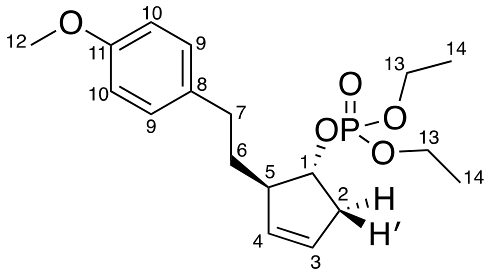

^{1}H NMR (400 MHz, $CDCl_3$) δ_H/ppm: 7.10 (d, *J* = 8.7 Hz, 2H, ArH-(10)), 6.81 (d, *J* = 8.7 Hz, 2H, ArH-(9)), 5.74 – 5.69 (m, 1H, CH-(4)), 5.69 – 5.65 (m, 1H, CH-(3)), 4.74 (tt, *J* = 6.4, 3.0 Hz, 1H, CH-(1)), 4.09 (pd, *J* = 7.4, 3.2 Hz, 4H, $2CH_2$-(13)), 3.78 (s, 3H, CH_3-(12)), 2.85 – 2.74 (m, 2H, C***H***H´-(2), CH-(5)), 2.63 (t, *J* = 7.4 Hz, 2H, CH_2-(7)), 2.56 – 2.47 (m, 1H, CH***H´***-(2)), 1.78 – 1.54 (m, 2H, CHH´-(6)), 1.32 (tdd, *J* = 7.1, 2.6, 1.0 Hz, 6H, $2CH_3$-(14)); ^{13}C NMR (101 MHz, $CDCl_3$) δ_C/ppm: 157.9 (C(11)), 134.2 (C(9)), 132.5 (C(4)), 129.4 (C(10)), 127.6 (C(3)), 113.9 (C(8)), 83.1 (C(1)), 63.7 (C(13)), 55.4 (C(12)), 52.6 (C(5)), 39.9 (C(2)), 34.9 (C(7)), 32.9 (C(6)), 16.3

(C(14)); ^{31}P NMR (162 MHz, $CDCl_3$) δ_P/ppm: -1.44; HRMS (ESI) *m/z* calcd for $C_{18}H_{27}O_5NaP$ $[M+Na]^+$: 377.14883, found: 377.14877; IR (ATR) ν (cm^{-1}) thin film, $CHCl_3$: 3058.8 (w), 2931.8 (w), 2853.3 (w), 2360.3 (w), 1612.0 (w), 1512.7 (m), 1456.3 (w), 1246.7 (m), 1178.1 (w), 1031.4 (s), 977.9 (m), 821.2 (w), 752.5 (w); $[\alpha]^{25}_{589}$ = -62.8 (c=1.0 in $CHCl_3$, 91% ee).

Diethyl ((1*R*,2*R*)-2-(4-methoxyphenethyl)cyclohex-3-en-1-yl) phosphate (2k)

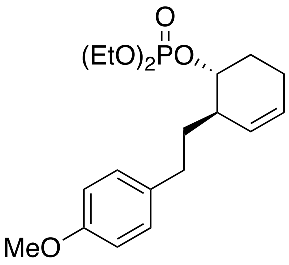

2k

Chemical Formula: $C_{19}H_{29}O_5P$
Molecular Weight: 368.4098

Synthesised according to general procedure 1b, using *meso*-5-cyclohexene-1,4-bisdiethylphosphate (**2a**) and 4-methoxystyrene as the alkene partner (hydrozirconation time 20 mins). Purified by flash chromatography (25-50% EtOAc in petroleum ether) to give **2k** as a yellow oil (103 mg, 70%). **NOTE**: Compound unstable in air and should be stored under an Ar atmosphere in a freezer.

SFC analysis indicated an enantiomeric excess of 99% [Chiralpak® IG; flow: 1.5 mL/min; $MeOH/CO_2$: 1% to 30%; λ = 210 nm; major enantiomer, t_R = 3.393 min; minor enantiomer, t_R = 3.462 min].

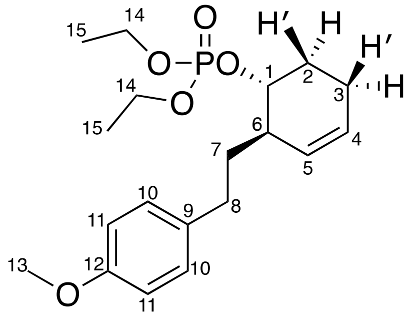

^{1}H NMR (400 MHz, $CDCl_3$) δ_H/ppm: 7.11 (d, J = 8.6 Hz, 2H, ArH-(11)), 6.81 (d, J = 8.6 Hz, 2H, ArH-(10)), 5.73 – 5.67 (m, 1H, CH-(4)), 5.57 (dd, J = 10.0, 1.8 Hz, 1H,

CH-(5)), 4.40 (dtd, *J* = 9.3, 6.7, 2.9 Hz, 1H, CH-(1)), 4.08 (pd, *J* = 7.2, 2.2 Hz, 4H, $2CH_2$-(14)), 3.77 (s, 3H, CH_2-(13)), 2.70 (ddd, *J* = 13.8, 10.6, 5.5 Hz, 1H, CHH´-(8)), 2.55 (ddd, *J* = 13.8, 10.4, 6.1 Hz, 1H, CHH´-(8)), 2.36 – 2.27 (m, 1H, CH-(6)), 2.27 – 2.16 (m, 1H, C***H***H´-(3)), 2.16 – 2.07 (m, 1H, CH***H´***-(3)), 2.07 – 2.01 (m, 1H, C***H***H´-(2)), 1.89 – 1.77 (m, 2H, CH***H´***-(2) CHH´-(7)), 1.68 – 1.57 (m, 1H, CHH´-(7)), 1.31 (qd, *J* = 7.1, 1.0 Hz, 6H, $2CH_3$-(15)); ^{13}C NMR (101 MHz, $CDCl_3$) δ_C/ppm: 157.8 (C(12)), 134.4 (C(10)), 129.3 (C(11)), 127.8 (C(5)), 126.8 (C(4)) 113.9 (C(9)), 78.4 (C(1)), 63.7 (C(14)), 55.4 (C(13)), 41.3 (C(6)), 34.8 (C(8)), 31.6 (C(7)), 27.8 (C(2)), 23.4 (C(3)), 16.3 (C(18)); ^{31}P NMR (162 MHz, $CDCl_3$) δ_P/ppm: -1.44; HRMS (ESI) *m/z* calcd for $C_{19}H_{29}O_5NaP$ $[M+Na]^+$: 391.16448, found: 391.16517; IR (ATR) ν (cm^{-1}) thin film, $CHCl_3$: 3649.5 (w), 2980.7 (m), 2360.3 (w), 1612.1 (w), 1512.6 (m), 1457.5 (w), 1392.8 (w), 1247.5 (m), 1176.5 (w), 1032.5 (s), 972.5 (m), 818.1 (w), 726.4 (w); $[\alpha]^{25}_{589}$ = -55.9 (c=1.0 in $CHCl_3$, 97% ee).

Diethyl ((1*R*,2*R*)-2-(4-methoxyphenethyl)cyclohept-3-en-1-yl) phosphate (3k)

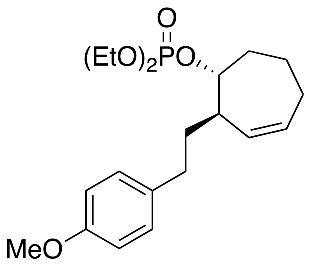

3k

Chemical Formula: $C_{20}H_{31}O_5P$
Molecular Weight: 382.4368

Synthesised according to general procedure 1b, using *meso*-6-cycloheptene-1,5-bisdiethylphosphate (**3a**) and 4-methoxystyrene as the alkene partner (hydrozirconation time 20 mins). Purified by flash chromatography (25-50% EtOAc in petroleum ether) to give **3k** as a yellow oil (80 mg, 52%). **NOTE**: Compound unstable in air and should be stored under an Ar atmosphere in a freezer.

HPLC analysis indicated an enantiomeric excess of 94% [Chiralpak® IA; flow: 1.0 mL/min; hexane/iPrOH: 93:7; λ = 210 nm; major enantiomer, t_R = 11.108 min; minor enantiomer, t_R = 11.960 min].

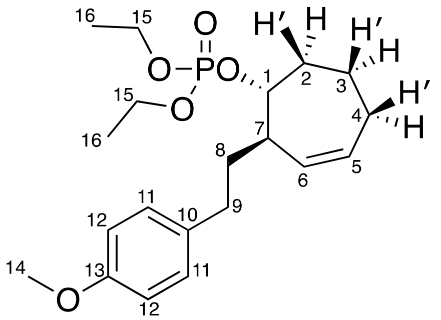

^{1}H NMR (400 MHz, $CDCl_3$) δ_H/ppm: 7.10 (d, *J* = 6.1 Hz, 2H, ArH-(12)), 6.81 (d, *J* = 8.7 Hz, 2H, ArH-(11)), 5.91 (dtd, *J* = 11.2, 6.23, 1.5 Hz, 1H, CH-(5)), 5.52 (dd, *J* = 11.2, 5.3 Hz, 1H, CH-(6)), 4.31 (qd, *J* = 7.9, 3.0 Hz, 1H, CH-(1)), 4.11 – 4.02 (m, 4H, $2CH_2$-(15)), 3.78 (s, 3H, CH_3-(14)), 2.71 (ddd, *J* = 13.8, 10.7, 4.9 Hz, 1H, CHH´-(9)), 2.63 – 2.55 (m, 1H, CH-(7)), 2.50 (ddd, *J* = 13.8, 10.4, 6.4 Hz, 1H, CHH´-(9)), 2.24 – 2.17 (m, 1H, C***H***H´-(2)), 2.11 (q, *J* = 5.6 Hz, 2H, C***HH´***-(4)), 1.98 – 1.84 (m, 2H, CH***H´***-(2), CHH´-(8)), 1.78 – 1.66 (m, 2H, C***H***H´-(3), CHH´-(8)), 1.55 – 1.42 (m, 1H, CH***H´***-(3)), 1.30 (dtd, *J* = 10.9, 7.1, 1.0 Hz, 6H, $2CH_3$-(16)); ^{13}C NMR (101 MHz, $CDCl_3$) δ_c/ppm: 157.8 (C(13)), 134.5 (C(12)), 133.4 (C(5)), 131.1 (C(6)), 129.4 (C(11)), 113.8 (C(10)), 79.2 (C(1)), 63.6 (C(15)), 55.4 (C(14)), 44.0 (C(7)), 36.4 (C(2)), 34.0 (C(9)), 32.2 (C(8)), 28.1 (C(4)), 23.0 (C(3)), 16.3 (C(16)); ^{31}P NMR (162 MHz, $CDCl_3$) δ_P/ppm: -1.73; HRMS (ESI) *m/z* calcd for $C_{20}H_{31}O_5NaP$ $[M+Na]^+$: 405.18013, found: 405.17959; IR (ATR) ν (cm^{-1}) thin film, $CHCl_3$: 2932.2 (w), 1612.0 (w), 1512.6 (m), 1444.4 (w), 1392.8 (w), 1246.9 (m), 1177.9 (w), 1032.7 (s), 998.9 (s), 819.5 (w), 700.5 (w); $[\alpha]^{25}_{589}$ = -34.3 (c=1.0 in $CHCl_3$, 94% ee).

(1*R*,2*R*)-2-(2-bromophenethyl)cyclopent-3-en-1-yl diethyl phosphate (1l)

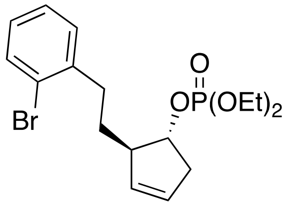

1l

Chemical Formula: $C_{17}H_{24}BrO_4P$
Molecular Weight: 403.2528

Synthesised according to general procedure 1b, using *meso*-4-cyclopentene-1,3-bisdiethylphosphate (**1a**) and 2-bromostyrene as the alkene partner (hydrozirconation

time 20 mins). Purified by flash chromatography (25-50% EtOAc in petroleum ether) to give **1l** as a yellow oil (85 mg, 53%).

HPLC analysis indicated an enantiomeric excess of 83% [Chiralpak® ID; flow: 1.0 mL/min; hexane/iPrOH: 90:10; λ = 210 nm; major enantiomer, t_R = 17.193 min; minor enantiomer, t_R = 18.143 min].

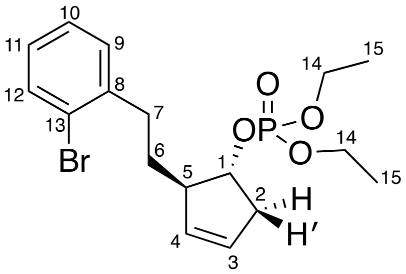

^{1}H NMR (400 MHz, $CDCl_3$) δ_H/ppm: 7.56 – 7.50 (m, 1H, ArH-(12)), 7.28 – 7.21 (m, 2H, ArH-(9, 10)), 7.10 – 7.05 (m, 1H, ArH-(11)), 5.78 (d, *J* = 6.0, 1.9 Hz, 1H, CH-(4)), 5.73 (d, *J* = 6.0, 2.0 Hz, 1H, CH-(3)), 4.82 (tt, *J* = 6.5, 3.1 Hz, 1H, CH-(1)), 4.09 (pd, *J* = 7.1, 3.2 Hz, 4H, $2CH_2$-(14)), 2.94 – 2.74 (m, 4H, C***H***H´-(2), CH-(5), CH_2-(7)), 2.57 (dd, *J* = 17.5, 2.0 Hz, 1H, CH***H´***-(2)), 1.82 – 1.59 (m, 2H, CHH´-(6)), 1.36 (tt, *J* = 7.1, 0.8 Hz, 6H, $2CH_3$-(15)); ^{13}C NMR (101 MHz, $CDCl_3$) δ_C/ppm: 141.4 (C(8)), 132.9 (C(4)), 132.3 (C(12)), 130.5 (C(10)), 127.9 (C(3)), 127.8 (C(9)), 127.6 (C(11)), 124.4 (C(13)), 82.9 (C(1)), 63.8 (C(14)), 52.9 (C(5)), 40.0 (C(2)), 34.2 (C(7)), 33.0 (C(6)), 16.3 (C(15)); ^{31}P NMR (162 MHz, $CDCl_3$) δ_P/ppm: -1.48; HRMS (ESI) *m/z* calcd for $C_{17}H_{24}O_4{}^{79}BrNaP$ $[M+Na]^+$: 425.04878, found: 425.04845; IR (ATR) ν (cm^{-1}) thin film, $CHCl_3$: 3649.2 (w), 3059.2 (w), 2980.7 (m), 2362.7 (w), 1471.8 (w), 1440.7 (w), 1392.4 (w), 1263.4 (m), 1164.2 (w), 1027.2 (s), 970.6 (m), 821.2 (w), 753.1 (w), 658.2 (w); $[\alpha]^{25}_{589}$ = -66.9 (c=1.0 in $CHCl_3$, 83% ee).

(1*R*,2*R*)-2-(2-bromophenethyl)cyclohex-3-en-1-yl diethyl phosphate (2l)

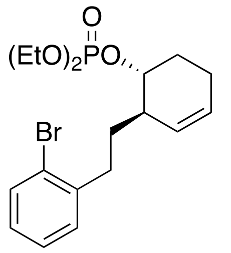

2l

Chemical Formula: $C_{18}H_{26}BrO_4P$
Molecular Weight: 417.2798

Synthesised according to general procedure 1b, using *meso*5-cyclohexene-1,4-bisdiethylphosphate (**2a**) and 2-bromostyrene as the alkene partner (hydrozirconation time 20 mins). Purified by flash chromatography (25-50% EtOAc in petroleum ether) to give **2l** as a yellow oil (102 mg, 61%).

HPLC analysis indicated an enantiomeric excess of 91% [Chiralpak® IA; flow: 0.7 mL/min; hexane/iPrOH: 99:1; λ = 210 nm; major enantiomer, t_R = 45.337 min; minor enantiomer, t_R = 54.833 min].

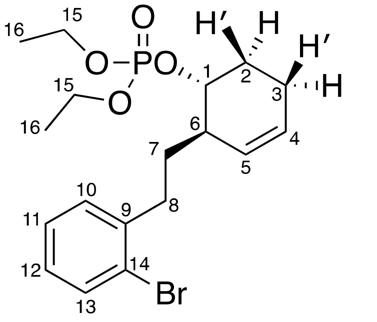

^{1}H NMR (400 MHz, $CDCl_3$) δ_H/ppm: 7.50 (dd, *J* = 8.0, 1.2 Hz, 1H, ArH-(13)), 7.28 – 7.18 (m, 2H, ArH-(10, 11)), 7.08 – 7.00 (m, 1H, ArH-(12)), 5.77 – 5.67 (m, 1H, CH-(4)), 5.67 – 5.62 (m, 1H, CH-(5)), 4.42 (dtd, *J* = 9.7, 7.0, 3.0 Hz, 1H, CH-(1)), 4.15 – 4.05 (m, 4H, $2CH_2$-(15)), 2.85 (ddd, *J* = 13.6, 11.2, 5.4 Hz, 1H, CHH´-(8)), 2.75 (ddd, *J* = 13.6, 10.9, 5.6 Hz, 1H, CHH´-(8)), 2.41 – 2.31 (m, 1H, CH-(6)), 2.26 – 2.16 (m, 1H, C***H***H´-(3)), 2.16 – 2.09 (m, 1H, CH***H´***-(3)), 2.09 – 2.03 (m, 1H, C***H***H´-(2)), 1.96 – 1.78 (m, 2H, CH***H´***-(2) CHH´-(7)), 1.69 – 1.55 (m, 1H, CHH´-(7)), 1.31 (qd, *J* =

7.0, 1.0 Hz, 6H, 2CH_3-(16)); ^{13}C NMR (101 MHz, $CDCl_3$) δ_c/ppm: 141.7 (C(9)), 132.9 (C(13)), 130.5 (C(11)), 127.7 (C(5)), 127.6 (C(10)), 127.6 (C(12)), 126.9 (C(4)) 124.5 (C(14)), 78.0 (C(1)), 63.7 (C(15)), 41.7 (C(6)), 33.0 (C(8)), 32.8 (C(7)), 27.8 (C(2)), 23.5 (C(3)), 16.3 (C(16)); ^{31}P NMR (162 MHz, $CDCl_3$) δ_P/ppm: -1.49; HRMS (ESI) *m/z* calcd for $C_{18}H_{26}O_4{}^{79}BrNaP$ $[M+Na]^+$: 439.06443, found: 439.06403; IR (ATR) ν (cm^{-1}) thin film, $CHCl_3$: 2983.7 (w), 2932.2 (w), 2360.5 (w), 1472.0 (w), 1439.0 (w), 1262.8 (m), 1165.5 (w), 1023.1 (s), 979.7 (m), 818.1 (w), 752.3 (w); $[\alpha]^{25}_{589}$ = -44.4 (c=1.0 in $CHCl_3$, 91% ee).

(1*S*,2*R*,5*R*)-2-benzyl-2-methyl-5-(4-phenylbutyl)cyclopent-3-en-1-yl diethyl phosphate (4b)

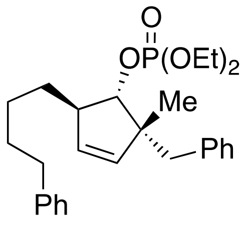

Chemical Formula: $C_{27}H_{37}O_4P$
Molecular Weight: 456.5628

Synthesised according to general procedure 1c, using (1*R*,2*S*,3*S*)-2-benzyl-2-methylcyclopent-4-ene-1,3-bis(diethyl phosphate) (**4a**) and 4-phenyl-1-butene as the alkene partner (hydrozirconation time 20 mins). Purified by flash chromatography (10-20-30% EtOAc in petroleum ether) to give **4b** as a yellow oil (127 mg, 70%).

HPLC analysis indicated an enantiomeric excess of 94% [Chiralpak® IC; flow: 1.0 mL/min; hexane/iPrOH: 95:5; λ = 210 nm; major enantiomer, t_R = 14.330 min; minor enantiomer, t_R = 15.586 min].

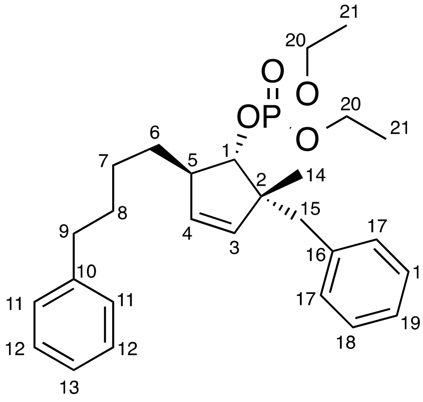

^{1}H NMR (400 MHz, $CDCl_3$) δ_H/ppm: 7.31 – 7.22 (m, 4H, 4ArH-(12, 17), 7.22 – 7.12 (m, 6H, 6ArH-(11, 13, 18, 19), 5.57 (d, *J* = 6.3 Hz, 1H, CH-(3)), 5.32 (dd, *J* = 6.3, 2.3 Hz, 1H, CH-(4)), 4.33 (dd, *J* = 8.4, 7.4 Hz, 1H, CH-(1)), 4.23 – 4.11 (m, 4H, $2CH_2$-(20)), 2.85 (d, *J* = 13.0 Hz, 1H, CHH′-(15)), 2.79 – 2.72 (m, 1H, CH-(5)), 2.66 – 2.60 (m, 2H, CH_2-(9)), 2.65 (d, *J* = 13.0 Hz, 1H, CHH′-(15)), 1.85 – 1.74 (m, 1H, CHH′-(6)), 1.71 – 1.61 (m, 2H, CH_2-(8)), 1.56 – 1.46 (m, 1H, CHH′-(6)), 1.43 – 1.33 (m, 2H, CH_2-(7)), 1.39 (td, *J* = 7.1, 1.1 Hz, 3H, CH_3-(21)), 1.36 (td, *J* = 7.1, 1.1 Hz, 3H,

CH_3-(21)), 1.13 (s, 3H, CH_3-(14)); ^{13}C NMR (101 MHz, $CDCl_3$) δ_C/ppm: 142.8 (C(10)), 138.8 (C(16)), 137.5 (C(4)), 130.9 (C(17)), 129.8 (C(3)), 128.5 (C(12)), 128.4 (C(11)), 127.7 (C(18)), 126.0 (C(19)), 125.8 (C(13)), 91.2 (C(1)), 63.9 (C(20)), 50.6 (C(2)), 50.1 (C(5)), 41.0 (C(15)), 36.0 (C(9)), 32.2 (C(6)), 31.8 (C(8)), 27.3 (C(7)), 24.5 (C(14)), 16.4 (C(21)); ^{31}P NMR (162 MHz, $CDCl_3$) δ_P/ppm: -1.15; HRMS (ESI) *m/z* calcd for $C_{27}H_{37}O_4NaP$ $[M+Na]^+$: 479.23217, found: 479.23213; IR (ATR) ν (cm^{-1}) thin film, $CHCl_3$: 2928.8 (w), 2857.3 (w), 1263.9 (m), 1027.6 (s), 972.6 (m), 745.9 (m), 701.4 (m); $[\alpha]^{25}_{589}$ = -44.3 (c=1.0 in $CHCl_3$, 94% ee).

(1*S*,2*R*,5*R*)-2-Benzyl-5-(3-(benzyloxy)propyl)-2-methylcyclopent-3-en-1-yl diethyl phosphate (4c)

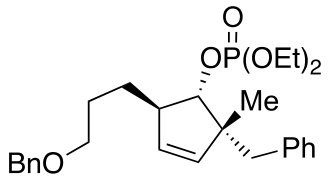

4c

Chemical Formula: $C_{27}H_{37}O_5P$
Molecular Weight: 472.5618

Synthesised according to general procedure 1c, using (1*R*,2*S*,3*S*)-2-benzyl-2-methylcyclopent-4-ene-1,3-bis(diethyl phosphate) (**4a**) and allyl benzyl ether as the alkene partner (hydrozirconation time 20 mins). Purified by flash chromatography (10-30% EtOAc in heptane) to give **4c** as a yellow oil (127 mg, 67%).

SFC analysis indicated an enantiomeric excess of 89% [Chiralpak® IG; flow: 1.5 mL/min; MeOH/CO_2: 1% to 30%; λ = 210 nm; minor enantiomer, t_R = 3.597 min; major enantiomer, t_R = 3.855 min].

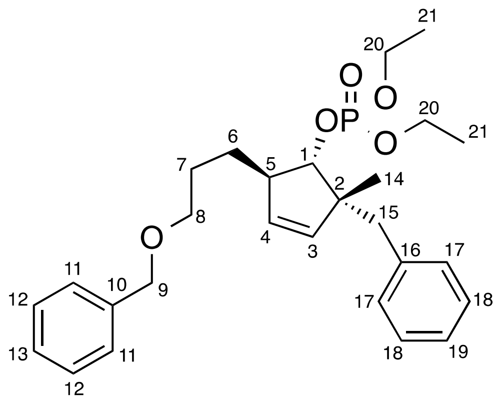

^{1}H NMR (500 MHz, $CDCl_3$) δ_H/ppm: 7.26 (d, *J* = 4.4 Hz, 4H, 4ArH-(11, 12)), 7.23 – 7.19 (m, 1H, ArH-(13)), 7.19 – 7.15 (m, 2H, 2ArH-(18)), 7.14 – 7.10 (m, 1H, ArH-(19)), 7.09 – 7.05 (m, 2H, 2ArH-(17)), 5.51 (d, *J* = 6.3 Hz, 1H, CH-(3)), 5.26 (dd, *J* = 6.3, 2.3 Hz, 1H, CH-(4)), 4.43 (s, 2H, CH_2-(9)), 4.27 (dd, *J* = 8.4, 7.4 Hz, 1H, CH-(1)), 4.14 – 4.04 (m, 4H, 2CH_2-(20)), 3.43 (t, *J* = 6.5 Hz, 2H, CH_2-(8)), 2.78 (d, *J* = 13.0 Hz, 1H, CHH´-(15)), 2.74 – 2.68 (m, 1H, CH-(5)), 2.59 (d, *J* = 13.0 Hz, 1H, CHH´-(15)), 1.81 – 1.73 (m, 1H, CHH´-(6)), 1.73 – 1.65 (m, 1H, CHH´-(7)), 1.64 – 1.55 (m, 1H, CHH´-(7)), 1.40 – 1.34 (m, 1H, CHH´-(6)), 1.31 (td, *J* = 7.1, 1.0 Hz, 3H, CH_3-(21)), 1.27 (td, *J* = 7.1, 1.0 Hz, 3H, CH_3-(21)), 1.06 (s, 3H, CH_3-(14)); ^{13}C NMR (101 MHz, $CDCl_3$) δ_c/ppm: 138.8 (C(16)), 138.7 (C(10)), 137.7 (C(4)), 130.9 (C(17)), 129.7 (C(3)), 128.5 (C(18)), 127.7 (C(12)), 127.7 (C(11)), 127.6 (C(13)), 126.0 (C(19)), 91.1 (C(1)), 73.0 (C(9)), 70.6 (C(8)), 63.9 (C(20)), 50.7 (C(2)), 50.0 (C(5)), 41.1 (C(15)), 28.9 (C(6)), 27.8 (C(7)), 24.5 (C(14)), 16.3 (C(21)); ^{31}P NMR (162 MHz, $CDCl_3$) δ_P/ppm: -1.19; HRMS (ESI) *m/z* calcd for $C_{27}H_{37}O_5NaP$ $[M+Na]^+$: 495.22708, found: 495.22637; IR (ATR) ν (cm^{-1}) thin film, $CHCl_3$: 2930.5 (w), 1453.5 (w), 1262.4 (w), 1102.1 (w), 1025.6 (s), 974.3 (w), 742.3 (w), 702.0 (w); $[\alpha]^{25}_{589}$ = -98.6 (c=1.0 in $CHCl_3$, 89% ee).

(1*S*,2*R*,5*R*)-2-Benzyl-5-(4-bromobutyl)-2-methylcyclopent-3-en-1-yl diethyl phosphate (4d)

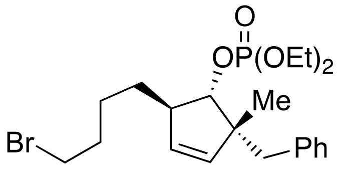

4d

Chemical Formula: $C_{21}H_{32}BrO_4P$
Molecular Weight: 459.3608

Synthesised according to general procedure 1c, using (1*R*,2*S*,3*S*)-2-benzyl-2-methylcyclopent-4-ene-1,3-bis(diethyl phosphate) (**4a**) and 4-bromobut-1-ene as the alkene partner (hydrozirconation time 20 mins). Purified by flash chromatography (10-30% EtOAc in heptane) to give **4d** as a yellow oil (81 mg, 44%).

The product was derivatised according to general procedure 2 for HPLC analysis.

HPLC analysis indicated an enantiomeric excess of 90% [Chiralpak® IC; flow: 0.8 mL/min; hexane/iPrOH: 98:2; λ = 210 nm; major enantiomer, t_R = 22.900 min ; minor enantiomer, t_R = 25.200 min].

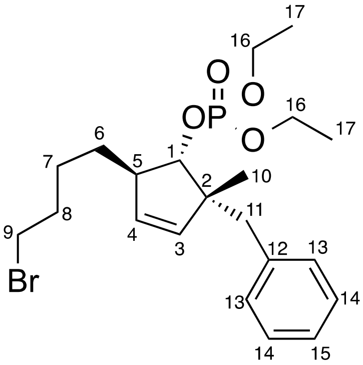

^{1}H NMR (500 MHz, $CDCl_3$) δ_H/ppm: 7.20 – 7.15 (m, 2H, 2ArH-(14)), 7.14 – 7.10 (m, 1H, ArH-(15)), 7.08 – 7.05 (m, 2H, 2ArH-(13)), 5.50 (d, *J* = 6.3 Hz, 1H, CH-(3)), 5.27 (dd, *J* = 6.3, 2.4 Hz, 1H, CH-(2)), 4.28 – 4.24 (m, 1H, CH-(1)), 4.15 – 4.06 (m, 4H, $2CH_2$-(16)), 3.35 (td, *J* = 6.8, 1.2 Hz, 2H, CH_2-(9)), 2.78 (d, *J* = 13.0 Hz, 1H, CHH´-(11)), 2.72 – 2.66 (m, 1H, CH-(5)), 2.58 (d, *J* = 13.0 Hz, 1H, CHH´-(11)), 1.86 – 1.78 (m, 2H, CH_2-(8)), 1.75 – 1.66 (m, 1H, CHH´-(6)), 1.57 – 1.48 (m, 1H, CHH´-(7)), 1.47 – 1.38 (m, 1H, CHH´-(7)), 1.35 – 1.29 (m, 6H, $2CH_3$-(17)), 1.33 – 1.26 (m, 1H, CHH´-(6)) 1.06 (s, 3H, CH_3-(10)); ^{13}C NMR (126 MHz, $CDCl_3$) δ_C/ppm: 138.7 (C(12)), 137.8 (C(4)), 130.9 (C(13)), 129.5 (C(3)), 127.8 (C(14)), 126.1 (C(15)), 91.0 (C(1)), 64.0 (C(16)), 50.7 (C(2)), 50.0 (C(5)), 41.1 (C(15)), 33.8 (C(9)), 33.0 (C(8)), 31.5 (C(6)), 26.1 (C(7)), 24.6 (C(10)), 16.4 (C(17)); ^{31}P NMR (162 MHz, $CDCl_3$) δ_P/ppm: -1.13; HRMS (ESI) *m/z* calcd for $C_{21}H_{32}O_4{}^{81}BrNaP$ $[M+Na]^+$: 483.10933, found: 483.10854; IR (ATR) ν (cm^{-1}) thin film, $CHCl_3$: 2931.0 (w), 1453.7 (w), 1263.0 (m), 1023.2 (s), 972.0 (m), 746.8 (w), 703.8 (w); $[\alpha]^{25}{}_{589}$ = -75.5 (c=1.0 in $CHCl_3$, 90% ee).

(1*S*,2*R*,5*R*)-2-Benzyl-5-(2-bromo-5-fluorophenethyl)-2-methylcyclopent-3-en-1-yl diethyl phosphate (4e)

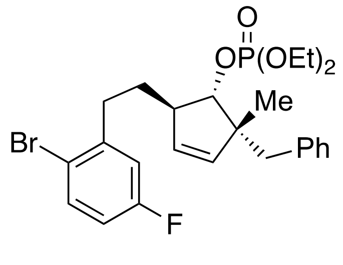

4e

Chemical Formula: $C_{25}H_{31}BrFO_4P$
Molecular Weight: 525.3952

Synthesised according to general procedure 1c, using (1*R*,2*S*,3*S*)-2-benzyl-2-methylcyclopent-4-ene-1,3-bis(diethyl phosphate) (**4a**) and 2-bromo-5-fluorostyene as the alkene partner (hydrozirconation time 20 mins). Purified by flash chromatography (10-30% EtOAc in heptane) to give **4e** as a yellow oil (112 mg, 53%).

SFC was performed on a Waters SFC 15 system equipped with a Waters 2998 PDA (UV) detector and Waters SQD2 mass spectrometer using a Daicel Chiralpak IA column (10 x 250 mm, 5 μm). Mobile phase was 90% CO_2 and 10% methanol with 20mM NH_3. The flow rate was 10 mL/min, the back pressure was 120 bar, and the column temperature was 40 °C. The PDA detector range was 210 – 400 nm.

SFC analysis indicated an enantiomeric excess of 86; minor enantiomer, t_R = 4.80 min ; major enantiomer, t_R = 5.14 min].

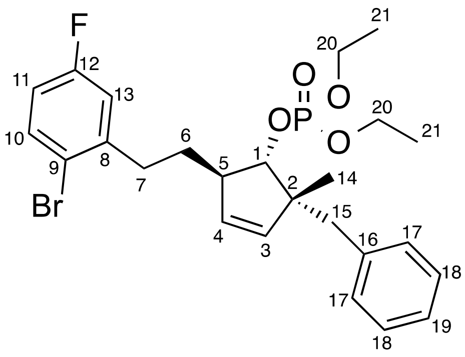

^1H NMR (500 MHz, $CDCl_3$) δ_H/ppm: 7.39 (dd, *J* = 8.8, 5.4 Hz, 1H, ArH-(10)), 7.19 – 7.15 (m, 2H, 2ArH-(18)), 7.14 – 7.10 (m, 1H, ArH-(19)), 7.09 – 7.06 (m, 2H, 2ArH-(17)), 6.90 (dd, *J* = 9.4, 3.1 Hz, 1H, ArH-(13)), 6.71 (td, *J* = 8.3, 3.0 Hz, 1H, ArH-(11)), 5.57 (d, *J* = 6.3 Hz, 1H, CH-(3)), 5.32 (dd, *J* = 6.3, 2.3 Hz, 1H, CH-(4)), 4.35 – 4.31 (m, 1H, CH-(1)), 4.15 – 4.04 (m, 4H, $2CH_2$-(20)), 2.80 (d, *J* = 13.1 Hz, 1H, CHH´-(15)), 2.78 – 2.66 (m, 3H, CH-(5), CH_2-(7)), 2.59 (d, *J* = 13.0 Hz, 1H, CHH´-(15)), 2.02 – 1.94 (m, 1H, , CHH´-(6)), 1.63 – 1.54 (m, 1H, CHH´-(6)), 1.31 (td, *J* = 7.1, 1.0 Hz, 3H, CH_3-(21)), 1.28 (td, *J* = 7.1, 1.0 Hz, 3H, CH_3-(21)), 1.08 (s, 3H, CH_3-(14)); ^{13}C NMR (126 MHz, $CDCl_3$) δ_C/ppm: 162.1 (C(12)), 143.7 (C(8)), 138.7 (C(16)), 138.1 (C(4)), 133.9 (C(10)), 130.9 (C(17)), 129.3 (C(3)), 127.8 (C(18)), 126.1 (C(19)), 118.5 (C(9)), 117.3 (C(13)), 114.8 (C(11)), 90.8 (C(1)), 64.0 (C(20)), 50.8 (C(2)), 49.8 (C(5)), 41.1 (C(15)), 34.2 (C(7)), 32.3 (C(6)), 24.6 (C(14)), 16.4 (C(21)); ^{31}P NMR (162 MHz, $CDCl_3$) δ_P/ppm: -1.05; HRMS (ESI) *m/z* calcd for $C_{25}H_{32}O_4{}^{79}BrFP$ $[M+H]^+$: 525.12001, found: 525.09136; IR (ATR) ν (cm^{-1}) thin film, $CHCl_3$: 2980.6 (m), 1470.0 (w), 1392.1 (w), 1261.6 (w), 1155.3 (w), 1025.2 (s), 964.5 (m), 808.3 (w), 744.6 (w), 703.6 (w); $[\alpha]^{25}_{589}$ = -101.1 (c=1.0 in $CHCl_3$, 86% ee).

(1*S*,2*R*,5*R*)-2-Benzyl-5-(4-(dibenzylamino)butyl)-2-methylcyclopent-3-en-1-yl diethyl phosphate (4f)

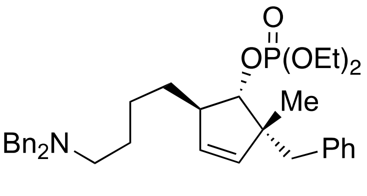

4f

Chemical Formula: $C_{35}H_{46}NO_4P$
Molecular Weight: 575.7298

Synthesised according to general procedure 1c, using (1*R*,2*S*,3*S*)-2-benzyl-2-methylcyclopent-4-ene-1,3-bis(diethyl phosphate) (**4a**) and N,N-dibenzylbut-3-en-1-amine as the alkene partner (hydrozirconation time 20 mins). Purified by flash chromatography (10-30% EtOAc in heptane) to give **4f** as a yellow oil (145 mg, 63%).

SFC was performed on a Waters UPC system equipped with a PDA (UV) detector and QDa (MS) detector using a Daicel Chiralpak IA column (3.0 x 50 mm, 3 μm).

Mobile phase A was CO_2 and mobile phase B was methanol with 20mM NH_3. A gradient from 5% to 50% mobile phase B over 3.5 minutes was used, followed by an increase to 60% over 0.05 minutes, a hold at 60% for 0.4 minutes and return to 5%. The total flow was 2 mL/min, the back pressure was 140 bar, and the column temperature was 40 °C. The PDA detector range was 210 – 400 nm.

The product was derivatised according to general procedure 2 (reduction only) for SFC analysis.

SFC analysis indicated an enantiomeric excess of 86%; minor enantiomer, t_R = 2.03 min ; major enantiomer, t_R = 2.21 min].

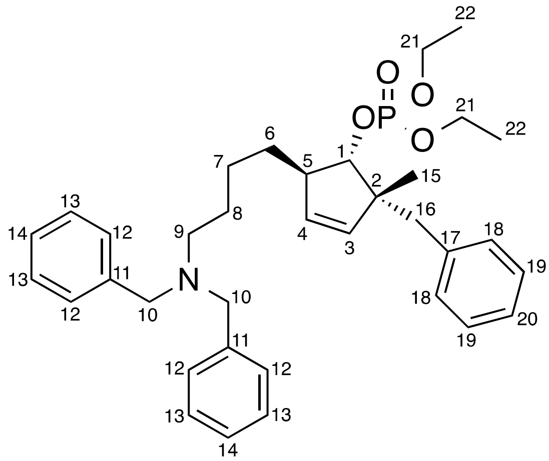

^{1}H NMR (500 MHz, $CDCl_3$) δ_H/ppm: 7.30 (d, *J* = 7.2 Hz, 4H, 4ArH-(12)), 7.23 (t, *J* = 7.6 Hz, 4H, 4ArH-(13)), 7.20 – 7.14 (m, 4H, 4ArH, (14, 19)), 7.14 (s, 1H, ArH-(20)), 7.09 – 7.06 (m, 2H, 2ArH-(18)), 5.47 (d, *J* = 6.3 Hz, 1H, CH-(3)), 5.25 (dd, *J* = 6.3, 2.4 Hz, 1H, CH-(4)), 4.21 (dd, *J* = 8.4, 7.4 Hz, 1H, CH-(1)), 4.13 – 4.02 (m, 4H, $2CH_2$-(21)), 3.55 – 3.45 (m, 4H, $2CH_2$-(10)), 2.78 (d, *J* = 13.0 Hz, 1H, CHH´-(15)), 2.66 – 2.61 (m, 1H, CH-(5)), 2.58 (d, *J* = 13.0 Hz, 1H, CHH´-(15)), 2.37 (t, *J* = 7.6 Hz, 2H, CH_2-(9)), 1.66 – 1.58 (m, 1H, CHH´-(6)), 1.56 – 1.40 (m, 2H, CH_2-(8)), 1.37 – 1.27 (m, 1H, CHH´-(7)), 1.30 (td, *J* = 7.1, 1.0 Hz, 3H, CH_3-(22)), 1.26 (td, *J* = 7.1, 1.0 Hz, 3H, CH_3-(22)), 1.24 – 1.18 (m, 1H, CHH´-(6)), 1.05 (s, 3H, CH_3-(15)); ^{13}C

NMR (126 MHz, $CDCl_3$) δ_C/ppm: 138.7 (C(17)), 137.3 (C(4)), 130.8 (C(18)), 129.7 (C(3)), 128.8 (C(12)), 128.2 (C(13)), 127.6 (C(14), 127.6 (C(19)), 126.8 (C(11)), 125.9 (C(20)), 91.1 (C(1)), 63.8 (C(21)), 58.3 (C(10)), 53.4 (C(9)), 50.5 (C(2)), 50.1 (C(5)), 40.9 (C(16)), 32.2 (C(6)), 27.2 (C(8)), 25.2 (C(7)), 24.4 (C(15)), 16.2 (C(22)); ^{31}P NMR (162 MHz, $CDCl_3$) δ_P/ppm: -1.22; HRMS (ESI) *m/z* calcd for $C_{35}H_{47}O_4NP$ $[M+H]^+$: 576.32372, found: 576.32325; IR (ATR) ν (cm^{-1}) thin film, $CHCl_3$: 2930.4 (w), 1453.2 (w), 1262.9 (m), 1026.9 (s), 973.3 (m), 745.7 (m), 700.8 (m); $[\alpha]^{25}_{589}$ = -94.0 (c=1.0 in $CHCl_3$, 86% ee).

(1*S*,5*R*)-2,2-Dimethyl-5-(4-phenylbutyl)cyclopent-3-en-1-yl diethyl phosphate (5b)

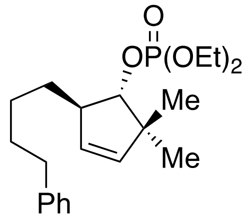

5b

Chemical Formula: $C_{21}H_{33}O_4P$
Molecular Weight: 380.4648

Synthesised according to general procedure 1c, using *cis*-2,2-dimethylcyclopent-4-ene-1,3-bis(diethyl phosphate) (**5a**) and 4-phenyl-1-butene as the alkene partner (hydrozirconation time 20 mins). Purified by flash chromatography (10-20-30% EtOAc in petroleum ether) to give **5b** as a yellow oil (112 mg, 74%).

HPLC analysis indicated an enantiomeric excess of 91% [Chiralpak® ID; flow: 0.8 mL/min; hexane/iPrOH: 96:4; λ = 210 nm; major enantiomer, t_R = 18.136 min; minor enantiomer, t_R = 18.920 min].

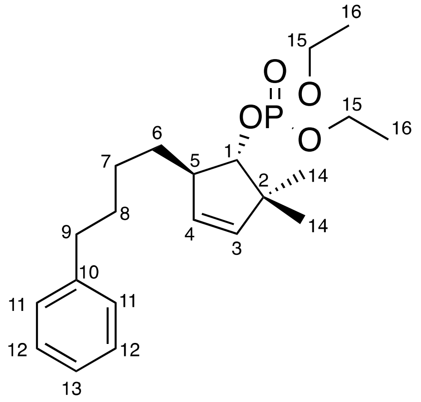

^{1}H NMR (400 MHz, $CDCl_3$) δ_H/ppm: 7.30 – 7.24 (m, 2H, 2ArH-(12)), 7.20 – 7.14 (m, 3H, 3ArH-(11, 13)), 5.47 (s, 1H, CH-(3/4)), 5.47 (s, 1H, CH-(3/4)), 4.22 (dd, J = 8.3, 6.7 Hz, 1H, CH-(1)), 4.12 (h, J = 7.1 Hz, 4H, 2CH_2-(15)), 2.79 – 2.71 (m, 1H, CH-(5)), 2.64 – 2.59 (m, 2H, CH_2-(9)), 1.82 – 1.71 (m, 1H, CHH´-(6)), 1.71 – 1.59 (m, 2H, CH_2-(8)), 1.53 – 1.44 (m, 1H, CHH´-(6)), 1.43 – 1.29 (m, 2H, CH_2-(8)), 1.34 (td, J = 7.1, 1.0 Hz, 6H, 2CH_3-(16)), 1.16 (s, 3H, CH_3-(14)), 1.02 (s, 3H, CH_3-(14)); ^{13}C NMR (101 MHz, $CDCl_3$) δ_c/ppm: 142.7 (C(10)), 139.4 (C(4)), 128.9 (C(3)), 128.5 (C(12)), 128.3 (C(11)), 125.7 (C(13)), 90.5 (C(1)), 63.7 (C(15)), 50.5 (C5)), 47.1 (C(2)), 36.0 (C(9)), 32.5 (C(6)), 31.8 (C(8)), 27.3 (C(7)), 27.3 (C14)), 21.6 (C(14)), 16.3 (C(16)); ^{31}P NMR (162 MHz, $CDCl_3$) δ_P/ppm: -1.20; HRMS (ESI) *m/z* calcd for $C_{21}H_{33}O_4NaP$ $[M+Na]^+$: 403.20087, found: 403.20079; IR (ATR) ν (cm^{-1}) thin film, $CHCl_3$: 2980.31 (w), 2929.6 (w), 2858.9 (w), 1263.6 (w), 1027.3 (s), 970.6 (m), 747.1 (w), 700.0 (w); $[\alpha]^{25}_{589}$ = -61.6 (c=1.0 in $CHCl_3$, 91% ee).

(1*S*,2*R*,5*R*)-2-Allyl-2-methyl-5-(4-phenylbutyl)cyclopent-3-en-1-yl diethyl phosphate (6b)

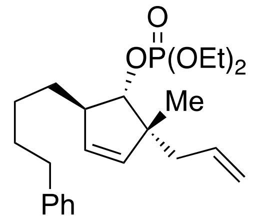

6b

Chemical Formula: $C_{23}H_{35}O_4P$
Molecular Weight: 406.5028

Synthesised according to general procedure 1c, using (1*R*,2*S*,3*S*)-2-allyl-2-methylcyclopent-4-ene-1,3-bis(diethyl phosphate) (**6a**) and 4-phenyl-1-butene as the alkene partner (hydrozirconation time 20 mins). Purified by flash chromatography (10-20-30% EtOAc in petroleum ether) to give **6b** as a yellow oil (143 mg, 88%).

HPLC analysis indicated an enantiomeric excess of 94% [Chiralpak® ID; flow: 1.0 mL/min; hexane/iPrOH: 95:5; λ = 210 nm; major enantiomer, t_R = 12.123 min; minor enantiomer, t_R = 12.992 min].

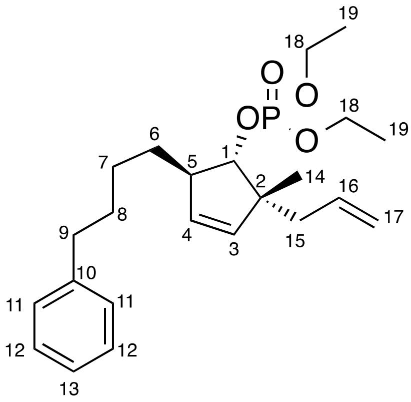

^{1}H NMR (400 MHz, $CDCl_3$) δ_H/ppm: 7.30 – 7.24 (m, 2H, 2ArH-(12)), 7.20 – 7.14 (m, 3H, 3ArH-(11, 13)), 5.87 – 5.74 (m, 1H, CH-(16)), 5.55 (dd, *J* = 6.2, 0.9 Hz, 1H, CH-(3)), 5.48 (dd, *J* = 6.2, 2.2 Hz, 1H, CH-(4)), 5.05 – 5.02 (m, 1H, CHH´-(18)), 5.01 – 4.98 (m, 1H, CHH´-(17)), 4.27 (dd, *J* = 8.4, 7.0 Hz, 1H, CH-(1)), 4.17 – 4.07 (m, 4H, $2CH_2$-(18)), 2.78 – 2.71 (m, 1H, CH-(5)), 2.62 (td, *J* = 8.0, 2.1 Hz, 2H, CH_2-(9)), 2.28 (dd, *J* = 13.5, 7.1 Hz, 1H, CHH´-(15)), 2.12 (dd, *J* = 13.5, 7.8 Hz, 1H, CHH´-(15)), 1.82 – 1.73 (m, 1H, CHH´-(6)), 1.69 – 1.60 (m, 2H, CH_2-(8)), 1.53 – 1.45 (m, 1H, CHH´-(6)), 1.44 – 1.29 (m, 2H, CH_2-(7)), 1.37 – 1.31 (m, 6H, $2CH_3$-(19)), 1.15 (s, 3H, CH_3-(19)); ^{13}C NMR (101 MHz, $CDCl_3$) δ_c/ppm: 142.8 (C(10)), 137.3 (C(4)), 135.4 (C(16)), 130.0 (C(3)), 128.5 (C(12)), 128.4 (C(11)), 125.7 (C(13)), 117.3 (C(17)), 91.0 (C(1)), 63.8 (C(18)), 50.8 (C(5)), 50.1 (C2)), 40.1 (C15)), 36.0 (C(9)), 32.6 (C(6)), 31.8 (C(8)), 27.3 (C7)), 25.0 (C(14)), 16.3 (C(19)); ^{31}P NMR (162 MHz, $CDCl_3$) δ_P/ppm: -1.18; HRMS (ESI) *m/z* calcd for $C_{23}H_{35}O_4NaP$ $[M+Na]^+$: 429.21644, found: 429.21652; IR (ATR) ν (cm^{-1}) thin film, $CHCl_3$: 2980.0 (w), 2929.2 (w), 2857.9 (w), 1263.3 (m), 1025.9 (s), 963.8 (m), 748.9 (m), 699.8 (w); $[\alpha]^{25}_{589}$ = -83.5 (c=1.0 in $CHCl_3$, 94% ee).

(1*S*,2*R*,5*R*)-2-(2-chlorobenzyl)-2-methyl-5-(4-phenylbutyl)cyclopent-3-en-1-yl diethyl phosphate (7b)

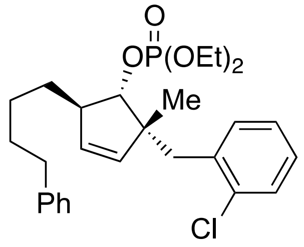

7b

Chemical Formula: $C_{27}H_{36}ClO_4P$
Molecular Weight: 491.0048

Synthesised according to general procedure 1c, using ((1*R*,2*S*,3*S*)-2-(2-chlorobenzyl)-2-methylcyclopent-4-ene-1,3-bis(diethyl phosphate) (**7a**) and 4-phenyl-1-butene as the alkene partner (hydrozirconation time 20 mins). Purified by flash chromatography (10-20-30% EtOAc in petroleum ether) to give **7b** as a yellow oil (154 mg, 79%).

HPLC analysis indicated an enantiomeric excess of 94% [Chiralpak® ID; flow: 1.0 mL/min; hexane/iPrOH: 95:5; λ = 210 nm; major enantiomer, t_R = 19.110 min; minor enantiomer, t_R = 19.878 min].

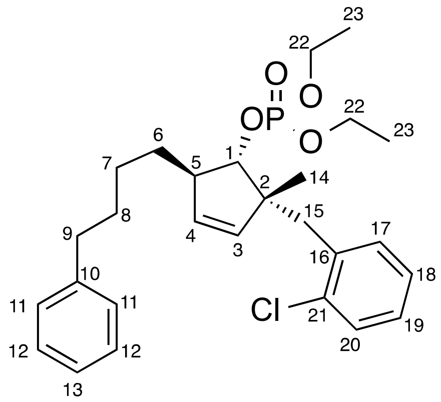

^{1}H NMR (400 MHz, $CDCl_3$) δ_H/ppm: 7.35 – 7.31 (m, 1H, ArH-(20)), 7.30 – 7.25 (m, 2H, 2ArH-(12)), 7.25 – 7.22 (m, 1H, ArH-(19)), 7.21 – 7.16 (m, 3H, 3ArH-(11, 13), 7.16 – 7.12 (m, 2H, 2ArH-(17, 19)), 5.55 (d, *J* = 6.3 Hz, 1H, CH-(3)), 5.40 (dd, *J* = 6.3, 2.3 Hz, 1H, CH-(4)), 4.34 (dd, *J* = 8.6, 7.5 Hz, 1H, CH-(1)), 4.25 – 4.13 (m, 4H, $2CH_2$-(22)), 3.03 (d, *J* = 13.2 Hz, 1H, CHH´-(15)), 2.94 (d, *J* = 13.2 Hz, 1H, CHH´-(15)), 2.87 – 2.78 (m, 1H, CH-(5)), 2.63 (dt, *J* = 7.7, 2.0, 1.7 Hz, 2H, CH_2-(9)), 1.86 – 1.76 (m, 1H, CHH´-(6)), 1.71 – 1.61 (m, 2H, CH_2-(8)), 1.56 – 1.46 (m, 1H, CHH´-

(6)), 1.44 – 1.32 (m, 2H, CH_2-(7)), 1.38 (dtd, *J* = 8.2, 7.1, 1.0 Hz, 6H, 2CH_3-(23)), 1.18 (s, 3H, CH_3-(14)); ^{13}C NMR (101 MHz, $CDCl_3$) δ_c/ppm: 142.8 (C(10)), 137.2 (C(4)), 137.0 (C(16)), 135.4 (C(21)), 132.8 (C(20)), 130.0 (C(3)), 129.7 (C(19)), 128.5 (C(12)), 128.4 (C(11)), 127.6 (C(17)), 126.1 (C(18)), 125.8 (C(13)), 91.2 (C(1)), 63.9 (C(22)), 51.4 (C(2)), 50.2 (C(5)), 36.9 (C(15)), 36.0 (C(9)), 32.2 (C(6)), 31.8 (C(8)), 27.3 (C(7)), 24.7 (C(14)), 16.4(C(23)); ^{31}P NMR (162 MHz, $CDCl_3$) δ_P/ppm: -1.13; HRMS (ESI) *m/z* calcd for $C_{27}H_{36}O_4{}^{35}ClNaP$ $[M+Na]^+$: 513.19319, found: 513.19327; IR (ATR) ν (cm^{-1}) thin film, $CHCl_3$: 2980.7 (w), 2929.7 (w), 2857.6 (w), 1263.9 (m), 1027.3 (s), 971.7 (m), 749.2 (m), 699.7 (w); $[\alpha]^{25}_{589}$ = -81.2 (c=1.0 in $CHCl_3$, 94% ee).

(1*S*,2*S*,5*R*)-2-((benzyloxy)methyl)-2-methyl-5-(4-phenylbutyl)cyclopent-3-en-1-yl diethyl phosphate (8b)

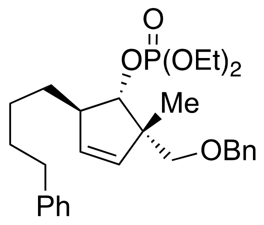

8b

Chemical Formula: $C_{28}H_{39}O_5P$
Molecular Weight: 486.5888

Synthesised according to general procedure 1c, using (1*R*,2*S*,3*S*)-2-((benzyloxy)methyl)-2-methylcyclopent-4-ene-1,3-bis(diethyl phosphate) (**8a**) and 4-phenyl-1-butene as the alkene partner (hydrozirconation time 20 mins). Purified by flash chromatography (10-25-35% EtOAc in petroleum ether) to give **8b** as a yellow oil (128 mg, 66%).

HPLC analysis indicated an enantiomeric excess of 90% [Chiralpak® ID; flow: 1.0 mL/min; hexane/iPrOH: 95:5; λ = 210 nm; major enantiomer, t_R = 18.954 min; minor enantiomer, t_R = 20.164 min].

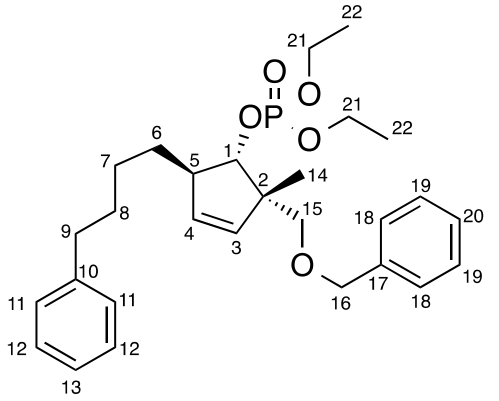

^{1}H NMR (400 MHz, $CDCl_3$) δ_H/ppm: 7.25 – 7.22 (m, 4H, 4ArH-(12, 19)), 7.22 – 7.16 (m, 3H, 3ArH-(11, 13)), 7.12 – 7.07 (m, 3H, 3ArH-(18, 20)), 5.58 – 5.54 (m, 1H, CH-(3)), 5.51 (dd, J = 6.1, 2.0 Hz, 1H, CH-(4)), 4.43 (d, J = 2.8 Hz, 2H, CH_2-(16)), 4.23 (dd, J = 8.5, 6.6 Hz, 1H, CH-(1)), 4.04 – 3.94 (m, 4H, $2CH_2$-(21)), 3.45 (d, J = 9.0 Hz, 1H, CHH´-(15)), 3.33 (d, J = 9.0 Hz, 1H, CHH´-(15)), 2.83 – 2.77 (m, 1H, CH-(5)), 2.58 – 2.51 (m, 2H, CH_2-(9)), 1.74 – 1.64 (m, 1H, CHH´-(6)), 1.62 – 1.51 (m, 2H, CH_2-(8)), 1.45 – 1.36 (m, 1H, CHH´-(6)), 1.36 – 1.25 (m, 2H, CH_2-(7)), 1.21 (qd, J = 6.9, 1.1 Hz, 6H, $2CH_3$-(22)), 1.14 (s, 3H, CH_3-(14)); ^{13}C NMR (101 MHz, $CDCl_3$) δ_c/ppm: 142.8 (C(10)), 138.9 (C(17)), 135.9 (C(4)), 131.2 (C(3)), 128.5 (C(12)), 128.4 (C(11)), 128.3 (C(19)), 127.4 (C(18)), 127.4 (C(20)), 125.7 (C(13)), 90.5 (C(1)), 74.2 (C(15)), 73.5 (C(16)), 63.8 (C(21)), 51.5 (C(2)), 51.4 (C(5)), 36.0 (C(9)), 32.8 (C(6)), 31.8 (C(8)), 27.3 (C(7)), 23.0 (C(14)), 16.3 (C(22)); ^{31}P NMR (162 MHz, $CDCl_3$) δ_P/ppm: -1.15; HRMS (ESI) *m/z* calcd for $C_{28}H_{39}O_5NaP$ $[M+Na]^+$: 509.24273, found: 509.24260; IR (ATR) ν (cm^{-1}) thin film, $CHCl_3$: 2980.7 (w), 2929.4 (w), 2857.0 (w), 1263.3 (m), 1100.2 (w), 1027.0 (s), 972.9 (m), 748.1 (w), 698.9 (w); $[\alpha]^{25}_{589}$ = -83.4 (c=1.0 in $CHCl_3$, 90% ee).

Diethyl ((1*S*,4*R*,5*S*)-4-(4-phenylbutyl)-2',3'-dihydrospiro[cyclopentane-1,1'-inden]-2-en-5-yl) phosphate (9b)

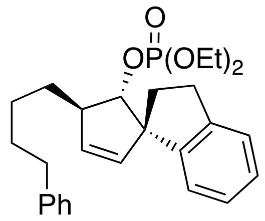

9b

Chemical Formula: $C_{27}H_{35}O_4P$
Molecular Weight: 454.5468

Synthesised according to general procedure 1c, using (1*S*,2*R*,5*S*)-2',3'-dihydrospiro[cyclopentane-1,1'-inden]-3-ene-2,5-bis(diethyl phosphate) (**9a**) and 4-phenyl-1-butene as the alkene partner (hydrozirconation time 20 mins). Purified by flash chromatography (10-20-30% EtOAc in petroleum ether) to give **9b** as a yellow oil (112 mg, 61%).

HPLC analysis indicated an enantiomeric excess of 90% [Chiralpak® ID; flow: 1.0 mL/min; hexane/iPrOH: 95:5; λ = 210 nm; major enantiomer, t_R = 21.376 min; minor enantiomer, t_R = 23.910 min].

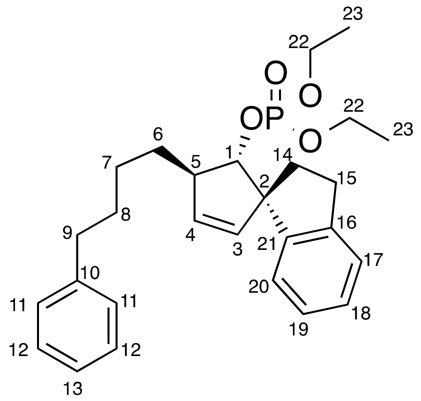

^{1}H NMR (400 MHz, $CDCl_3$) δ_H/ppm: 7.32 – 7.27 (m, 2H, 2ArH-(12), 7.23 – 7.18 (m, 4H, 4ArH-(11, 13, 17), 7.18 – 7.14 (m, 3H, 3ArH-(18, 19, 20), 5.81 (dd, *J* = 6.0, 2.0 Hz, 1H, CH-(3)), 5.63 (dd, *J* = 6.0, 1.7 Hz, 1H, CH-(4)), 4.59 (dd, *J* = 8.6, 4.7 Hz, 1H, CH-(1)), 3.87 – 3.74 (m, 2H, CH_2-(22)), 3.50 – 3.36 (m, 2H, CH_2-(22)), 3.17 – 3.07 (m, 1H, CHH´-(15)), 2.99 – 2.93 (m, 1H, CH-(5)), 2.92 – 2.83 (m, 1H, CHH´-(15)), 2.66 (t, *J* = 7.7 Hz, 2H, CH_2-(9)), 2.24 – 2.17 (m, 2H, CH_2-(14)), 1.85 – 1.74 (m, 1H,

CHH´-(6)), 1.73 – 1.64 (m, 2H, CH_2-(8)), 1.60 – 1.40 (m, 3H, CH_2-(7), CHH´-(6)), 1.19 (td, J = 7.1, 1.1 Hz, 3H, CH_3-(23)), 1.02 (td, J = 7.1, 1.1 Hz, 3H, CH_3-(23)); ^{13}C NMR (101 MHz, $CDCl_3$) δ_c/ppm: 144.6 (C(21)), 143.8 (C(16)), 142.7 (C(10)), 136.6 (C(4)), 132.0 (C(3)), 128.5 (C(12)), 128.4 (C(11)), 127.1 (C(17)), 126.0 (C(20)), 125.9 (C(18)), 125.7 (C(13)), 124.4 (C(19)), 88.7 (C(1)), 64.5 (C(2)), 63.5 (C(22)), 63.2 (C(22)), 52.7 (C(5)), 39.1 (C(14)), 36.0 (C(9)), 33.7 (C(15)), 31.8 (C(8)), 31.0 (C(6)), 27.5 (C(7)), 16.0 (C(23)); ^{31}P NMR (162 MHz, $CDCl_3$) δ_P/ppm: -1.97; HRMS (ESI) *m/z* calcd for $C_{27}H_{36}O_4P$ $[M+H]^+$: 455.23457, found: 455.23463; IR (ATR) ν (cm^{-1}) thin film, $CHCl_3$: 2980.8 (w), 2929.9 (w), 2856.9 (w), 1275.1 (w), 1262.7 (w), 1029.1 (s), 971.2 (m), 772.1 (w), 748.6 (w), 700.0 (w); $[\alpha]^{25}_{589}$ = -111.9 (c=1.0 in $CHCl_3$, 90% ee).

Diethyl ((4S,4'R,5'S)-4'-(4-phenylbutyl)spiro[chromane-4,1'-cyclopentan]-2'-en-5'-yl) phosphate (10b)

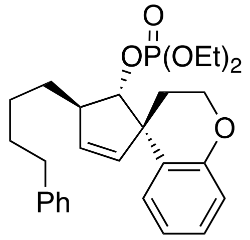

10b

Chemical Formula: $C_{27}H_{35}O_5P$
Molecular Weight: 470.5458

Synthesised according to general procedure 1c, using (2'*R*,4*S*,5'*S*)-spiro[chromane-4,1'-cyclopentan]-3'-ene-2',5'-bis(diethyl phosphate) (**10a**) and 4-phenyl-1-butene as the alkene partner (hydrozirconation time 20 mins). Purified by flash chromatography (10-20-30% EtOAc in petroleum ether) to give **10b** as a yellow oil (107 mg, 57%).

HPLC analysis indicated an enantiomeric excess of 91% [Chiralpak® ID; flow: 1.0 mL/min; hexane/iPrOH: 95:5; λ = 210 nm; major enantiomer, t_R = 30.321 min; minor enantiomer, t_R = 41.961 min].

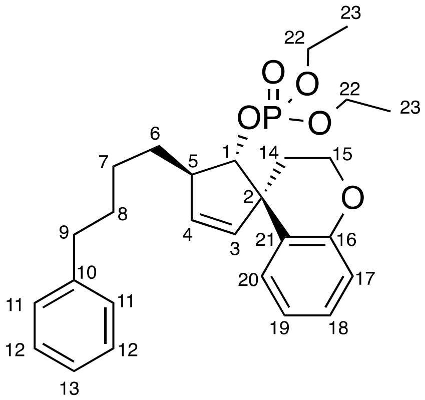

^{1}H NMR (400 MHz, $CDCl_3$) δ_H/ppm: 7.30 – 7.26 (m, 2H, 2ArH-(12)), 7.21 – 7.16 (m, 3H, 3ArH-(11, 13)), 7.11 – 7.06 (m, 2H, 2ArH-(18,20), 6.82 (td, *J* = 7.5, 1.3 Hz, 1H, ArH-(19)), 6.78 (dd, *J* = 8.7, 1.4 Hz, 1H, ArH-(17)), 5.87 (dd, *J* = 6.0, 2.1 Hz, 1H, CH-(3)), 5.54 (dd, *J* = 6.0, 1.9 Hz, 1H, CH-(4)), 4.46 (dd, *J* = 8.8, 4.1 Hz, 1H, CH-(1)), 4.42 (td, *J* = 11.6, 2.2 Hz, 1H, CHH´-(15)), 4.27 (dt, *J* = 11.3, 3.7 Hz, 1H, CHH´-(15)), 3.83 (p, *J* = 7.2 Hz, 2H, CH_2-(22)), 3.53 (dp, *J* = 10.1, 7.1 Hz, 1H, CHH´-(22)), 3.43 (dp, *J* = 10.1, 7.1 Hz, 1H, CHH´-(22)), 2.97 – 2.92 (m, 1H, CH-(5)), 2.64 (t, *J* = 7.7 Hz, 2H, CH_2-(9)), 2.19 – 2.11 (m, 1H, CHH´-(14)), 1.85 – 1.77 (m, 2H, CHH´-(14), CHH´-(6)), 1.72 – 1.63 (m, 2H, CH_2-(8)), 1.54 – 1.43 (m, 3H, CHH´-(6), CH_2-(7)), 1.19 (td, *J* = 7.1, 1.0 Hz, 3H, CH_3-(23)), 1.03 (td, *J* = 7.1, 1.0 Hz, 3H, CH_3-(23)); ^{13}C NMR (125 MHz, $CDCl_3$) δ_c/ppm: 154.7 (C(16)), 142.7 (C(10)), 136.7 (C(4)), 133.0 (C(3)), 130.7 (C(20)), 128.5 (C(12)), 128.4 (C(11)), 128.3 (C(18)), 125.8 (C(13)), 122.0 (C(21)), 119.8 (C(19)), 116.8 (C(17)), 89.9 (C(1)), 63.7 (C(22)), 63.4 (C(22)), 62.9 (C(15)), 53.8 (C(5)), 52.7 (C(2)), 36.0 (C(9)), 35.3 (C(14)), 34.3 (C(6)), 31.7 (C(8)), 27.6 (C(7)), 16.1 (C(23)); ^{31}P NMR (162 MHz, $CDCl_3$) δ_P/ppm: -2.29; HRMS (ESI) *m/z* calcd for $C_{27}H_{35}O_5NaP$ $[M+H]^+$: 493.21143, found: 493.21134; IR (ATR) ν (cm^{-1}) thin film, $CHCl_3$: 2980.7 (w), 2929.6 (w), 2857.1 (w), 1488.3 (w), 1452.0 (w), 1261.4 (w), 1224.3 (w), 1027.8 (s), 975.3 (m), 755.0 (w), 700.2 (w); $[\alpha]^{25}_{589}$ = -62.9 (c=1.0 in $CHCl_3$, 91% ee).

Diethyl ((3*S*,4*R*)-4-(4-phenylbutyl)-2,3,4,7-tetrahydrooxepin-3-yl) phosphate (11b)

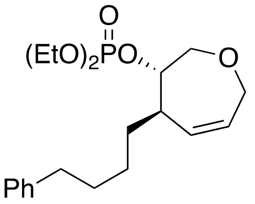

11b

Chemical Formula: $C_{20}H_{31}O_5P$
Molecular Weight: 382.44

Synthesised according to general procedure 1c, using tetraethyl ((3*R*,6*S*)-2,3,6,7-tetrahydrooxepine-3,6-diyl) bis(phosphate) (**11a**) and 4-phenyl-1-butene as the alkene partner (hydrozirconation time 20 mins). Purified by flash chromatography (10-30-40% EtOAc in petroleum ether) to give **11b** as a yellow oil (109 mg, 71%).

SFC analysis indicated an enantiomeric excess of 92% [Chiralpak® IG; flow: 1.5 mL/min; MeOH/CO_2: 1% to 30%; λ = 210 nm; minor enantiomer, t_R = 2.584 min; major enantiomer, t_R = 2.680 min].

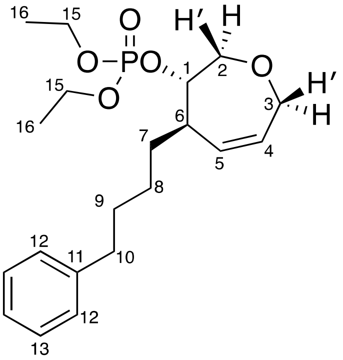

^{1}H NMR (400 MHz, $CDCl_3$) δ_H/ppm: 7.30 – 7.24 (m, 2H, 2ArH-(13)), 7.19 – 7.14 (m, 3H, 3ArH-(12, 14)), 5.61 – 5.55 (m, 1H, CH-(4)), 5.50 – 5.44 (m, 1H, CH-(5)), 4.40 (tt, *J* = 7.9, 3.6 Hz, 1H, CH-(1)), 4.33 (dq, *J* = 16.7, 2.4 Hz, 1H, CHH´-(3)), 4.16 (dt, *J* = 4.0, 1.6 Hz, 1H, CHH´-(3)), 4.13 – 4.04 (m, 4H, 2CH_2-(15)), 3.97 (dd, *J* = 4.9, 3.6 Hz, 2H, CH_2-(2)), 2.89 – 2.80 (m, 1H, CH-(6)), 2.61 (t, *J* = 7.6 Hz, 2H, CH_2-(10)), 1.74 – 1.60 (m, 3H, CH_2-(9), CHH´-(8)), 1.57 – 1.43 (m, 2H, CHH´-(7, 8)), 1.41 – 1.35 (m, 1H, CHH´-(7)), 1.32 (tdd, *J* = 7.1, 3.6, 1.0 Hz, 6H, 2CH_3-(16)); ^{13}C NMR

(101 MHz, $CDCl_3$) δ_C/ppm: 142.7 (C(11)), 130.1 (C(5)), 129.7 (C(4)), 128.5 (C(12)), 128.4 (C(13)), 125.8 (C(14)), 81.0 (C(1)), 73.2 (C(2)), 70.9 (C(3)), 63.9 (C(15)), 41.9 (C(6)), 36.0 (C(10)), 32.1 (C(8)), 31.7 (C(9)), 26.8 (C(7)), 16.3 (C(16)); ^{31}P NMR (162 MHz, $CDCl_3$) δ_P/ppm: –1.58; HRMS (ESI) *m/z* calcd for $C_{20}H_{32}O_5P$ $[M+H]^+$: 383.19819, found: 383.19822; IR (ATR) ν (cm^{-1}) thin film, $CHCl_3$: 3025.9 (w), 2926.1 (w), 2855.6 (w), 1496.7 (w), 1274.1 (w), 1103.1 (w), 1029.9 (s), 748.7 (w), 699.7 (w); $[\alpha]^{25}{}_{589}$ = -46.0 (c=1.0 in $CHCl_3$, 92% ee).

***tert*-Butyl (3*S*,4*R*)-3-((diethoxyphosphoryl)oxy)-4-(4-phenylbutyl)-2,3,4,7-tetrahydro-1*H*-azepine-1-carboxylate (12b)**

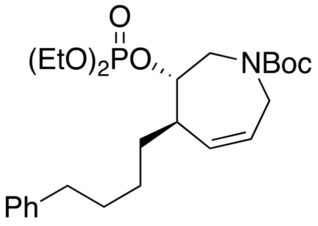

12b

Chemical Formula: $C_{25}H_{40}NO_6P$
Molecular Weight: 481.57

Synthesised according to general procedure 1c, using *tert*-butyl (3*R*,6*S*)-3,6-bis((diethoxyphosphoryl)oxy)-2,3,6,7-tetrahydro-1*H*-azepine-1-carboxylate (**12a**) and 4-phenyl-1-butene as the alkene partner (hydrozirconation time 20 mins). Purified by flash chromatography (10-30-40% EtOAc in petroleum ether) to give **12b** as a yellow oil (140 mg, 73%). Obtained as a mixture of rotamers.

SFC was performed on a Waters UPC system equipped with a PDA (UV) detector and QDa (MS) detector using a Lux Cellulose 2 column (3.0 x 50 mm, 3 μm). Mobile phase A was CO_2 and mobile phase B was IPA with 20mM NH_3. A gradient from 5% to 50% mobile phase B over 3.5 minutes was used, followed by an increase to 60% over 0.05 minutes, a hold at 60% for 0.4 minutes and return to 5%. The total flow was 2 mL/min, the back pressure was 140 bar, and the column temperature was 40 °C. The PDA detector range was 210 – 400 nm.

SFC analysis indicated an enantiomeric excess of 93%, major enantiomer, t_R = 2.27min; minor enantiomer, t_R = 2.77 min].

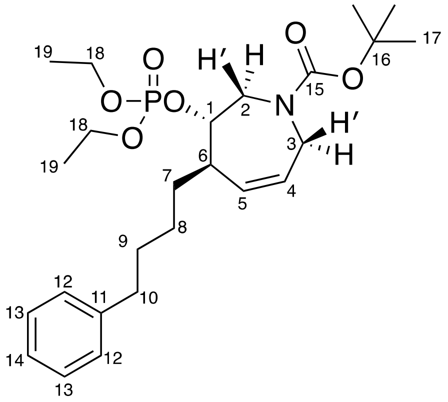

^{1}H NMR (500 MHz, DMSO-d_6, 338K) δ_H/ppm: 7.26 (t, J = 7.6 Hz, 2H, 2ArH- (13)), 7.19 – 7.13 (m, 3H, 3ArH-(12, 14)), 5.71 – 5.66 (m, 1H, CH-(4)), 5.52 – 5.46 (m, 1H, CH-(5)), 4.37 – 4.29 (m, 1H, CH-(1)), 4.08 – 4.00 (m, 2H, 2CH_2-(18)), 3.98 – 3.89 (m, 2H, CH_2-(3)), 3.89 – 3.83 (m, 1H, CHH´-(2)), 3.56 (dd, J = 14.7, 5.2 Hz, 1H, CHH´-(2)), 2.59 (t, J = 7.6 Hz, 2H, CH_2-(10)), 2.62 – 2.54 (m, 1H, CH-(6)), 1.64 – 1.57 (m, 3H, CH_2-(9), CHH´-(7)), 1.42 (s, 9H, 3CH_3-(17)), 1.46 – 1.31 (m, 3H, CH_2-(8), CHH´-(7)), 1.27 (td, J = 7.2, 5.9 Hz, 6H, 2CH_3-(19)); ^{13}C NMR (101 MHz, $CDCl_3$) δ_c/ppm: 155.3, 142.6, 131.0, 130.3, 128.9, 128.6, 128.5, 128.4, 125.8, 80.3, 79.9, 79.0, 63.9, 50.6, 46.8, 46.6, 42.3, 36.0, 32.5, 32.1, 31.7, 28.5, 26.9, 16.3; ^{31}P NMR (162 MHz, $CDCl_3$) δ_P/ppm: –1.60; HRMS (ESI) *m/z* calcd for $C_{25}H_{41}O_6NP$ $[M+H]^+$: 482.26660, found: 482.26662; IR (ATR) ν (cm^{-1}) thin film, $CHCl_3$: 2928.9 (w), 2856.8 (w), 1694.3 (s), 1248.3 (m), 1166.8 (m), 1131.0 (w), 1008.6 (s), 749.4 (w), 699.9 (w); $[\alpha]^{25}_{589}$ = -94.1 (c=1.0 in $CHCl_3$, 93% ee).

Diethyl ((1*S*,2*R*)-2-(4-phenylbutyl)cyclopent-3-en-1-yl) phosphate (14)

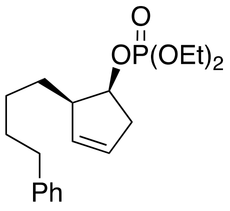

14

Chemical Formula: $C_{19}H_{29}O_4P$
Molecular Weight: 352.4108

Synthesised according to general procedure 1b, using (1*S*,3*S*)-cyclopent-4-ene-1,3-diyl tetraethyl bis(phosphate) (**13**) and 4-phenyl-1-butene as the alkene partner (hydrozirconation time 20 mins). Purified by flash chromatography (20-40% EtOAc in petroleum ether) to give **14** as a yellow oil (91 mg, 65%).

HPLC analysis indicated an enantiomeric excess of >99% [Chiralpak® IA; flow: 1.0 mL/min; hexane/iPrOH: 95:5; λ = 210 nm; minor enantiomer, t_R = 9.663 min; major enantiomer, t_R = 10.616 min].

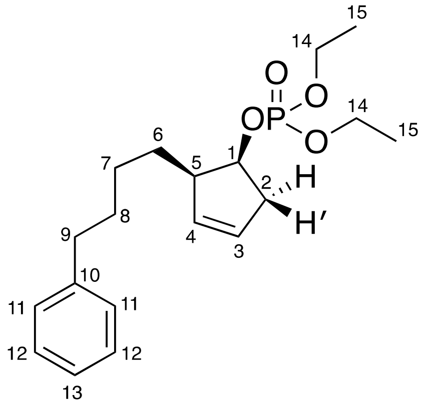

^{1}H NMR (400 MHz, $CDCl_3$) δ_H/ppm: 7.30 – 7.24 (m, 2H, ArH-(12)), 7.20 – 7.15 (m, 3H, ArH-(11, 13)), 5.72 – 5.67 (m, 2H, 2CH-(3, 4)), 4.99 (dtd, *J* = 6.8, 5.7, 3.7 Hz, 1H, CH-(1)), 4.14 – 4.03 (m, 4H, $2CH_2$-(14)), 2.74 – 2.66 (m, 1H, CH-(5)), 2.65 – 2.58 (m, 4H, $2CH_2$-(2, 9)), 1.71 – 1.59 (m, 3H, CH_2-(8), CHH′-(6)), 1.50 – 1.40 (m, 3H, CH_2-(7), CHH′-(6)), 1.32 (td, *J* = 7.1, 1.0 Hz, 6H, $2CH_3$-(15)); ^{13}C NMR (101 MHz, $CDCl_3$) δ_C/ppm: 142.8 (C(10)), 133.2 (C(4)), 128.5 (C(11)), 128.4 (C(12)), 127.4 (C(3)), 125.8 (C(13)), 79.8 (C(1)), 63.7 (C(14)), 49.1 (C(5)), 39.9 (C(2)), 36.1 (C(9)), 32.0 (C(6)), 28.1 (C(8)), 27.8 (C(7)), 16.3 (C(15)); ^{31}P NMR (162 MHz,

$CDCl_3$) δ_P/ppm: 7.26; HRMS (ESI) *m/z* calcd for $C_{19}H_{29}O_4NaP$ $[M+Na]^+$: 375.16957, found: 375.16960; IR (ATR) ν (cm^{-1}) thin film, $CHCl_3$: 2981.3 (w), 2932.2 (w), 2857.9 (w), 2360.2 (w), 1603.9 (w), 1454.2 (w), 1393.0 (w), 1262.5 (m), 1165.6 (w), 1029.5 (s), 973.5 (m), 819.8 (w), 746.5 (w), 700.2 (w); $[\alpha]^{25}_{589}$ = -36.1 (c=1.0 in $CHCl_3$, >99% ee).

Derivatisation Products

(1*R*,2*R*)-2-ethylcyclopent-3-en-1-ol (15)

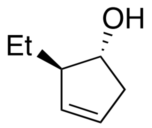

15

Chemical Formula: $C_7H_{12}O$
Molecular Weight: 112.1720

To a solution of **3** (98 µL, 0.4 mmol, 1.0 eq) in Et_2O was added $LiAlH_4$ (1.0 M in Et_2O, 0.89 mL, 0.89 mmol, 2.2 eq) at 0 ˚C. The mixture was left to stir at room temperature for 90 mins before quenching with dropwise addition of H_2O, until effervescence stopped. The resulting residue was suspended in CH_2Cl_2 and filtered through celite, washing the filter pad with CH_2Cl_2. The filtrate was evaporated *in vacuo* and purified on silica (40% Et_2O in pentane) to give **15** as a colourless oil (43 mg, 96%; **NOTE**: Product is volatile and should not be dried by high vacuum. Rotary evaporator at 35 ˚C, 150 mbar was sufficient).

The product was derivatised according to general procedure 2 (esterification process only) for HPLC analysis.

HPLC analysis indicated an enantiomeric excess of 90% [Chiralpak® ID; flow: 0.7 mL/min; hexane/iPrOH: 99:1; λ = 210 nm; minor enantiomer, t_R = 14.157 min; major enantiomer, t_R = 14.785 min].

^1H NMR (500 MHz, $CDCl_3$) δ_H/ppm: 5.68 – 5.65 (m, 1H, CH-(4)), 5.64 – 5.61 (m, 1H, CH-(3)), 4.66 (tt, *J* = 6.6, 3.1 Hz, 1H, CH-(1)), 4.08 (dtd, *J* = 14.6, 7.1, 3.2 Hz, 4H, $2CH_2$-(8)), 2.77 – 2.72 (m, 1H, C***H***H´-(2)), 2.73 – 2.67 (m, 1H, CH-(5)), 2.51 – 2.44 (m, 1H, CH***H´***-(2)), 1.52 – 1.39 (m, 1H, CHH´-(6)), 1.39 – 1.33 (m, 1H, CHH´-(6)), 1.31 (tdd, *J* = 7.1, 2.3, 1.0 Hz, 6H, CH_3-(9)), 0.93 (t, *J* = 7.5 Hz, 3H, $2CH_3$-(7)); ^{13}C NMR (126 MHz, $CDCl_3$) δ_C/ppm: 132.5 (C(4)), 127.3 (C(3)), 82.9 (C(1), 63.7 (C(8)), 54.6 (C(5)), 40.0 (C(2)), 25.6 (C(6)), 16.3 (C(9)), 11.8 (C(7)). Consistent with data found in the literature.[14]

Diethyl ((1*R*,4a*R*,10a*R*)-1,4,4a,9,10,10a-hexahydrophenanthren-1-yl) phosphate (16)

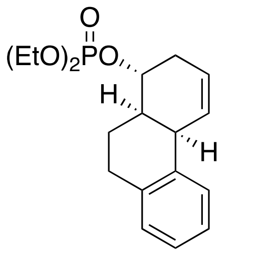

16

Chemical Formula: $C_{18}H_{25}O_4P$
Molecular Weight: 336.3678

A 10 mL round bottomed flask was charged with $Pd(OAc)_2$ (12 mg, 0.054 mmol, 0.15 eq), 1,3-bis(diphenylphosphino)propane (45 mg, 0.108 mmol, 0.30 eq) and K_2CO_3 (199 mg, 1.44 mmol, 4.0 eq). The flask was purged with Ar 3 times and capped with a rubber septum. The mixture was suspended in toluene (4 mL), before adding **2l** (119 µL, 0.36 mmol, 1.0 eq). The flask was wrapped in parafilm to prevent loss of solvent, and stirred at 110 ˚C (dark brown mixture forms) for 21 hours. The reaction mixture was cooled to room temperature, poured over H_2O (50 mL) and extracted with CH_2Cl_2 (3 x 50 mL). The organic layers were combined, washed with brine (50 mL), dried ($MgSO_4$) and evaporated *in vacuo* to give a brown oil. The oil was purified on silica (20-30-40% EtOAc in petroleum ether) to give **16** as a yellow oil (97 mg, 80%).

HPLC analysis indicated an enantiomeric excess of 91% [Chiralpak® IA; flow: 1.0 mL/min; hexane/iPrOH: 95:5; λ = 210 nm; major enantiomer, t_R = 8.687 min; minor enantiomer, t_R = 11.302 min].

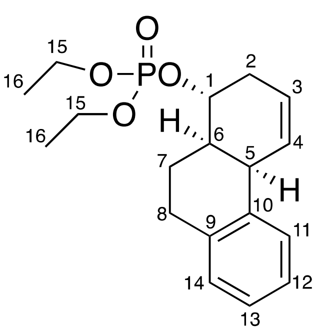

^{1}H NMR (500 MHz, $CDCl_3$) δ_H/ppm: 7.25 (d, *J* = 7.6 Hz, 1H, ArH-(11)), 7.18 (td, *J* = 7.4, 1.5 Hz, 1H, ArH-(13)), 7.13 (td, *J* = 7.4, 1.5 Hz, 1H, ArH-(12)), 7.08 (d, *J* = 7.3

Hz, 1H, ArH-(14)), 5.78 – 5.71 (m, 1H, CH-(4)), 5.55 – 5.48 (m, 1H, CH-(3)), 4.70 (dq, J = 7.6, 3.8 Hz, 1H, CH-(1)), 4.16 – 4.06 (m, 4H, CH_2-(15)), 3.69 (m, 1H, CH-(5)), 2.88 – 2.74 (m, 2H, CH_2-(8)), 2.44 – 2.38 (m, 1H, CH-(6)), 2.36 (m, 2H, CH_2-(2)), 1.75 – 1.56 (m, 2H, CH_2-(7)), 1.33 (dtd, J = 11.1, 7.1, 1.0 Hz, 6H, CH_3-(16)); ^{13}C NMR (126 MHz, $CDCl_3$) δ_C/ppm: 138.8 (C(10)), 136.6 (C(9)), 131.5 (C(4)), 129.3 (C(11)), 129.1 (C(14)), 126.4 (C(13)), 126.1 (C(12)), 120.8 (C(3)), 76.3 (C(1)), 63.8 (C(15)), 37.6 (C(6)), 35.7 (C(5)), 28.9 (C(8)), 28.6 (C(2)), 22.6 (C(7)), 16.3 (C(16)); ^{31}P NMR (162 MHz, $CDCl_3$) δ_P/ppm: -1.21; HRMS (ESI) *m/z* calcd for $C_{18}H_{26}O_4P$ $[M+H]^+$: 337.15632, found: 337.15604; IR (ATR) ν (cm^{-1}) thin film, $CHCl_3$: 2982.2 (w), 2929.8 (w), 1488.9 (w), 1448.1 (w), 1392.8 (w), 1261.4 (m), 1165.4 (w), 1028.8 (s), 1009.0 (s), 818.7 (w), 775.1 (w), 744.9 (m), 679.0 (w); $[\alpha]^{25}_{589}$ = -80.3 (c=1.0 in $CHCl_3$, 91% ee).

(1*S*,2*R*,3*R*,4*S*,5*R*)-2-benzyl-3,4-dihydroxy-2-methyl-5-(4-phenylbutyl)cyclopentyl diethyl phosphate (17)

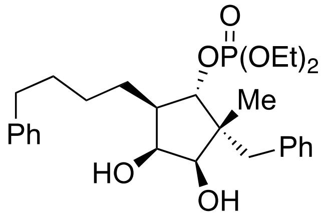

17

Chemical Formula: $C_{27}H_{39}O_6P$
Molecular Weight: 490.5768

To a solution of **4b** (100 mg, 0.22 mmol, 1.0 eq, 94% ee) in acetone (350 µL) and H_2O (90 µL) was added NMO (39 mg, 0.33 mmol, 1.50 eq) and K_2OsO_4•H_2O (3 mg, 0.008 mmol, 4 mol%) at rt. The resulting heterogeneous mixture was stirred for 21 hours at rt. The reaction mixture was quenched with sat. aq. $Na_2S_2O_3$ (1 mL) and stirred for a further 20 minutes. The mixture was extracted with EtOAc (3 x 2 mL). The organic layers were combined, dried ($MgSO_4$) and evaporated *in vacuo* to give a yellow oil. The yellow oil was purified on silica (30-70% EtOAc in heptane) to give **17** as a yellow oil (107 mg, 75%, 94% ee). Other diastereomer observed in crude NMR at a 4:1 ratio in favour of the isolated product.

SFC was performed on a Waters UPC system equipped with a PDA (UV) detector and QDa (MS) detector using a Daicel Chiralpak IC column (3.0 x 50 mm, 3 μm). Mobile phase A was CO_2 and mobile phase B was methanol with 20mM NH_3. A gradient from 5% to 50% mobile phase B over 3.5 minutes was used, followed by an increase to 60% over 0.05 minutes, a hold at 60% for 0.4 minutes and return to 5%. The total flow was 2 mL/min, the back pressure was 140 bar, and the column temperature was 40 °C. The PDA detector range was 210 – 400 nm.

SFC analysis indicated an enantiomeric excess of 94%; major enantiomer, t_R = 1.65 min ; minor enantiomer, t_R = 2.20 min].

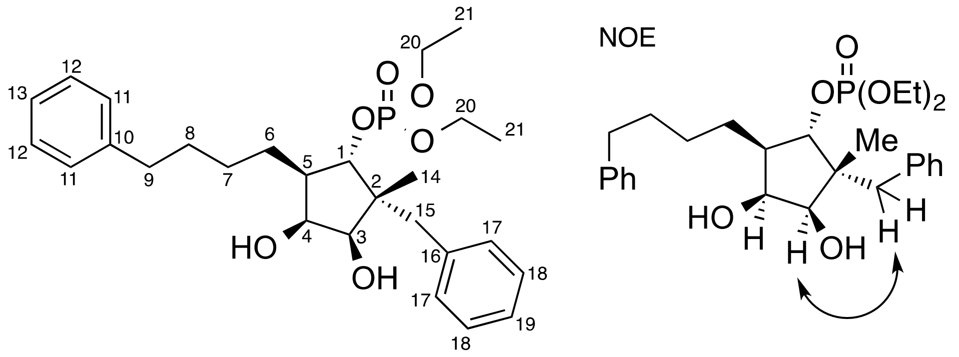

^{1}H NMR (500 MHz, $CDCl_3$) δ_H/ppm: 7.31 – 7.26 (m, 4H, 4ArH-(12, 18), 7.25 – 7.20 (m, 3H, 3ArH-(17, 19)), 7.20 – 7.15 (m, 3H, 3ArH-(11, 13)), 4.46 (t, *J* = 9.0 Hz, 1H, CH-(1)), 4.18 – 4.09 (m, 5H, CH-(4)), $2CH_2$-(20)), 4.03 (d, *J* = 4.5 Hz, 1H, CH-(3)), 2.77 (d, *J* = 13.5 Hz, 1H, CHH´-(15)), 2.70 – 2.57 (m, 2H), 2.65 (d, *J* = 12.1 Hz, 1H, CHH´-(15)), 1.97 – 1.89 (m, 1H, CH-(5)), 1.75 – 1.62 (m, 4H, $2CH_2$-(6, 8)), 1.60 – 1.50 (m, 1H, CHH´-(7)), 1.42 – 1.30 (m, 1H, CHH´-(7)), 1.36 (m, 6H, $2CH_3$-(21)), 1.14 (s, 3H, CH_3-(14)); ^{13}C NMR (126 MHz, $CDCl_3$) δ_c/ppm: 142.8 (C(10)), 138.4 (C(16)), 130.6 (C(17)), 128.5 (C(12)), 128.3 (C(11)), 128.3 (C(18)), 126.5 (C(19)), 125.7 (C(13)), 91.0 (C(1)), 76.9 (C(3)), 71.4 (C(4)), 64.0 (C(20)), 46.3 (C(5)), 46.2 (C(2)), 41.8 (C(15)), 36.0 (C(9)), 31.8 (C(8)), 27.8 (C(7)), 26.6 (C(6)), 21.2 (C(14)), 16.3 (C(21)); ^{31}P NMR (162 MHz, $CDCl_3$) δ_P/ppm: 1.23; HRMS (ESI) *m/z* calcd for $C_{27}H_{39}O_6NaP$ $[M+Na]^+$: 513.23765, found: 513.23778; IR (ATR) ν (cm^{-1}) thin film, $CHCl_3$: 3360.5 (br. w), 3026.6 (w), 2933.1 (w), 1248.7 (w), 1024.2 (s), 981.0 (w), 754.9 (w), 701.2 (w); $[\alpha]^{25}_{589}$ = -42.9 (c=1.0 in $CHCl_3$, 94% ee).

Supplementary Figures 2 - 67

Supplementary Figure 2 - *Meso*-4-cyclopentene-1,3-bisdimethylphosphate

^{1}H NMR (400 MHz, $CDCl_3$)

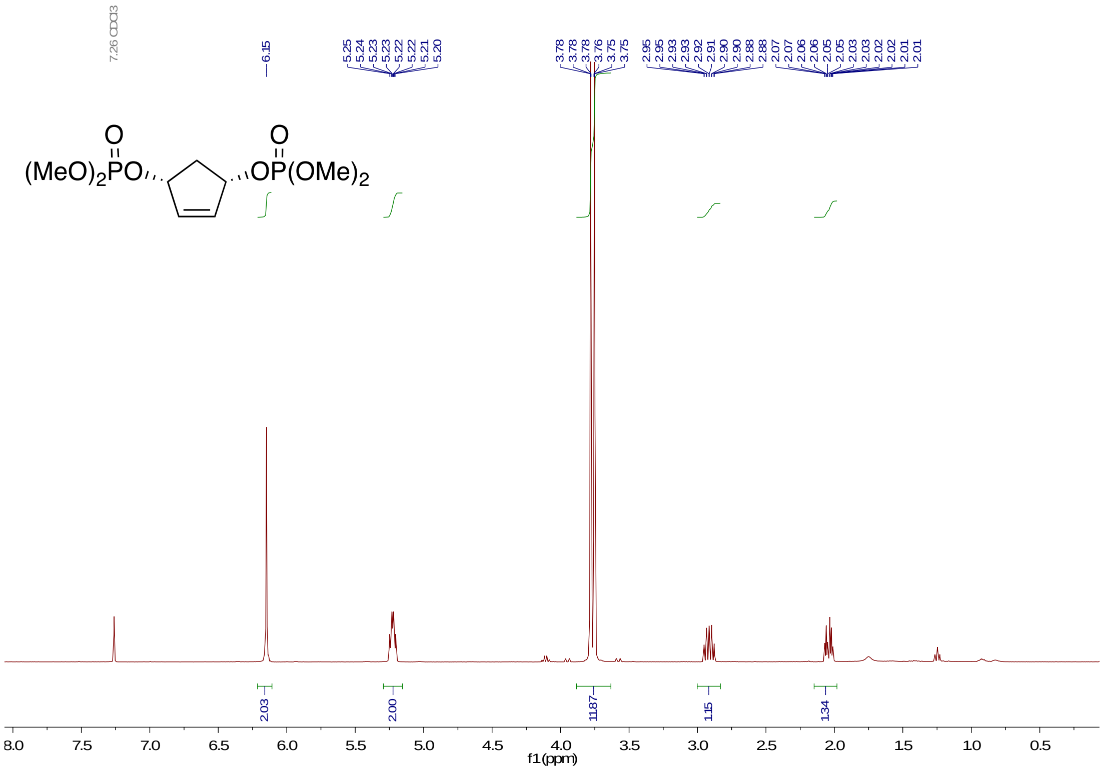

^{13}C NMR (101 MHz, $CDCl_3$)

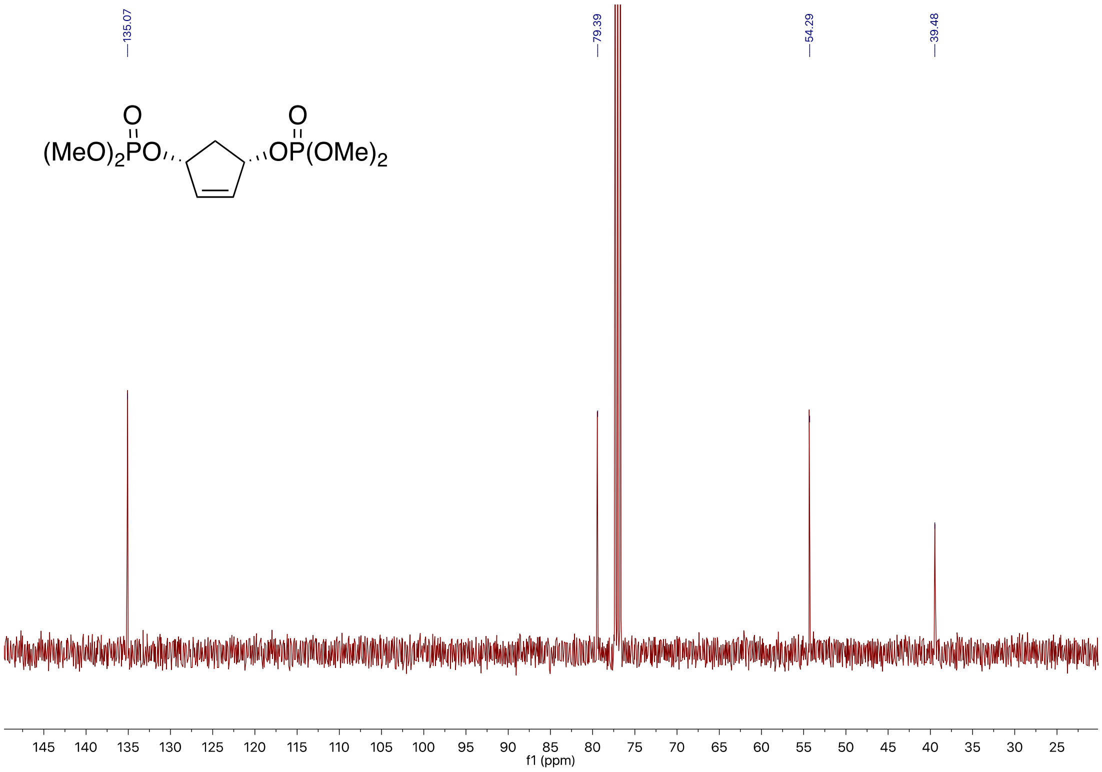

^{31}P NMR (162 MHz, $CDCl_3$)

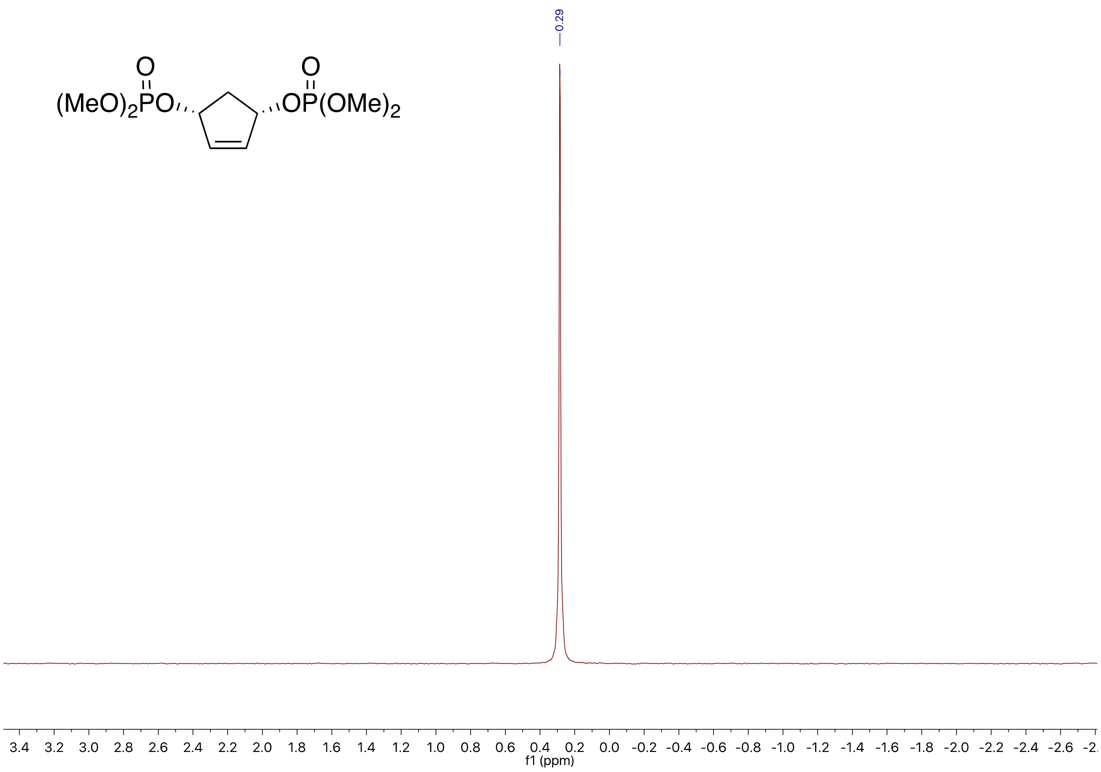

Supplementary Figure 3 - *Meso*-4-cyclopentene-1,3-bisdiethylphosphate (**1a**)
^{1}H NMR (400 MHz, $CDCl_3$)

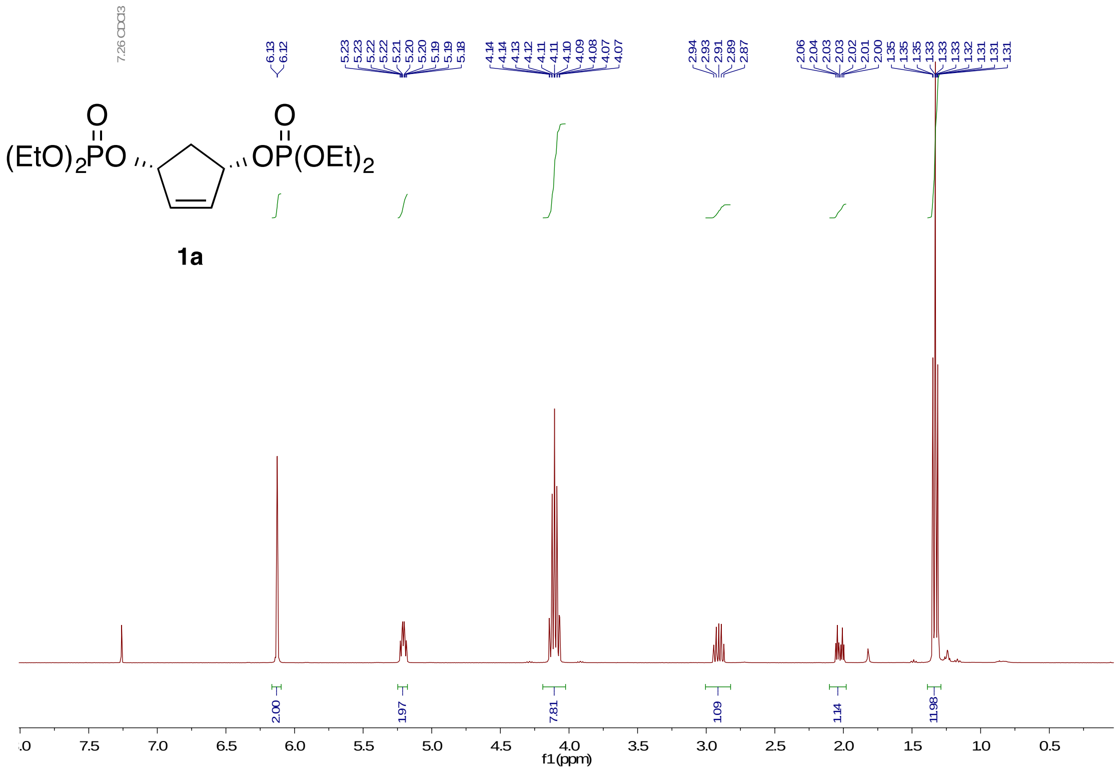

^{13}C NMR (101 MHz, $CDCl_3$)

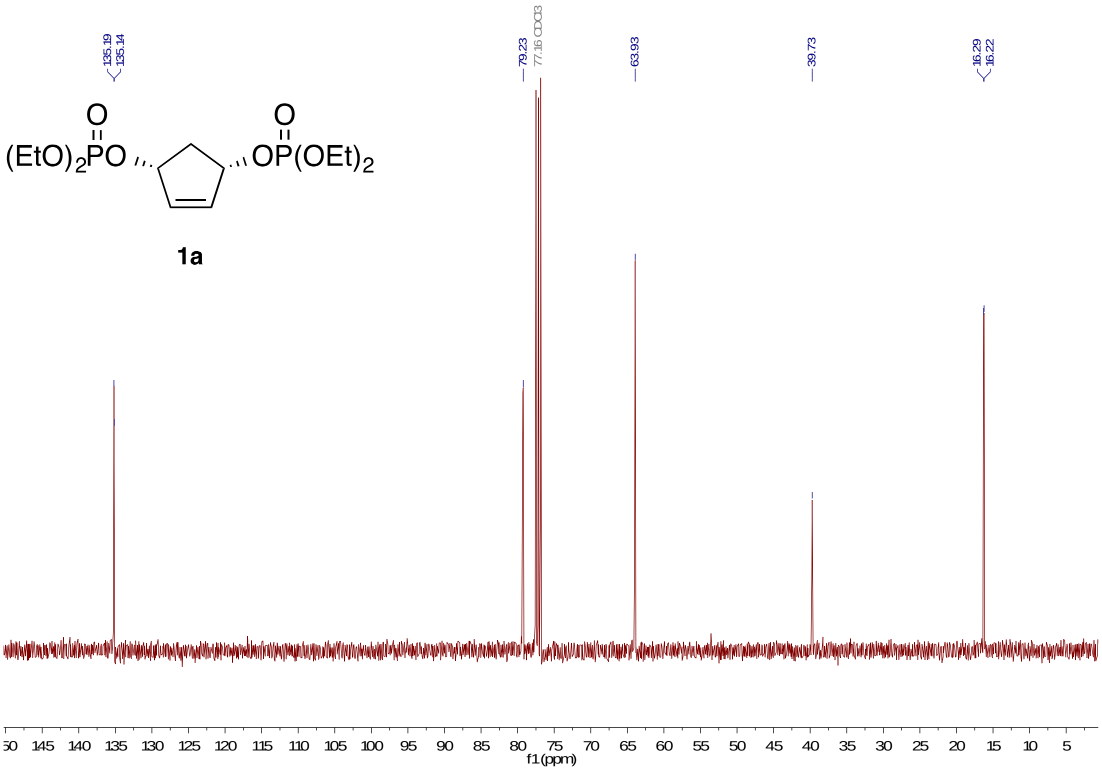

^{31}P NMR (162 MHz, $CDCl_3$)

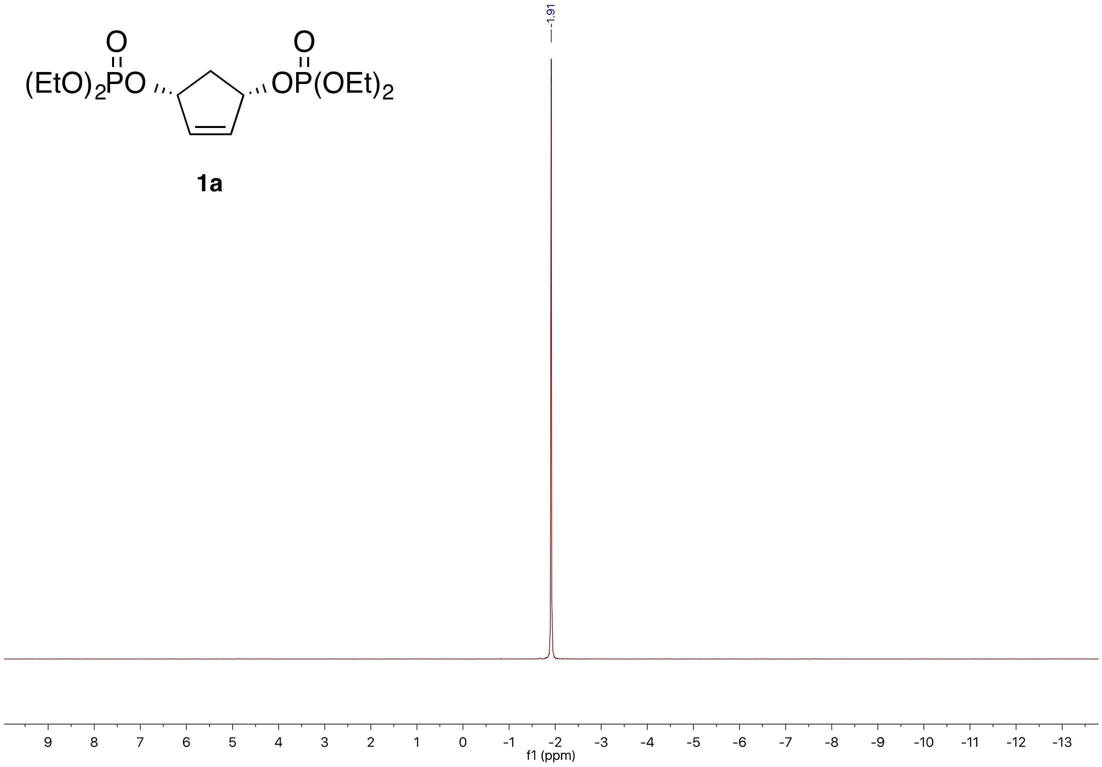

Supplementary Figure 4 - *Meso*-4-cyclopentene-1,3-bisdiisopropylphosphate

^{1}H NMR (400 MHz, $CDCl_3$)

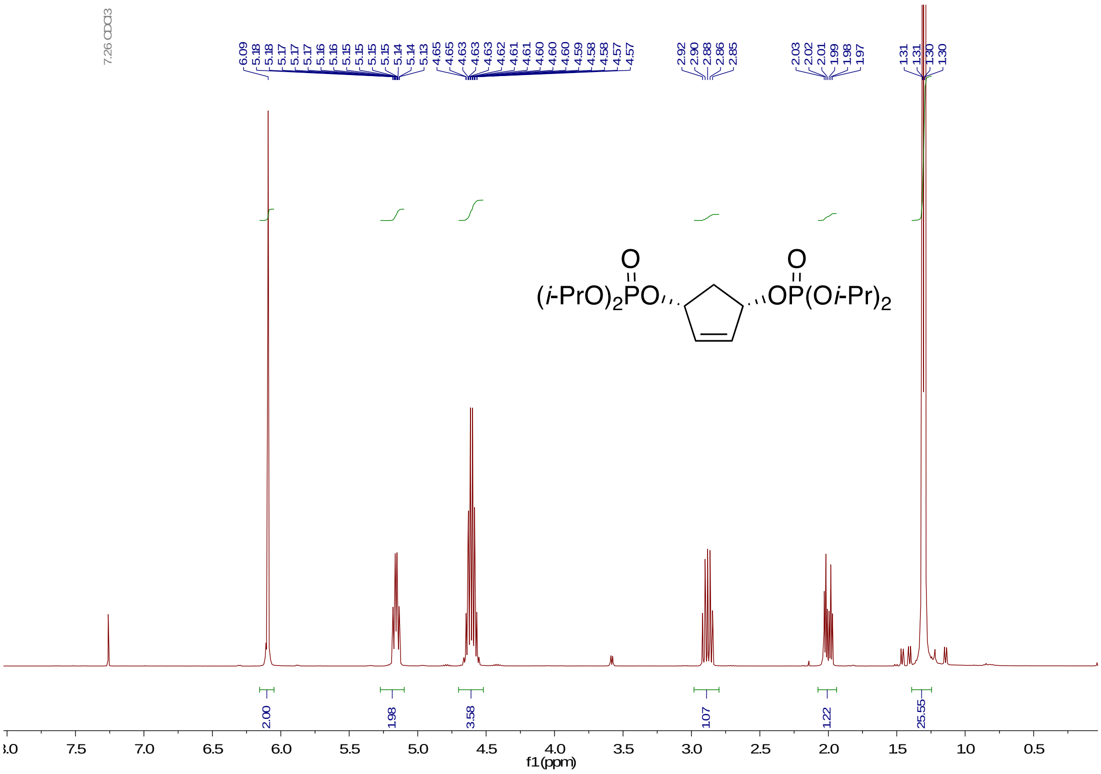

^{13}C NMR (101 MHz, $CDCl_3$)

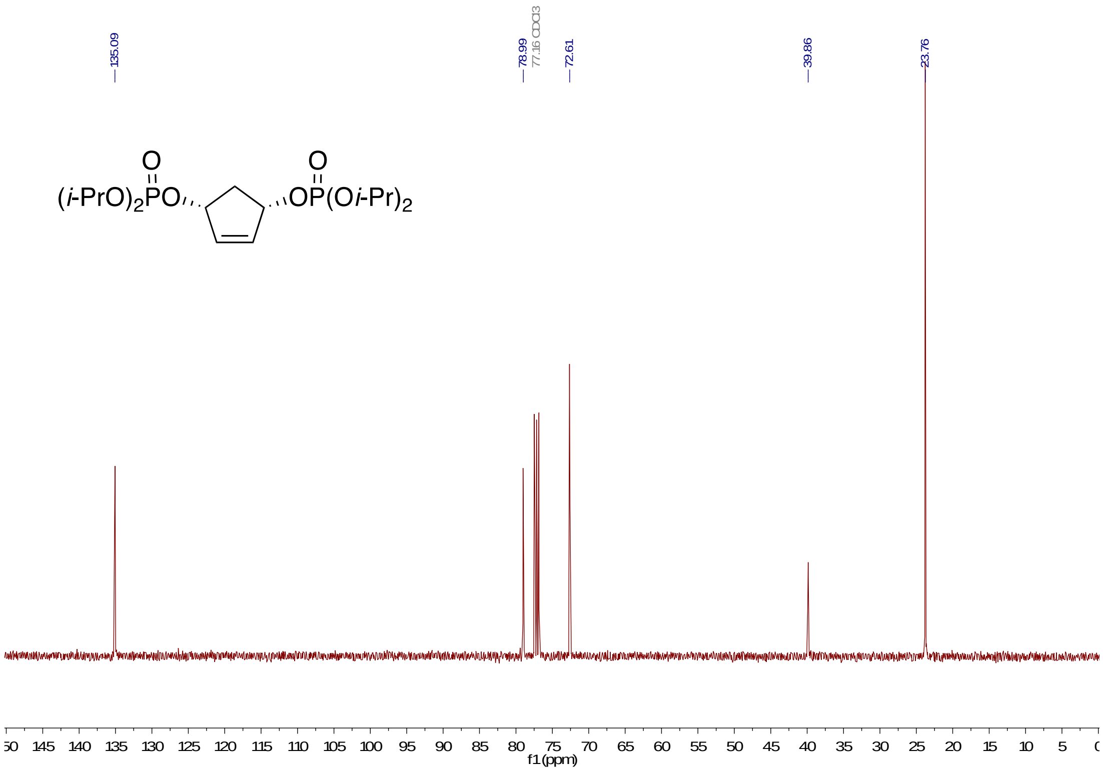

^{31}P NMR (162 MHz, $CDCl_3$)

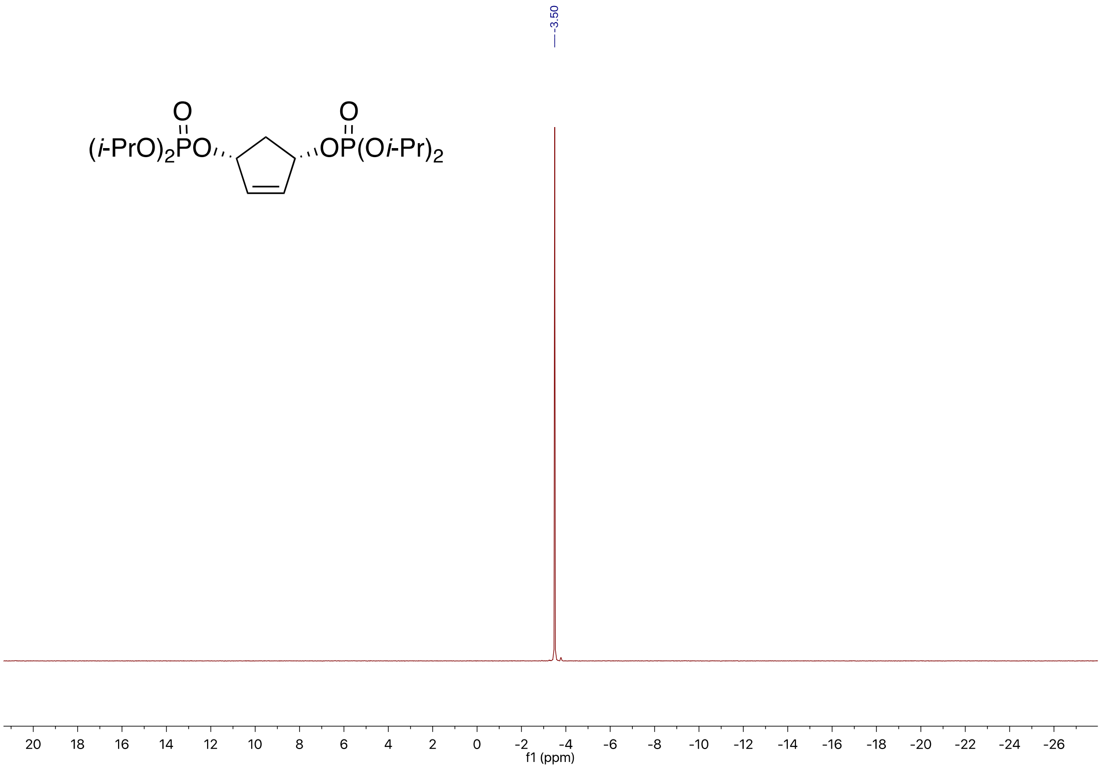

Supplementary Figure 5 - *Meso*-4-cyclopentene-1,3-bisdiphenylphosphate
^{1}H NMR (400 MHz, $CDCl_3$)

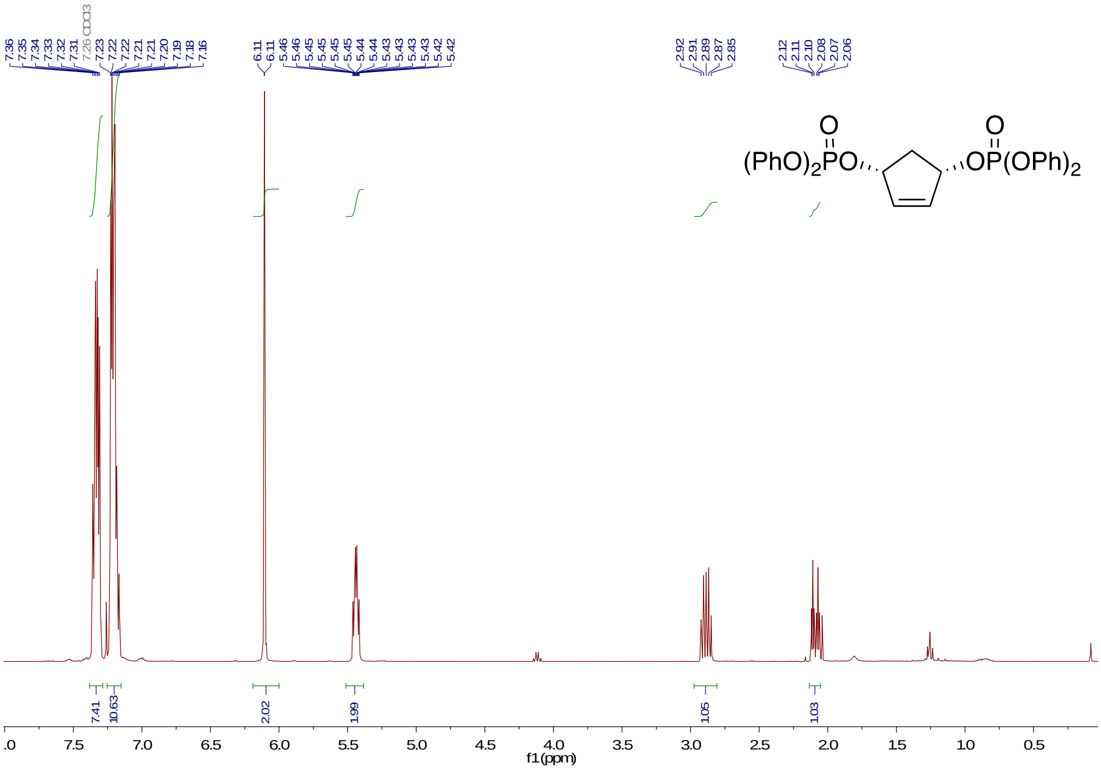

^{13}C NMR (101 MHz, CDCl$_3$)

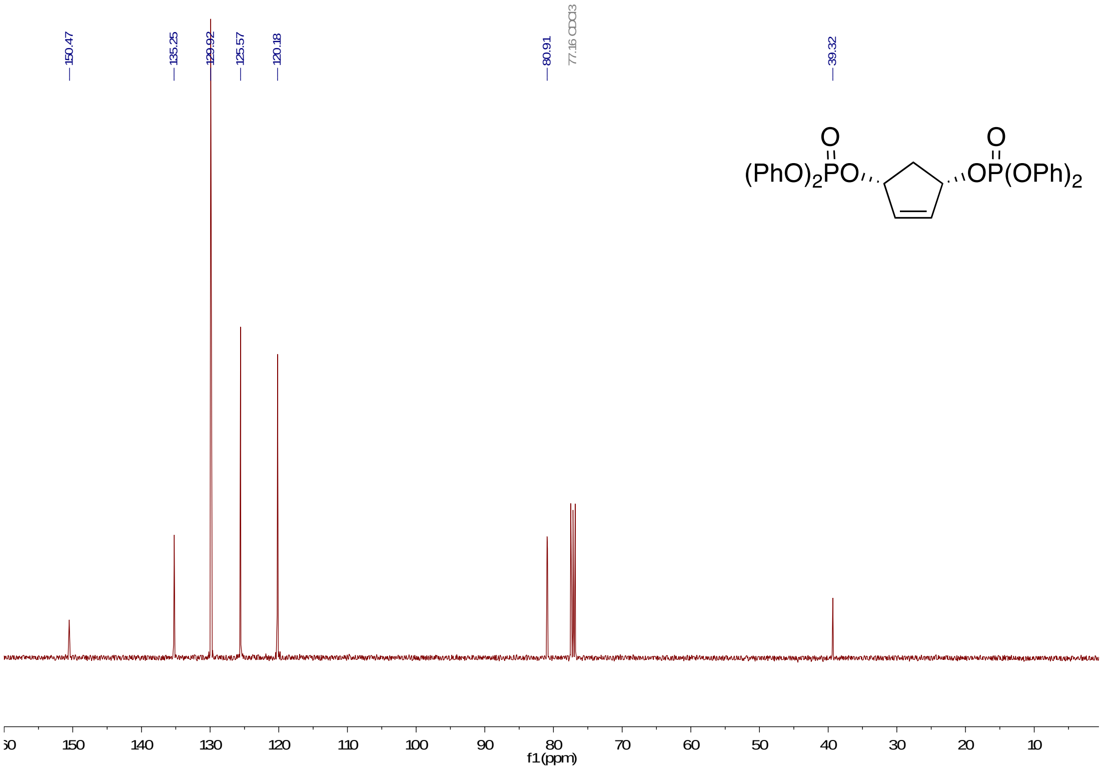

^{31}P NMR (162 MHz, CDCl$_3$)

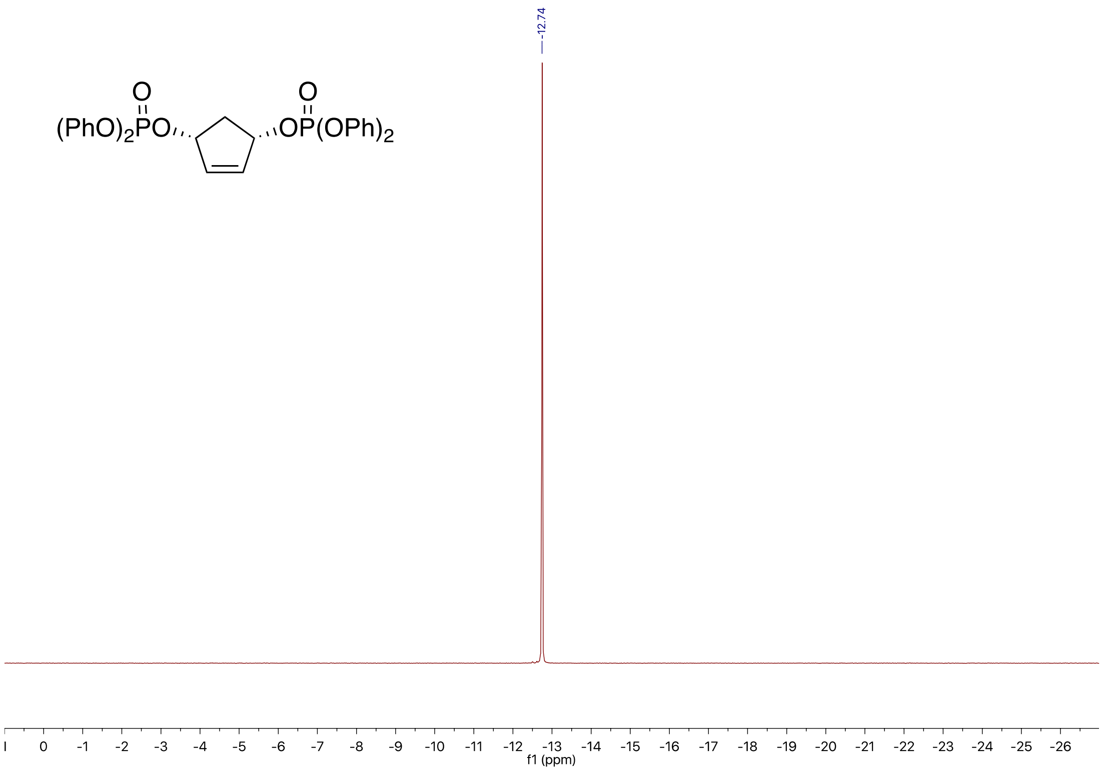

Supplementary Figure 6 - *Meso*-5-cyclohexene-1,4-bisdiethylphosphate (**2a**)

^{1}H NMR (400 MHz, $CDCl_3$)

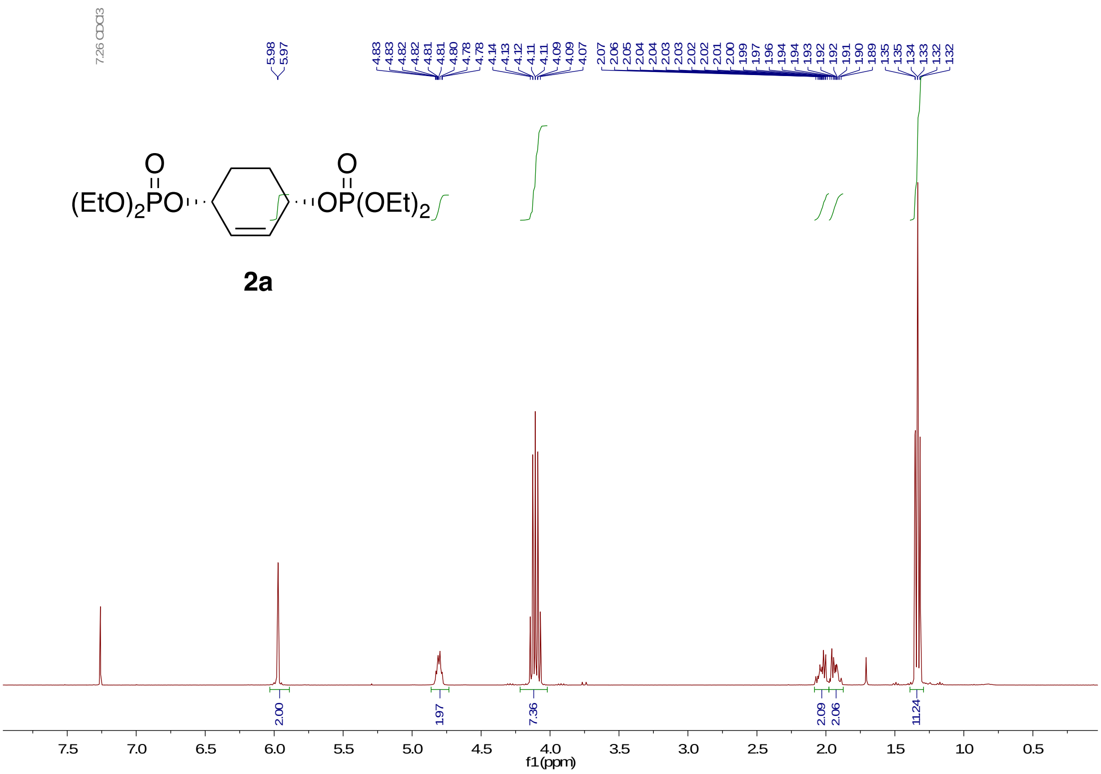

^{13}C NMR (101 MHz, $CDCl_3$)

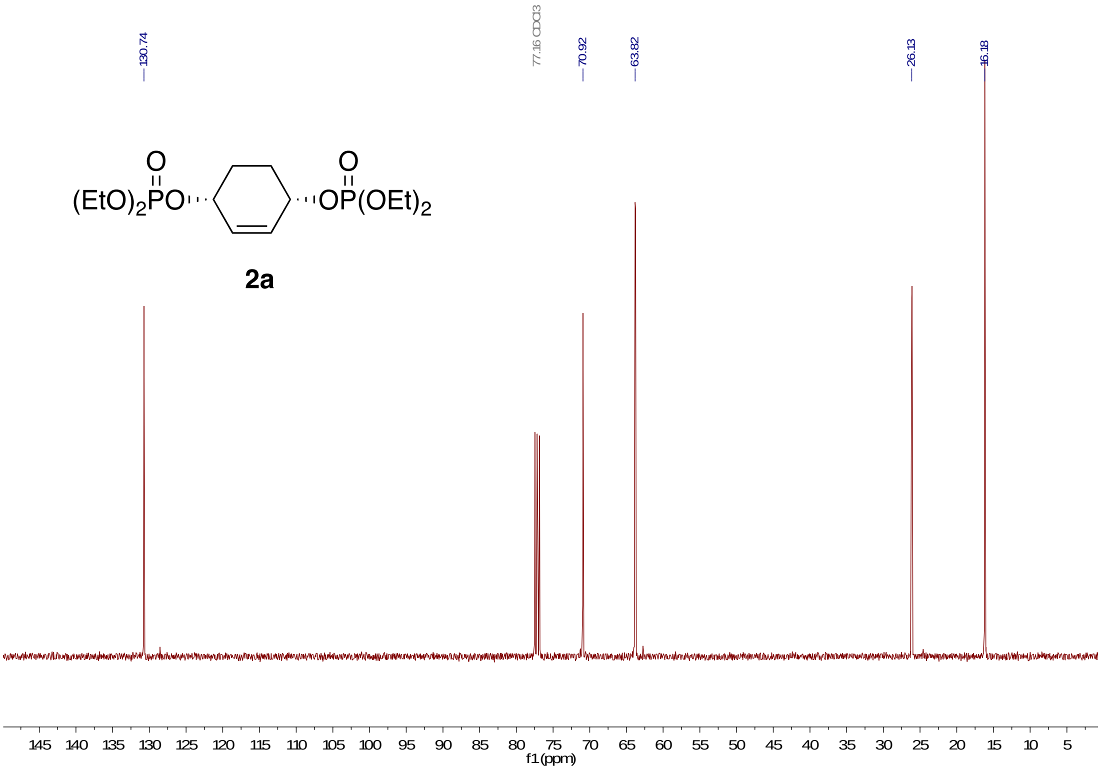

^{31}P NMR (162 MHz, $CDCl_3$)

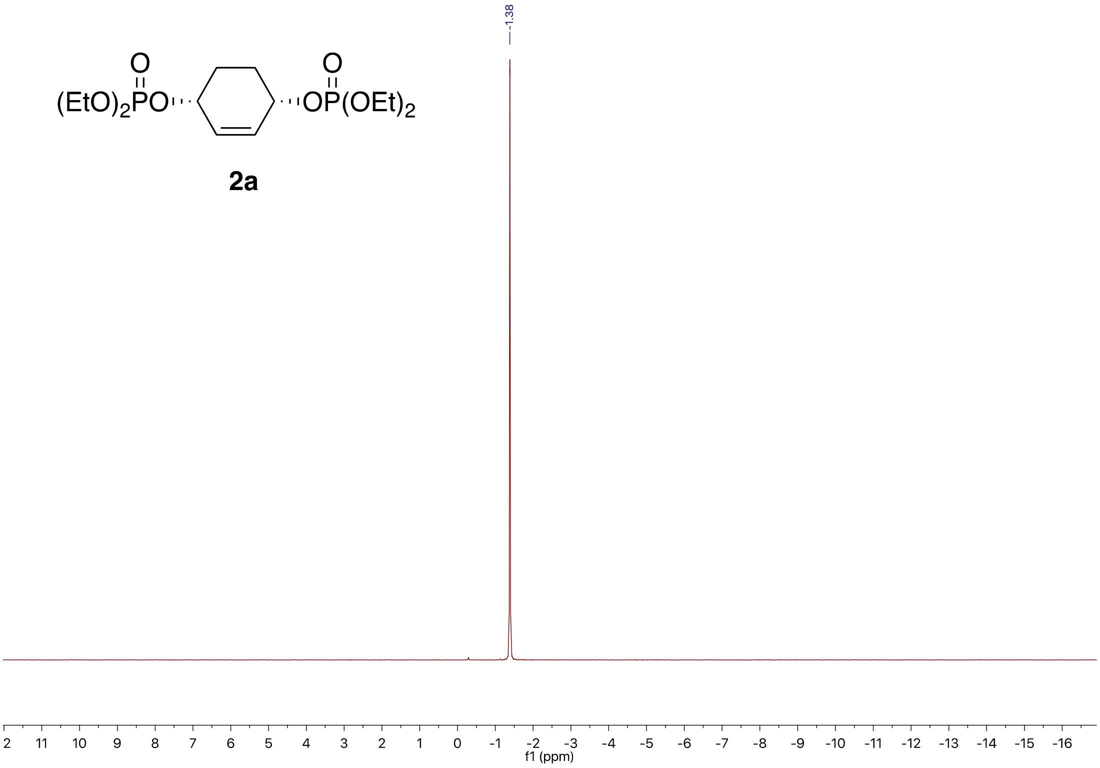

Supplementary Figure 7 - *Meso*-6-cycloheptene-1,5-bisdiethylphosphate (**3a**)
^{1}H NMR (400 MHz, $CDCl_3$)

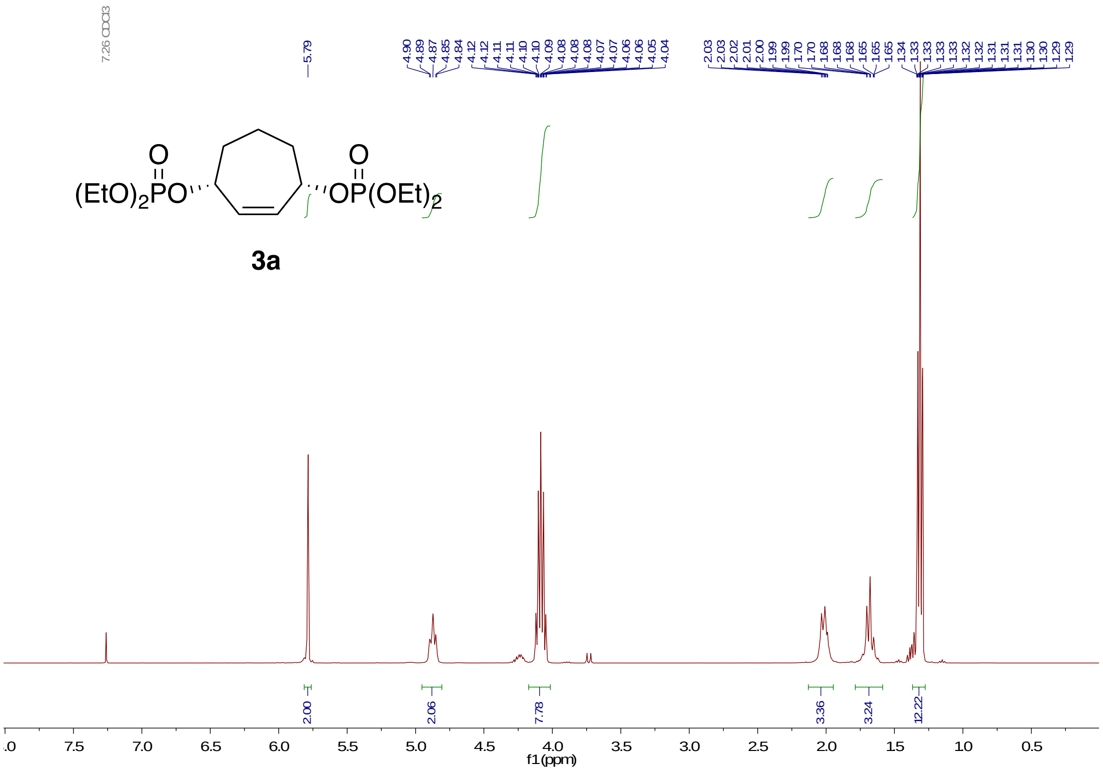

^{13}C NMR (101 MHz, $CDCl_3$)

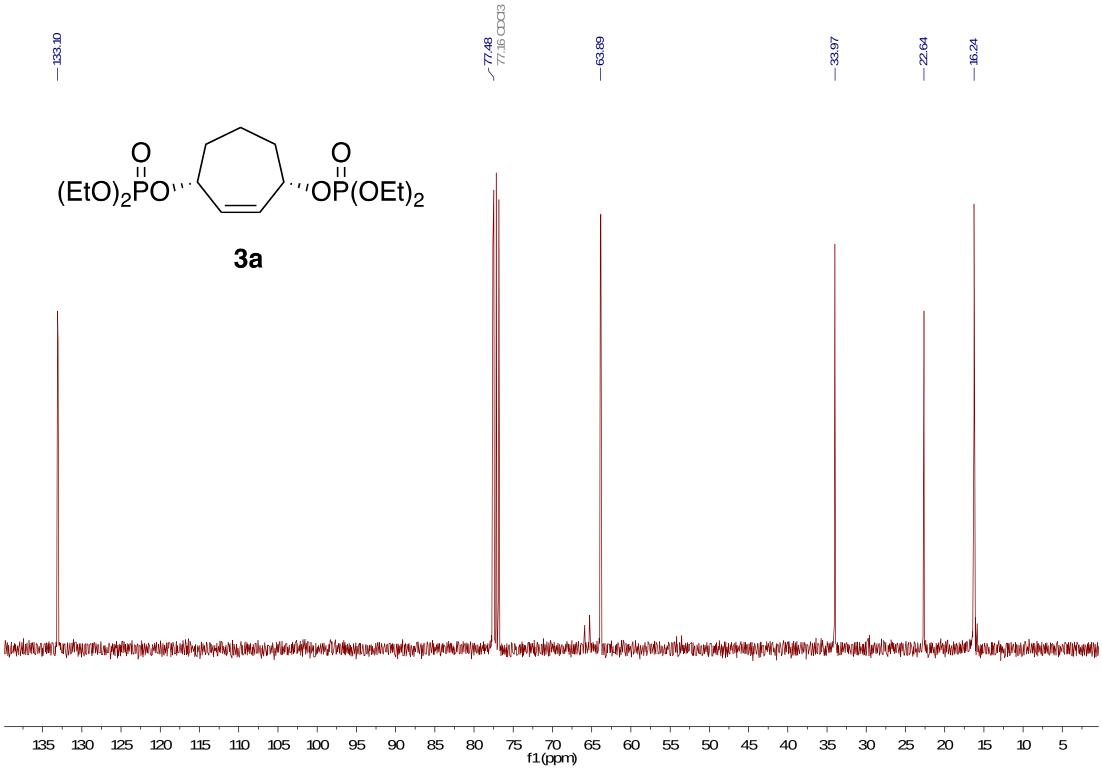

^{31}P NMR (162 MHz, $CDCl_3$)

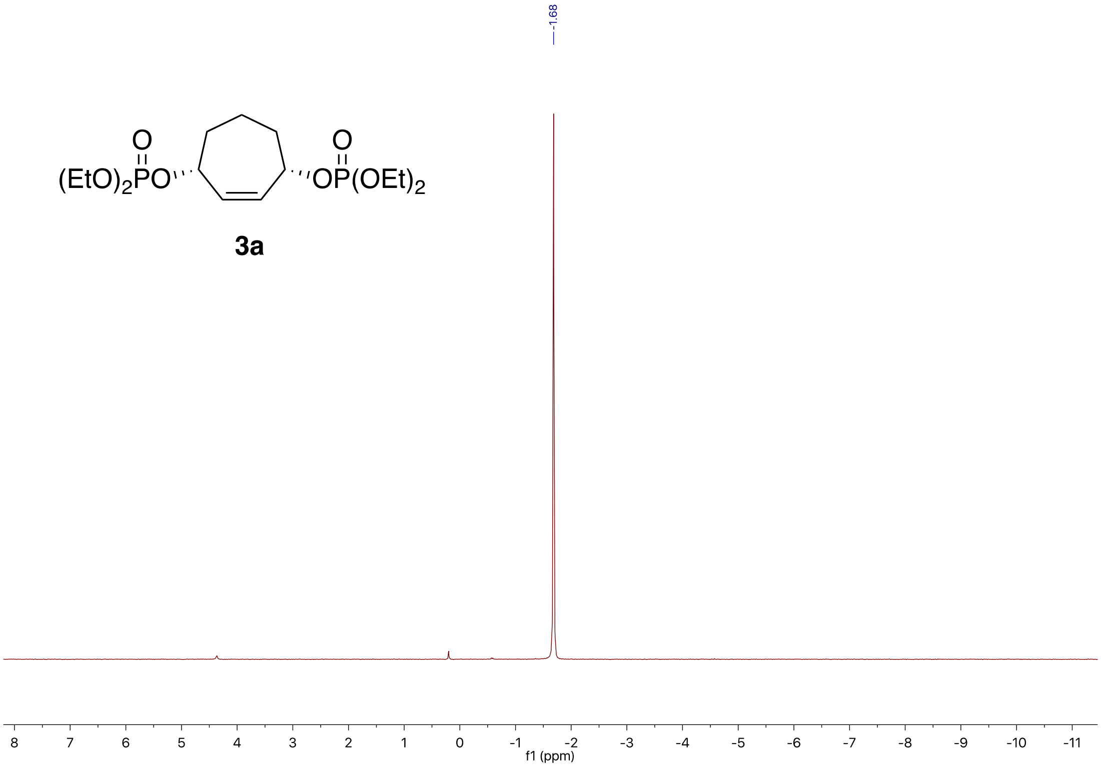

Supplementary Figure 8 - (1*S*,3*S*)-Cyclopent-4-ene-1,3-diyl tetraethyl bis(phosphate) **(13)**

^{1}H NMR (400 MHz, $CDCl_3$)

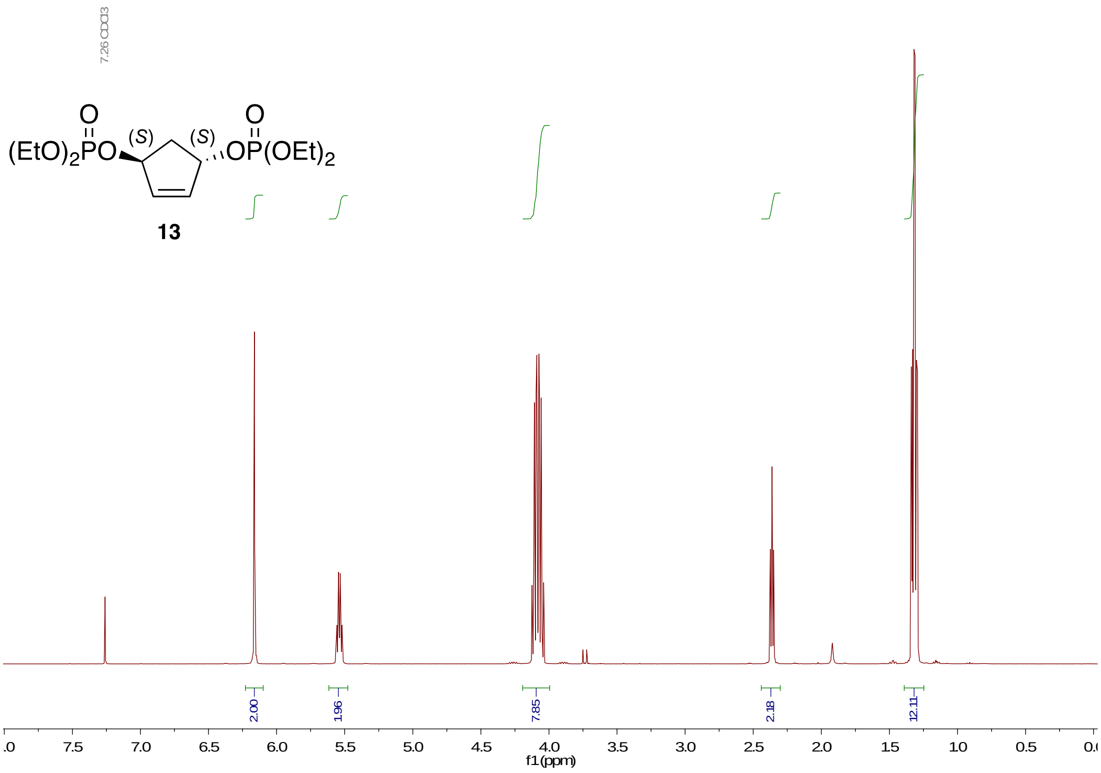

^{13}C NMR (101 MHz, $CDCl_3$)

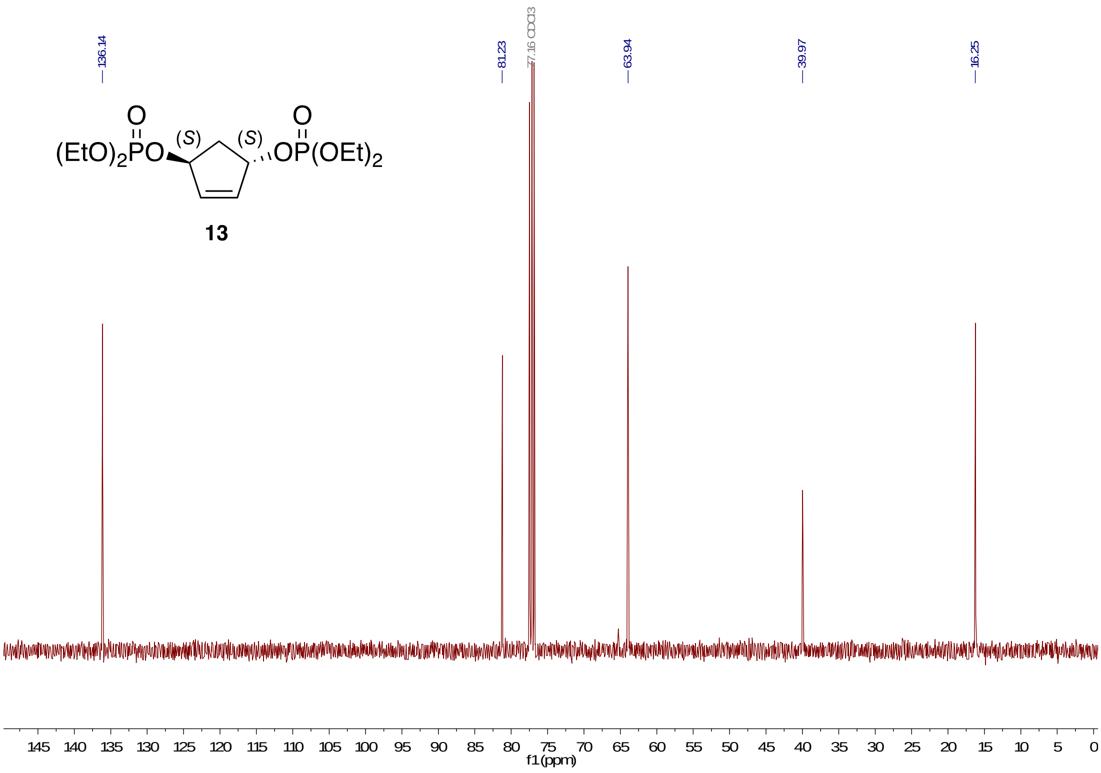

^{31}P NMR (162 MHz, $CDCl_3$)

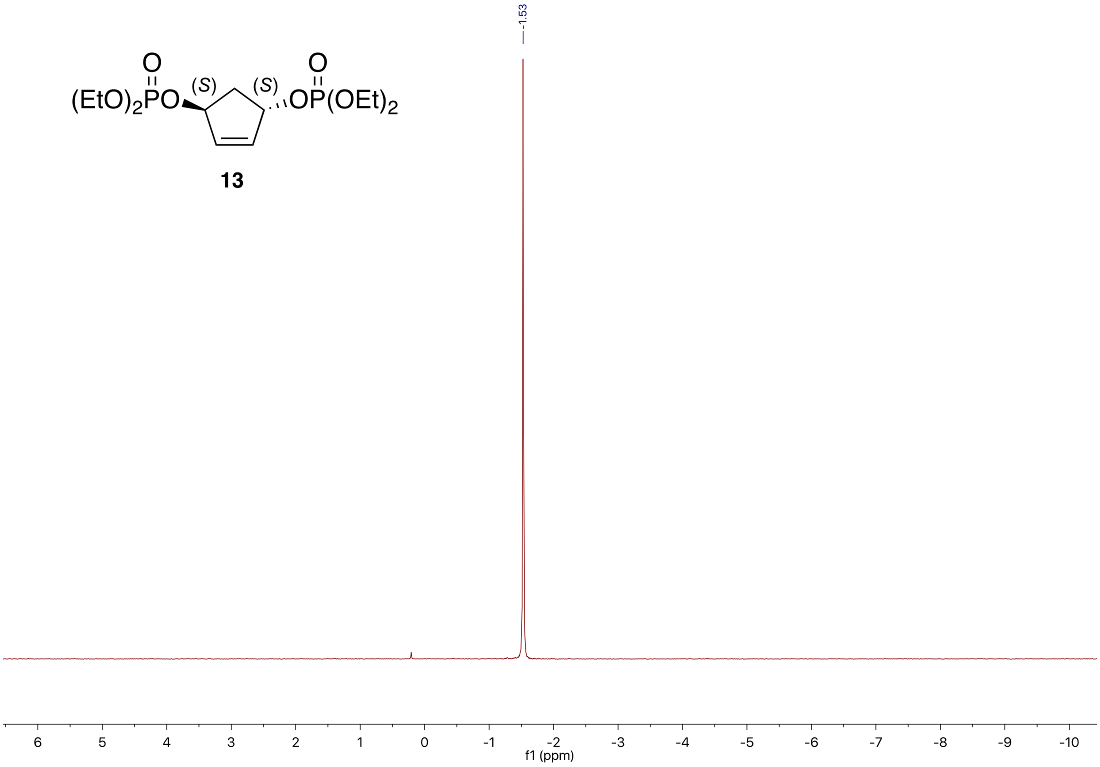

13

Supplementary Figure 9 - (1*R*,2*S*,3*S*)-2-Allyl-2-methylcyclopent-4-ene-1,3-diol

^{1}H NMR (400 MHz, $CDCl_3$)

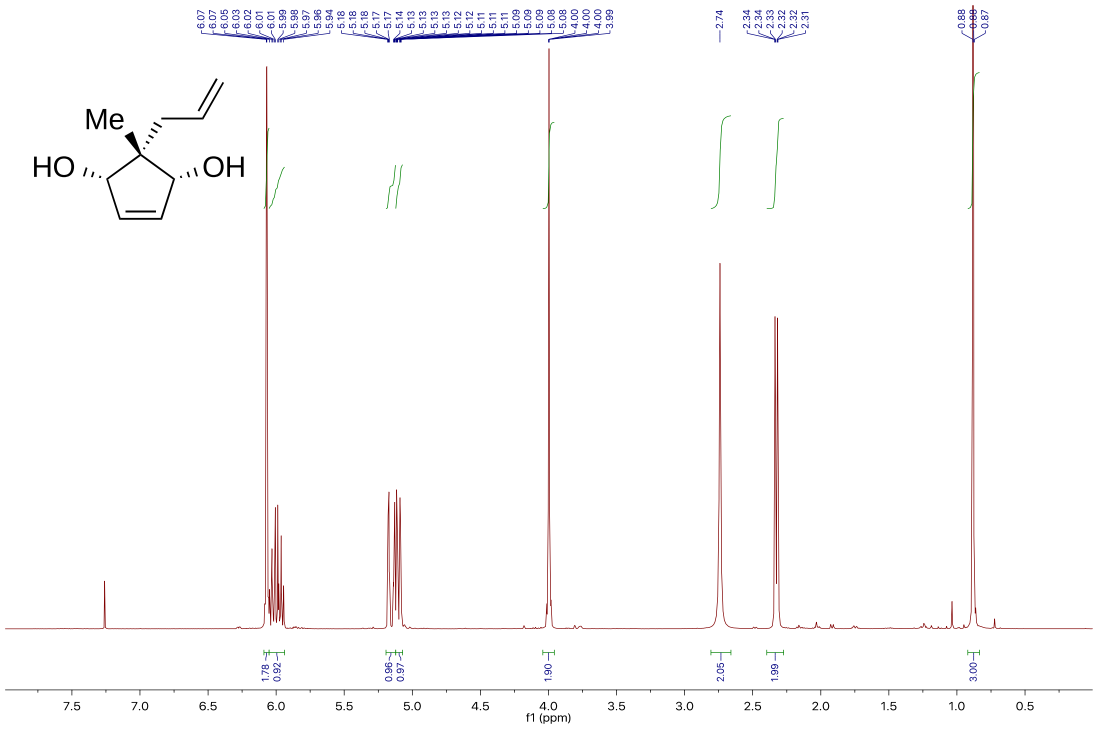

^{13}C NMR (101 MHz, $CDCl_3$)

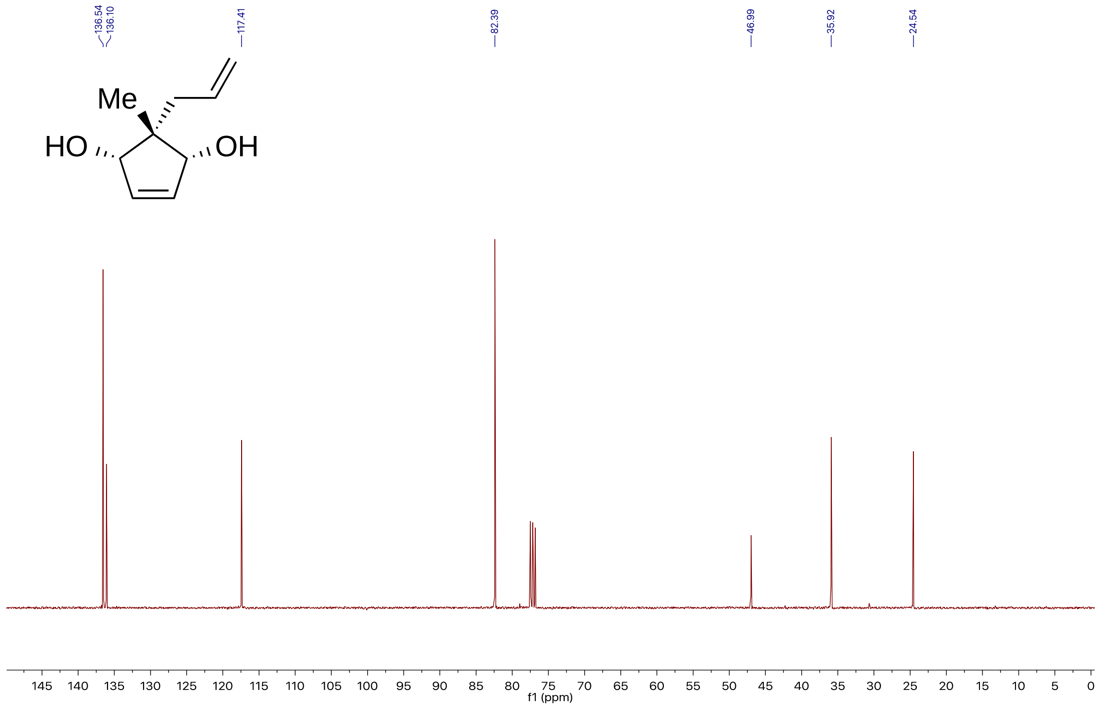

Supplementary Figure 10 - (1*R*,2*S*,3*S*)-2-Allyl-2-methylcyclopent-4-ene-1,3-diol

^{1}H NMR (500 MHz, $CDCl_3$)

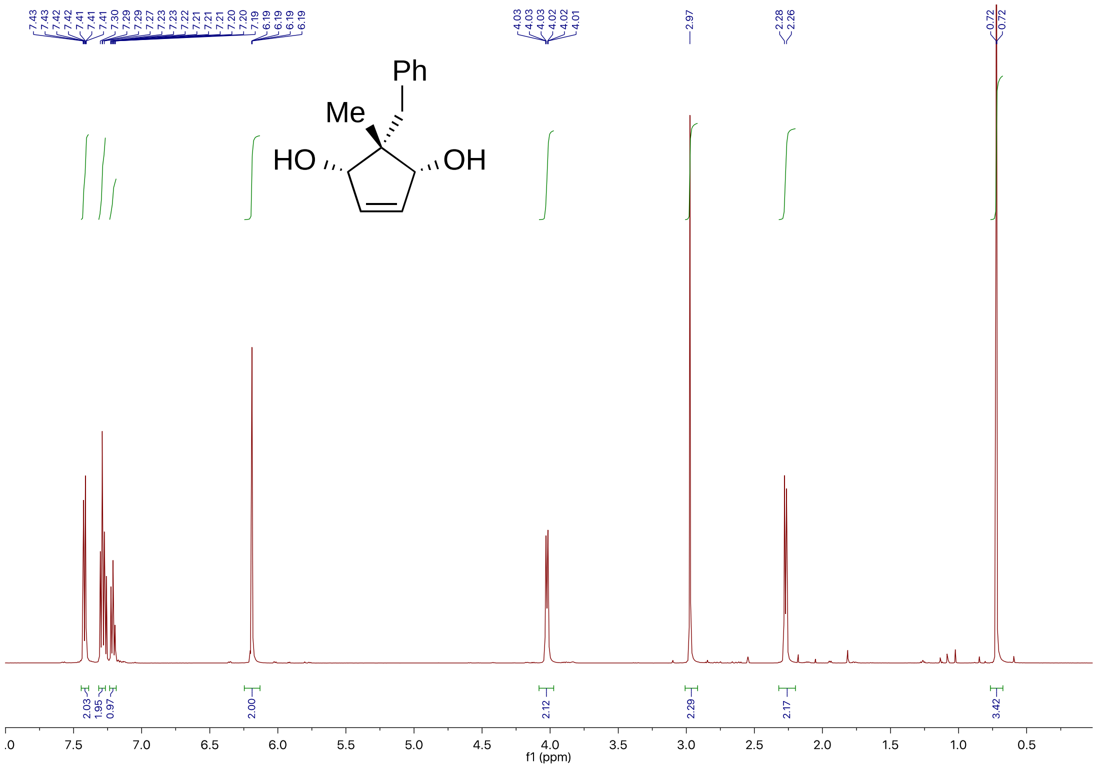

^{13}C NMR (101 MHz, $CDCl_3$)

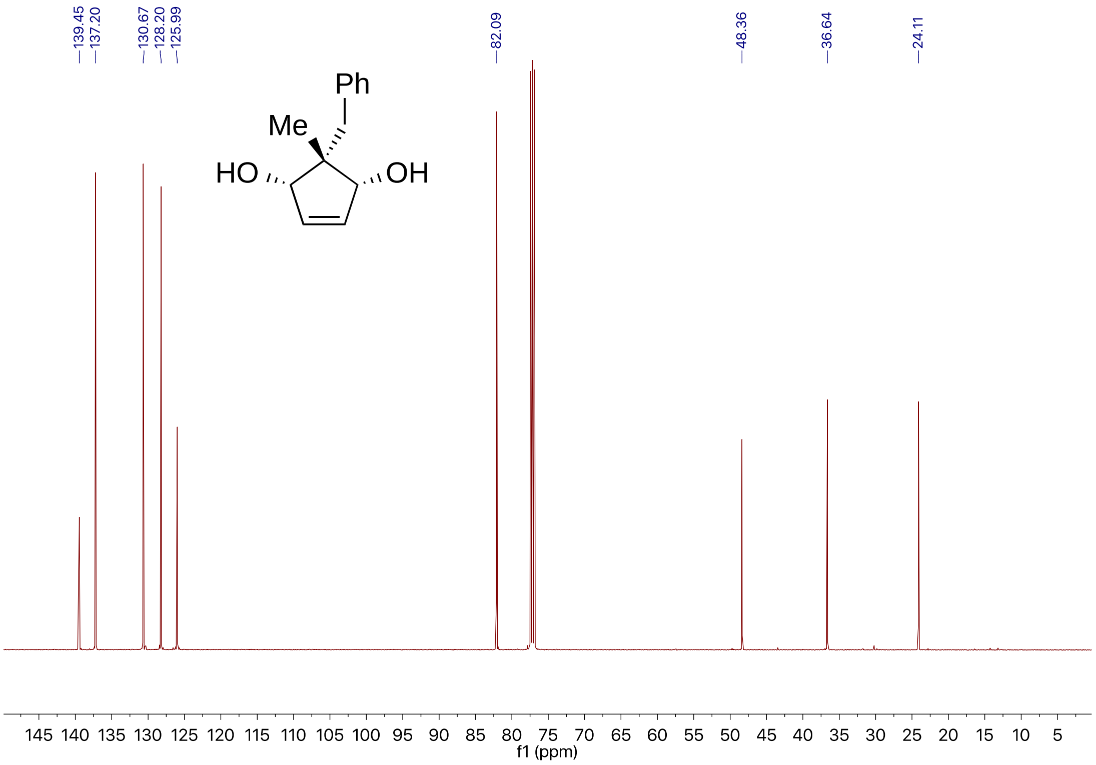

Supplementary Figure 11 - (1*R*,2*S*,3*S*)-2-(2-Chlorobenzyl)-2-methylcyclopent-4-ene-1,3-diol

^{1}H NMR (500 MHz, $CDCl_3$)

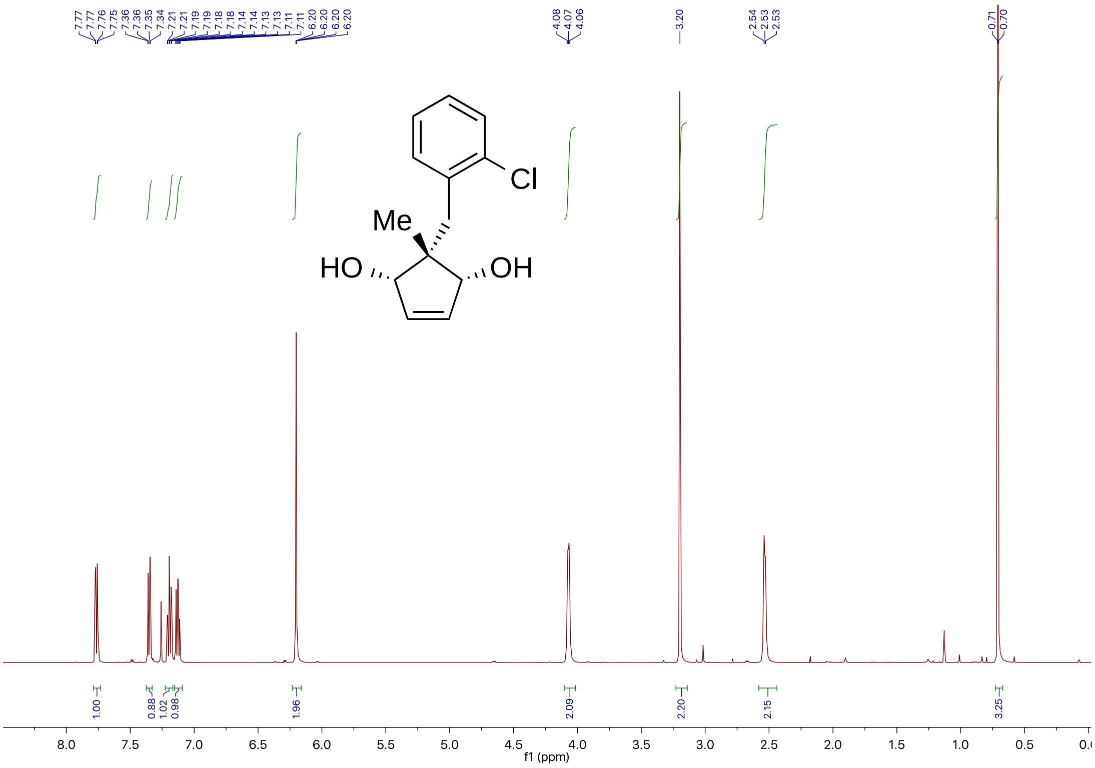

^{13}C NMR (101 MHz, $CDCl_3$)

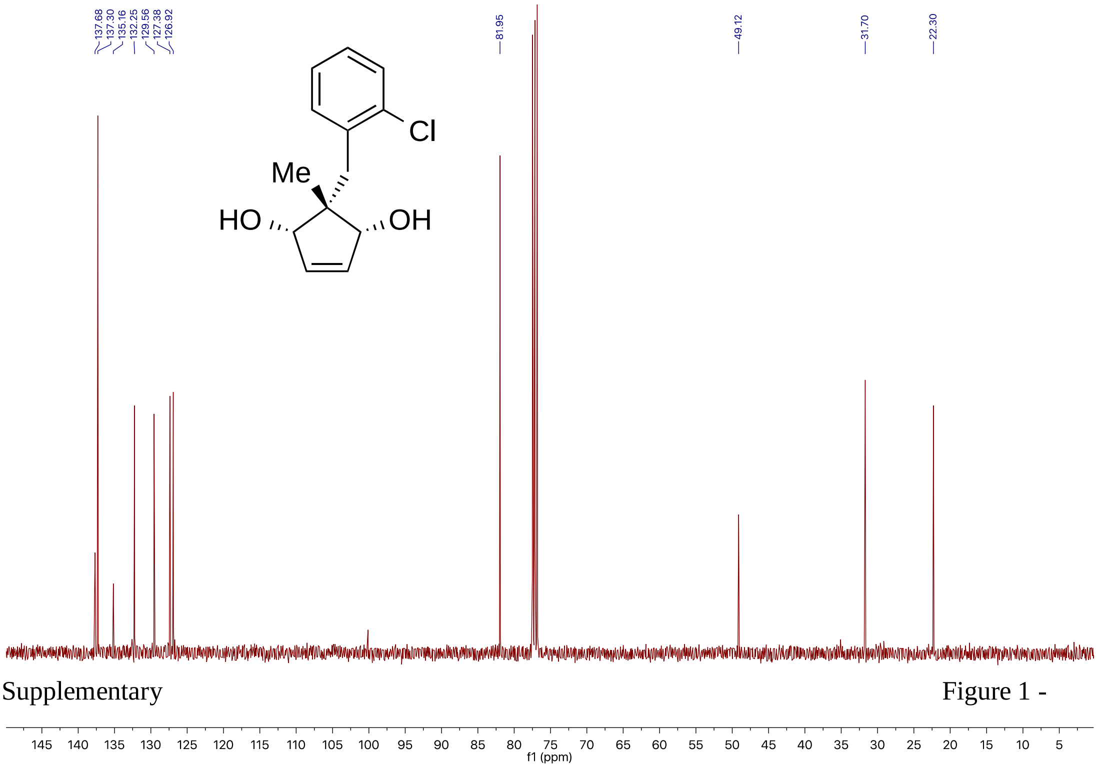

Supplementary Figure 1 -

Supplementary Figure 12 - (1*R*,2*S*,3*S*)-2-((Benzyloxy)methyl)-2-methylcyclopent-4-ene-1,3-diol ^{1}H NMR (500 MHz, $CDCl_3$)

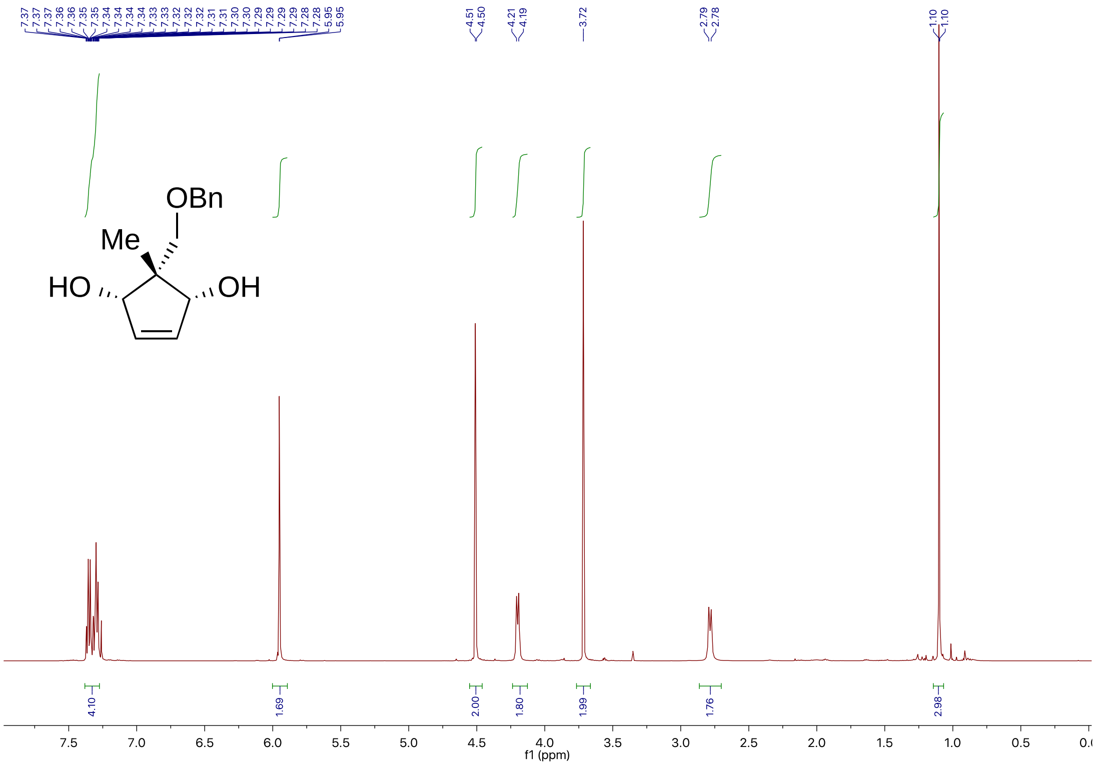

^{13}C NMR (101 MHz, $CDCl_3$)

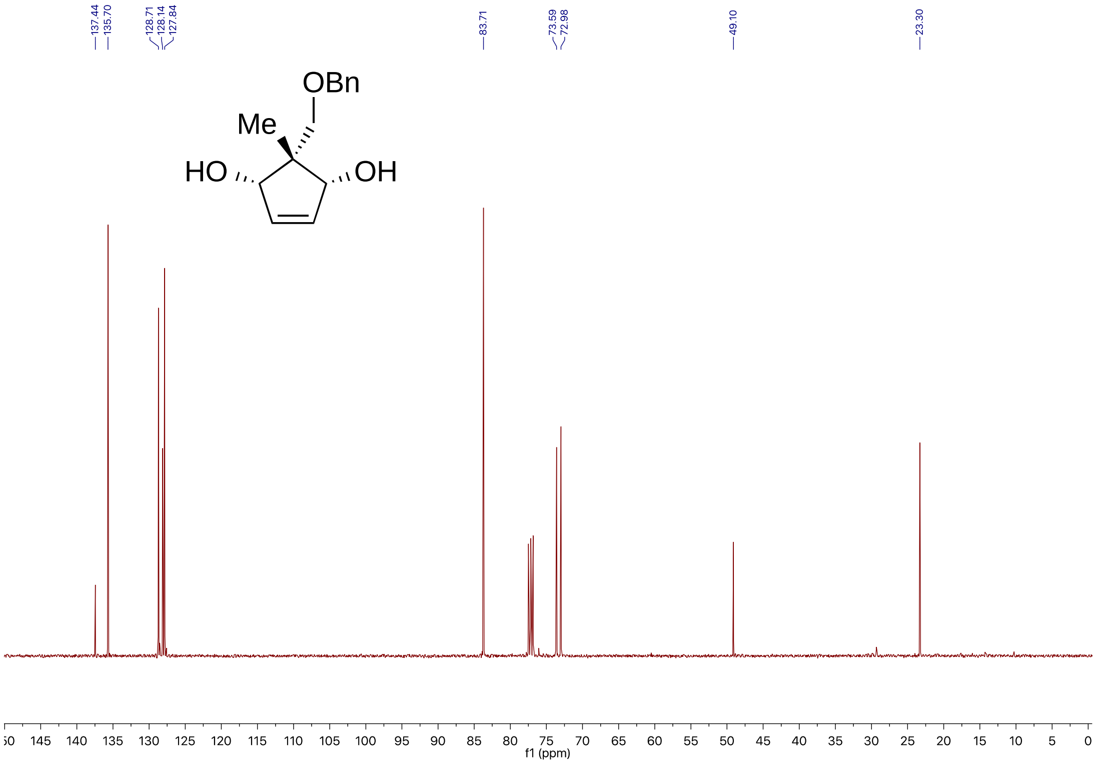

Supplementary Figure 13 - (1*S*,2*R*,5*S*)-2',3'-Dihydrospiro[cyclopentane-1,1'-inden]-3-ene-2,5-diol ^{1}H NMR (500 MHz, $CDCl_3$)

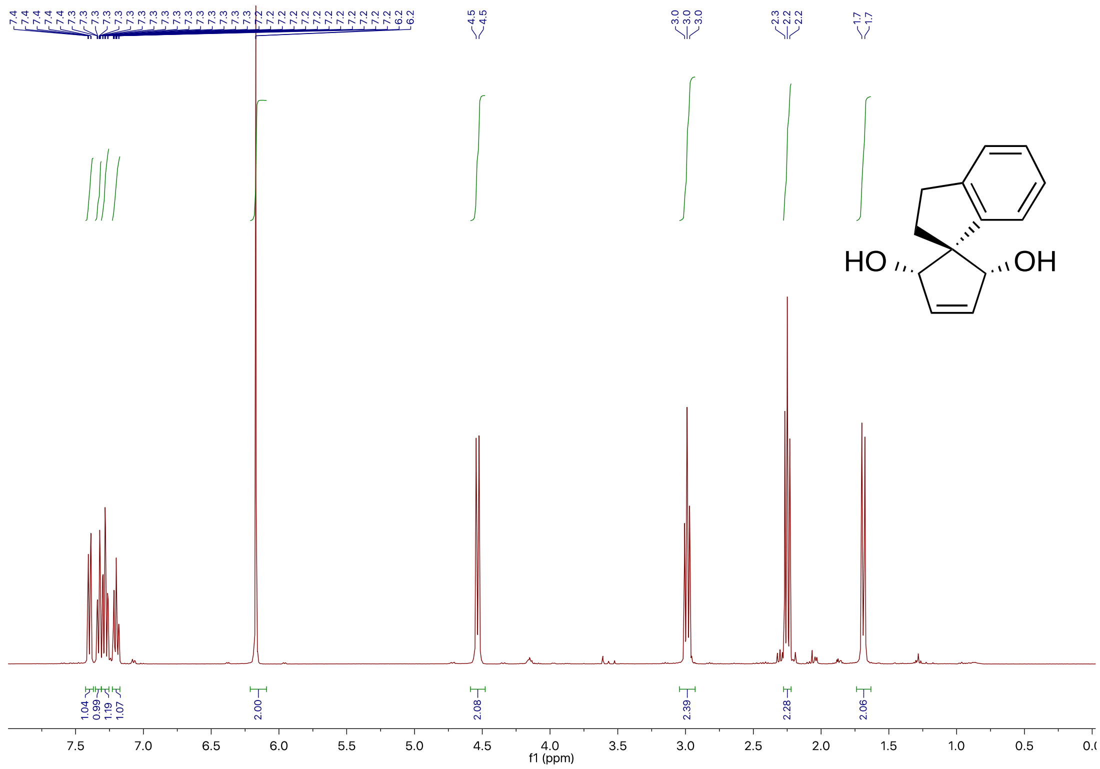

^{13}C NMR (101 MHz, $CDCl_3$)

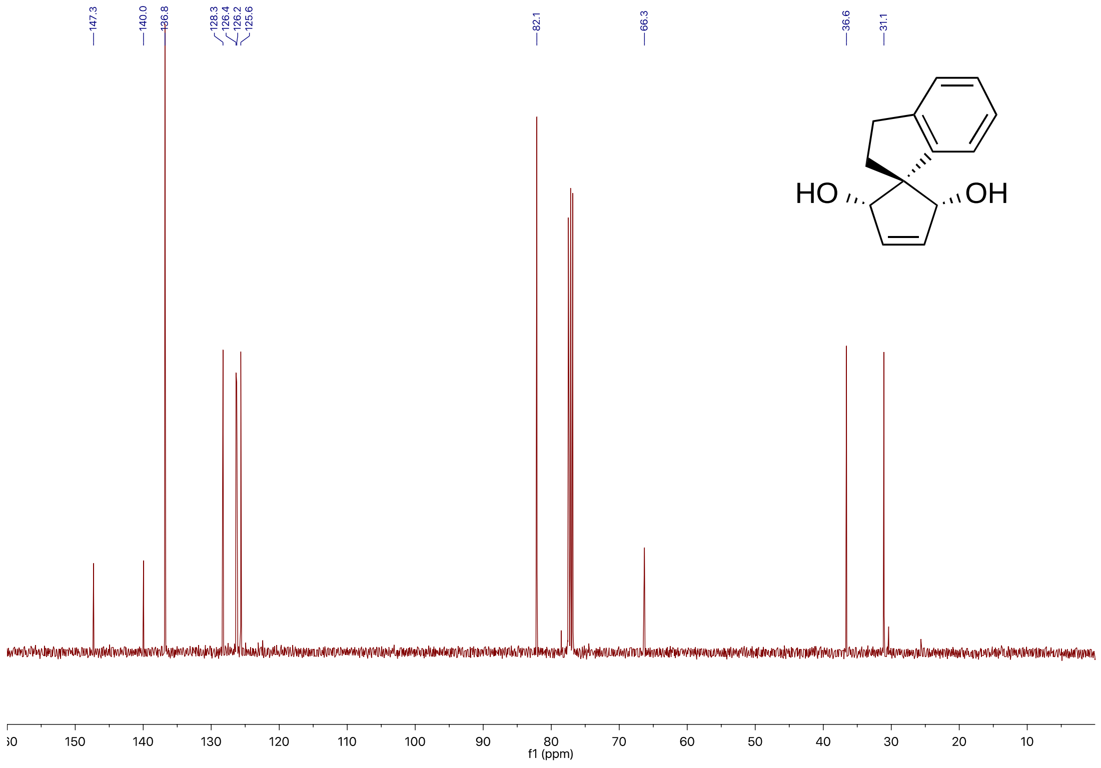

Supplementary Figure 14 - (2'*R*,4*S*,5'*S*)-Spiro[chromane-4,1'-cyclopentan]-3'-ene-2',5'-diol ^{1}H NMR (500 MHz, $CDCl_3$)

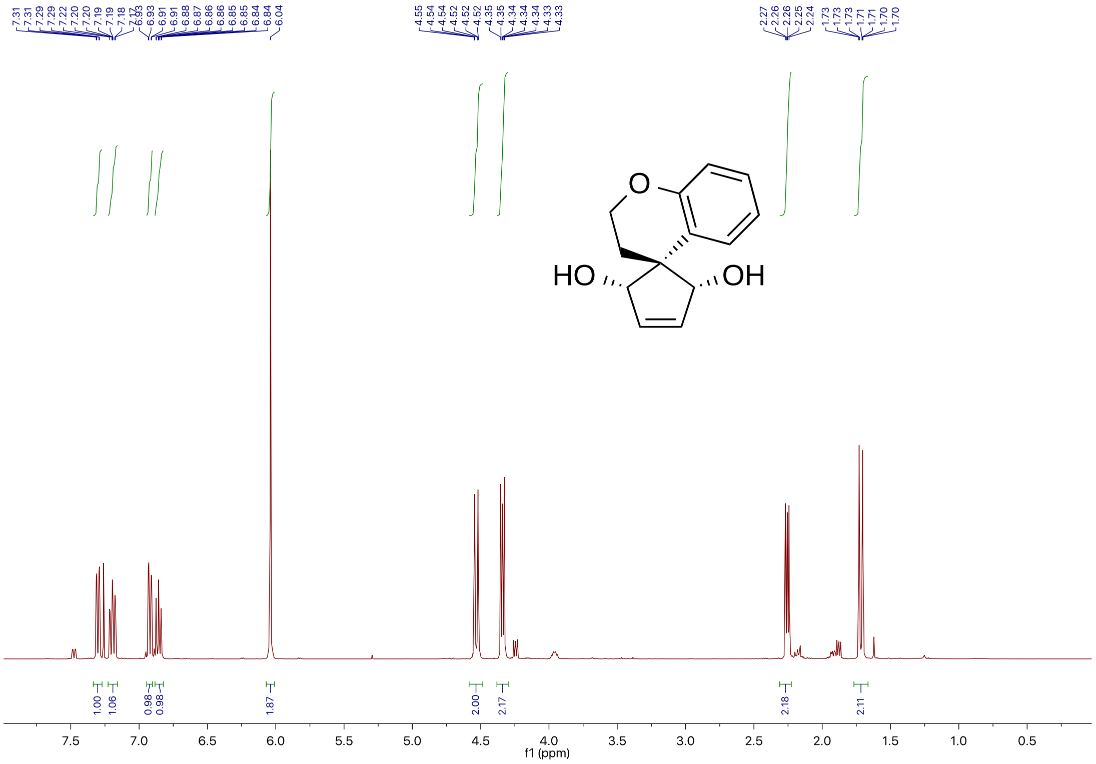

^{13}C NMR (101 MHz, $CDCl_3$)

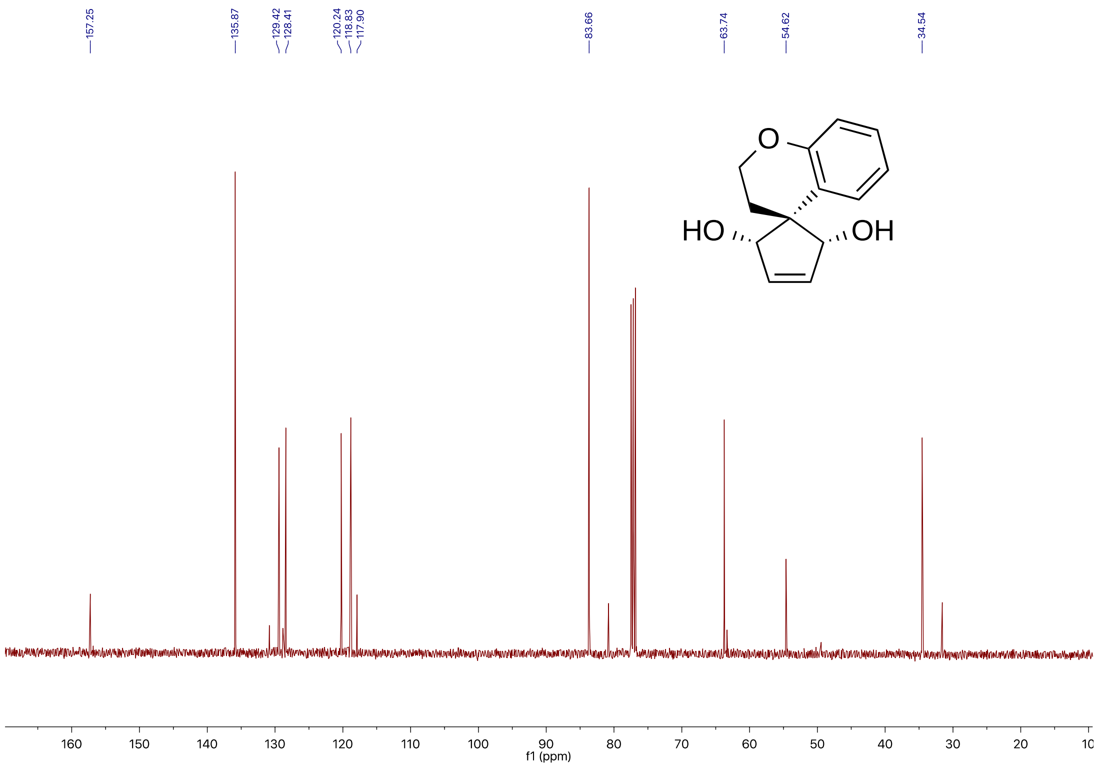

Supplementary Figure 15 - (1*R*,2*S*,3*S*)-2-Benzyl-2-methylcyclopent-4-ene-1,3-bis(diethyl phosphate) (**4a**) ^{1}H NMR (400 MHz, $CDCl_3$)

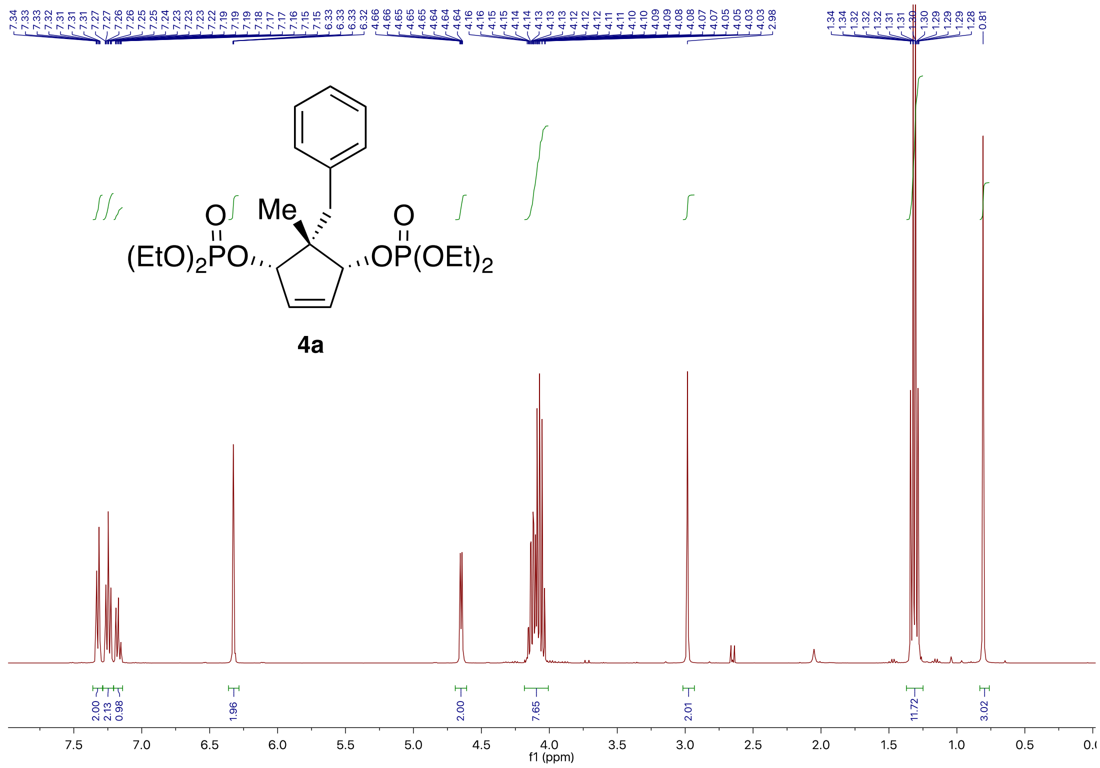

^{13}C NMR (101 MHz, $CDCl_3$)

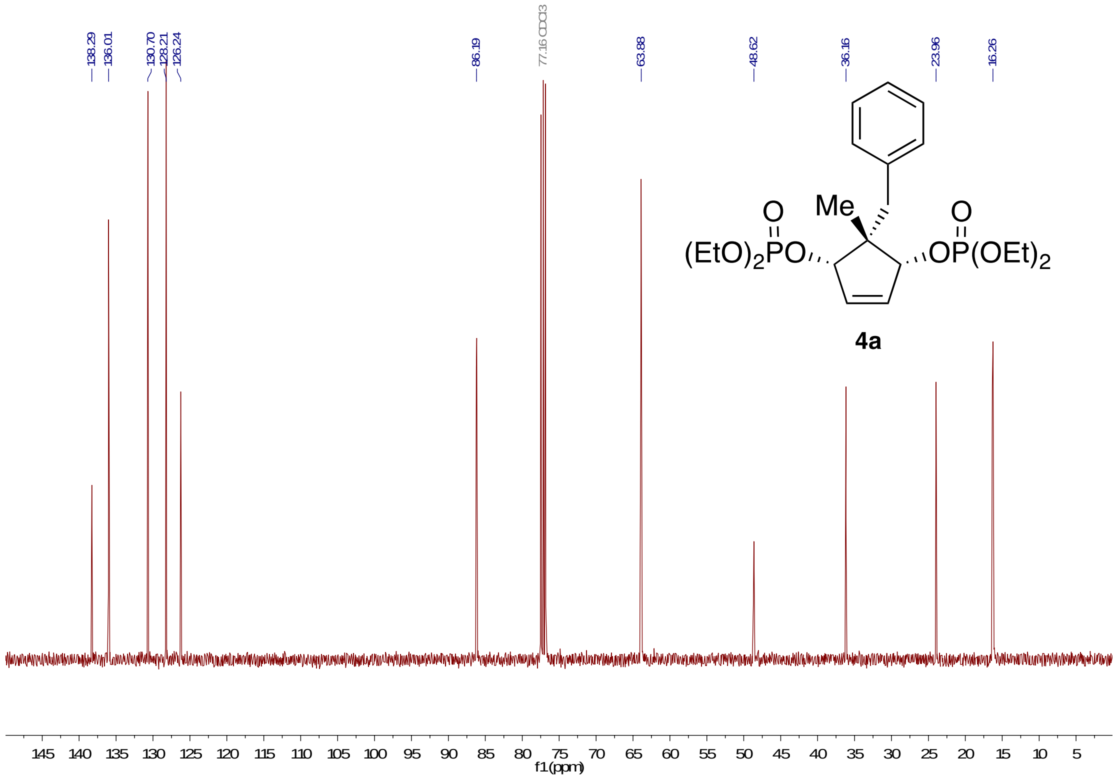

^{31}P NMR (162 MHz, $CDCl_3$)

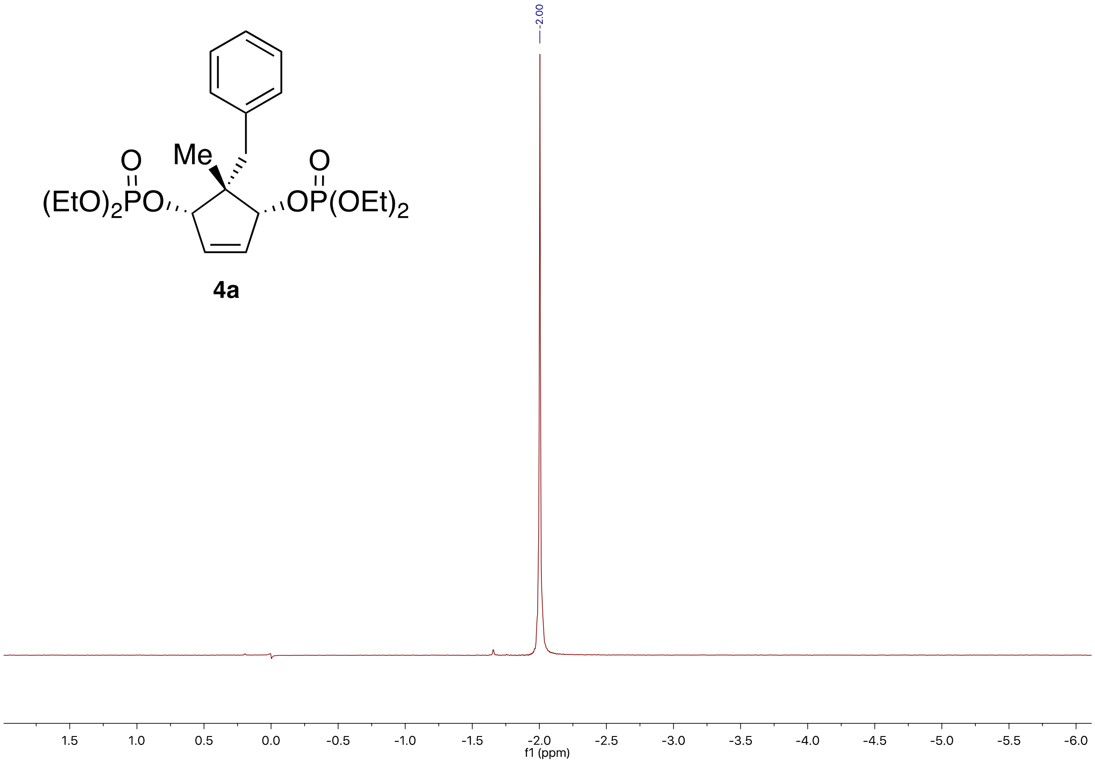

Supplementary Figure 16 - *Cis*-2,2-dimethylcyclopent-4-ene-1,3-bis(diethyl phosphate) (**5a**) ^{1}H NMR (400 MHz, $CDCl_3$)

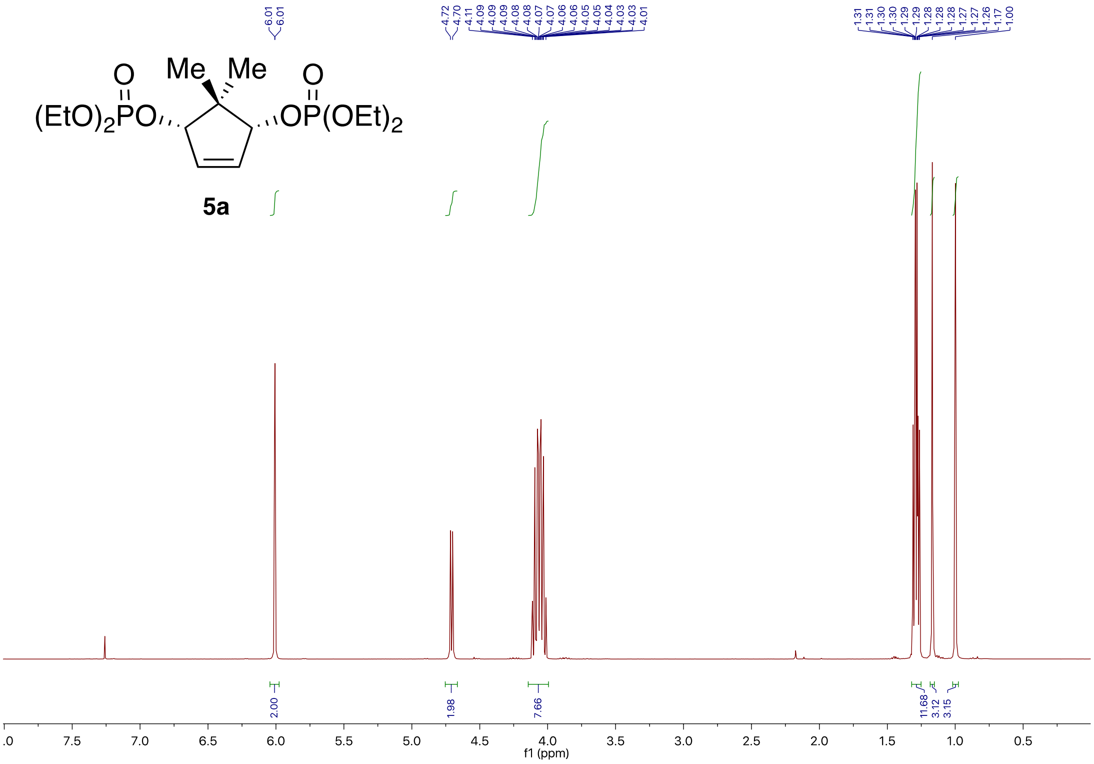

^{13}C NMR (101 MHz, $CDCl_3$)

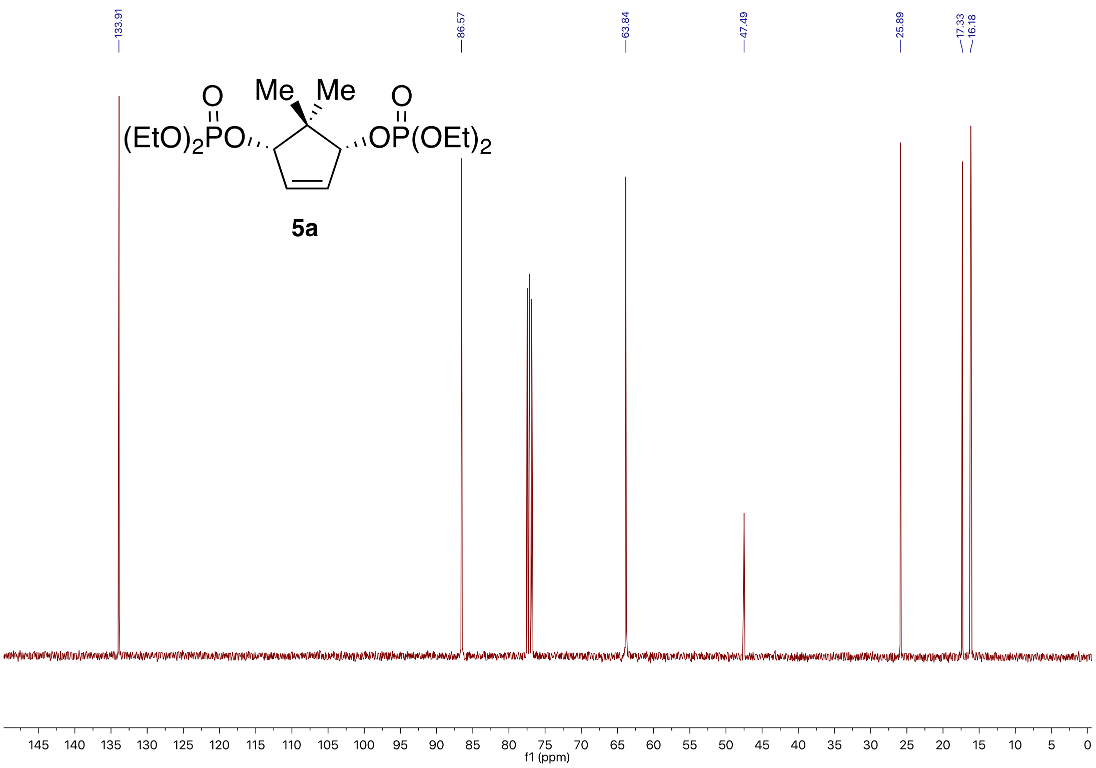

^{31}P NMR (162 MHz, $CDCl_3$)

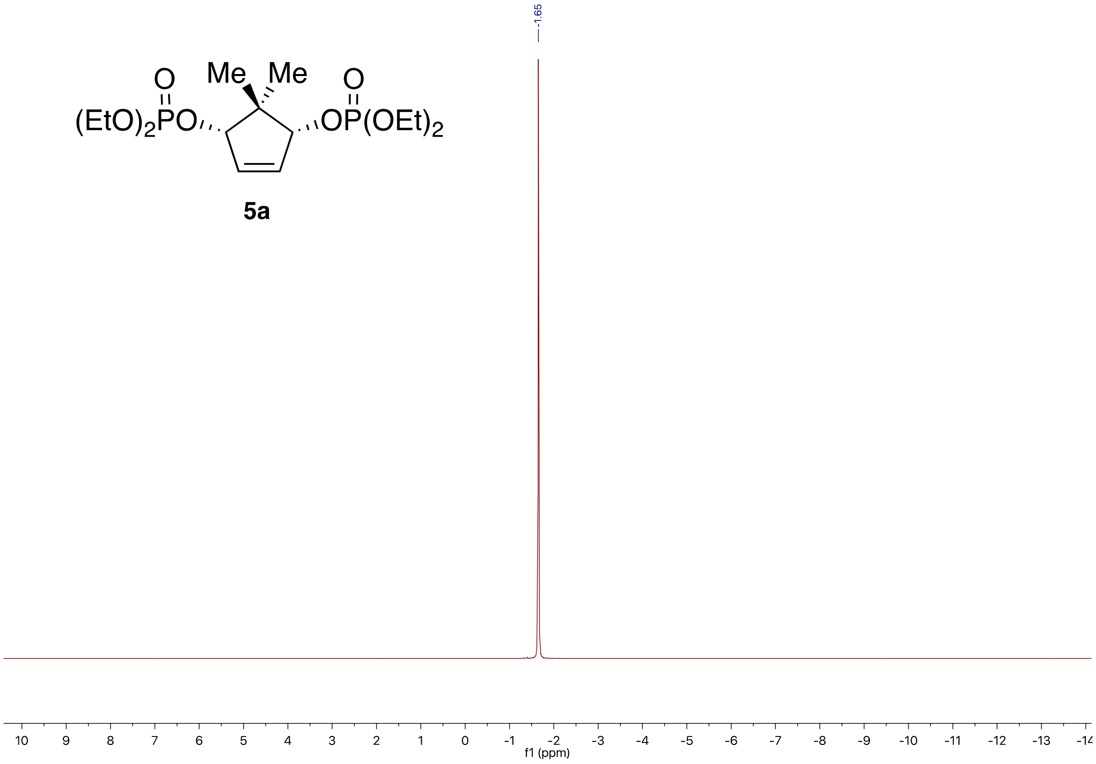

Supplementary Figure 17 - (1*R*,2*S*,3*S*)-2-Allyl-2-methylcyclopent-4-ene-1,3-bis(diethyl phosphate) (**6a**) ^{1}H NMR (400 MHz, $CDCl_3$)

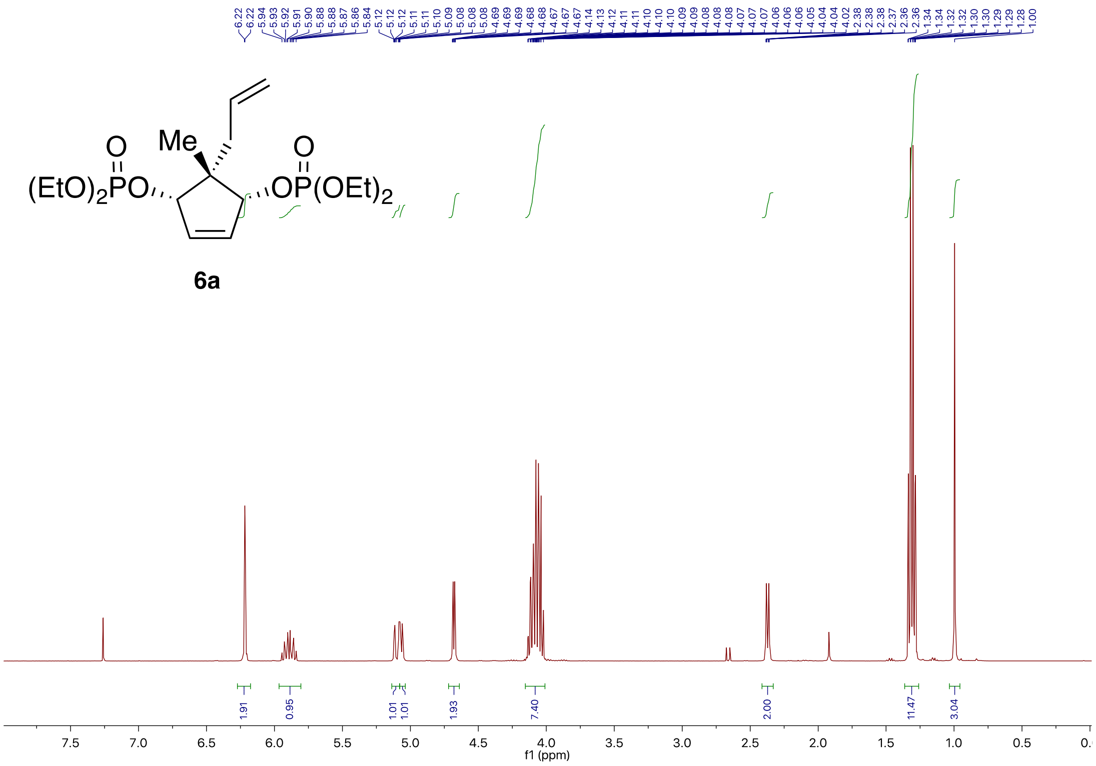

^{13}C NMR (101 MHz, $CDCl_3$)

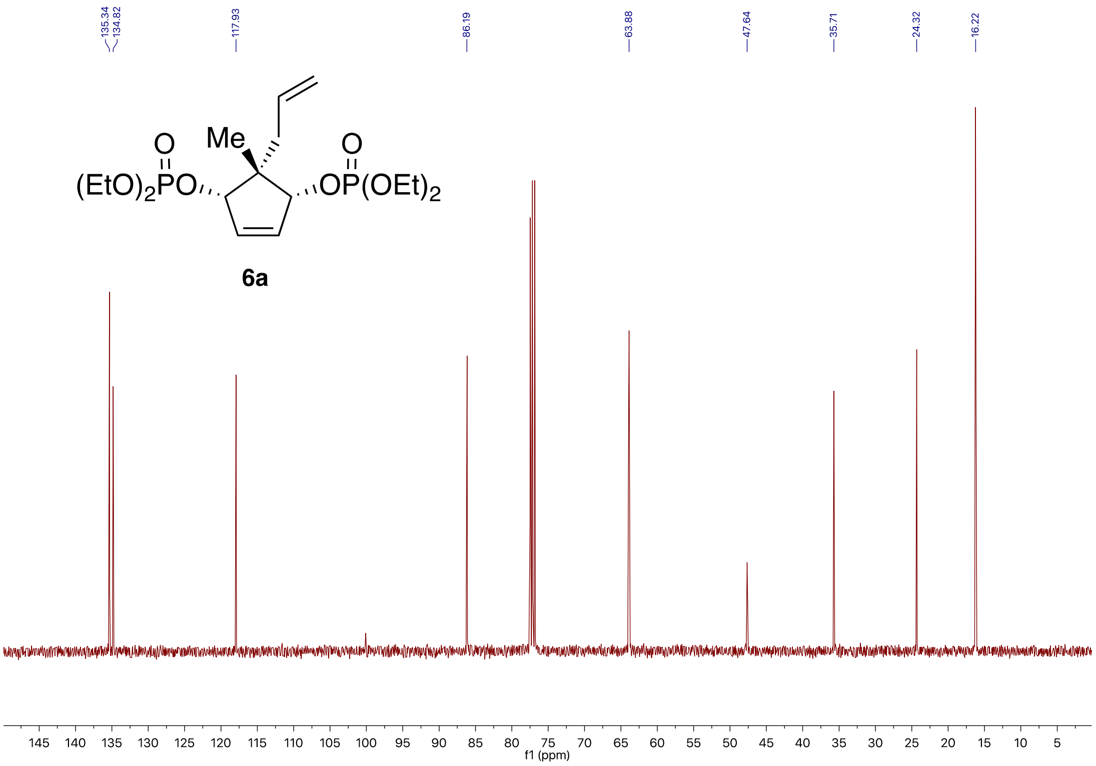

^{31}P NMR (162 MHz, CDCl$_3$)

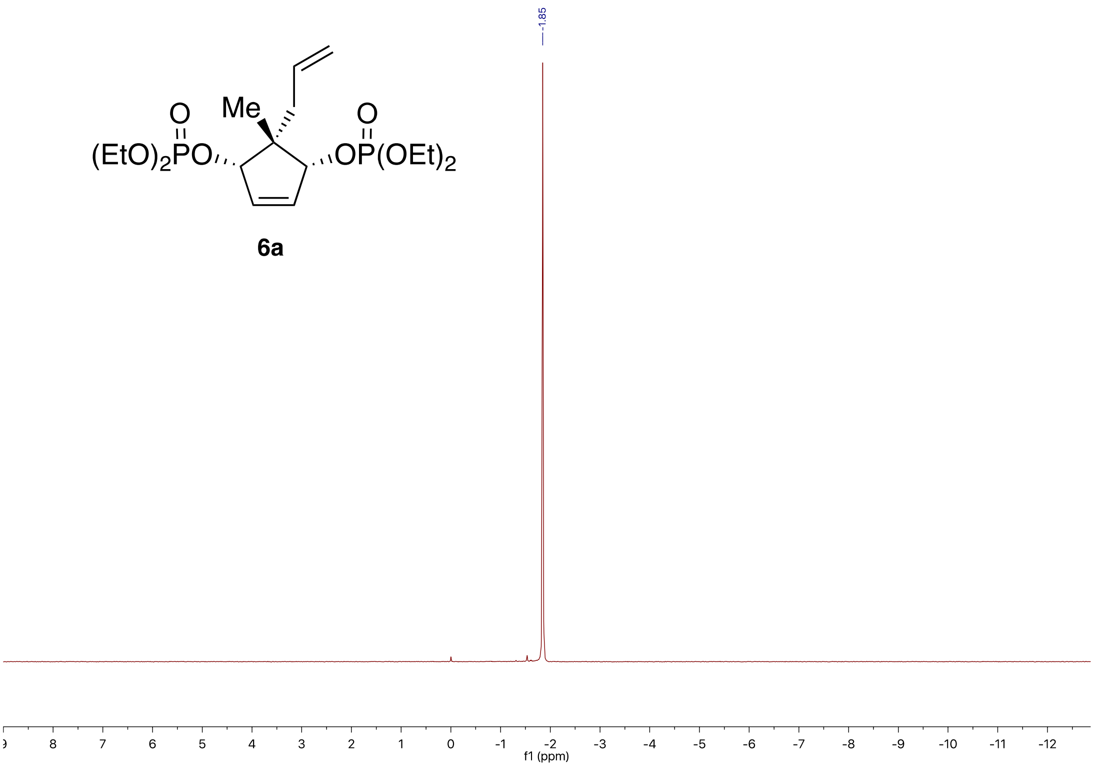

Supplementary Figure 18 - (1*R*,2*S*,3*S*)-2-(2-Chlorobenzyl)-2-methylcyclopent-4-ene-1,3-bis(diethyl phosphate) (**7a**) ^{1}H NMR (400 MHz, CDCl$_3$)

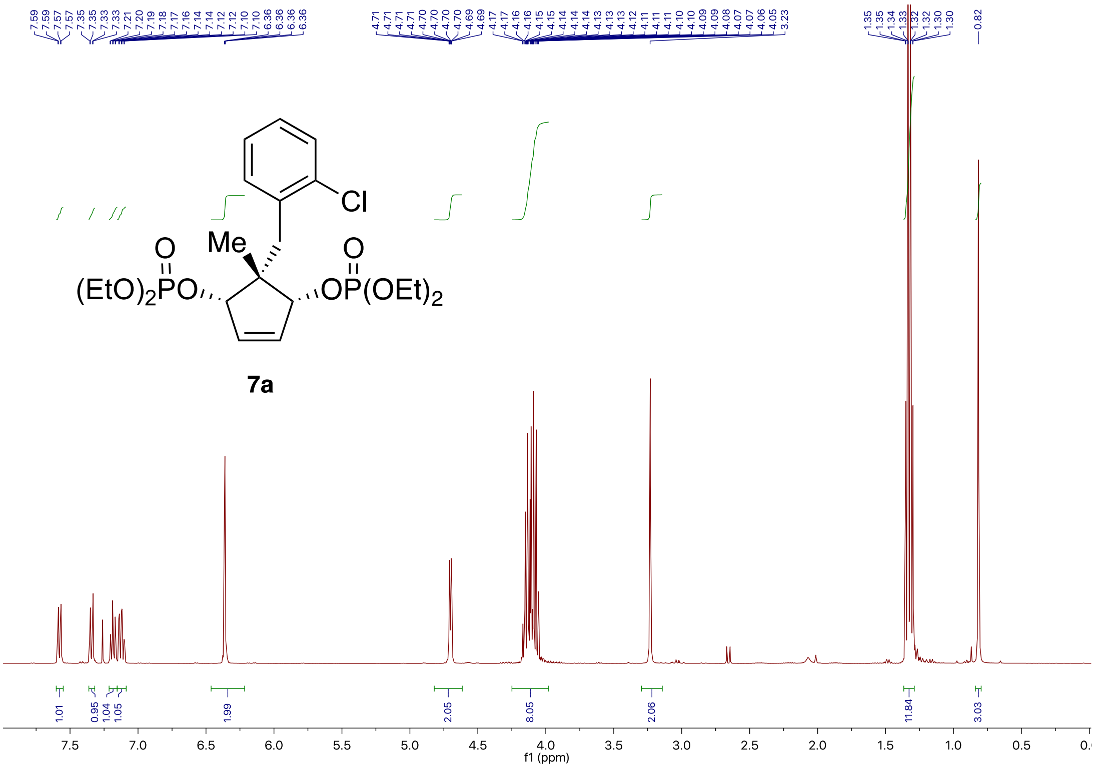

^{13}C NMR (101 MHz, $CDCl_3$)

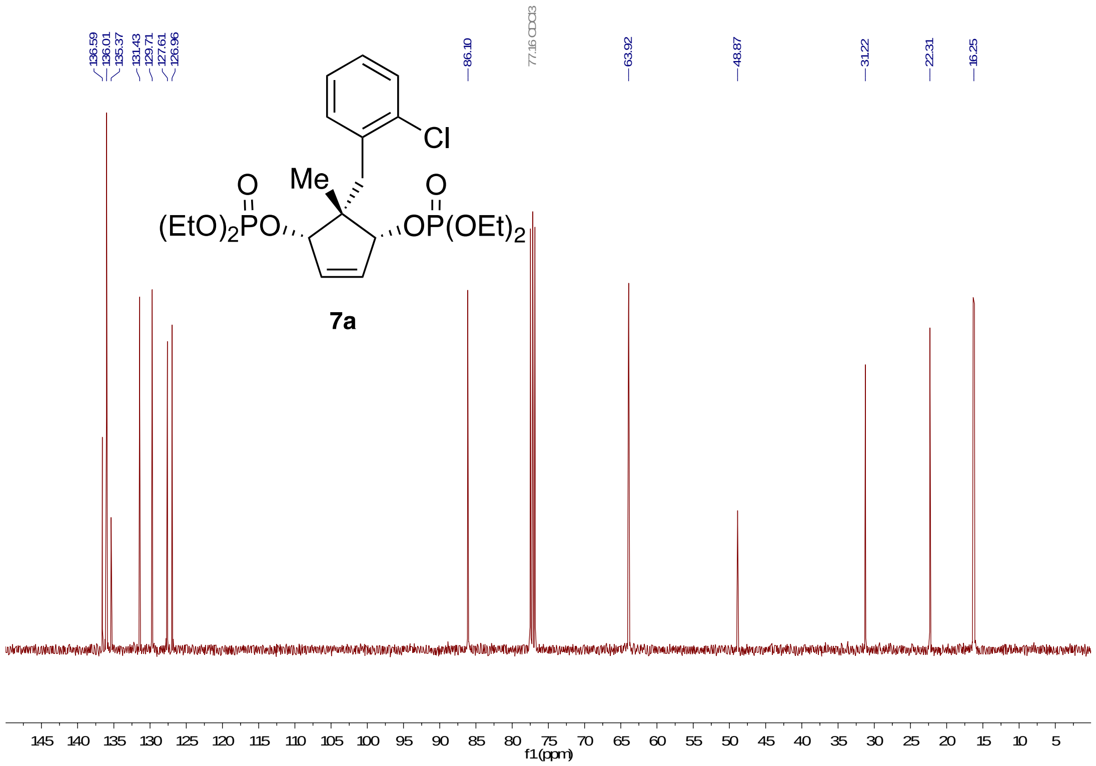

^{31}P NMR (162 MHz, $CDCl_3$)

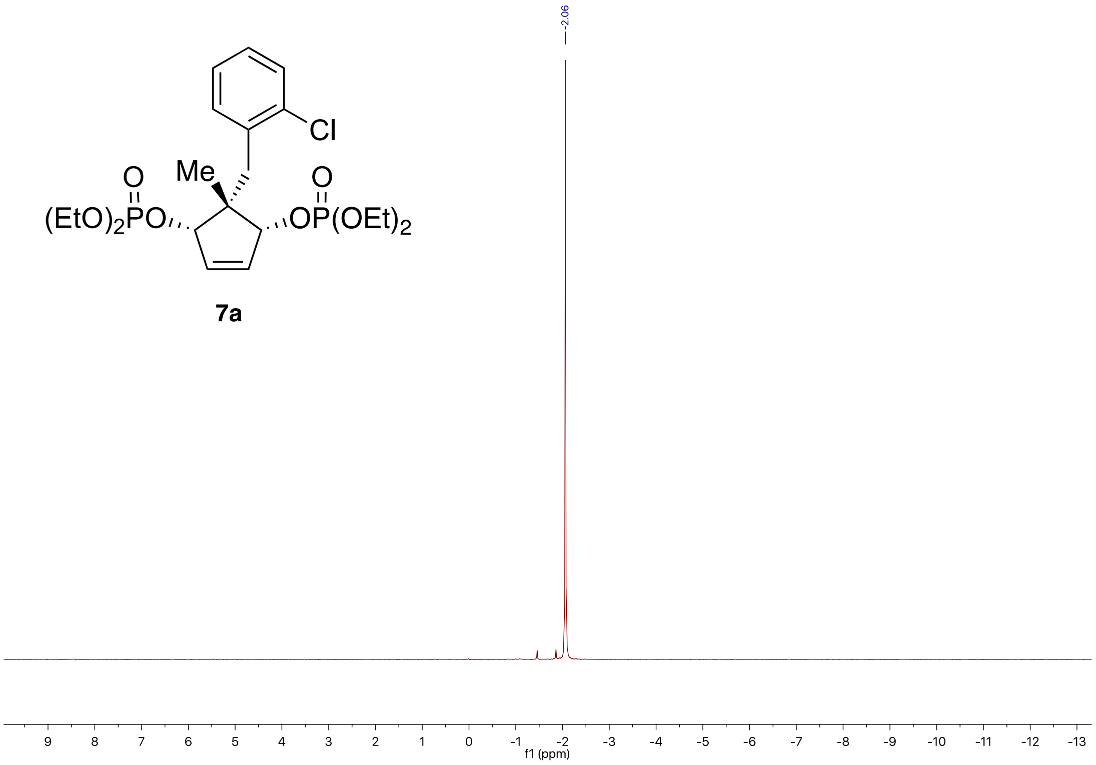

Supplementary Figure 19 - (1*R*,2*S*,3*S*)-2-((Benzyloxy)methyl)-2-methylcyclopent-4-ene-1,3-bis(diethyl phosphate) (**8a**) ^{1}H NMR (400 MHz, $CDCl_3$)

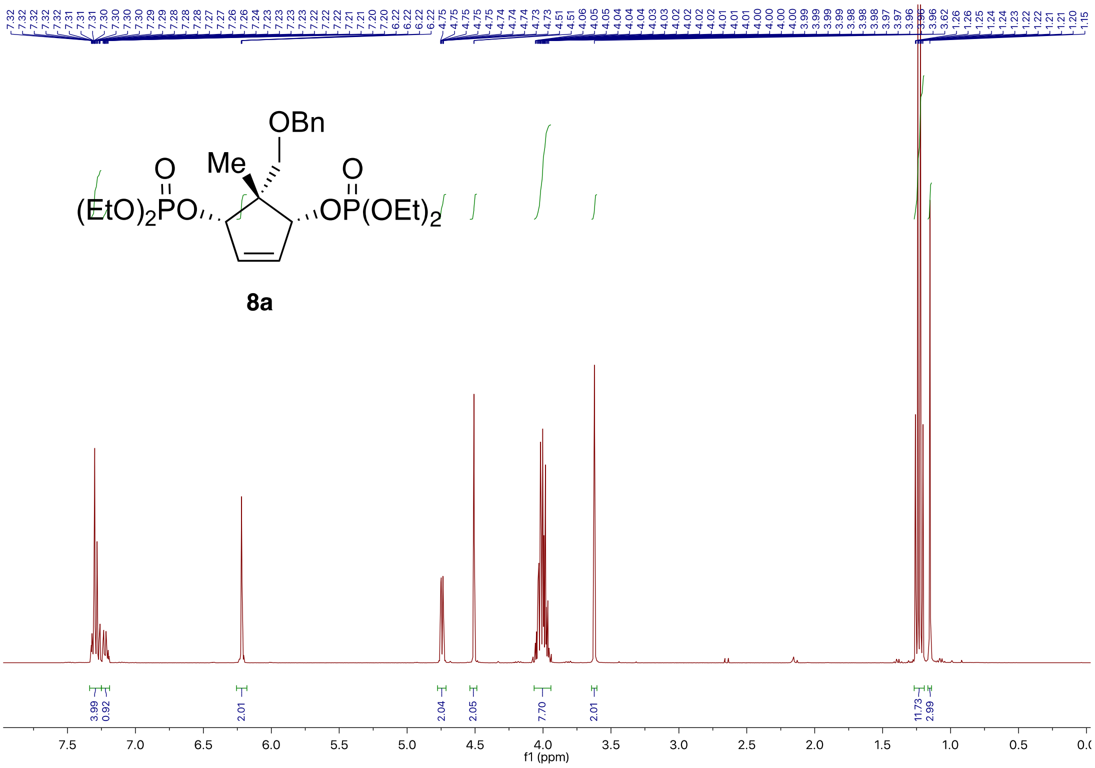

^{13}C NMR (101 MHz, $CDCl_3$)

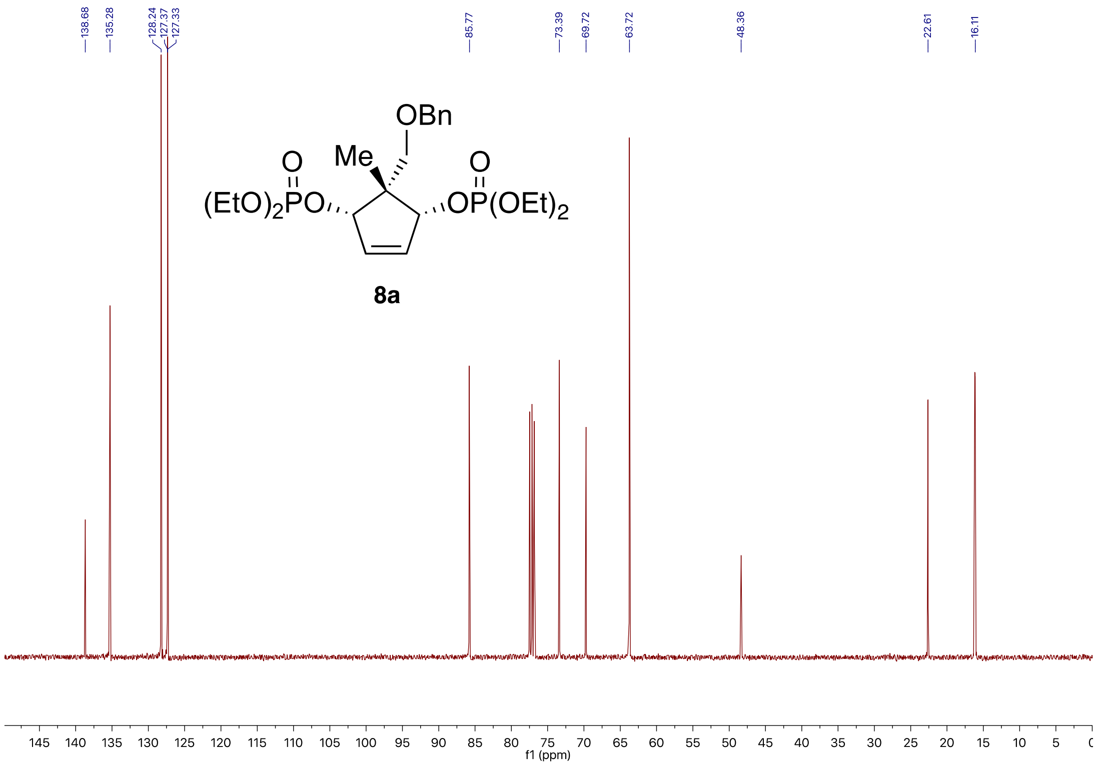

^{31}P NMR (162 MHz, CDCl$_3$)

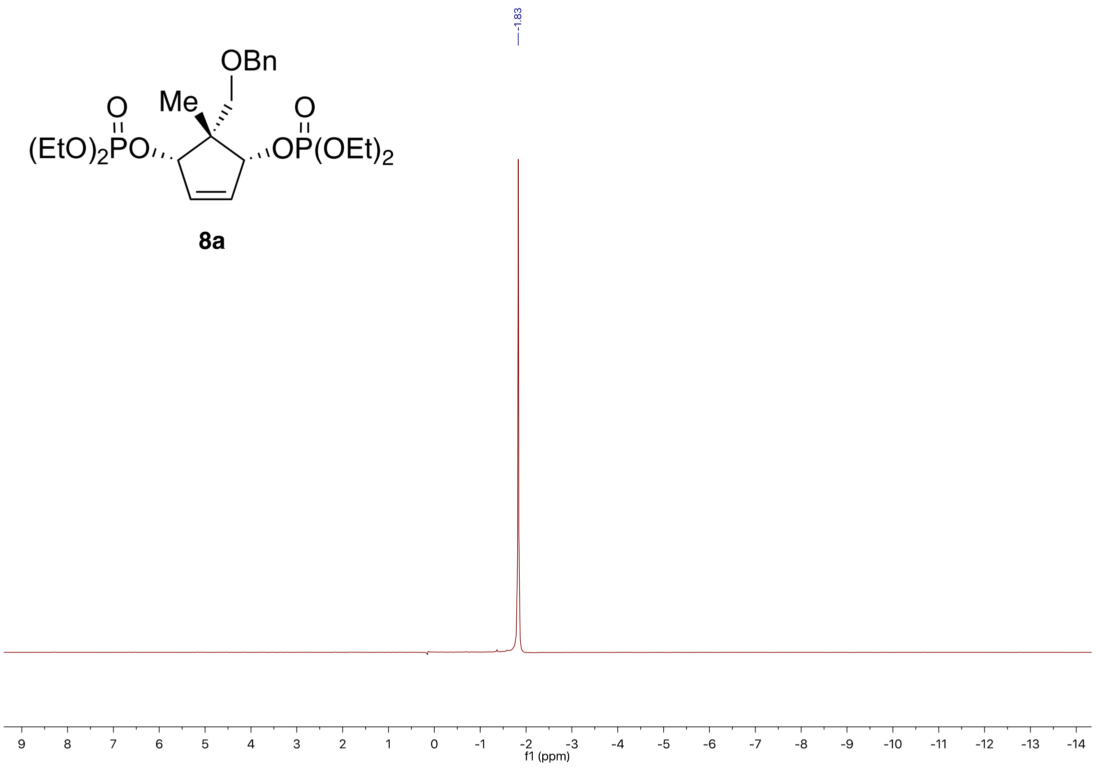

Supplementary Figure 20 - (1*S*,2*R*,5*S*)-2',3'-Dihydrospiro[cyclopentane-1,1'-inden]-3-ene-2,5-bis(diethyl phosphate) (**9a**) ^{1}H NMR (400 MHz, CDCl$_3$)

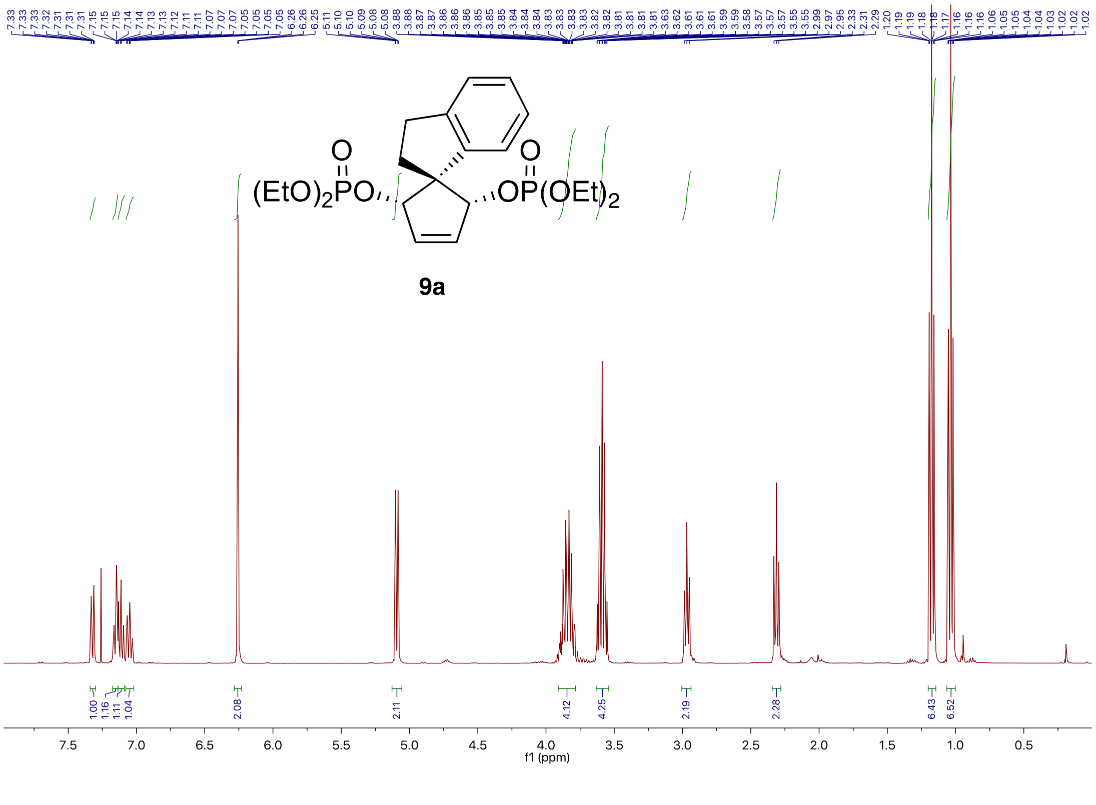

^{13}C NMR (101 MHz, $CDCl_3$)

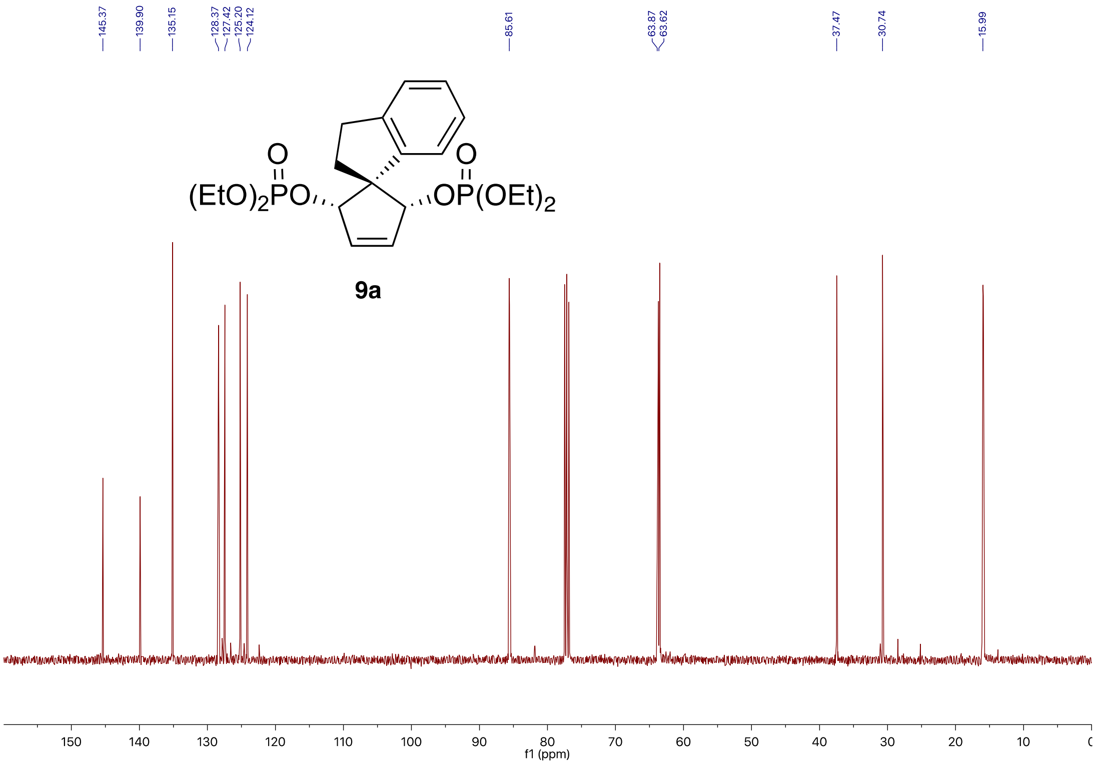

^{31}P NMR (162 MHz, $CDCl_3$)

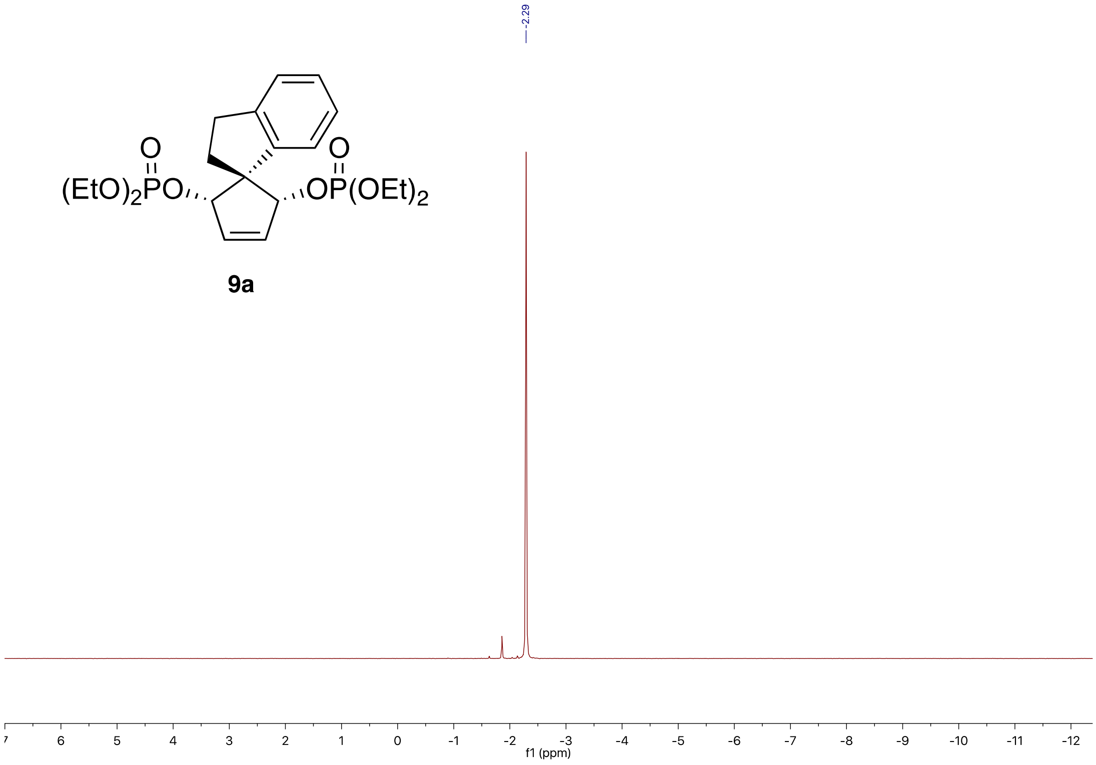

Supplementary Figure 21 - (2'*R*,4*S*,5'*S*)-Spiro[chromane-4,1'-cyclopentan]-3'-ene-2',5'-bis(diethyl phosphate) (**10a**) ^{1}H NMR (400 MHz, $CDCl_3$)

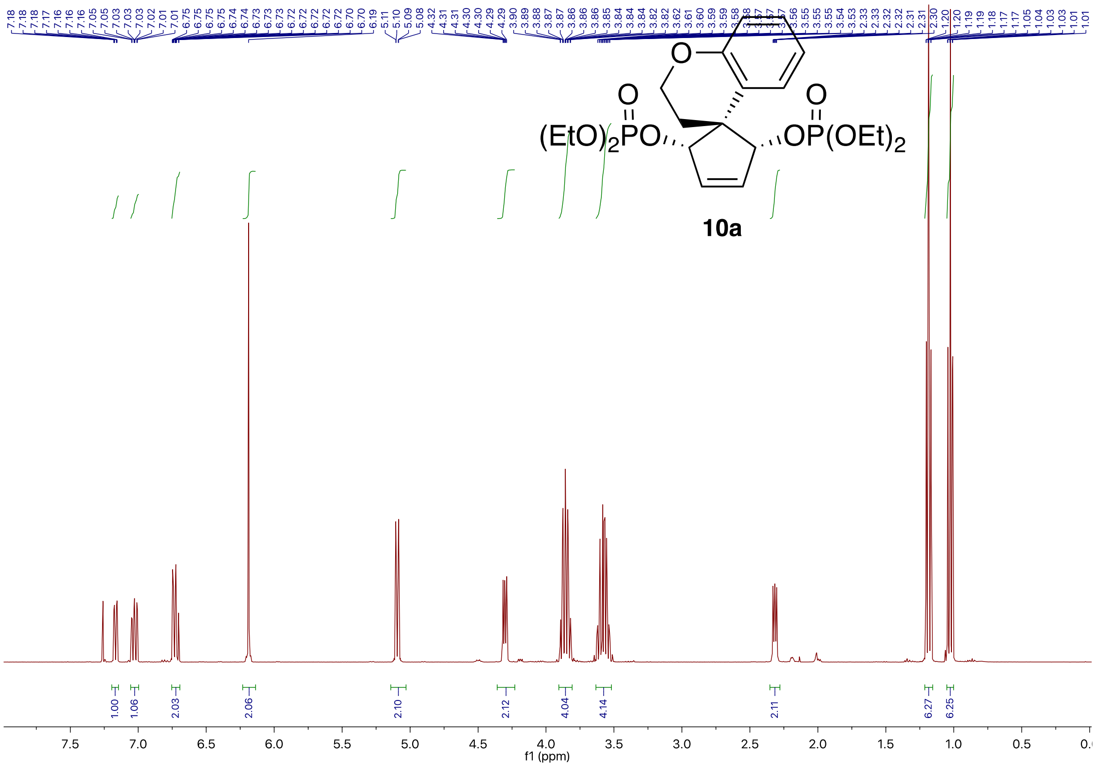

^{13}C NMR (101 MHz, $CDCl_3$)

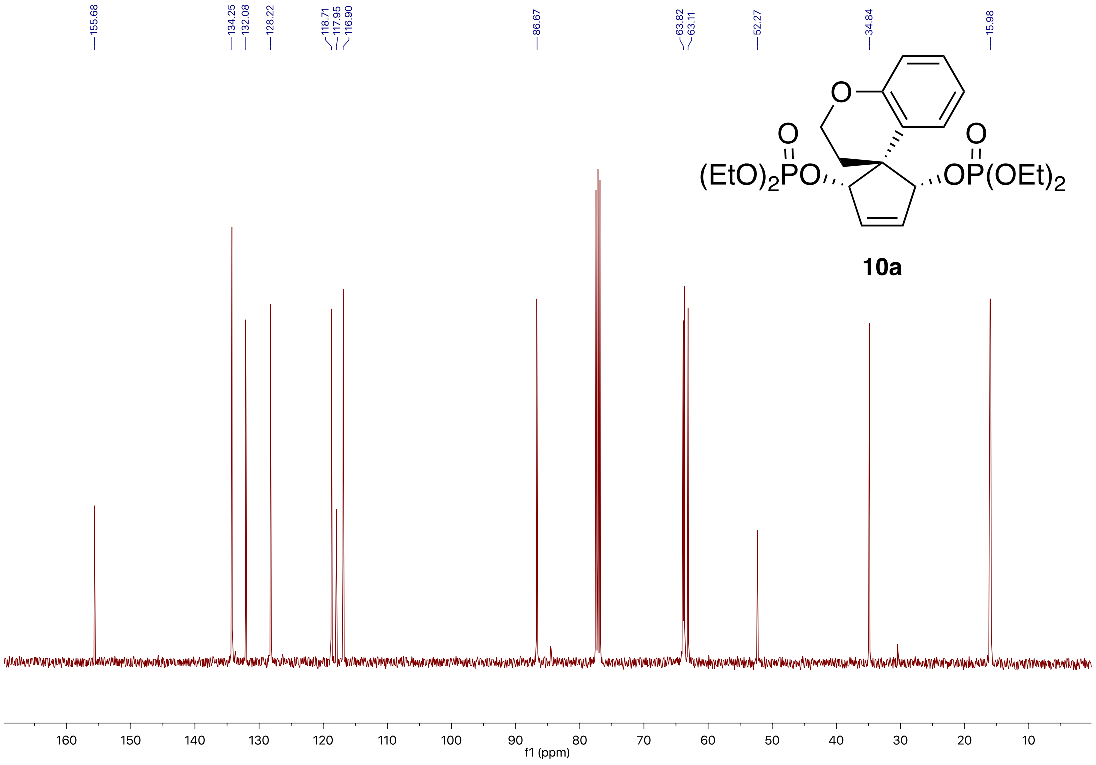

^{31}P NMR (162 MHz, $CDCl_3$)

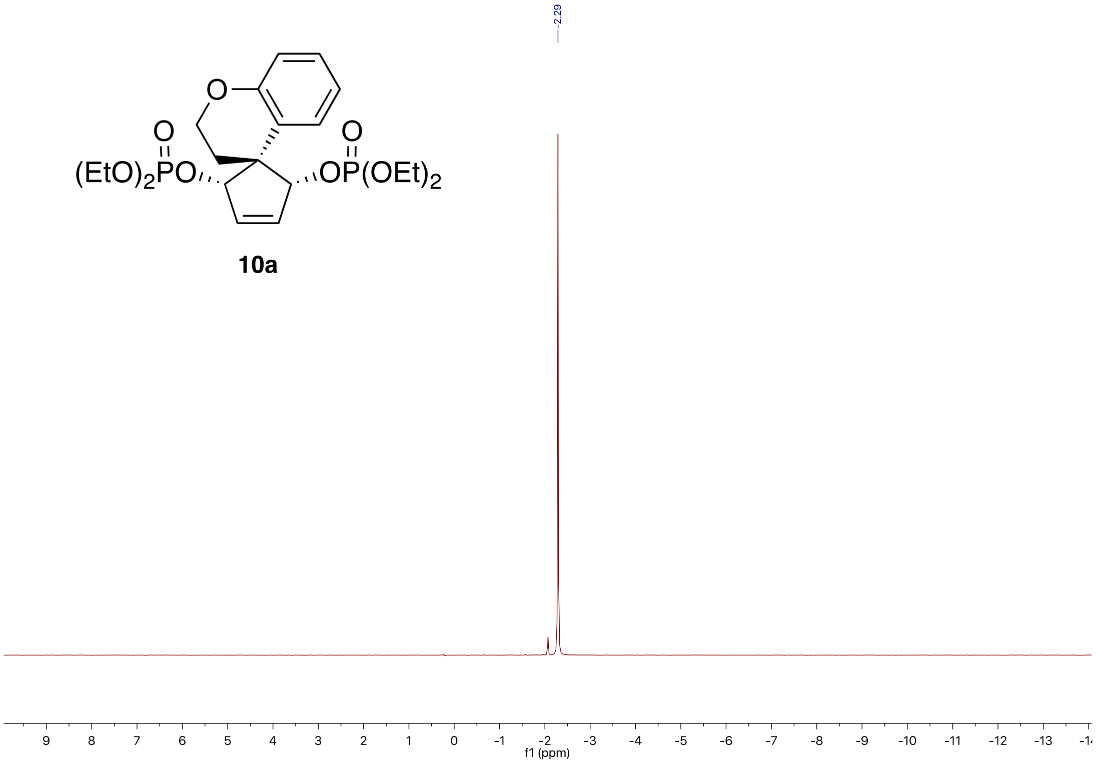

Supplementary Figure 22 - Tetraethyl ((3*R*,6*S*)-2,3,6,7-tetrahydrooxepine-3,6-diyl) bis(phosphate) **(11a)** ^{1}H NMR (400 MHz, $CDCl_3$)

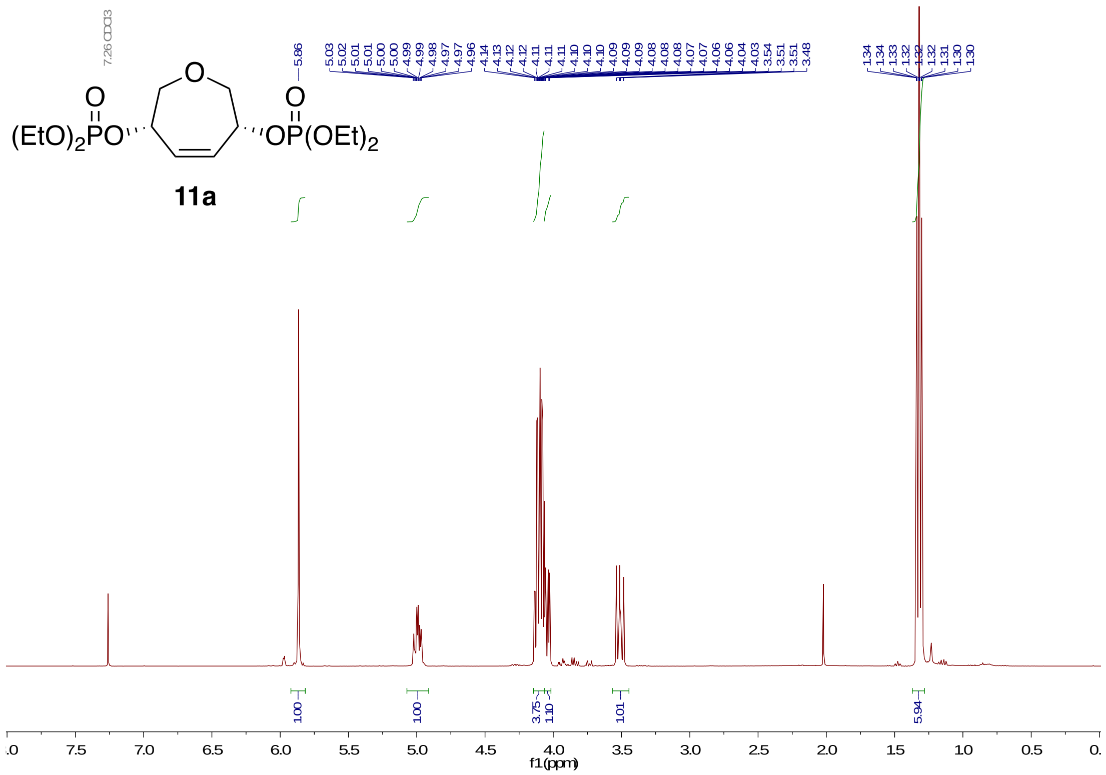

^{13}C NMR (101 MHz, $CDCl_3$)

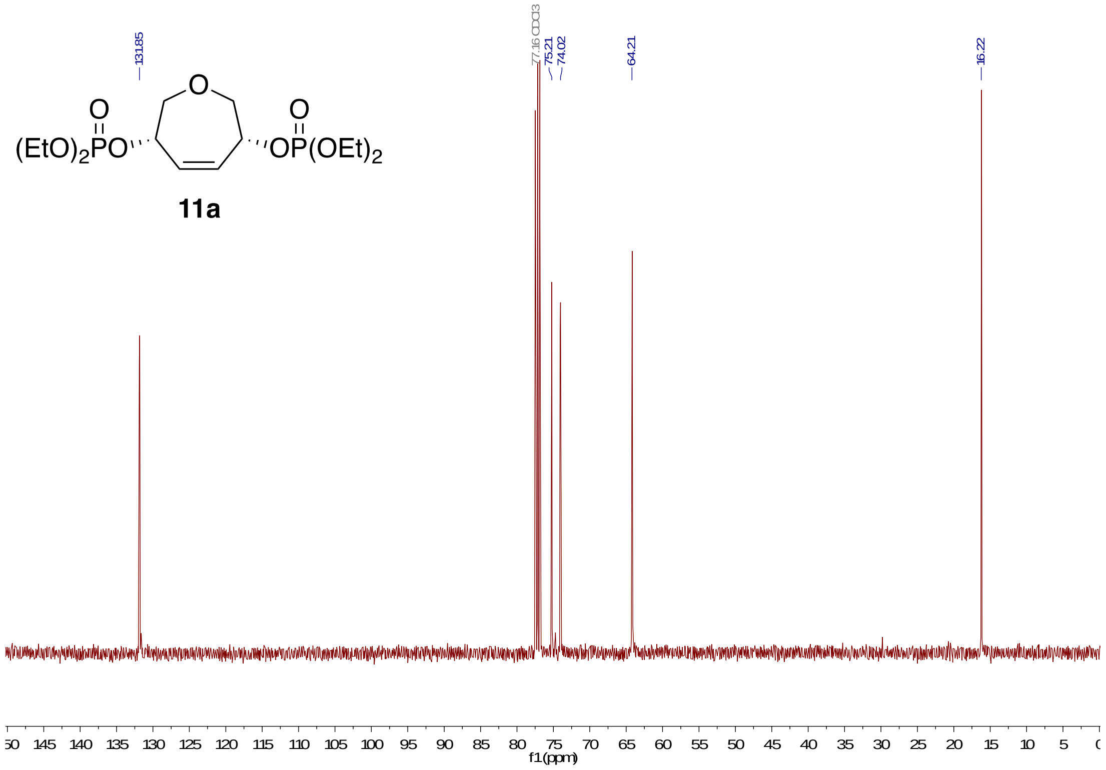

^{31}P NMR (162 MHz, $CDCl_3$)

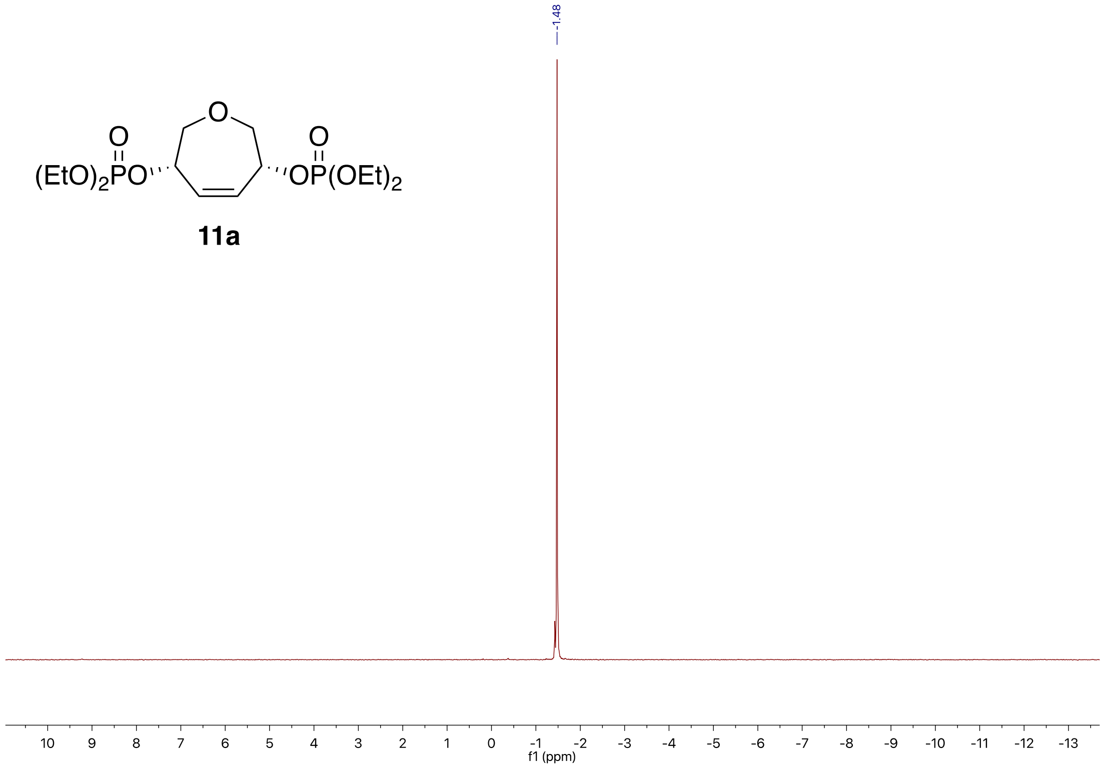

Supplementary Figure 23 - *tert*-Butyl (3*R*,6*S*)-3,6-bis((diethoxyphosphoryl)oxy)-2,3,6,7-tetrahydro-1*H*-azepine-1-carboxylate **(12a)** ^{1}H NMR (400 MHz, DMSO-d_6, 338K)

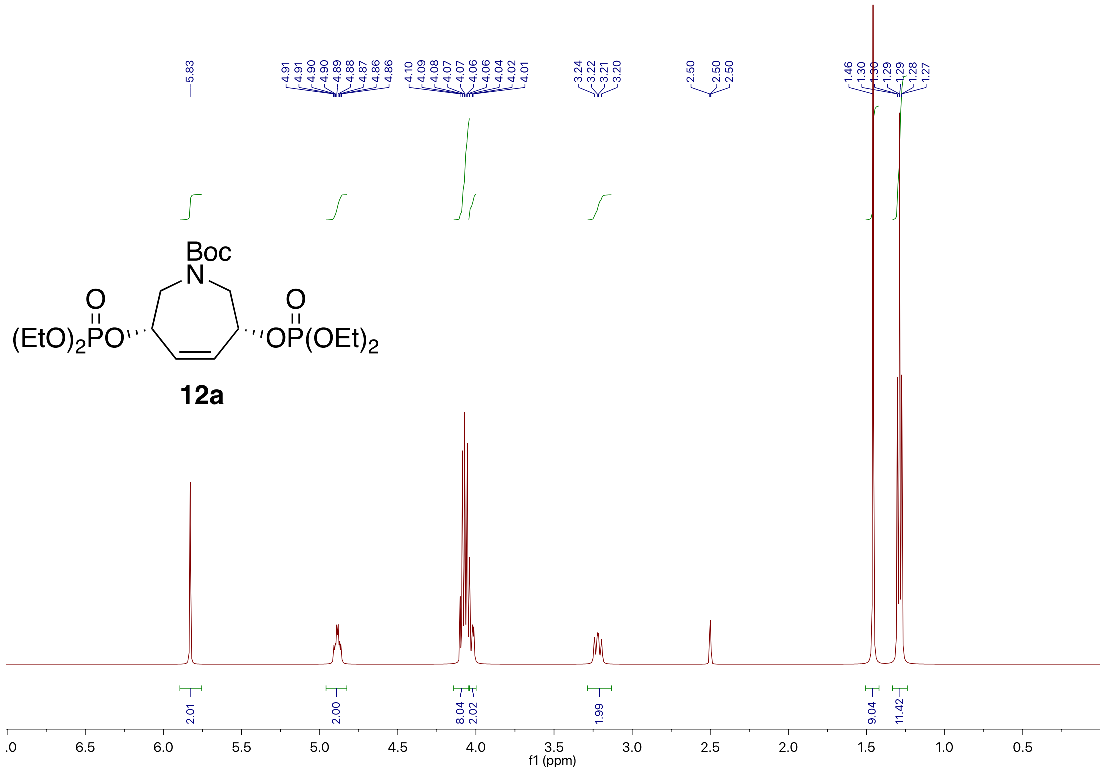

^{13}C NMR (101 MHz, $CDCl_3$)

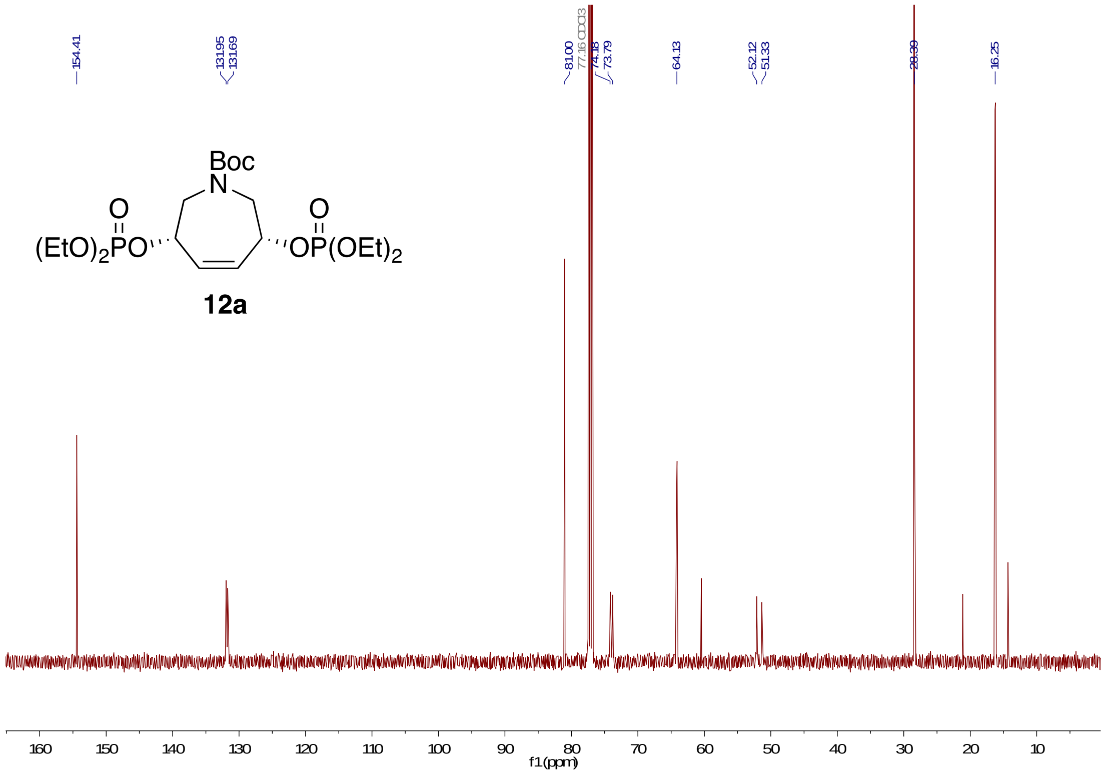

^{31}P NMR (162 MHz, CDCl$_3$)

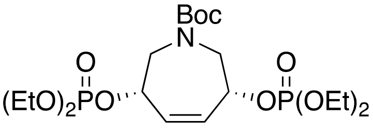

12a

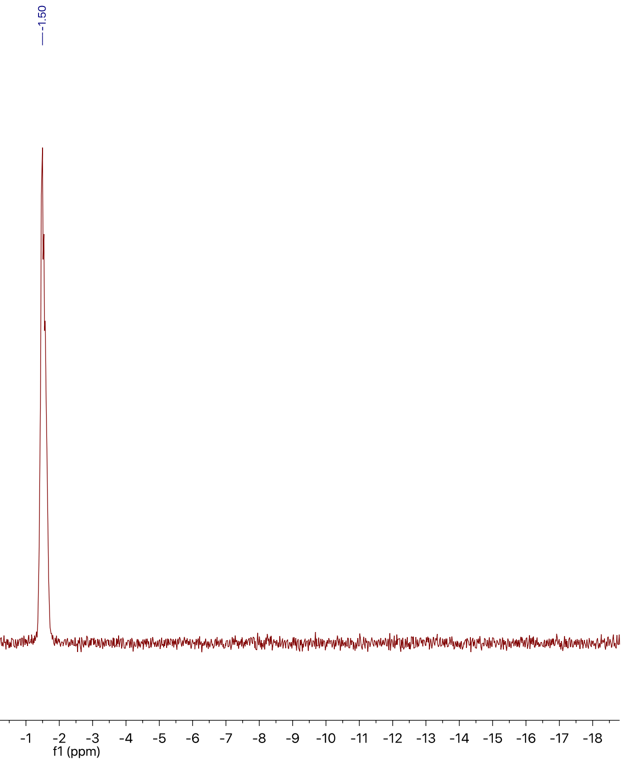

Supplementary Figure 24 - Dimethyl ((1*R*,2*R*)-2-(4-phenylbutyl)cyclopent-3-en-1-yl) phosphate ^{1}H NMR (400 MHz, $CDCl_3$)

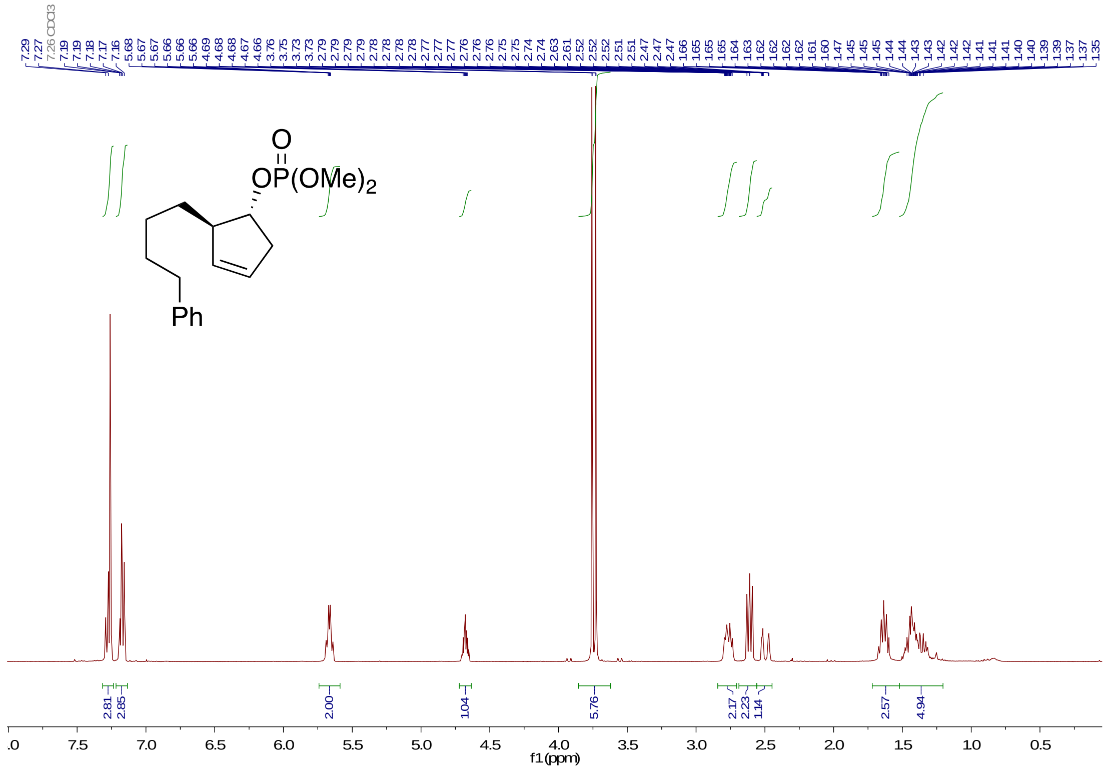

^{13}C NMR (101 MHz, $CDCl_3$)

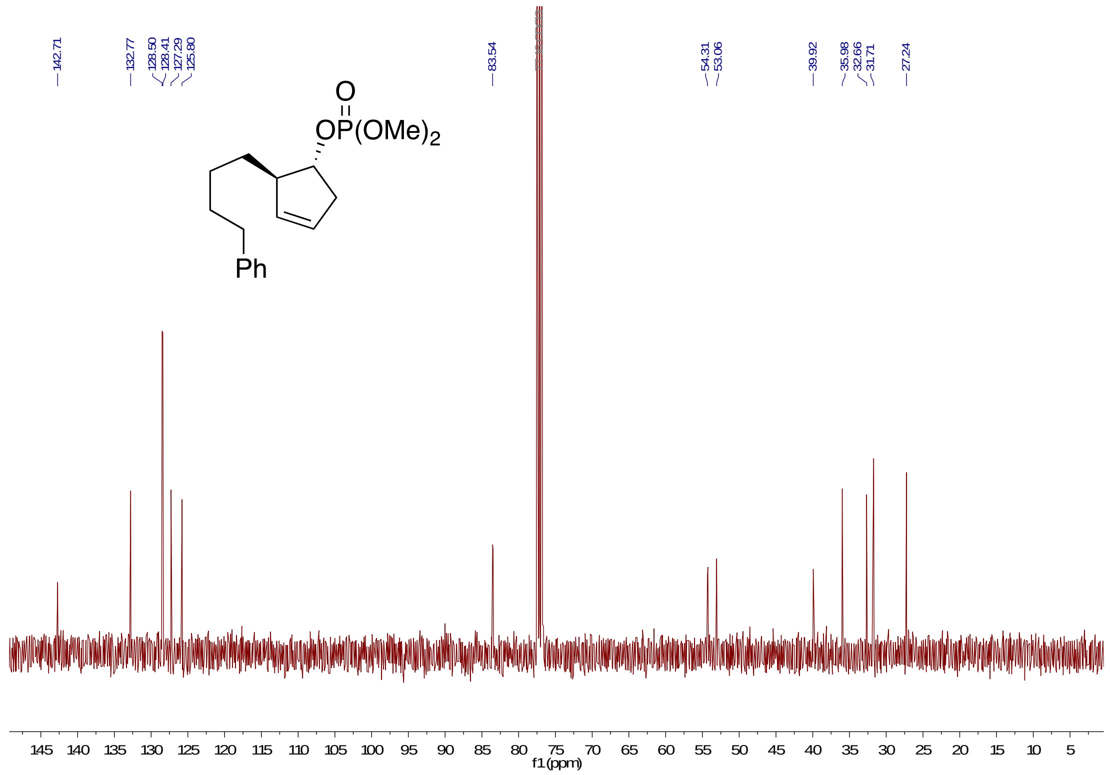

^{31}P NMR (162 MHz, $CDCl_3$)

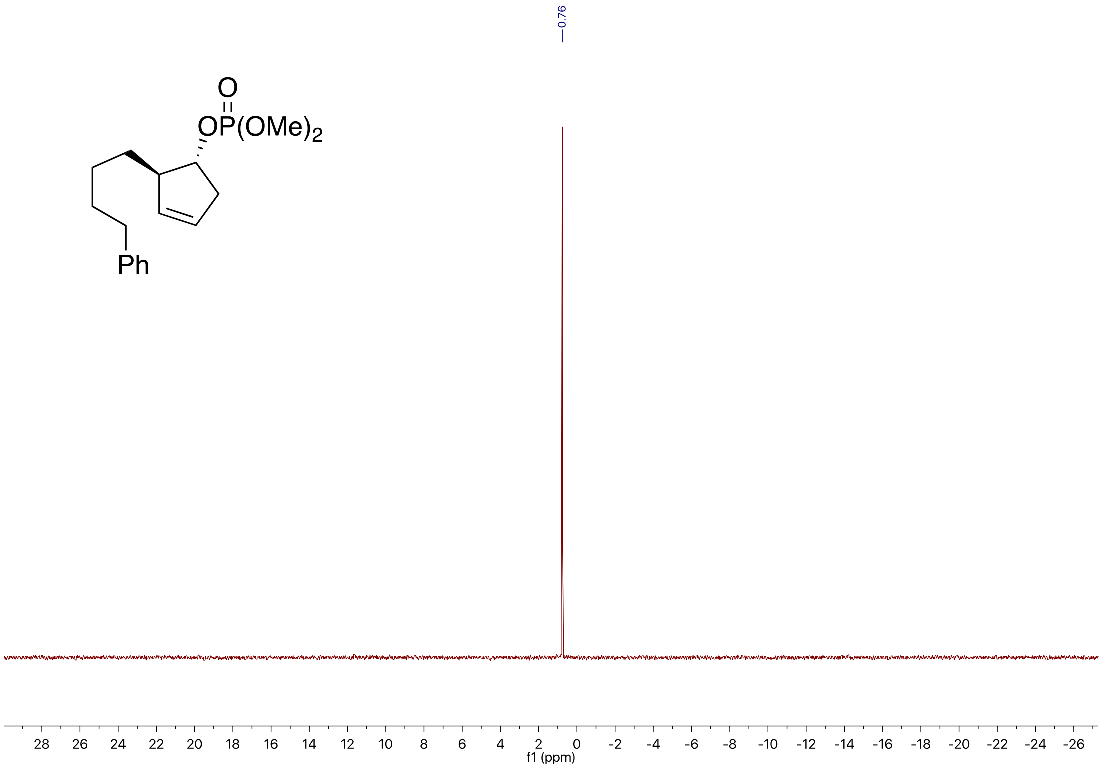

HPLC Trace

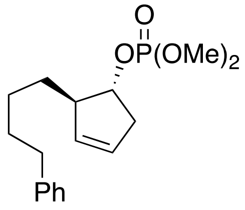

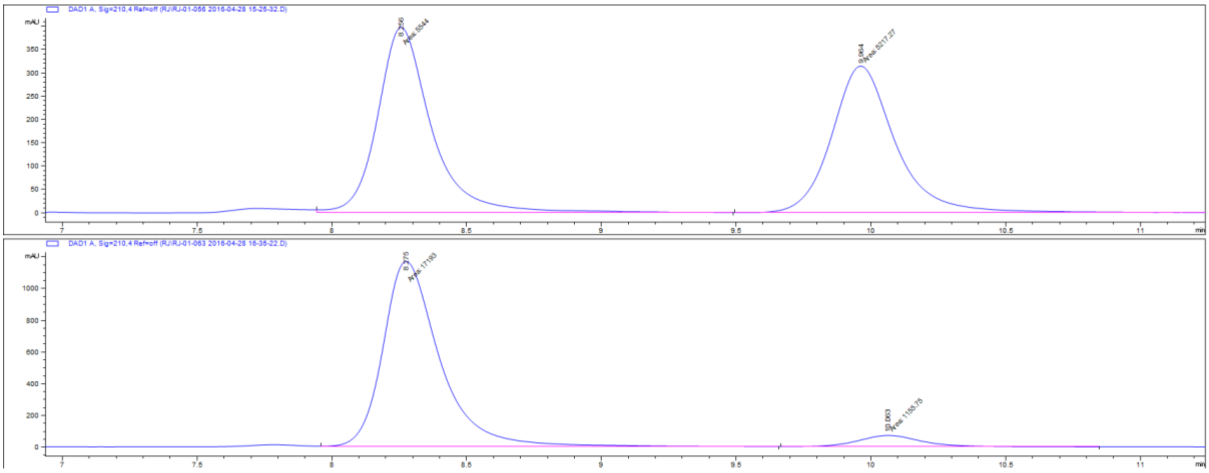

Supplementary Figure 25 - Diethyl ((1*R*,2*R*)-2-(4-phenylbutyl)cyclopent-3-en-1-yl) phosphate (**1b**) ^{1}H NMR (400 MHz, $CDCl_3$)

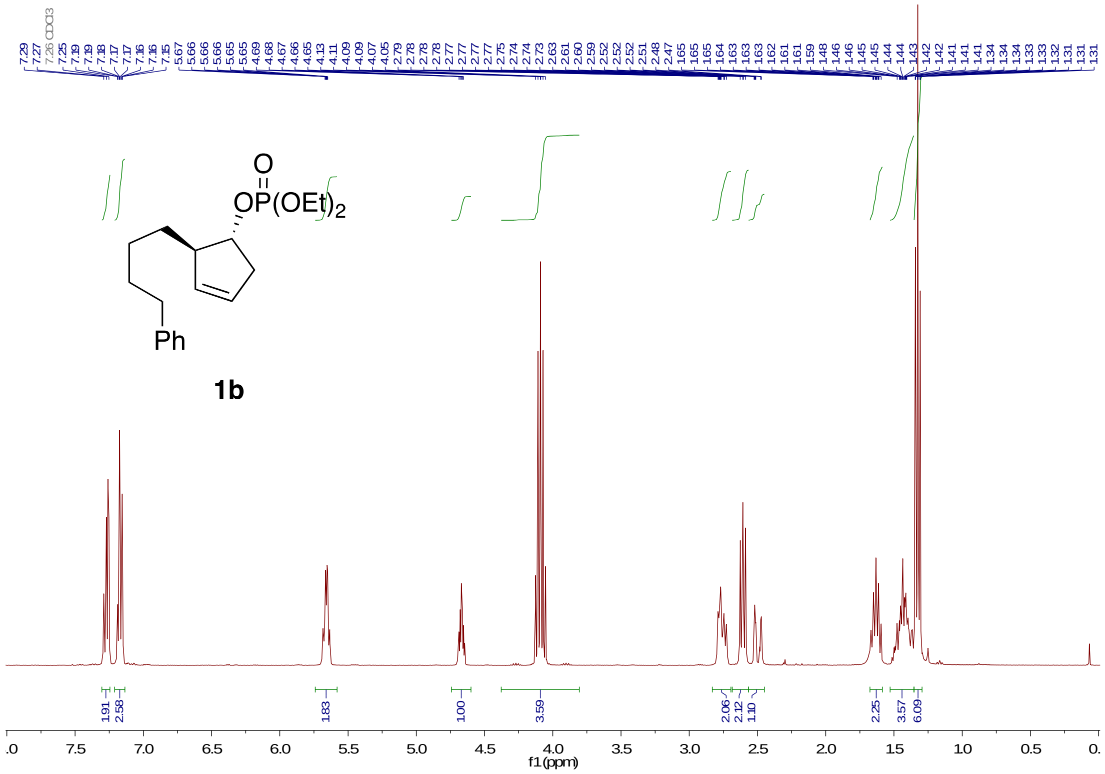

^{13}C NMR (101 MHz, $CDCl_3$)

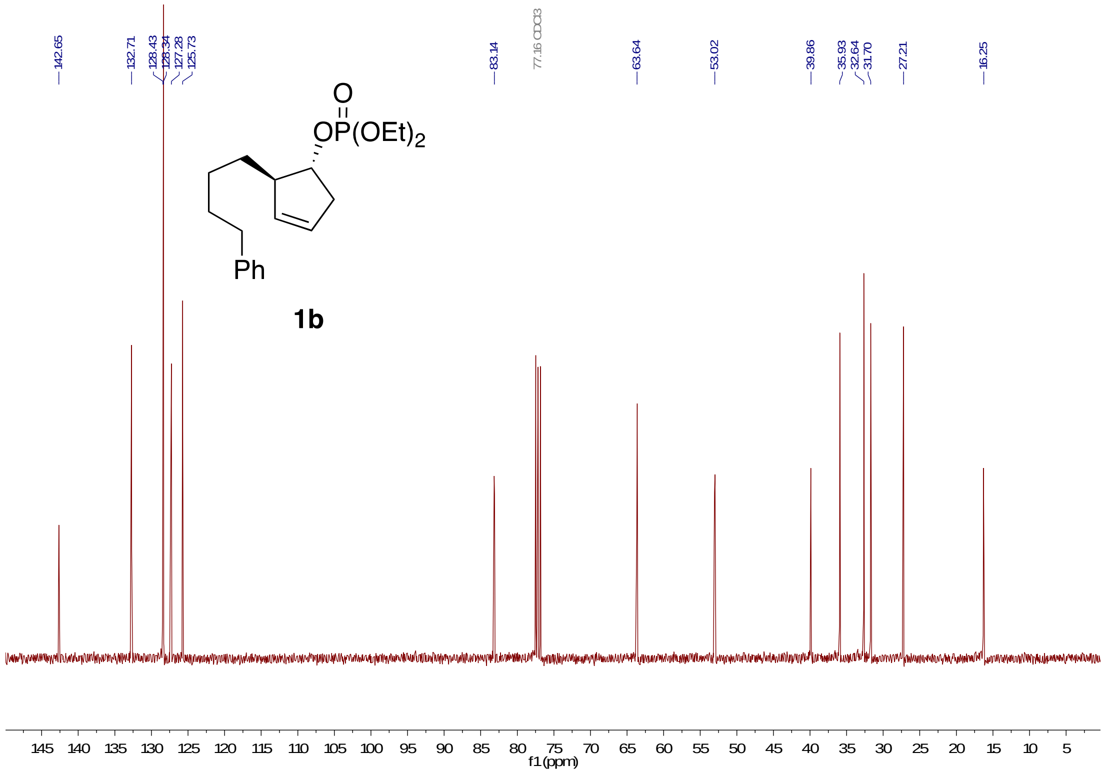

^{31}P NMR (162 MHz, $CDCl_3$)

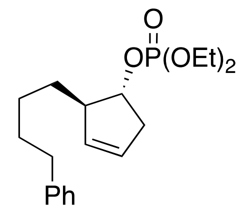

1b

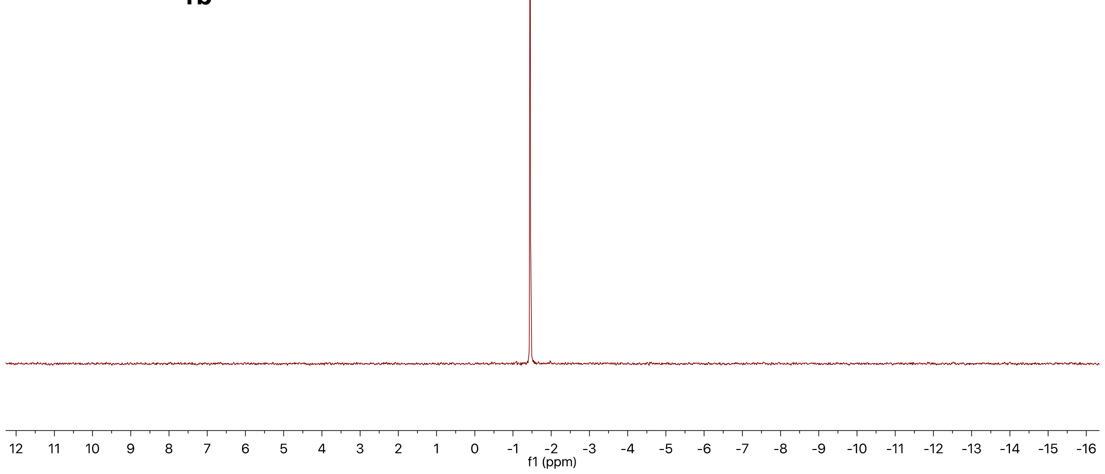

HPLC Trace

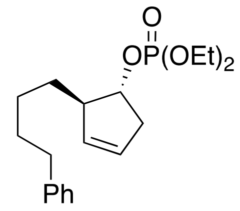

1b

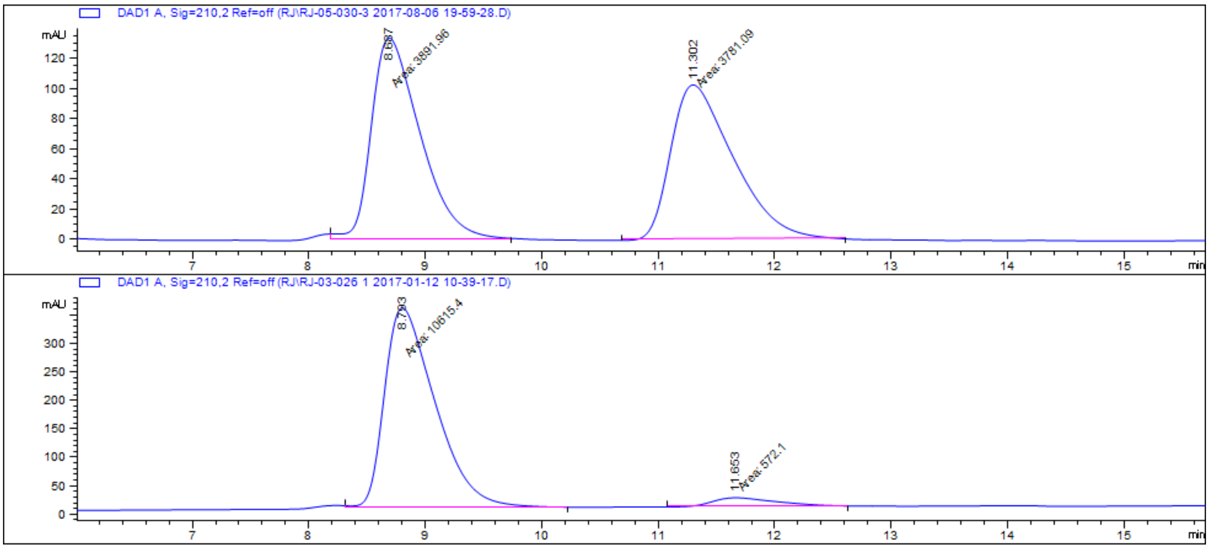

Supplementary Figure 26 - Diisopropyl ((1*R*,2*R*)-2-(4-phenylbutyl)cyclopent-3-en-1-yl) phosphate ^{1}H NMR (400 MHz, $CDCl_3$)

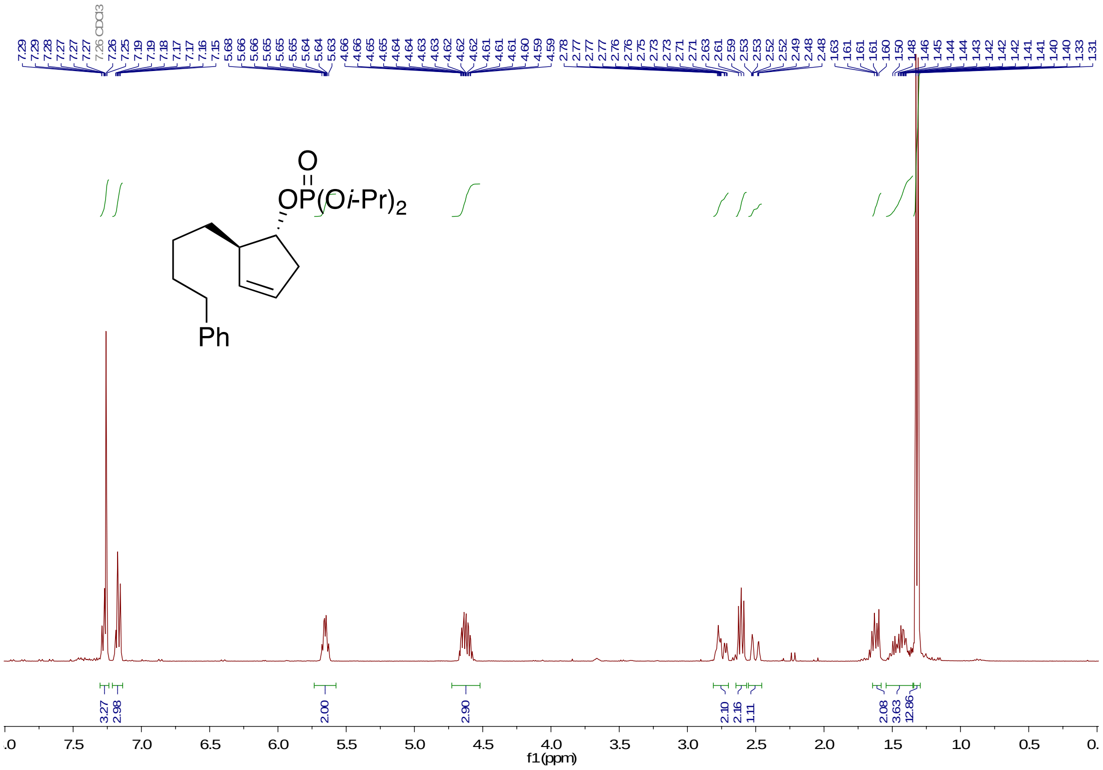

^{13}C NMR (101 MHz, $CDCl_3$)

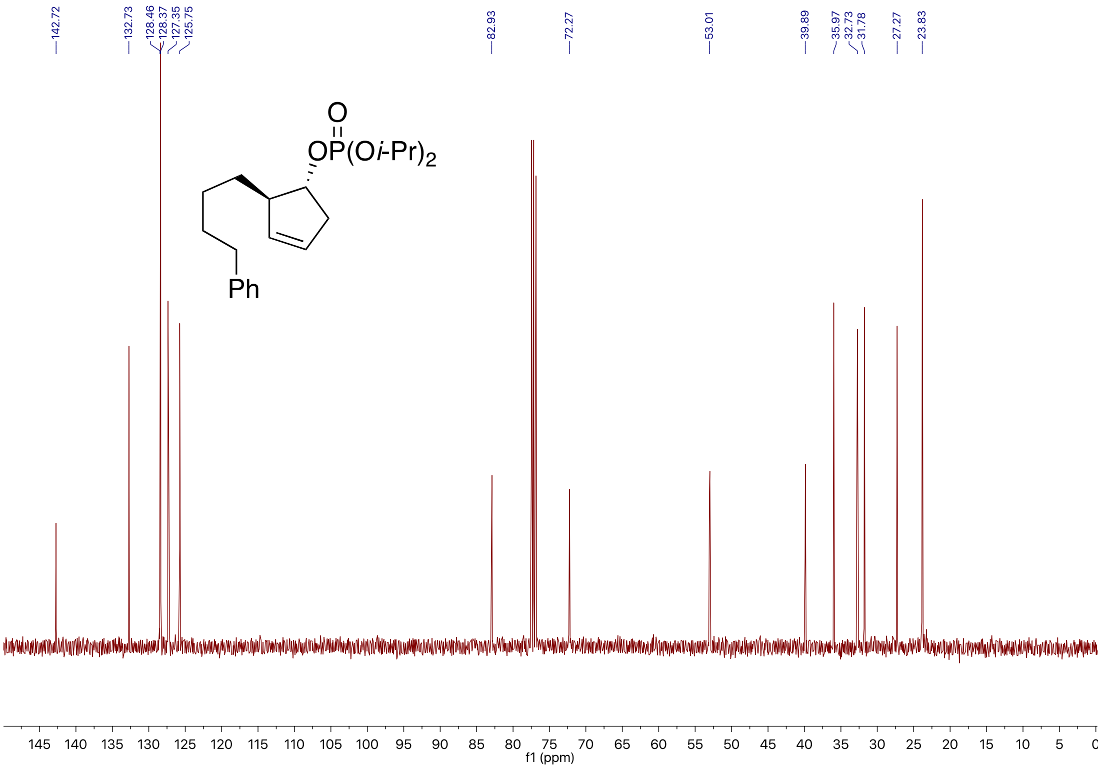

^{31}P NMR (162 MHz, $CDCl_3$)

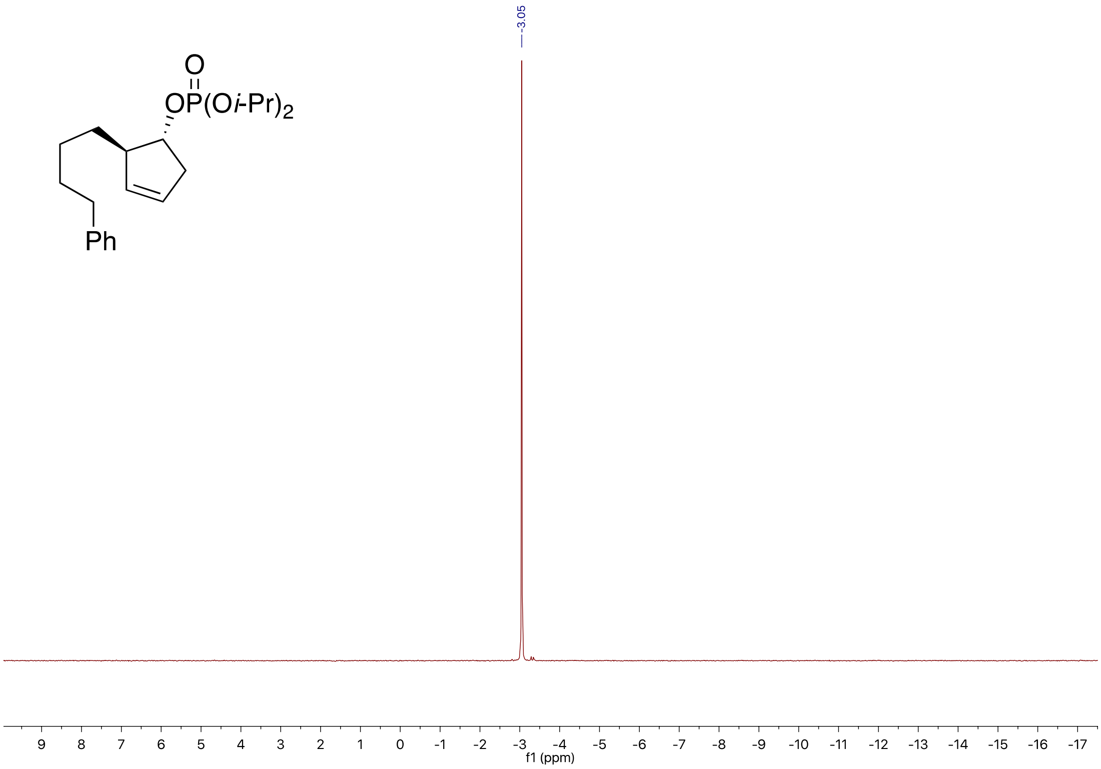

HPLC Trace

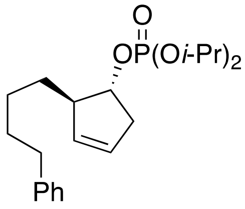

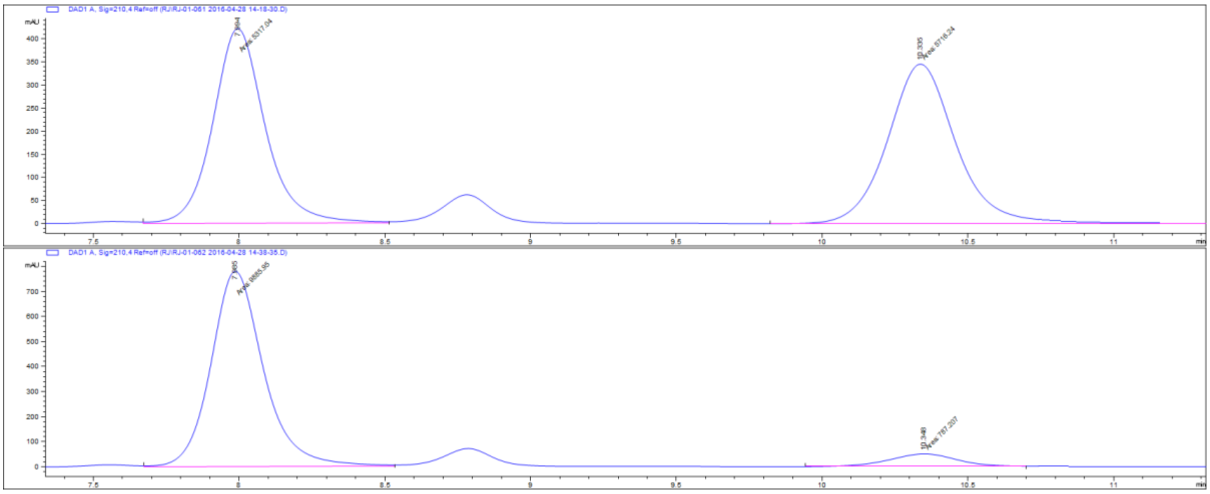

Supplementary Figure 27 - Diphenyl ((1*R*,2*R*)-2-(4-phenylbutyl)cyclopent-3-en-1-yl) phosphate ^{1}H NMR (400 MHz, $CDCl_3$)

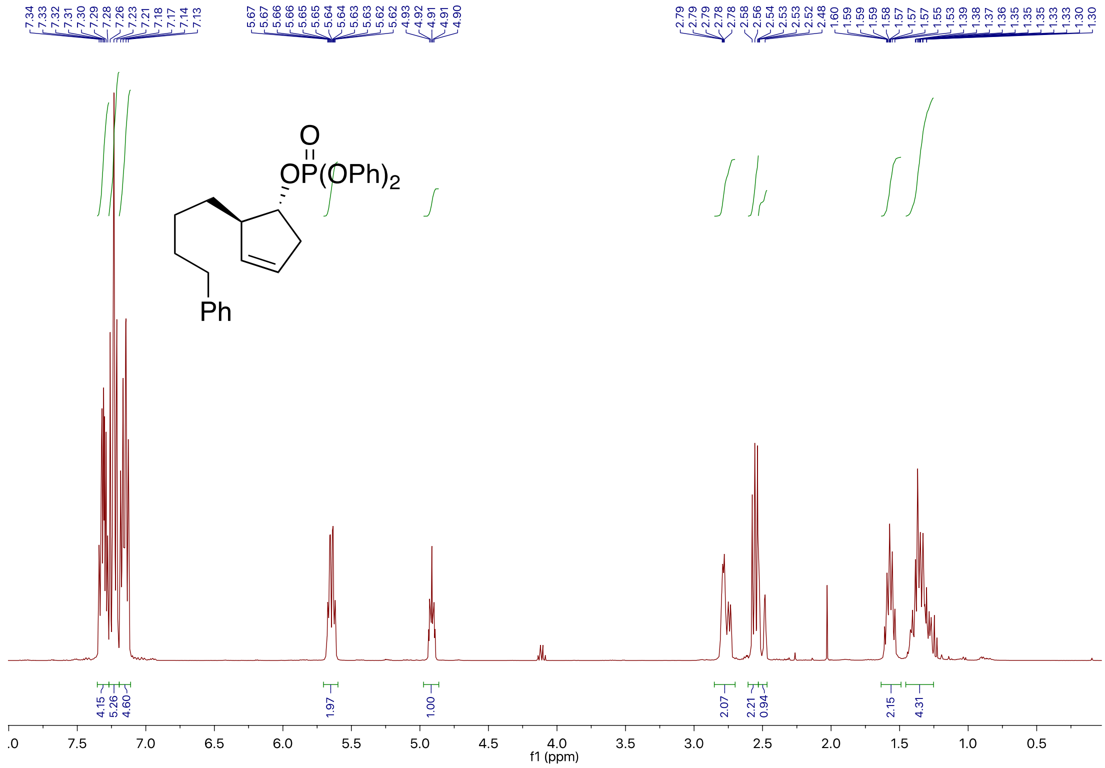

^{13}C NMR (101 MHz, $CDCl_3$)

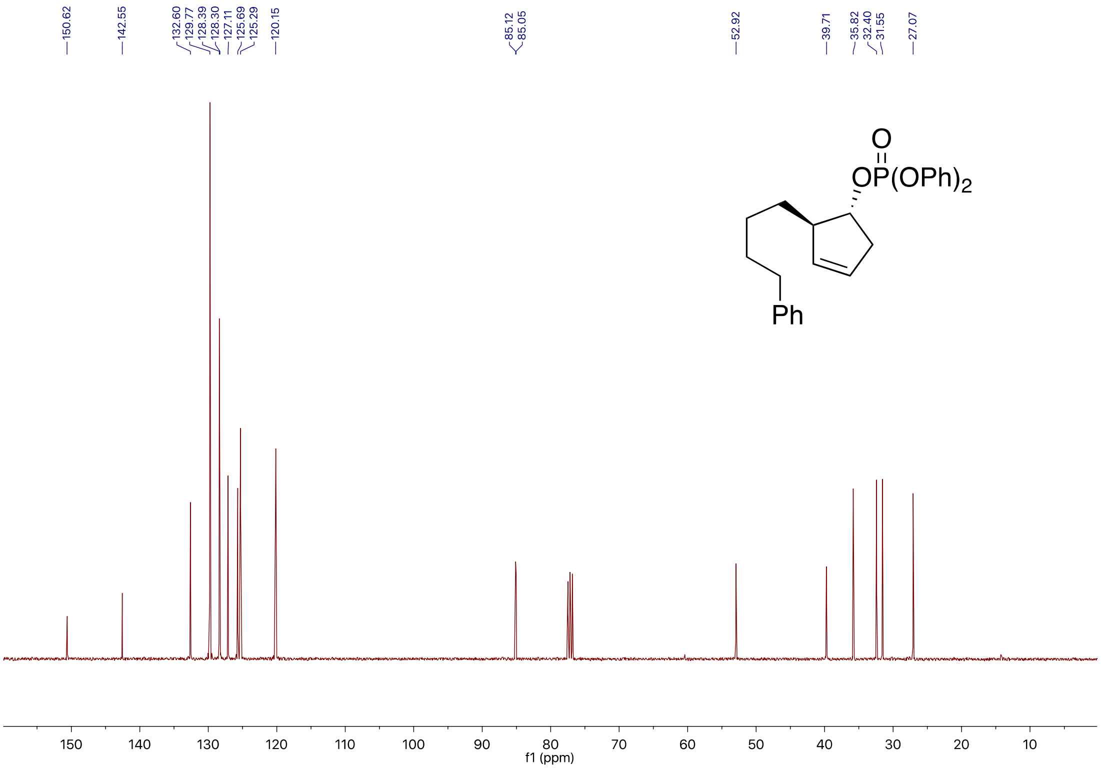

^{31}P NMR (162 MHz, $CDCl_3$)

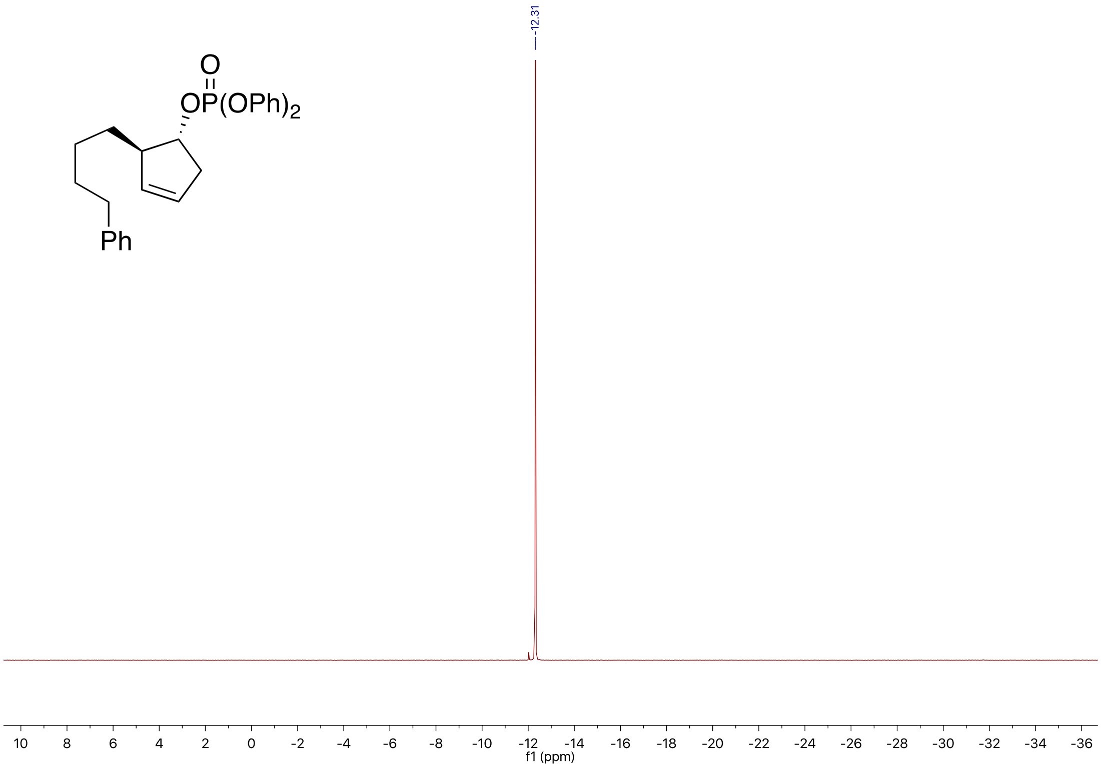

HPLC Trace

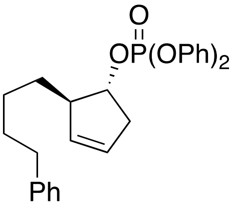

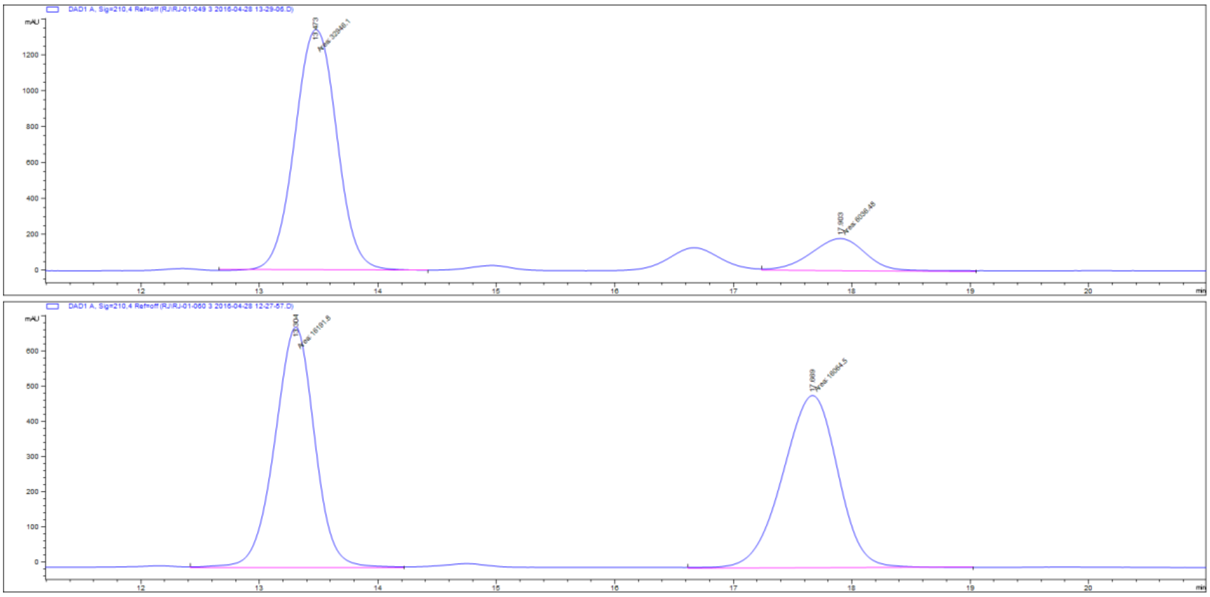

Supplementary Figure 28 - Diethyl ((1*R*,2*R*)-2-(4-phenylbutyl)cyclohex-3-en-1-yl) phosphate (**2b**) ^{1}H NMR (400 MHz, $CDCl_3$)

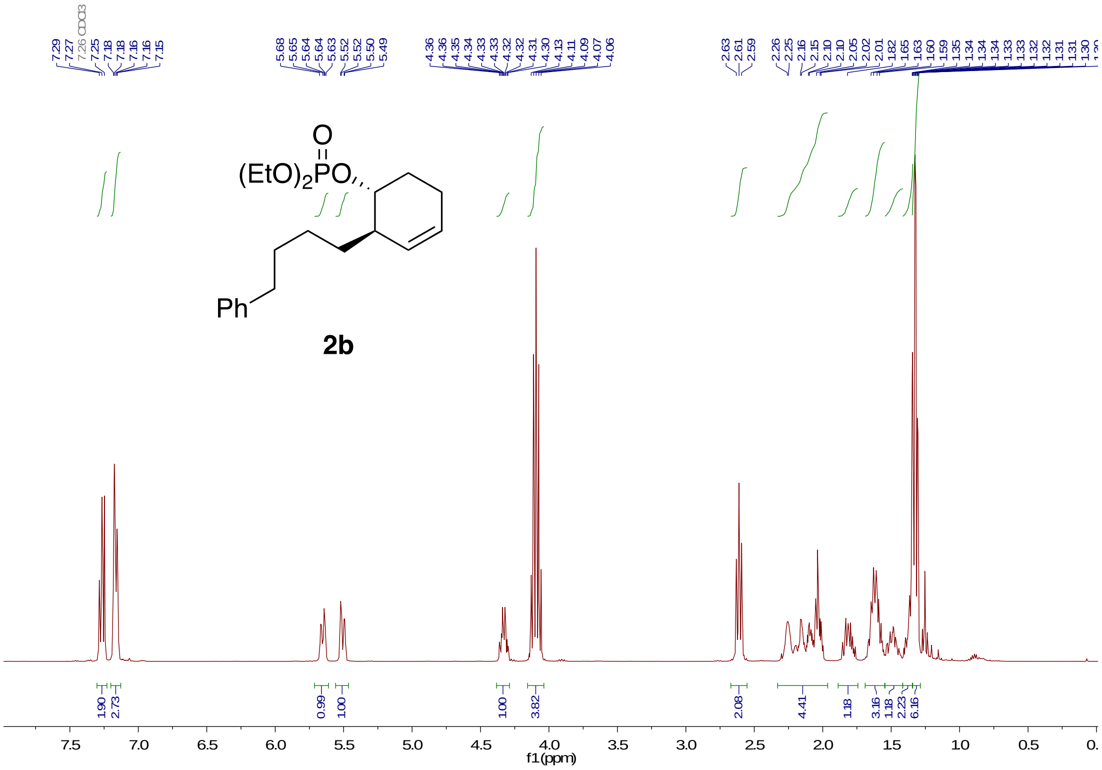

^{13}C NMR (101 MHz, $CDCl_3$)

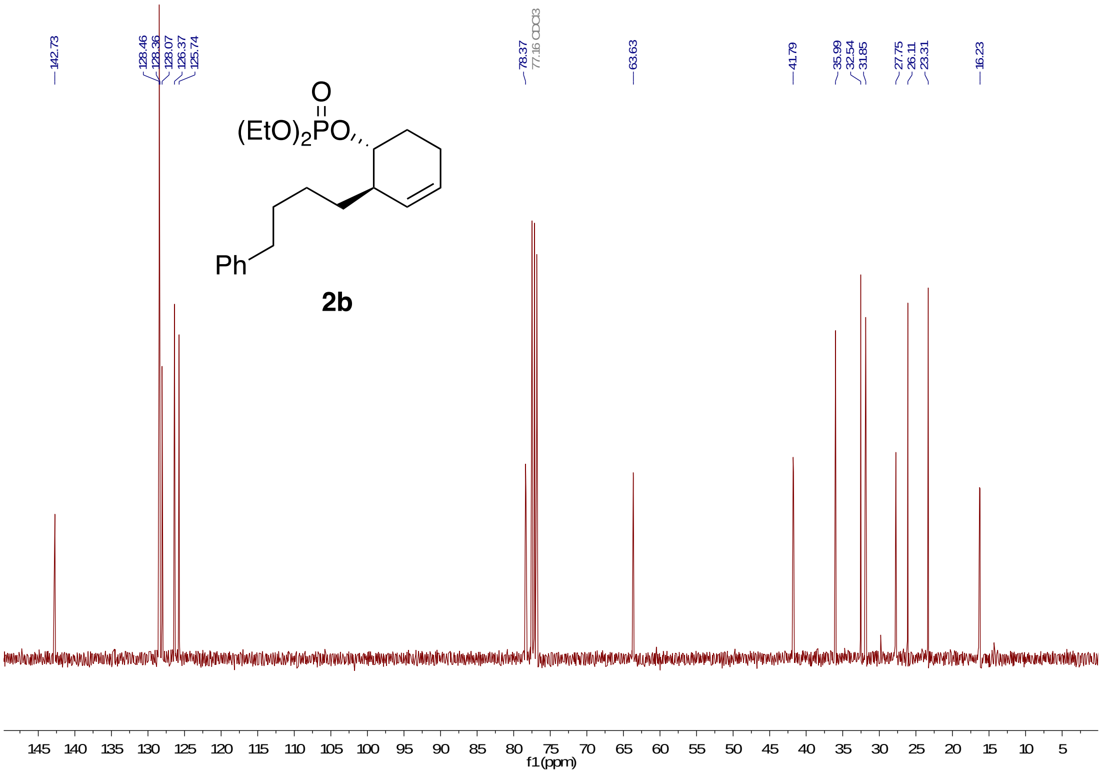

^{31}P NMR (162 MHz, $CDCl_3$)

2b

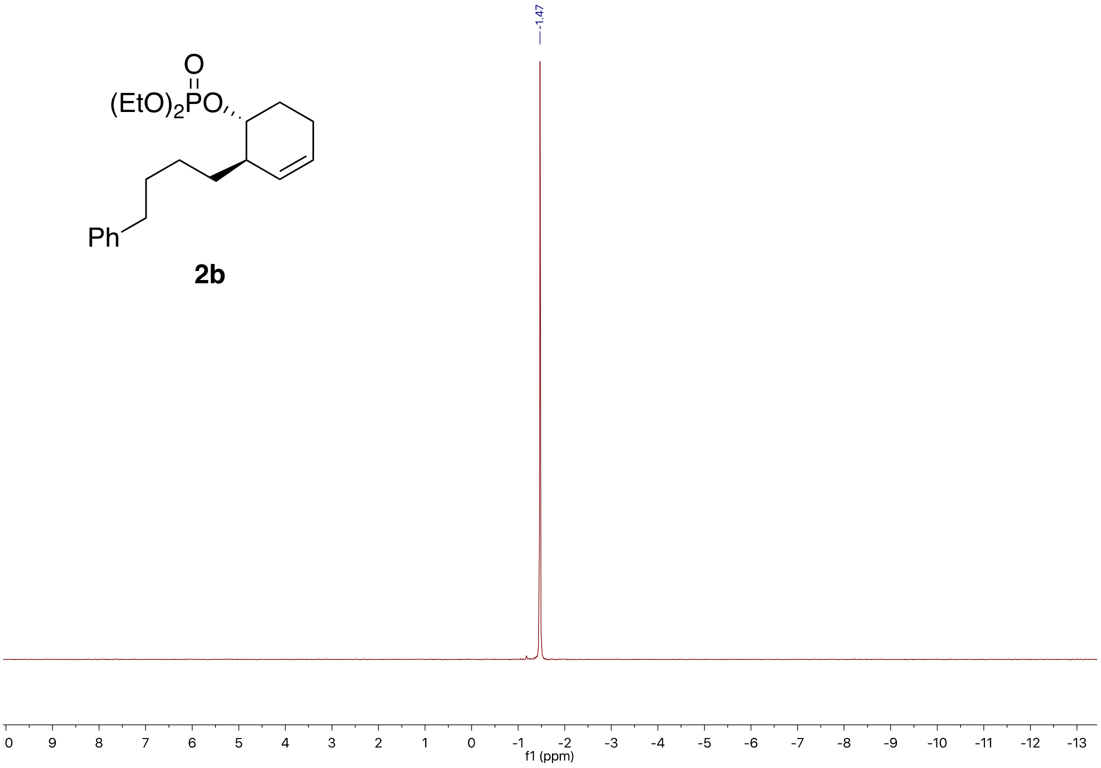

HPLC Trace

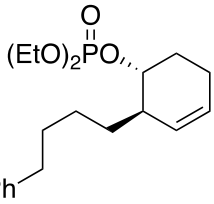

2b

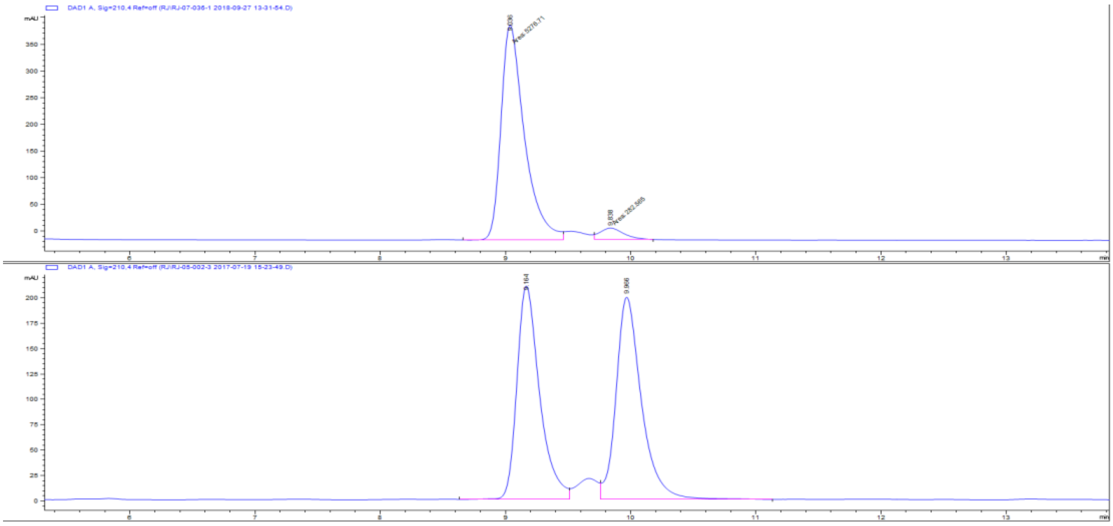

Supplementary Figure 29 - Diethyl ((1*R*,2*R*)-2-(4-phenylbutyl)cyclohept-3-en-1-yl) phosphate (**3b**) ^{1}H NMR (400 MHz, $CDCl_3$)

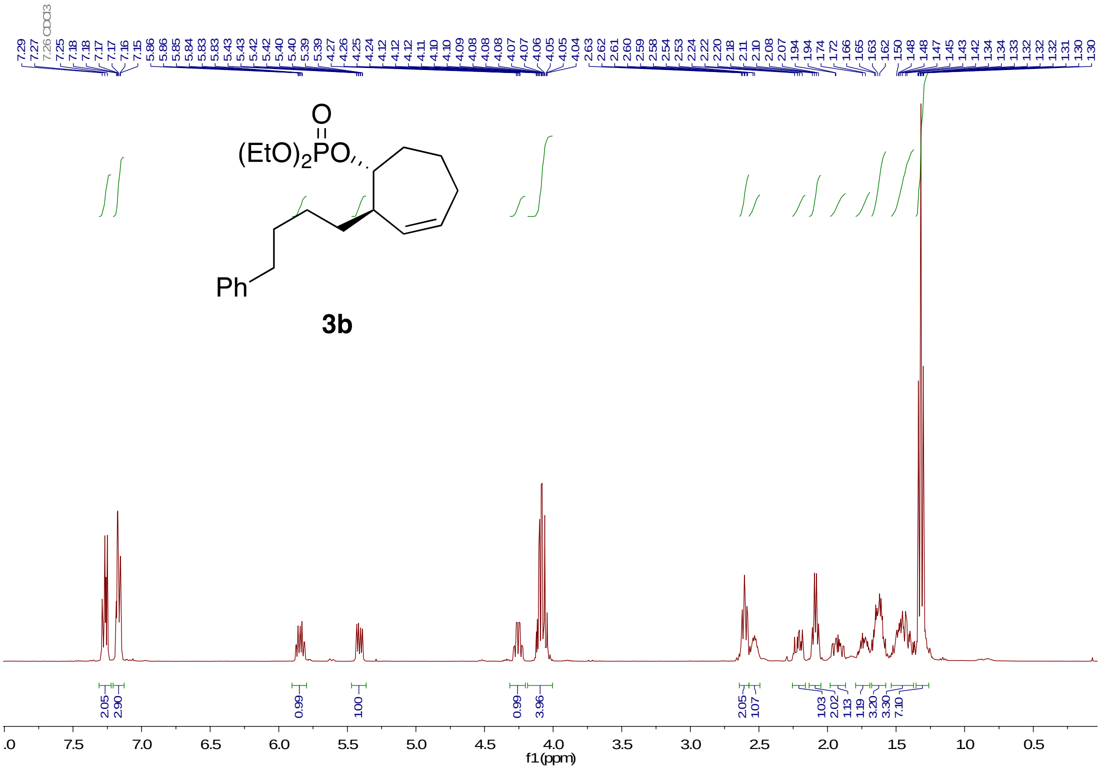

^{13}C NMR (101 MHz, $CDCl_3$)

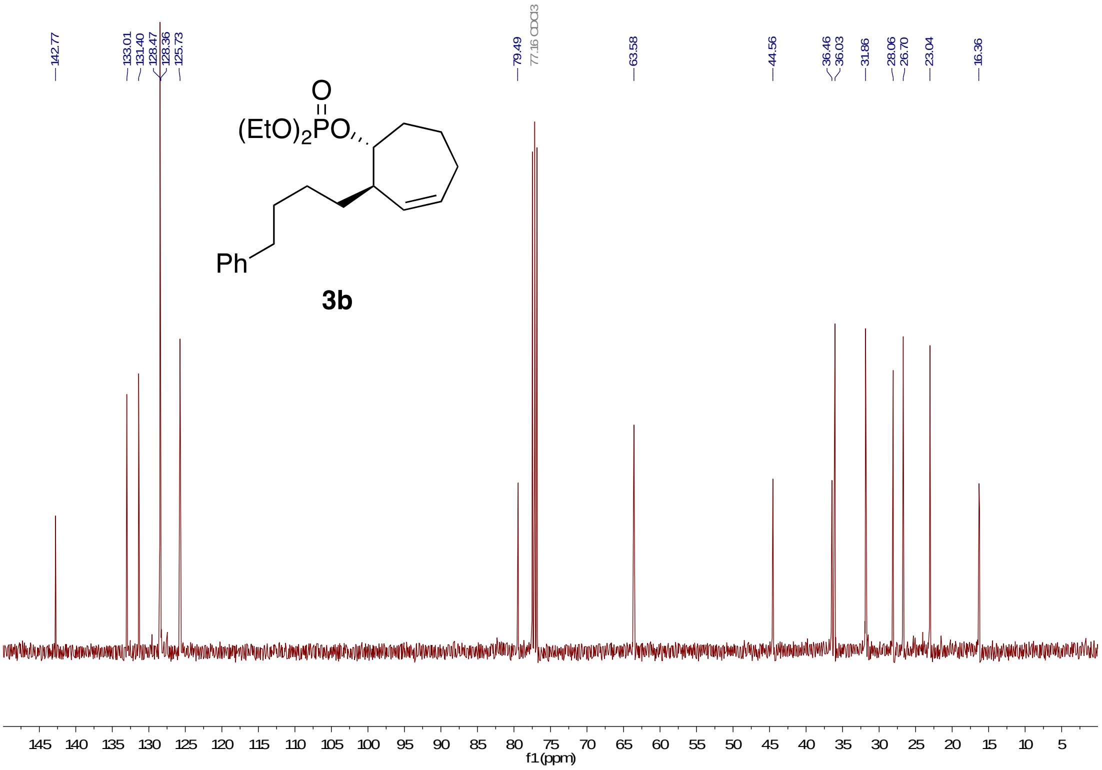

^{31}P NMR (162 MHz, $CDCl_3$)

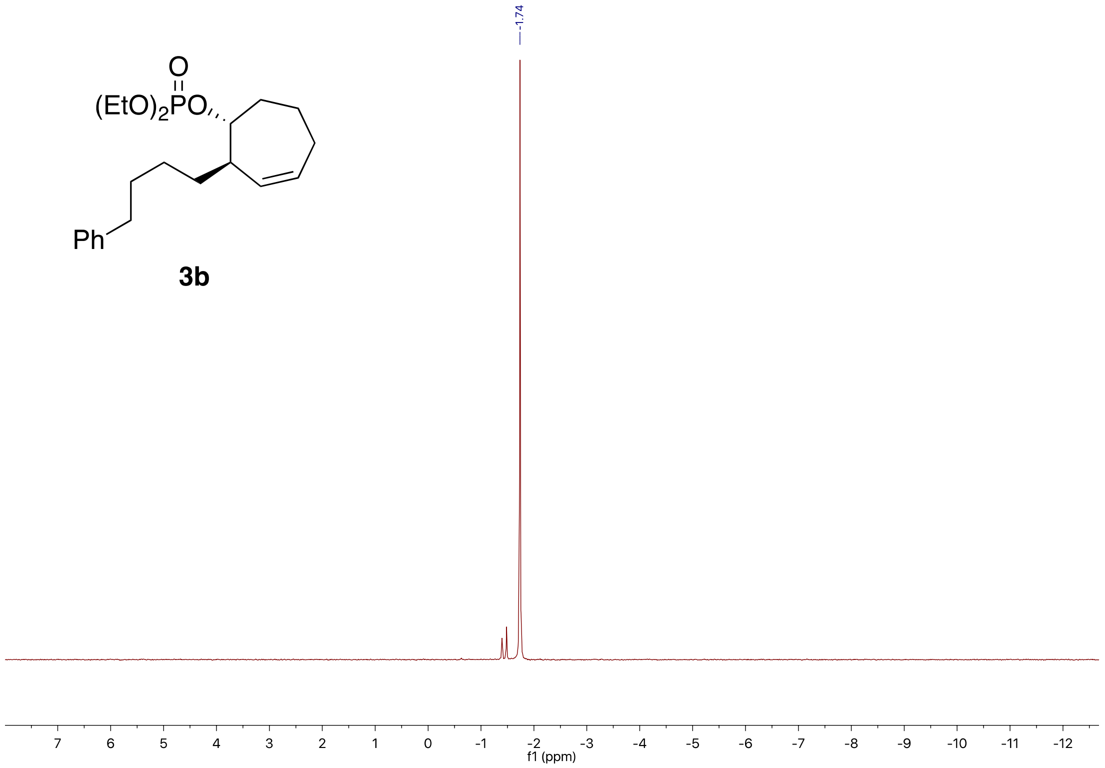

HPLC Trace

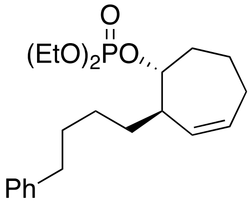

3b

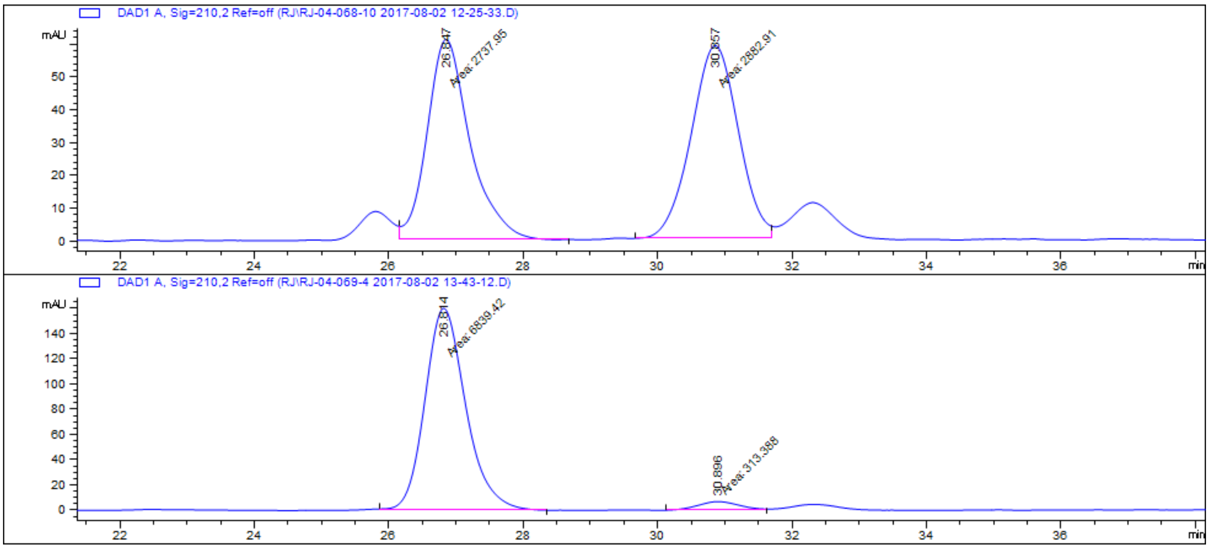

Supplementary Figure 30 - Diethyl ((1*R*,2*R*)-2-ethylcyclopent-3-en-1-yl) phosphate (**1c**) ^{1}H NMR (500 MHz, $CDCl_3$)

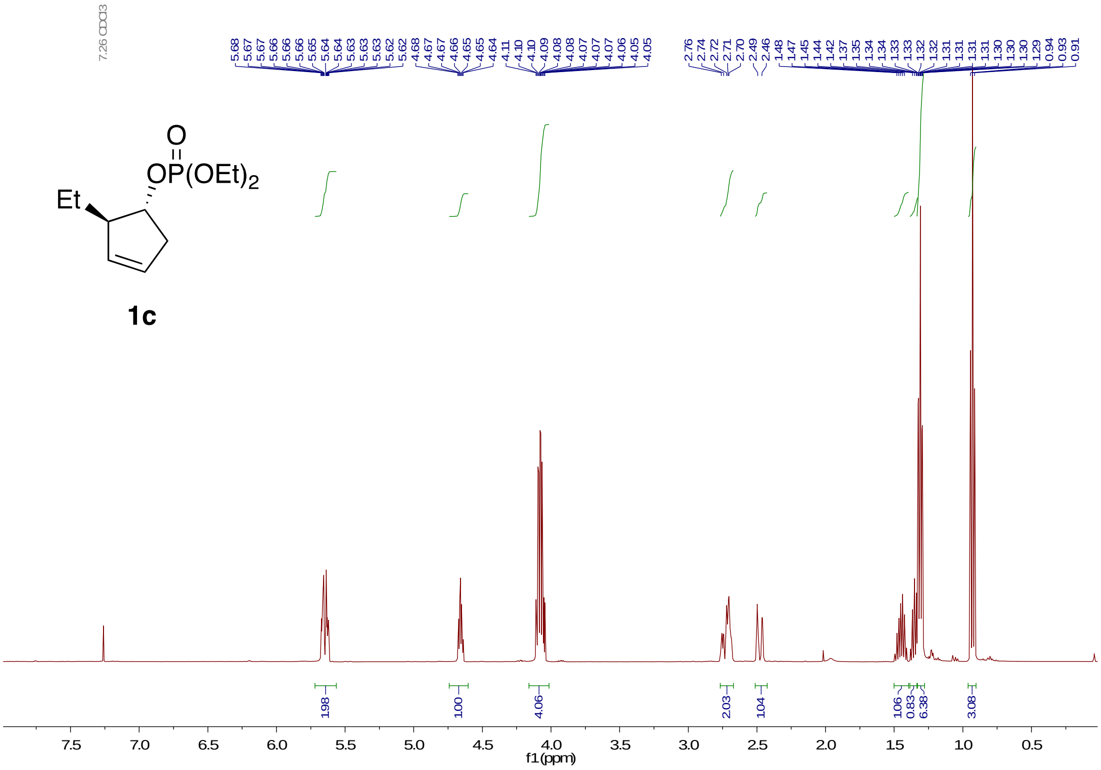

^{13}C NMR (126 MHz, $CDCl_3$)

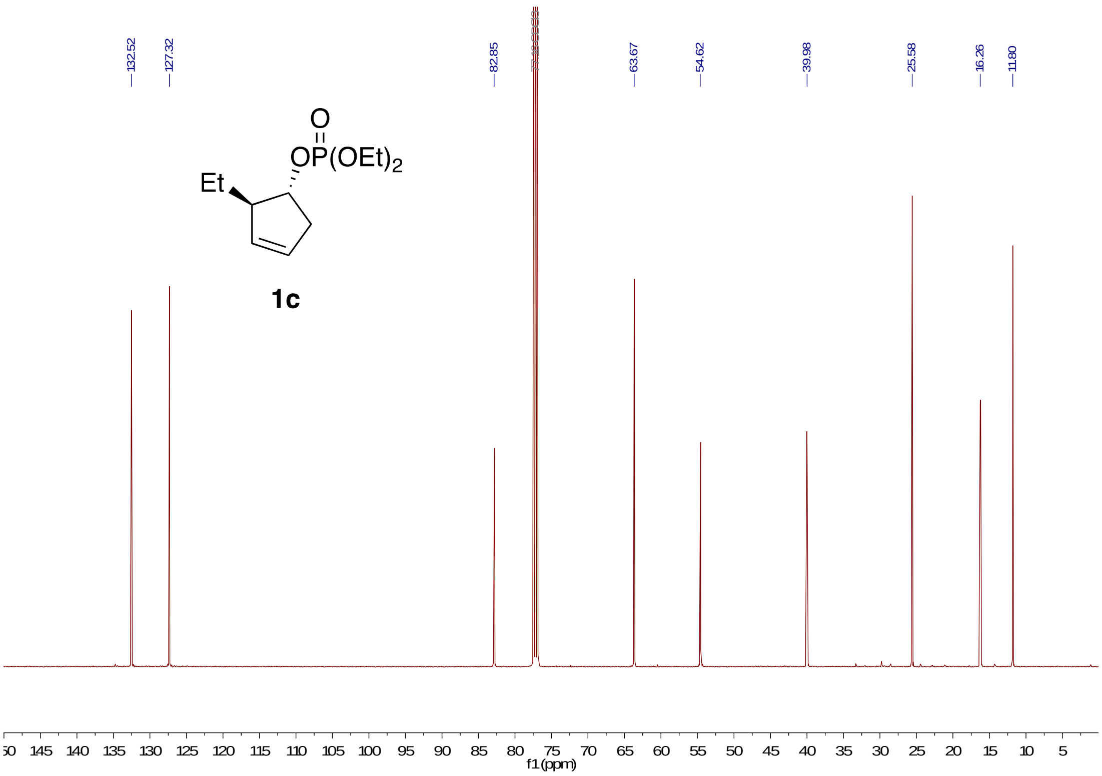

^{31}P NMR (202 MHz, $CDCl_3$)

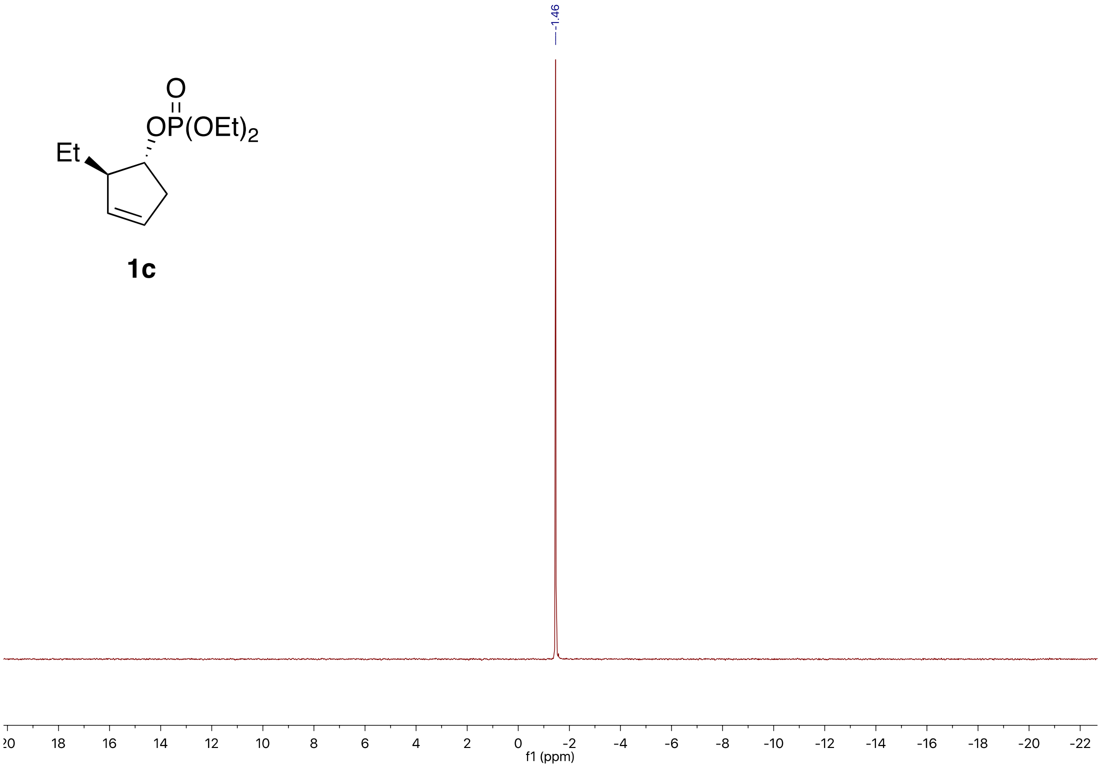

HPLC Trace

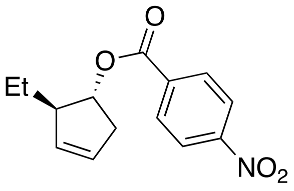

1c'

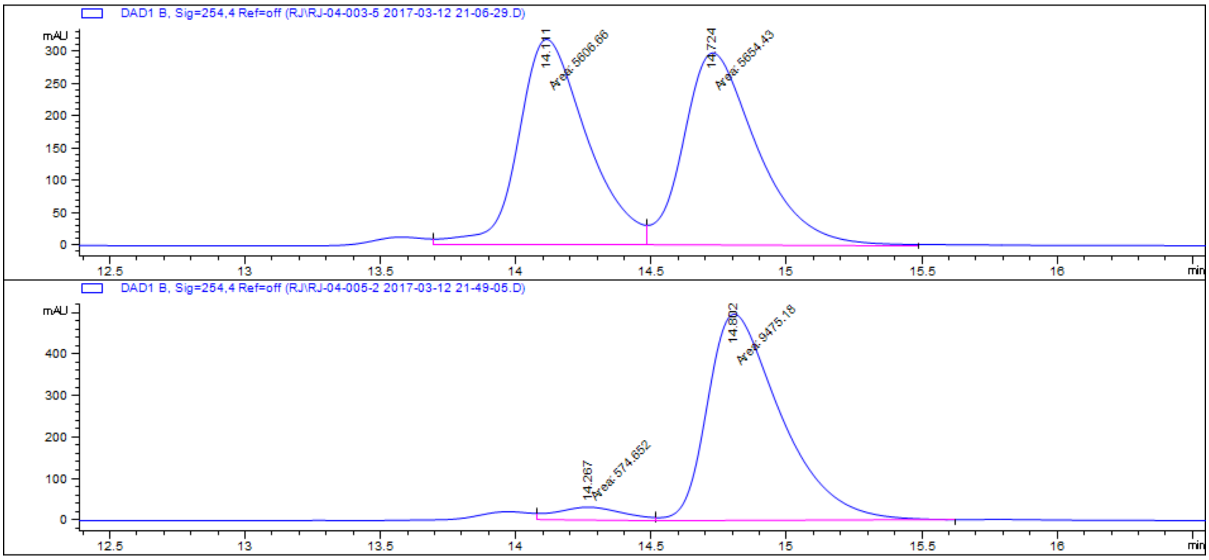

Supplementary Figure 31 - Diethyl ((1*R*,2*R*)-2-ethylcyclohex-3-en-1-yl) phosphate (**2c**) ^{1}H NMR (500 MHz, $CDCl_3$)

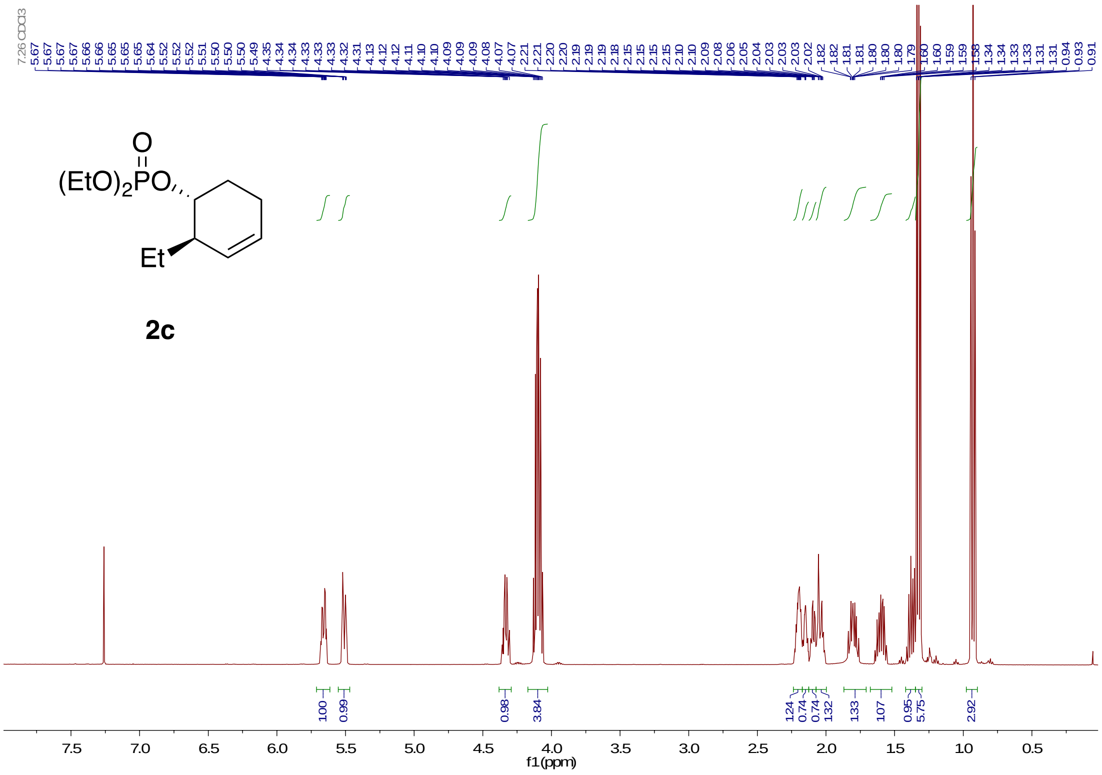

^{13}C NMR (126 MHz, $CDCl_3$)

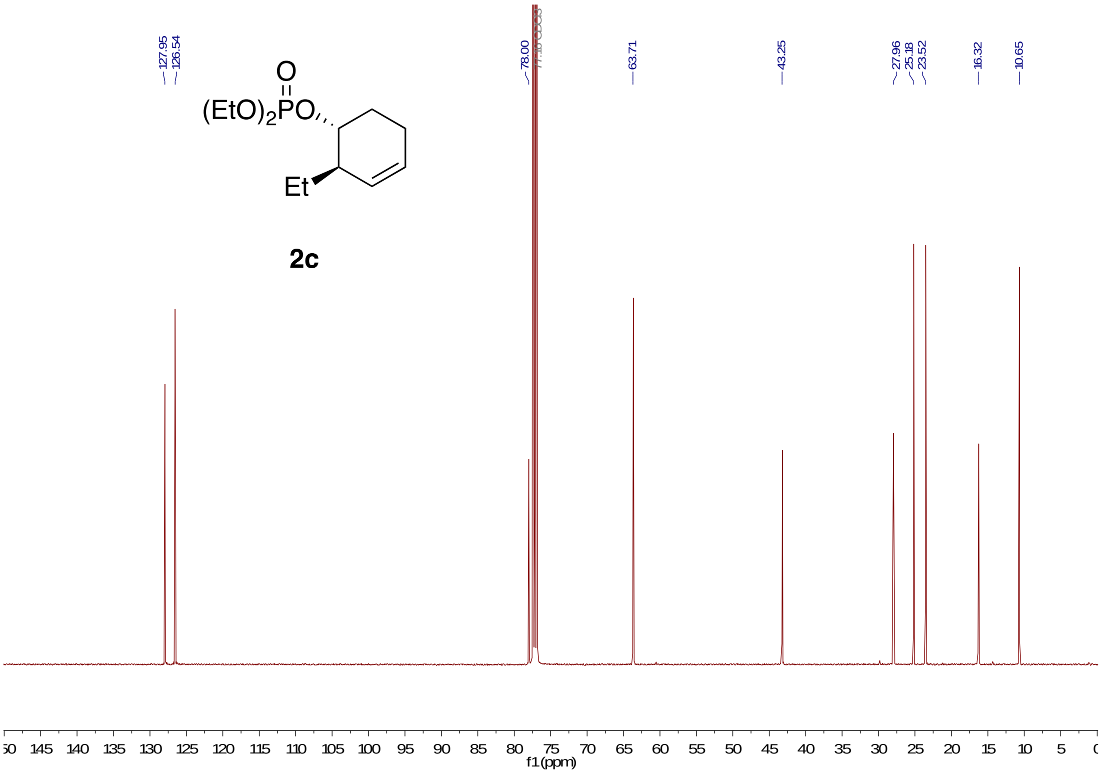

^{31}P NMR (202 MHz, CDCl$_3$)

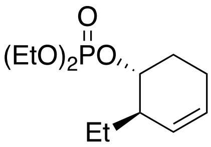

2c

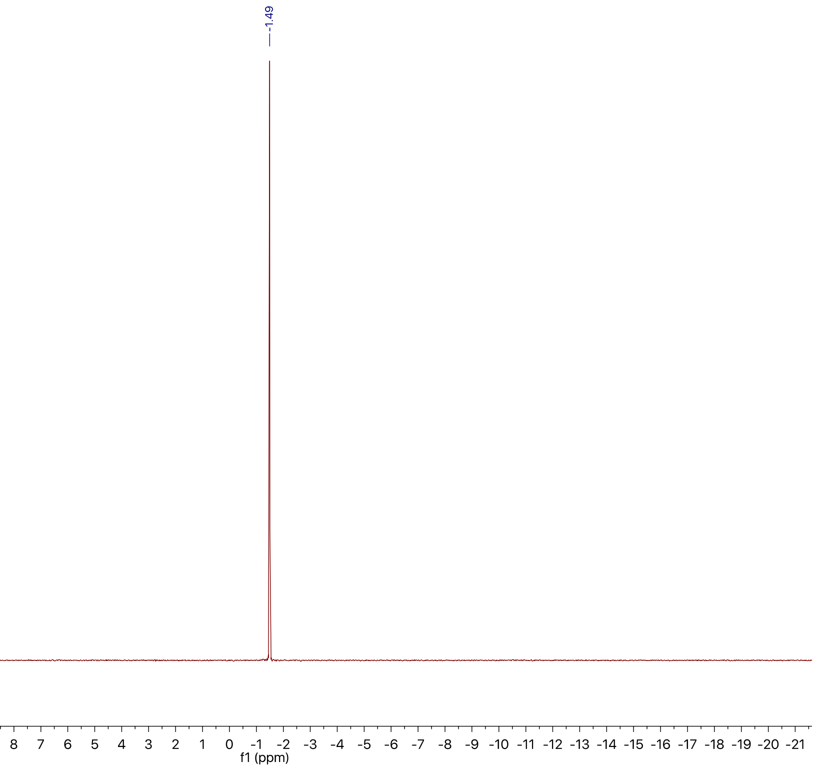

HPLC Trace

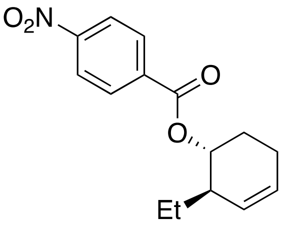

2c'

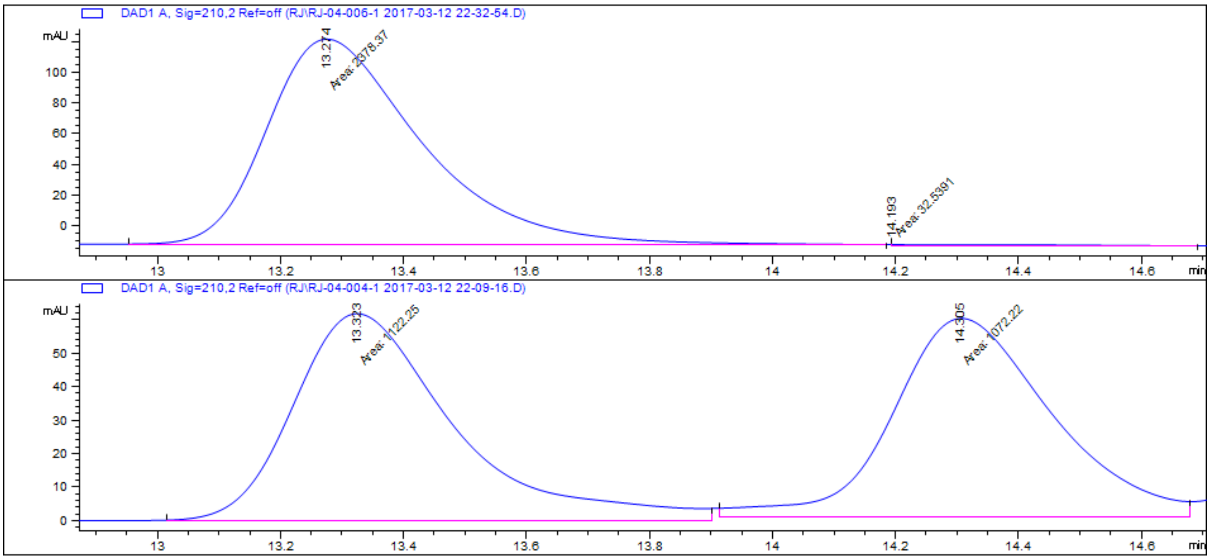

Supplementary Figure 32 - Diethyl ((1*R*,2*R*)-2-hexylcyclopent-3-en-1-yl) phosphate (**1d**) ^{1}H NMR (400 MHz, $CDCl_3$)

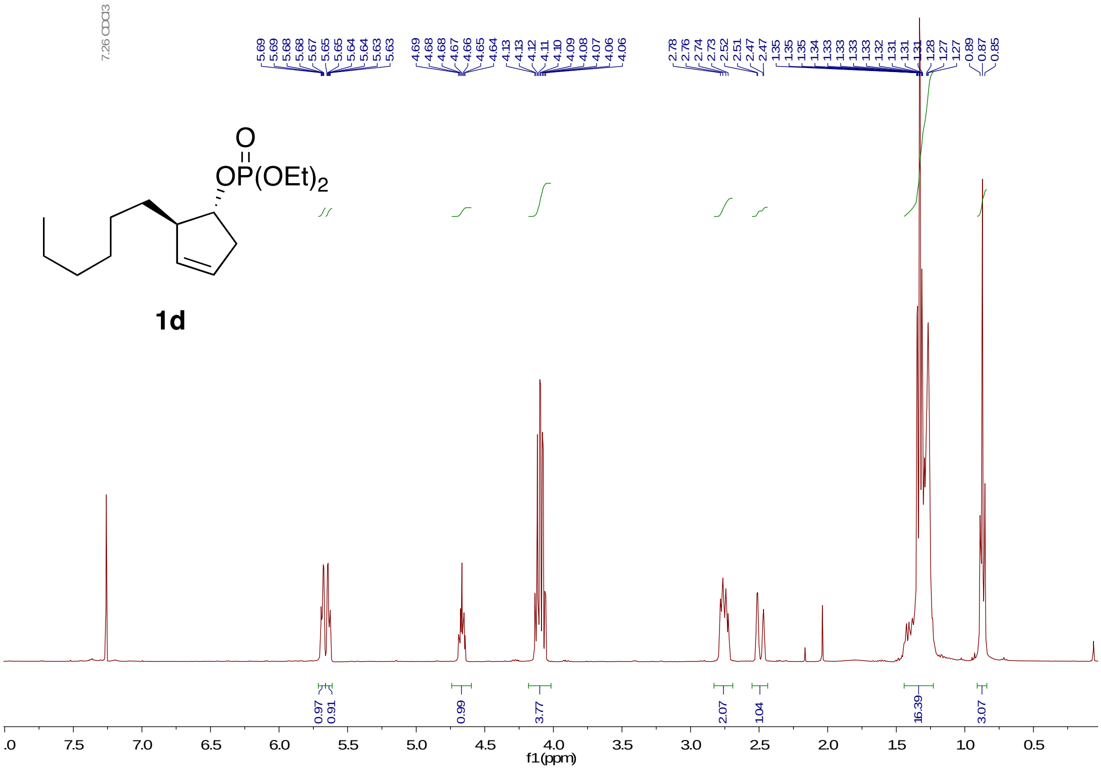

^{13}C NMR (101 MHz, $CDCl_3$)

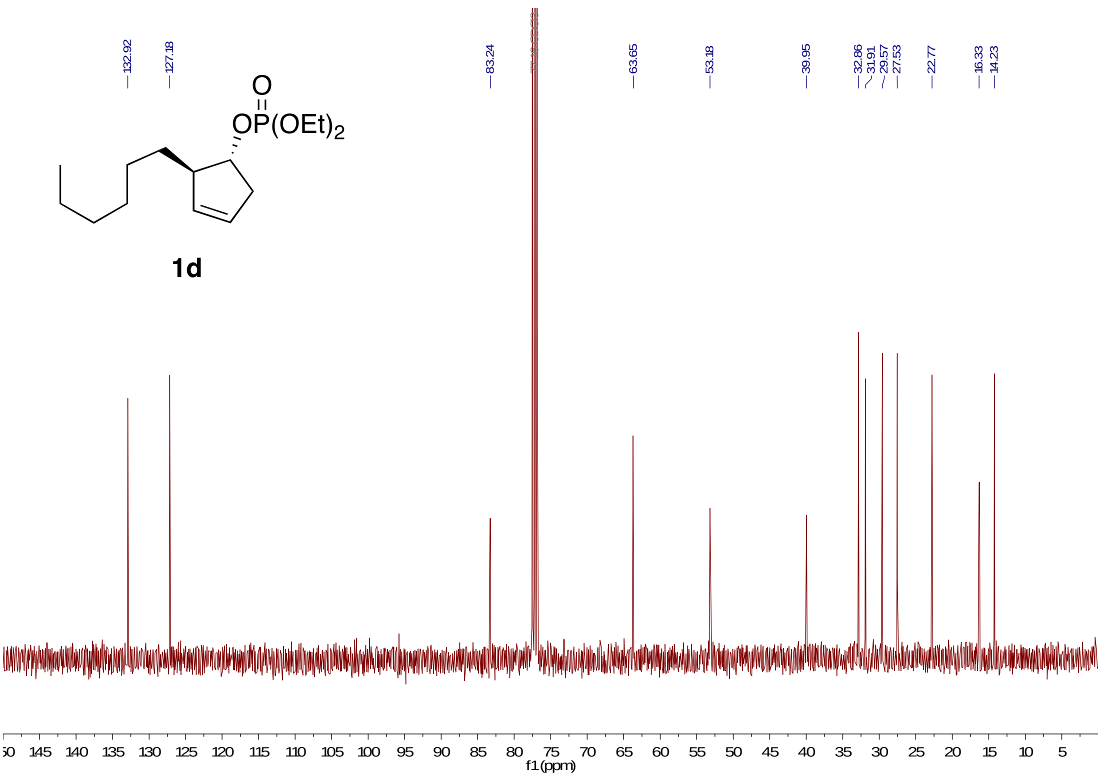

^{31}P NMR (162 MHz, $CDCl_3$)

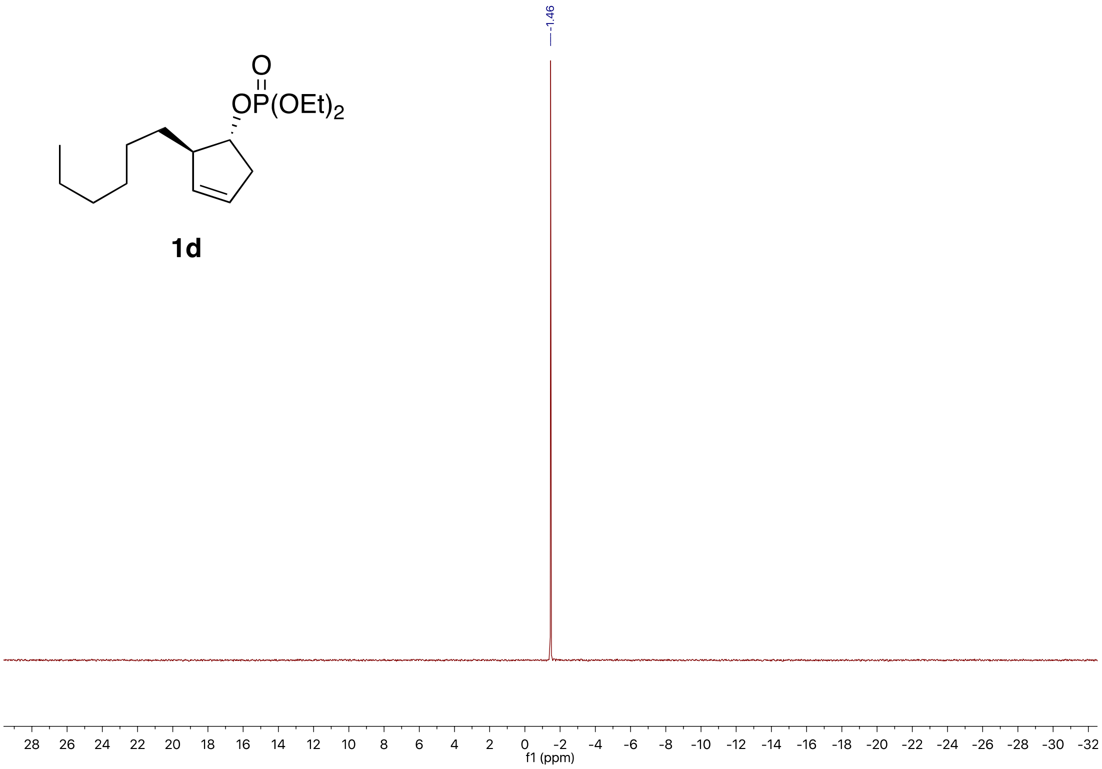

GC Trace

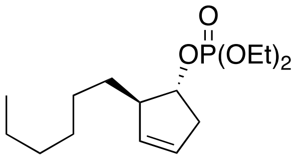

1d

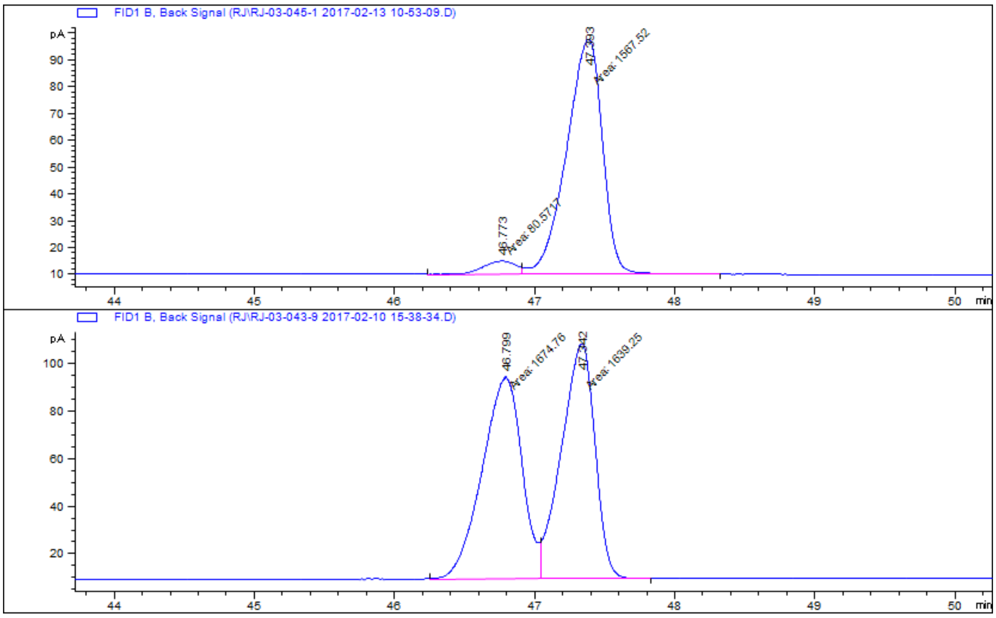

Supplementary Figure 33 - Diethyl ((1*R*,2*R*)-2-hexylcyclohex-3-en-1-yl) phosphate (**2d**) ^{1}H NMR (400 MHz, $CDCl_3$)

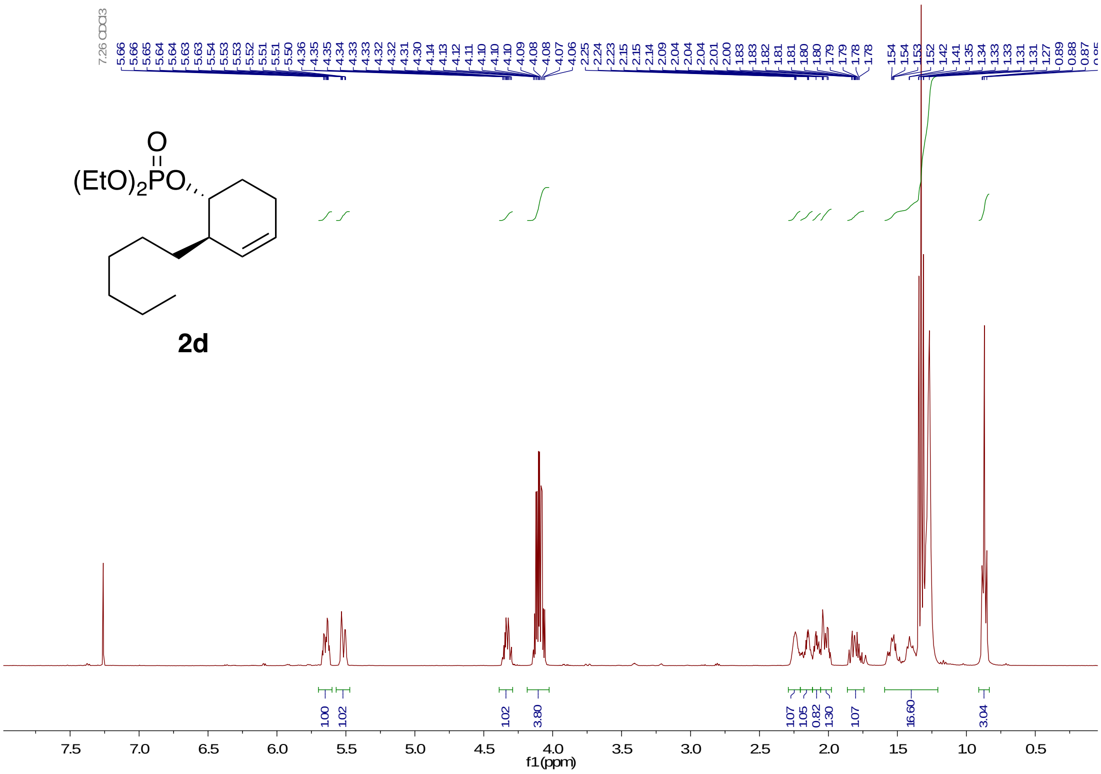

^{13}C NMR (101 MHz, $CDCl_3$)

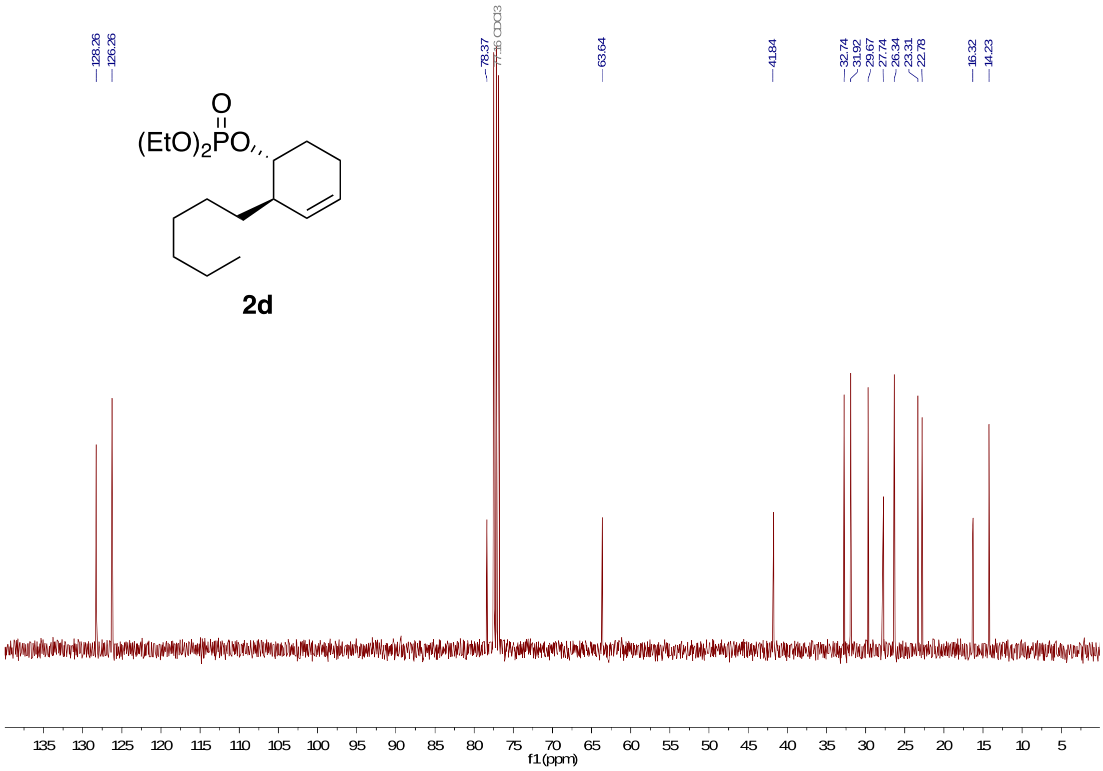

^{31}P NMR (162 MHz, $CDCl_3$)

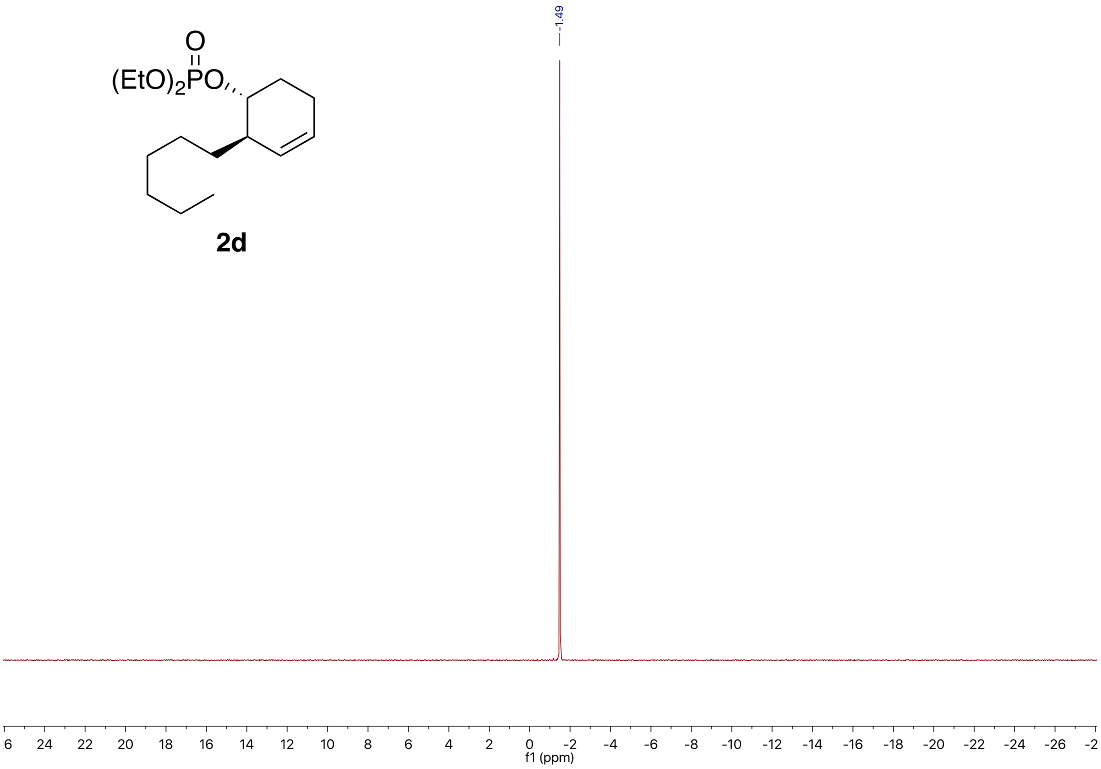

GC Trace

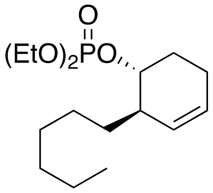

2d

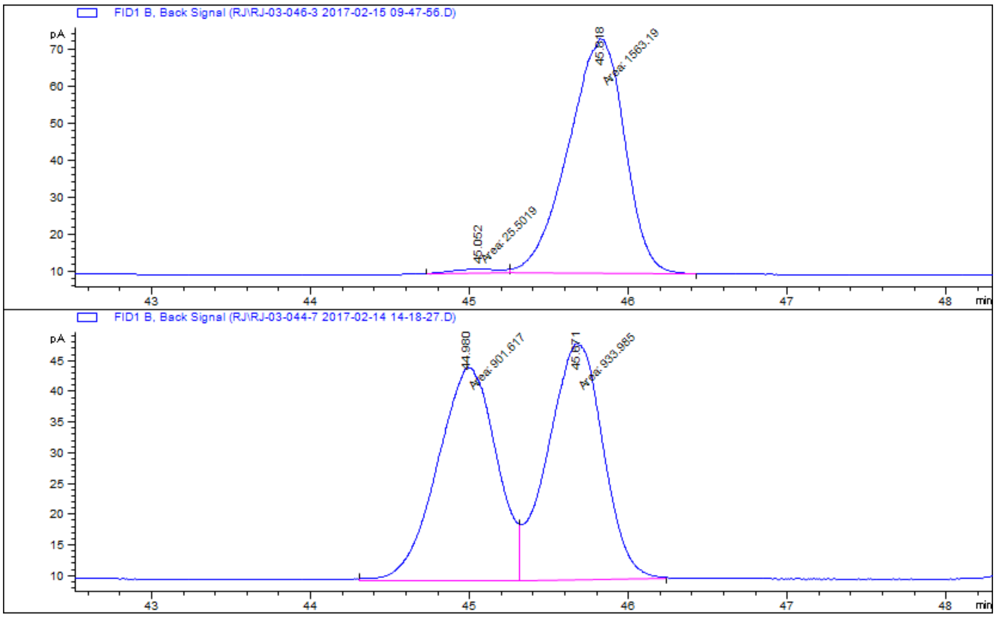

Supplementary Figure 34 - Diethyl ((1*R*,2*R*)-2-tetradecylcyclopent-3-en-1-yl) phosphate (**1e**) ^{1}H NMR (400 MHz, $CDCl_3$)

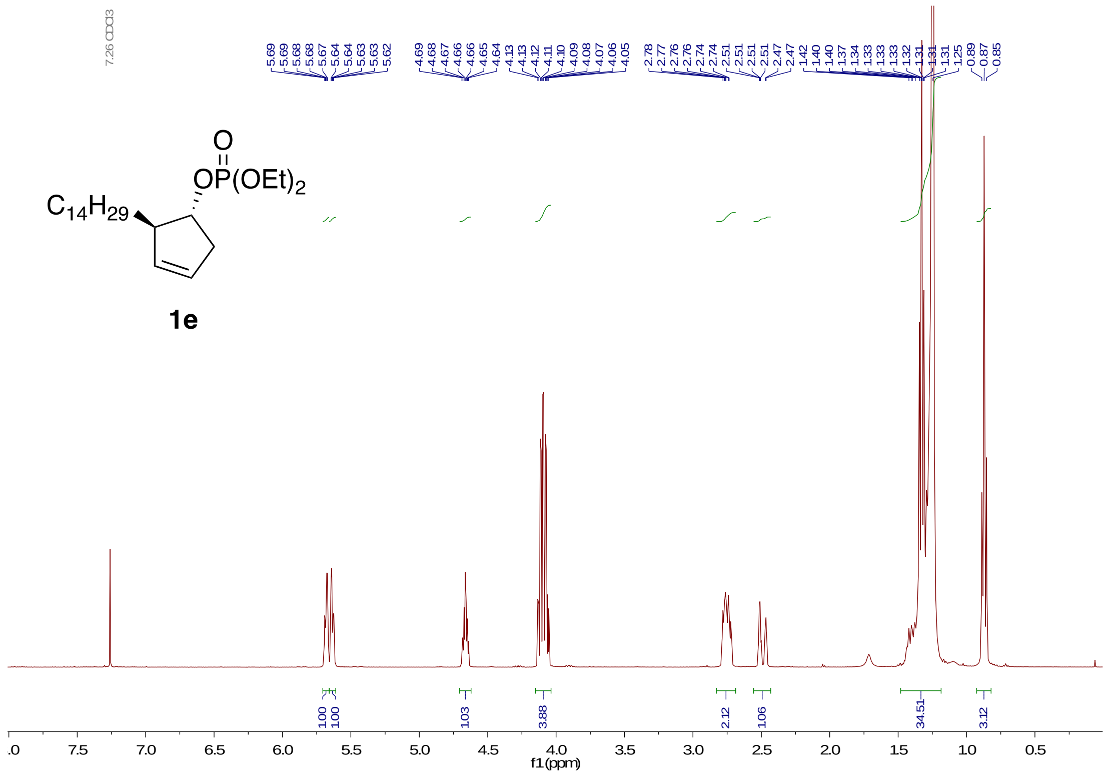

^{13}C NMR (101 MHz, $CDCl_3$)

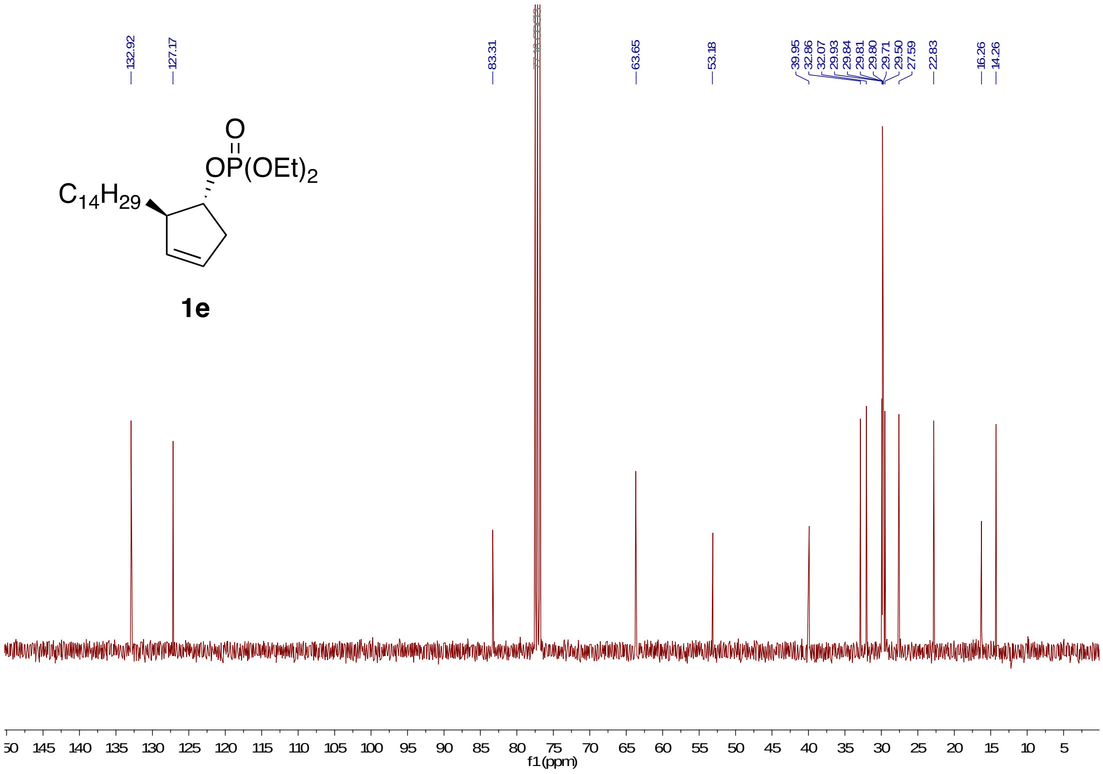

^{31}P NMR (162 MHz, $CDCl_3$)

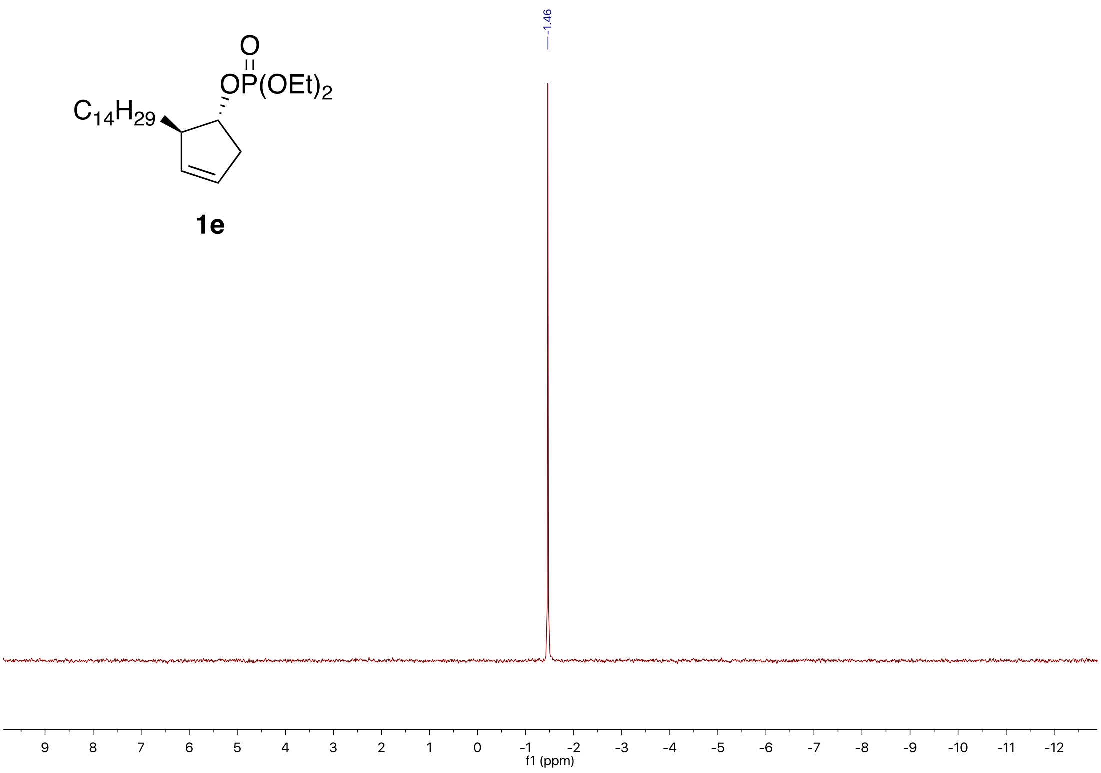

HPLC Trace

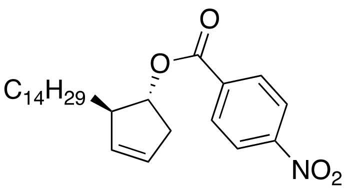

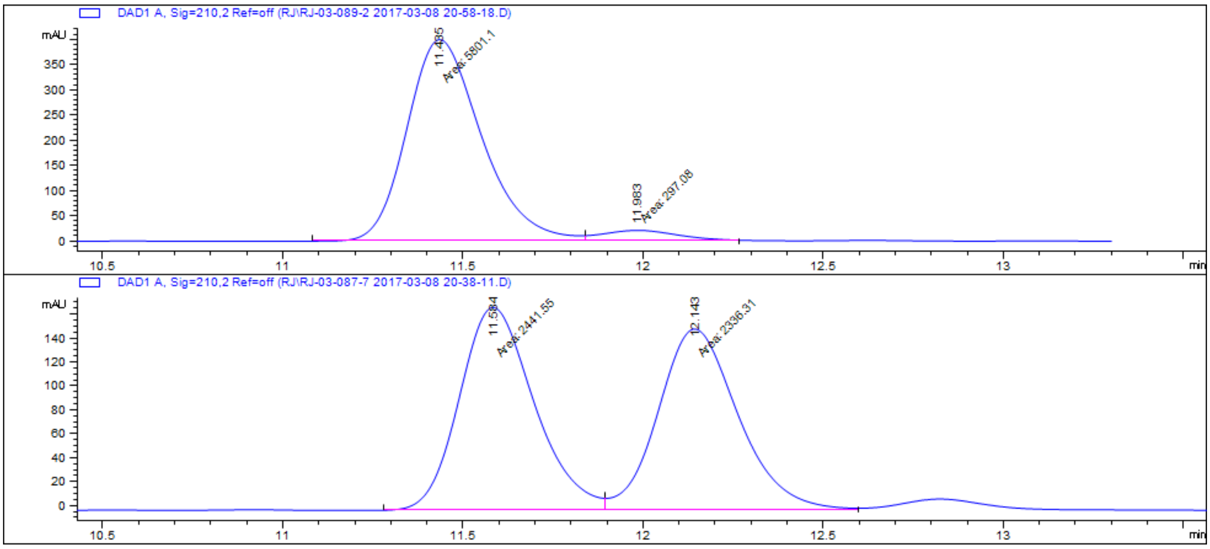

Supplementary Figure 35 - Diethyl ((1*R*,2*R*)-2-tetradecylcyclohex-3-en-1-yl) phosphate (**2e**) ^{1}H NMR (400 MHz, $CDCl_3$)

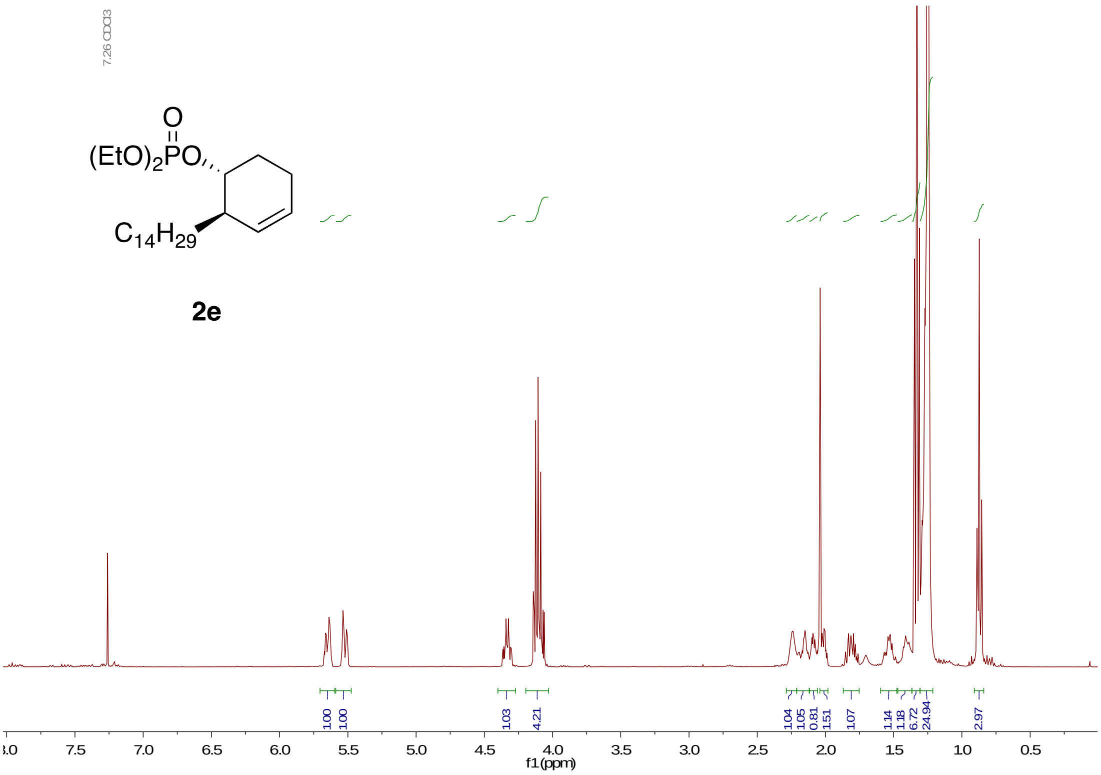

^{13}C NMR (101 MHz, $CDCl_3$)

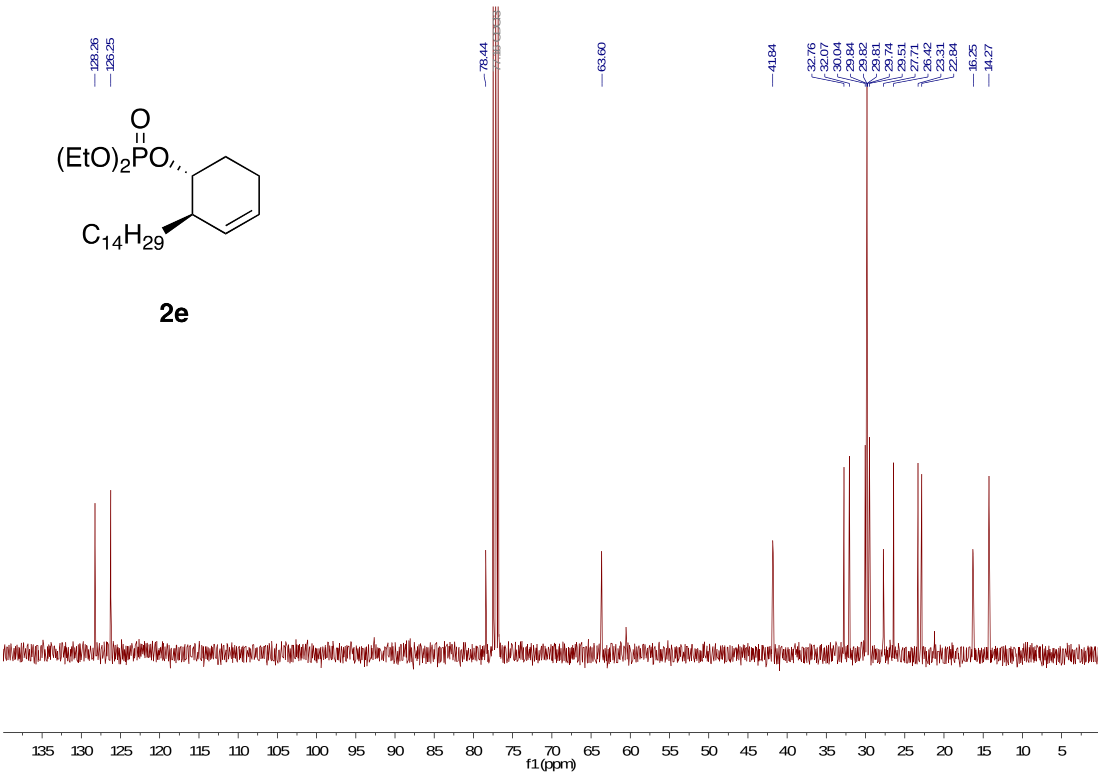

^{31}P NMR (162 MHz, $CDCl_3$)

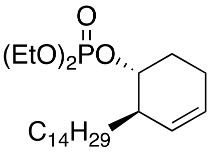

2e

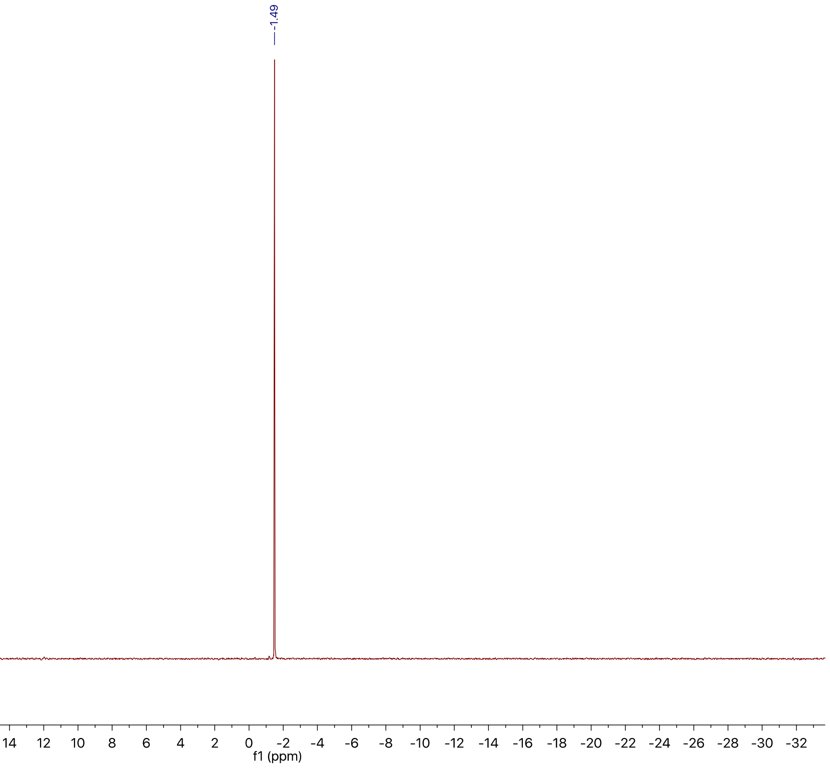

HPLC Trace

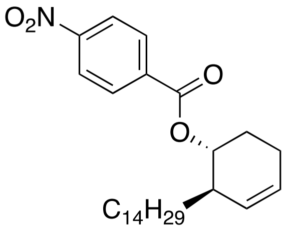

2e’

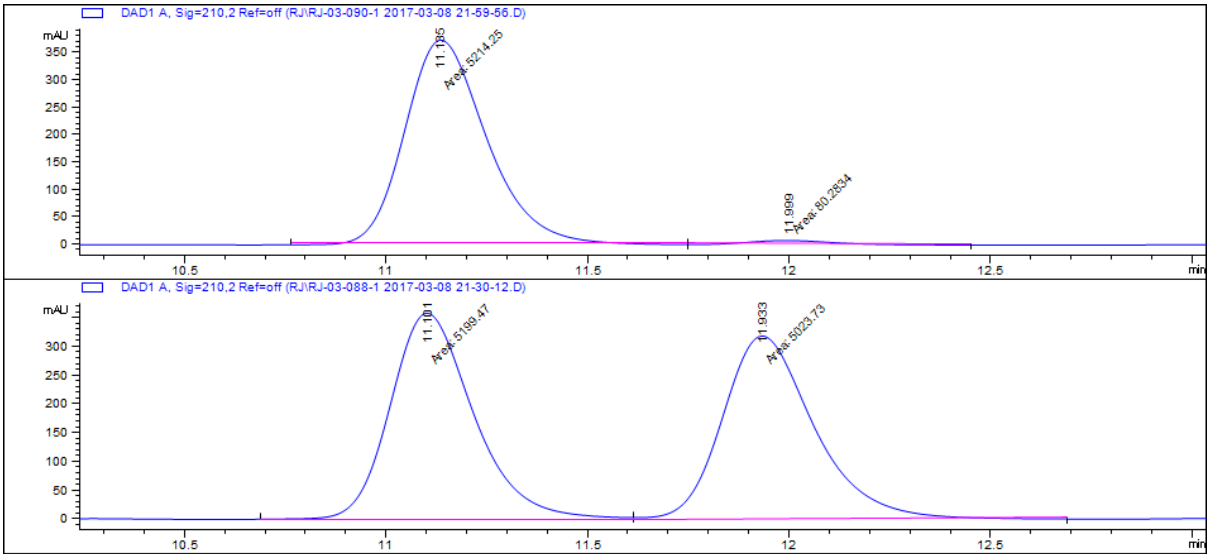

Supplementary Figure 36 - Diethyl ((1*R*,2*R*)-2-isopentylcyclopent-3-en-1-yl) phosphate (**1f**) ^{1}H NMR (400 MHz, $CDCl_3$)

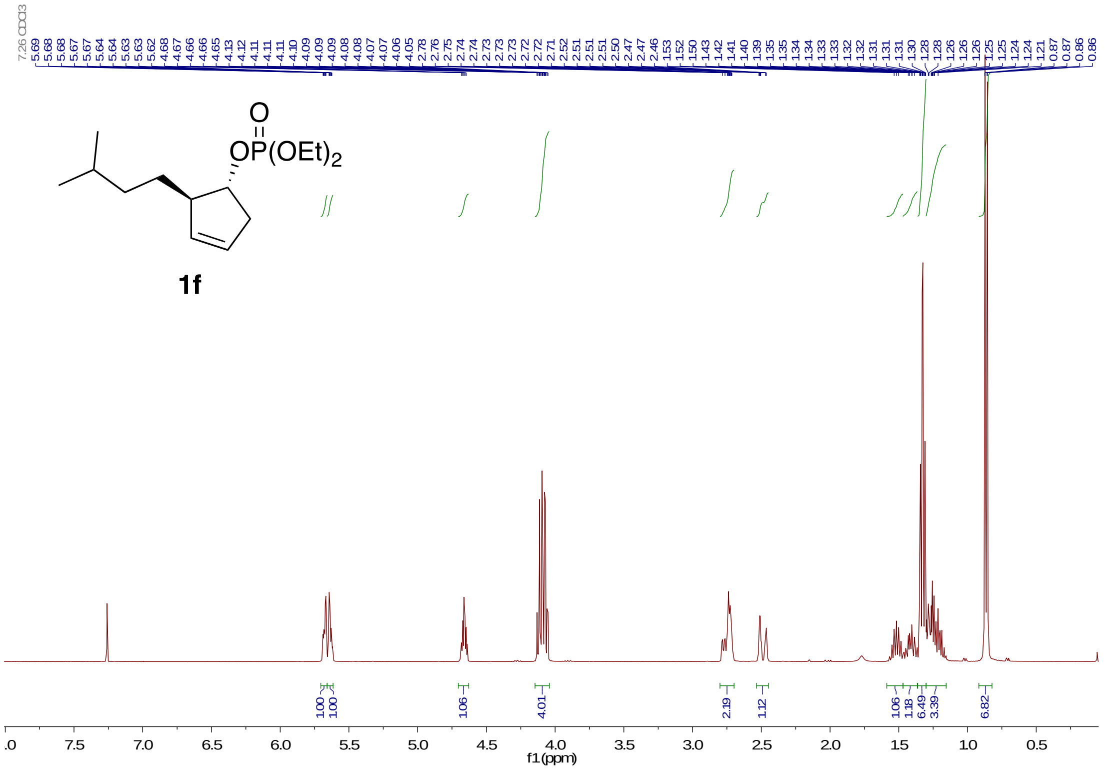

^{13}C NMR (101 MHz, $CDCl_3$)

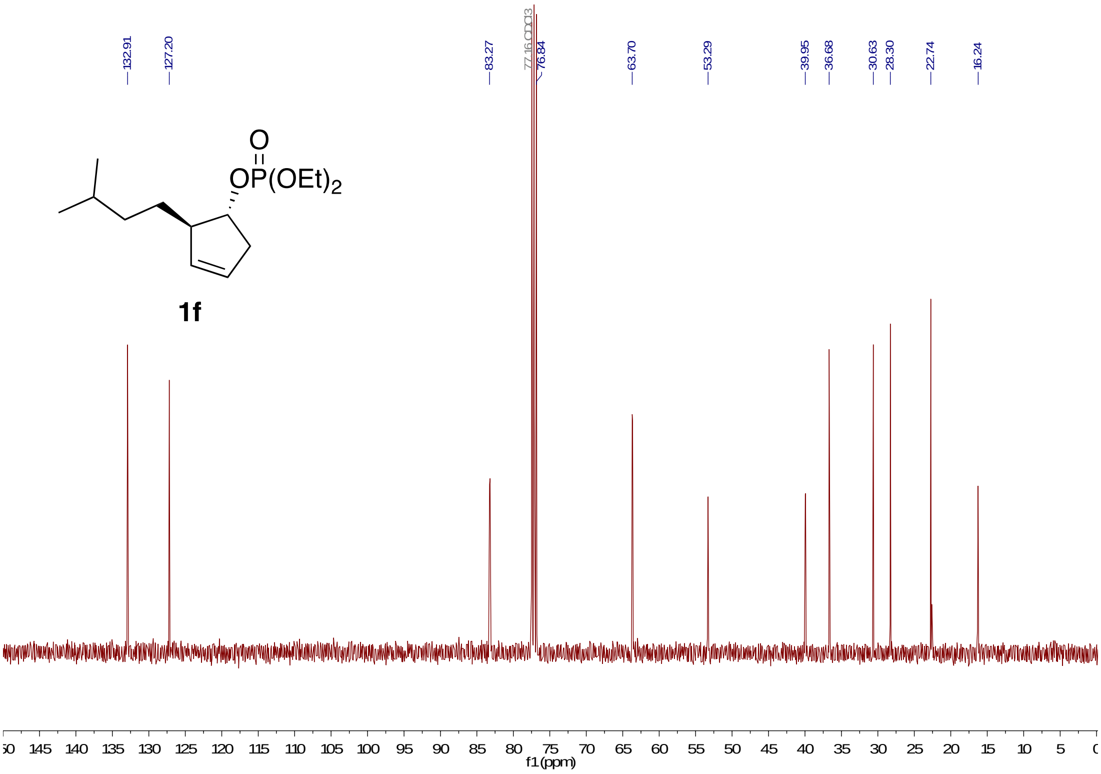

^{31}P NMR (162 MHz, $CDCl_3$)

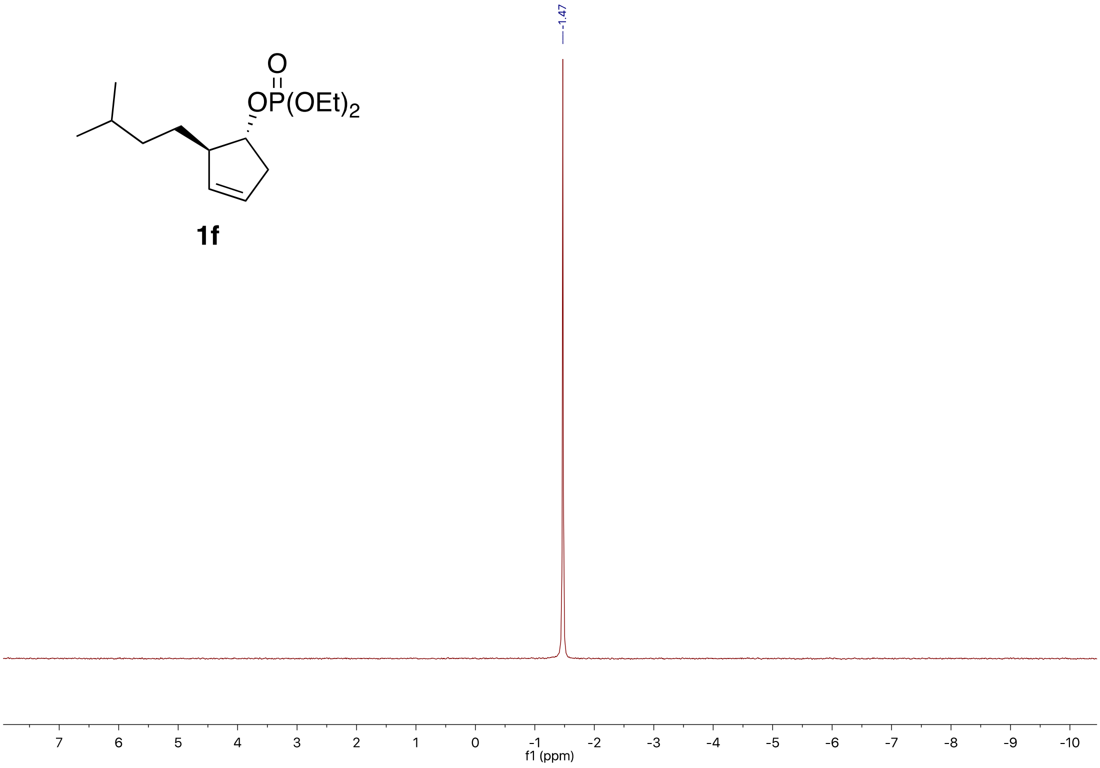

GC Trace

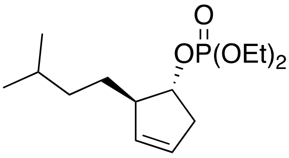

1f

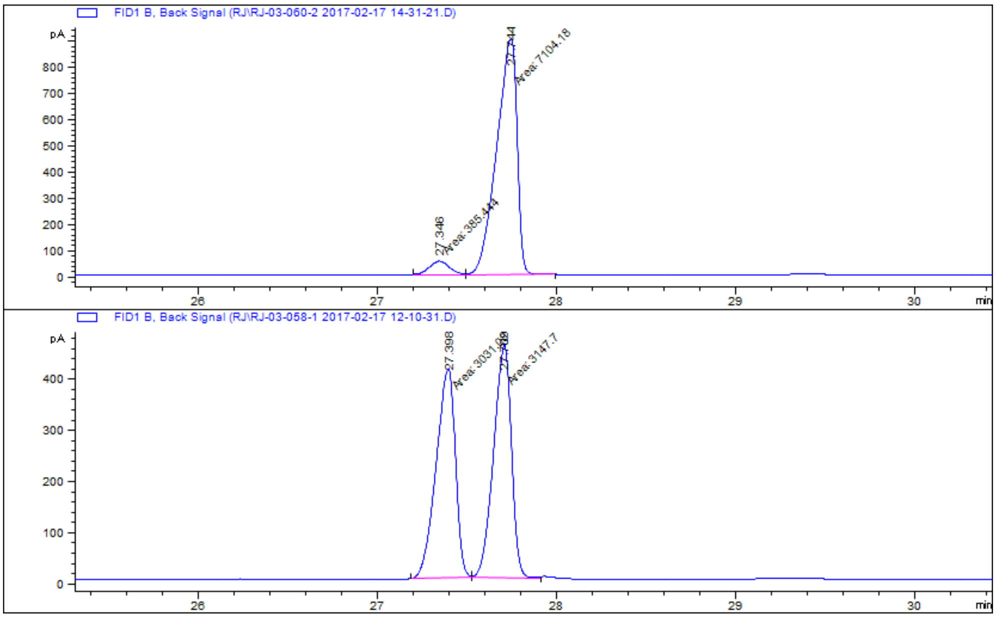

Supplementary Figure 37 - Diethyl ((1*R*,2*R*)-2-isopentylcyclohex-3-en-1-yl) phosphate (**10b**) ^{1}H NMR (400 MHz, $CDCl_3$)

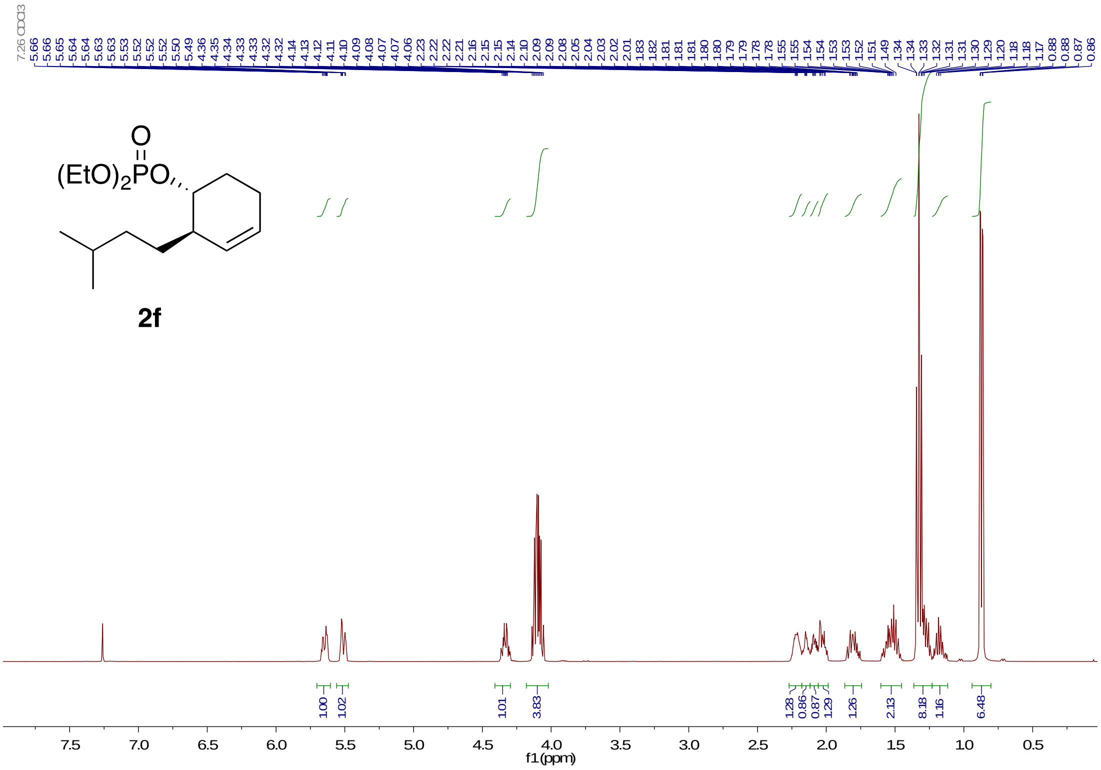

^{13}C NMR (101 MHz, $CDCl_3$)

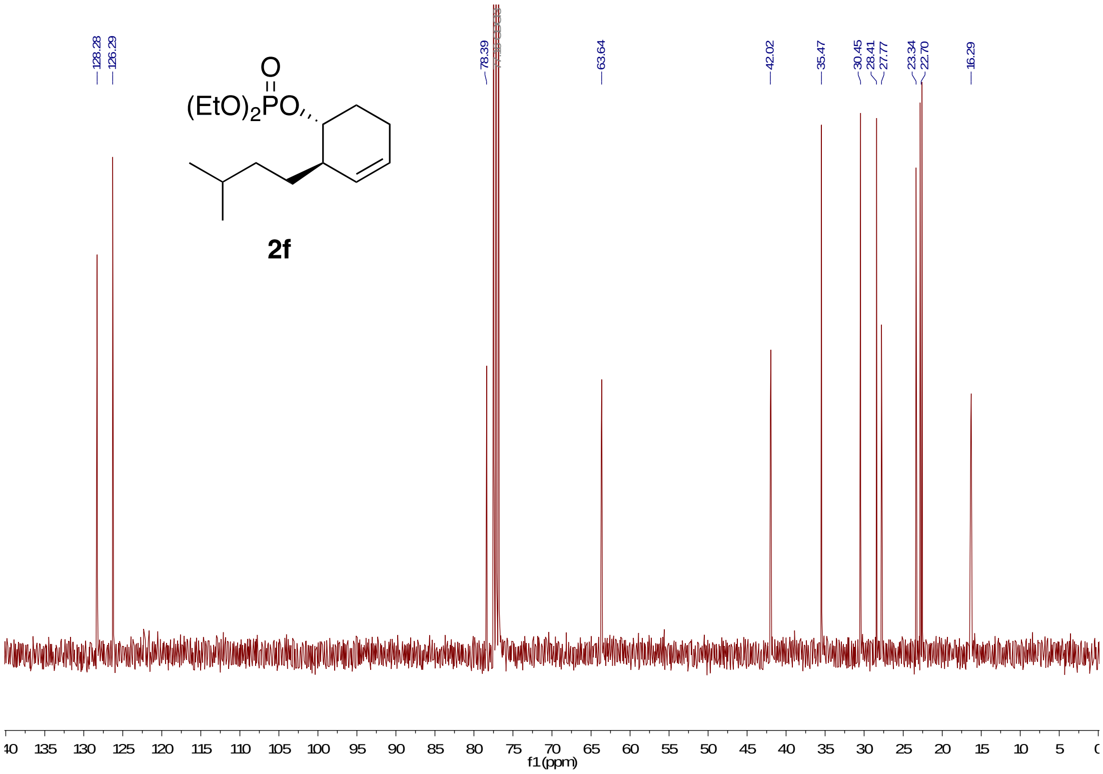

^{31}P NMR (162 MHz, CDCl$_3$)

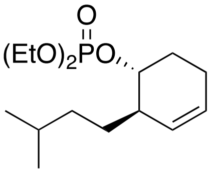

2f

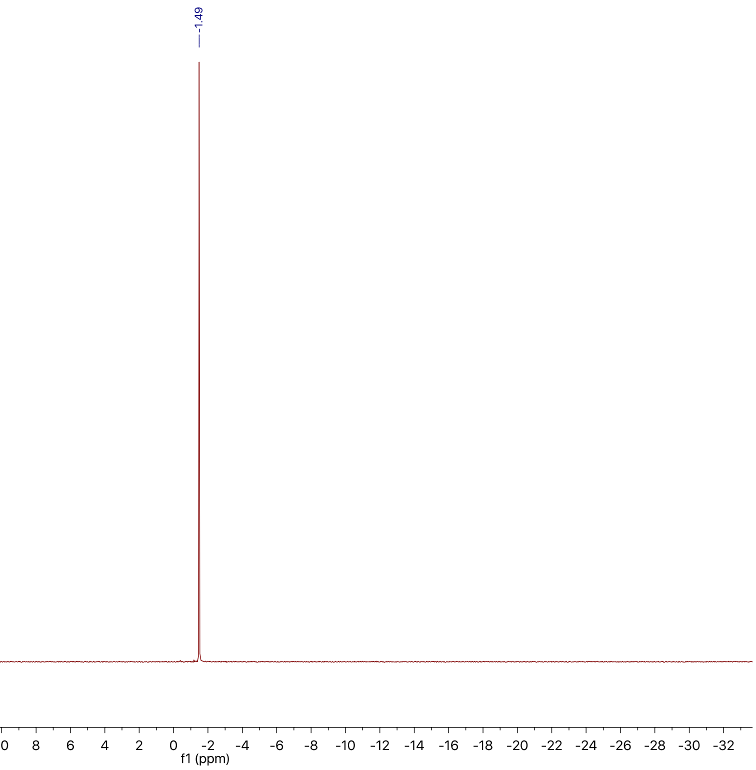

GC Trace

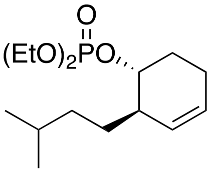

2f

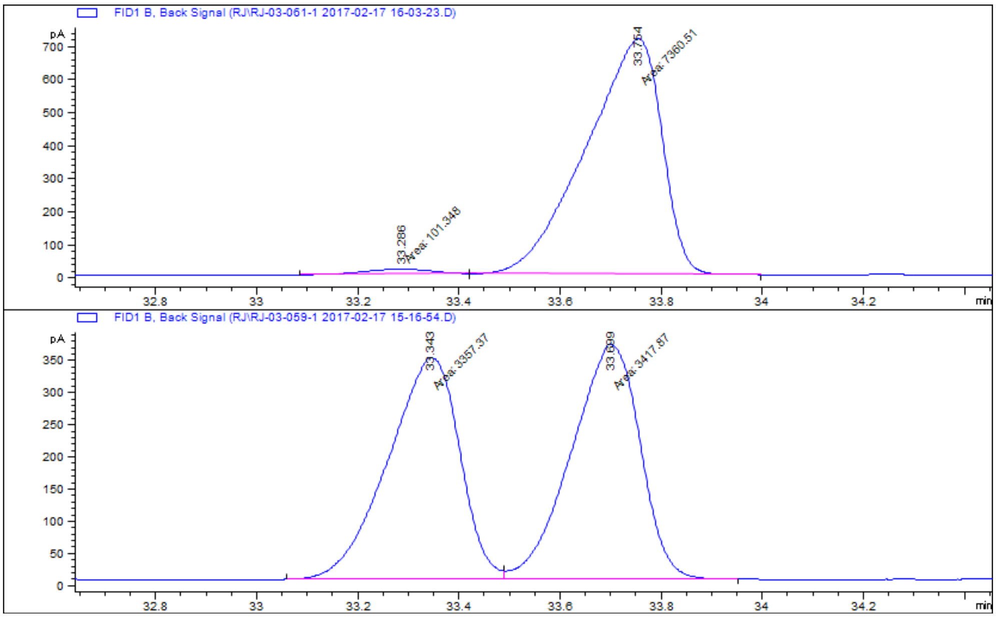

Supplementary Figure 38 - Diethyl (1*R*,2*R*)-2-(3,3-dimethylbutyl)cyclopent-3-en-1-yl) phosphate (**1g**) ^{1}H NMR (400 MHz, CDCl$_3$)

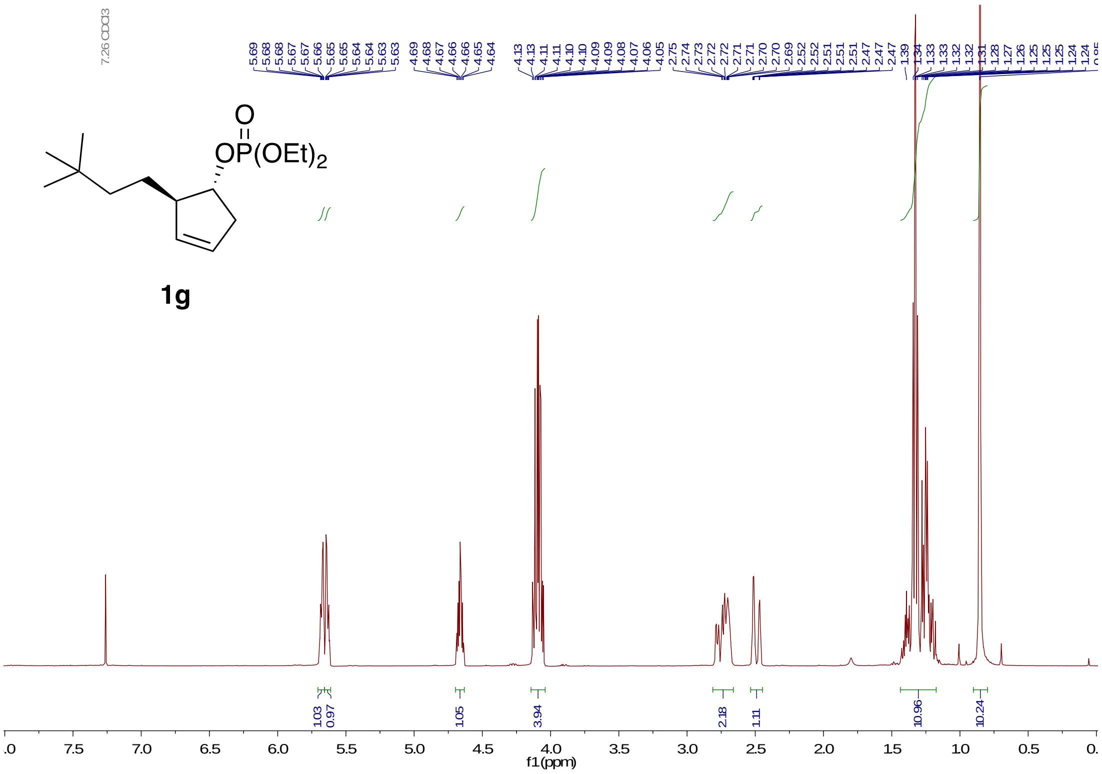

^{13}C NMR (101 MHz, CDCl$_3$)

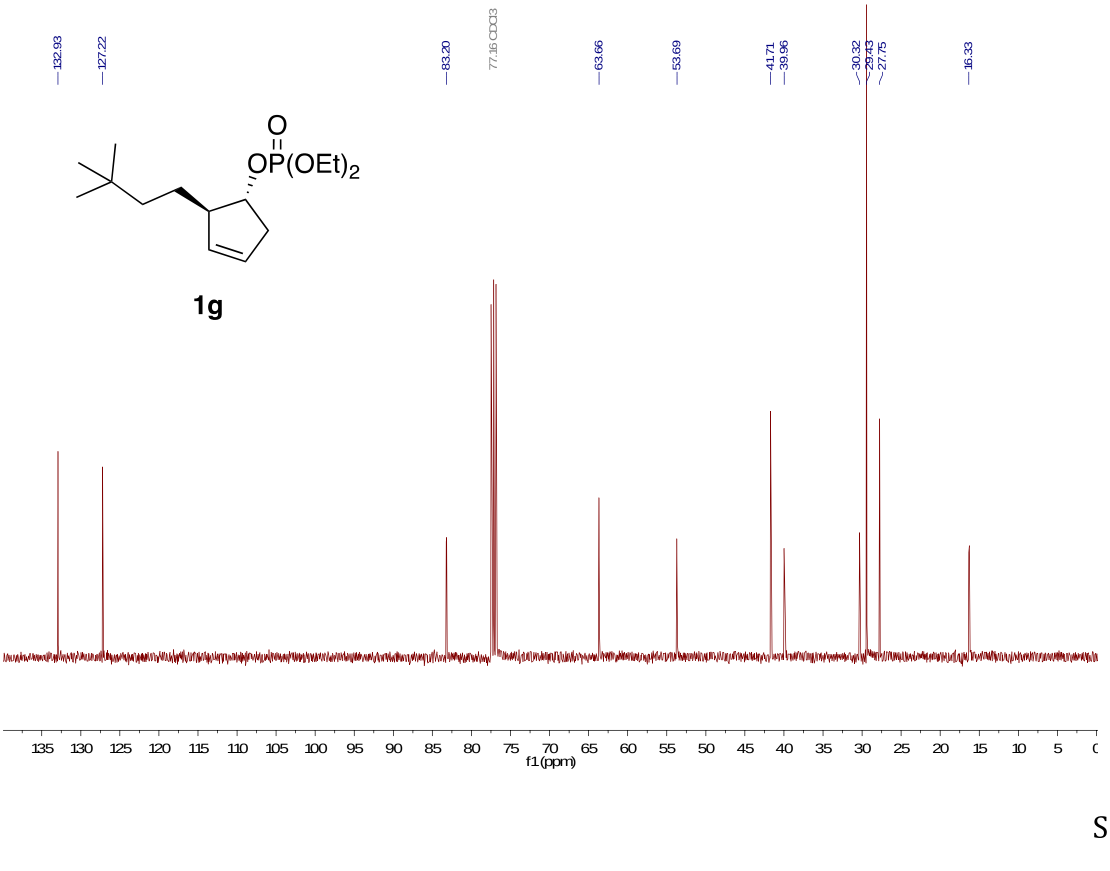

^{31}P NMR (162 MHz, $CDCl_3$)

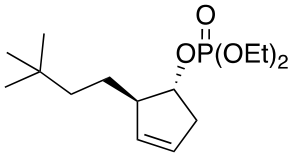

1g

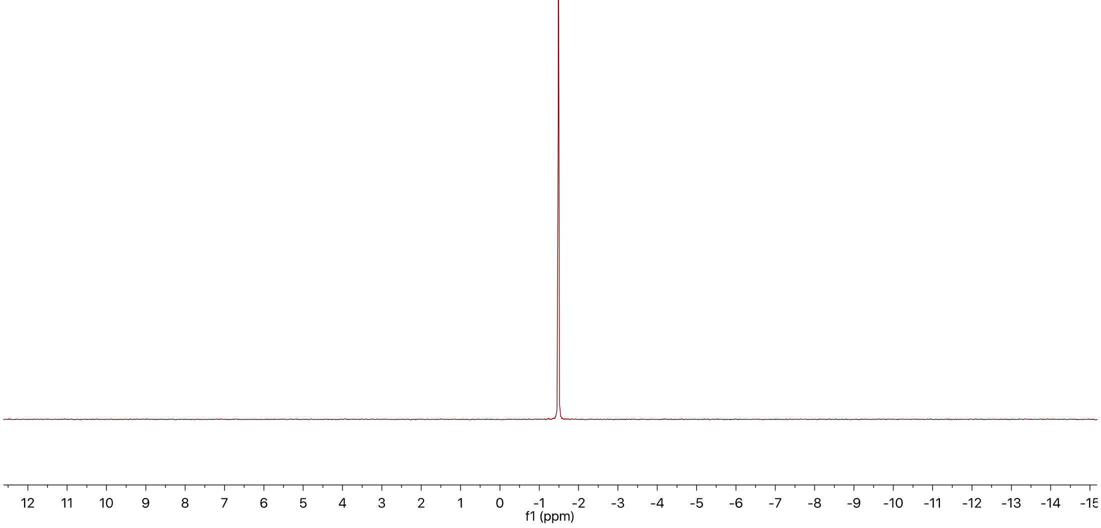

GC Trace

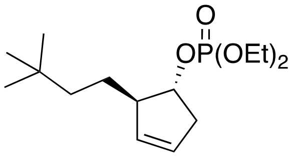

1g

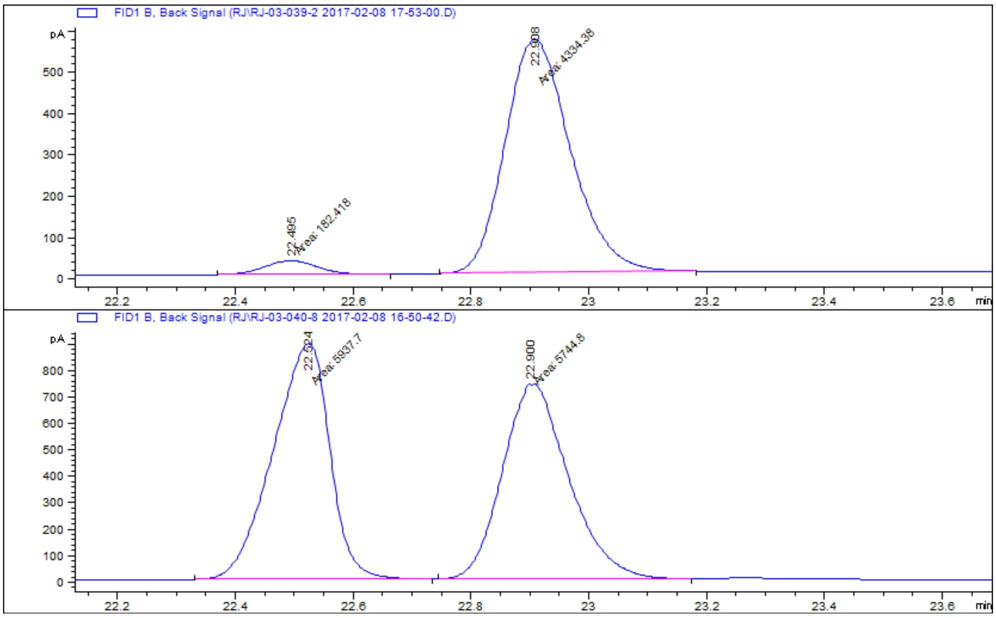

Supplementary Figure 39 - Diethyl (1*R*,2*R*)-2-(3,3-dimethylbutyl)cyclohex-3-en-1-yl) phosphate (**2g**) ^{1}H NMR (400 MHz, $CDCl_3$)

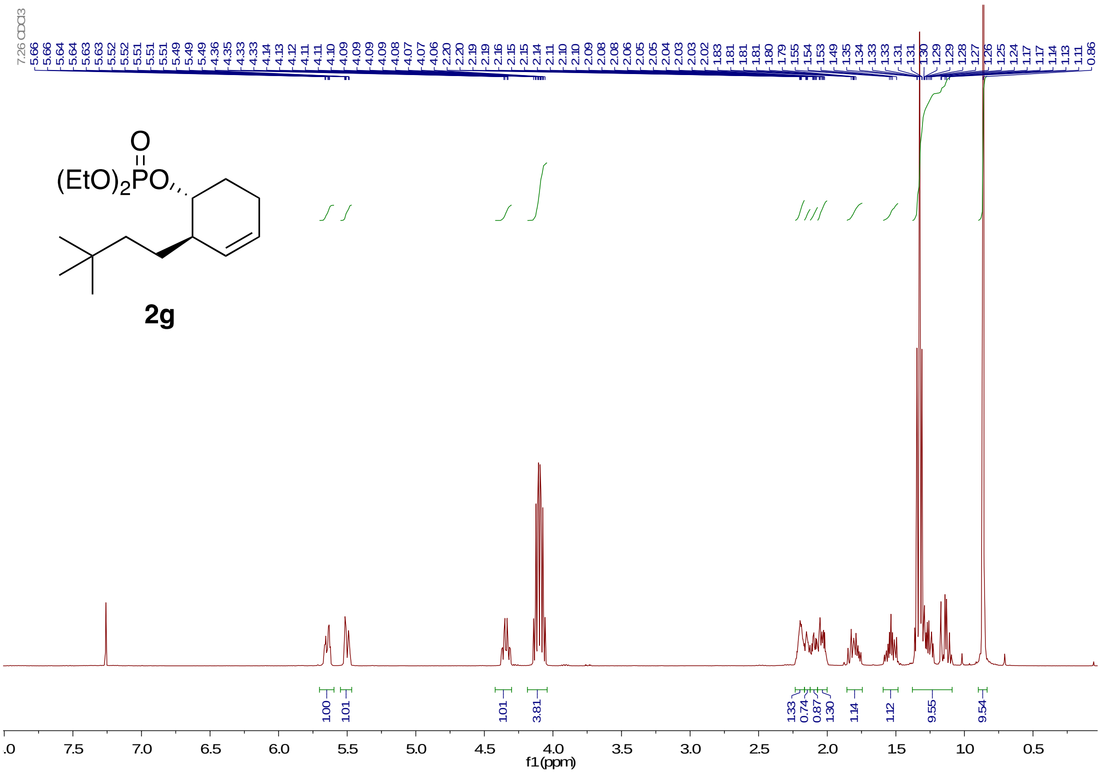

^{13}C NMR (101 MHz, $CDCl_3$)

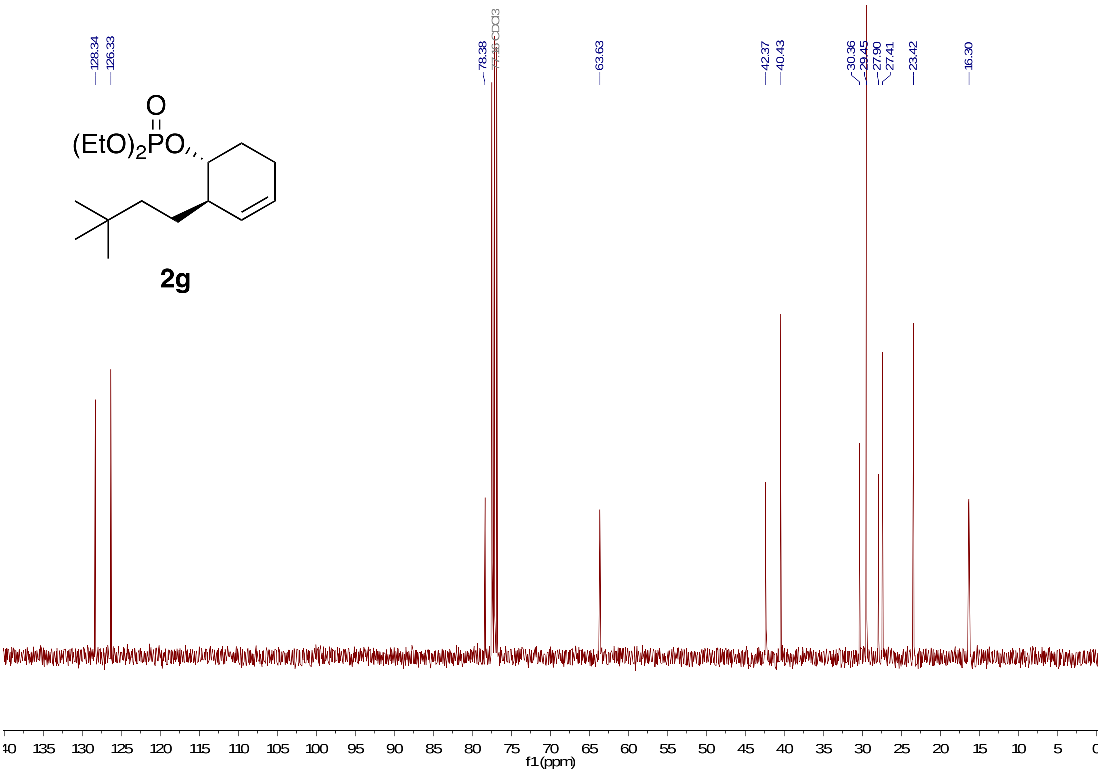

^{31}P NMR (162 MHz, CDCl$_3$)

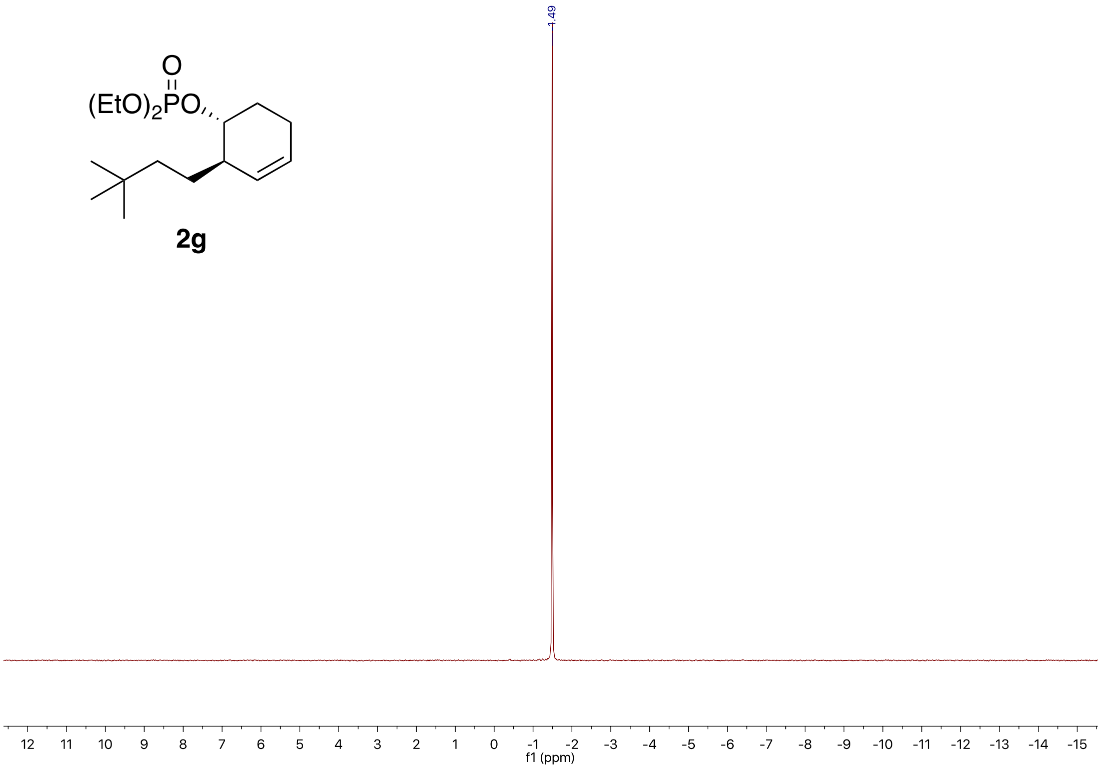

GC Trace

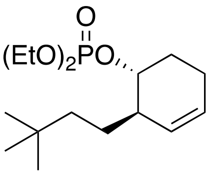

2g

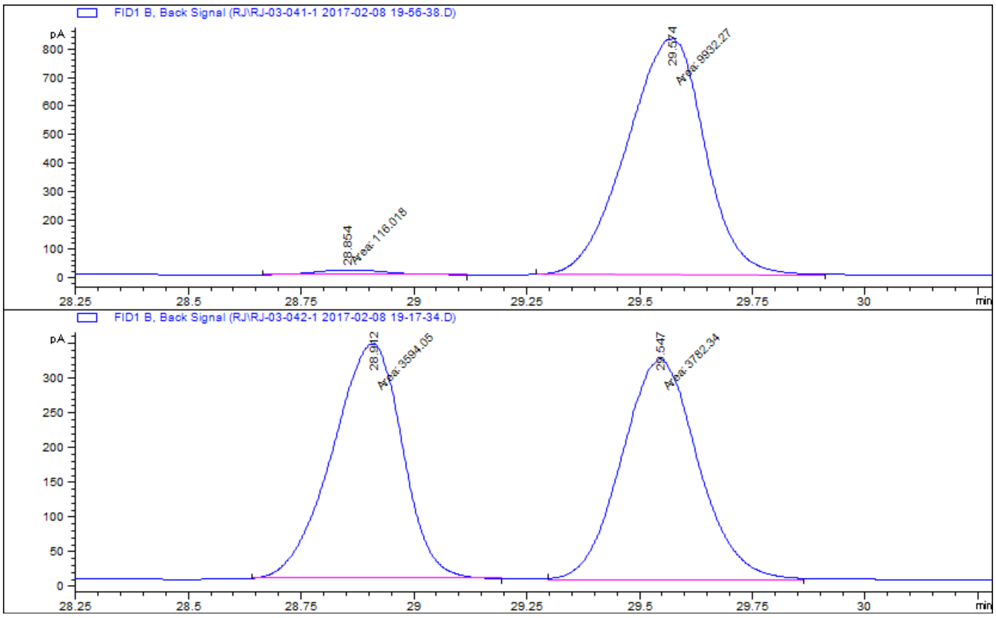

Supplementary Figure 40 - Diethyl (1*R*,2*R*)-2-(2-cyclohexylethyl)cyclopent-3-en-1-yl phosphate (**1h**) ^{1}H NMR (400 MHz, $CDCl_3$)

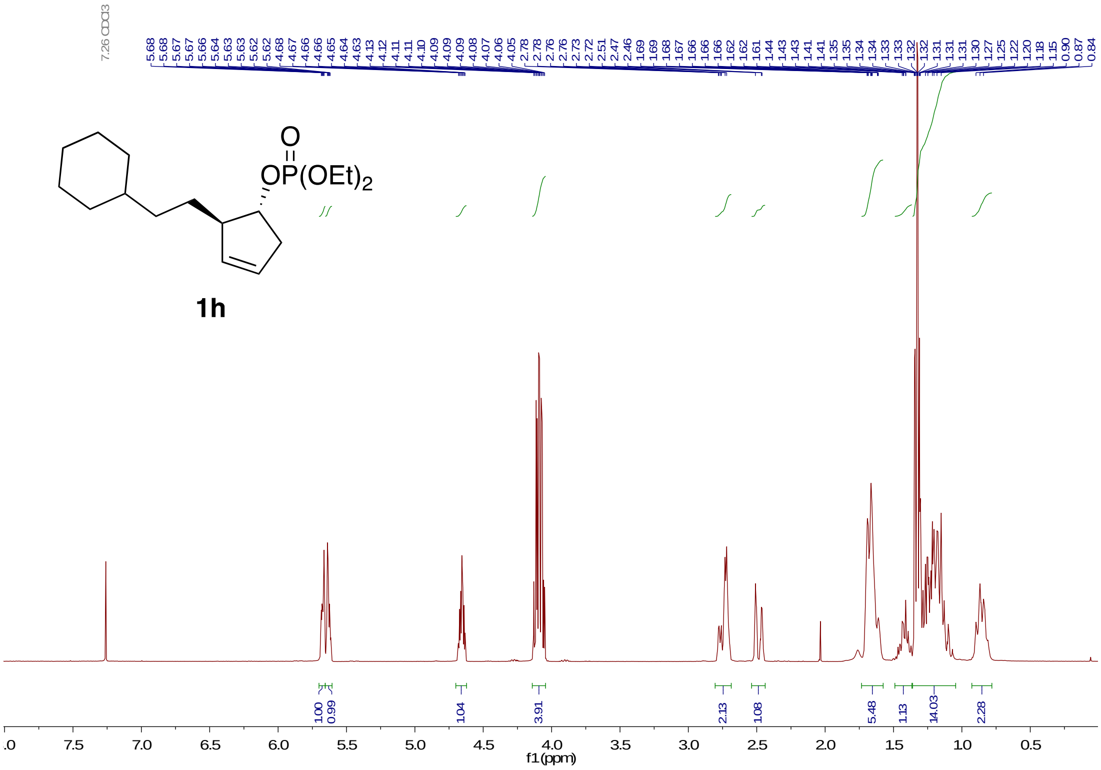

^{13}C NMR (101 MHz, $CDCl_3$)

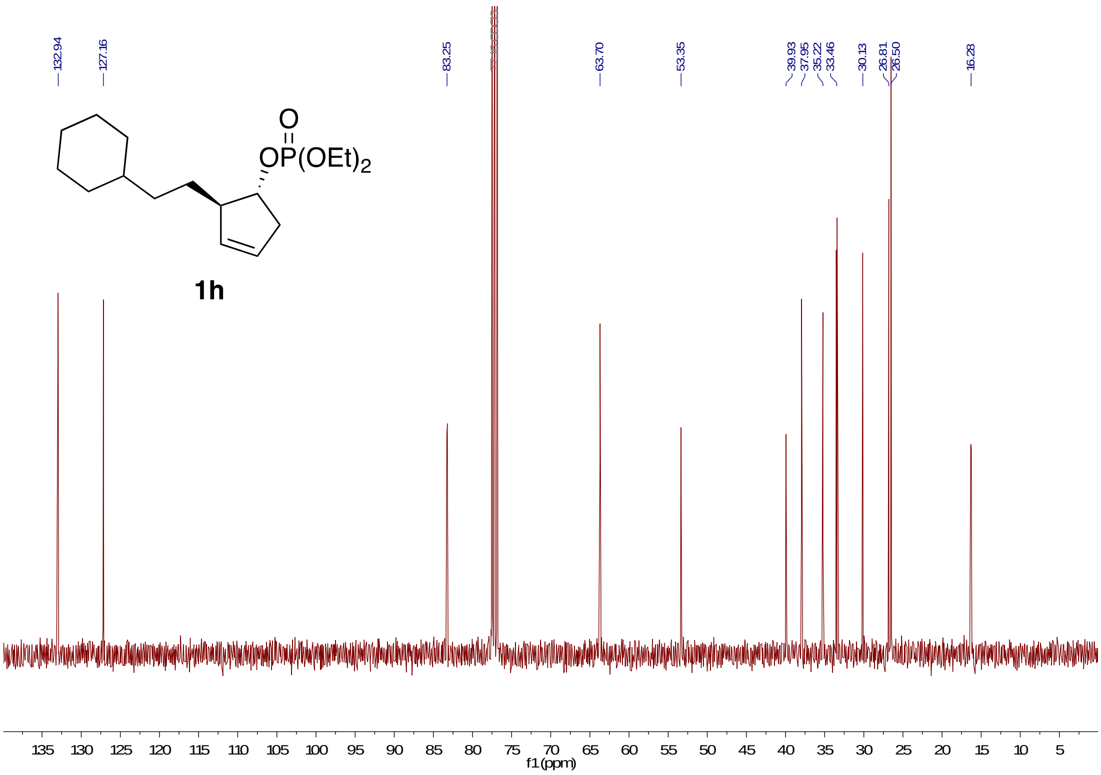

^{31}P NMR (162 MHz, $CDCl_3$)

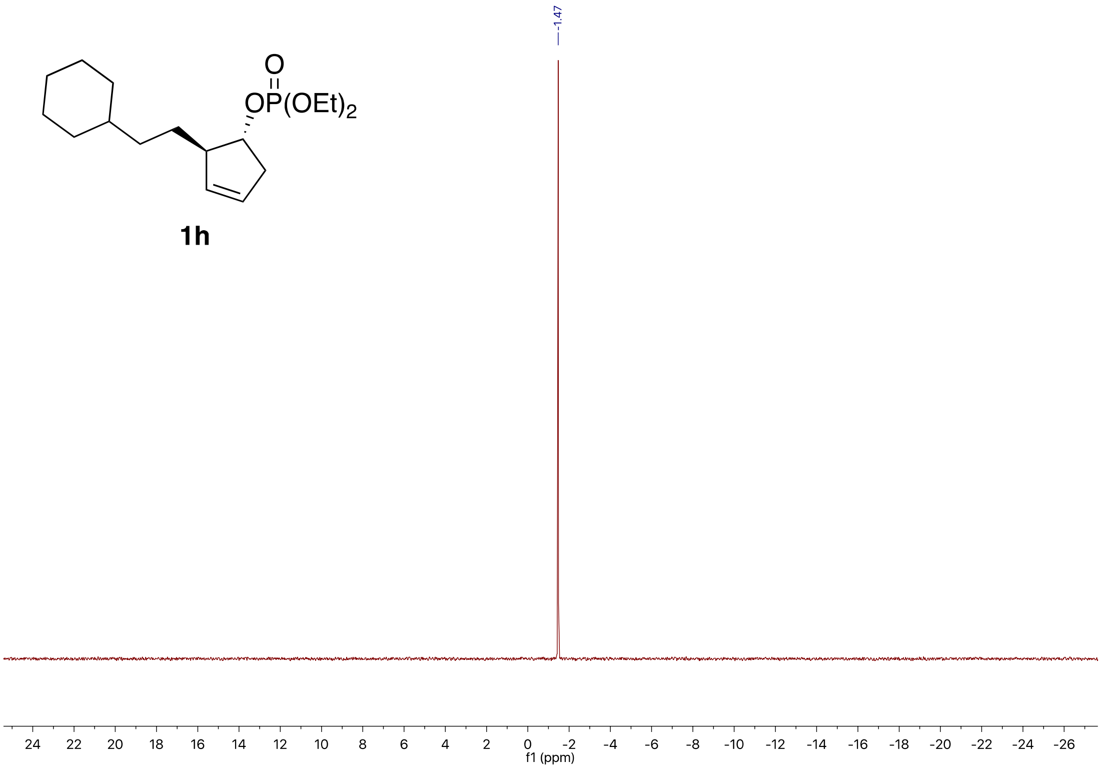

GC Trace

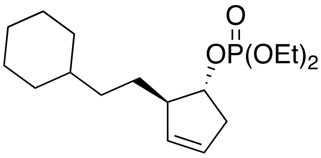

1h

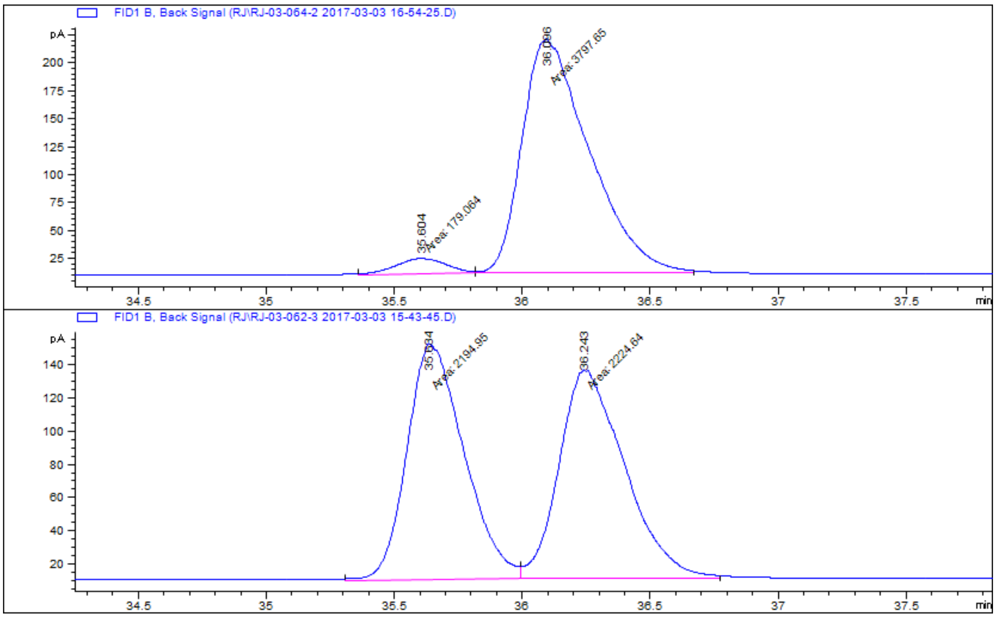

Supplementary Figure 41 - Diethyl (1*R*,2*R*)-2-(2-cyclohexylethyl)cyclohex-3-en-1-yl phosphate (**2h**) ^{1}H NMR (400 MHz, $CDCl_3$)

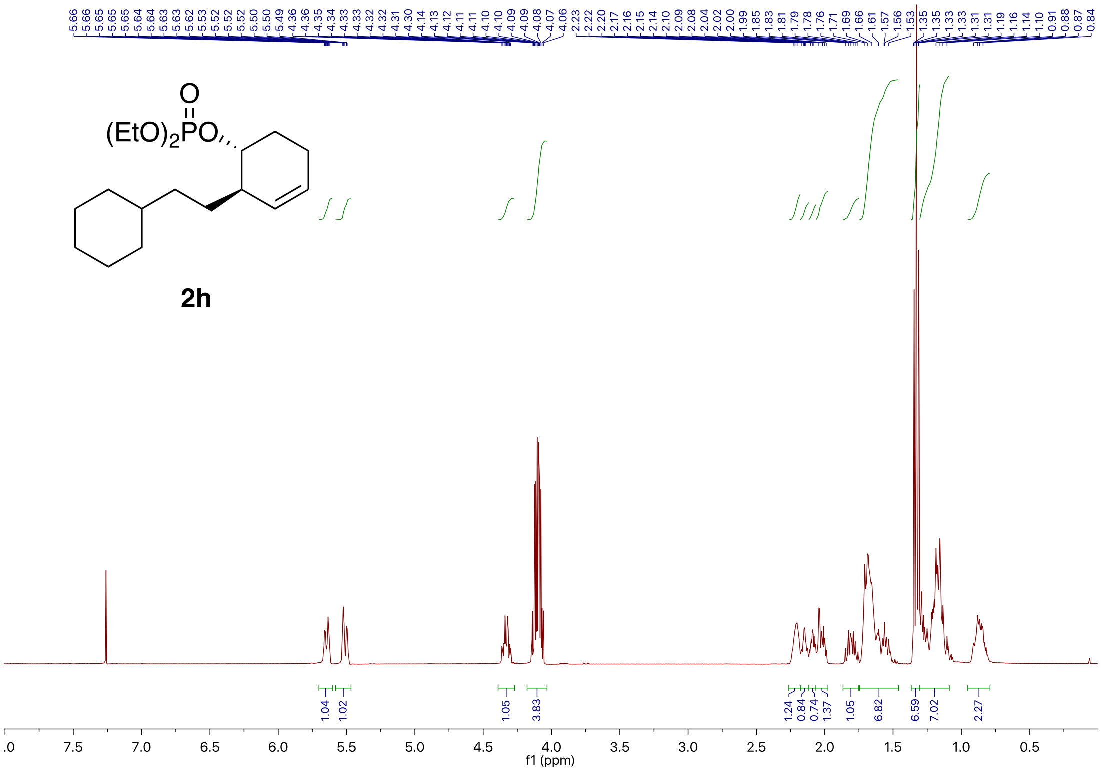

^{13}C NMR (101 MHz, $CDCl_3$)

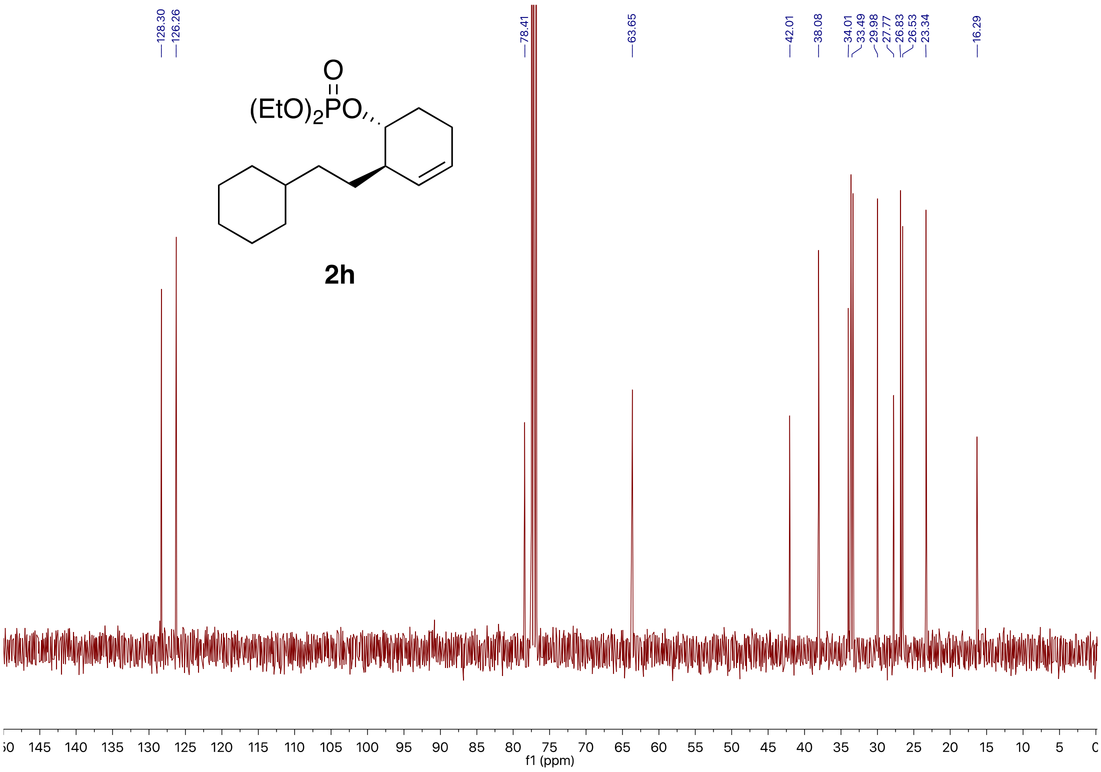

^{31}P NMR (162 MHz, CDCl$_3$)

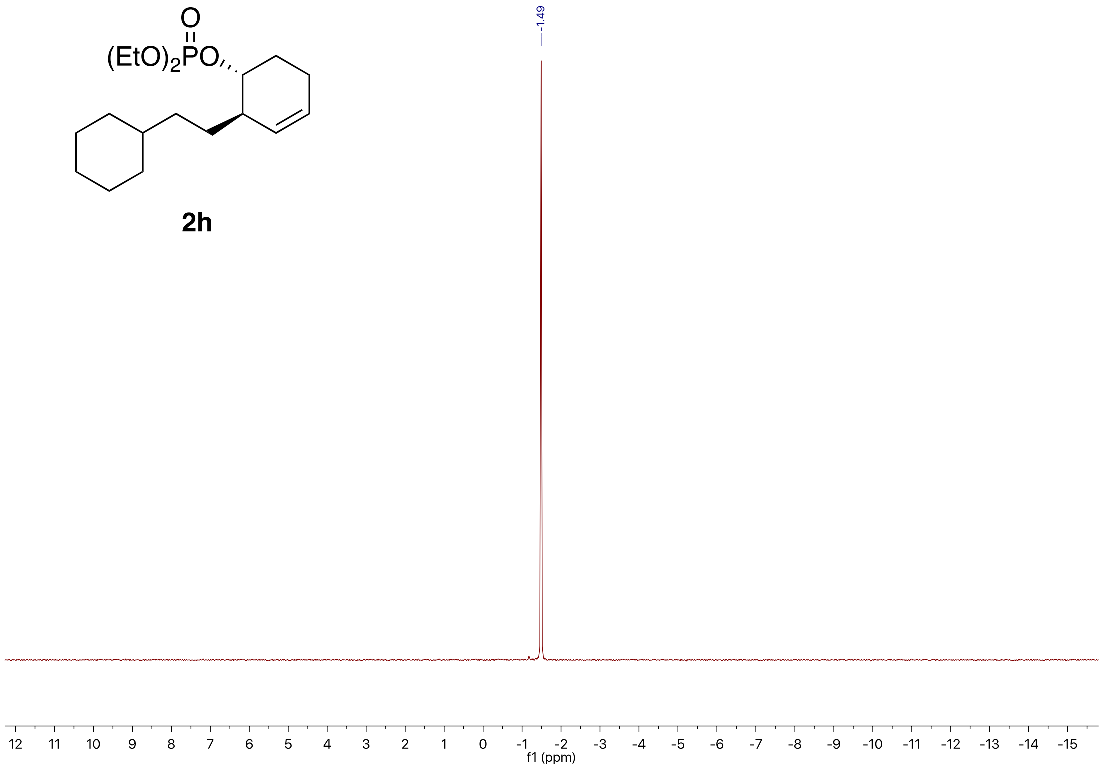

GC Trace

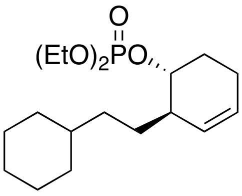

2h

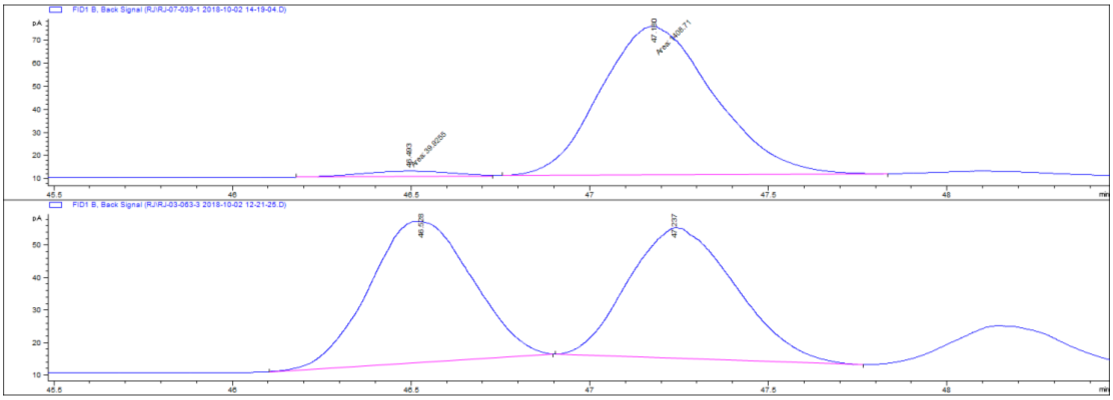

Supplementary Figure 42 - (1*R*,2*R*)-2-(6-chlorohexyl)cyclopent-3-en-1-yl diethyl phosphate (**1i**) ^{1}H NMR (400 MHz, $CDCl_3$)

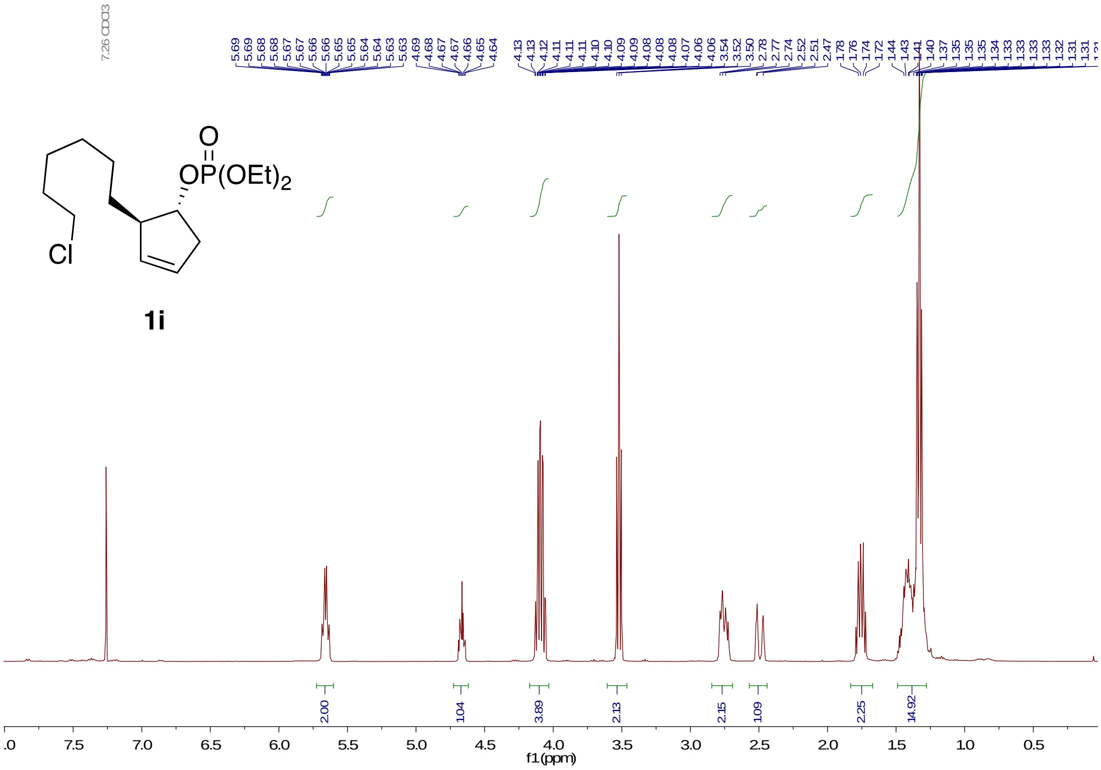

^{13}C NMR (101 MHz, $CDCl_3$)

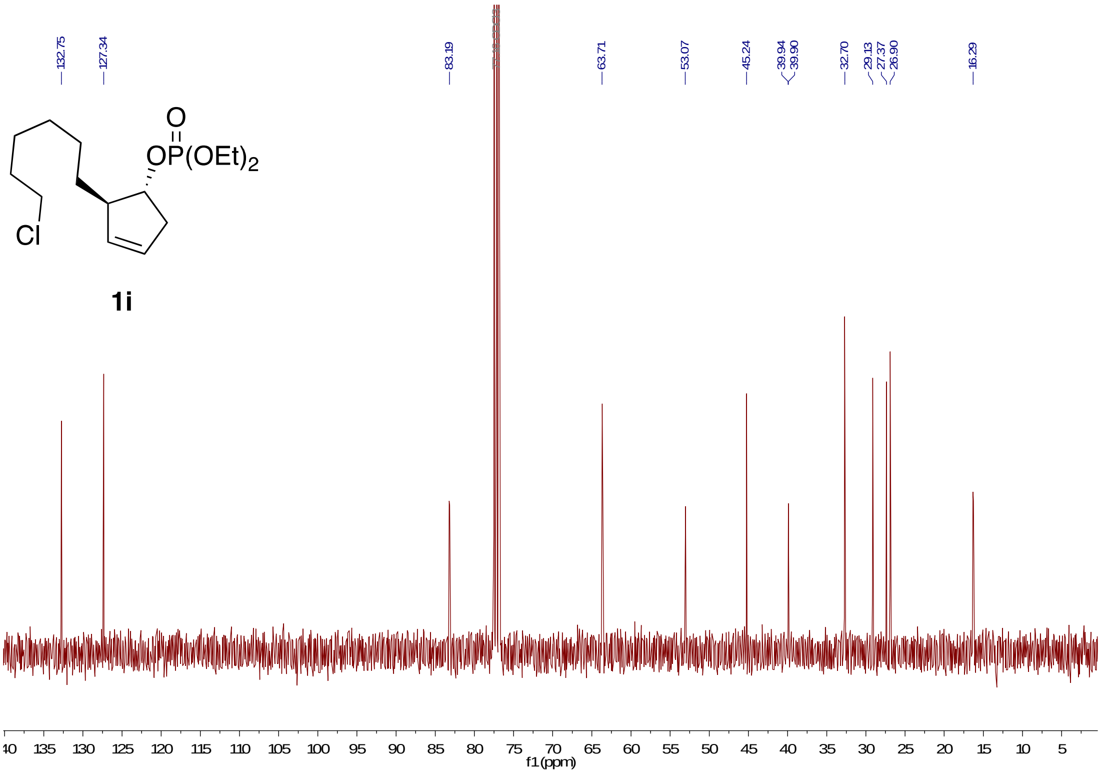

^{31}P NMR (162 MHz, $CDCl_3$)

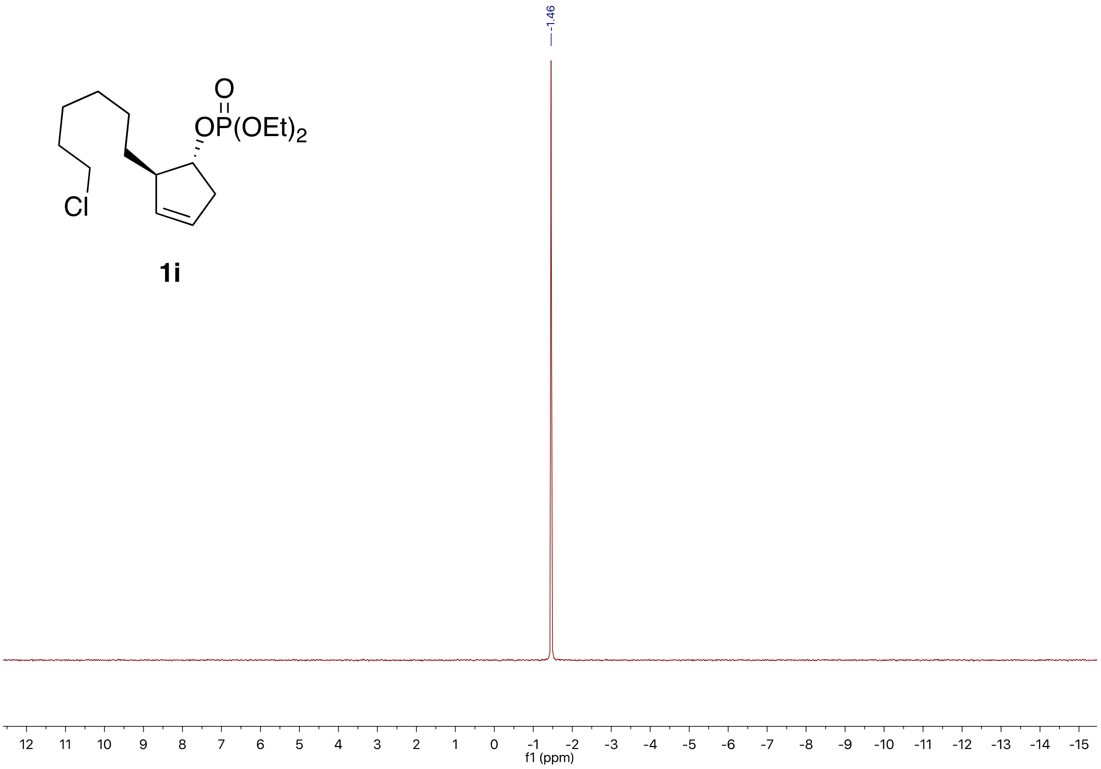

HPLC Trace

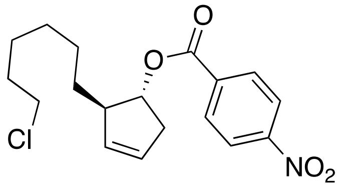

1i'

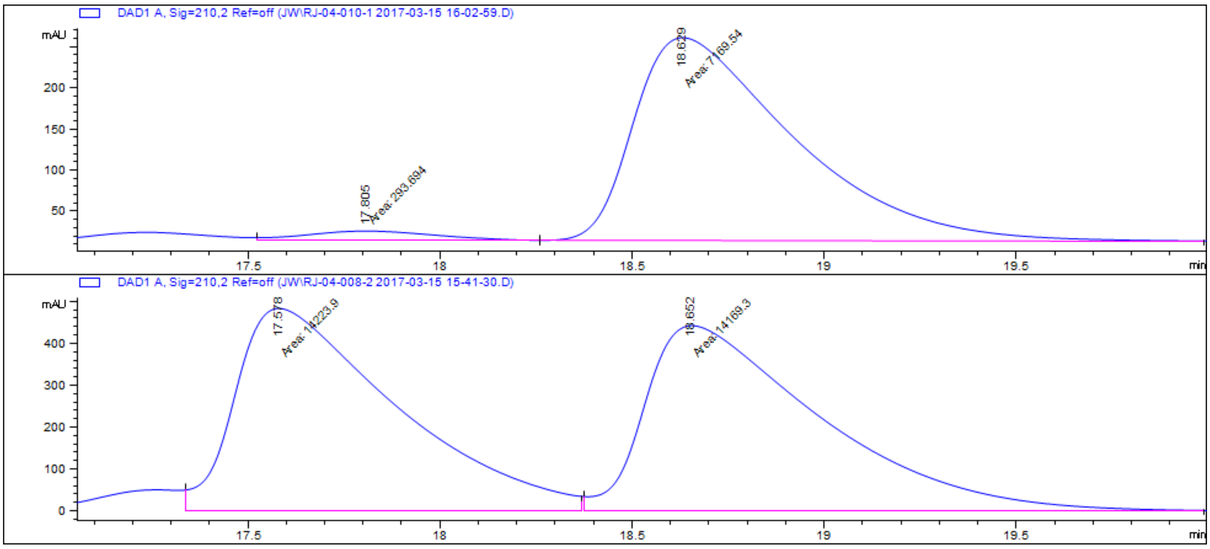

Supplementary Figure 43 - (1*R*,2*R*)-2-(6-chlorohexyl)cyclohex-3-en-1-yl diethyl phosphate (**2i**) ^{1}H NMR (400 MHz, $CDCl_3$)

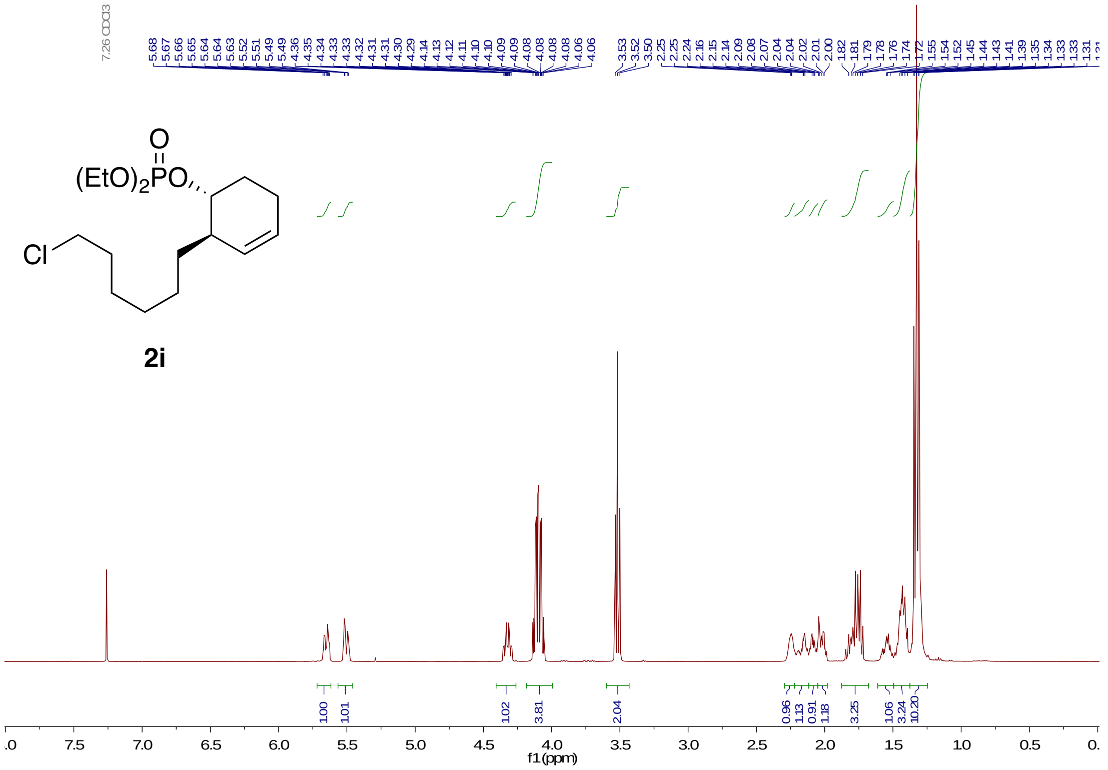

^{13}C NMR (101 MHz, $CDCl_3$)

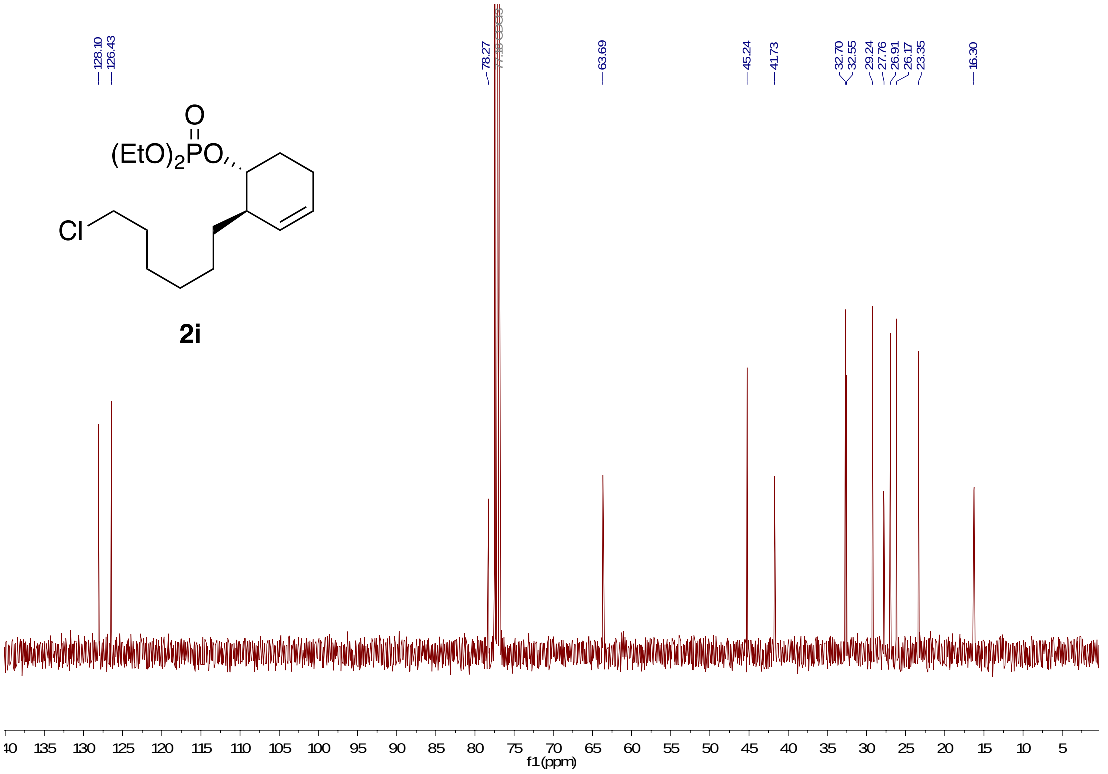

^{31}P NMR (162 MHz, CDCl$_3$)

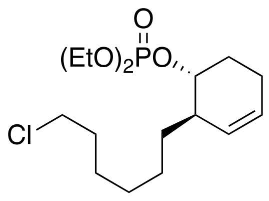

2i

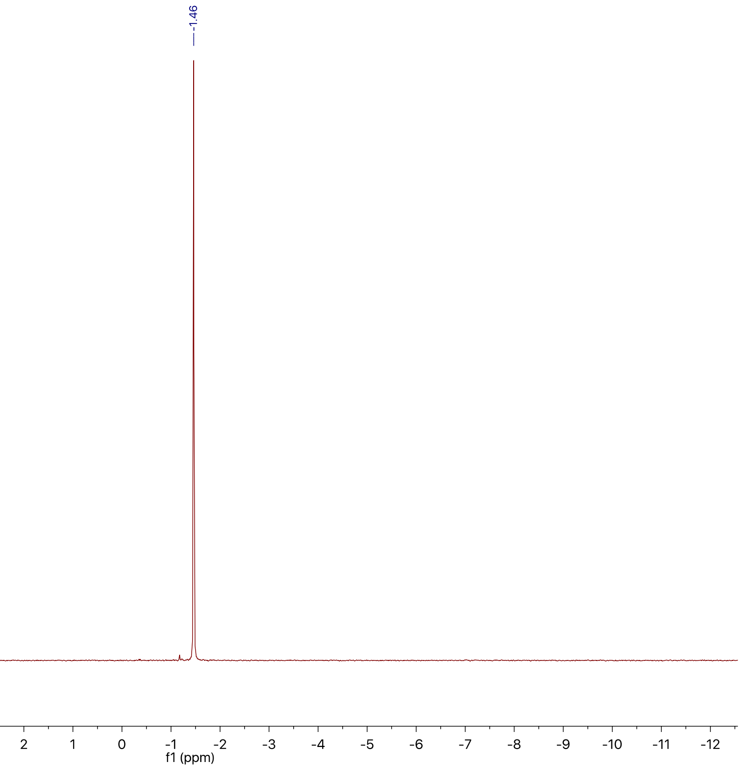

HPLC Trace

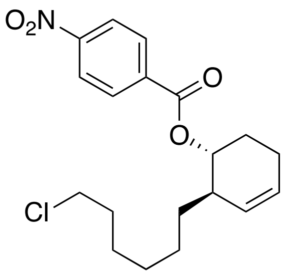

1i'

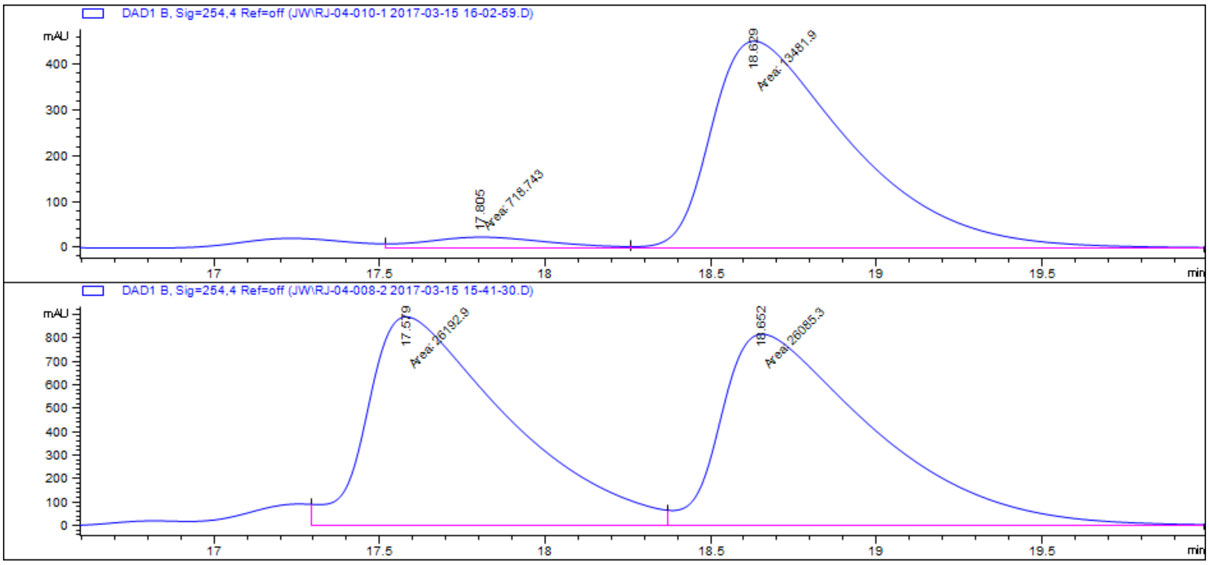

Supplementary Figure 44 - (1*R*,2*R*)-2-(4-((*tert*-butyldiphenylsilyl)oxy)butyl)cyclopent-3-en-1-yl diethyl phosphate (**1j**) ^{1}H NMR (400 MHz, $CDCl_3$)

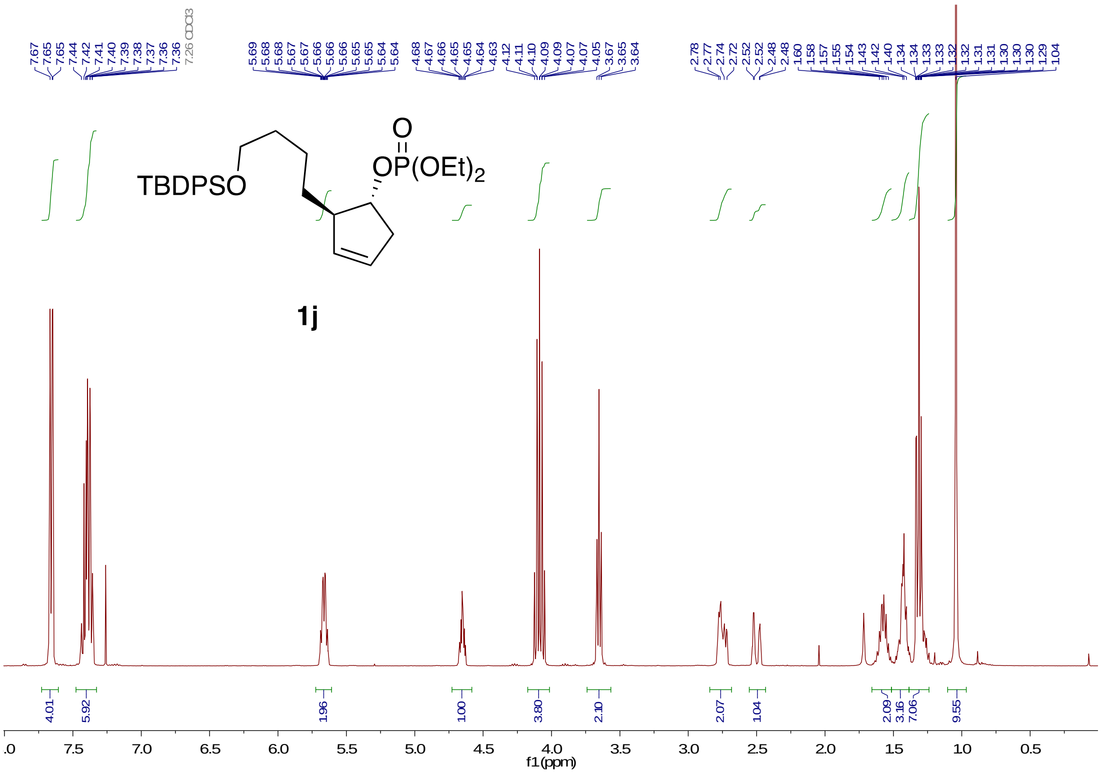

^{13}C NMR (101 MHz, $CDCl_3$)

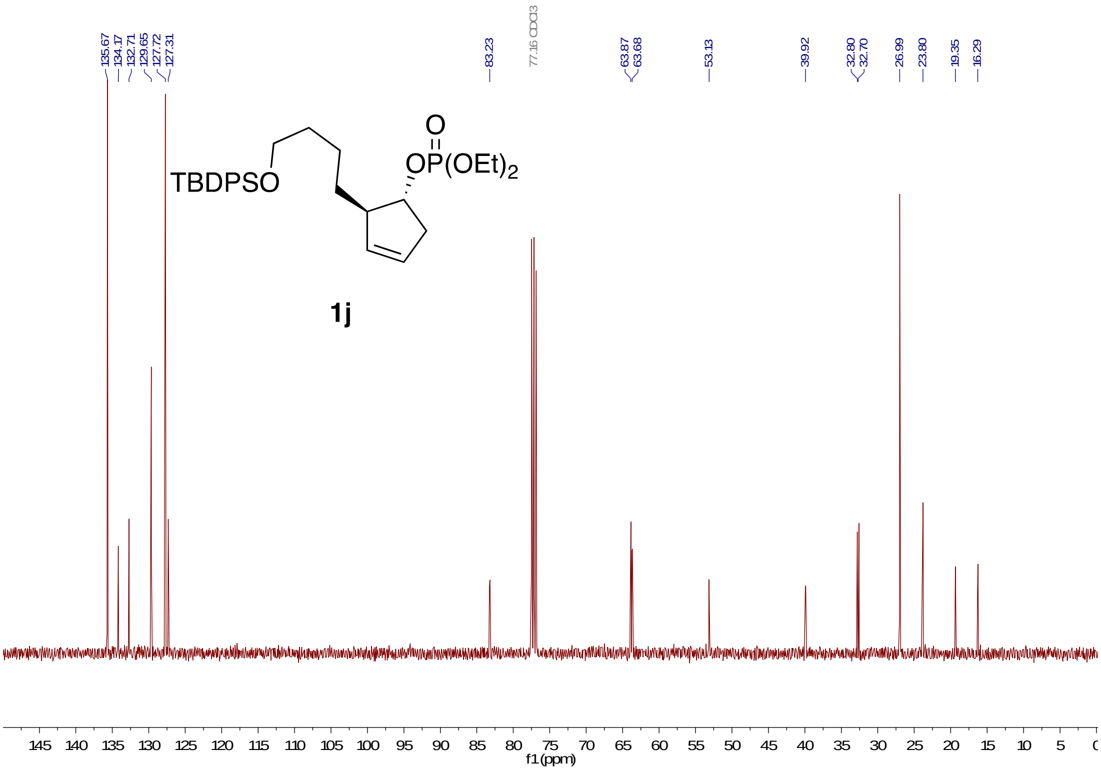

^{31}P NMR (162 MHz, $CDCl_3$)

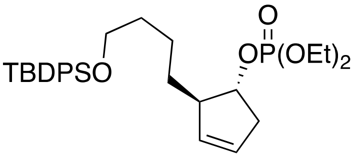

1j

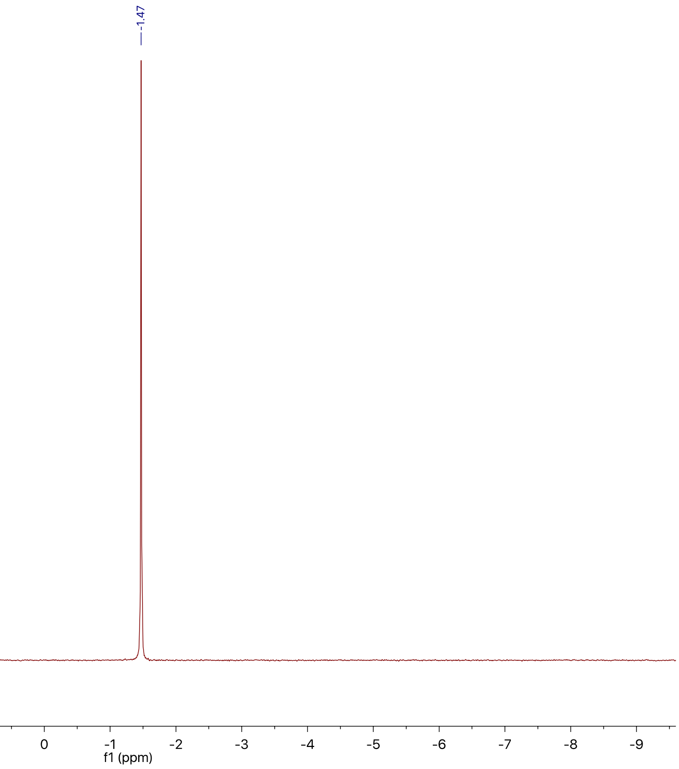

HPLC Trace

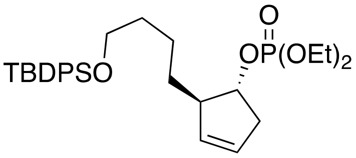

1j

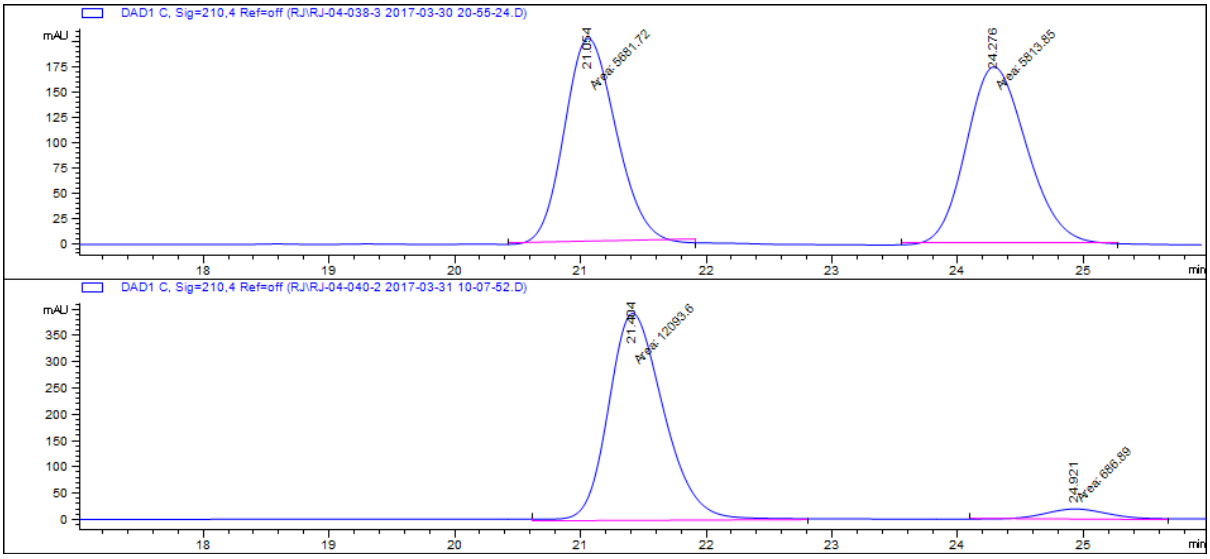

Supplementary Figure 45 - (1*R*,2*R*)-2-(4-((*tert*-butyldiphenylsilyl)oxy)butyl)cyclohex-3-en-1-yl diethyl phosphate (**2j**) ^{1}H NMR (400 MHz, $CDCl_3$)

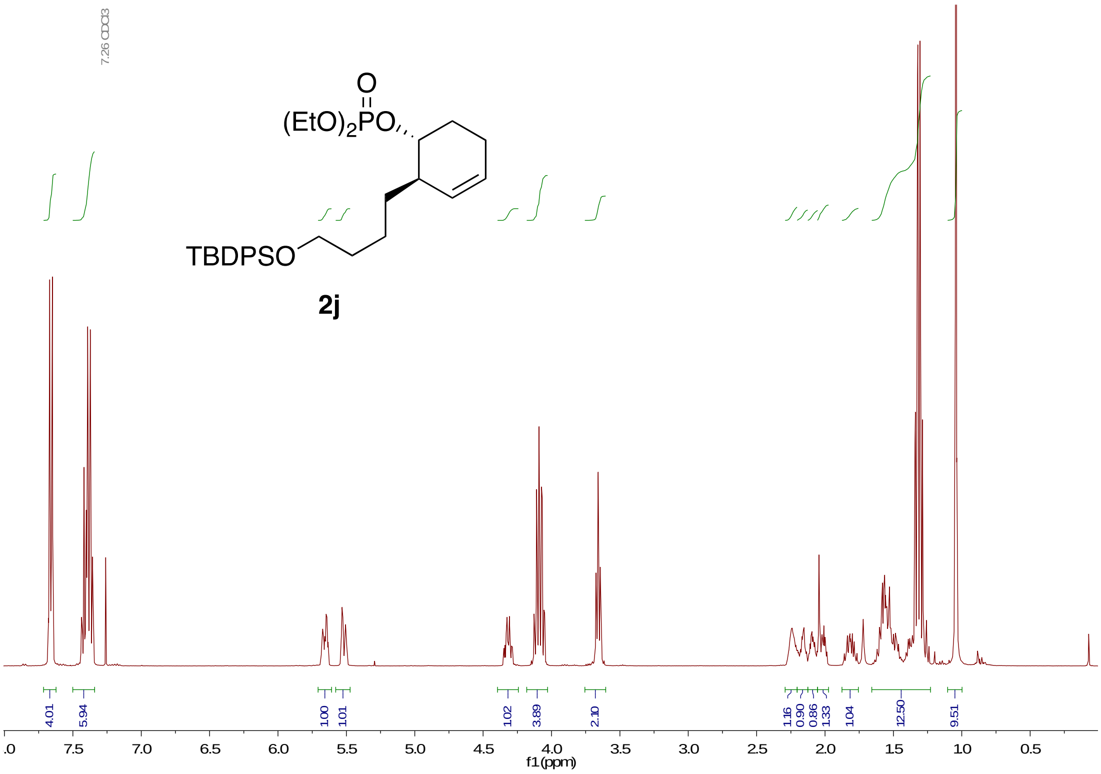

^{13}C NMR (101 MHz, $CDCl_3$)

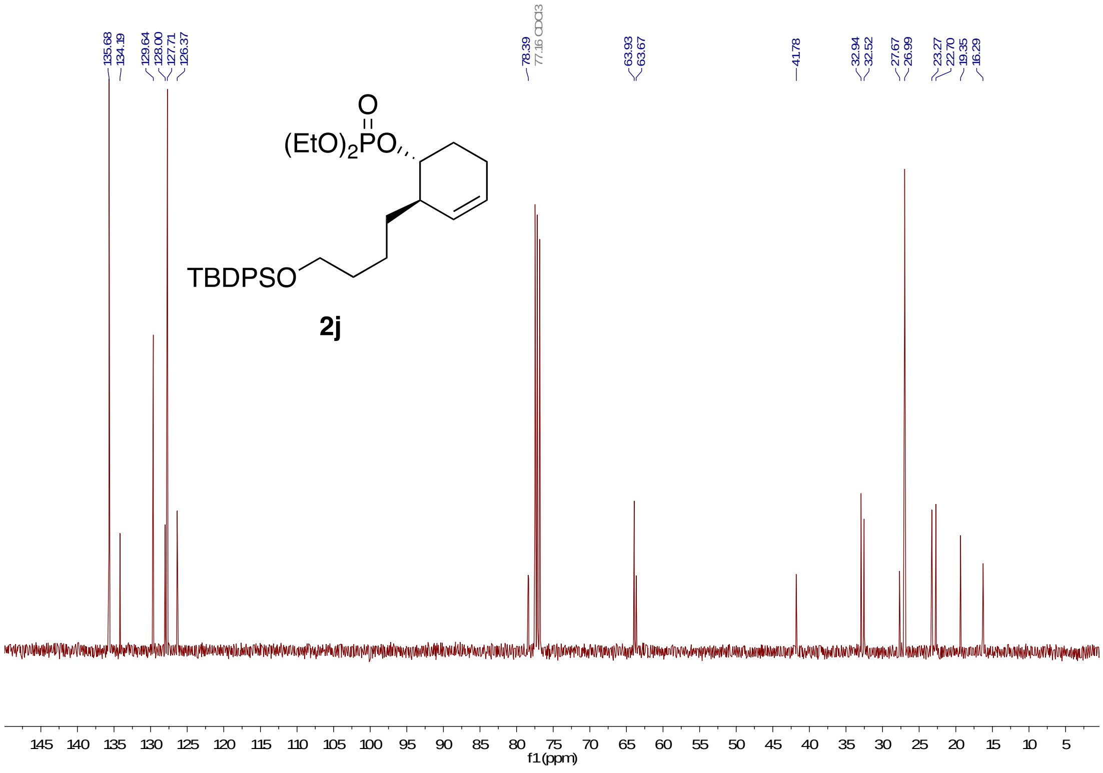

^{31}P NMR (162 MHz, $CDCl_3$)

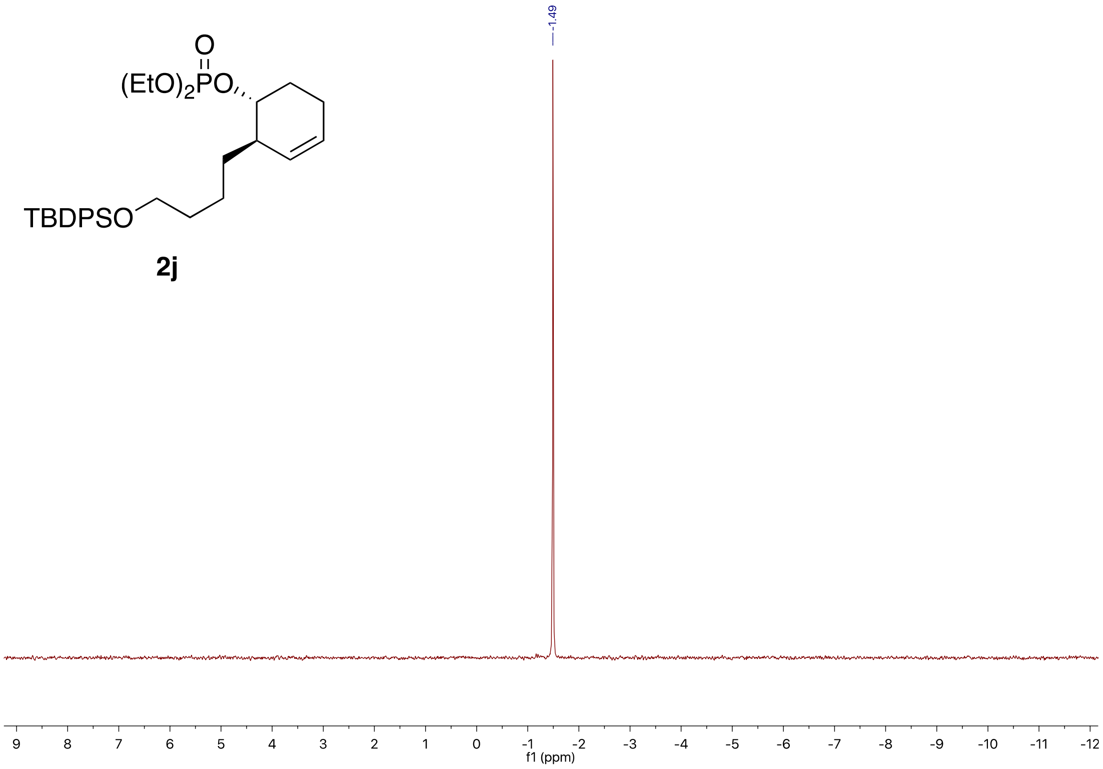

HPLC Trace

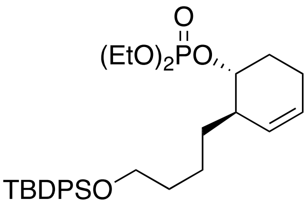

2j

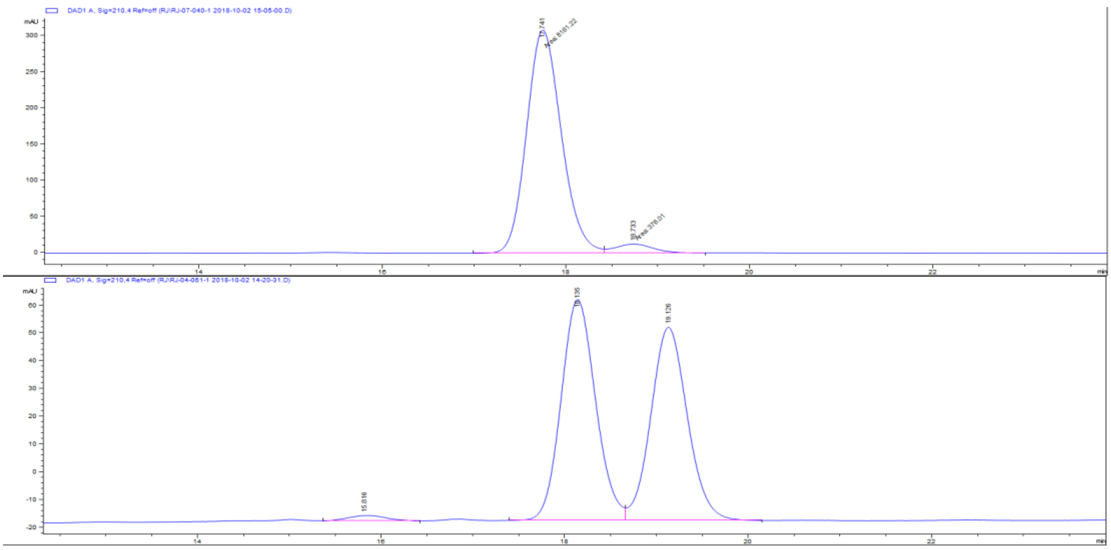

Supplementary Figure 46 - (1*R*,2*R*)-2-(4-((*tert*-butyldiphenylsilyl)oxy)butyl)cyclohept-3-en-1-yl diethyl phosphate (**3j**) ^{1}H NMR (400 MHz, $CDCl_3$)

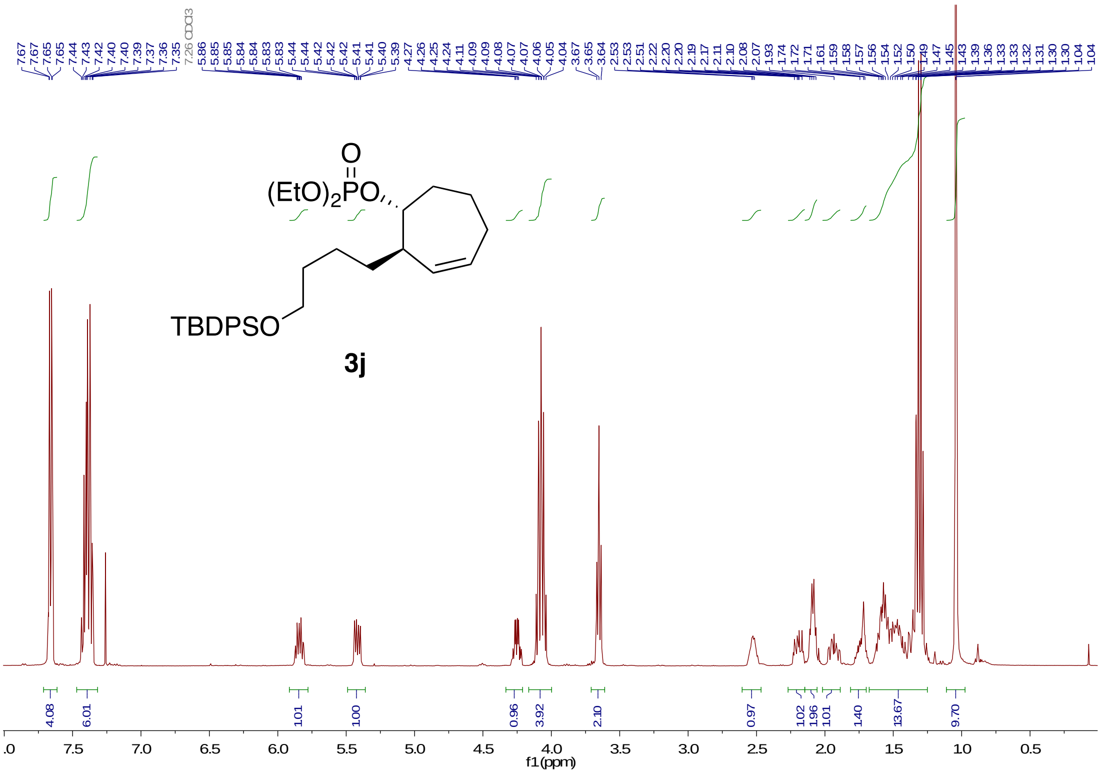

^{13}C NMR (101 MHz, $CDCl_3$)

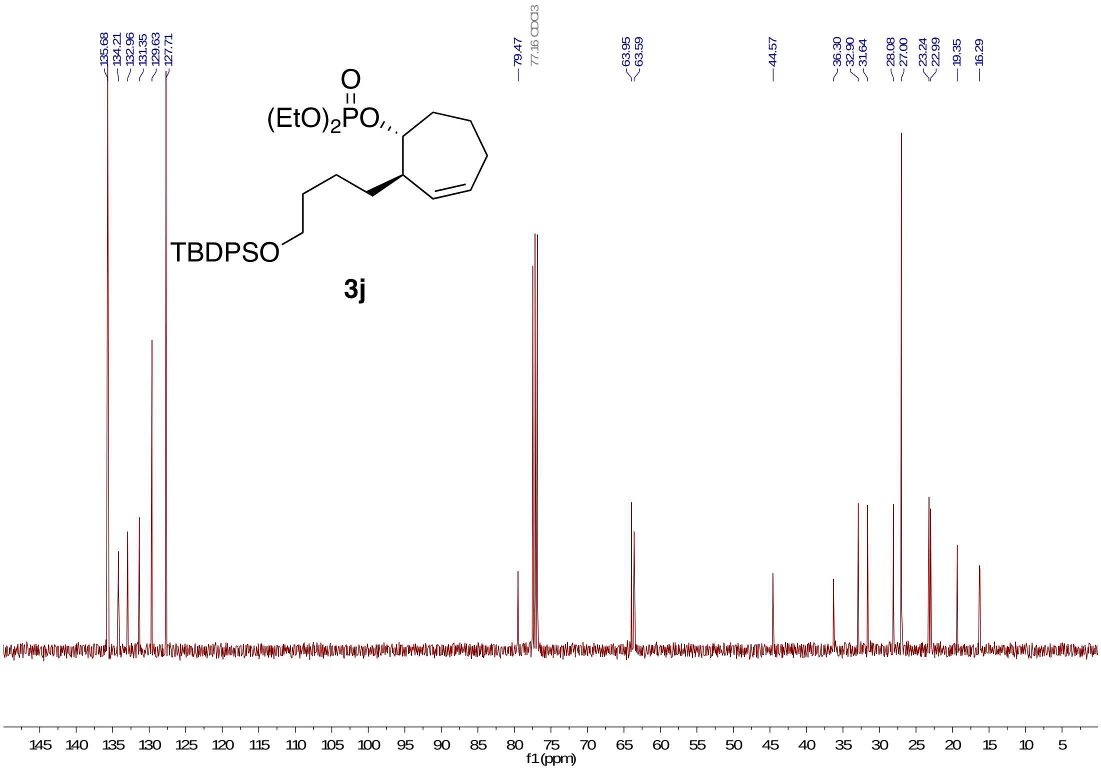

^{31}P NMR (162 MHz, $CDCl_3$)

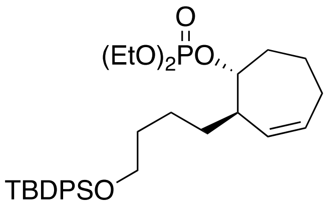

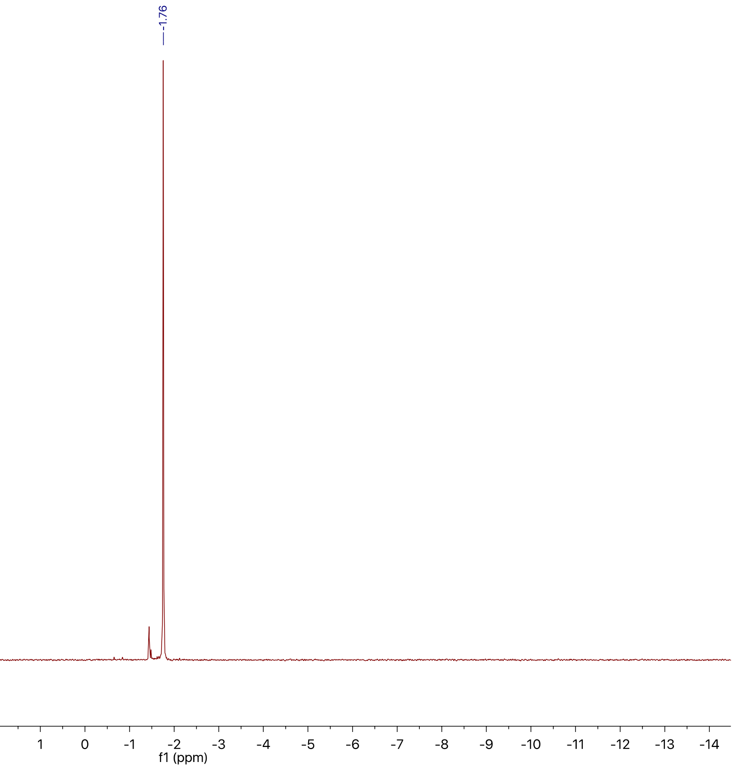

HPLC Trace

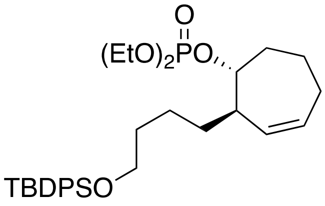

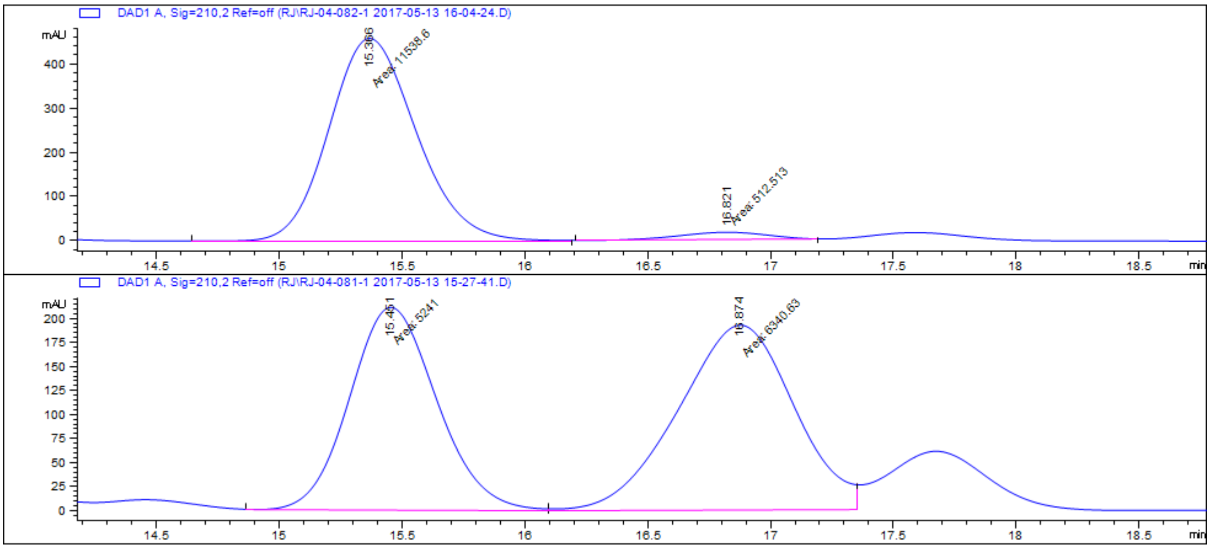

Supplementary Figure 47 - Diethyl ((1*R*,2*R*)-2-(4-methoxyphenethyl)cyclopent-3-en-1-yl) phosphate (**1k**)

^{1}H NMR (400 MHz, CDCl$_3$)

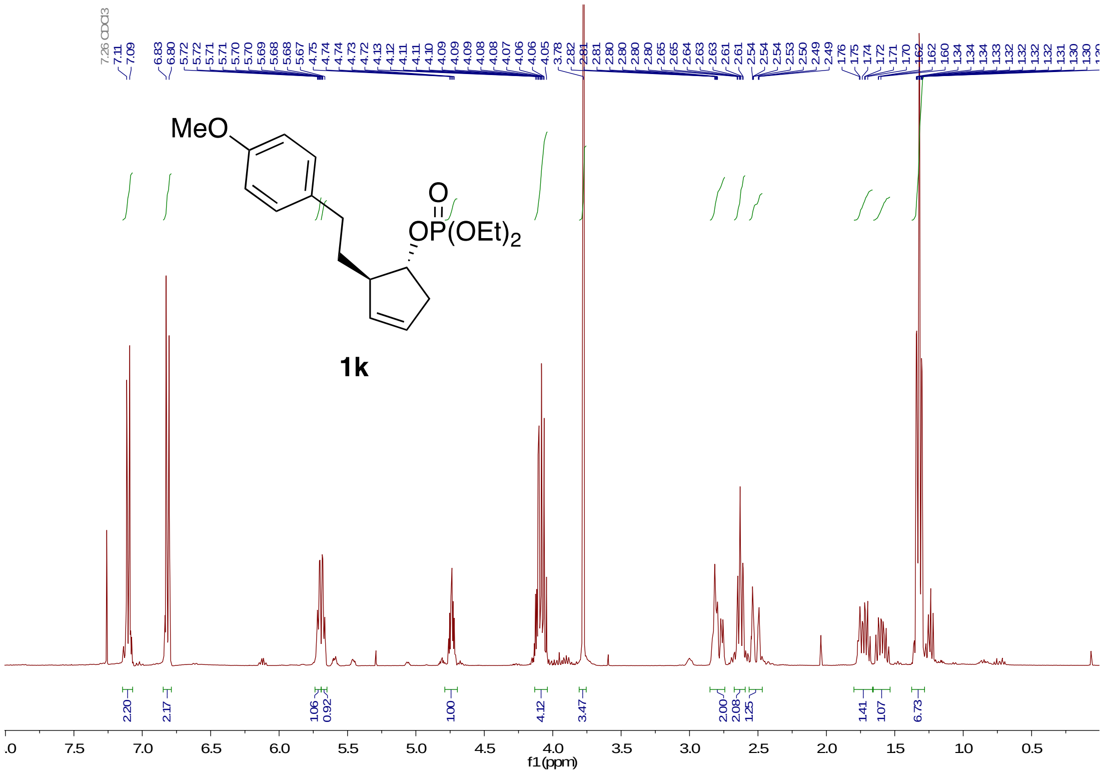

^{13}C NMR (101 MHz, CDCl$_3$)

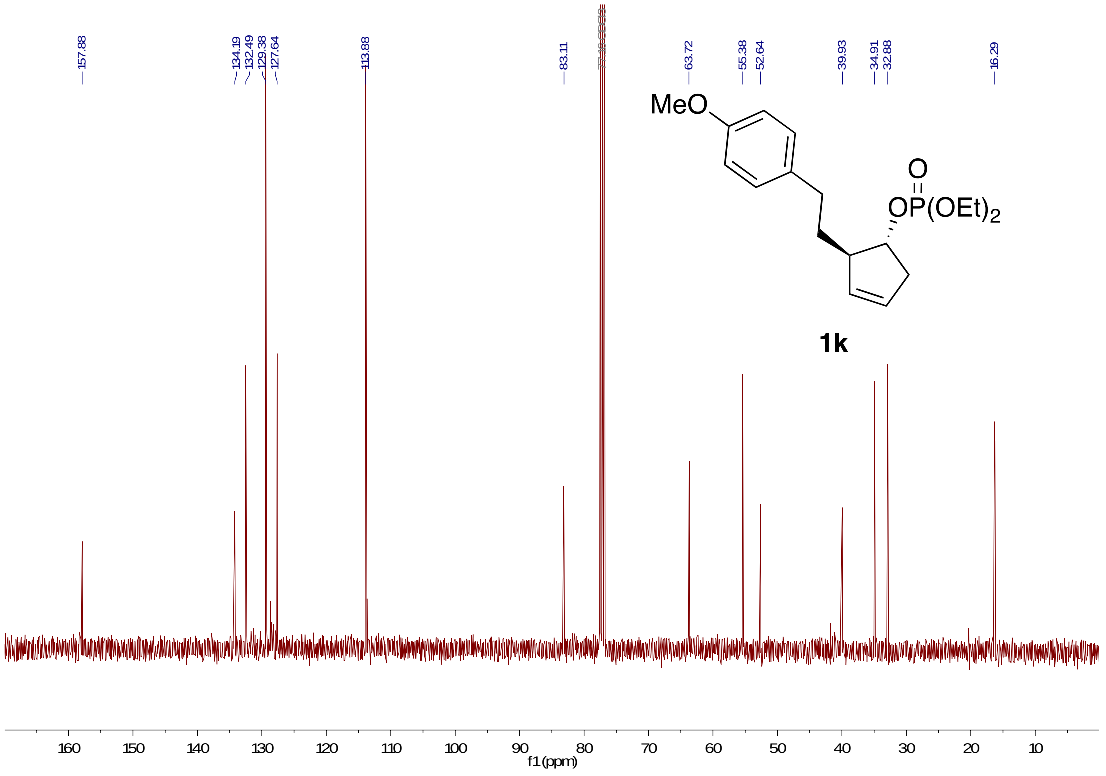

^{31}P NMR (162 MHz, $CDCl_3$)

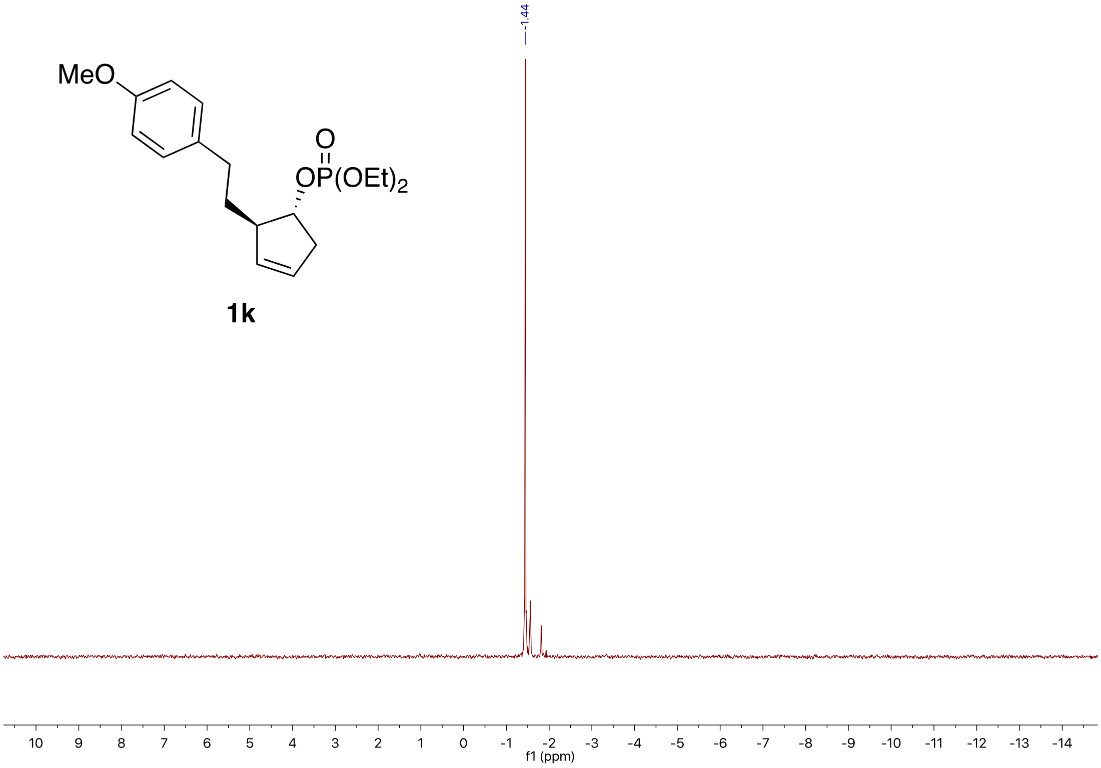

HPLC Trace

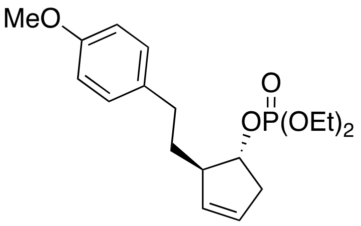

1k

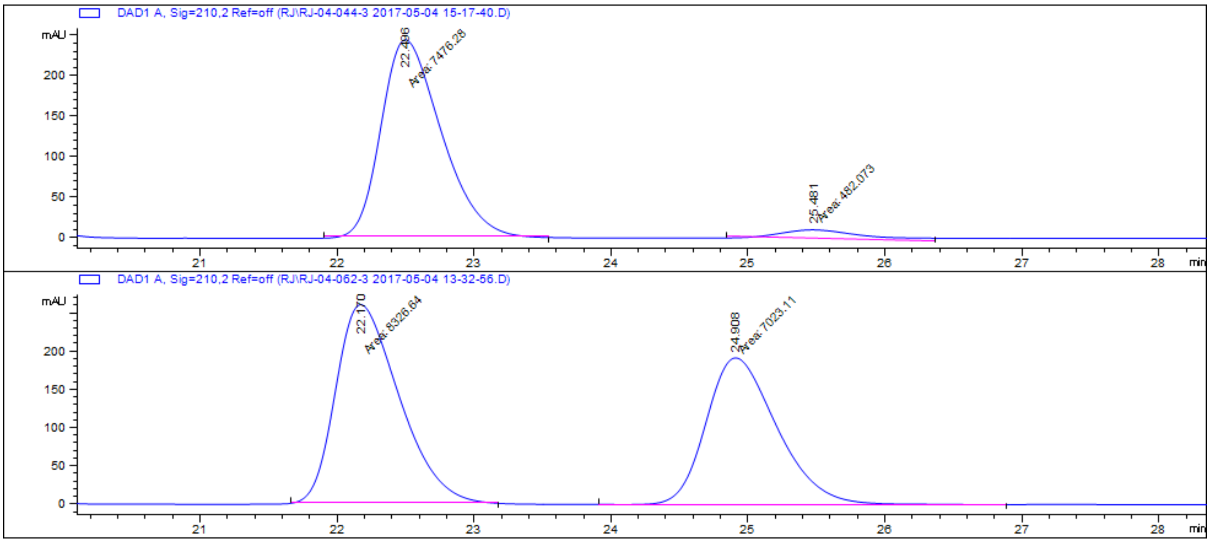

Supplementary Figure 48 - Diethyl ((1*R*,2*R*)-2-(4-methoxyphenethyl)cyclohex-3-en-1-yl) phosphate (**2k**) ^{1}H NMR (400 MHz, $CDCl_3$)

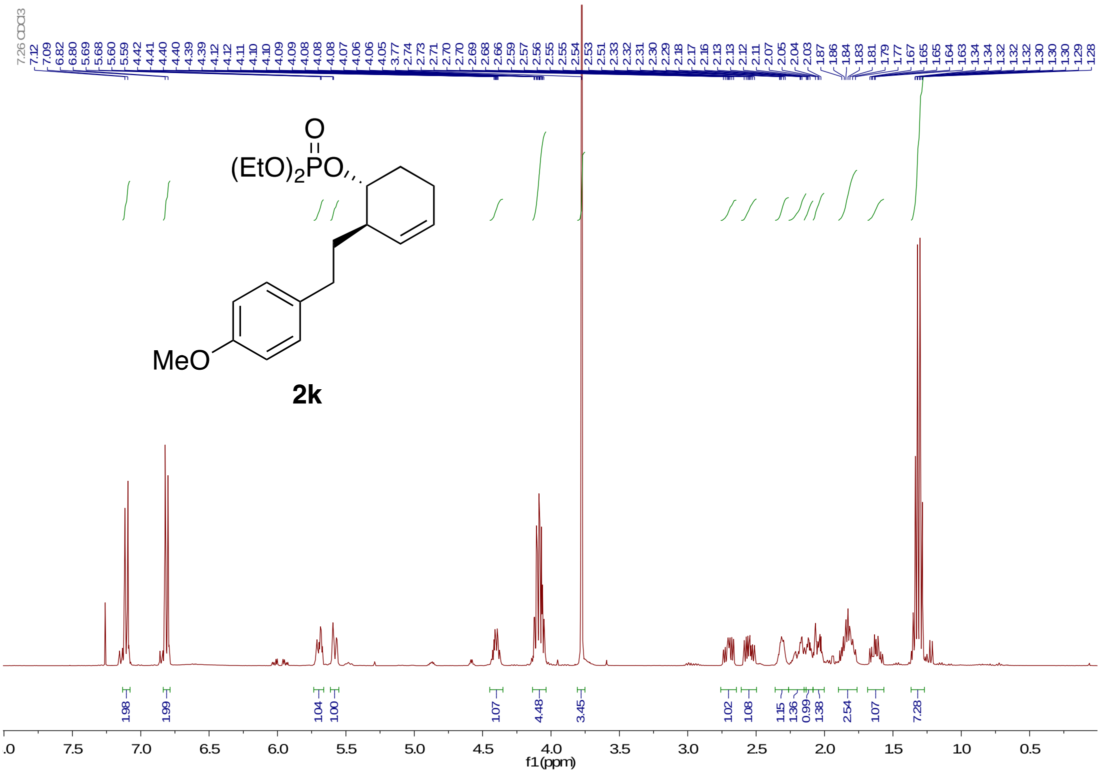

^{13}C NMR (101 MHz, $CDCl_3$)

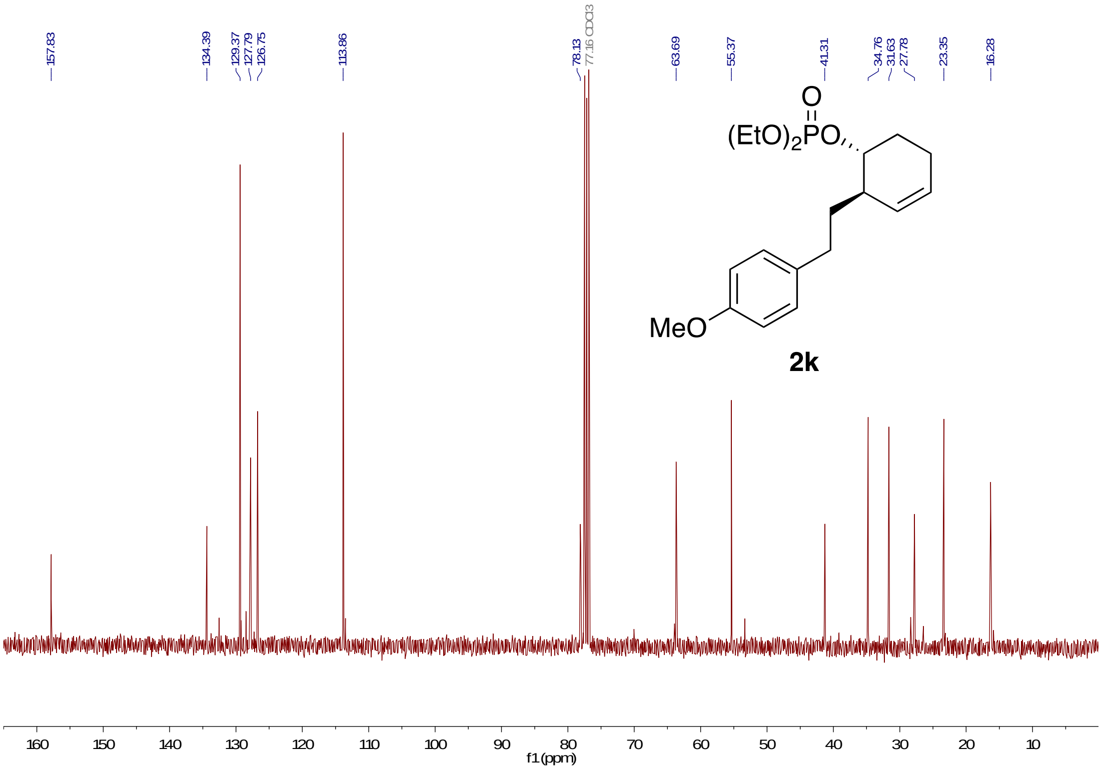

^{31}P NMR (162 MHz, $CDCl_3$)

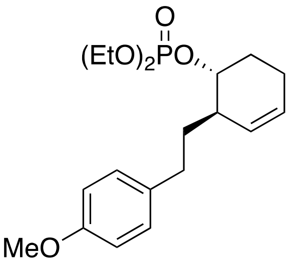

2k

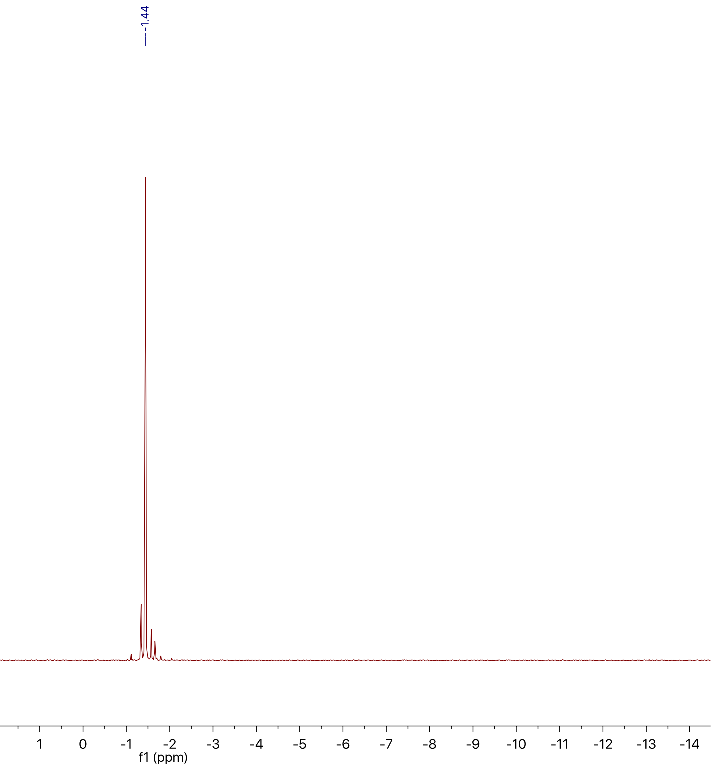

HPLC Trace

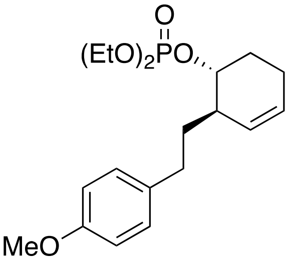

2k

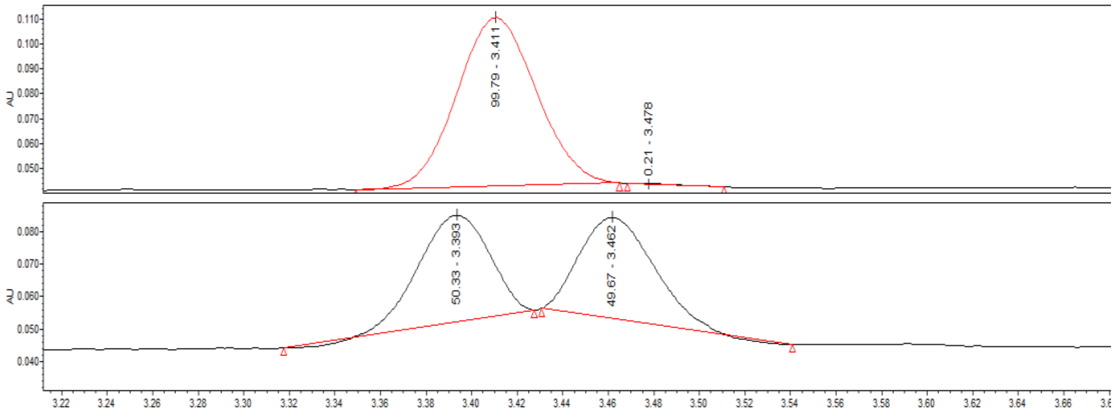

Supplementary Figure 49 - Diethyl ((1*R*,2*R*)-2-(4-methoxyphenethyl)cyclohept-3-en-1-yl) phosphate (**3k**) ^{1}H NMR (400 MHz, $CDCl_3$)

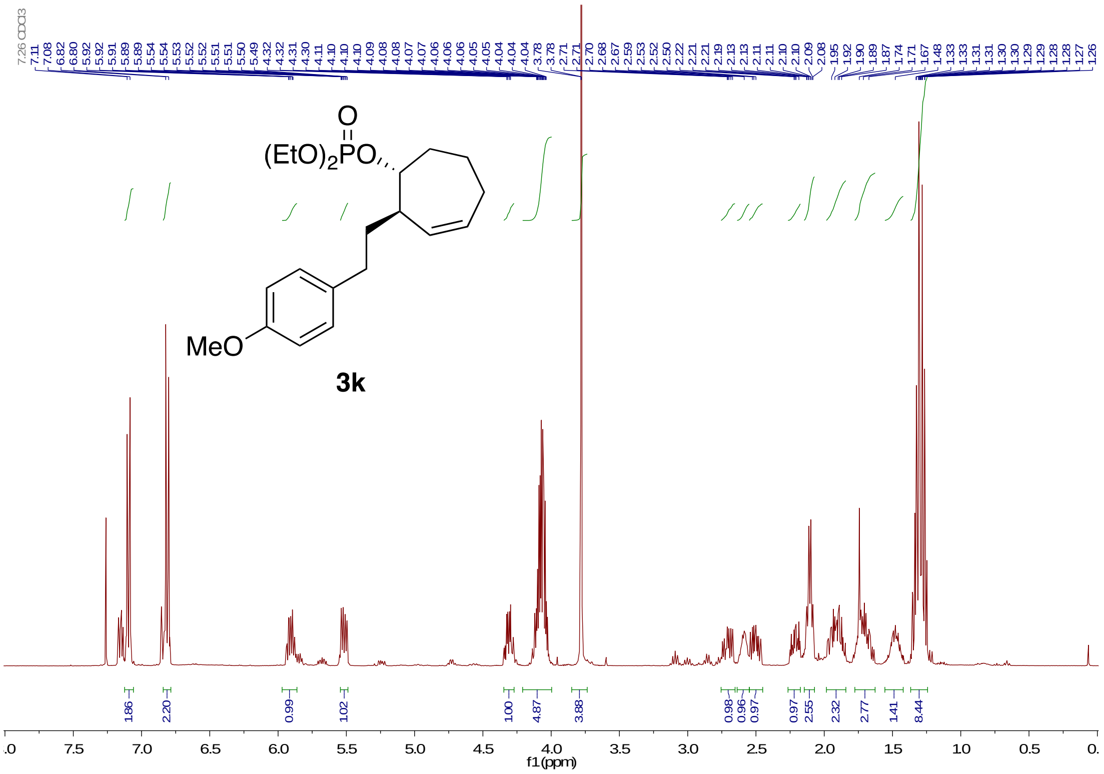

^{13}C NMR (101 MHz, $CDCl_3$)

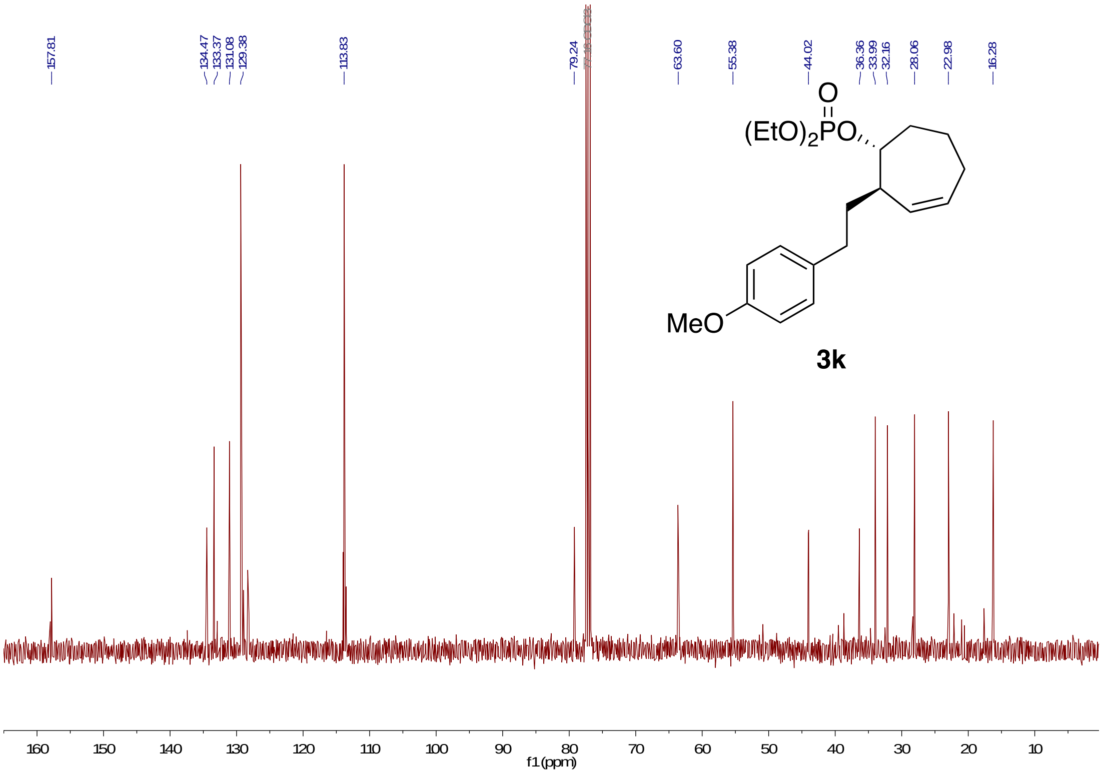

^{31}P NMR (162 MHz, CDCl$_3$)

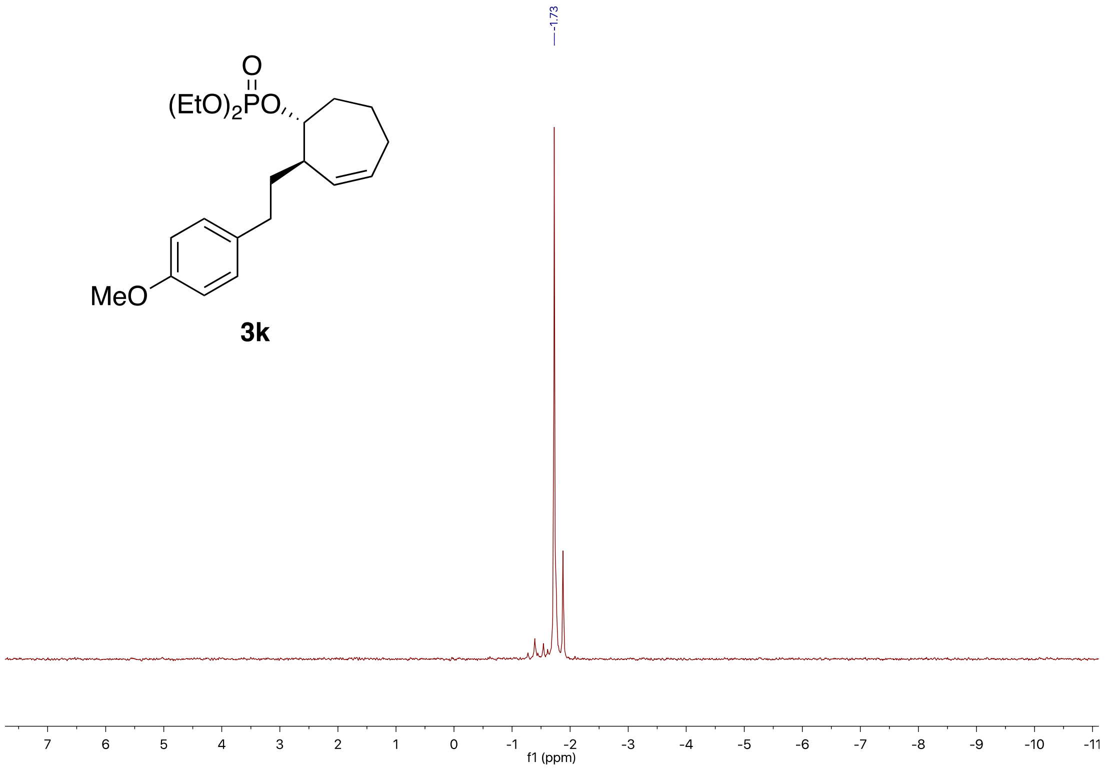

HPLC Trace

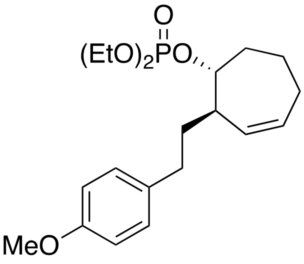

3k

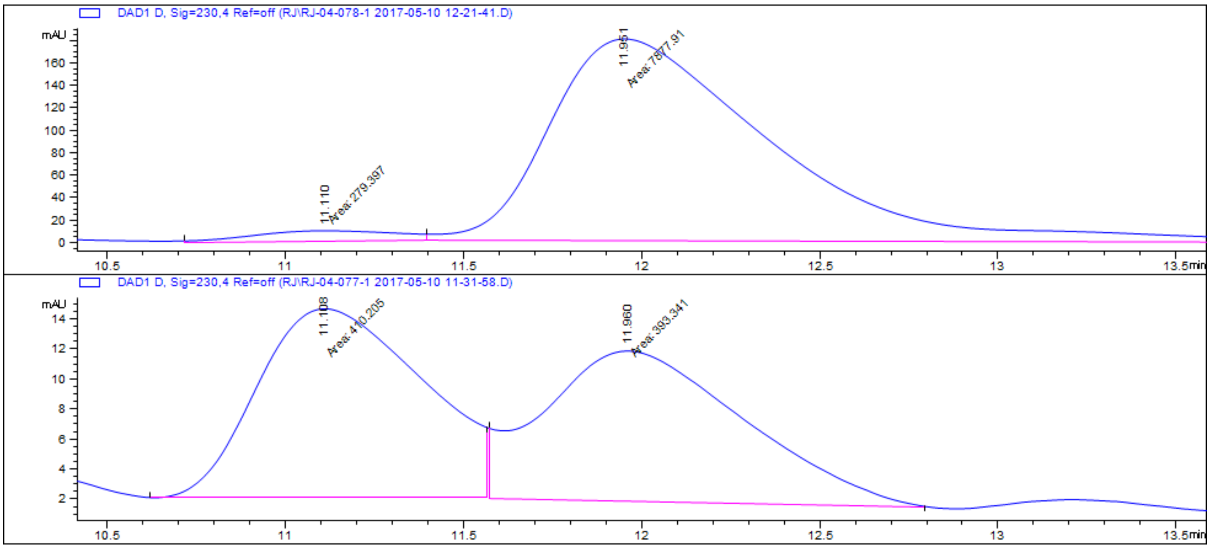

Supplementary Figure 50 - (1*R*,2*R*)-2-(2-bromophenethyl)cyclopent-3-en-1-yl diethyl phosphate (**1l**) ^{1}H NMR (400 MHz, $CDCl_3$)

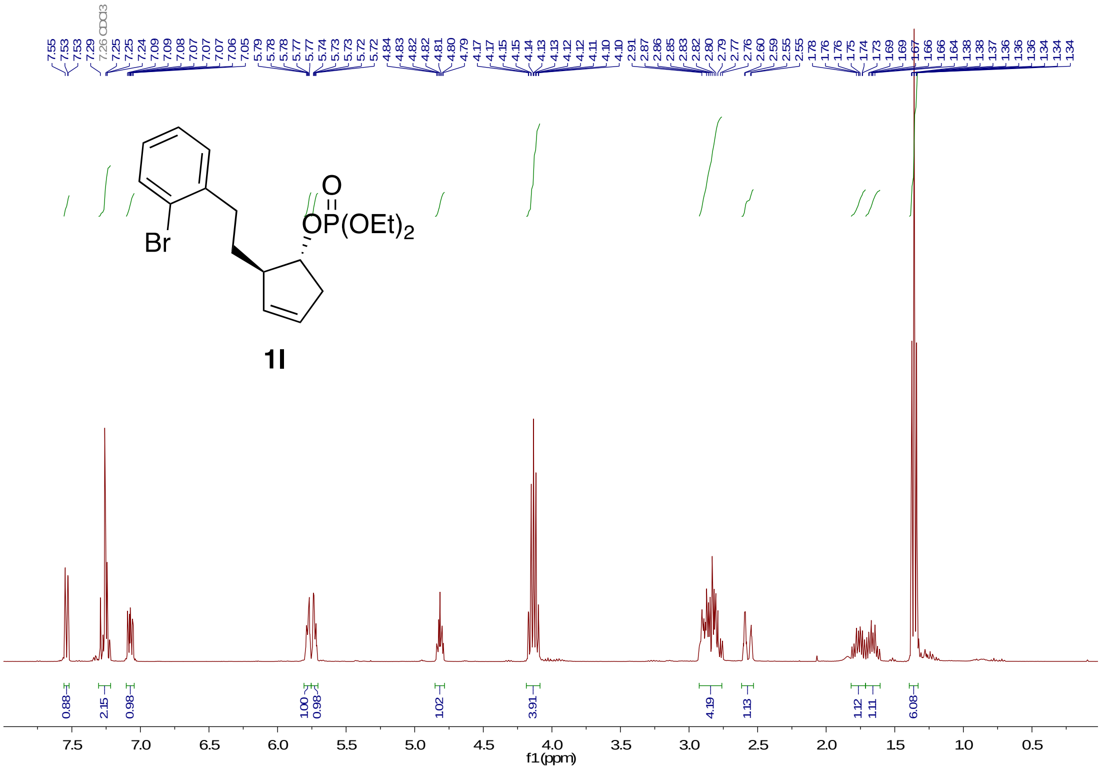

^{13}C NMR (101 MHz, $CDCl_3$)

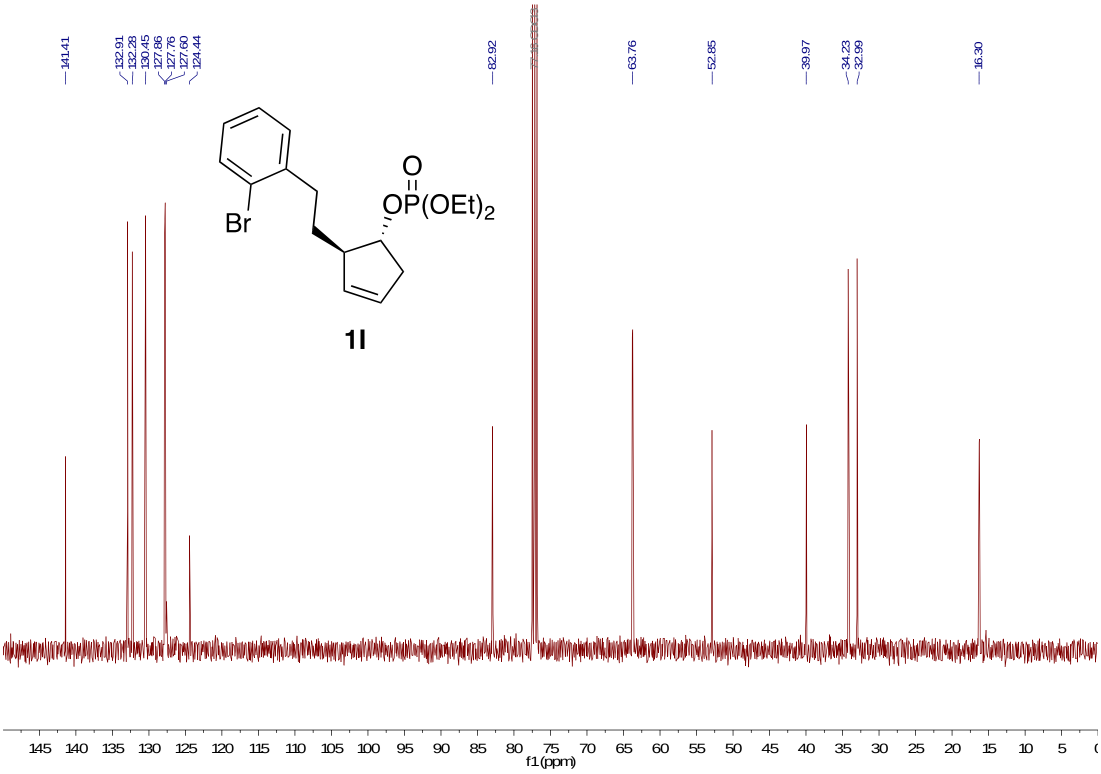

^{31}P NMR (162 MHz, $CDCl_3$)

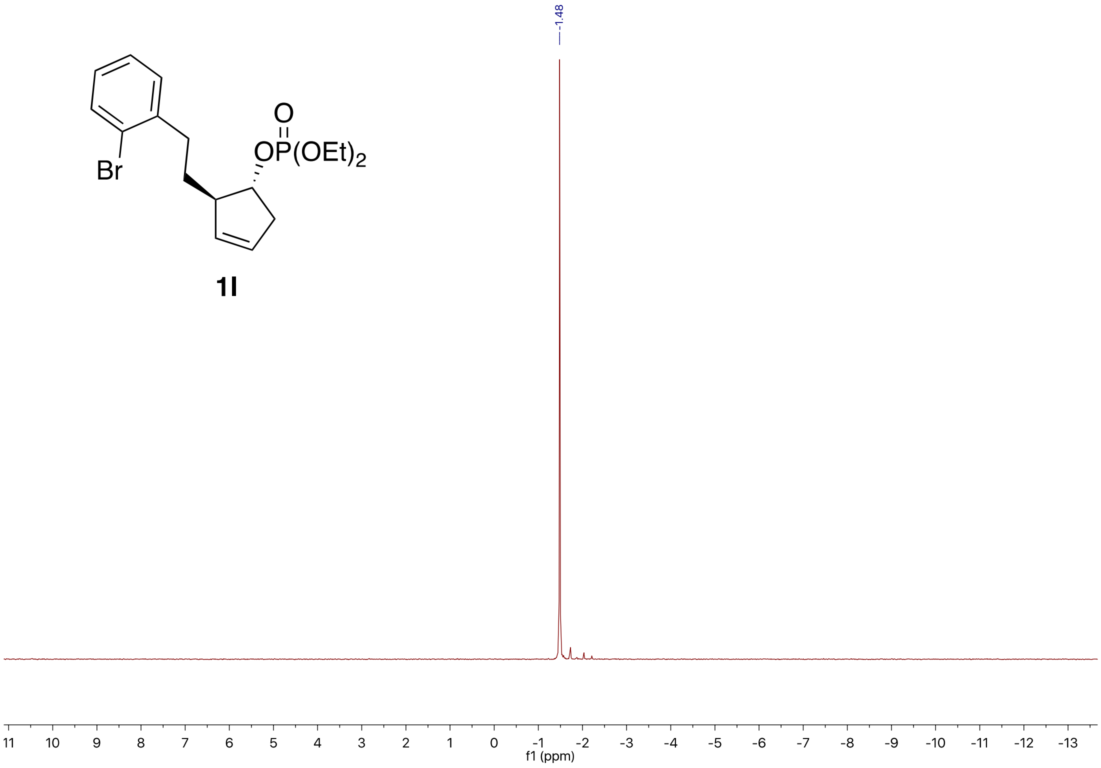

HPLC Trace

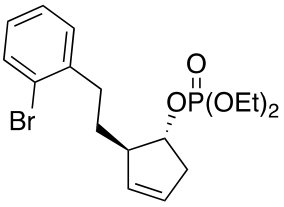

1l

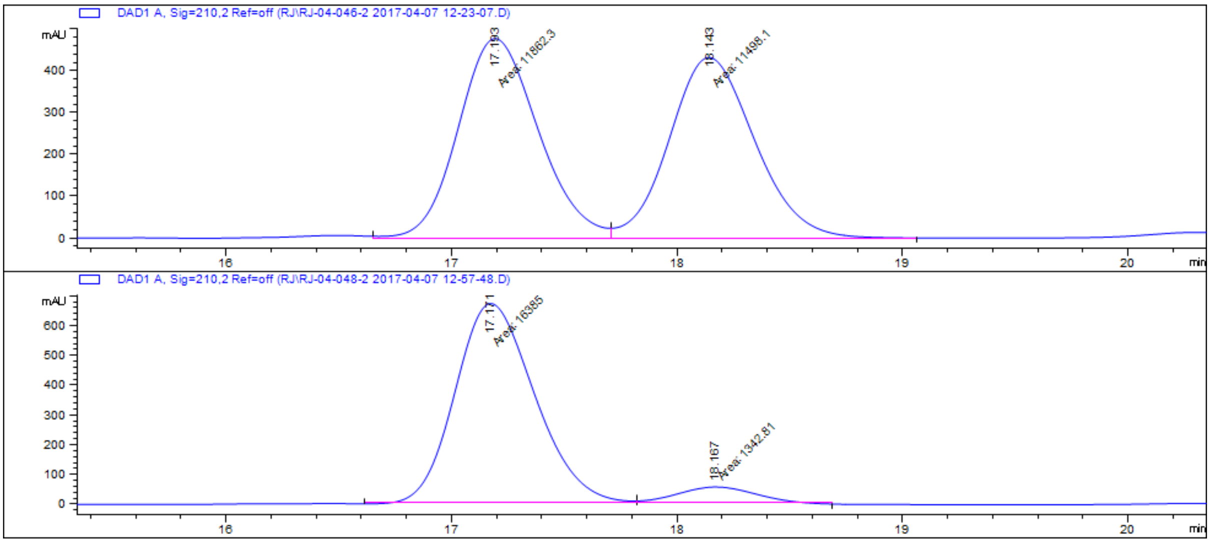

Supplementary Figure 51 - (1*R*,2*R*)-2-(2-bromophenethyl)cyclohex-3-en-1-yl diethyl phosphate (**2l**) ^{1}H NMR (400 MHz, $CDCl_3$)

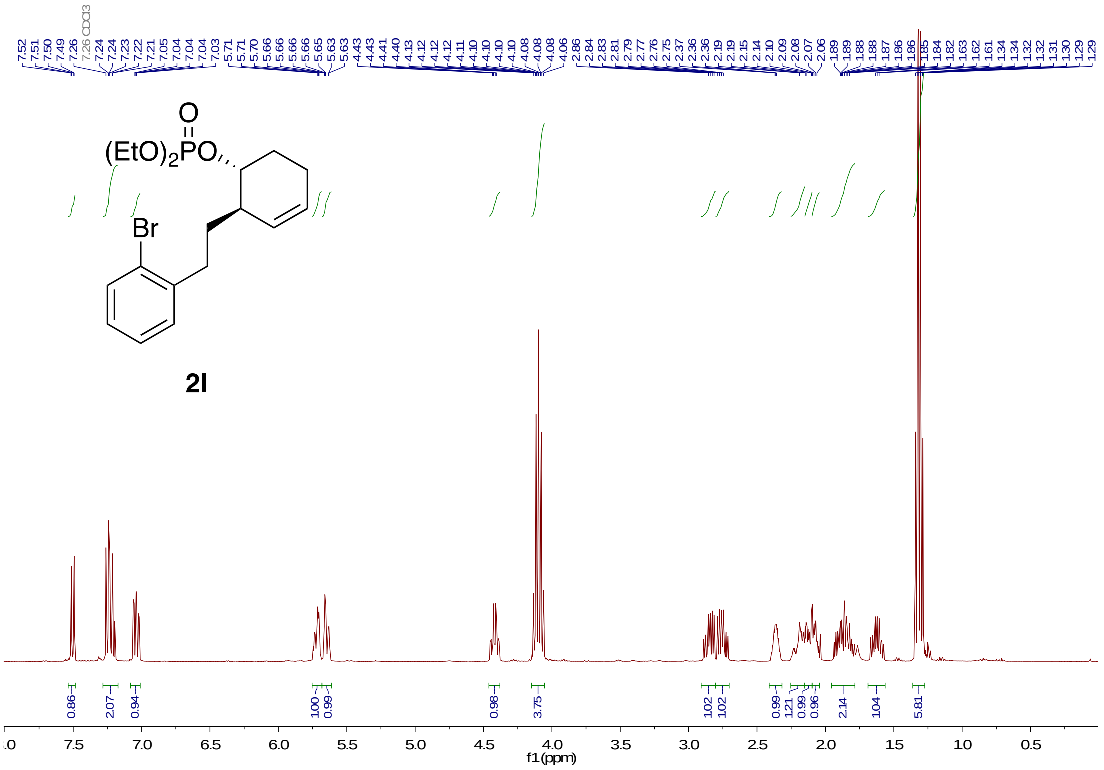

^{13}C NMR (101 MHz, $CDCl_3$)

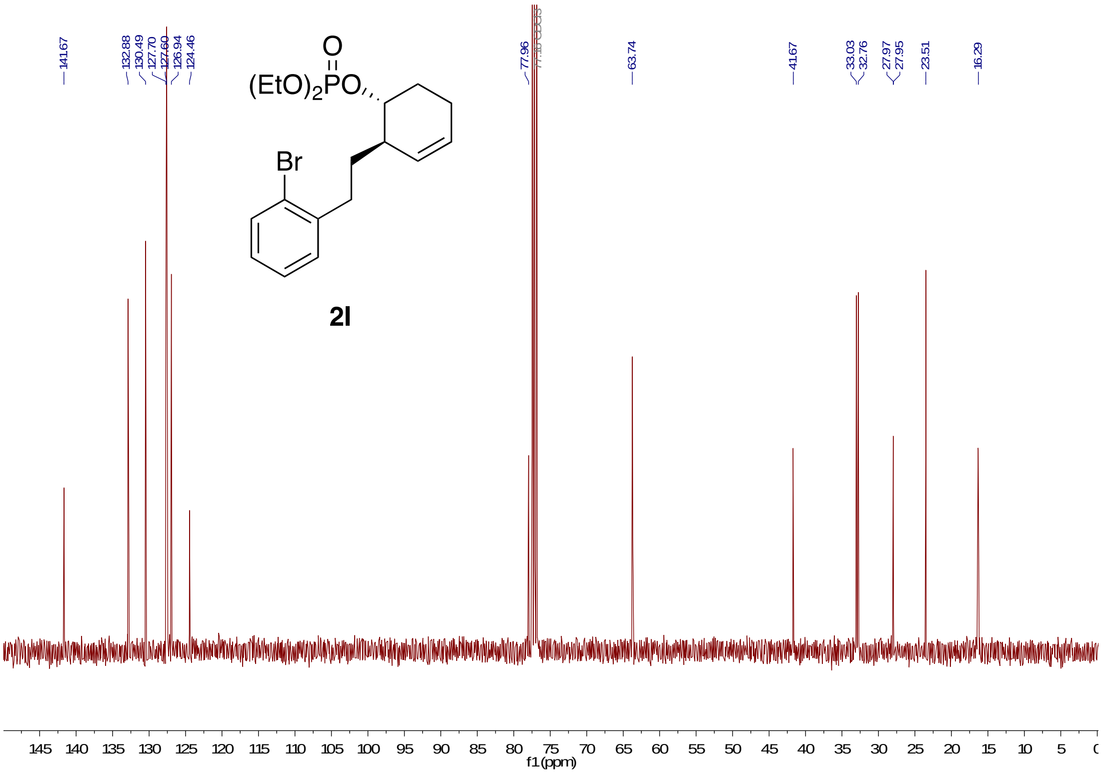

^{31}P NMR (162 MHz, $CDCl_3$)

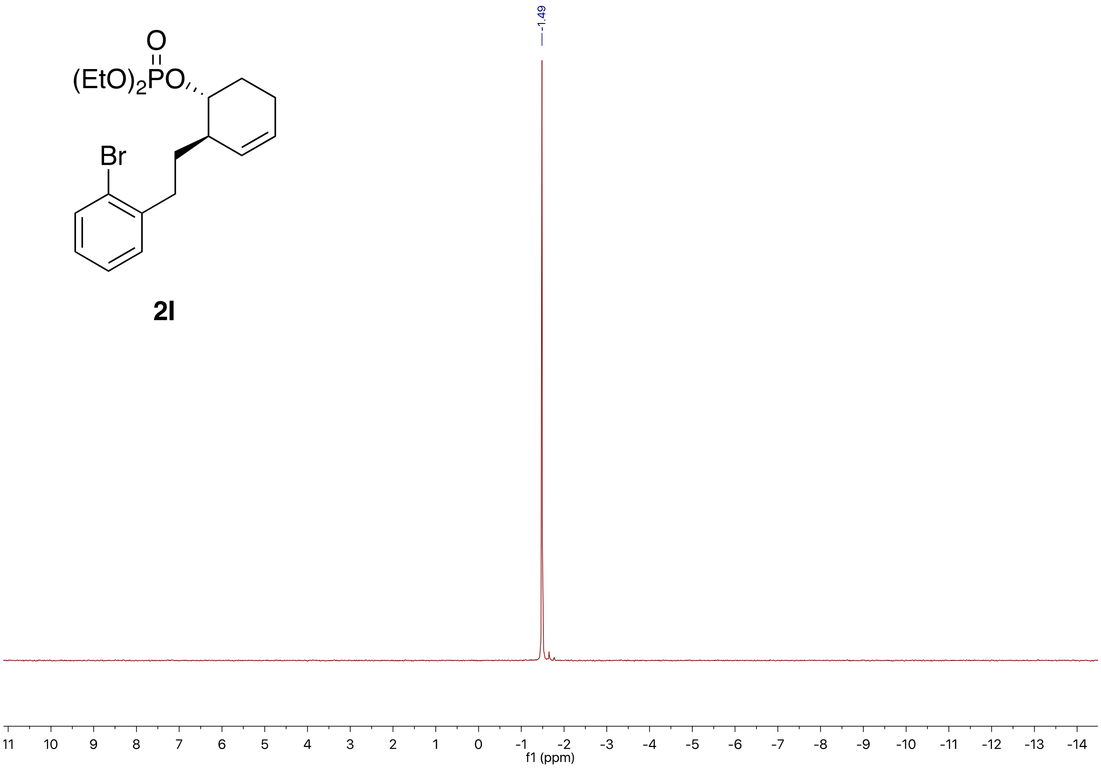

HPLC Trace

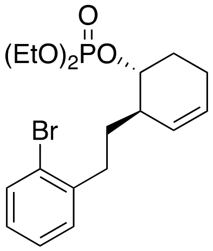

2l

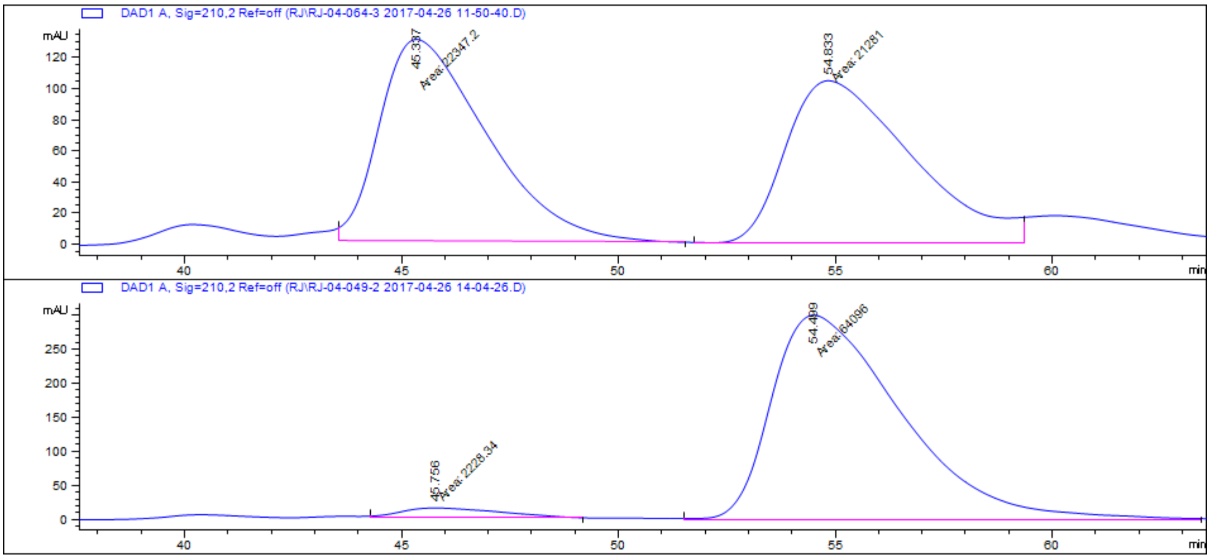

Supplementary Figure 52 - (1*S*,2*R*,5*R*)-2-Benzyl-2-methyl-5-(4-phenylbutyl)cyclopent-3-en-1-yl diethyl phosphate (**4b**) ^{1}H NMR (400 MHz, $CDCl_3$)

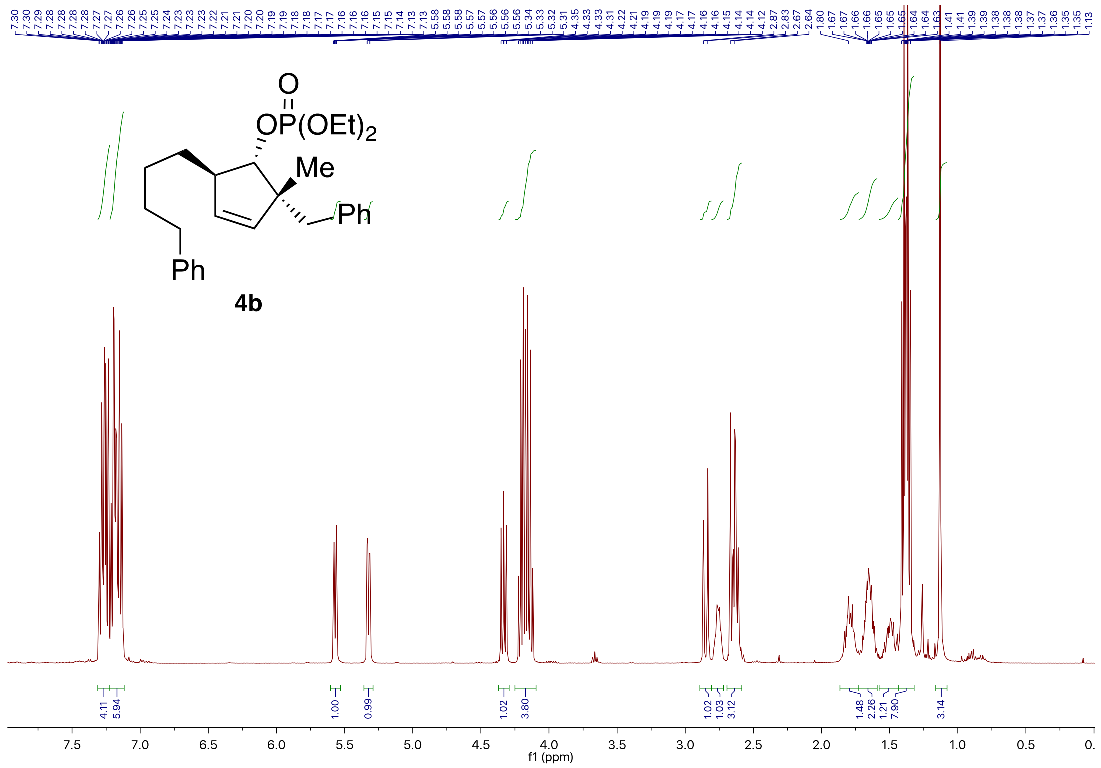

^{13}C NMR (101 MHz, $CDCl_3$)

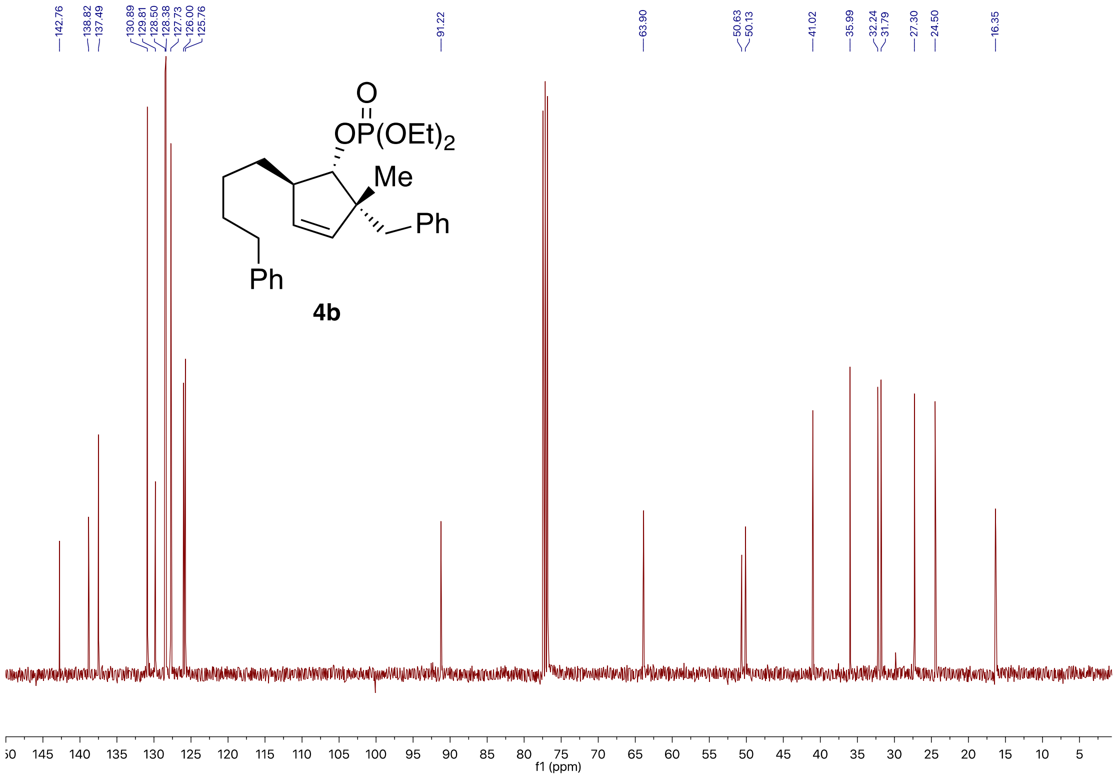

^{31}P NMR (162 MHz, $CDCl_3$)

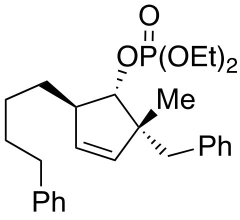

4b

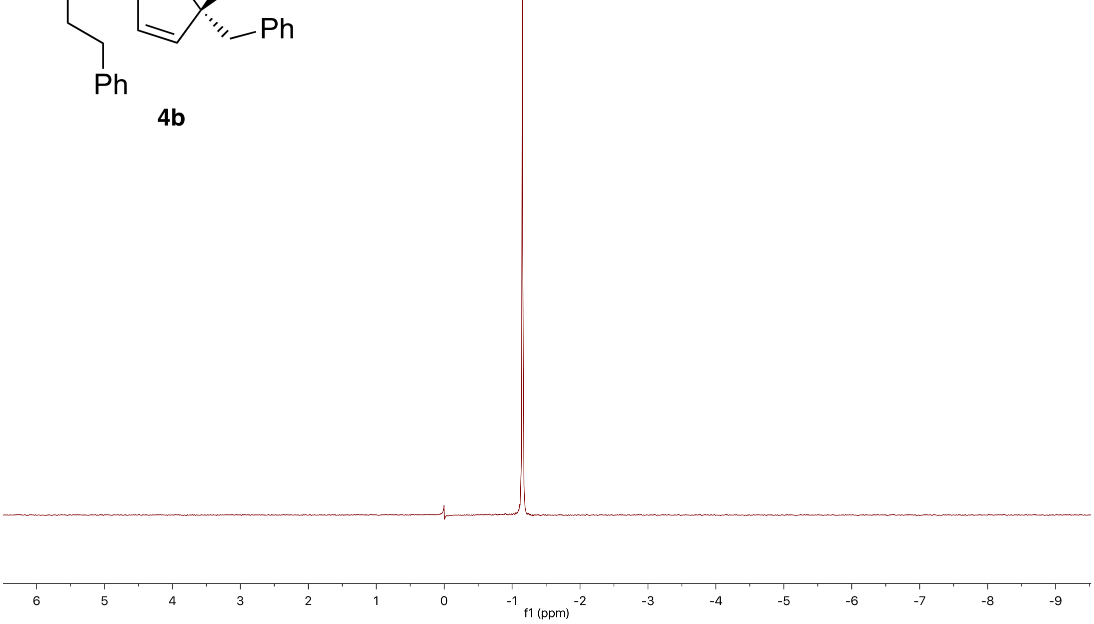

HPLC Trace

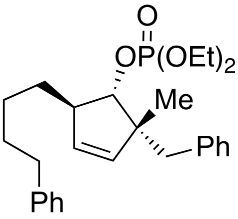

4b

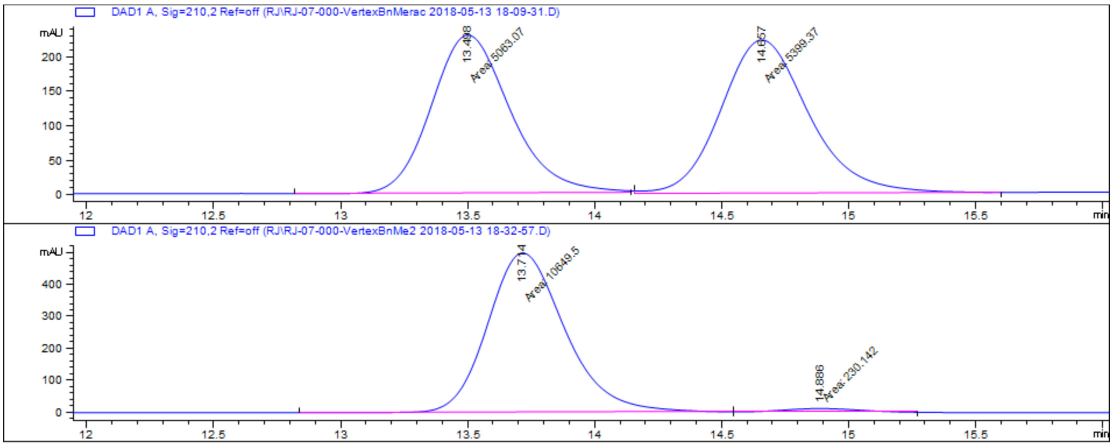

Supplementary Figure 53 - (1*S*,2*R*,5*R*)-2-Benzyl-5-(3-(benzyloxy)propyl)-2-methylcyclopent-3-en-1-yl diethyl phosphate (**4c**) ^{1}H NMR (500 MHz, CDCl$_3$)

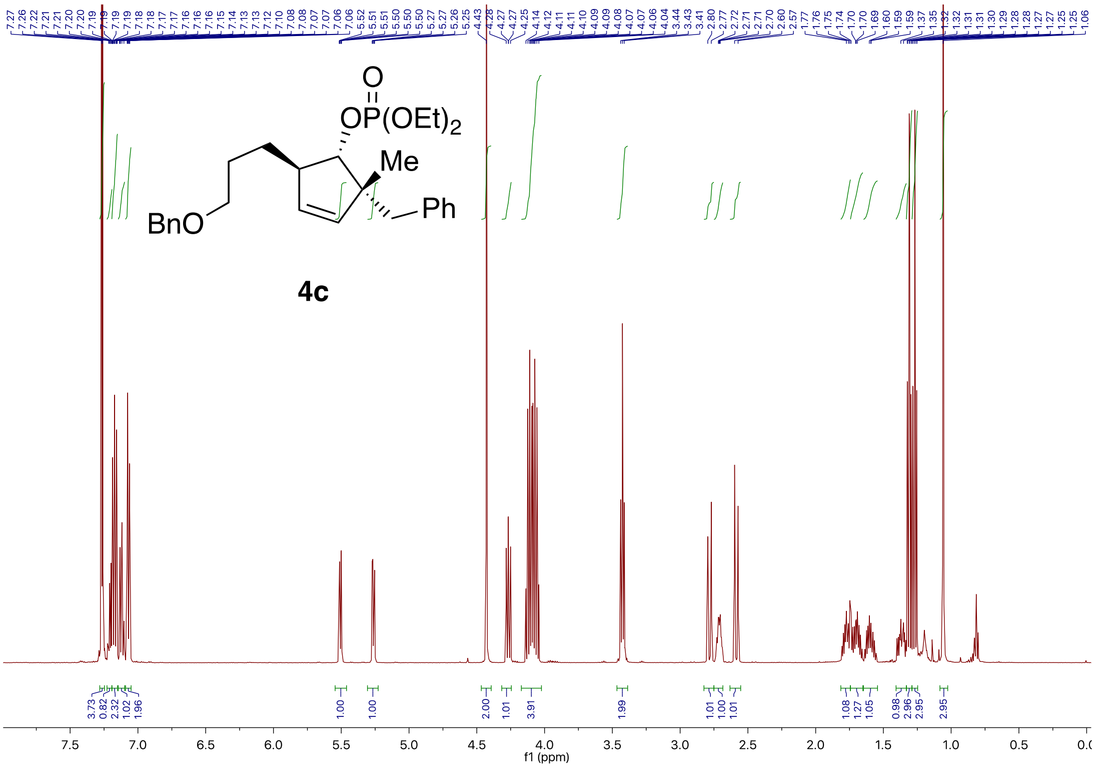

^{13}C NMR (126 MHz, CDCl$_3$)

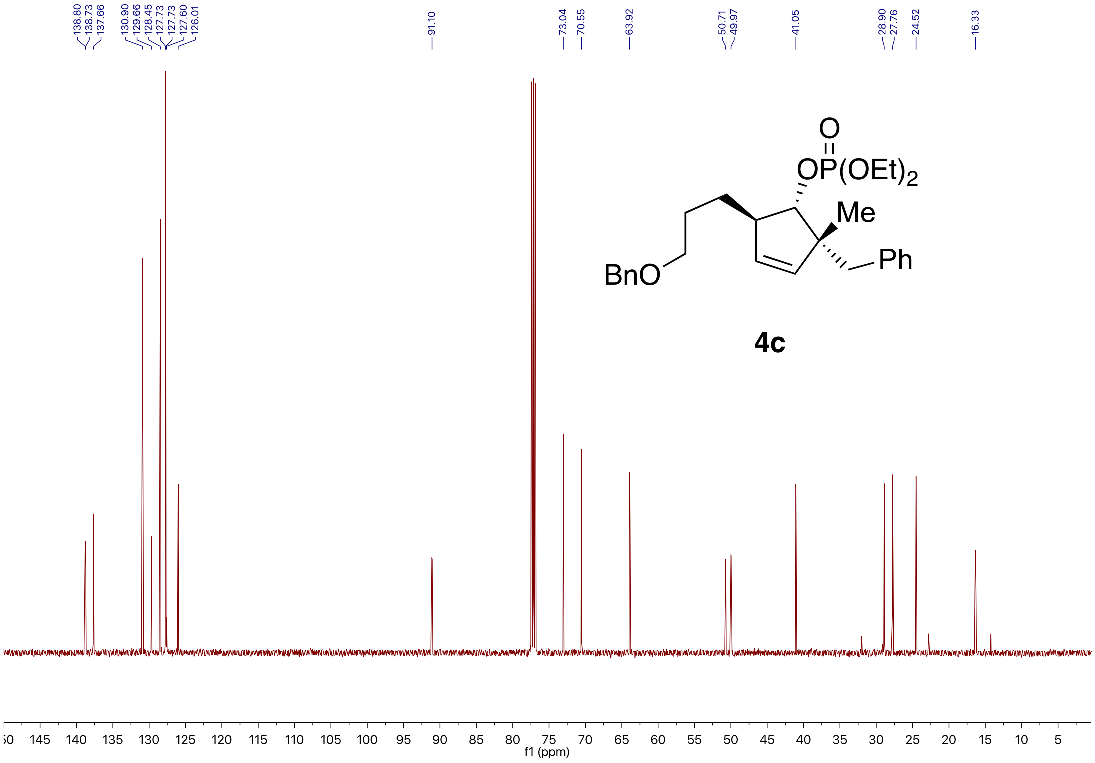

^{31}P NMR (162 MHz, $CDCl_3$)

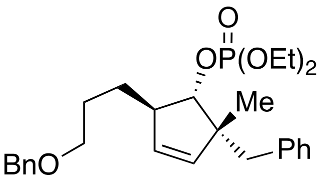

4c

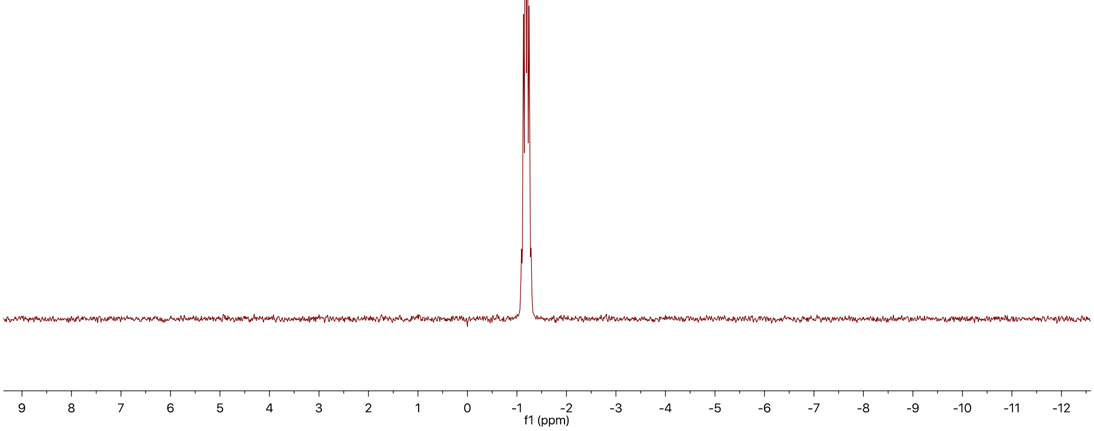

SFC Trace

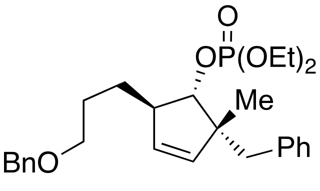

4c

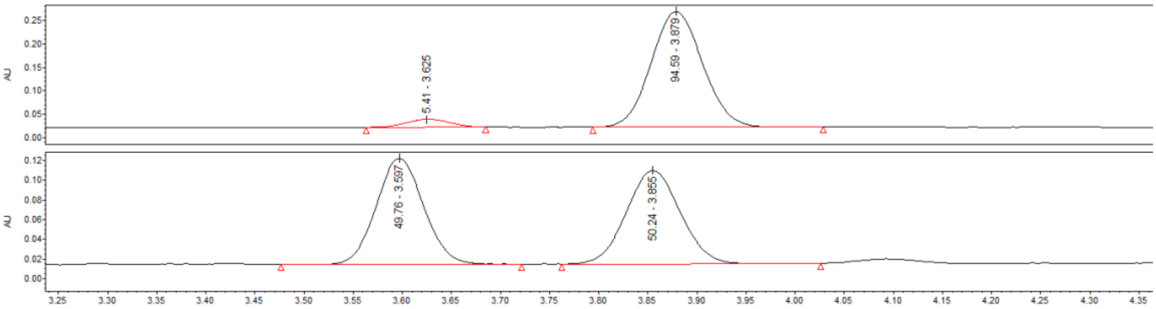

Supplementary Figure 54 - (1*S*,2*R*,5*R*)-2-benzyl-5-(4-bromobutyl)-2-methylcyclopent-3-en-1-yl diethyl phosphate (**4d**) ^{1}H NMR (500 MHz, $CDCl_3$)

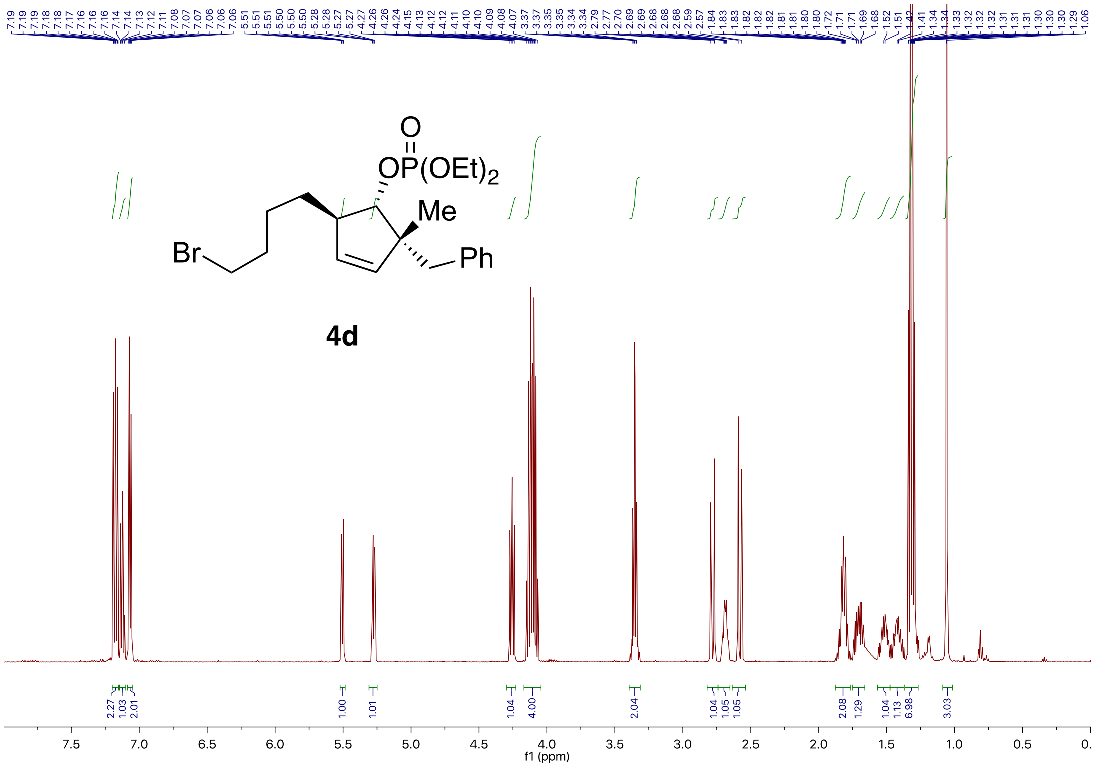

^{13}C NMR (126 MHz, $CDCl_3$)

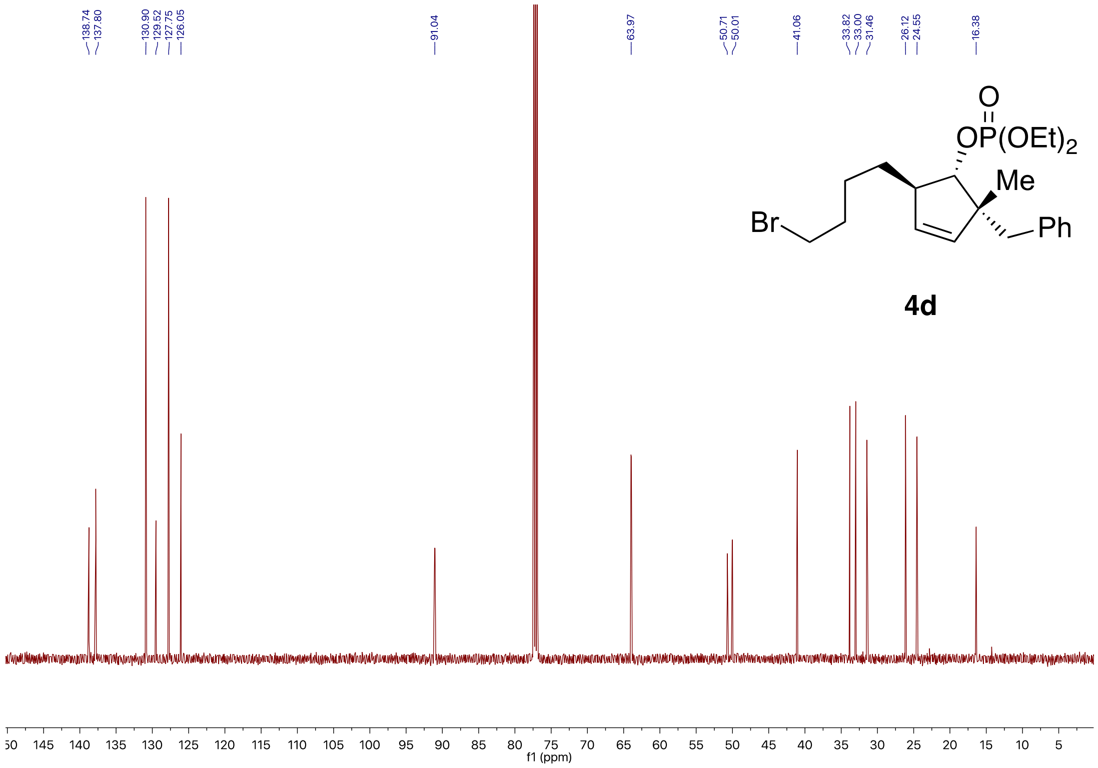

^{31}P NMR (162 MHz, $CDCl_3$)

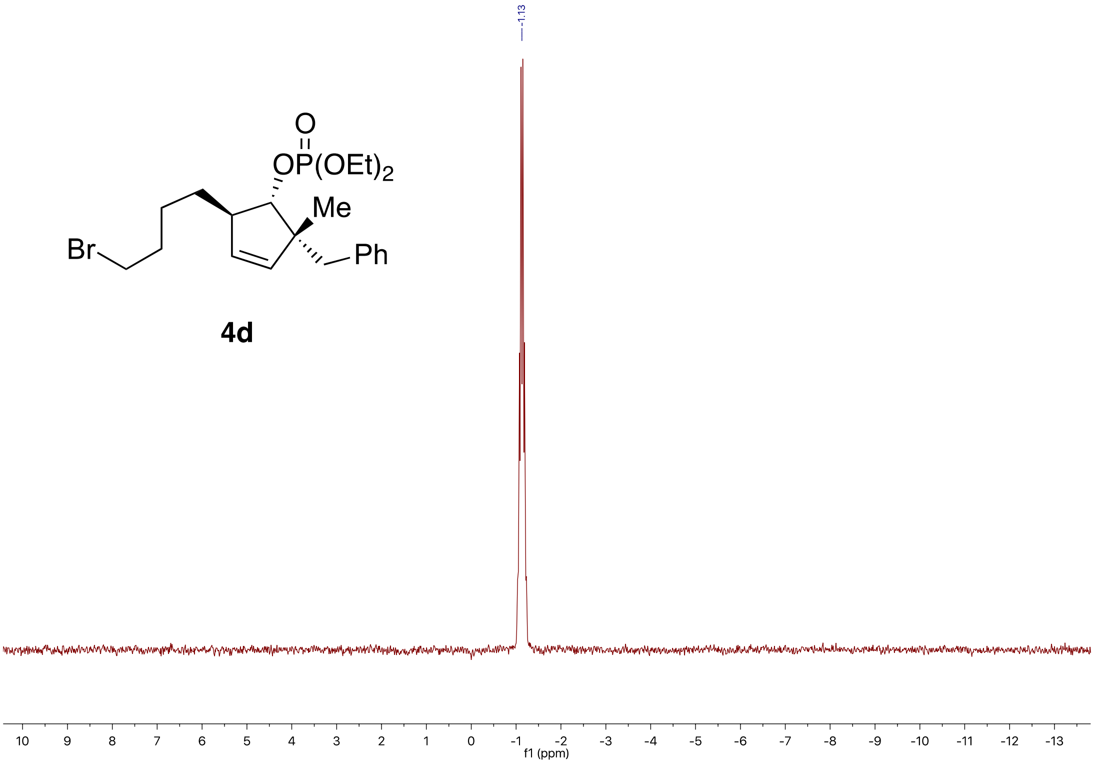

4d

HPLC Trace

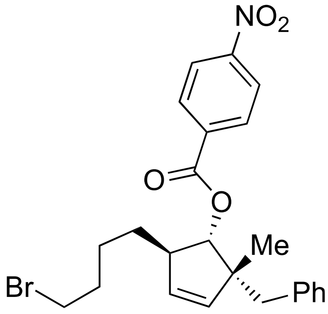

4d'

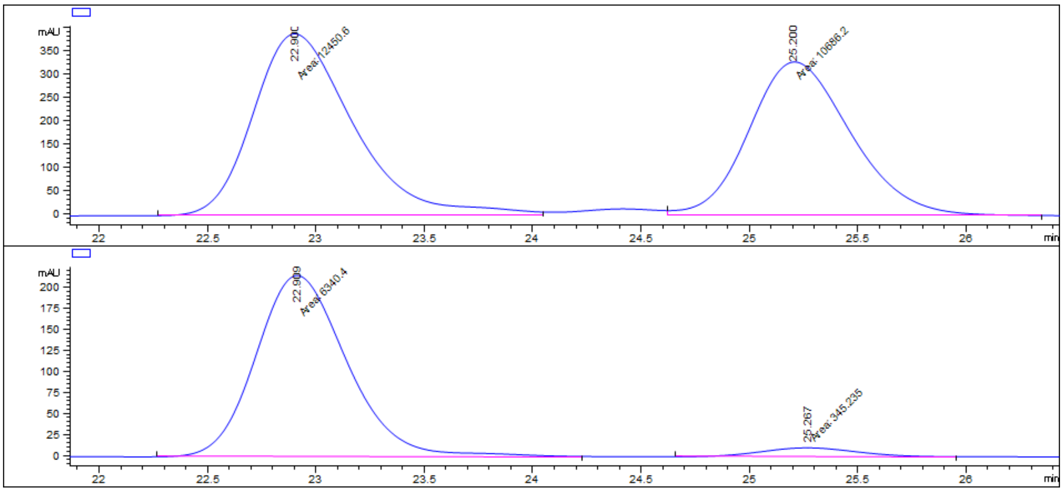

Supplementary Figure 55 - (1*S*,2*R*,5*R*)-2-Benzyl-5-(2-bromo-5-fluorophenethyl)-2-methylcyclopent-3-en-1-yl diethyl phosphate (**4e**) ^{1}H NMR (500 MHz, $CDCl_3$)

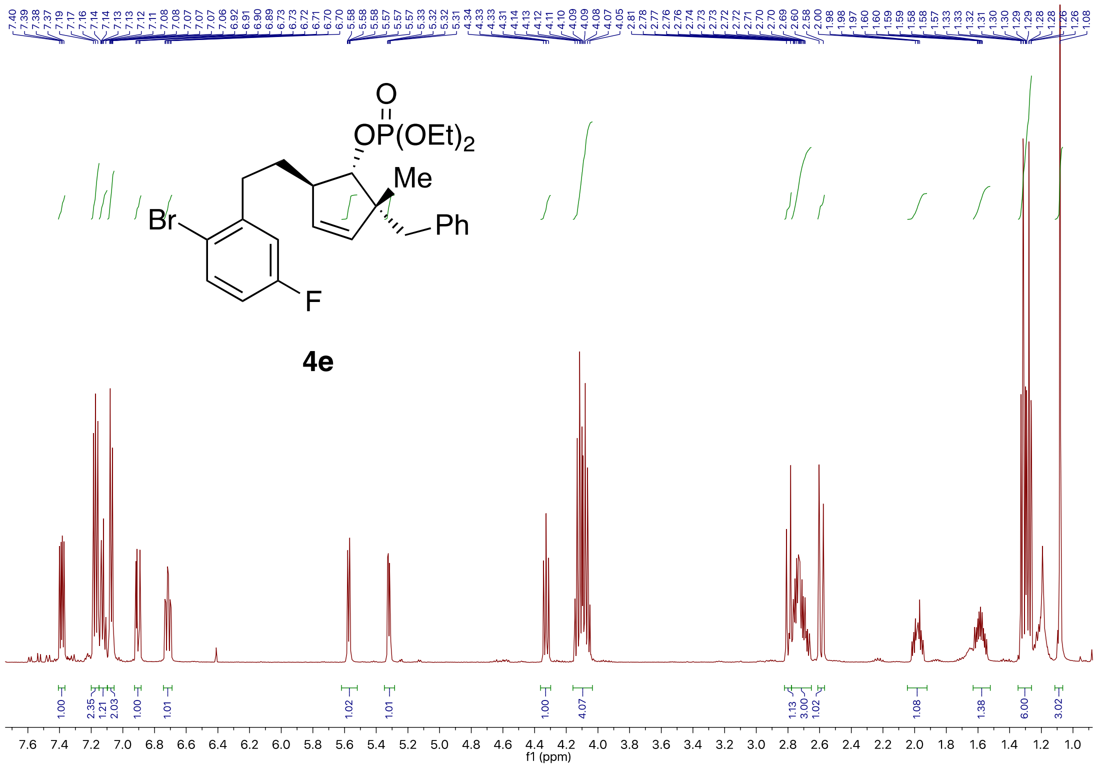

^{13}C NMR (126 MHz, $CDCl_3$)

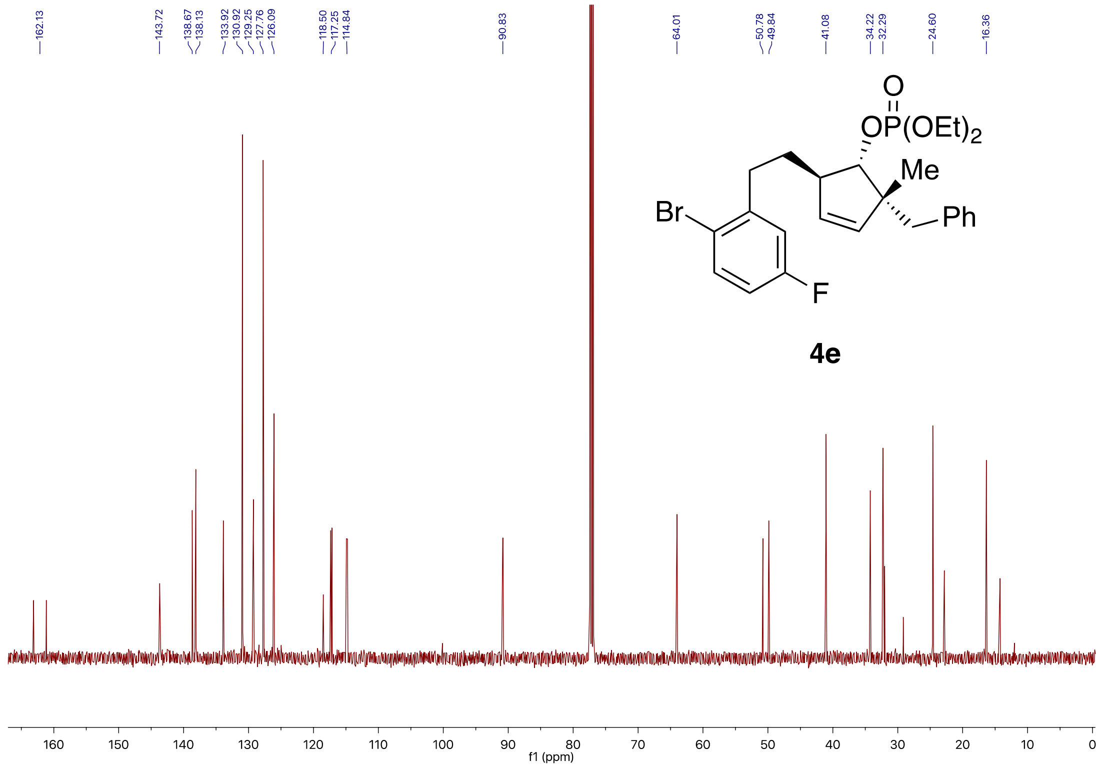

^{31}P NMR (162 MHz, $CDCl_3$)

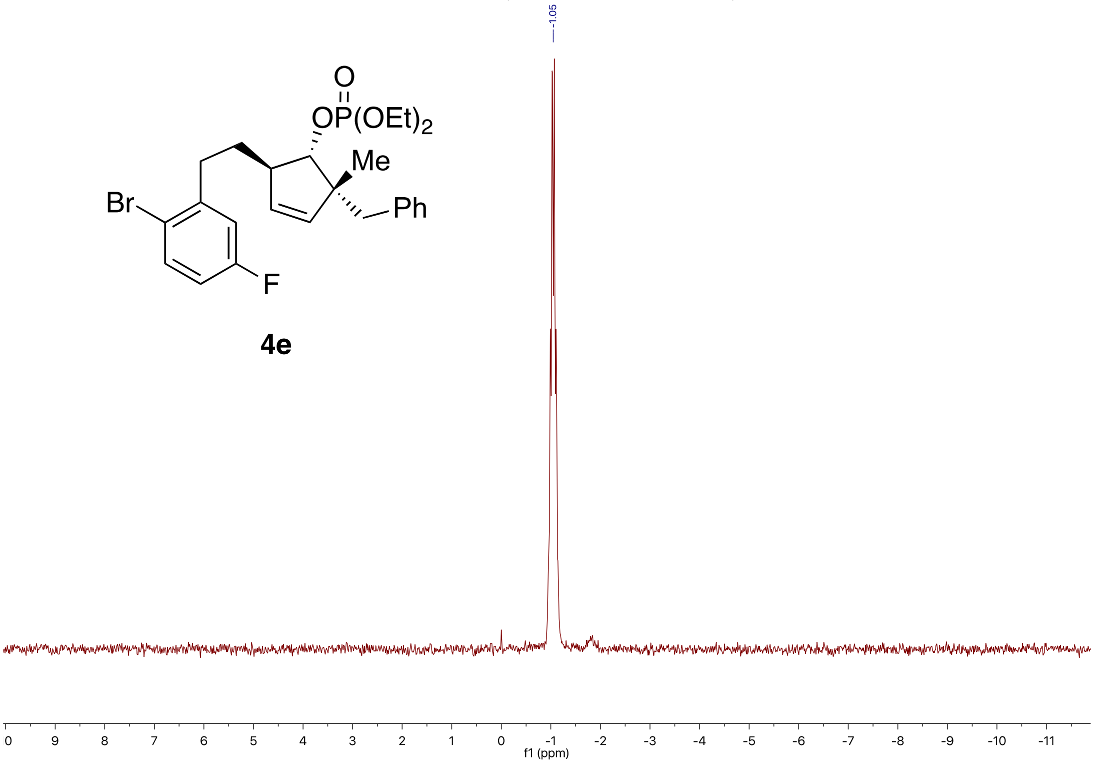

4e

SFC Trace

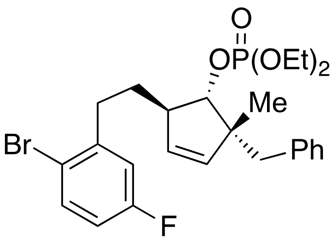

4e

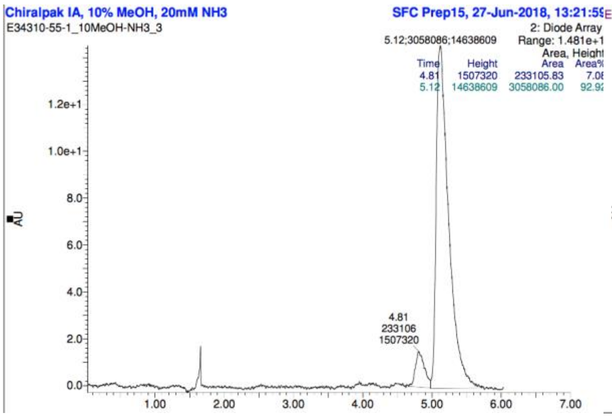

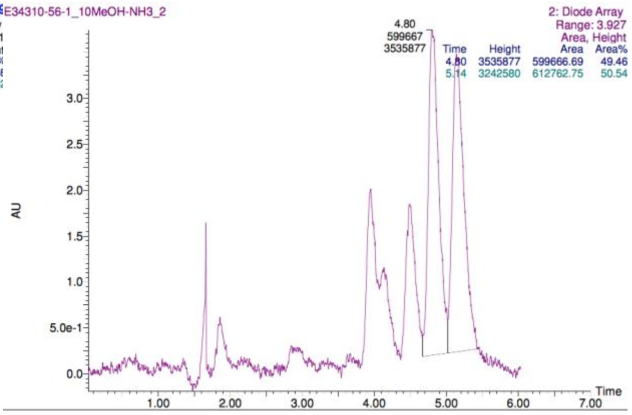

Supplementary Figure 56 - (1*S*,2*R*,5*R*)-2-Benzyl-5-(4-(dibenzylamino)butyl)-2-methylcyclopent-3-en-1-yl diethyl phosphate (**4f**) ^{1}H NMR (500 MHz, $CDCl_3$)

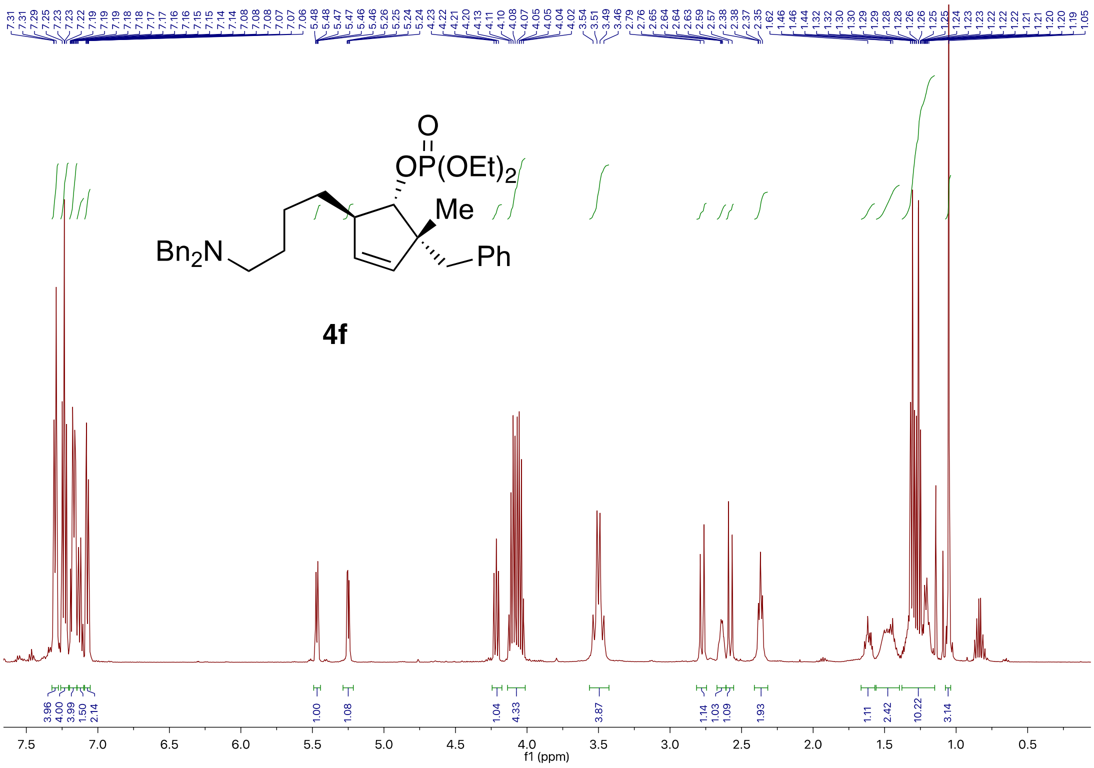

^{13}C NMR (126 MHz, $CDCl_3$)

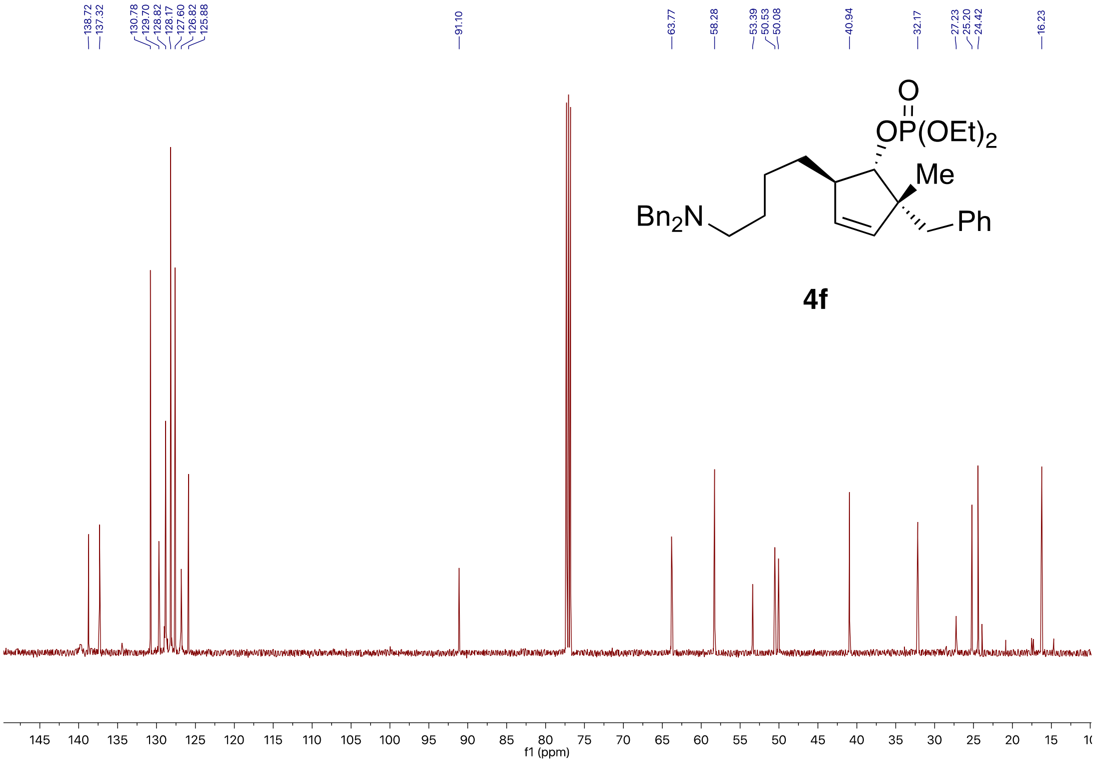

^{31}P NMR (162 MHz, $CDCl_3$)

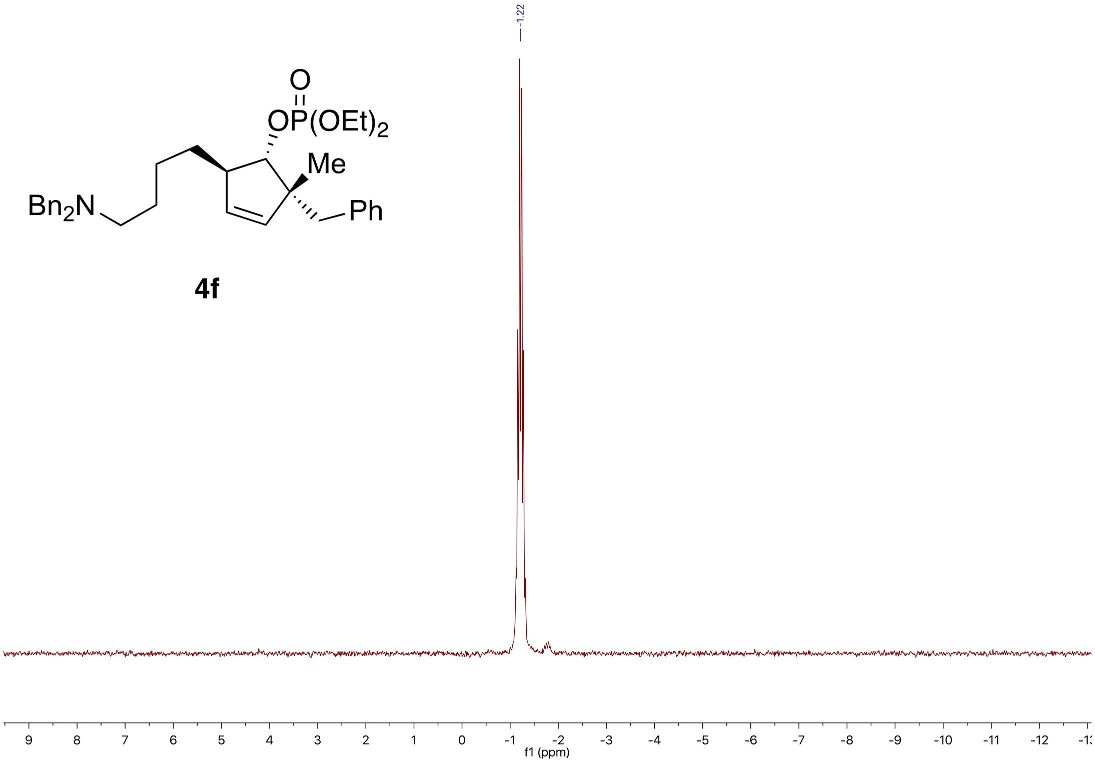

SFC Trace

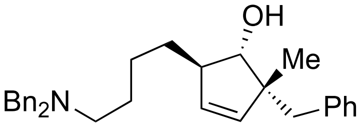

4f'

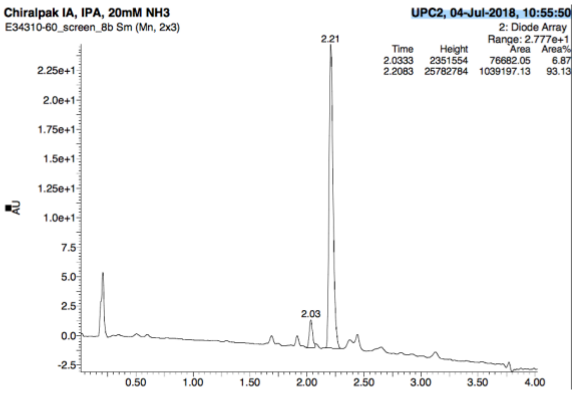

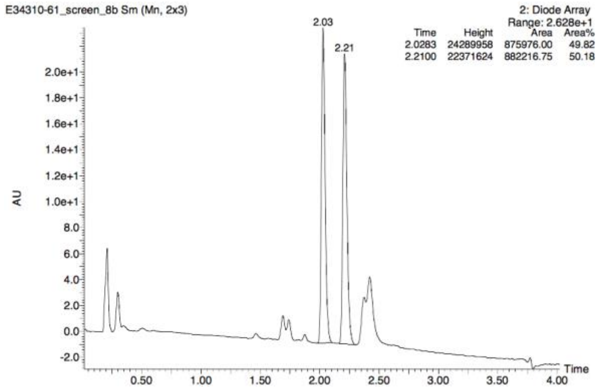

Supplementary Figure 57 - (1*S*,5*R*)-2,2-Dimethyl-5-(4-phenylbutyl)cyclopent-3-en-1-yl diethyl phosphate (**5b**) ^{1}H NMR (400 MHz, $CDCl_3$)

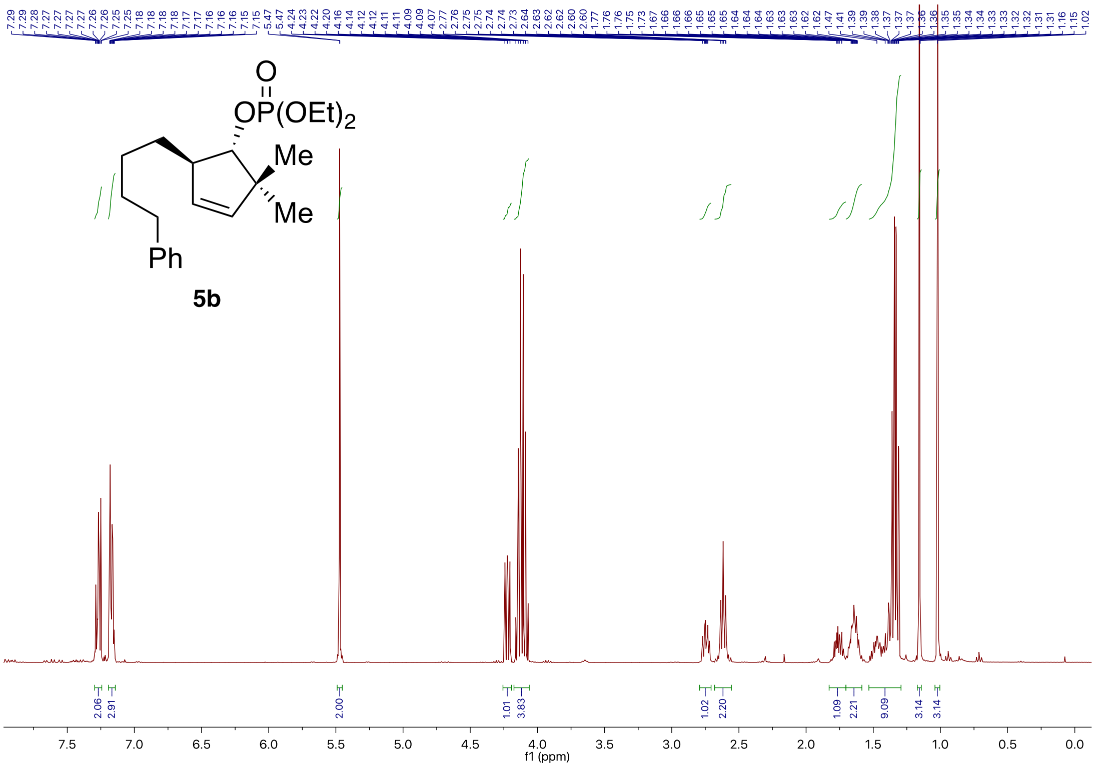

^{13}C NMR (101 MHz, $CDCl_3$)

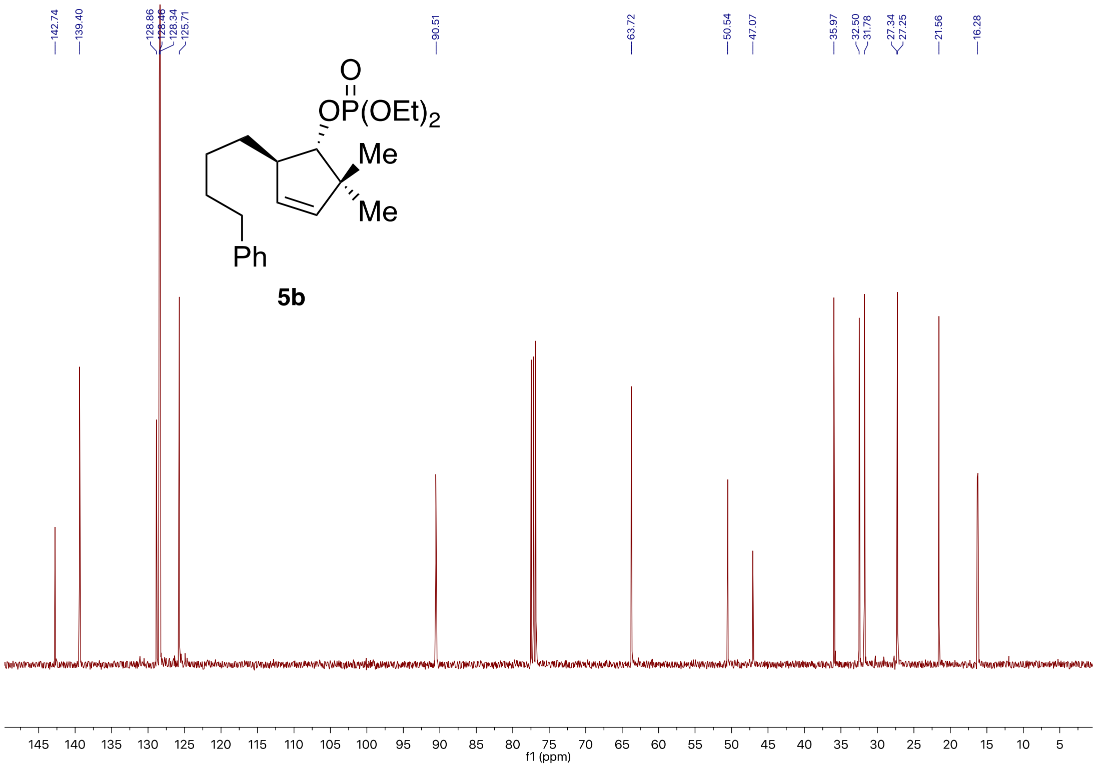

^{31}P NMR (162 MHz, $CDCl_3$)

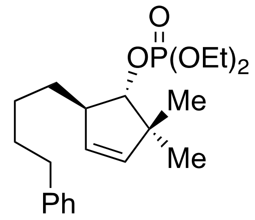

5b

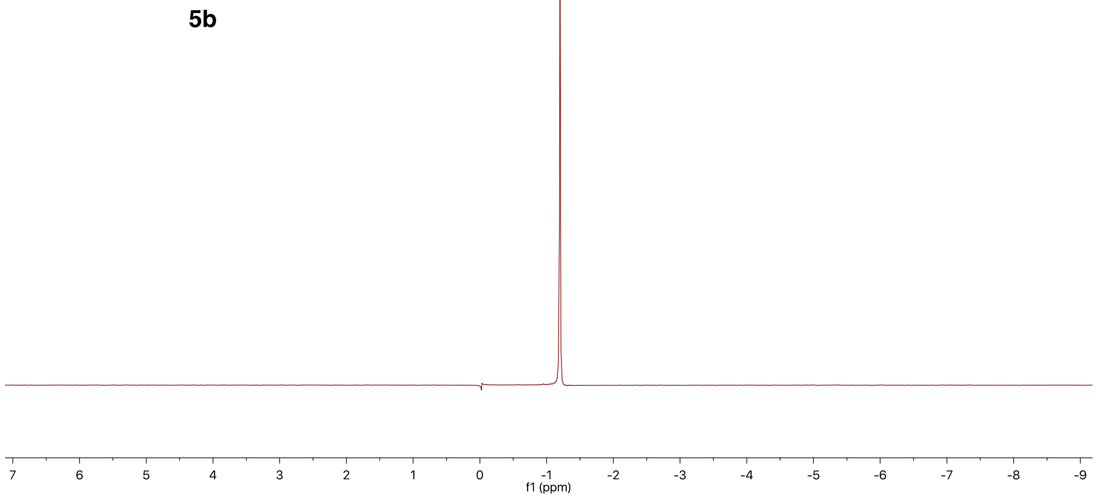

HPLC Trace

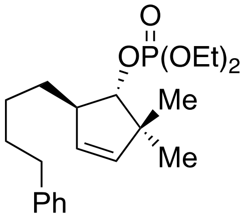

5b

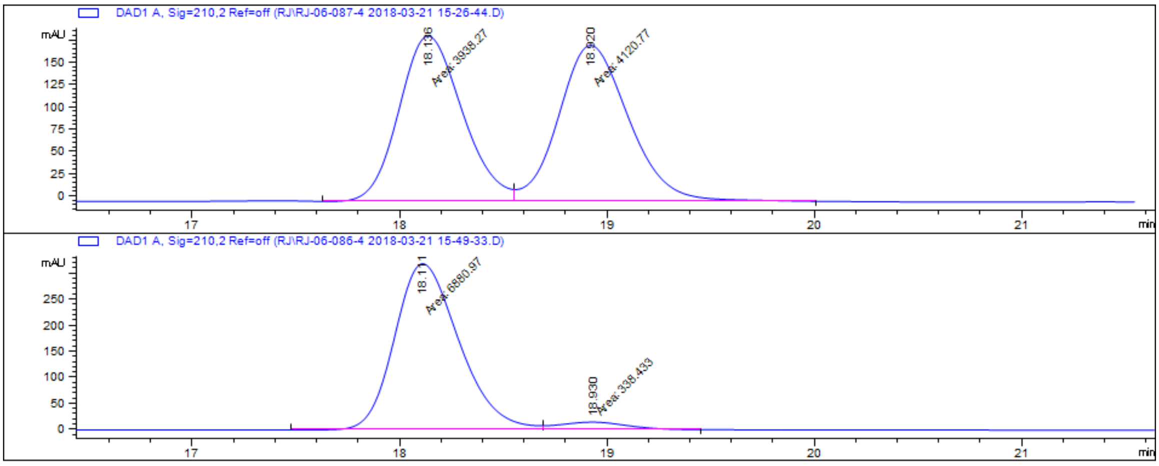

Supplementary Figure 58 - (1*S*,2*R*,5*R*)-2-Allyl-2-methyl-5-(4-phenylbutyl)cyclopent-3-en-1-yl diethyl phosphate **(6b)** ^{1}H NMR (400 MHz, $CDCl_3$)

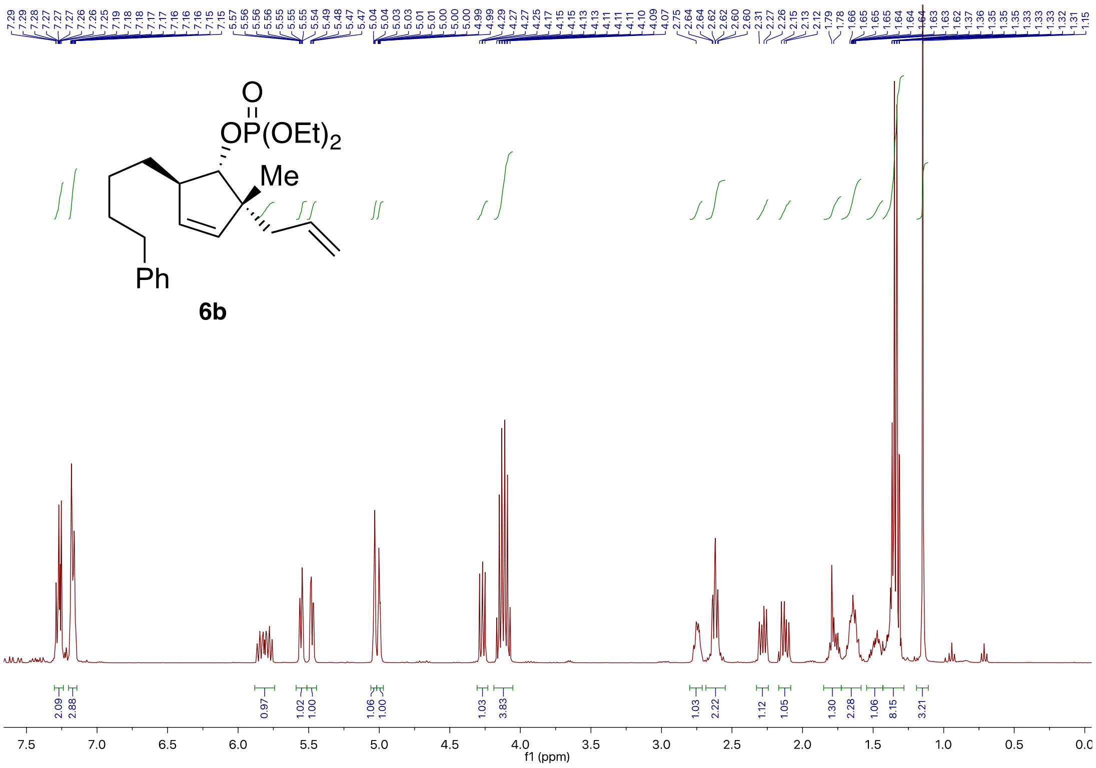

^{13}C NMR (101 MHz, $CDCl_3$)

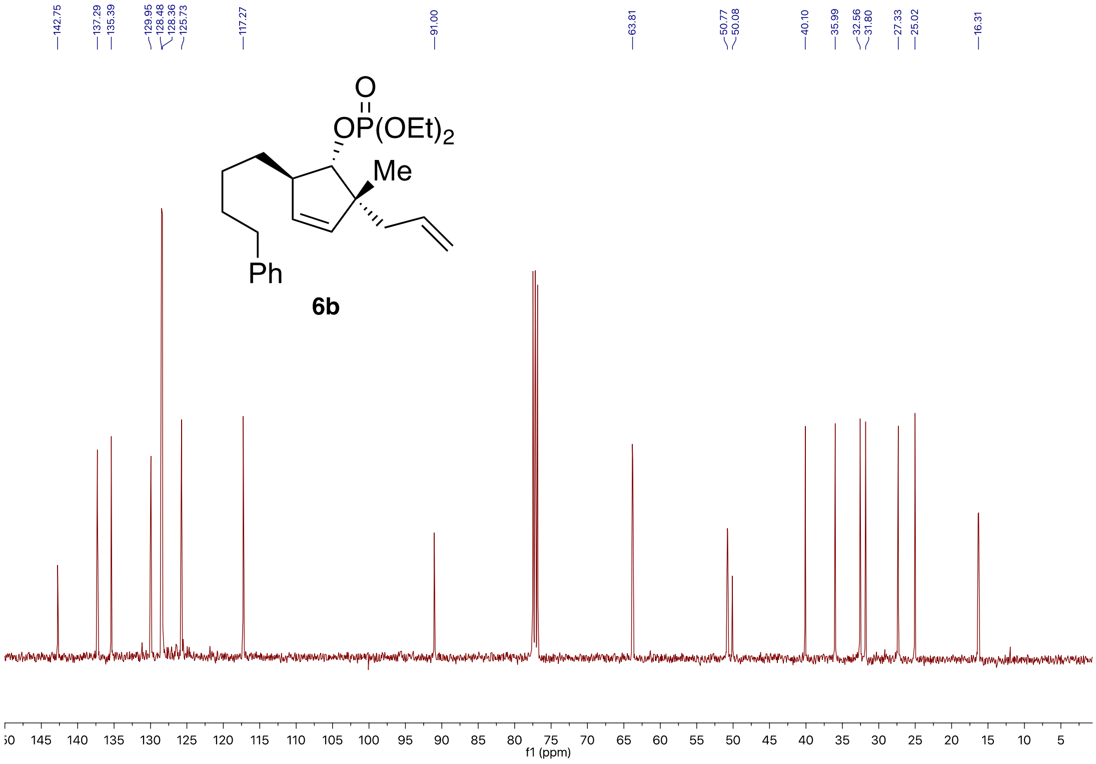

^{31}P NMR (162 MHz, $CDCl_3$)

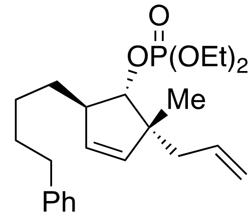

6b

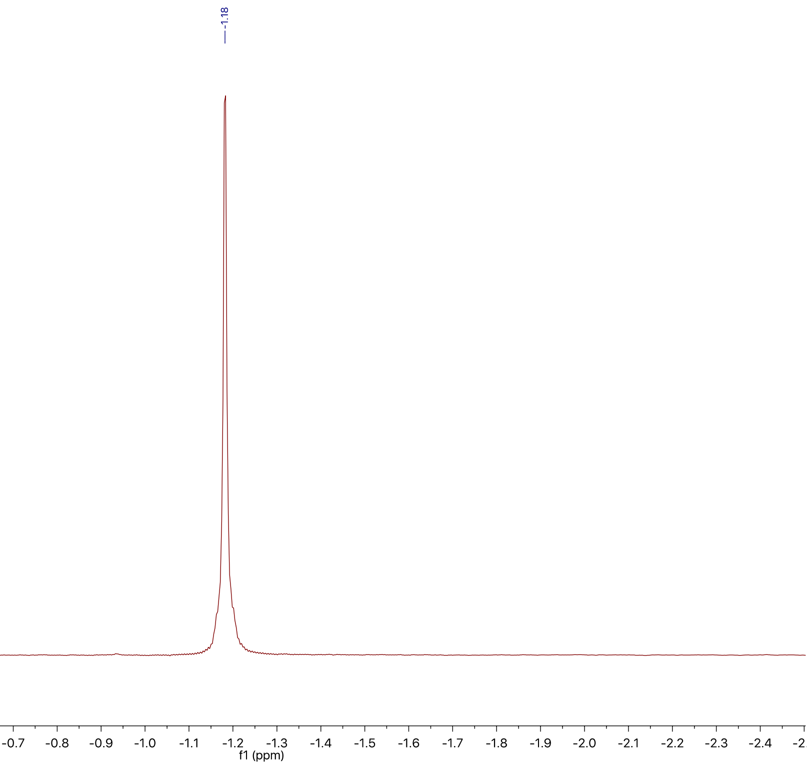

HPLC Trace

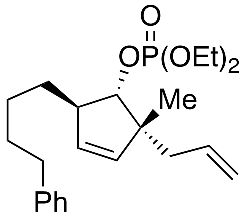

6b

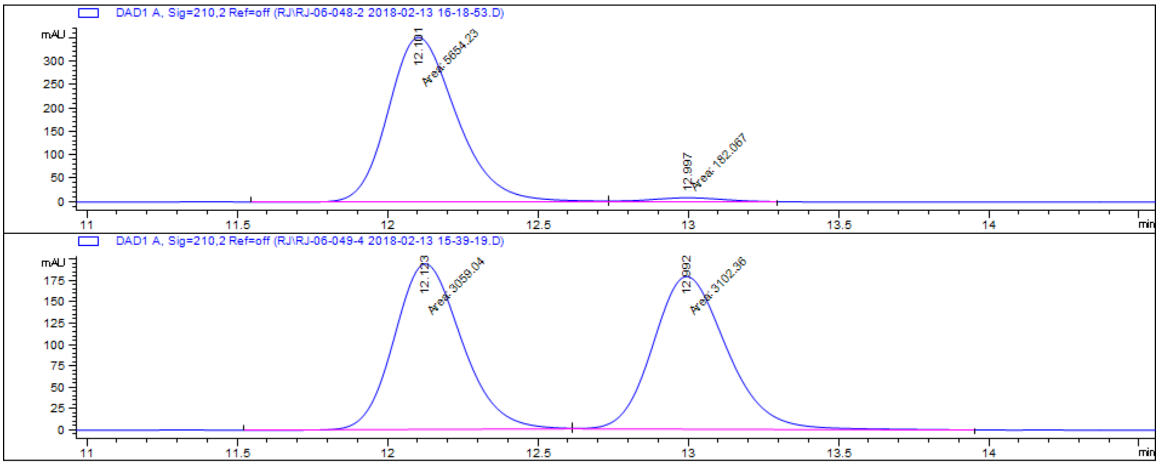

Supplementary Figure 59 - (1*S*,2*R*,5*R*)-2-(2-chlorobenzyl)-2-methyl-5-(4-phenylbutyl)cyclopent-3-en-1-yl diethyl phosphate (**7b**) ^{1}H NMR (400 MHz, $CDCl_3$)

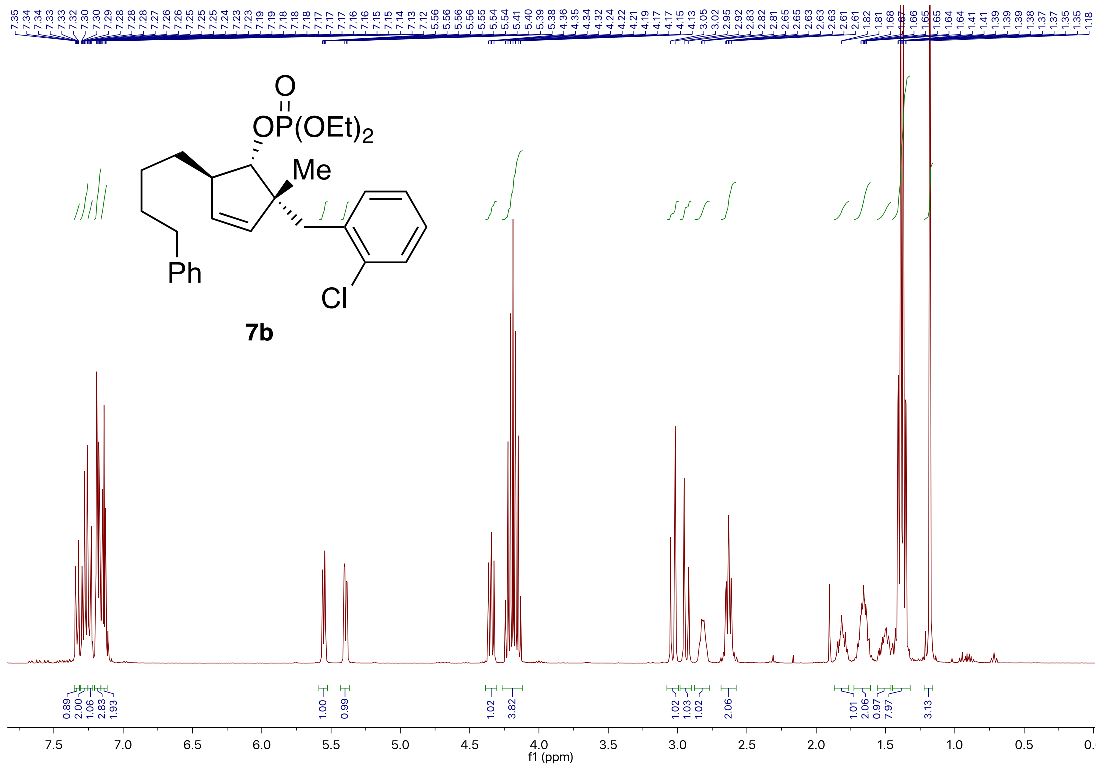

^{13}C NMR (101 MHz, $CDCl_3$)

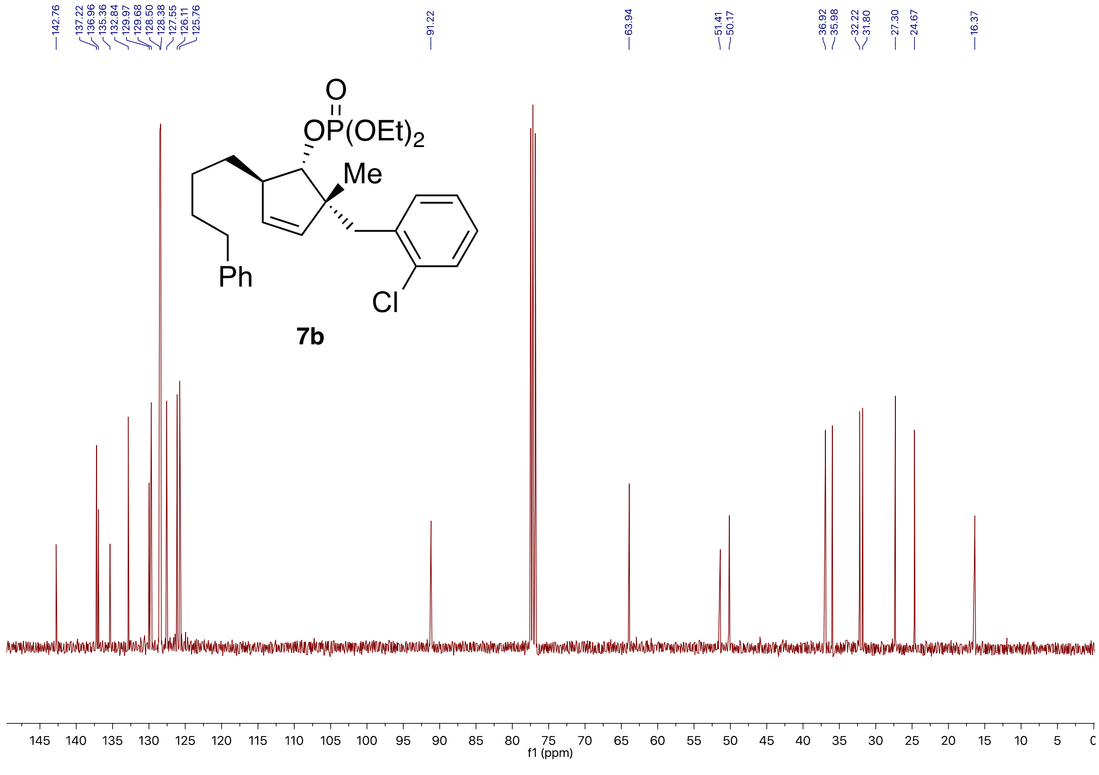

^{31}P NMR (162 MHz, $CDCl_3$)

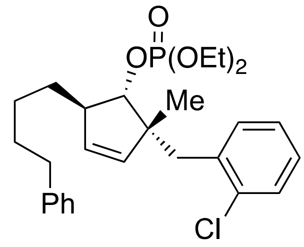

7b

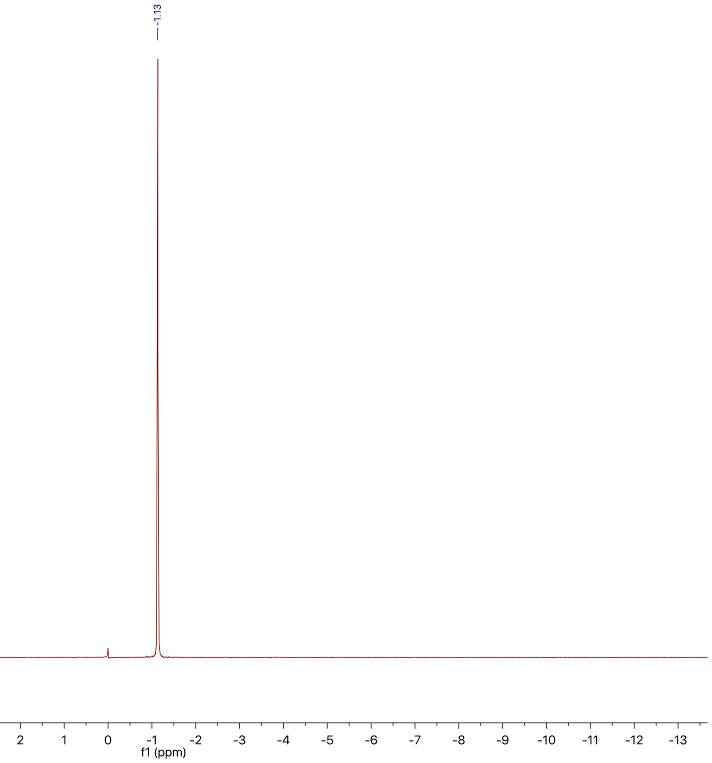

HPLC Trace

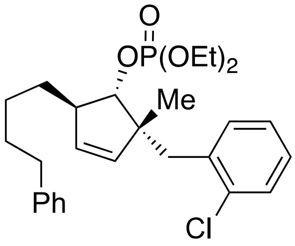

7b

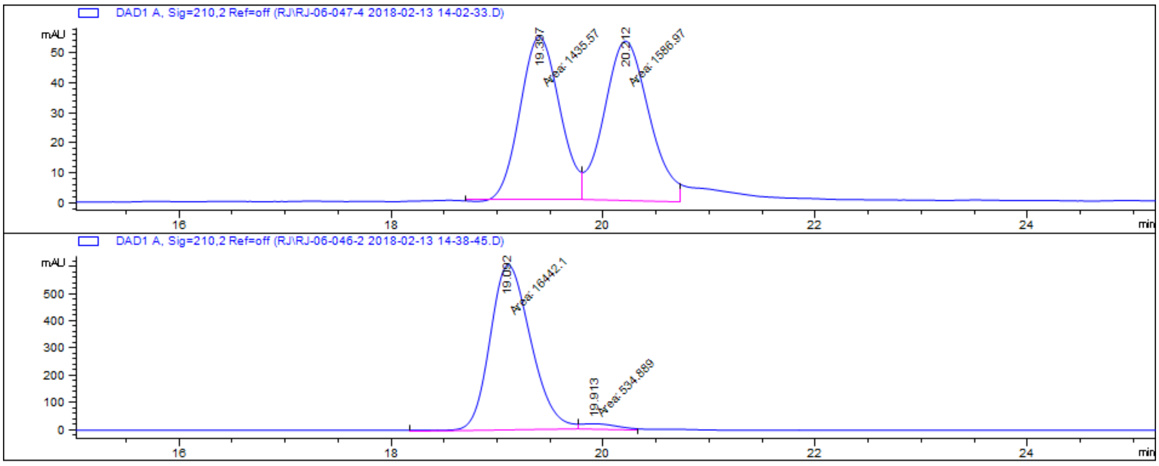

Supplementary Figure 60 - (1*S*,2*S*,5*R*)-2-((benzyloxy)methyl)-2-methyl-5-(4-phenylbutyl)cyclopent-3-en-1-yl diethyl phosphate (**8b**) ^{1}H NMR (400 MHz, $CDCl_3$)

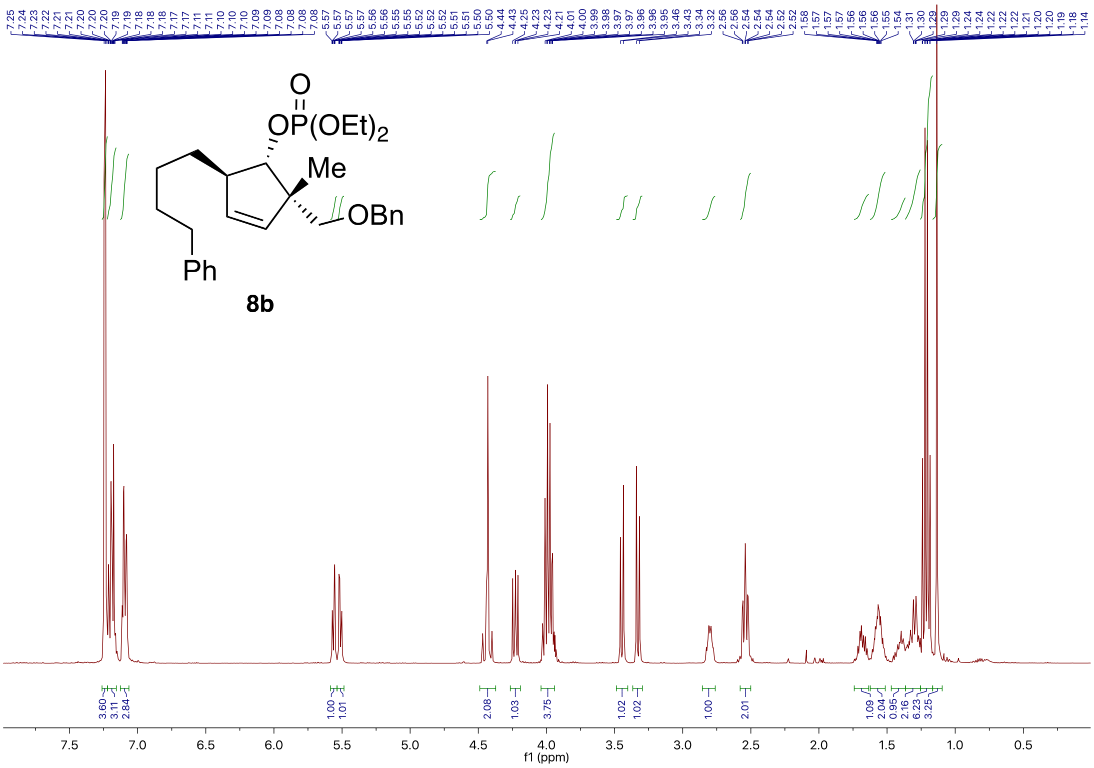

^{13}C NMR (101 MHz, $CDCl_3$)

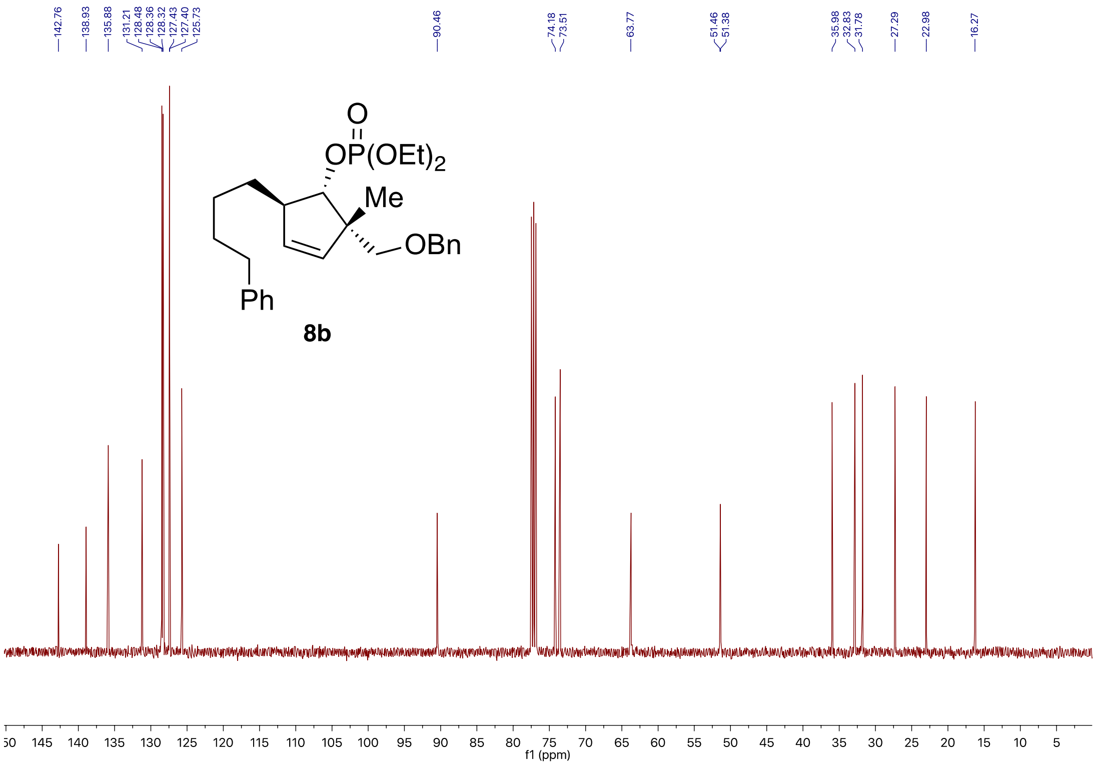

^{31}P NMR (162 MHz, $CDCl_3$)

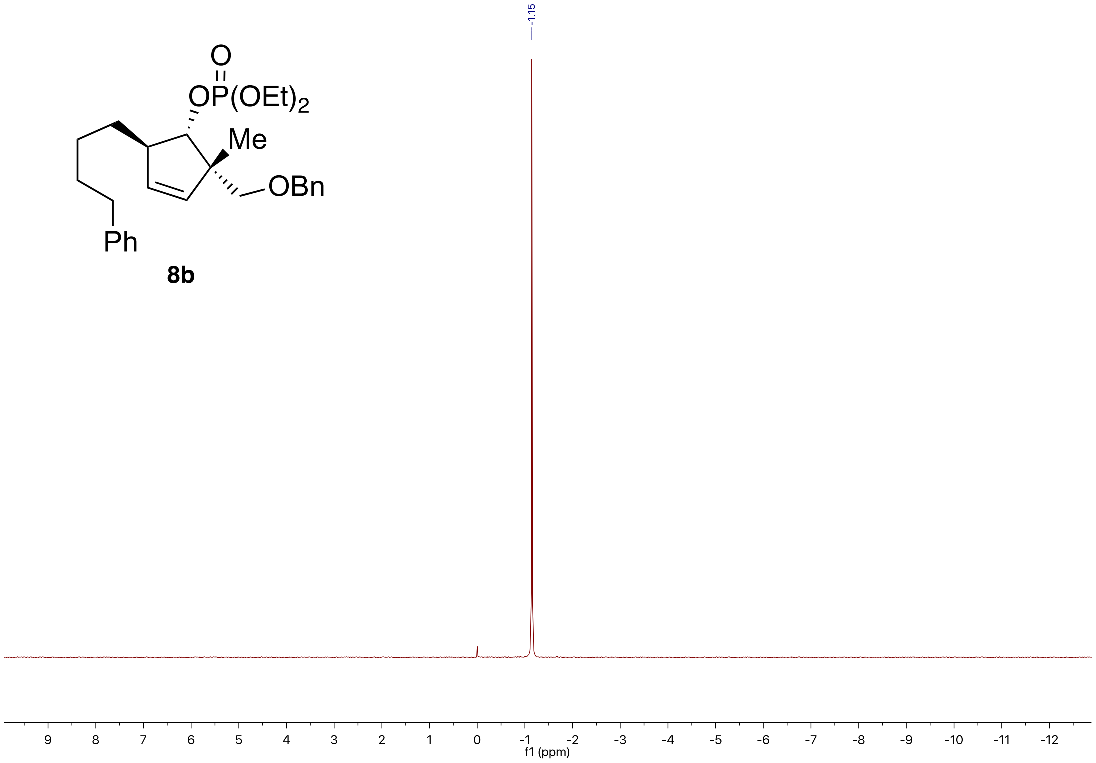

HPLC Trace

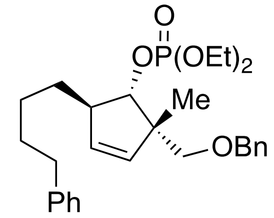

8b

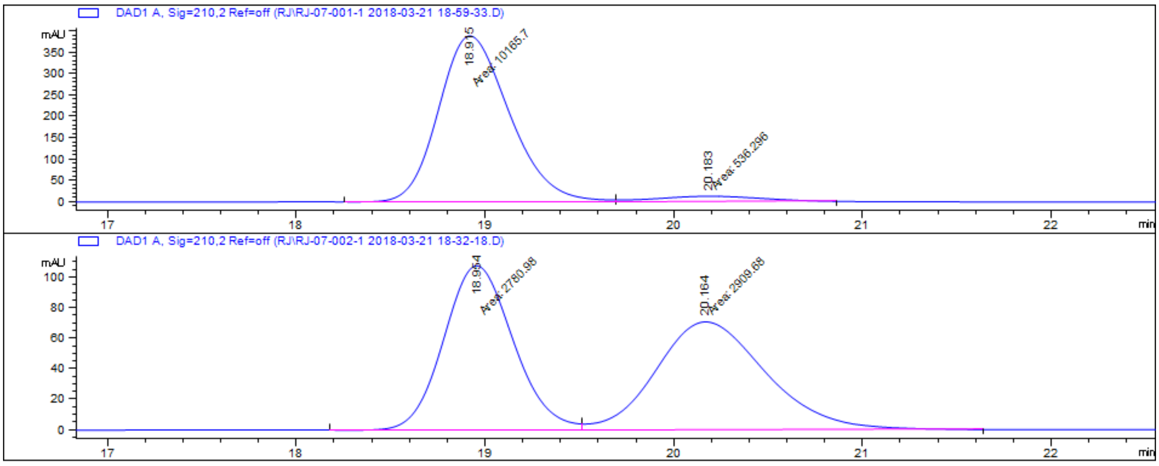

Supplementary Figure 61 - Diethyl ((1*S*,4*R*,5*S*)-4-(4-phenylbutyl)-2',3'-dihydrospiro[cyclopentane-1,1'-inden]-2-en-5-yl) phosphate **(9b)** ^{1}H NMR (400 MHz, $CDCl_3$)

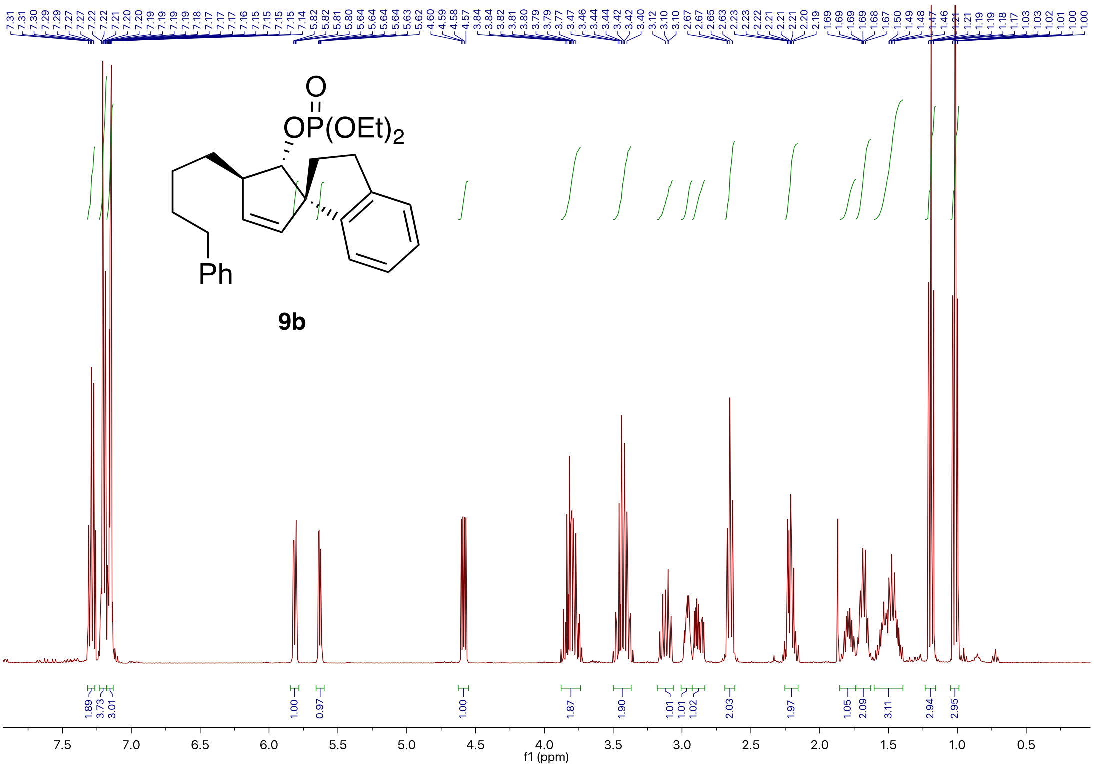

^{13}C NMR (101 MHz, $CDCl_3$)

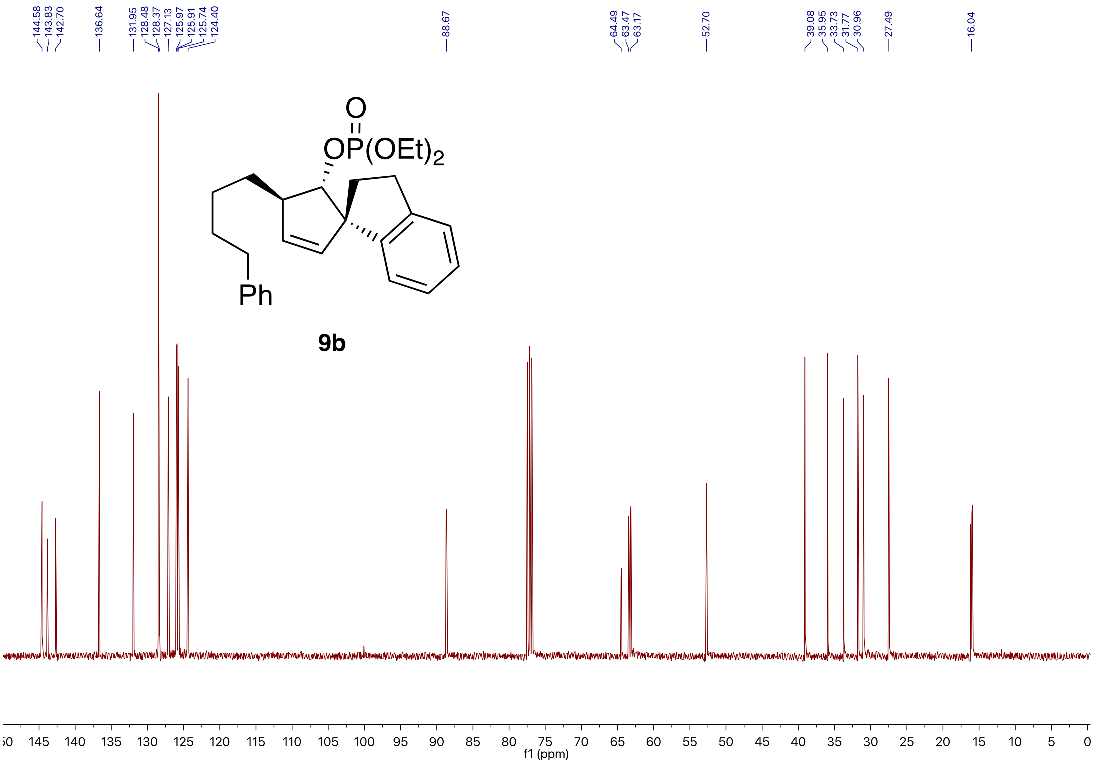

^{31}P NMR (162 MHz, $CDCl_3$)

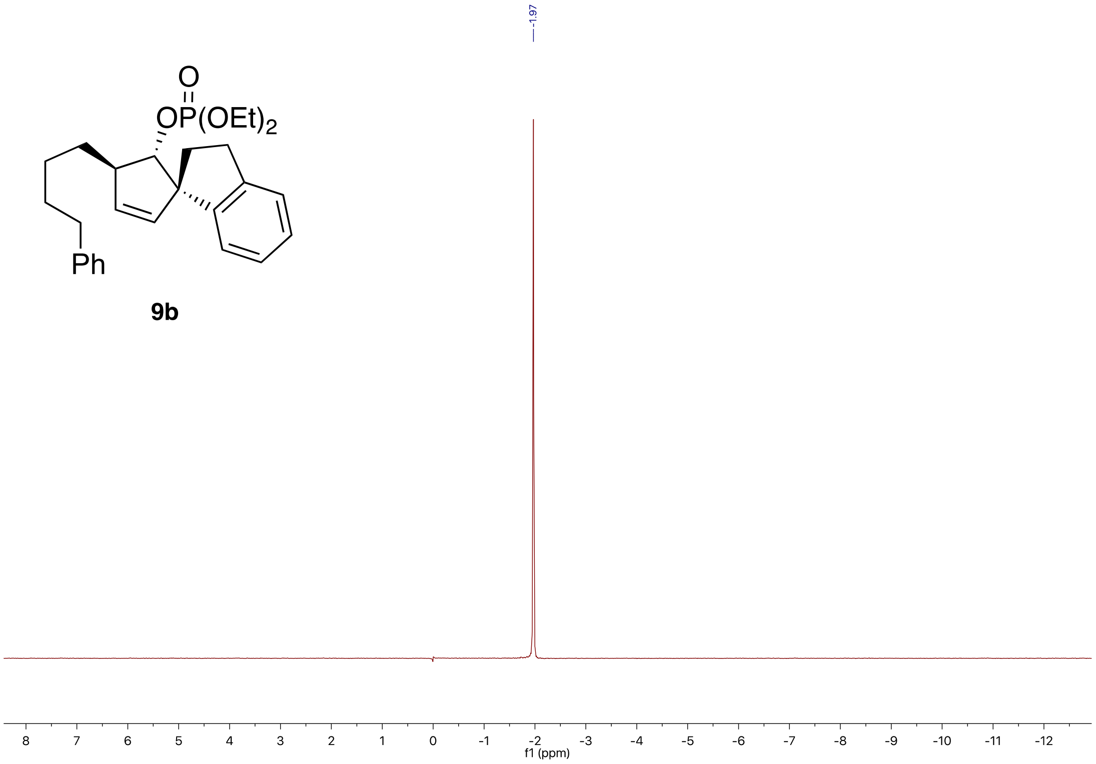

HPLC Trace

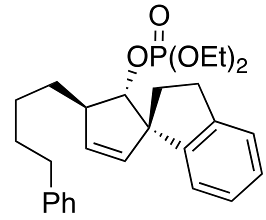

9b

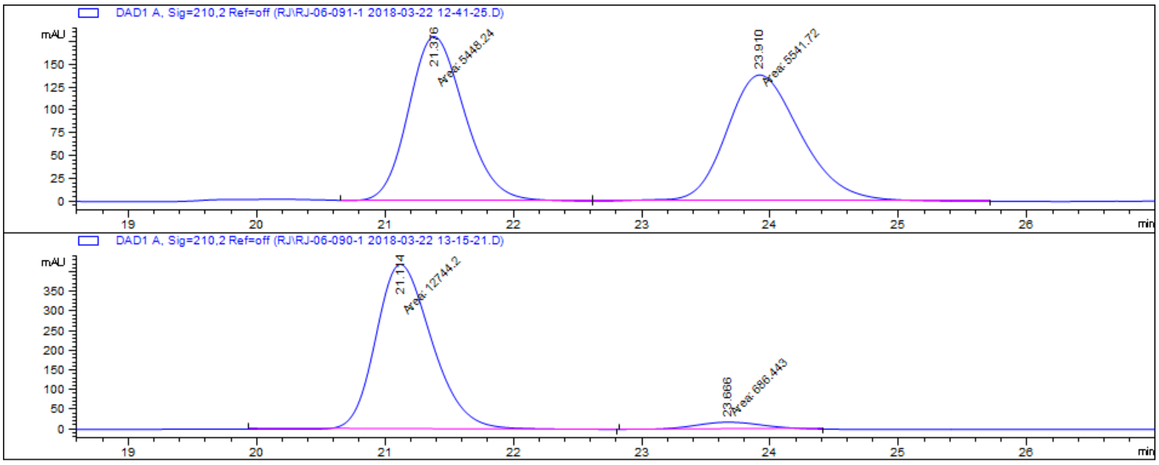

Supplementary Figure 62 - Diethyl ((4*S*,4'*R*,5'*S*)-4'-(4-phenylbutyl)spiro[chromane-4,1'-cyclopentan]-2'-en-5'-yl) phosphate **(10b)** ^{1}H NMR (400 MHz, CDCl$_3$)

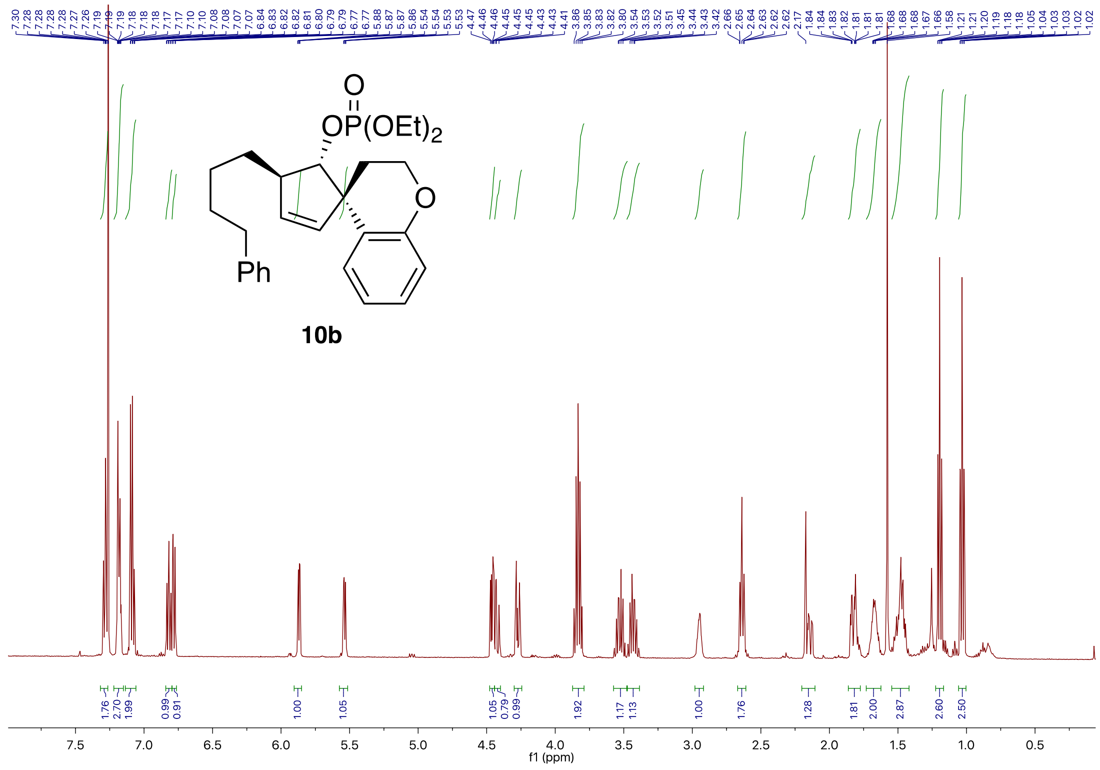

^{13}C NMR (125 MHz, CDCl$_3$)

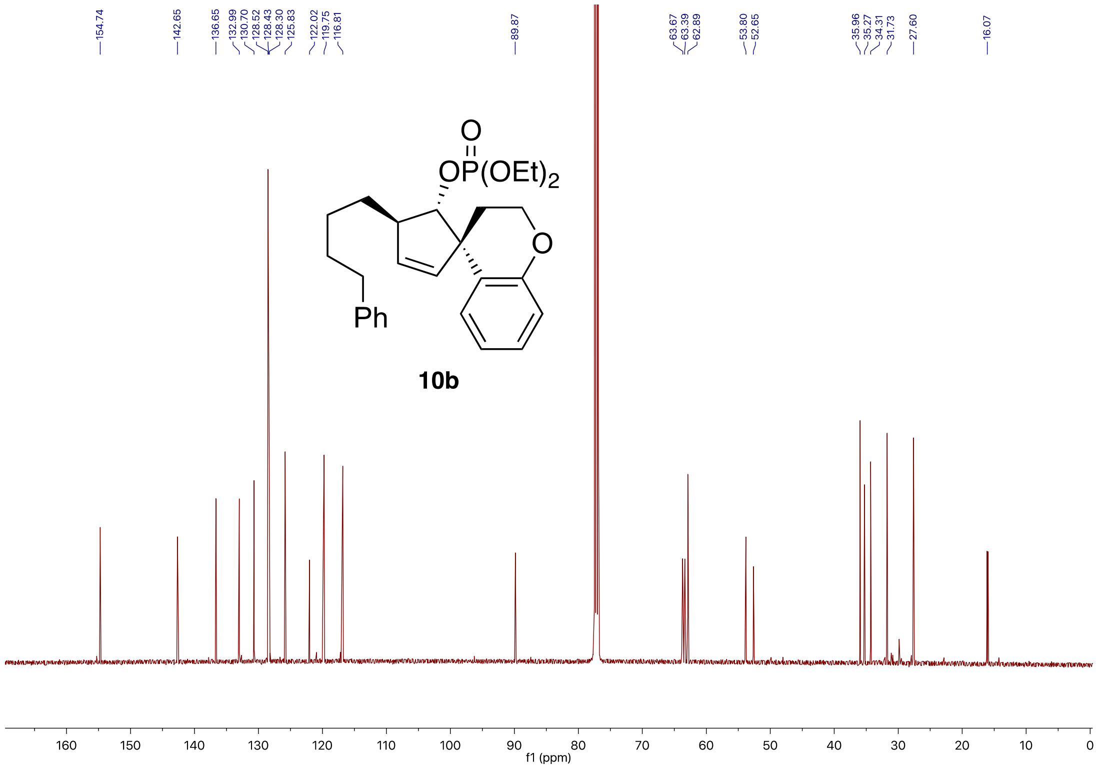

^{31}P NMR (162 MHz, $CDCl_3$)

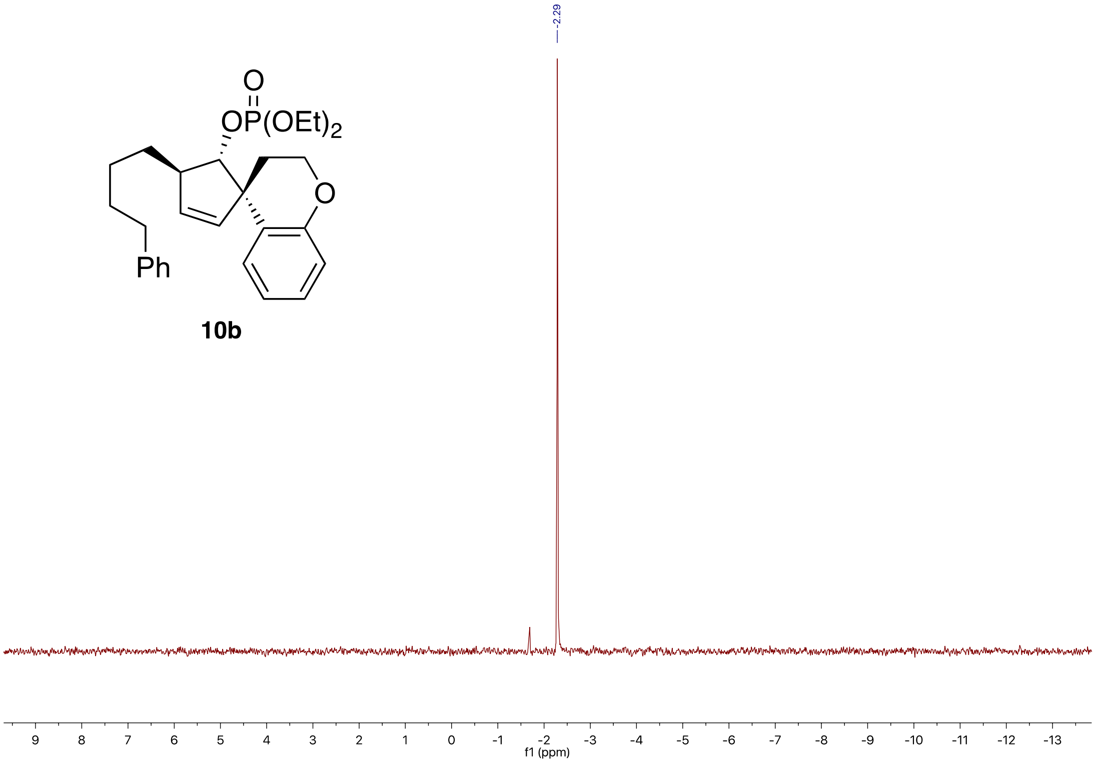

HPLC Trace

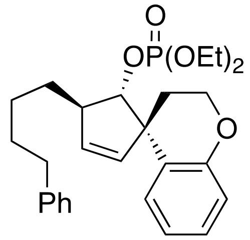

10b

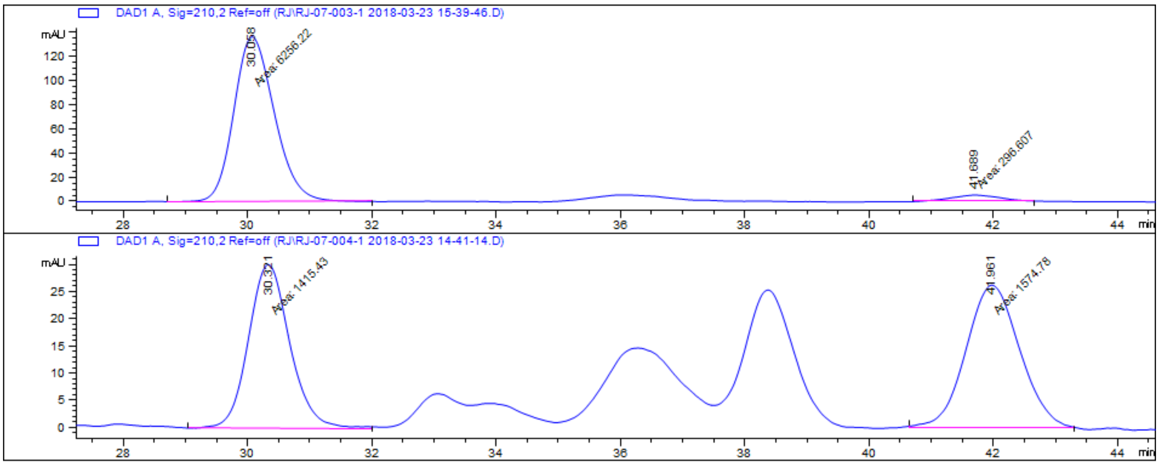

Supplementary Figure 63 - Diethyl ((3*S*,4*R*)-4-(4-phenylbutyl)-2,3,4,7-tetrahydrooxepin-3-yl) phosphate **(11b)** ^{1}H NMR (400 MHz, $CDCl_3$)

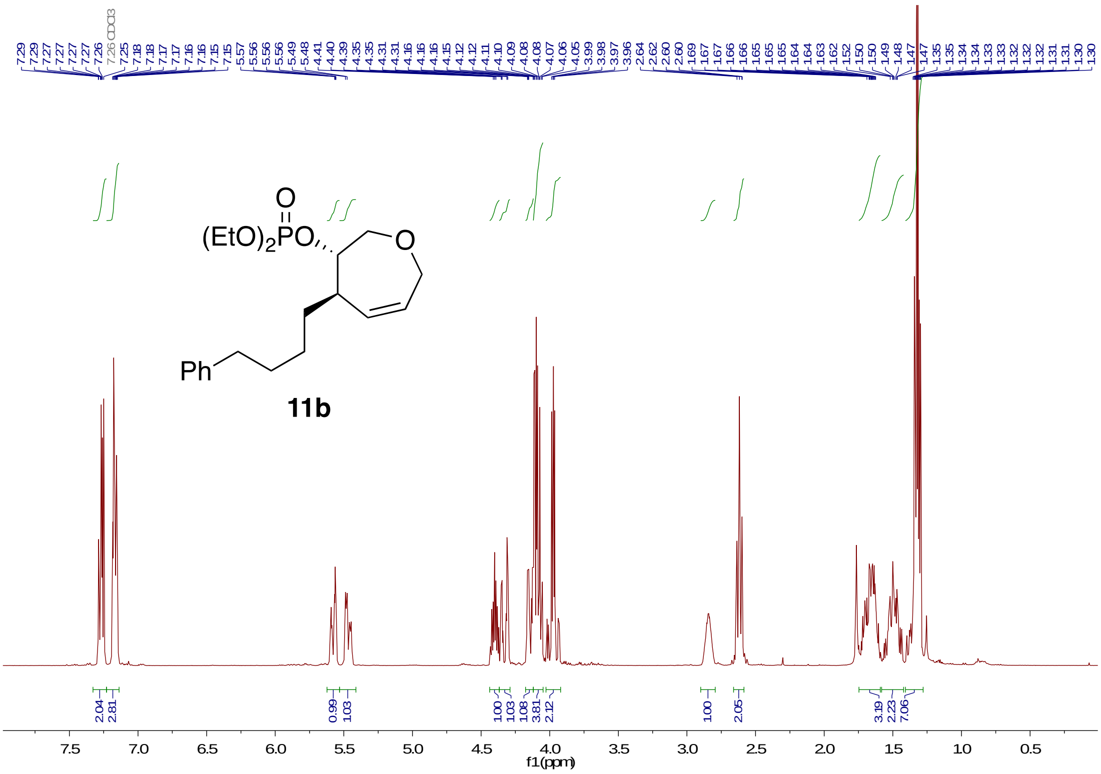

^{13}C NMR (101 MHz, $CDCl_3$)

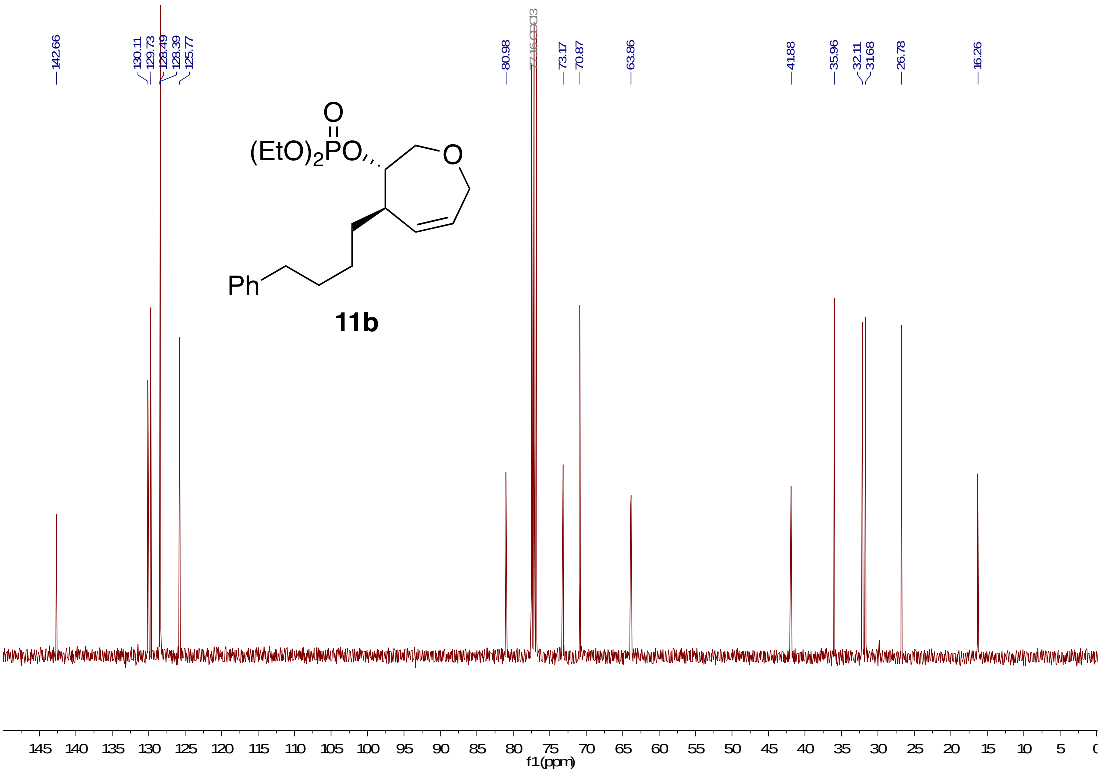

^{31}P NMR (162 MHz, $CDCl_3$)

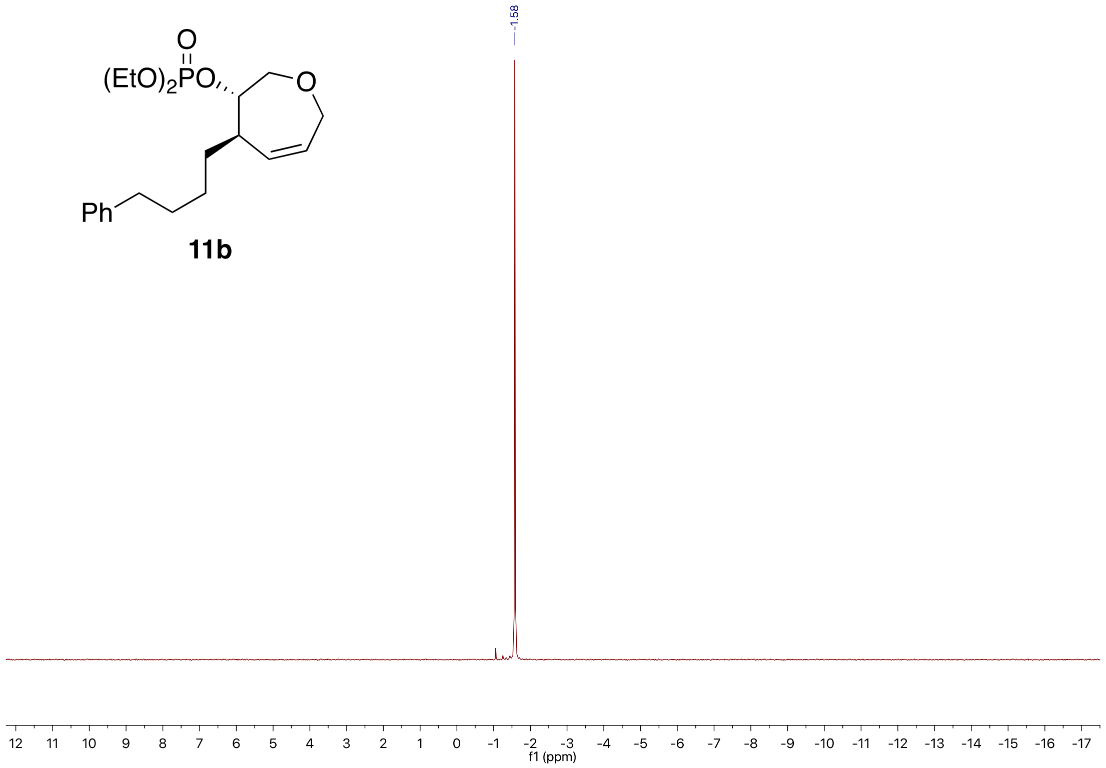

11b

SFC Trace

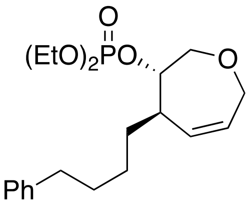

11b

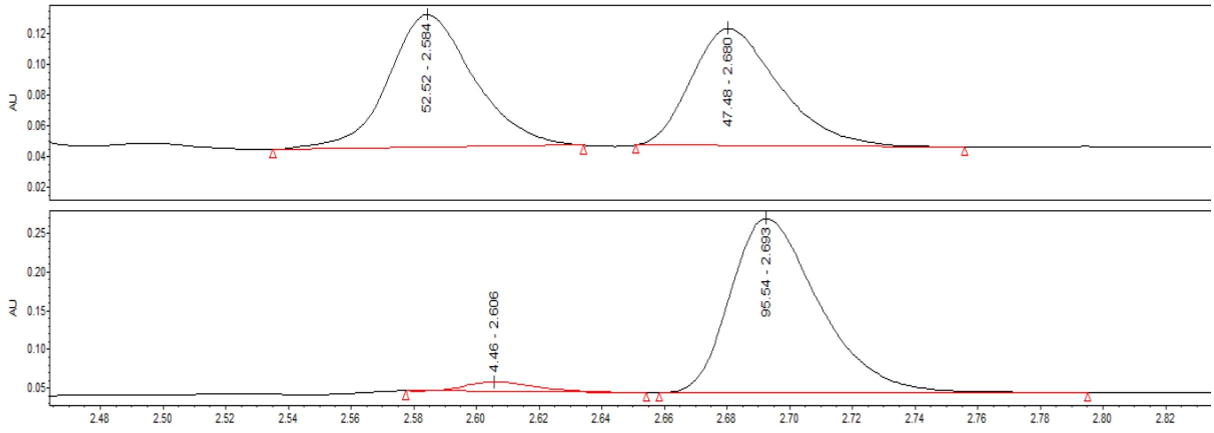

Supplementary Figure 64 - *tert*-Butyl (3*S*,4*R*)-3-((diethoxyphosphoryl)oxy)-4-(4-phenylbutyl)-2,3,4,7-tetrahydro-1*H*-azepine-1-carboxylate **(12b)** ^{1}H NMR (500 MHz, DMSO-d_6, 338K)

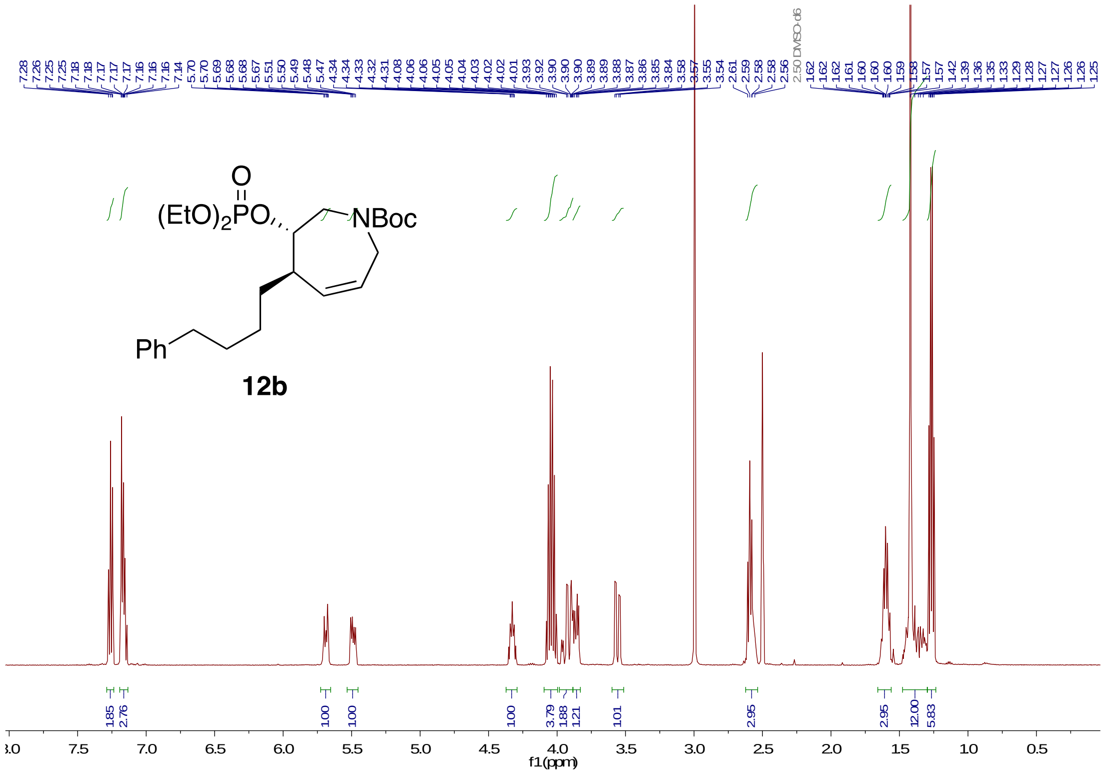

^{13}C NMR (101 MHz, $CDCl_3$)

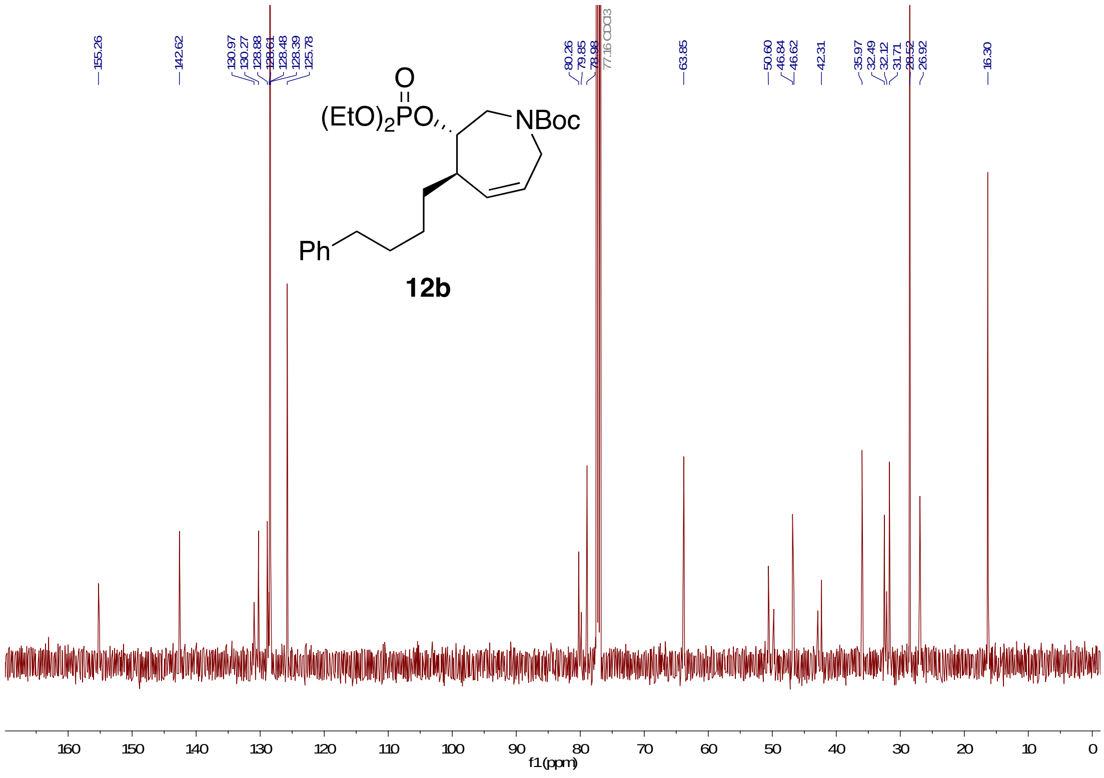

^{31}P NMR (162 MHz, $CDCl_3$)

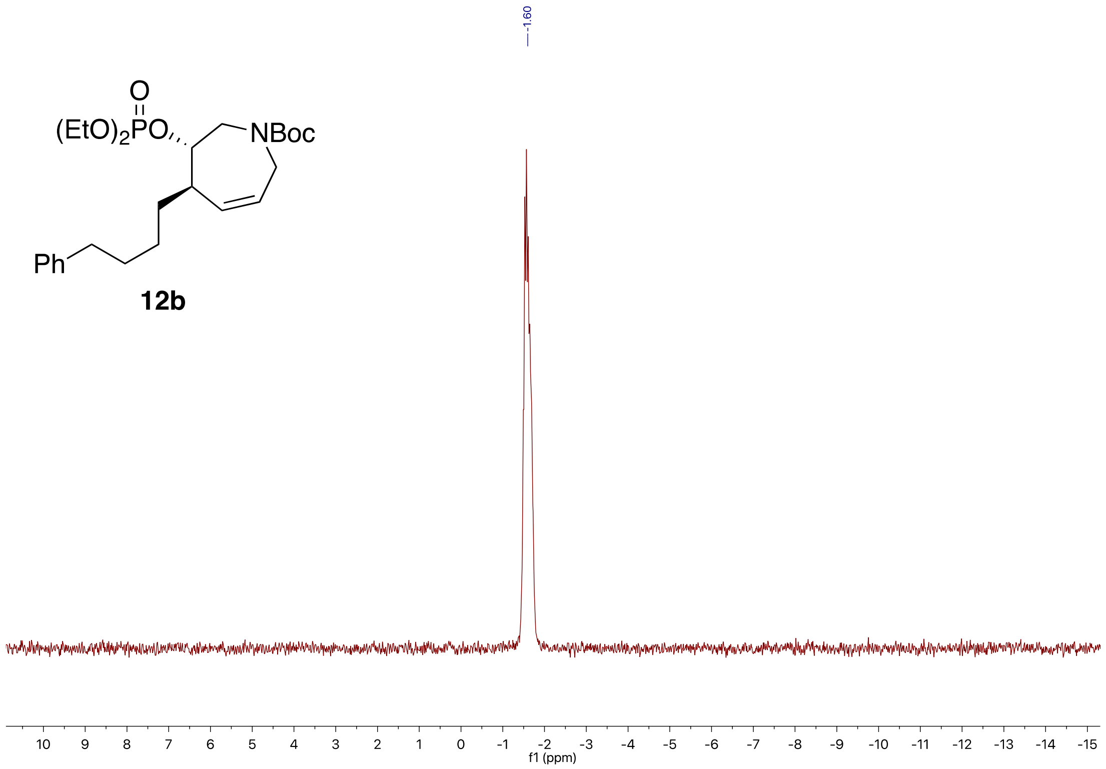

SFC Trace

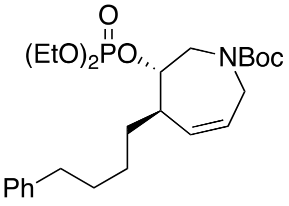

12b

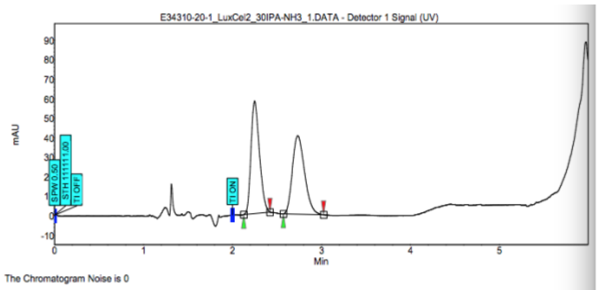

The Chromatogram Noise is 0

Results Table:

Index	Name	Start [Min]	Time [Min]	End [Min]	RT Offset [Min]	Quantity [% Area]	Height [µV]	Area [µV.Min]	Area [%]
1	UNKNOWN	2.13	2.25	2.42	0.00	49.81	57.6	6.3	49.813
2	UNKNOWN	2.57	2.73	3.02	0.00	50.19	40.2	6.4	50.187
Total						100.00	97.8	12.7	100.000

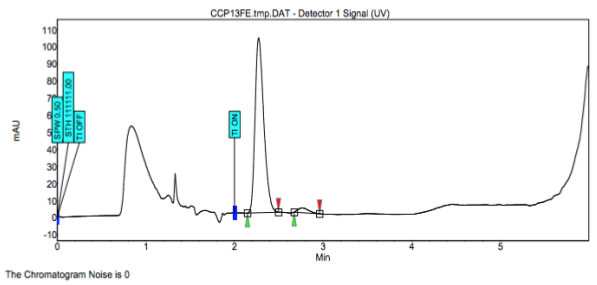

The Chromatogram Noise is 0

Results Table:

Index	Name	Start [Min]	Time [Min]	End [Min]	RT Offset [Min]	Quantity [% Area]	Height [µV]	Area [µV.Min]	Area [%]
1	UNKNOWN	2.15	2.27	2.50	0.00	96.73	102.3	11.7	96.735
2	UNKNOWN	2.67	2.77	2.96	0.00	3.27	2.8	0.4	3.265
Total						100.00	105.1	12.1	100.000

Supplementary Figure 65 - Diethyl ((1*S*,2*R*)-2-(4-phenylbutyl)cyclopent-3-en-1-yl) phosphate (**14**) ^{1}H NMR (500 MHz, CDCl$_3$)

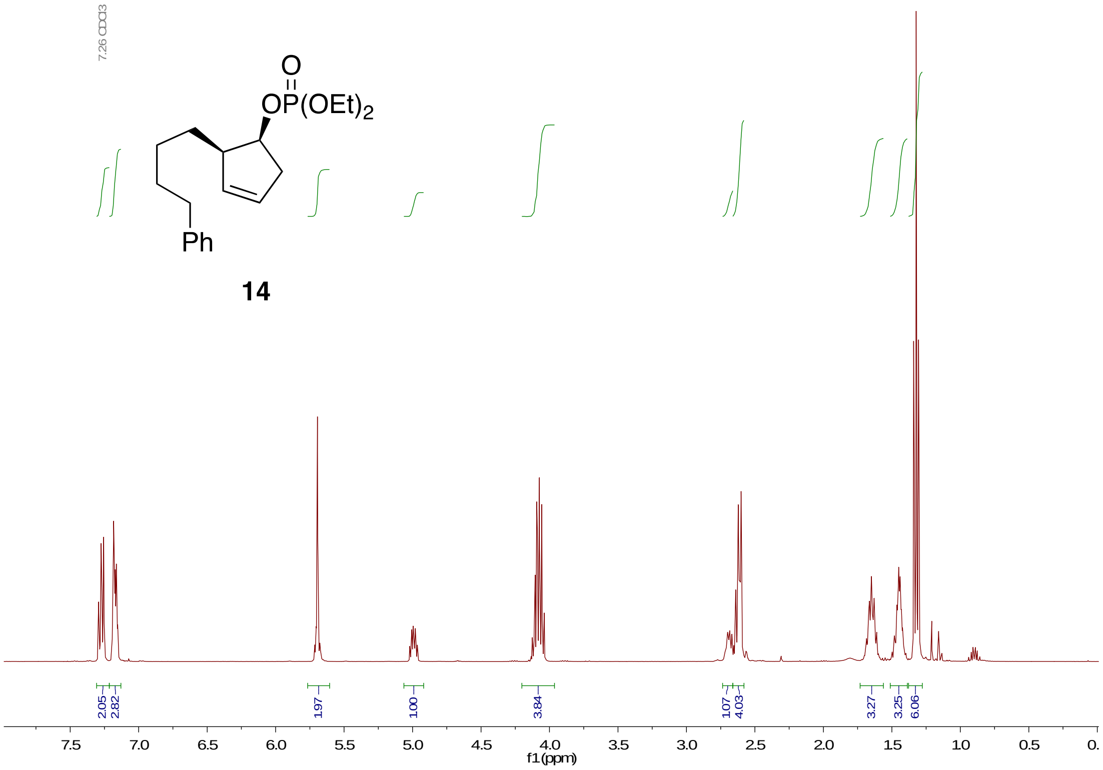

^{13}C NMR (162 MHz, CDCl$_3$)

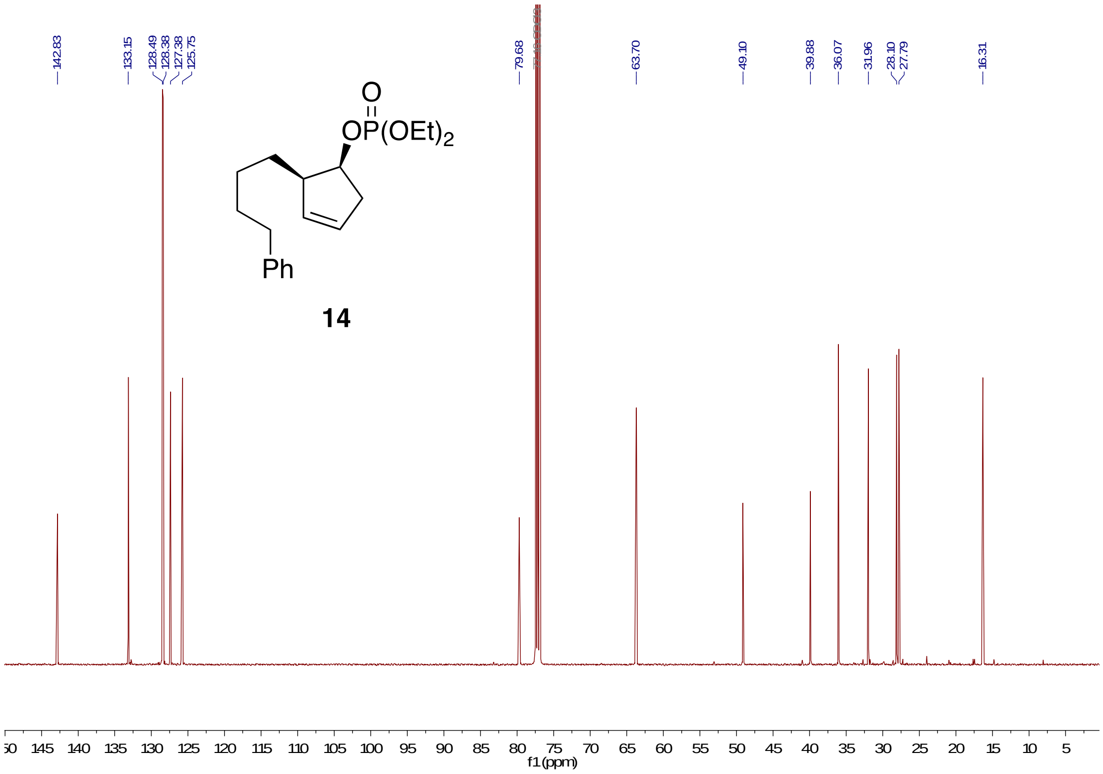

^{31}P NMR (162 MHz, CDCl$_3$)

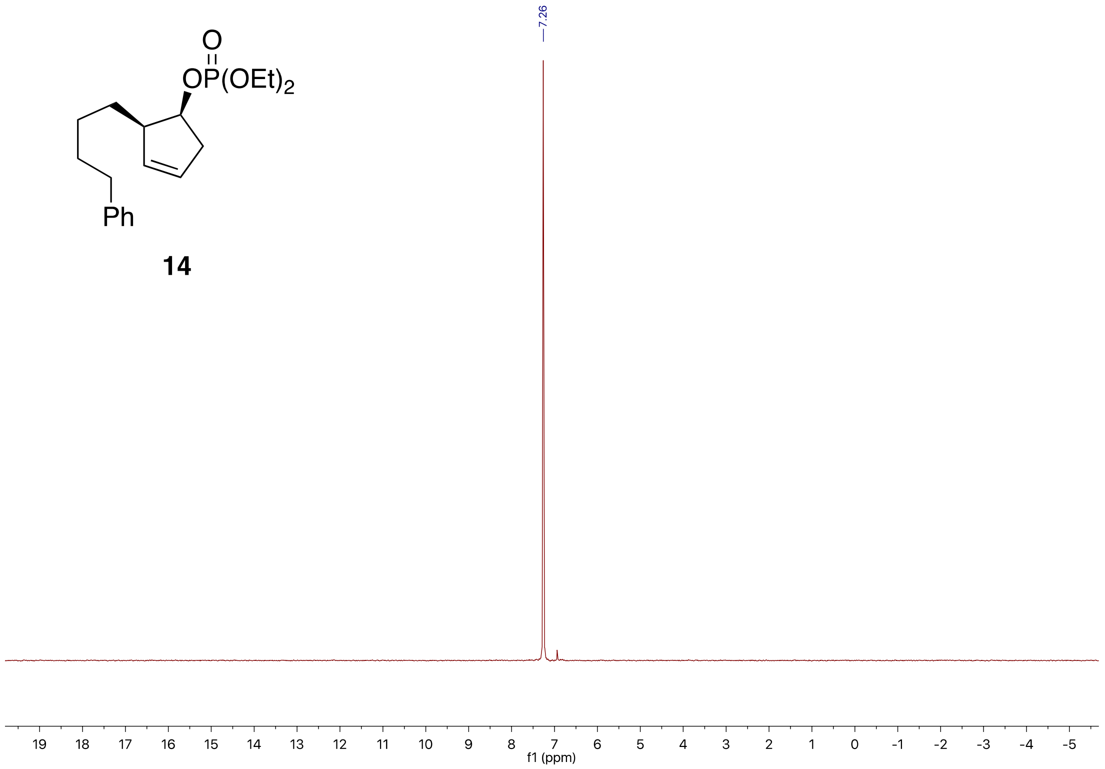

14

HPLC Trace

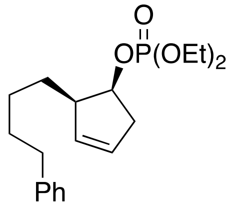

14

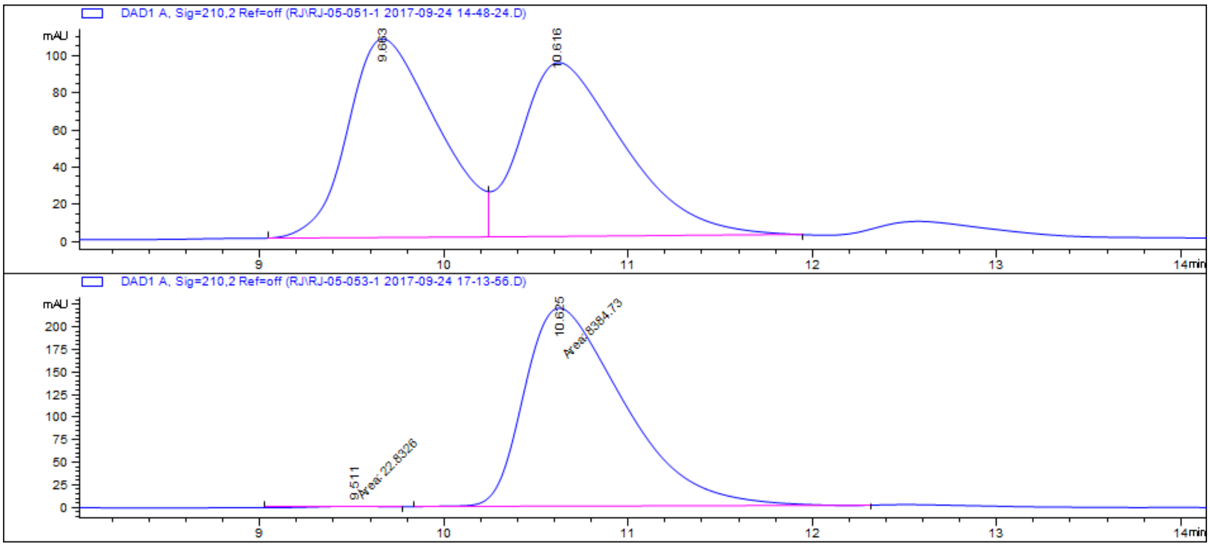

Supplementary Figure 66 - Diethyl ((1*R*,4a*R*,10a*R*)-1,4,4a,9,10,10a-hexahydrophenanthren-1-yl) phosphate (**16**) ^{1}H NMR (500 MHz, $CDCl_3$)

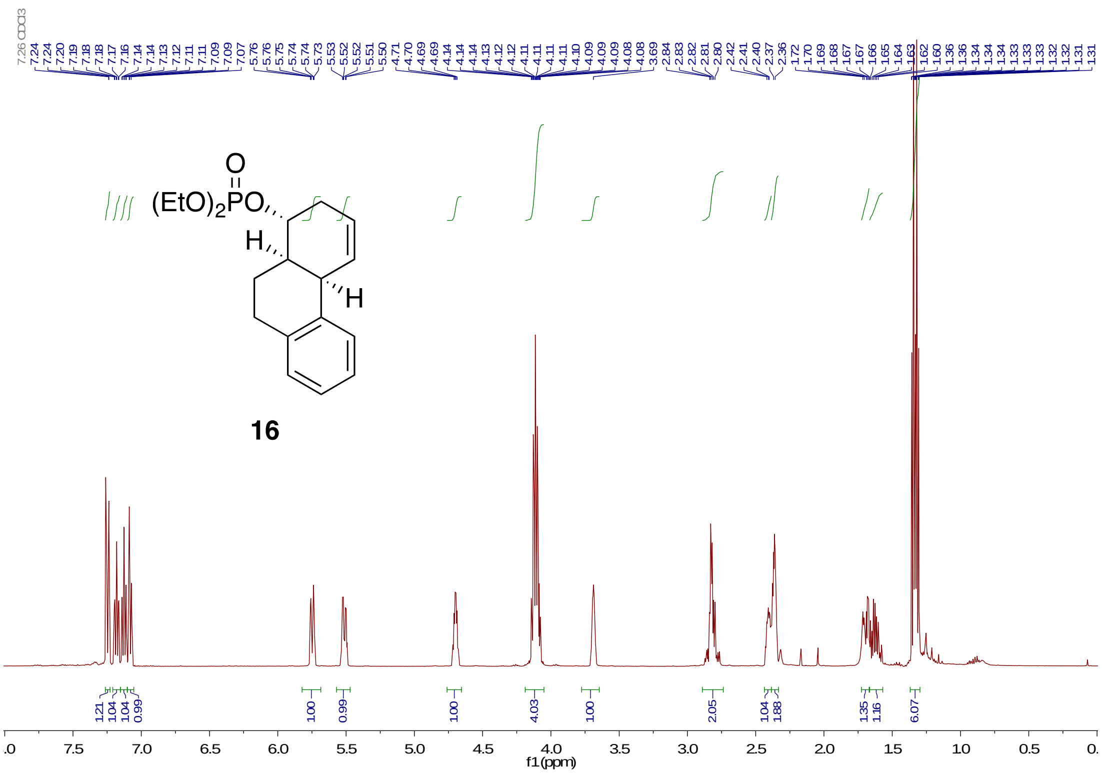

^{13}C NMR (126 MHz, $CDCl_3$)

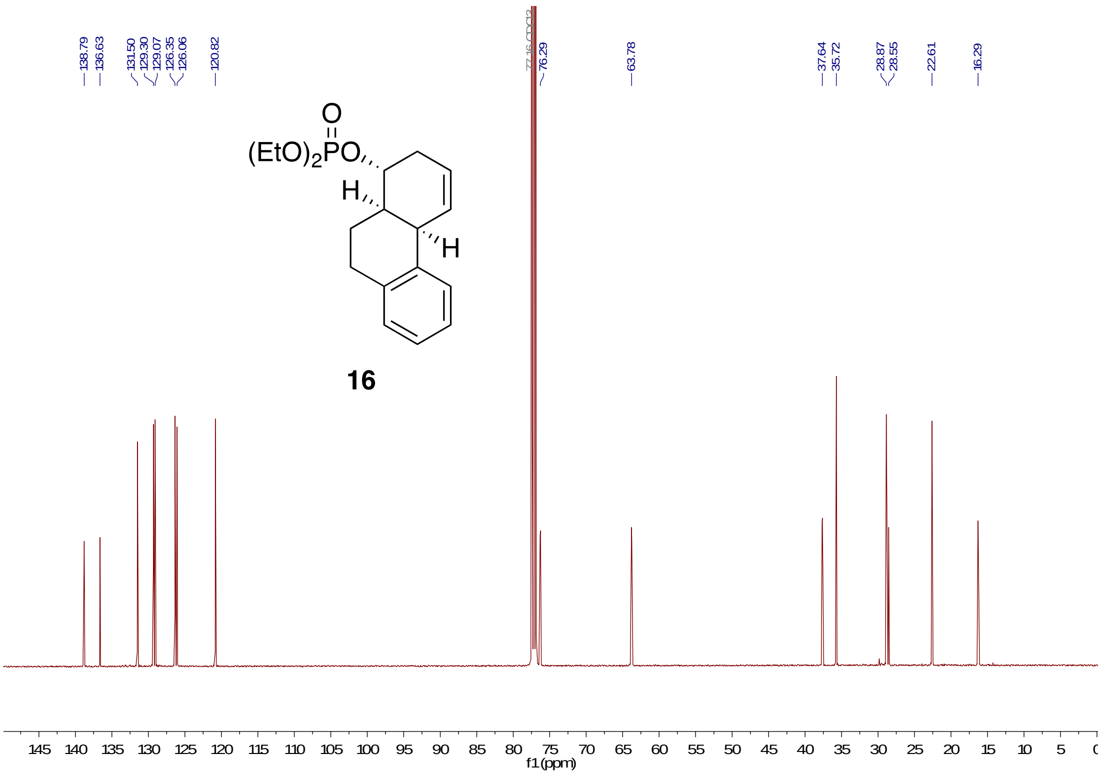

^{31}P NMR (162 MHz, $CDCl_3$)

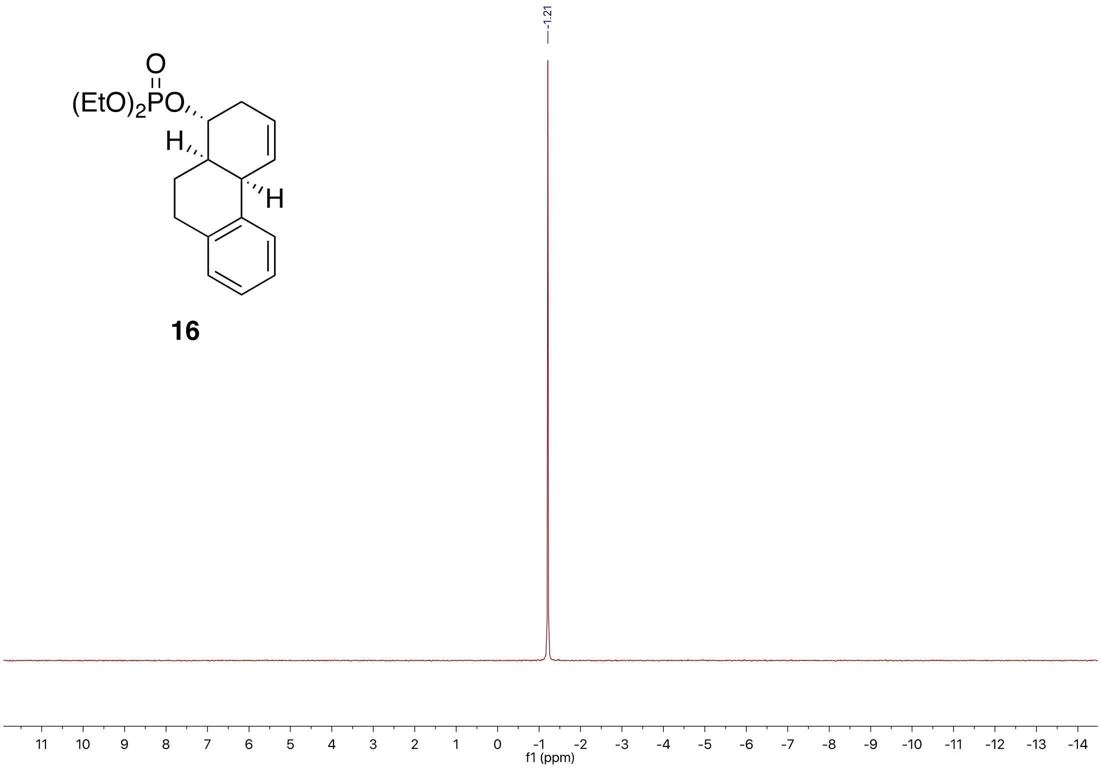

HPLC Trace

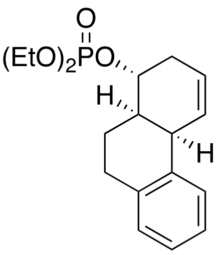

16

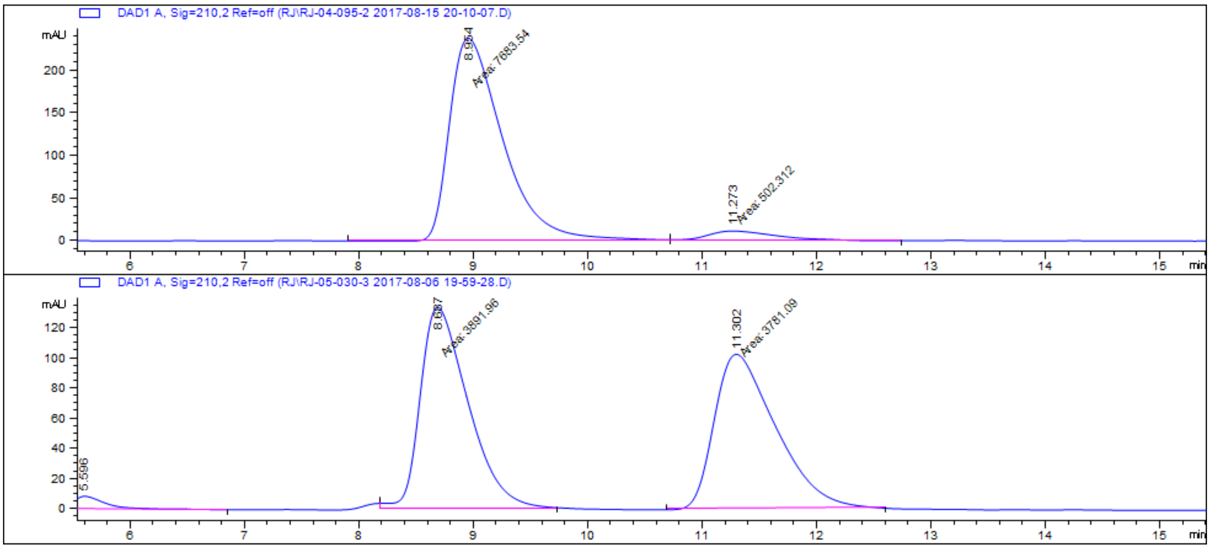

Supplementary Figure 67 - (1*S*,2*R*,3*R*,4*S*,5*R*)-2-benzyl-3,4-dihydroxy-2-methyl-5-(4-phenylbutyl)cyclopentyl diethyl phosphate **(17)** ^{1}H NMR (500 MHz, $CDCl_3$)

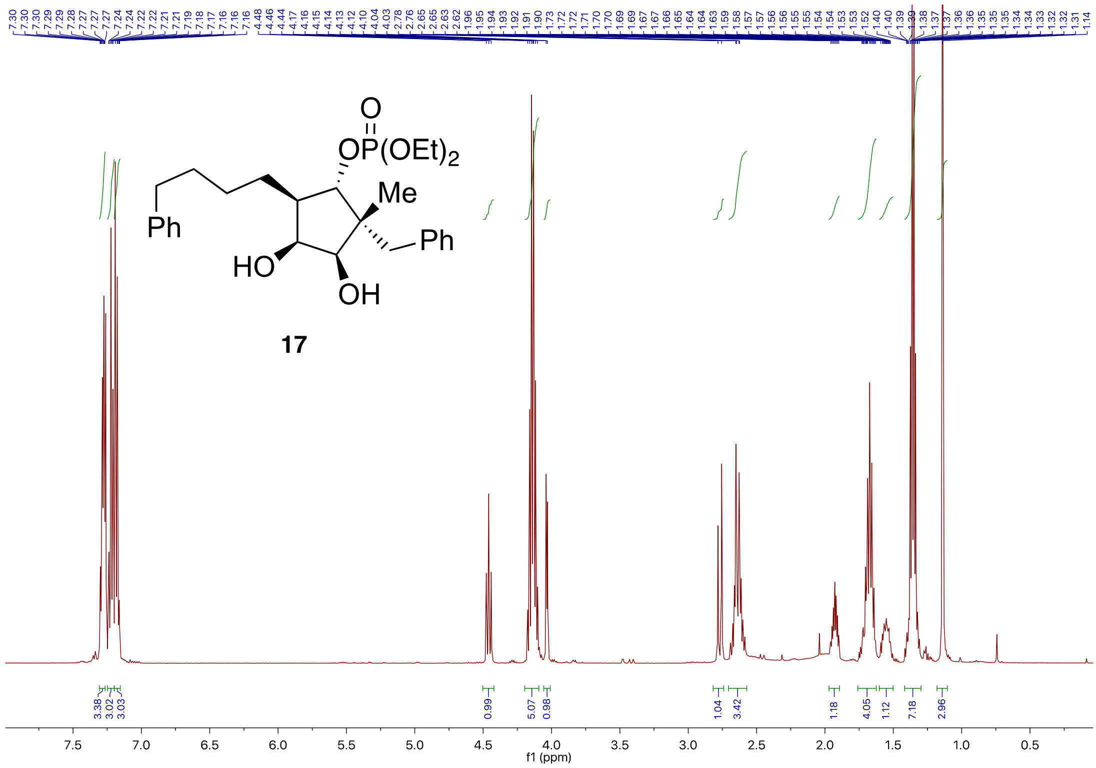

^{13}C NMR (126 MHz, $CDCl_3$)

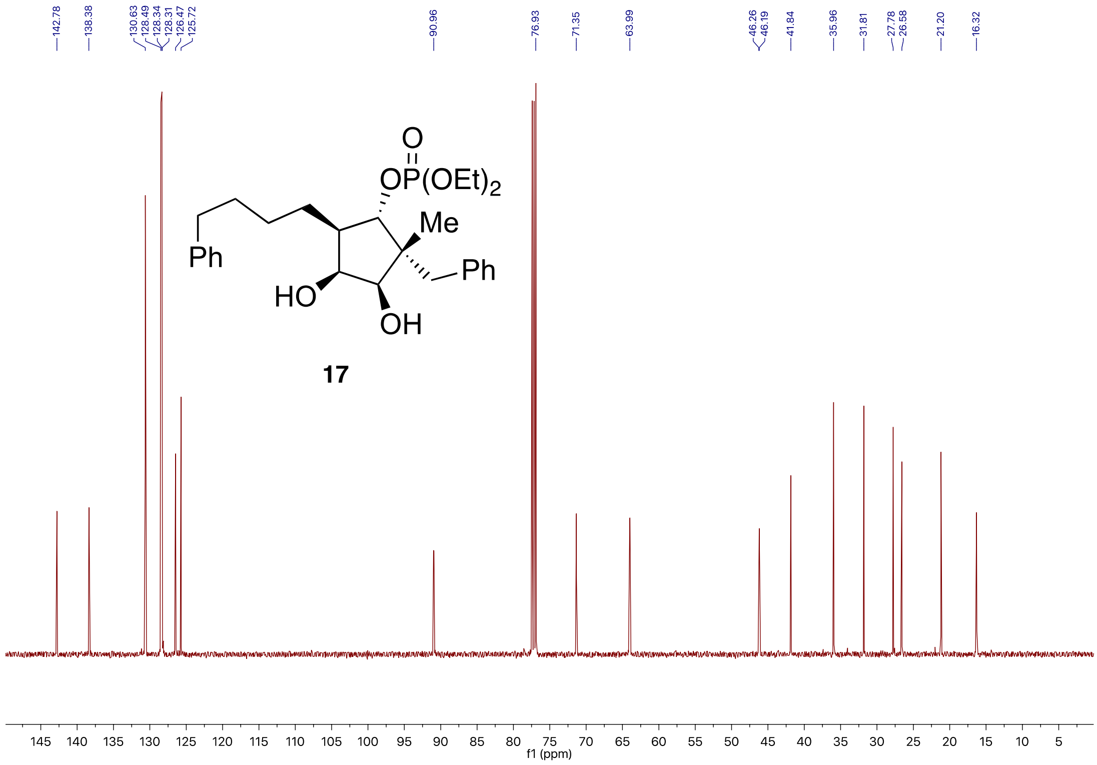

^{31}P NMR (162 MHz, $CDCl_3$)

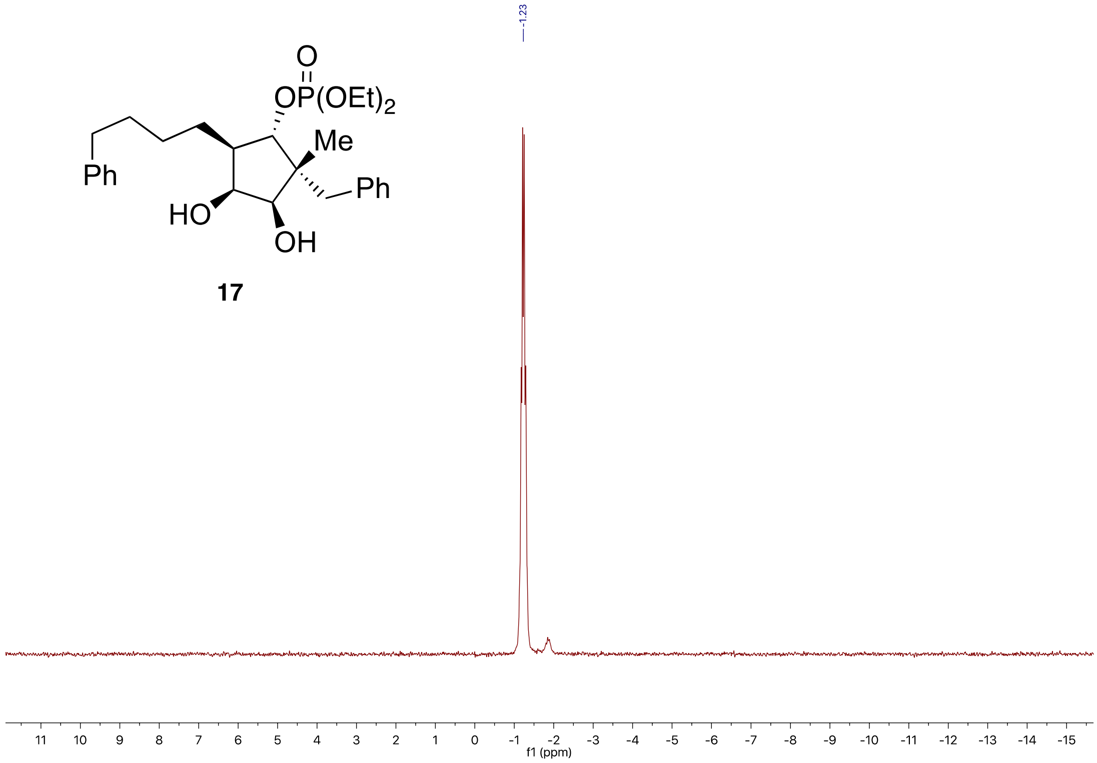

17

SFC Trace

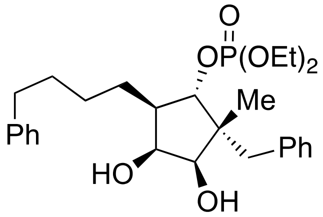

17

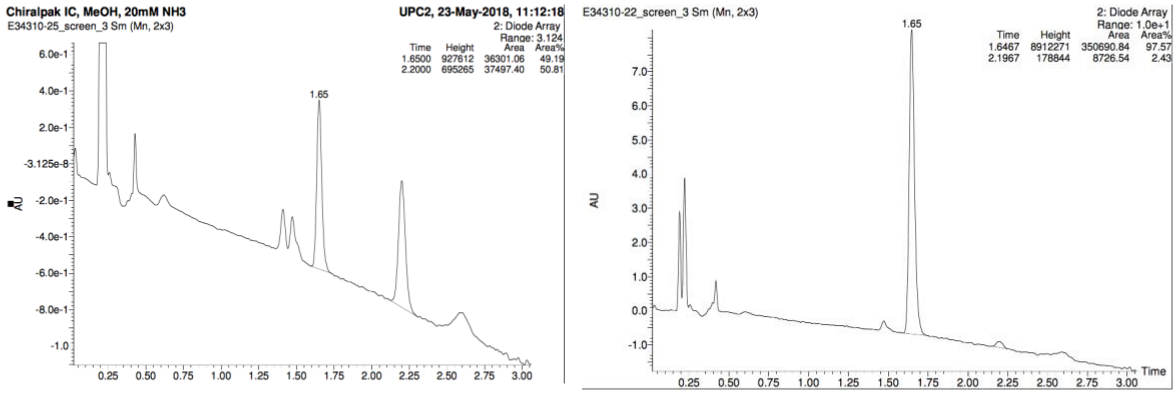

Supplementary References

[1] Buchwald, S. L., LaMaire, S. J. & Nielsen, R.B. *Org. Synth.* **71,** 77-82 (1993).

[2] Menard, F., Perez, D., Roman, D. S., Chapman, T. M. & Lautens, M. *J. Org. Chem.* **75,** 4056-4068 (2010).

[3] Feng, K., Wu, L., Zhang, L. & Tung, C. *Tetrahedron*, **63,** 4907-4911 (2007).

[4] Shiramizu, M. & Toste, D. F. *Angew. Chem. Int. Ed.* **52,** 12905-12909 (2013).

[5] Piarulli, U., Claverie, C., Daubos, P., Gennari, C., Minnaard, A. J. & Feringa, B. L. *Org. Lett.* **5,** 4493-4496 (2003).

[6] Holec, C., Sandkuhl, D., Rother, D., Kroutil, W. & Pietruszka, J. *ChemCatChem*. **7,** 3125-3130 (2015).

[7] Agosta, W. C. & Smith, A. B. *J. Org. Chem.* **35,** 3856-3860 (1970).

[8] Manna, M. S. & Mukherjee, S. *Chem. Sci.* **5,** 1627-1633 (2014).

[9] Aikawa, K., Okamoto, T. & Mikami, M. *J. Am. Chem. Soc.* **134,** 10329-10332 (2012).

[10] Crane, S. N. & Burnell, D. J. *J. Org. Chem*, **63,** 1352-1355 (1998).

[11] Brun, O., Agramunt, J., Raich, L., Rovira, C., Pedroso, E. & Grandas, A. *Org. Lett.* **18,** 4836-4839 (2016).

[12] Evans, J. C., Klix, R. C. & Bach, R. D. *J. Org. Chem*, **53,** 5519-5527 (1988).

[13] Miyaoka, H., Sagawa, S., Nagaoka, H. & Y. Yamada, Y. *Tetrahedron: Asymmetry*, **6,** 587-594 (1995).

[14] Ito, M., Matsuumi, M., Murugesh, M. G. & Kobayashi, Y. *J. Org. Chem,* **66,** 5881-5889 (2001).

[15] Brawn, R. A., Guimaraes, C. R. W., McClure, K. F. and Liras, S. *Org. Lett.*, **14**, 4802-4805 (2012)

[16] Hartman, G. D. and Kuduk, S. WO Patent Application, 2015109130 (2015)
